# Influence of puberty timing on adiposity and cardiometabolic traits: A Mendelian randomisation study

**DOI:** 10.1371/journal.pmed.1002641

**Published:** 2018-08-28

**Authors:** Joshua A. Bell, David Carslake, Kaitlin H. Wade, Rebecca C. Richmond, Ryan J. Langdon, Emma E. Vincent, Michael V. Holmes, Nicholas J. Timpson, George Davey Smith

**Affiliations:** 1 Medical Research Council Integrative Epidemiology Unit, Population Health Sciences, Bristol Medical School, University of Bristol, Bristol, United Kingdom; 2 School of Cellular and Molecular Medicine, University of Bristol, Bristol, United Kingdom; 3 Clinical Trial Service Unit & Epidemiological Studies Unit, Nuffield Department of Population Health, University of Oxford, Oxford, United Kingdom; 4 Medical Research Council Population Health Research Unit, University of Oxford, Oxford, United Kingdom; 5 National Institute for Health Research Oxford Biomedical Research Centre, Oxford University Hospital, Oxford, United Kingdom; Pennsylvania State University University Park: Penn State, UNITED STATES

## Abstract

**Background:**

Earlier puberty is widely linked with future obesity and cardiometabolic disease. We examined whether age at puberty onset likely influences adiposity and cardiometabolic traits independent of childhood adiposity.

**Methods and findings:**

One-sample Mendelian randomisation (MR) analyses were conducted on up to 3,611 white-European female and male offspring from the Avon Longitudinal Study of Parents and Children (ALSPAC) cohort recruited at birth via mothers between 1 April 1991 and 31 December 1992. Time-sensitive exposures were age at menarche and age at voice breaking. Outcomes measured at age 18 y were body mass index (BMI), dual-energy X-ray absorptiometry–based fat and lean mass indices, blood pressure, and 230 cardiometabolic traits derived from targeted metabolomics (150 concentrations plus 80 ratios from nuclear magnetic resonance [NMR] spectroscopy covering lipoprotein subclasses of cholesterol and triglycerides, amino acids, inflammatory glycoproteins, and others). Adjustment was made for pre-pubertal BMI measured at age 8 y. For negative control MR analyses, BMI and cardiometabolic trait measures taken at age 8 y (before puberty, and which therefore cannot be an outcome of puberty itself) were used. For replication analyses, 2-sample MR was conducted using summary genome-wide association study data on up to 322,154 adults for post-pubertal BMI, 24,925 adults for post-pubertal NMR cardiometabolic traits, and 13,848 children for pre-pubertal obesity (negative control). Like observational estimates, 1-sample MR estimates in ALSPAC using 351 polymorphisms for age at menarche (explaining 10.6% of variance) among 2,053 females suggested that later age at menarche (per year) was associated with −1.38 kg/m^2^ of BMI at age 18 y (or −0.34 SD units, 95% CI −0.46, −0.23; *P =* 9.77 × 10^−09^). This coefficient attenuated 10-fold upon adjustment for BMI at age 8 y, to −0.12 kg/m^2^ (or −0.03 SDs, 95% CI −0.13, 0.07; *P =* 0.55). Associations with blood pressure were similar, but associations across other traits were small and inconsistent. In negative control MR analyses, later age at menarche was associated with −0.77 kg/m^2^ of pre-pubertal BMI measured at age 8 y (or −0.39 SDs, 95% CI −0.50, −0.29; *P =* 6.28 × 10^−13^), indicating that variants influencing menarche also influence BMI before menarche. Cardiometabolic trait associations were weaker and less consistent among males and both sexes combined. Higher BMI at age 8 y (per 1 kg/m^2^ using 95 polymorphisms for BMI explaining 3.4% of variance) was associated with earlier menarche among 2,648 females (by −0.26 y, 95% CI −0.37, −0.16; *P =* 1.16 × 10^−06^), likewise among males and both sexes combined. In 2-sample MR analyses using 234 polymorphisms and inverse variance weighted (IVW) regression, each year later age at menarche was associated with −0.81 kg/m^2^ of adult BMI (or −0.17 SD units, 95% CI −0.21, −0.12; *P =* 4.00 × 10^−15^). Associations were weaker with cardiometabolic traits. Using 202 polymorphisms, later menarche was associated with lower odds of childhood obesity (IVW-based odds ratio = 0.52 per year later, 95% CI 0.48, 0.57; *P =* 6.64 × 10^−15^). Study limitations include modest sample sizes for 1-sample MR, lack of inference to non-white-European populations, potential selection bias through modest completion rates of puberty questionnaires, and likely disproportionate measurement error of exposures by sex. The cardiometabolic traits examined were heavily lipid-focused and did not include hormone-related traits such as insulin and insulin-like growth factors.

**Conclusions:**

Our results suggest that puberty timing has a small influence on adiposity and cardiometabolic traits and that preventive interventions should instead focus on reducing childhood adiposity.

## Abstract 2

In a mendelian randomization study, Joshua Bell and colleagues investigate possible health implications of the age of puberty.

## Author summary

**Why was this study done?:**

- People who enter puberty at younger ages are more likely to develop obesity and cardiometabolic diseases in adulthood, but whether this is because of puberty timing itself is unknown.
- Childhood adiposity (fatness) may induce earlier puberty and may also track forward into adulthood, making childhood adiposity an important potential confounder. Most prior studies of puberty timing as a risk factor for adult obesity and cardiometabolic dysfunction did not consider childhood adiposity and used crude measures of cardiometabolic traits. They also relied upon observational methods, which are prone to wider confounding.

**What did the researchers do and find?:**

- We used naturally occurring genetic variation in age at menarche to examine whether puberty timing itself is likely to influence adiposity and cardiometabolic traits.
- Using data from >3,600 offspring from a multigenerational British birth cohort study, we examined genetically proxied age at menarche/voice breaking in relation to body mass index, objective fat and lean mass indices, blood pressure, and over 200 detailed traits from targeted metabolomics. These outcome traits were all measured at age 18 y (after puberty onset) and at age 8 y (before puberty onset), which allowed us to examine the effects of puberty timing while accounting for outcome trait levels in childhood.
- We found that apparent effects of later puberty onset on lower adiposity and blood pressure at age 18 y were largely attenuated when accounting for adiposity at age 8 y. Effects on other cardiometabolic traits were small and inconsistent. We also found a strong effect of higher adiposity at age 8 y on earlier puberty onset.

**What do these findings mean?:**

- Our findings support puberty timing as a marker, not a driver, of adiposity and cardiometabolic trait levels.
- These findings suggest that interventions to prevent adult obesity and cardiometabolic disease should focus on childhood adiposity, not puberty timing.

## Introduction

The onset of puberty is pivotal for human growth and development [1–3]. Beyond immediate effects on physical and sexual maturity, however, earlier onset of puberty is widely linked with adverse health outcomes in adulthood. These include greater risk of obesity, hyperinsulinemia, hyperlipidaemia [4], type 2 diabetes [5], hypertension [4], coronary heart disease [6], and early mortality [7]. Whether puberty timing is a concern for cardiometabolic health in populations, however, depends on whether it is truly causal. Higher adiposity in childhood may induce an earlier puberty [8] and track forward into adulthood [9–13], making it an important potential confounder. Prospective studies of puberty timing in relation to cardiometabolic outcomes tend not to account for differences in pre-pubertal adiposity [14,15], and the few studies that do often find substantial attenuation of puberty–outcome associations [4,16,17]. No studies to our knowledge have yet examined puberty timing in relation to detailed traits from targeted metabolomics in adulthood, and, importantly, evidence has so far been observational, with inherent susceptibilities to residual confounding.

Direct manipulation of puberty timing to examine its cardiometabolic effects within a randomised controlled trial setting would be costly, time intensive, and potentially unethical, and such interventions have not been performed on non-human mammals. Advances in human population genetics do, however, allow us to employ Mendelian randomisation (MR)—an instrumental variable method that exploits the random assignment of exposure-associated risk alleles. This method is by nature less prone to confounding and reverse causation bias, which limit causal inference from observational data [18–20]. Nearly 400 common genetic variants so far associate strongly and independently with age at menarche among females [21]. Nearly half of these variants also associate with age at voice breaking among males, corroborating functional work that indicates a heavily overlapping genetic and molecular architecture of puberty timing between sexes [1,21,22]. Of concern, however, is the high degree of genetic overlap between puberty timing and adiposity [21], which challenges our ability to examine effects of puberty timing variants that operate exclusively through puberty timing—a core assumption of MR. Multivariate MR methods exist [23] but carry risk of inducing serious bias in this instance through stratification on a potential mediator (post-pubertal adiposity) [24]. Given the time-sensitive nature of pubertal age, it would be advantageous to account for a pre-pubertal measure of adiposity within an MR setting as this may help to further address confounding.

This study aimed to determine whether puberty timing is likely to have a distinct influence on adiposity and cardiometabolic traits in adulthood. First, we used data from a British birth cohort study (the Avon Longitudinal Study of Parents and Children [ALSPAC]) to examine observational estimates of age at menarche among females in relation to adiposity and a large set of cardiometabolic traits measured at age 18 y, accounting for pre-pubertal body mass index (BMI) measured at age 8 y. We then examined 1-sample MR estimates of age at menarche in relation to adiposity and cardiometabolic traits, also accounting for pre-pubertal BMI. Data from international genome-wide association study (GWAS) consortia were used for replication of MR estimates.

## Methods

### Study population

Data on offspring from ALSPAC were used for the main analyses. ALSPAC is a population-based birth cohort study in which 14,541 pregnant women with an expected delivery date between 1 April 1991 and 31 December 1992 were recruited from the former Avon county of southwest England. Since then, the mothers and their offspring (*N =* 13,988 who were alive at 1 y) have been followed repeatedly with questionnaire- and clinic-based assessments [25], with an additional 713 children enrolled over the course of the study. Non-sibling participants were considered for the present analyses (excluding 202 non-first-borns). A further 604 participants of a non-white ethnicity were excluded to minimise the confounding of associations by ancestral population structure. Participants provided written informed consent, and ethical approval was obtained from the ALSPAC Law and Ethics Committee and the local research ethics committee. Cohort details and data descriptions are publicly available (http://www.bristol.ac.uk/alspac/researchers/access).

### Prespecified study protocol

A study protocol was written in December 2016 for an ALSPAC data proposal prior to analyses (S1 Protocol) as part of wider investigations into puberty timing and cancer. Analyses that were not prespecified but added following peer review included 1-sample MR analyses of BMI with summary cardiometabolic trait outcomes for sample power comparisons, funnel plots of polymorphisms for age at menarche in relation to adult adiposity and blood pressure to assess heterogeneity, multivariate MR of age at menarche and childhood BMI in relation to adult adiposity and blood pressure to compare estimates with main results, and 2-sample MR of the effect of age at menarche on childhood obesity.

### Assessment of puberty timing

Among females, puberty timing was indicated by the age (in years) at which menarche occurred. Data were reported by females through questionnaires distributed annually from the ages of 8 y to 17 y, covering the expected duration of puberty. The earliest report of age at menarche was used for analyses when several were available, assuming this to be closer to the event and thus most accurate. Among males, puberty timing was indicated by the age (in years) at which voice breaking occurred. Items from annual questionnaires asked whether voice changes had partially or totally occurred by the time of assessment but not the specific age of occurrence; we therefore estimated age at voice breaking by taking the age at which any stage of voice breaking (partial or total) was first reported and subtracting 6 months from this age to represent the midpoint between this occasion of first report and the occasion immediately prior. In cases where data on this most recent prior occasion were missing yet data on an older occasion were not, this subtraction was increased to 12 months, 24 months, etc., to reflect the midpoint between the occasion of first report and the most recent prior occasion known.

### Assessment of genotype and genetic instruments

Genotype was assessed using the Illumina HumanHap550 quad chip platform. After quality control through exclusion of participants with sex mismatch, minimal or excessive heterozygosity, disproportionately missing data, insufficient sample replication, cryptic relatedness, and non-European ancestry, 500,527 single nucleotide polymorphisms (SNPs) were measured directly. Imputation using the 1000 Genomes reference panel from the Impute2 repository resulted in coverage of 8,099,747 SNPs after further quality control.

An instrument for puberty timing was constructed within a 1-sample MR framework [18,26,27] using genetic variants robustly and independently associated with age at menarche in the largest GWAS to date, which meta-analysed imputed genomic scans of self-reported age at menarche (in years, unadjusted for BMI) from 329,345 post-pubertal women from 42 cohort studies [21]. A total of 389 independent variants reached a *P*-value threshold for genome-wide significance (*P <* 5 × 10^−8^) in a discovery sample; these were largely replicated in a separate sample of 39,543 post-pubertal women from the Icelandic deCODE study, explaining 7.4% of the variance in age at menarche in the replication sample. Of these 389 variants, 363 were SNPs (not deletion/insertion polymorphisms). Twelve of these SNPs were not available in ALSPAC imputed genotype files, leaving 351 SNPs for instrumentation. A genetic risk score (GRS) was constructed using PLINK 1.9 software, specifying the effect (age at menarche–raising) allele and beta coefficients from the source GWAS as external weightings. Scoring was done by multiplying the number of effect alleles (or probabilities of effect alleles if imputed) at each SNP (0, 1, or 2) by its weighting, summing these, and dividing by the total number of SNPs used. The score therefore reflects the average per-SNP effect on age at menarche. Among 1,831 females eligible for analyses in ALSPAC, this score was associated with measured age at menarche (*P =* 1.87 × 10^−46^), explaining 10.6% of the variance (*F =* 216.8). It was less strongly associated with age at voice breaking among 1,312 males (*P =* 0.01), explaining 0.50% of the variance (*F =* 6.5). Among 3,143 females and males combined, this score was associated with age at puberty onset (*P =* 2.18 × 10^−18^), explaining 2.4% of the variance (*F =* 77.5). This GRS was also directly associated with BMI at age 18 y among females (*P =* 3.44 × 10^−06^), among males (*P =* 0.002), and among both sexes combined (*P =* 1.37 × 10^−07^).

A refined GRS for puberty timing was also considered based on SNPs associated genome-wide with age at menarche that were also associated (at *P <* 0.05) with age at voice breaking (measured in years) in a separate sample of 55,871 males in the 23andMe study [21], to better represent exposure among males. Of 119 SNPs, 115 were available in ALSPAC imputed genotype files for instrumentation. This refined GRS was similarly associated with age at voice breaking among males as the full GRS (*P =* 0.01) and explained a similarly low amount of variance (0.58%, *F =* 7.7). Its *P*-value for direct association with BMI at age 18 y was 0.11.

A GRS for BMI was also constructed using SNPs associated genome-wide with adult BMI from the most recent meta-analysis of the Genetic Investigation of Anthropometric Traits (GIANT) consortium, which comprises 322,154 men and women from 114 cohort studies [28], using the same methods described above. Of 97 SNPs, 95 were available in ALSPAC imputed genotype files for instrumentation. Among 3,171 females and males in ALSPAC, this score was associated with BMI measured at age 8 y (*P =* 6.62 × 10^−26^), explaining 3.44% of the variance (*F =* 112.8).

### Assessment of adiposity and cardiometabolic traits

Height and weight were measured while in light clothing and without shoes during each clinic visit from age 3 y onwards; data from approximate ages of 8 y and 18 y were used in the present analyses. Weight was recorded to the nearest 0.1 kg using a Tanita scale, and height to the nearest 0.1 cm using a Harpenden stadiometer. BMI was calculated based on weight (in kilograms) divided by the square of height (in meters). Participants also underwent body composition scanning with dual-energy X-ray absorptiometry using a Lunar Prodigy narrow fan beam densitometer, from which estimates of total body fat and lean mass (in kilograms) were obtained. Fat mass index and lean mass index were derived in the same way as for BMI (in kg/m^2^).

Systolic and diastolic blood pressure (SBP and DBP, respectively) were additionally examined twice in succession while seated and at rest, with the arm supported, using a cuff and DINAMAP 9301 device; the mean of these 2 measures represented resting blood pressure. Blood samples were drawn during these same 2 clinic visits, the age 8 y sample while not fasting and the age 18 y sample while fasting (the metabolic trait concentrations included in the study have shown stability over different durations of fasting time [29]). Proton nuclear magnetic resonance (NMR) spectroscopy as part of a targeted high-throughput metabolomics platform [30] was performed on both blood samples to quantify 230 cardiometabolic traits (150 concentrations plus 80 ratios) comprising lipoprotein cholesterol subclass particle concentrations and sizes, glycerides and phospholipids, apolipoproteins, fatty acids, glycolysis-related factors, amino acids, ketone bodies, and factors related to fluid balance and inflammation.

### Assessment of covariates

Covariates considered were basic demographic variables of participant age (in months) at the time of each outcome trait assessment and the highest level of education attained by their mother as answered by her through questionnaire after birth (grouped based on English standards as Certificate of Secondary Education, vocational, O level, A level, or degree) as an indicator of childhood socioeconomic position. Health behaviour covariates were not considered due to individual differences in exposure timing.

### Statistical analyses

**Fig 1:**
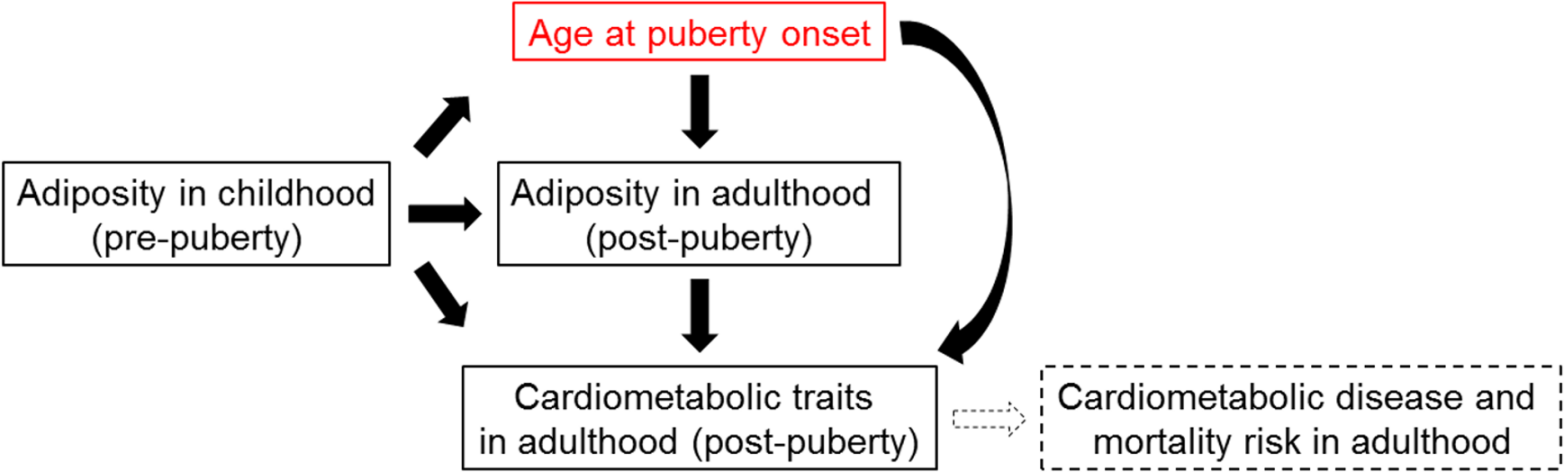
Conceptual framework of this study.

The conceptual framework of this study is outlined in Fig 1.

In observational analyses, linear regression models were used to examine age at menarche/voice breaking (in years) separately among females and males in relation to the dependent variables BMI, fat mass index, lean mass index, SBP, DBP, and other cardiometabolic traits, measured at approximately age 18 y. Outcomes were standardised (mean = 0, SD = 1) to allow comparability of effect sizes, and robust standard errors were used to accommodate skewed outcome distributions [31]. The following models were constructed: (i) adjusted for age (in months) at outcome assessment and maternal education; (ii) adjusted for age, maternal education, and BMI measured at age 8 y, to examine whether puberty timing is associated with outcomes at age 18 y independent of pre-pubertal BMI; and (iii) adjusted for age, maternal education, and the age 8 y value of the specific outcome trait being examined at age 18 y, to examine whether puberty timing is associated with post-pubertal outcomes independent of pre-pubertal values of those same traits. Observational analyses were repeated among females and males combined.

One-sample MR analyses were first conducted among females only, given the greater strength of age at menarche instruments. Two-stage least squares (2SLS) regression models with robust standard errors were used to examine the full GRS for age at menarche (351 SNPs with external weightings) as an instrument for age at menarche in relation to the outcomes of BMI, fat mass index, lean mass index, SBP, DBP, and other cardiometabolic traits at age 18 y. The following models were constructed: (i) unadjusted; (ii) adjusted for measured BMI at age 8 y (in both first- and second-stage models), to examine whether instrumented age at menarche is associated with outcomes at age 18 y independent of pre-menarche BMI; and (iii) adjusted for the age 8 y value of the specific outcome trait being examined at age 18 y, to examine whether instrumented age at menarche is associated with post-menarche outcomes independent of pre-menarche values of those specific traits. These models were repeated among females and males combined (based on age at menarche/voice breaking, respectively) to examine whether greater sample size improves the precision of results despite reduced instrument strength. Models were also repeated using the refined GRS for age at menarche (115 SNPs also associated with age at voice breaking among males) among males only.

In the second set of 1-sample MR analyses, 2SLS models with robust standard errors were used to examine the full GRS for age at menarche as an instrument for age at menarche among females in relation to BMI, fat mass index, lean mass index, SBP, DBP, and other cardiometabolic traits measured at age 8 y (before menarche). This analysis served as a negative control analysis intended to illustrate the extent of pleiotropy within the genetic instrument, given that any associations of instrumented age at menarche with traits measured before menarche cannot be generated by age at menarche itself (because this is temporally implausible). These models were repeated on females and males combined.

In the third set of 1-sample MR analyses, 2SLS models with robust standard errors were used to examine the GRS of 95 SNPs for BMI as an instrument for BMI at age 8 y in relation to age at puberty onset among females, among males, and among both sexes combined.

All analyses described above were performed on unrestricted samples of participants (sample sizes varying across models). To examine the extent to which potentially non-randomly missing data biased results, all models were repeated on complete case samples of participants with all relevant data.

To gauge the adequacy of ALSPAC sample power to generate reliable 1-sample MR estimates, we examined estimates for BMI at age 8 y and 18 y (each standardised, given dissimilar variance over time) in relation to standardised summary cardiometabolic traits at age 8 y and 18 y. Strong evidence of association is expected for BMI at least in relation to SBP, DBP, and triglycerides, based on known effects among young adults [12], if the sample is adequately powered.

For comparison with 1-sample MR estimates based on measured pre-pubertal BMI adjustments, we performed multivariate MR analyses of age at menarche in relation to adiposity and blood pressure at age 18 y using genetically predicted trait adjustments in ALSPAC. For unadjusted models, genetically predicted age at menarche and childhood BMI were first derived by regressing each exposure trait with its externally weighted GRS as an independent variable (using the same SNP inclusion criteria as prior). Predicted values from first-stage regressions were then used as independent variables in relation to outcome traits in second-stage models. For multivariate adjusted models, genetically predicted age at menarche and childhood BMI were first derived by regressing each exposure trait with *both* externally weighted GRSs as 2 independent variables. Predicted values from first-stage regressions were then used as independent variables in relation to outcome traits in second-stage models [32].

Given the large number of statistical tests performed and the correlated nature of outcome traits, the *P*-value threshold of 0.05 commonly used for guiding nominal statistical significance can be corrected for multiple testing using the Bonferroni method assuming 33 independent tests (the number of principle components explaining 95% of the variance in the cardiometabolic outcomes studied here in previous multi-cohort analyses [12]); the *P*-value threshold for statistical significance becomes *P <* 0.002. Here, *P*-values were interpreted as continuous indicators of the strength of evidence against the null hypothesis [33], with effect size and precision considered most informative. Analyses were conducted using Stata 14 (StataCorp).

### Replication analyses

As a replication analysis for 1-sample MR, 2-sample MR was additionally carried out using summary-level GWAS data available through the MR-Base platform (http://www.mrbase.org) [34]. Data for BMI were from the GIANT consortium [28], described above, representing 322,154 post-pubertal females and males. Data for cardiometabolic traits were from Kettunen et al. [35], based on trait concentrations derived from an NMR-based metabolomics platform (the same used for the above ALSPAC analyses) on 13,171 to 24,925 post-pubertal females and males from 14 cohort studies. Following additional SNP pruning for high linkage disequilibrium (LD) using *R*^2^ < 0.01 within a 10,000-kb distance to satisfy modelling criteria, inverse variance weighted (IVW) regression models were used to produce estimates of linear association (assuming no sample overlap and the same underlying population [20]). Coefficients were interpreted as standardised mean differences in outcome per year later age at menarche [21]. When an exposure-associated SNP was absent in the outcome GWAS, a proxy SNP in high LD (*R*^2^ ≥ 0.80) was used with the same criteria as above [34]. If no proxy SNP was available, then that SNP was excluded. Because of this and possible allele harmonisation issues, the number of instruments used for estimates varies and is reported. Where IVW coefficients showed strong evidence of effect, results from 2 sensitivity models were examined to account for directional pleiotropy. The first was MR-Egger regression [36], which tests for differences from a 0 intercept representing the average pleiotropic effect across all variants assuming that pleiotropic effects on the outcome are independent of their effects on the exposure; the slope provides an estimate of association magnitude allowing all such variants to be pleiotropic. The second sensitivity method was weighted median (WM) regression [37], which is interpreted similarly to IVW models but allows the weight of up to half of the contributing variants to be pleiotropic and is less influenced by outliers. To complement negative control 1-sample MR analyses for adiposity, we also examined the effect of later menarche on childhood obesity, defined as a ≥95th percentile BMI value (versus <50th percentile BMI value) using outcome data on 13,848 children (mean age across 14 cohorts = 2–10 y) from the Early Growth Genetics (EGG) consortium [38]. Analyses were performed using R-Studio 1.0.44 (R version 3.3.2) and publicly available code (TwoSampleMR; https://mrcieu.github.io/TwoSampleMR).

## Results

### Sample characteristics

Characteristics of ALSPAC females who were eligible for any analysis (i.e., who had data on age at menarche, covariates, at least 1 adiposity indicator, and at least 1 cardiometabolic trait) are shown in Table 1. Among 2,112 females, age at menarche ranged from 8 y to 16 y (mean = 12.37 y). Among 1,499 males, age at voice breaking ranged from 9.08 y to 16.67 y (mean = 13.21 y) (S1 Table). Females and males who had missing data on age at menarche/voice breaking but were otherwise eligible for inclusion in analyses had lower maternal education but showed no clear difference in adiposity and cardiometabolic traits (S2 Table).

**Table 1:** Characteristics of females by age at menarche group in ALSPAC at age 8 y and 18 y.

Characteristic	Age at menarche among females Sample mean (SD) = 12.37 y (1.12 y)			*P*-value	
	Earliest (8–11 y) *N =* 447	Intermediate (12 y) *N =* 722	Latest (13–16 y) *N =* 943	Intermediate versus earliest	Latest versus earliest
Mother has no further academic education—*N* (%)	249 (55.70)	384 (53.19)	493 (52.28)	0.40	0.23
***After puberty onset (age 18 y assessment)***					
Body mass index (kg/m^2^)—mean (SD)	24.39 (4.73)	23.15 (4.30)	22.04 (3.67)	6.76 × 10^−06^	4.53 × 10^−20^
Fat mass index (kg/m^2^)—mean (SD)	9.06 (3.76)	8.08 (3.32)	7.21 (3.04)	7.85 × 10^−06^	4.70 × 10^−19^
Systolic blood pressure (mm Hg)—mean (SD)	111.07 (8.19)	110.23 (7.84)	109.23 (7.58)	0.09	7.70 × 10^−05^
Diastolic blood pressure (mm Hg)—mean (SD)	66.30 (6.22)	64.97 (6.20)	64.34 (5.76)	4.42 × 10^−04^	3.19 × 10^−08^
Triglycerides (mmol/l)—mean (SD)	0.93 (0.32)	0.92 (0.31)	0.92 (0.31)	0.54	0.60
HDL cholesterol (mmol/l)—mean (SD)	1.47 (0.23)	1.49 (0.23)	1.50 (0.23)	0.22	0.05
LDL cholesterol (mmol/l)—mean (SD)	1.12 (0.35)	1.14 (0.35)	1.16 (0.36)	0.39	0.15
Total cholesterol (mmol/l)—mean (SD)	3.71 (0.65)	3.76 (0.65)	3.80 (0.68)	0.30	0.08
Glucose (mmol/l)—mean (SD)	4.06 (0.36)	4.04 (0.31)	4.05 (0.41)	0.42	0.68
Glycoprotein acetyls (mmol/l)—mean (SD)	1.26 (0.15)	1.24 (0.13)	1.24 (0.13)	0.11	0.06
***Before puberty onset (age 8 y assessment)***					
Body mass index (kg/m^2^)—mean (SD)	17.21 (2.32)	16.42 (2.13)	15.71 (1.72)	6.03 × 10^−08^	4.41 × 10^−29^
Fat mass index (kg/m^2^)—mean (SD)	5.91 (2.32)	5.01 (2.26)	4.26 (2.02)	2.68 × 10^−09^	1.47 × 10^−31^
Systolic blood pressure (mm Hg)—mean (SD)	100.88 (9.93)	99.02 (9.18)	97.73 (8.72)	0.003	1.25 × 10^−07^
Diastolic blood pressure (mm Hg)—mean (SD)	57.79 (6.67)	57.07 (6.56)	56.29 (6.34)	0.09	2.21 × 10^−04^
Triglycerides (mmol/l)—mean (SD)	1.09 (0.42)	1.09 (0.36)	1.06 (0.36)	0.98	0.27
HDL cholesterol (mmol/l)—mean (SD)	1.45 (0.21)	1.48 (0.19)	1.50 (0.20)	0.02	0.001
LDL cholesterol (mmol/l)—mean (SD)	1.23 (0.31)	1.29 (0.36)	1.27 (0.32)	0.03	0.09
Total cholesterol (mmol/l)—mean (SD)	3.94 (0.58)	4.04 (0.62)	4.02 (0.59)	0.04	0.06
Glucose (mmol/l)—mean (SD)	4.17 (0.51)	4.14 (0.47)	4.11 (0.51)	0.40	0.14
Glycoprotein acetyls (mmol/l)—mean (SD)	1.27 (0.14)	1.25 (0.13)	1.23 (0.14)	0.06	0.001

Sample sizes vary as participants described are those with data on age at menarche, covariates, at least 1 adiposity trait, and at least 1 cardiometabolic trait. Age at menarche was self-reported rounded down to the nearest whole year; the mean value was calculated from these values. ‘No further academic education’ defined as highest level of education attained being Certificate of Secondary Education, vocational, or O level (not A level or degree).

HDL, high-density lipoprotein; LDL, low-density lipoprotein.

### Observational associations of age at menarche with adiposity and cardiometabolic traits at age 18 y

**Fig 2:**
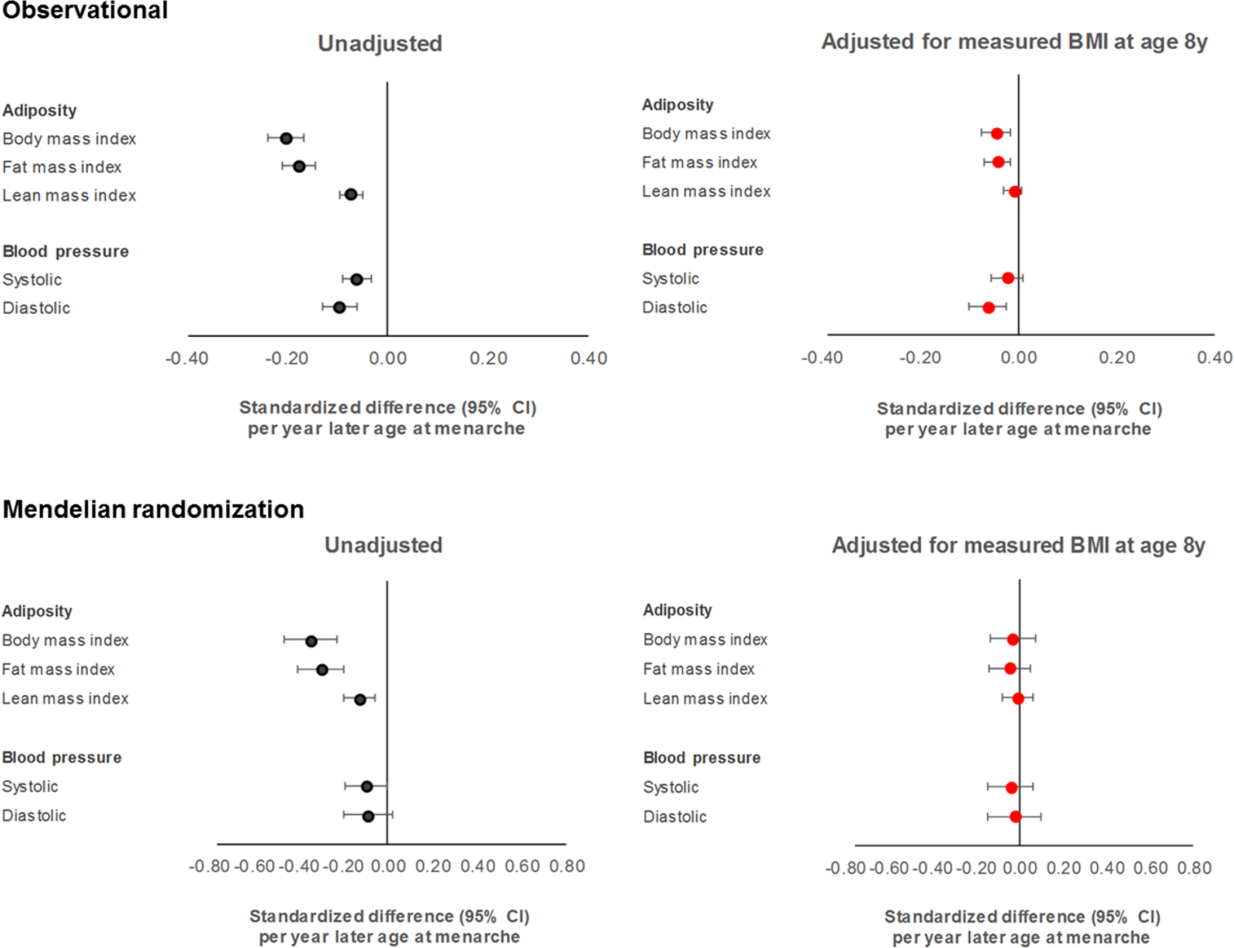
Associations of age at menarche with adiposity and blood pressure at age 18 y among females in ALSPAC.

Females in the latest versus earliest group of age at menarche had a lower BMI at 18 y (22.04 versus 24.39 kg/m^2^, *P* = 4.53 × 10^−20^), a difference already apparent at age 8 y (15.71 versus 17.21 kg/m^2^, *P* = 4.41 × 10^−29^; Table 1). In models adjusting for age and maternal education among 2,230 females (S3 Table; Fig 2), each year later age at menarche was associated with −0.81 kg/m^2^ of BMI at age 18 y (or −0.20 SD-units, 95% CI −0.24, −0.17; *P =* 5.91 × 10^−29^). Upon additional adjustment for BMI at age 8 y to account for differences in pre-pubertal BMI, this coefficient attenuated 4-fold from −0.81 kg/m^2^ to −0.20 kg/m^2^ (or −0.05 SDs, 95% CI −0.07, −0.02; *P =* 0.002). Patterns were similar for fat mass index but less apparent for lean mass index. Similar results were seen among males based on age at voice breaking: lower BMI was already observed at age 8 y with later voice breaking (S1 Table), and associations of later voice breaking with lower BMI at age 18 y attenuated upon adjustment for BMI at age 8 y (S4 Table). These associations were weaker among females and males combined than among the sexes separately (S5 Table).

**Fig 3:**
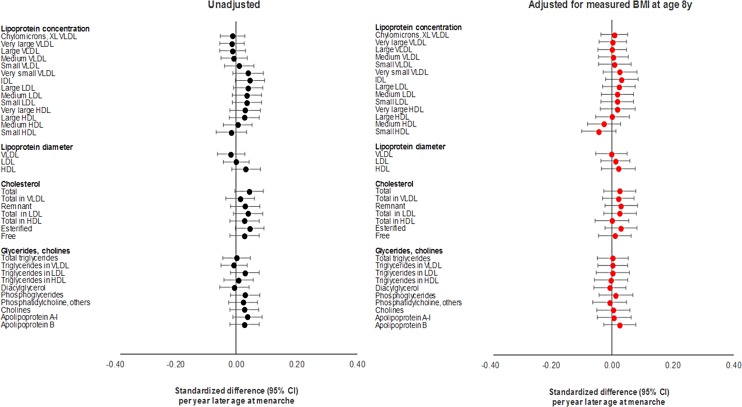
Observational associations of age at menarche with lipid cardiometabolic traits at age 18 y among females in ALSPAC. HDL, high-density lipoprotein; IDL, intermediate-density lipoprotein; LDL, low-density lipoprotein; VLDL, very low-density lipoprotein; XL VLDL, chylomicrons and extremely large very low-density lipoprotein.

**Fig 4:**
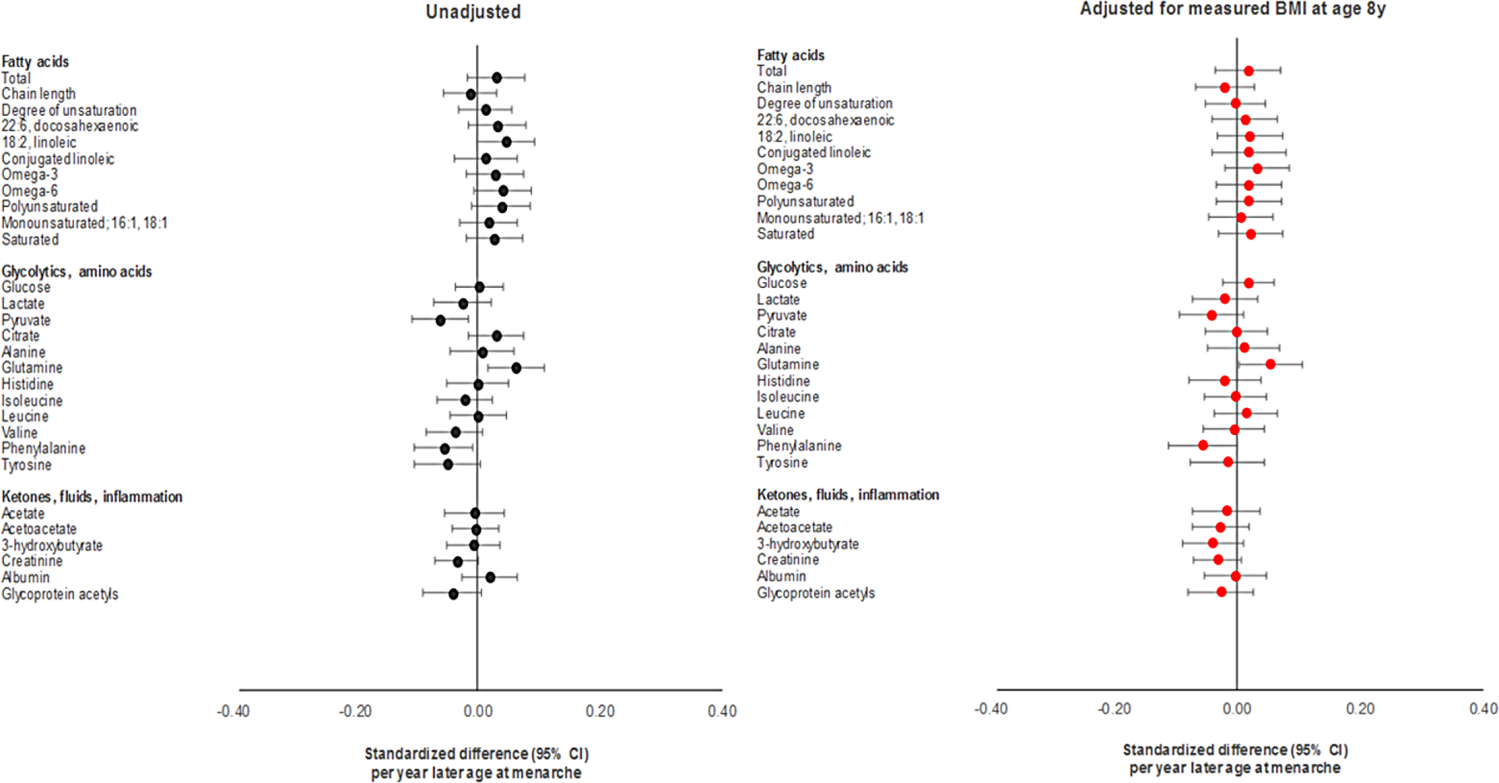
Observational associations of age at menarche with non-lipid cardiometabolic traits at age 18 y among females in ALSPAC.

Differences in summary cardiometabolic traits at age 18 y and at age 8 y among females in the latest versus earliest group of age at menarche were small, being largest for SBP and DBP (Table 1). In models adjusting for age and maternal education on up to 2,087 females (S3 Table; Figs 2–4), associations of later age at menarche with other cardiometabolic traits at age 18 y were generally small in magnitude, with moderate-to-high *P*-values. The direction of the effect with later age at menarche was inconsistent across very low-density lipoprotein (VLDL) traits, but was generally positive across intermediate-density lipoprotein (IDL), low-density lipoprotein (LDL), high-density lipoprotein (HDL), triglyceride, and fatty acid traits, and negative across SBP, DBP, and amino acid traits. The largest effect size was observed with DBP, at −0.63 mm Hg (or −0.10 SD-units, 95% CI −0.13, −0.06; *P =* 1.00 × 10^−07^). Attenuation was slight when adjusting for BMI at age 8 y, and when adjusting for specific outcome trait values at age 8 y (S1–S3 Figs) (e.g., the coefficient for DBP at age 18 y reduced from −0.63 mm Hg to −0.38 mm Hg when adjusting for BMI at age 8 y, and from −0.63 mm Hg to −0.50 mm Hg when adjusting for DBP at age 8 y). Among males, associations of age at voice breaking with cardiometabolic traits were generally smaller in magnitude; attenuations were slight upon adjustment for BMI at age 8 y and for specific outcome traits at age 8 y (S4 Table), likewise among females and males combined (S5 Table; S4–S9 Figs).

### One-sample MR of age at menarche for adiposity and cardiometabolic traits at age 18 y

Using the full GRS as an instrument among 2,053 females (S6 Table; Fig 2), each year later age at menarche was associated with −1.38 kg/m^2^ of BMI at age 18 y (or −0.34 SD-units, 95% CI −0.46, −0.23; *P =* 9.77 × 10^−09^). Effect size was larger for fat mass index (at −1.11 kg/m^2^) than for lean mass index (at −0.28 kg/m^2^). Upon adjustment for measured BMI at age 8 y, the coefficient for BMI at 18 y attenuated over 10-fold from −1.38 kg/m^2^ to −0.12 kg/m^2^ (or −0.03 SDs, 95% CI −0.13, 0.07; *P =* 0.55), with similar attenuations for fat and lean mass indices and upon adjustment for specific outcome trait values at age 8 y (S1–S3 Figs). Among females and males combined, attenuation of the coefficient for BMI was also evident, but with a greater effect on lean mass index than on fat mass index (S7 Table; S7 Fig). Similar results were seen when using the refined GRS among males (S8 Table). Results of a multivariate MR model using full GRSs among females (S9 Table) showed a similar unadjusted effect of later genetically predicted age at menarche on BMI at age 18 y (−0.28 SD-units, 95% CI −0.36, −0.19; *P =* 2.92 × 10^−10^) and a similar near-complete attenuation of this effect upon adjustment for genetically predicted BMI at age 8 y (to −0.001 SD-units, 95% CI −0.10, 0.10; *P =* 0.99). In this multivariate model, higher genetically predicted BMI at 8 y showed an expectedly greater effect on BMI at 18 y; this effect was minimally attenuated upon adjustment for genetically predicted age at menarche.

**Fig 5:**
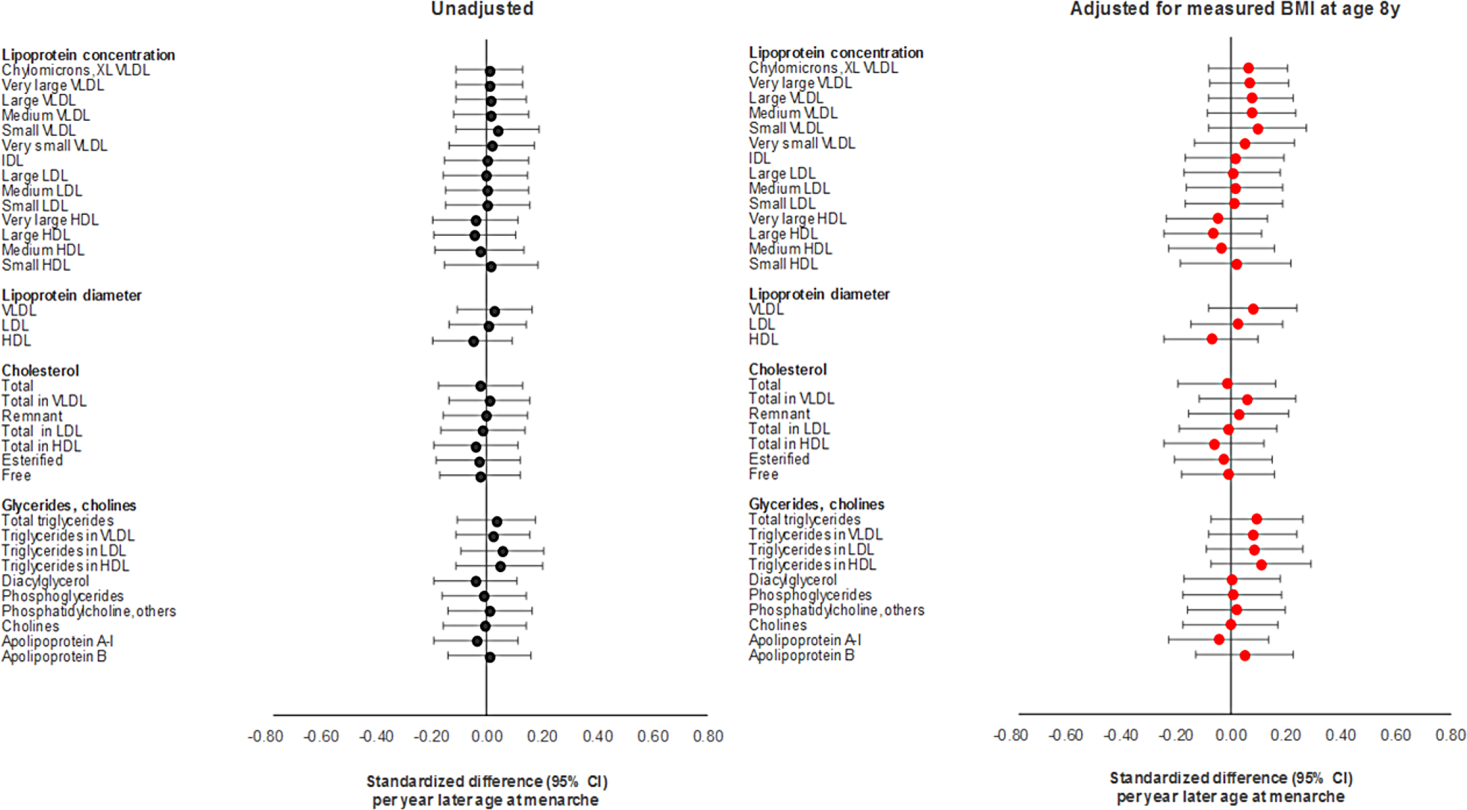
Mendelian randomisation of age at menarche for lipid cardiometabolic traits at age 18 y among females in ALSPAC. HDL, high-density lipoprotein; IDL, intermediate-density lipoprotein; LDL, low-density lipoprotein; VLDL, very low-density lipoprotein; XL VLDL, chylomicrons and extremely large very low-density lipoprotein.

**Fig 6:**
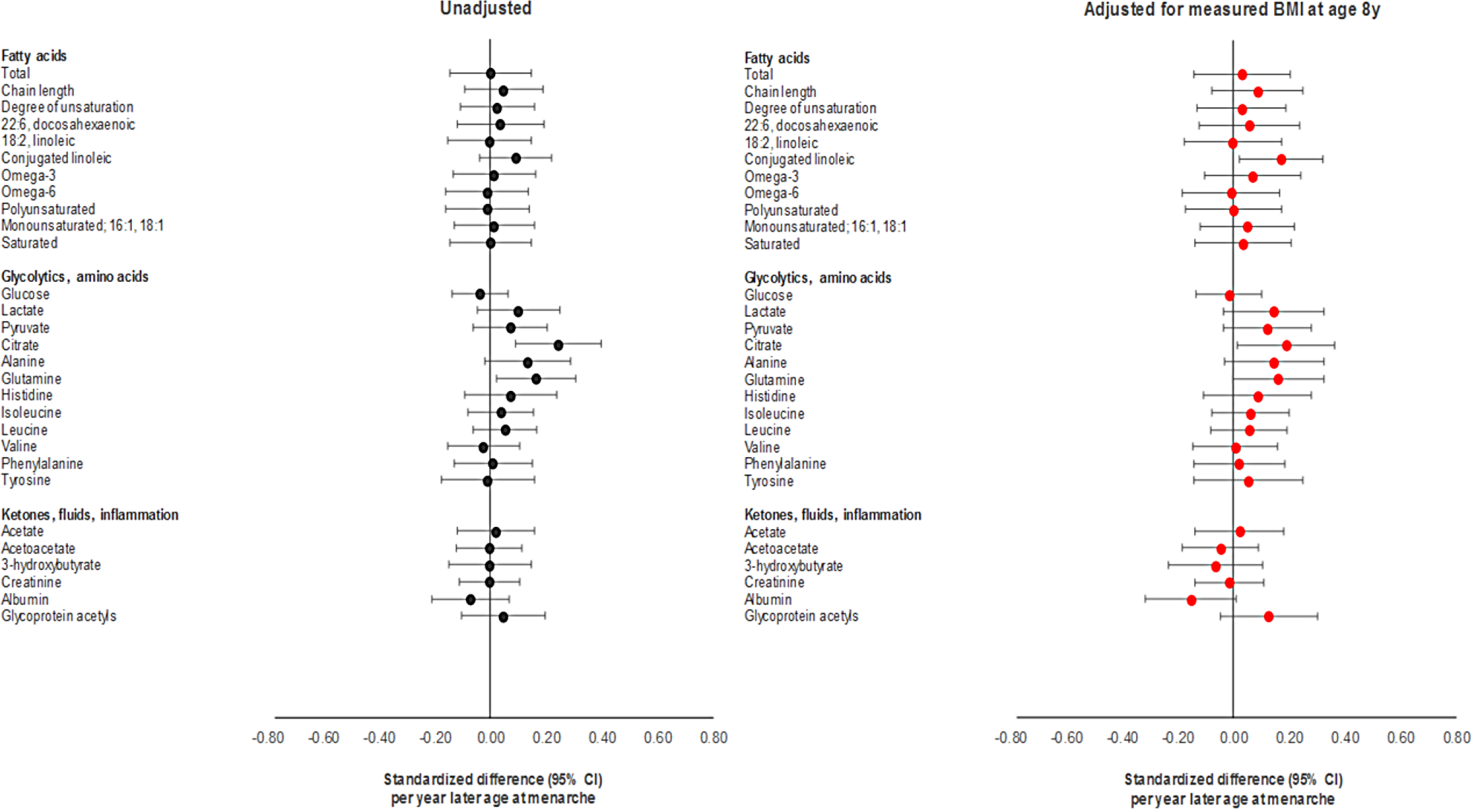
Mendelian randomisation of age at menarche for non-lipid cardiometabolic traits at age 18 y among females in ALSPAC.

Using the full GRS among females, effect sizes for later age at menarche for cardiometabolic traits were close to 0, with the direction of coefficients being inconsistent across VLDL traits; positive across IDL, LDL, HDL, triglyceride, and fatty acid traits; and negative across SBP, DBP, and amino acid traits (S6 Table; Figs 5 and 6). Substantial attenuation of coefficients for SBP and DBP was seen with adjustment for BMI at age 8 y; this attenuation was less pronounced when adjusting for genetically predicted BMI at age 8 y in a multivariate MR model (S9 Table). Attenuations showed no clear pattern across other cardiometabolic traits. Attenuations were similarly unclear with adjustment for specific outcome trait values at age 8 y (S1–S3 Figs). Among females and males combined (S7 Table; S4–S9 Figs), associations with cardiometabolic traits were more pronounced, with effect size being largest for SBP, at −3.88 mm Hg (or −0.40 SD-units, 95% CI −0.59, −0.22; *P =* 2.00 × 10^−05^), and for citrate, at 0.40 SD-units (95% CI 0.19, 0.61; *P =* 1.55 × 10^−40^). Attenuations, if any, were modest with adjustment for BMI at age 8 y but were more substantial with adjustment for specific outcome trait values at age 8 y. Using the refined GRS of 115 SNPs as an instrument among males (S8 Table), associations were generally weaker with adiposity and cardiometabolic traits, with greater inconsistency and imprecision than when using the full GRS among females and among both sexes combined, both before and after adjustment for BMI at age 8 y and for specific outcome trait values at age 8 y.

### Negative control 1-sample MR of age at menarche for adiposity and cardiometabolic traits at age 8 y

**Fig 7:**
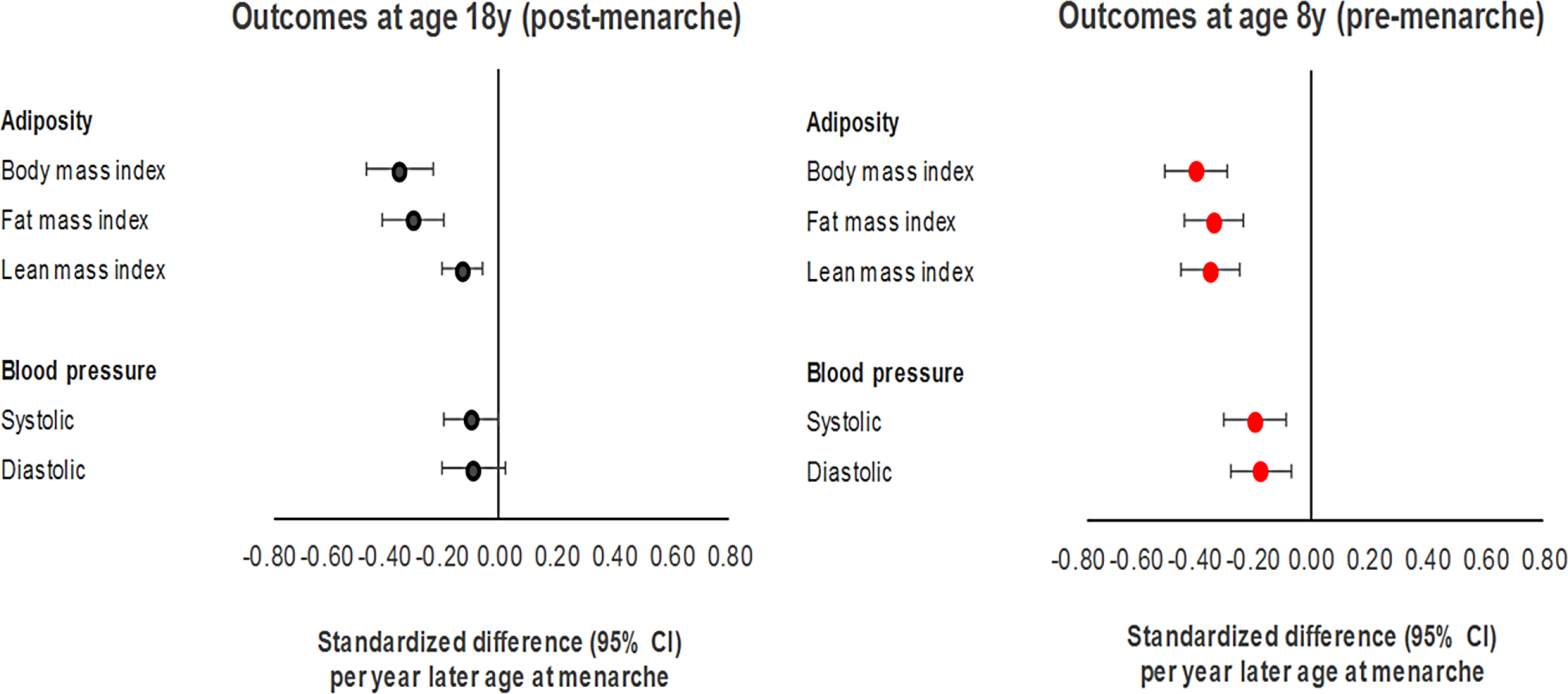
Negative control Mendelian randomisation of age at menarche for adiposity and blood pressure at age 8 y among females in ALSPAC.

Using the full GRS among females (Fig 7; S10 Table), each year later age at menarche was associated with −0.77 kg/m^2^ of BMI (or −0.39 SDs, 95% CI −0.50, −0.29; *P =* 6.28 × 10^−13^) measured at age 8 y. Effect sizes were similar for fat and lean mass indices, and were relatively high for SBP, at −1.73 mm Hg (or −0.19 SD-units, 95% CI −0.30, −0.08; *P =* 4.94 × 10^−04^), and DBP, at −1.13 mm Hg (or −0.17 SD-units, 95% CI −0.27, −0.07; *P =* 1.25 × 10^−03^), measured at age 8 y. Associations with other cardiometabolic traits at age 8 y were of a similar or higher magnitude to those seen at age 18 y (S10 Fig). Associations using the full GRS were of a larger magnitude among females and males combined than among females only (S11 Table; S11 and S12 Figs), likewise when using the refined GRS as an instrument among males (S12 Table).

### One-sample MR of pre-pubertal adiposity for age at puberty onset

Using the GRS of 95 SNPs for BMI as an instrument, higher BMI (per 1 kg/m^2^) at age 8 y was associated with an earlier age at menarche among 2,648 females, at −0.26 y (95% CI −0.37, −0.16; *P =* 1.16 × 10^−06^); an earlier age at voice breaking among 2,150 males, at −0.33 y (95% CI −0.57, −0.09; *P =* 0.01); and an earlier age at puberty onset among females and males combined, at −0.27 y (95% CI −0.39, −0.16; *P =* 3.43 × 10^−06^).

### Additional analyses

Results of complete case analyses for all above analyses are presented in S3–S12 Tables. Observational associations among a consistent sample of 664 females and 605 males were similar as among unrestricted samples; the largest effect sizes for later age at menarche were for BMI, fat mass index, lean mass index, SBP, and DBP at age 18 y, with similar attenuation patterns seen with adjustment for BMI and specific outcome trait values at age 8 y. Estimates from 1-sample MR models among a consistent sample of 629 females were similar as among unrestricted samples, as were those among a consistent sample of 1,193 females and males.

In additional analyses for power comparisons (S13 Table), higher BMI at age 8 y (per SD-unit higher) among males and females combined was expectedly associated with higher SBP (at 0.47 SD-units, 95% CI 0.33, 0.61; *P =* 3.75 × 10^−11^) and higher DBP (at 0.35 SD-units, 95% CI 0.20, 0.50; *P =* 5.93 × 10^−06^) at age 8 y. These associations were weaker but directionally concordant at age 18 y. Associations were also relatively strong with glycoprotein acetyls, HDL-cholesterol, triglycerides, and glucose. These associations were most consistent by sex for BMI with SBP, DBP, and glycoprotein acetyls.

Funnel plots of individual SNP-based effects of later age at menarche on adiposity and blood pressure at age 18 y (those outcomes with the strongest evidence of association in prior analyses) are shown in S13–S17 Figs. Effects were generally symmetrical around 0 for each outcome, with attenuations upon adjustment for measured BMI at age 8 y appearing general rather than SNP-specific. Some extreme outliers existed but carried the lowest 1/SE values, indicating the lowest precision.

### Replication analyses

Of 363 instrumentable SNPs for age at menarche identified in the source GWAS, up to 303 were available in summary-level outcome data after pruning. Based on IVW models using 234 SNPs and summary-level data for adult BMI, each year later age at menarche was associated with −0.81 kg/m^2^ of adult BMI (or −0.17 SD units, 95% CI −0.21, −0.12; *P =* 4.00 × 10^−15^; S14 Table). The Cochran *Q*-statistic for heterogeneity in this overall IVW estimate was 1,721.5 (*P =* 5.33 × 10^−225^), indicating a high degree of heterogeneity in individual SNP effects. The MR-Egger intercept did not differ from 0 however (*P =* 0.83), suggesting that individual SNP heterogeneity was largely balanced (no directional pleiotropy). Corresponding effect estimates from MR-Egger and WM sensitivity models were −0.18 SD units (95% CI −0.30, −0.06; *P =* 0.003) and −0.04 SD units (95% CI −0.06, −0.02; *P =* 0.002) of BMI, allowing for directional pleiotropy in up to 100% and 50% of genetic weights, respectively. Based on 202 SNPs, later menarche was associated with lower odds of childhood obesity in the EGG consortium data (IVW-based odds ratio = 0.52 per year later, 95% CI 0.48, 0.57; *P =* 6.64 × 10^−15^). Individual SNP heterogeneity was apparent (IVW *Q* = 384.7; *P =* 1.19 × 10^−13^), with 137 SNPs exerting a negative effect and 67 exerting a positive effect. This heterogeneity did not appear unbalanced (MR-Egger intercept *P =* 0.56).

Effect sizes for associations of age at puberty onset with adult cardiometabolic traits were generally low in magnitude (absolute values between 0.01 to 0.04 SD units). The direction of effects related to later age at menarche was generally negative across VLDL, IDL, and LDL traits, positive across HDL traits, and negative across fatty acids and amino acids. Using 82 SNPs as instruments for both age at menarche and voice breaking (S15 Table) resulted in a similar magnitude of association of later puberty onset with lower BMI, at −0.16 SD units (95% CI −0.24, −0.08; *P =* 4.33 × 10^−05^). The MR-Egger intercept did not differ from 0 (*P =* 0.93), and MR-Egger and WM models estimated the association as being −0.17 SD units (95% CI −0.37, 0.03; *P =* 0.10) and −0.04 SD units (95% CI −0.08, −0.00; *P =* 0.06), respectively. Association magnitudes with cardiometabolic traits were similar but generally more modest using 104 SNPs for both age at menarche and voice breaking (S15 Table).

## Discussion

We sought in this study to determine whether puberty timing is likely to have a distinct influence on adiposity and cardiometabolic traits in adulthood. To do this, we used both observational and genetically informed methods, which together allow more reliable inference on causality than previously possible. Our results suggest that apparent effects of puberty timing on adiposity and cardiometabolic traits in adulthood are largely confounded by pre-pubertal adiposity and are not likely driven by puberty timing itself. Strong evidence was also found for an effect of higher pre-pubertal adiposity on earlier age at puberty onset. These findings together point to childhood adiposity as a primary intervention target.

Our first set of analyses used observational data on age at menarche among females and age at voice breaking among males in relation to adiposity measured at age 18 y. Results suggested that later menarche among females was associated with lower subsequent adiposity (assessed using both BMI and objective fat mass), but that these associations were largely statistically accounted for by differences in adiposity at age 8 y, and thereby reflect known tendencies within individuals for adiposity levels in childhood to track forward to later life stages [9,10]. We also examined over 200 detailed cardiometabolic traits in adulthood using the same approach, generally finding weaker evidence for associations and some attenuation of already small effect sizes when accounting for pre-pubertal trait values, again suggesting that metabolic disturbances likely predate puberty onset. Associations were similar albeit weaker among males, possibly due to greater measurement error in age at voice breaking.

Our second set of analyses used genetic variants as instruments for puberty timing within a 1-sample MR framework to minimise issues of confounding. However, since genetic variants for age at menarche are expected to be pleiotropic with BMI [21], these MR estimates were further adjusted for BMI at age 8 y, which should help further address residual confounding since this measure preceded the onset of puberty (a time-sensitive exposure). Unadjusted results among females suggested a causal effect of later menarche on lower adult adiposity as reported previously [21,39], but this effect was almost completely attenuated with adjustment for BMI measured at age 8 y, supporting confounding by pre-pubertal adiposity. The time-sensitive nature of puberty timing as an exposure also allowed us to conduct negative control MR analyses, whereby pre-pubertal traits were examined as outcomes to confirm the non-existence of effects that should not exist if age at puberty onset were a distinct exposure with valid instruments. Any effects of puberty timing on traits measured before puberty onset must be generated not by puberty timing itself (because it is temporally implausible) but by confounding factors. Results of these negative control analyses suggested apparent effects of later menarche on lowering pre-menarche adiposity and blood pressure that were at least as strong as when outcomes were measured post-menarche—likewise for the effect of later menarche on lower odds of childhood obesity in the 2-sample MR setting—further indicating confounding of puberty timing effects, likely by adiposity. Beyond reinforcing the need to adjust MR estimates of puberty timing for a pre-pubertal measure of BMI, known overlap in genetic variants also challenges the notion of puberty timing and adiposity being distinct phenomena.

Conceptual overlap certainly exists between pre-pubertal growth and pre-pubertal adiposity (body size), with ‘age at puberty onset’ likely intended as a marker of the rate of pre-pubertal growth. Overlap was also supported statistically by way of a high degree of heterogeneity in individual puberty-variant effects on adult BMI in the 2-sample MR setting, suggesting that multiple pathways of effect exist, although this heterogeneity appeared balanced. In MR terms, this could therefore be a case of vertical rather than horizontal pleiotropy if genetic variants influence puberty timing by first influencing pre-pubertal adiposity, which in turn brings puberty forward. Primary effects of puberty-inducing variants could also operate via pathways unrelated to pre-pubertal adiposity. Effects of puberty timing on later adiposity may therefore be genuine but part of a wider chain of events in which pre-pubertal adiposity influences puberty timing, which then further influences adiposity. Still, a literal and direct interpretation of age at puberty onset within a confounding framework can serve as a means of interrogating these conceptual overlaps and identifying a unifying root cause that can then be targeted for intervention. Together with evidence of an effect of higher pre-pubertal BMI on earlier puberty onset, our results support puberty timing as a marker, not a driver, of adiposity and cardiometabolic trait levels, positioning childhood adiposity as a primary intervention target. Interventions are important given results of a recent Danish study suggesting that moderately raised adiposity in childhood (at age 7 y, before puberty) does not confer increased risk for type 2 diabetes in older age if it does not persist into post-pubertal life stages [40]. Moderately raised adiposity during puberty (age 13 y) conferred increased diabetes risk in older age whether it persisted or not, as did very high childhood adiposity. Current MR results in ALSPAC support strong effects of higher childhood adiposity on adulthood adiposity, indicating that exposure tends to persist. Lifelong high exposure likely confers the greatest long-term diabetes risk [40].

Beyond blood pressure, generally weak evidence was found for causal associations of genetically instrumented later age at menarche among females with lipid- and glycaemic-related traits derived from targeted metabolomics. Coefficients were practically null across VLDL, IDL, LDL, HDL, and fatty acid traits, but slightly positive across glycolysis and amino acid traits. All were accompanied by relatively weak levels of statistical significance, particularly when considering a more stringent threshold corrected for multiple testing. Attenuations of already weak associations after adjustment for pre-pubertal BMI were minimal, and changes were instead often away from the null, likewise when adjusting for pre-pubertal values of specific trait outcomes. Association patterns across non-blood-pressure cardiometabolic traits also differed when considering females and males combined, with coefficients generally being positive across lipid-related and fatty acid traits and negative across amino acid traits; these coefficients were largely uninfluenced by adjustment for pre-pubertal BMI. Despite larger sample size, the reliability of the results in the sex-combined analyses is questionable given markedly reduced instrument strength (the variance explained in exposure traits was approximately 11% among females versus approximately 2% among females and males combined). Overall, the inconsistent association patterns across cardiometabolic traits are more indicative of chance than robust causality.

Two-sample MR analyses were also conducted using publicly available GWAS data as a means of external replication. Results supported effects of later age at puberty onset on lowering adult BMI, and suggested associations with a range of adult cardiometabolic traits. Directions of effect were often opposite to those seen with 1-sample MR estimates however: negative across lipid traits except HDL, and negative across fatty acids, amino acids, and inflammatory glycoproteins. These patterns closely reflect what would be expected in response to lower adiposity [12] and likely indicate pleiotropy of puberty timing instruments with BMI. Two-sample models have the advantage of larger sample sizes and newly developed sensitivity methods to account for directional pleiotropy, but only 1-sample models could account for adiposity and cardiometabolic trait values as specifically measured before puberty onset. Results of one recent MR study using UK Biobank data also support an effect of later puberty on lowering adult BMI; this effect attenuated 2-fold when adjusting puberty-variant effects for their effect on childhood BMI in the EGG consortium data using multivariate MR [39]. Genetic variants used were based on an older GWAS and were fewer in number (122 versus up to 351 here), and a *P-*value threshold for association with childhood BMI in EGG was relied upon for subsequent analyses; this is likely inadequate given strong residual associations of manually BMI-pruned puberty GRSs with BMI [21]. Thus, it is likely that despite lower estimate precision in our study due to smaller sample size, the attenuation patterns seen for adiposity in adjusted 1-sample MR models are most reflective of the influence of puberty timing itself.

### Strengths and limitations

Key strengths of this study include its use of both prospective observational and genetically informed analyses to address causality more comprehensively than previously possible. Repeat data were used for BMI, objective fat mass, and over 200 detailed cardiometabolic traits measured before and after puberty onset, offering rare and extensive insight into temporality. Replications of 1-sample MR analyses were carried out using large-scale genetic data within a 2-sample MR setting on up to 322,154 adults. The strength of genetic instruments for puberty timing was high among females in ALSPAC, explaining nearly 11% of exposure variance.

Limitations of this study include modest completion rates of puberty questionnaires in ALSPAC (e.g., 58.4% among females), as is typical for sensitive topics; results here and elsewhere are thus prone to selection bias. This proportion of responders is higher among those with adiposity and cardiometabolic outcome data, at 83.2%, and comparisons suggested little influence of selection on adiposity and cardiometabolic traits. Measurement error is likely among those who did participate [41], especially among males [42] given the sporadic nature of voice breaking. Estimates may therefore be less precise among males than among females. This imprecision may have also made MR analyses of males prone to weak instrument bias, which would bias association estimates towards the observational estimate in a 1-sample setting and towards the null in a 2-sample setting. That evidence for association was generally weak in both settings, as well as observationally, suggests that measurement imprecision is a core concern. The non-blood-pressure cardiometabolic traits examined were heavily lipid-focused [30], and relations of puberty timing with hormone-related traits such as insulin and insulin-like growth factors were not examined. As noted, health behaviour covariates were not considered in observational analyses due to individual differences in exposure timing. Behaviours such as physical activity are also subject to change over the course of puberty [43], and recorded levels in childhood may not represent levels in adolescence. The prevalence and duration of cigarette smoking and alcohol consumption are also expected to be low at puberty onset. In any case, MR analyses negated the need for such covariates as risk alleles are expected to be distributed randomly [44]. Not all cardiometabolic traits were available for 2-sample MR analyses, but available traits covered most lipid subclass, fatty acid, amino acid, and inflammatory domains.

Participants not of a white-European ancestry were excluded to reduce confounding by population stratification and because genetic variants for puberty timing were identified in predominantly white-European samples. Future GWAS efforts could focus on other ethnic ancestries to allow causal inference across diverse populations. The MR methods used assume that modelled associations are linear. Some previous observational studies have suggested that age at puberty onset may relate non-linearly to cardiometabolic and other diseases, with increased risk apparently conferred by both early and late onset [45,46]. But the extent to which these non-linear relations reflect genuine biology or confounding is unclear. Several apparently non-linear associations, such as those observed between BMI and mortality [47] and between alcohol consumption and cardiovascular disease [48], are not supported by instrumental variable methods [49–52] and may therefore reflect confounding by subclinical disease or social factors. Similar distortions of observed associations are possible with age at puberty onset. Finally, despite extensive participant phenotyping, sample sizes for ALSPAC analyses were modest. Substantial variance in age at menarche explained by its genetic instrument afforded reasonable precision, however, and expectedly positive estimates of higher BMI with higher SBP, DBP, and several other summary cardiometabolic traits at age 8 y and 18 y provided further reassurance of adequate sample power.

Puberty spans several years for both sexes; the exposure of interest here was the age at which it begins as this is what has been widely linked with health outcomes, cardiometabolic and otherwise [46]. Data permitting, future studies among females could examine the full duration of time between menarche and end of menopause to better capture lifetime exposure to sex-hormone-related growth factors. Future work may also utilise observational and genetic data on more objective and precise measures of puberty timing such as age at peak height velocity [53]. Use of a 2-sample MR design allowed large sample sizes and for inferences to be drawn from multiple cohorts. Relevant to this, however, is the issue of participant overlap between exposure and outcome GWAS datasets, which may bias effect estimates towards the confounding-prone observed association [54,55]. Potentially 25 of the 42 cohorts forming the GWAS for age at menarche [21] were also part of the GWAS for BMI [28], while 3 of the age at menarche cohorts may have also been part of the GWAS for metabolic traits [35]. A further 9 of the 114 cohorts for BMI may have also been part of the metabolic trait GWAS (this representing over half of the cohorts in the latter). Removing these participants from existing summary-level data is infeasible and would carry trade-offs including reduced power. This overlap issue will become increasingly important with the growing scale of multi-cohort collaborations and large-scale biobanks. Methodological advancements are needed to address this.

### Conclusions

Results of this study suggest that age at puberty onset has only a small influence on adiposity and cardiometabolic traits in adulthood because effects are largely confounded by pre-pubertal adiposity. Strong evidence was also found for an effect of higher pre-pubertal adiposity on earlier puberty onset, together pointing to childhood adiposity as a primary intervention target.

## Supporting information

S1 STROBE Checklist STROBE statement. (PDF)

S1 Fig Associations of age at menarche with adiposity and blood pressure at age 18 y among females in ALSPAC, with adjustment for specific outcome trait values at age 8 y. (TIF)

S2 Fig Associations of age at menarche with lipid cardiometabolic traits at age 18 y among females in ALSPAC, with adjustment for specific outcome trait values at age 8 y. (TIF)

S3 Fig Associations of age at menarche with non-lipid cardiometabolic traits at age 18 y among females in ALSPAC, with adjustment for specific outcome trait values at age 8 y. (TIF)

S4 Fig Associations of age at puberty onset with adiposity and blood pressure at age 18 y among females and males in ALSPAC. (TIF)

S5 Fig Associations of age at puberty onset with lipid cardiometabolic traits at age 18 y among females and males in ALSPAC. (TIF)

S6 Fig Associations of age at puberty onset with non-lipid cardiometabolic traits at age 18 y among females and males in ALSPAC. (TIF)

S7 Fig Associations of age at puberty onset with adiposity and blood pressure at age 18 y among females and males in ALSPAC, adjusted for specific outcome trait values at age 8 y. (TIF)

S8 Fig Associations of age at puberty onset with lipid cardiometabolic traits at age 18 y among females and males in ALSPAC, adjusted for specific outcome trait values at age 8 y. (TIF)

S9 Fig Associations of age at puberty onset with non-lipid cardiometabolic traits at age 18 y among females and males in ALSPAC, adjusted for specific outcome trait values at age 8 y. (TIF)

S10 Fig Negative control MR of age at menarche for cardiometabolic traits at age 8 y among females in ALSPAC. (TIF)

S11 Fig Negative control MR of age at puberty onset for adiposity and blood pressure at age 8 y among females and males in ALSPAC. (TIF)

S12 Fig Negative control MR of age at puberty onset for cardiometabolic traits at age 8 y among females and males in ALSPAC. (TIF)

S13 Fig Individual SNP effects of later age at menarche on BMI at age 18 y among females in ALSPAC, unadjusted (*N =* 2,054) and adjusted (*N =* 1,839) for measured BMI at age 8 y. (TIF)

S14 Fig Individual SNP effects of later age at menarche on fat mass index at age 18 y among females in ALSPAC, unadjusted (*N =* 1,977) and adjusted (*N =* 1,780) for measured BMI at age 8 y. (TIF)

S15 Fig Individual SNP effects of later age at menarche on lean mass index at age 18 y among females in ALSPAC, unadjusted (*N =* 1,977) and adjusted (*N =* 1,780) for measured BMI at age 8 y. (TIF)

S16 Fig Individual SNP effects of later age at menarche on SBP at age 18 y among females in ALSPAC, unadjusted (*N =* 1,934) and adjusted (*N =* 1,740) for measured BMI at age 8 y. (TIF)

S17 Fig Individual SNP effects of later age at menarche on DBP at age 18 y among females in ALSPAC, unadjusted (*N =* 1,934) and adjusted (*N =* 1,740) for measured BMI at age 8 y. (TIF)

S1 Protocol Prespecified study protocol. (PDF)

S1 Table Characteristics of males by age at voice breaking in ALSPAC. (PDF)

S2 Table Characteristics of participants by missing data status on age at menarche/voice breaking in ALSPAC. (PDF)

S3 Table Observational associations of age at menarche (per year later) with adiposity and cardiometabolic traits at age 18 y among females in ALSPAC. (PDF)

S4 Table Observational associations of age at voice breaking (per year later) with adiposity and cardiometabolic traits at age 18 y among males in ALSPAC. (PDF)

S5 Table Observational associations of age at puberty onset (per year later) with adiposity and cardiometabolic traits at age 18 y among females and males in ALSPAC. (PDF)

S6 Table One-sample MR estimates of associations of age at menarche (per year later) with adiposity and cardiometabolic traits at age 18 y among females in ALSPAC, using a full GRS of 351 SNPs for age at menarche. (PDF)

S7 Table One-sample MR estimates of associations of puberty timing (per year later) with adiposity and cardiometabolic traits at age 18 y among males and females in ALSPAC, using a full GRS of 351 SNPs for age at menarche. (PDF)

S8 Table One-sample MR estimates of associations of age at voice breaking (per year later) with adiposity and cardiometabolic traits at age 18 y among males in ALSPAC, using a refined GRS of 115 SNPs for age at menarche/voice breaking. (PDF)

S9 Table One-sample multivariate Mendelian randomisation estimates of genetically predicted age at menarche and childhood BMI with adulthood adiposity and blood pressure among females in ALSPAC, for comparisons with Fig 2. (PDF)

S10 Table Negative control 1-sample MR estimates of associations of age at menarche (per year later) with adiposity and cardiometabolic traits at age 8 y among females in ALSPAC, using a full GRS of 351 SNPs for age at menarche. (PDF)

S11 Table Negative control 1-sample MR estimates of associations of puberty timing (per year later) with adiposity and cardiometabolic traits at age 8 y among males and females in ALSPAC, using a full GRS of 351 SNPs for age at menarche. (PDF)

S12 Table Negative control 1-sample MR estimates of associations of age at voice breaking (per year later) with adiposity and cardiometabolic traits at age 8 y among males in ALSPAC, using a refined GRS of 115 SNPs for age at menarche/voice breaking. (PDF)

S13 Table One-sample MR estimates of associations of BMI with blood pressure and summary cardiometabolic traits at age 8 y and 18 y in ALSPAC, for sample power comparisons. (PDF)

S14 Table Two-sample MR estimates of associations of puberty timing (per year later) with post-pubertal adiposity and cardiometabolic traits among males and females in GWAS data, using a full set of up to 303 SNPs for age at menarche. (PDF)

S15 Table Two-sample MR estimates of associations of puberty timing (per year later) with post-pubertal adiposity and cardiometabolic traits among males and females in GWAS data, using a refined set of up to 104 SNPs for age at menarche. (PDF)

## Abbreviations

2SLS: 2-stage least squares
ALSPAC: Avon Longitudinal Study of Parents and Children
BMI: body mass index
DBP: diastolic blood pressure
EGG: Early Growth Genetics
GIANT: Genetic Investigation of Anthropometric Traits
GRS: genetic risk score
GWAS: genome-wide association study
HDL: high-density lipoprotein
IDL: intermediate-density lipoprotein
IVW: inverse variance weighted
LDL: low-density lipoprotein
MR: Mendelian randomisation
NMR: nuclear magnetic resonance
SBP: systolic blood pressure
SNP: single nucleotide polymorphism
VLDL: very low-density lipoprotein
WM: weighted median

## References

[1] Perry JR, Murray A, Day FR, Ong KK. Molecular insights into the aetiology of female reproductive ageing. Nat Rev Endocrinol. 2015;11(12):725–34. 10.1038/nrendo.2015.167 26460341 PMC6309261

[2] Patton GC, Viner R. Pubertal transitions in health. Lancet. 2007;369(9567):1130–9. 10.1016/S0140-6736(07)60366-3 17398312

[3] Sisk CL, Foster DL. The neural basis of puberty and adolescence. Nat Neurosci. 2004;7(10):1040–7. 10.1038/nn1326 15452575

[4] Widén E, Silventoinen K, Sovio U, Ripatti S, Cousminer DL, Hartikainen A-L, et al Pubertal timing and growth influences cardiometabolic risk factors in adult males and females. Diabetes Care. 2012;35(4):850–6. 10.2337/dc11-1365 22338106 PMC3308310

[5] Elks CE, Ong KK, Scott RA, Van Der Schouw YT, Brand JS, Wark PA, et al Age at menarche and type 2 diabetes risk. Diabetes Care. 2013;36(11):3526–34. 10.2337/dc13-0446 24159179 PMC3816901

[6] Canoy D, Beral V, Balkwill A, Wright FL, Kroll ME, Reeves GK, et al Age at menarche and risks of coronary heart and other vascular diseases in a large UK cohort. Circulation. 2015;131(3):237–44. 10.1161/CIRCULATIONAHA.114.010070 25512444

[7] Charalampopoulos D, McLoughlin A, Elks CE, Ong KK. Age at menarche and risks of all-cause and cardiovascular death: a systematic review and meta-analysis. Am J Epidemiol. 2014;180(1):29–40. 10.1093/aje/kwu113 24920784 PMC4070937

[8] Ahmed ML, Ong KK, Dunger DB. Childhood obesity and the timing of puberty. Trends Endocrinol Metab. 2009;20(5):237–42. 10.1016/j.tem.2009.02.004 19541497

[9] Power C, Lake JK, Cole TJ. Body mass index and height from childhood to adulthood in the 1958 British born cohort. Am J Clin Nutr. 1997;66(5):1094–101. 10.1093/ajcn/66.5.1094 9356525

[10] Johnson W, Li L, Kuh D, Hardy R. How has the age-related process of overweight or obesity development changed over time? Co-ordinated analyses of individual participant data from five United Kingdom birth cohorts. PLoS Med. 2015;12(5):e1001828 10.1371/journal.pmed.1001828 25993005 PMC4437909

[11] Holmes MV, Lange LA, Palmer T, Lanktree MB, North KE, Almoguera B, et al Causal effects of body mass index on cardiometabolic traits and events: a Mendelian randomization analysis. Am J Hum Genet. 2014;94(2):198–208. 10.1016/j.ajhg.2013.12.014 24462370 PMC3928659

[12] Würtz P, Wang Q, Kangas AJ, Richmond RC, Skarp J, Tiainen M, et al Metabolic signatures of adiposity in young adults: Mendelian randomization analysis and effects of weight change. PLoS Med. 2014;11(12):e1001765 10.1371/journal.pmed.1001765 25490400 PMC4260795

[13] Wade KH, Chiesa ST, Hughes AD, Chaturvedi N, Charakida M, Rapala A, et al Assessing the causal role of body mass index on cardiovascular health in young adults: Mendelian randomization and recall-by-genotype analyses. bioRxiv. 2017 4 10 10.1101/112912 PMC6250296 30524135

[14] Prentice P, Viner R. Pubertal timing and adult obesity and cardiometabolic risk in women and men: a systematic review and meta-analysis. Int J Obes (Lond). 2013;37(8):1036–43. 23164700 10.1038/ijo.2012.177

[15] Dreyfus J, Jacobs DR, Mueller N, Schreiner PJ, Moran A, Carnethon MR, et al Age at menarche and cardiometabolic risk in adulthood: the coronary artery risk development in young adults study. J Pediatr. 2015;167(2):344–52.e1. 10.1016/j.jpeds.2015.04.032 25962931 PMC4516565

[16] Kivimäki M, Lawlor DA, Davey Smith G, Elovainio M, Jokela M, Keltikangas-Järvinen L, et al Association of age at menarche with cardiovascular risk factors, vascular structure, and function in adulthood: the Cardiovascular Risk in Young Finns study. Am J Clin Nutr. 2008;87(6):1876–82. 10.1093/ajcn/87.6.1876 18541580

[17] Bubach S, Menezes AMB, Barros FC, Wehrmeister FC, Gonçalves H, Assunção MCF, et al Impact of the age at menarche on body composition in adulthood: results from two birth cohort studies. BMC Public Health. 2016;16(1):1007 10.1186/s12889-016-3649-x 27660104 PMC5034580

[18] Davey Smith G, Ebrahim S. ‘Mendelian randomization’: can genetic epidemiology contribute to understanding environmental determinants of disease? Int J Epidemiol. 2003;32(1):1–22. 12689998 10.1093/ije/dyg070

[19] Evans DM, Davey Smith G. Mendelian randomization: new applications in the coming age of hypothesis-free causality. Annu Rev Genomics Hum Genet. 2015;16:327–50. 10.1146/annurev-genom-090314-050016 25939054

[20] Burgess S, Scott RA, Timpson NJ, Davey Smith G, Thompson SG, Consortium E-I. Using published data in Mendelian randomization: a blueprint for efficient identification of causal risk factors. Eur J Epidemiol. 2015;30(7):543–52. 10.1007/s10654-015-0011-z 25773750 PMC4516908

[21] Day FR, Thompson DJ, Helgason H, Chasman DI, Finucane H, Sulem P, et al Genomic analyses identify hundreds of variants associated with age at menarche and support a role for puberty timing in cancer risk. Nat Genet. 2017;49(6):834–41. 10.1038/ng.3841 28436984 PMC5841952

[22] Day FR, Bulik-Sullivan B, Hinds DA, Finucane HK, Murabito JM, Tung JY, et al Shared genetic aetiology of puberty timing between sexes and with health-related outcomes. Nat Commun. 2015;6:8842 10.1038/ncomms9842 26548314 PMC4667609

[23] Burgess S, Thompson SG. Multivariable Mendelian randomization: the use of pleiotropic genetic variants to estimate causal effects. Am J Epidemiol. 2015;181(4):251–60. 10.1093/aje/kwu283 25632051 PMC4325677

[24] Holmes MV, Ala-Korpela M, Davey Smith G. Mendelian randomization in cardiometabolic disease: challenges in evaluating causality. Nat Rev Cardiol. 2017;14:577–90. 10.1038/nrcardio.2017.78 28569269 PMC5600813

[25] Boyd A, Golding J, Macleod J, Lawlor DA, Fraser A, Henderson J, et al Cohort profile: the ‘children of the 90s’—the index offspring of the Avon Longitudinal Study of Parents and Children. Int J Epidemiol. 2012;42(1):111–27. 10.1093/ije/dys064 22507743 PMC3600618

[26] Davey Smith G, Hemani G. Mendelian randomization: genetic anchors for causal inference in epidemiological studies. Hum Mol Gen. 2014;23(R1):R89–98. 10.1093/hmg/ddu328 25064373 PMC4170722

[27] Haycock PC, Burgess S, Wade KH, Bowden J, Relton C, Davey Smith G. Best (but oft-forgotten) practices: the design, analysis, and interpretation of Mendelian randomization studies. Am J Clin Nutr. 2016;103(4):965–78. 10.3945/ajcn.115.118216 26961927 PMC4807699

[28] Locke AE, Kahali B, Berndt SI, Justice AE, Pers TH, Day FR, et al Genetic studies of body mass index yield new insights for obesity biology. Nature. 2015;518(7538):197–206. 10.1038/nature14177 25673413 PMC4382211

[29] Sidhu D, Naugler C. Fasting time and lipid levels in a community-based population: a cross-sectional study. Arch Intern Med. 2012;172(22):1707–10. 10.1001/archinternmed.2012.3708 23147400

[30] Soininen P, Kangas AJ, Würtz P, Suna T, Ala-Korpela M. Quantitative serum nuclear magnetic resonance metabolomics in cardiovascular epidemiology and genetics. Circ Cardiovasc Genet. 2015;8(1):192–206. 10.1161/CIRCGENETICS.114.000216 25691689

[31] Ferreira DLS, Williams DM, Kangas AJ, Soininen P, Ala-Korpela M, Davey Smith G, et al Association of pre-pregnancy body mass index with offspring metabolic profile: analyses of 3 European prospective birth cohorts. PLoS Med. 2017;14(8):e1002376 10.1371/journal.pmed.1002376 28829768 PMC5568725

[32] Sanderson E, Smith GD, Windmeijer F, Bowden J. An examination of multivariable Mendelian randomization in the single sample and two-sample summary data settings. bioRxiv. 2018 4 27 10.1101/306209 PMC7394940 32529219

[33] Sterne JA, Davey Smith G. Sifting the evidence—what’s wrong with significance tests? BMJ. 2001;322(7280):226–31. 11159626 10.1136/bmj.322.7280.226 PMC1119478

[34] Hemani G, Zheng J, Elsworth B, Wade KH, Haberland V, Baird D, et al The MR-Base platform supports systematic causal inference across the human phenome. eLife. 2018;7:e34408 10.7554/eLife.34408 29846171 PMC5976434

[35] Kettunen J, Demirkan A, Würtz P, Draisma HH, Haller T, Rawal R, et al Genome-wide study for circulating metabolites identifies 62 loci and reveals novel systemic effects of LPA. Nat Commun. 2016;7:11122 10.1038/ncomms11122 27005778 PMC4814583

[36] Bowden J, Davey Smith G, Burgess S. Mendelian randomization with invalid instruments: effect estimation and bias detection through Egger regression. Int J Epidemiol. 2015;44(2):512–25. 10.1093/ije/dyv080 26050253 PMC4469799

[37] Bowden J, Davey Smith G, Haycock PC, Burgess S. Consistent estimation in Mendelian randomization with some invalid instruments using a weighted median estimator. Genet Epidemiol. 2016;40(4):304–14. 10.1002/gepi.21965 27061298 PMC4849733

[38] Bradfield JP, Taal HR, Timpson NJ, Scherag A, Lecoeur C, Warrington NM, et al A genome-wide association meta-analysis identifies new childhood obesity loci. Nat Genet. 2012;44(5):526 10.1038/ng.2247 22484627 PMC3370100

[39] Gill D, Brewer CF, Fabiola Del Greco M, Sivakumaran P, Bowden J, Sheehan NA, et al Age at menarche and adult body mass index: a Mendelian randomization study. Int J Obes (Lond). 2018 2 26 10.1038/s41366-018-0048-7 29549348

[40] Bjerregaard LG, Jensen BW, Ängquist L, Osler M, Sørensen TI, Baker JL. Change in overweight from childhood to early adulthood and risk of type 2 diabetes. N Engl J Med. 2018;378(14):1302–12. 10.1056/NEJMoa1713231 29617589

[41] Cooper R, Blell M, Hardy R, Black S, Pollard T, Wadsworth M, et al Validity of age at menarche self-reported in adulthood. J Epidemiol Community Health. 2006;60(11):993–7. 10.1136/jech.2005.043182 17053289 PMC2465480

[42] Ong KK, Bann D, Wills AK, Ward K, Adams JE, Hardy R, et al Timing of voice breaking in males associated with growth and weight gain across the life course. J Clin Endocrinol Metab. 2012;97(8):2844–52. 10.1210/jc.2011-3445 22654120 PMC3579950

[43] Dumith SC, Gigante DP, Domingues MR, Kohl HW. Physical activity change during adolescence: a systematic review and a pooled analysis. Int J Epidemiol. 2011;40(3):685–98. 10.1093/ije/dyq272 21245072

[44] Davey Smith G, Lawlor DA, Harbord R, Timpson N, Day I, Ebrahim S. Clustered environments and randomized genes: a fundamental distinction between conventional and genetic epidemiology. PLoS Med. 2007;4(12):e352 10.1371/journal.pmed.0040352 18076282 PMC2121108

[45] Peters SA, Woodward M. Women’s reproductive factors and incident cardiovascular disease in the UK Biobank. Heart. 2018;104(13):1069–75. 10.1136/heartjnl-2017-312289 29335253

[46] Day FR, Elks CE, Murray A, Ong KK, Perry JR. Puberty timing associated with diabetes, cardiovascular disease and also diverse health outcomes in men and women: the UK Biobank study. Sci Rep. 2015;5:11208 10.1038/srep11208 26084728 PMC4471670

[47] Global BMI Mortality Collaboration. Body-mass index and all-cause mortality: individual-participant-data meta-analysis of 239 prospective studies in four continents. Lancet. 2016;388(10046):776–86. 10.1016/S0140-6736(16)30175-1 27423262 PMC4995441

[48] Wood AM, Kaptoge S, Butterworth AS, Willeit P, Warnakula S, Bolton T, et al Risk thresholds for alcohol consumption: combined analysis of individual-participant data for 599 912 current drinkers in 83 prospective studies. Lancet. 2018;391(10129):1513–23. 10.1016/S0140-6736(18)30134-X 29676281 PMC5899998

[49] Carslake D, Davey Smith G, Gunnell D, Davies N, Nilsen TI, Romundstad P. Confounding by ill health in the observed association between BMI and mortality: evidence from the HUNT Study using offspring BMI as an instrument. Int J Epidemiol. 2017 12 1 10.1093/ije/dyx246 PMC6005033 29206928

[50] Davey Smith G, Sterne JA, Fraser A, Tynelius P, Lawlor DA, Rasmussen F. The association between BMI and mortality using offspring BMI as an indicator of own BMI: large intergenerational mortality study. BMJ. 2009;339:b5043 10.1136/bmj.b5043 20028778 PMC2797052

[51] Holmes MV, Dale CE, Zuccolo L, Silverwood RJ, Guo Y, Ye Z, et al Association between alcohol and cardiovascular disease: Mendelian randomisation analysis based on individual participant data. BMJ. 2014;349:g4164 10.1136/bmj.g4164 25011450 PMC4091648

[52] Chen L, Davey Smith G, Harbord RM, Lewis SJ. Alcohol intake and blood pressure: a systematic review implementing a Mendelian randomization approach. PLoS Med. 2008;5(3):e52 10.1371/journal.pmed.0050052 18318597 PMC2265305

[53] Tilling K, Macdonald-Wallis C, Lawlor DA, Hughes RA, Howe LD. Modelling childhood growth using fractional polynomials and linear splines. Ann Nutr Metab. 2014;65(2–3):129–38. 10.1159/000362695 25413651 PMC4264511

[54] Burgess S, Davies NM, Thompson SG. Bias due to participant overlap in two‐sample Mendelian randomization. Genet Epidemiol. 2016;40(7):597–608. 10.1002/gepi.21998 27625185 PMC5082560

[55] Pierce BL, Burgess S. Efficient design for Mendelian randomization studies: subsample and 2-sample instrumental variable estimators. Am J Epidemiol. 2013;178(7):1177–84. 10.1093/aje/kwt084 23863760 PMC3783091

