# Supplementary material for: Influence of puberty timing on adiposity and cardiometabolic traits: A Mendelian randomisation study
Source: PLoS Med. 2018 Aug 28;15(8):e1002641. doi: 10.1371/journal.pmed.1002641 (PMC6112630; doi:10.1371/journal.pmed.1002641)
Supplement: S1 STROBE Checklist — (PDF) [file pmed.1002641.s001.pdf]

STROBE Statement—Checklist of items that should be included in reports of ***cohort studies***

	Item No	Recommendation
**Title and abstract**	1	(*a*) Indicate the study's design with a commonly used term in the title or the abstract TITLE & ABSTRACT: PARA. 2
		(*b*) Provide in the abstract an informative and balanced summary of what was done and what was found ABSTRACT: PARA. 2 & 3
**Introduction**		
Background/rationale	2	Explain the scientific background and rationale for the investigation being reported INTRO: PARA. 1 & 2
Objectives	3	State specific objectives, including any prespecified hypotheses INTRO: PARA. 1, 2 & 3
**Methods**		
Study design	4	Present key elements of study design early in the paper METHODS: STATISTICAL ANALYSES SECTION & REPLICATION ANALYSES SECTION
Setting	5	Describe the setting, locations, and relevant dates, including periods of recruitment, exposure, follow-up, and data collection METHODS: STUDY POPULATION SECTION, ASSESSMENT OF PUBERTY TIMING SECTION, ASSESSMENT OF GENOTYPE AND GENETIC INSTRUMENTS SECTION, ASSESSMENT OF ADIPOSITY AND CARDIOMETABOLIC TRAITS SECTION & ASSESSMENT OF COVARIATES SECTION
Participants	6	(*a*) Give the eligibility criteria, and the sources and methods of selection of participants. Describe methods of follow-up METHODS: STUDY POPULATION SECTION, ASSESSMENT OF PUBERTY TIMING SECTION, ASSESSMENT OF GENOTYPE AND GENETIC INSTRUMENTS SECTION, ASSESSMENT OF ADIPOSITY AND CARDIOMETABOLIC TRAITS SECTION & ASSESSMENT OF COVARIATES SECTION
		(*b*) For matched studies, give matching criteria and number of exposed and unexposed NA
Variables	7	Clearly define all outcomes, exposures, predictors, potential confounders, and effect modifiers. Give diagnostic criteria, if applicable METHODS: STUDY POPULATION SECTION, ASSESSMENT OF PUBERTY TIMING SECTION, ASSESSMENT OF GENOTYPE AND GENETIC INSTRUMENTS SECTION, ASSESSMENT OF ADIPOSITY AND CARDIOMETABOLIC TRAITS SECTION & ASSESSMENT OF COVARIATES SECTION
Data sources/ measurement	8*	For each variable of interest, give sources of data and details of methods of assessment (measurement). Describe comparability of assessment methods if there is more than one group METHODS: STUDY POPULATION SECTION, ASSESSMENT OF PUBERTY TIMING SECTION, ASSESSMENT OF GENOTYPE AND GENETIC INSTRUMENTS SECTION, ASSESSMENT OF ADIPOSITY AND CARDIOMETABOLIC TRAITS SECTION & ASSESSMENT OF COVARIATES SECTION
Bias	9	Describe any efforts to address potential sources of bias METHODS: STATISTICAL ANALYSES SECTION & REPLICATION ANALYSES SECTION
Study size	10	Explain how the study size was arrived at METHODS: STATISTICAL ANALYSES SECTION & REPLICATION ANALYSES SECTION
Quantitative variables	11	Explain how quantitative variables were handled in the analyses. If applicable, describe which groupings were chosen and why METHODS: STATISTICAL ANALYSES SECTION & REPLICATION ANALYSES SECTION

Statistical methods	12	(*a*) Describe all statistical methods, including those used to control for confounding METHODS: STATISTICAL ANALYSES SECTION & REPLICATION ANALYSES SECTION
		(*b*) Describe any methods used to examine subgroups and interactions METHODS: STATISTICAL ANALYSES SECTION & REPLICATION ANALYSES SECTION
		(*c*) Explain how missing data were addressed METHODS: STATISTICAL ANALYSES SECTION
		(*d*) If applicable, explain how loss to follow-up was addressed METHODS: STATISTICAL ANALYSES SECTION
		(*e*) Describe any sensitivity analyses METHODS: STATISTICAL ANALYSES SECTION & REPLICATION ANALYSES SECTION
**Results**		
Participants	13*	(a) Report numbers of individuals at each stage of study—eg numbers potentially eligible, examined for eligibility, confirmed eligible, included in the study, completing follow-up, and analysed RESULTS: SAMPLE CHARACTERISTICS SECTION
		(b) Give reasons for non-participation at each stage RESULTS: SAMPLE CHARACTERISTICS SECTION & S2 TABLE
		(c) Consider use of a flow diagram NA
Descriptive data	14*	(a) Give characteristics of study participants (eg demographic, clinical, social) and information on exposures and potential confounders RESULTS: SAMPLE CHARACTERISTICS SECTION, TABLE 1 & S1 TABLE
		(b) Indicate number of participants with missing data for each variable of interest S2 TABLE
		(c) Summarise follow-up time (eg, average and total amount) RESULTS: SAMPLE CHARACTERISTICS SECTION, TABLE 1 & S1 TABLE
Outcome data	15*	Report numbers of outcome events or summary measures over time RESULTS: TABLE 1, S1-S15 TABLE
Main results	16	(*a*) Give unadjusted estimates and, if applicable, confounder-adjusted estimates and their precision (eg, 95% confidence interval). Make clear which confounders were adjusted for and why they were included RESULTS: TABLE 1, S1-S15 TABLE
		(*b*) Report category boundaries when continuous variables were categorized RESULTS: TABLE 1
		(*c*) If relevant, consider translating estimates of relative risk into absolute risk for a meaningful time period NA
Other analyses	17	Report other analyses done—eg analyses of subgroups and interactions, and sensitivity analyses RESULTS: TABLE 1, S1-S15 TABLE
**Discussion**		
Key results	18	Summarise key results with reference to study objectives DISCUSSION: PARA. 1-6
Limitations	19	Discuss limitations of the study, taking into account sources of potential bias or imprecision. Discuss both direction and magnitude of any potential bias DISCUSSION: STRENGTHS AND LIMITATIONS SECTION
Interpretation	20	Give a cautious overall interpretation of results considering objectives, limitations, multiplicity of analyses, results from similar studies, and other relevant evidence DISCUSSION: CONCLUSIONS SECTION
Generalisability	21	Discuss the generalisability (external validity) of the study results DISCUSSION: PARA. 1-6 & STRENGTHS AND LIMITATIONS SECTION
**Other information**		
Funding	22	Give the source of funding and the role of the funders for the present study and, if

	applicable, for the original study on which the present article is based ONLINE

*Give information separately for exposed and unexposed groups.

Note: An Explanation and Elaboration article discusses each checklist item and gives methodological background and published examples of transparent reporting. The STROBE checklist is best used in conjunction with this article (freely available on the Web sites of PLoS Medicine at http://www.plosmedicine.org/, Annals of Internal Medicine at http://www.annals.org/, and Epidemiology at http://www.epidem.com/). Information on the STROBE Initiative is available at http://www.strobe-statement.org.
