# Supplementary figures and images for: Influence of puberty timing on adiposity and cardiometabolic traits: A Mendelian randomisation study
Source: PLoS Med. 2018 Aug 28;15(8):e1002641. doi: 10.1371/journal.pmed.1002641 (PMC6112630; doi:10.1371/journal.pmed.1002641)

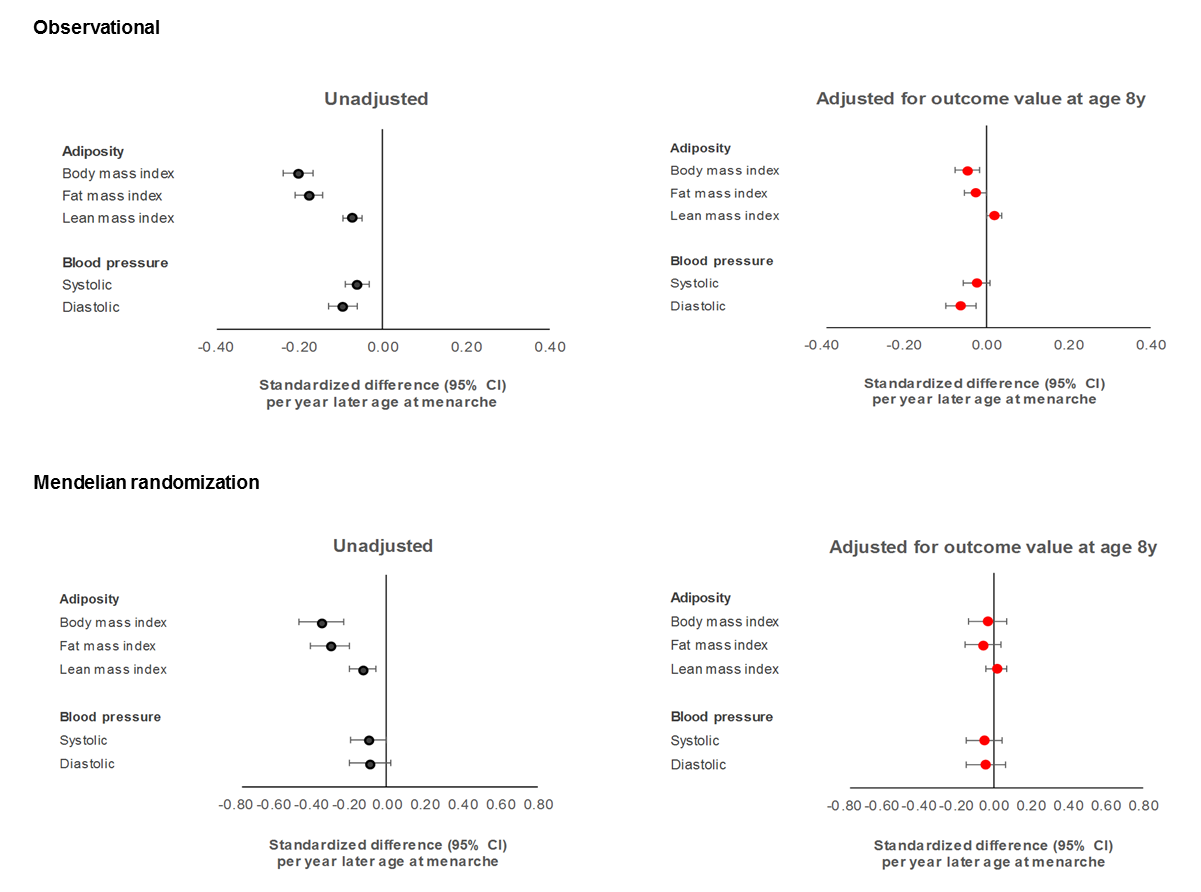

Supplement: S1 Fig — (TIF) [file pmed.1002641.s002.tif]

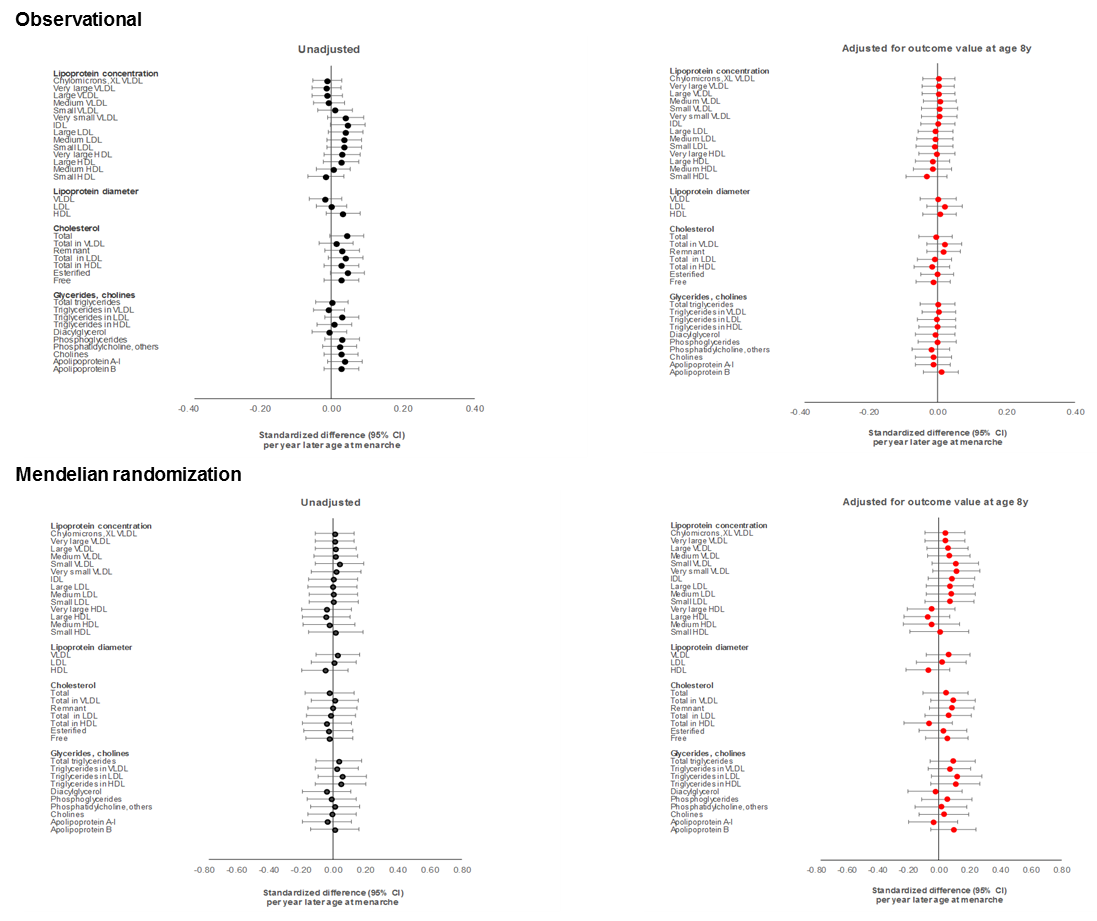

Supplement: S2 Fig — (TIF) [file pmed.1002641.s003.tif]

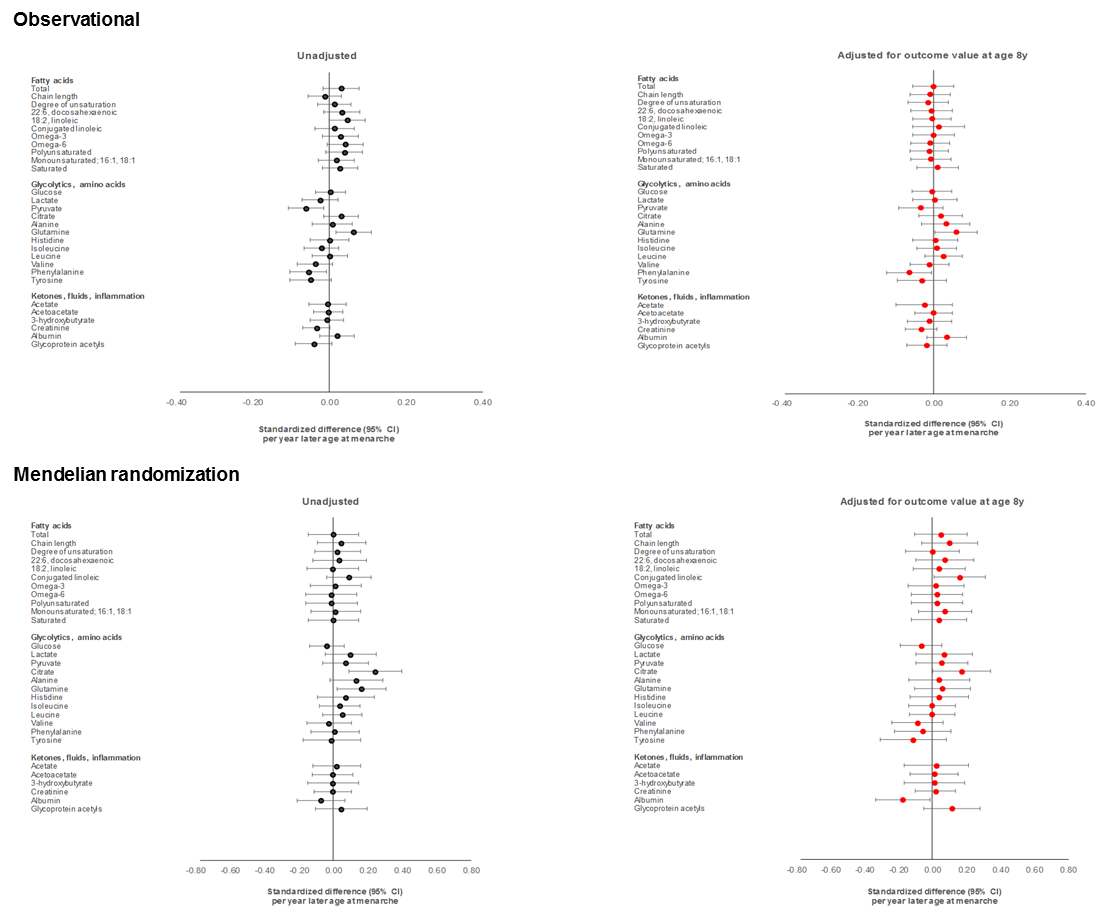

Supplement: S3 Fig — (TIF) [file pmed.1002641.s004.tif]

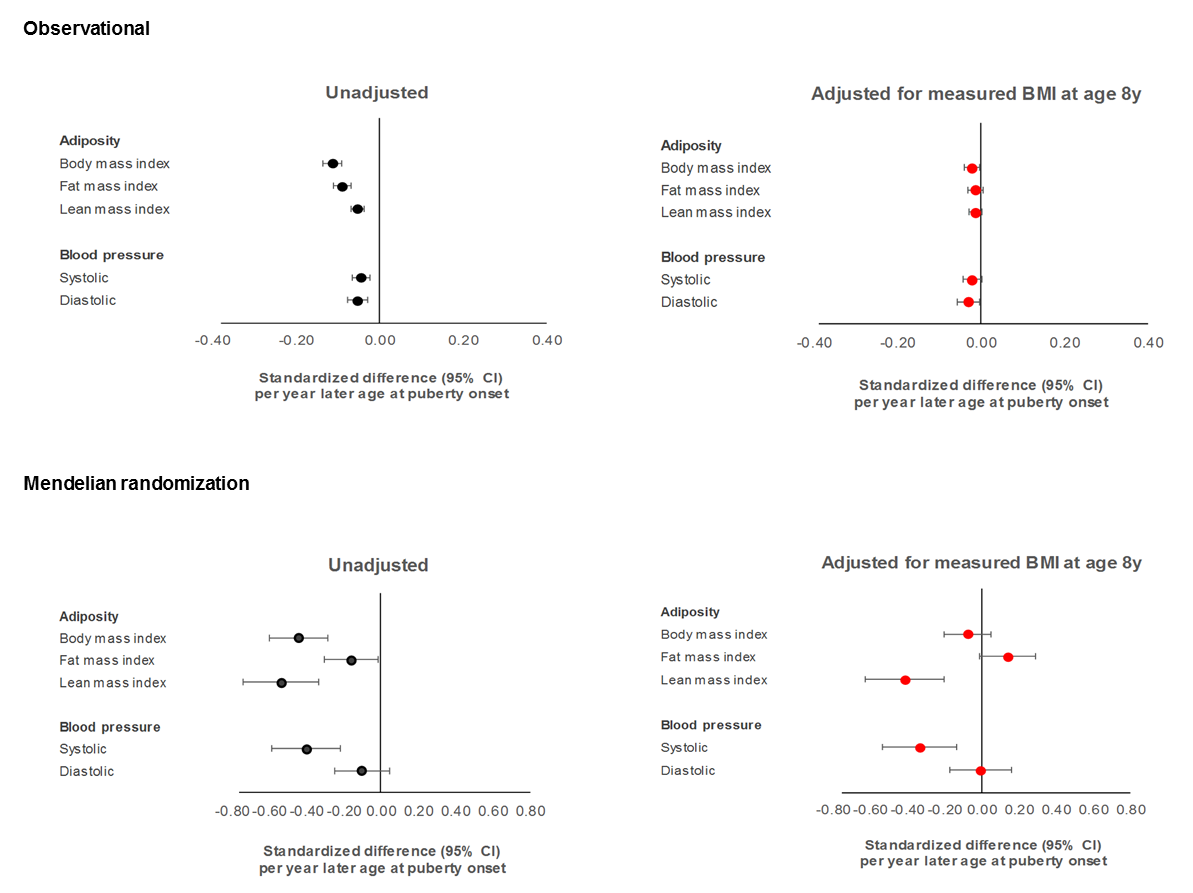

Supplement: S4 Fig — (TIF) [file pmed.1002641.s005.tif]

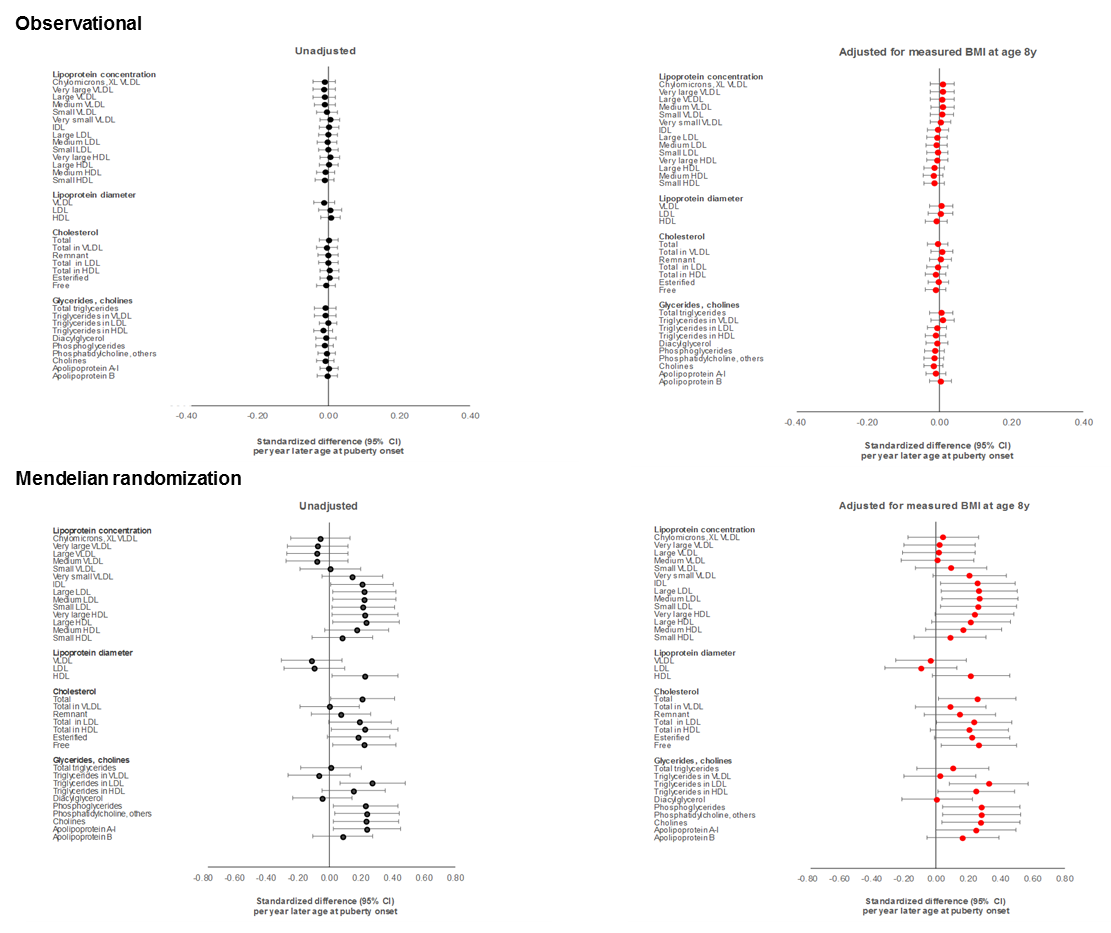

Supplement: S5 Fig — (TIF) [file pmed.1002641.s006.tif]

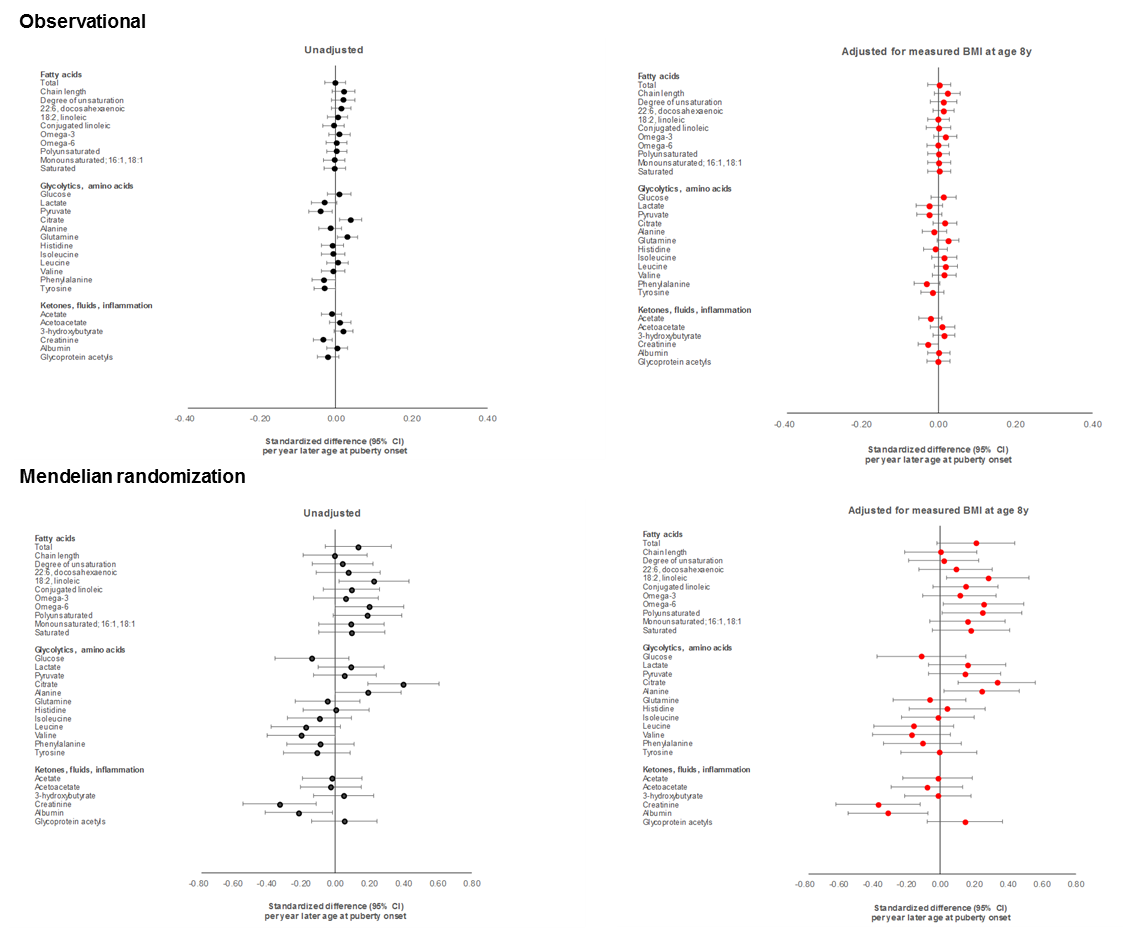

Supplement: S6 Fig — (TIF) [file pmed.1002641.s007.tif]

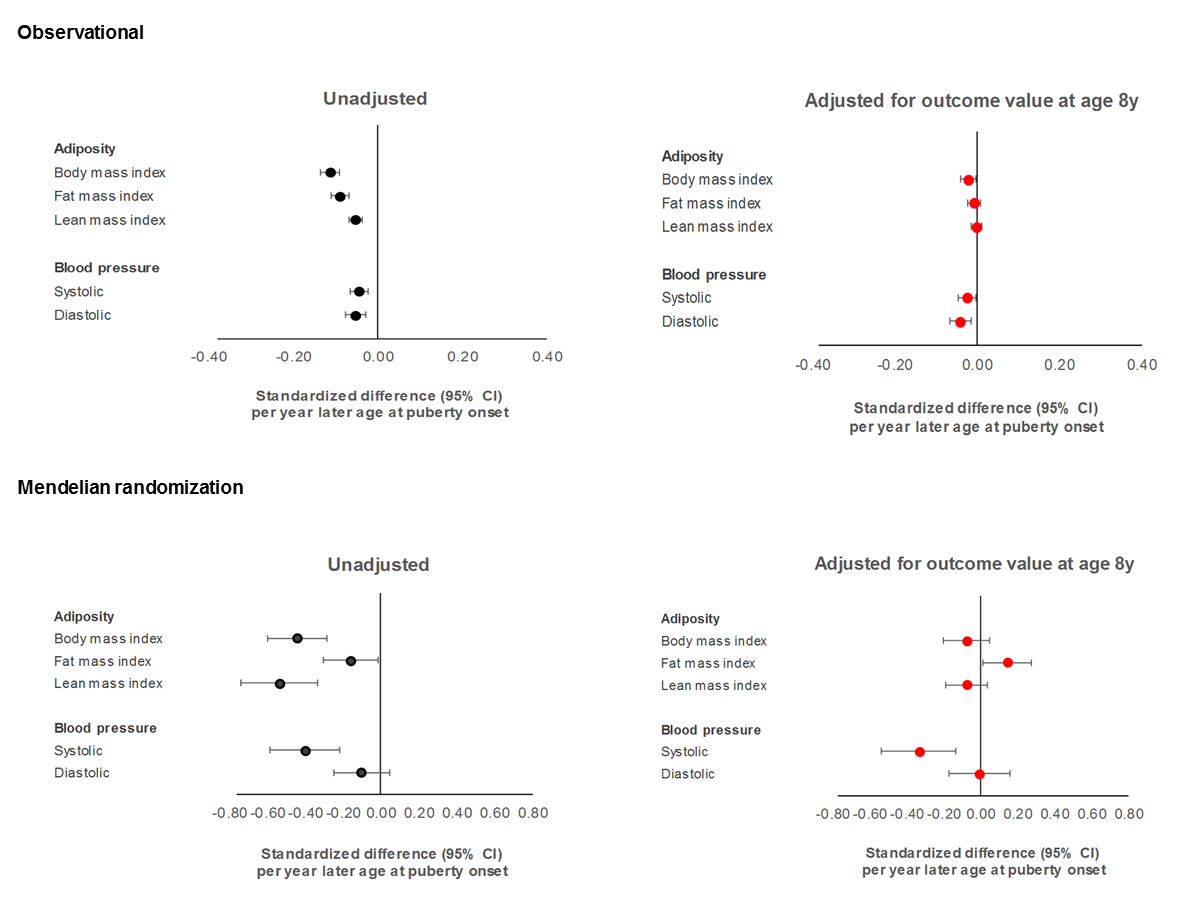

Supplement: S7 Fig — (TIF) [file pmed.1002641.s008.tif]

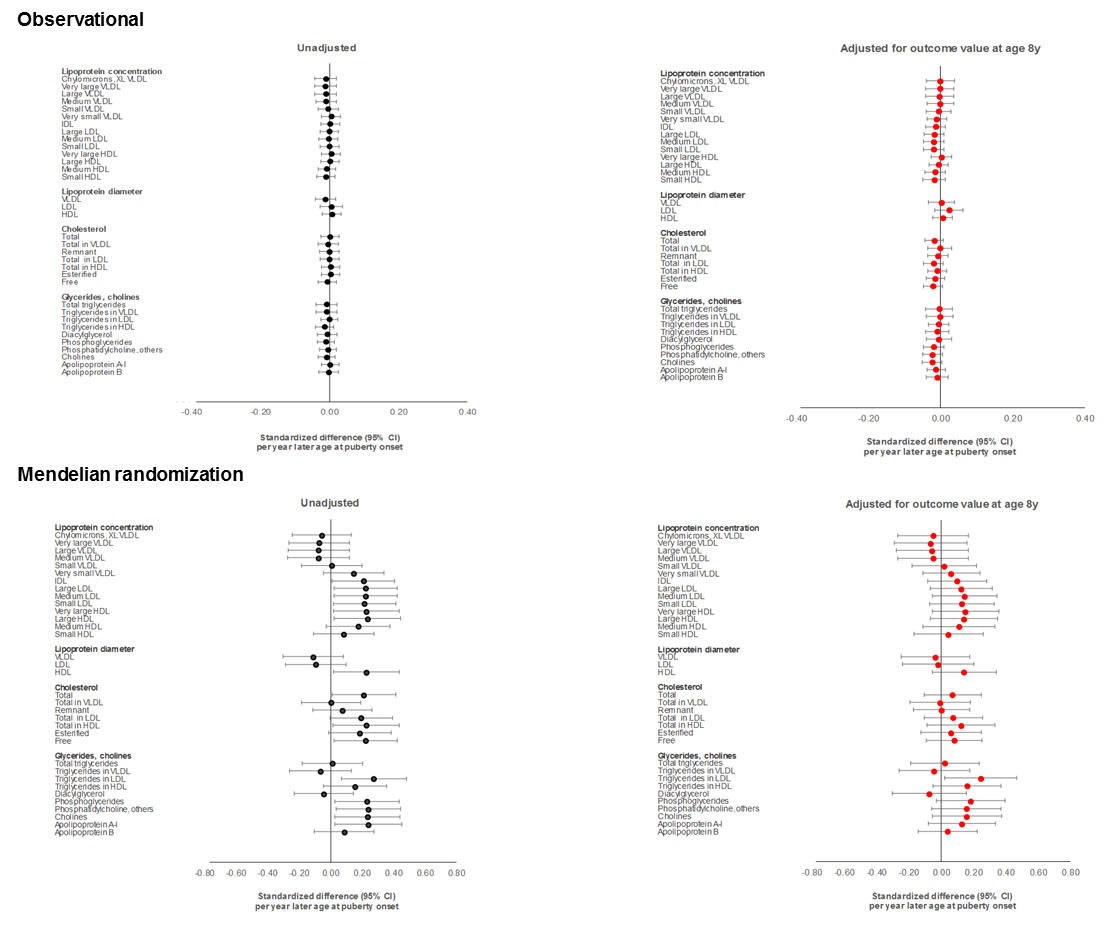

Supplement: S8 Fig — (TIF) [file pmed.1002641.s009.tif]

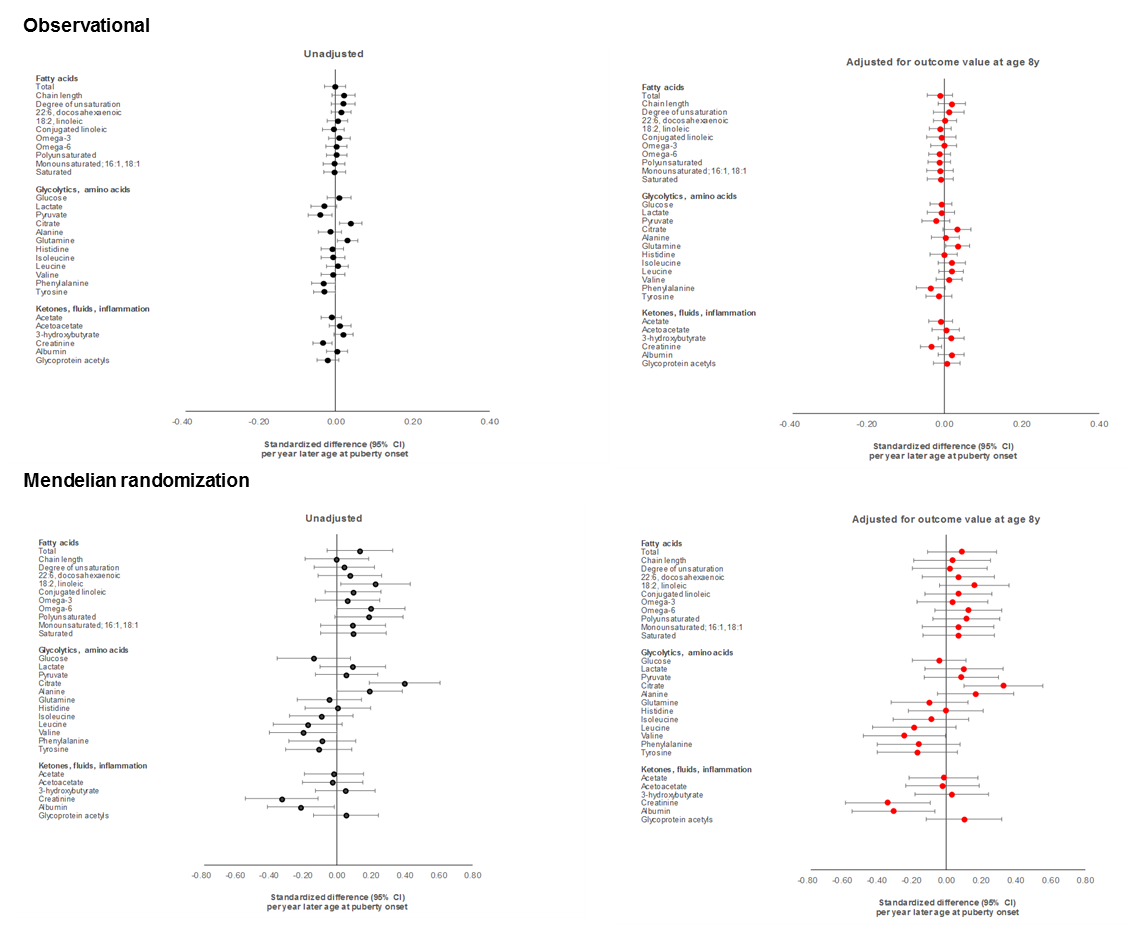

Supplement: S9 Fig — (TIF) [file pmed.1002641.s010.tif]

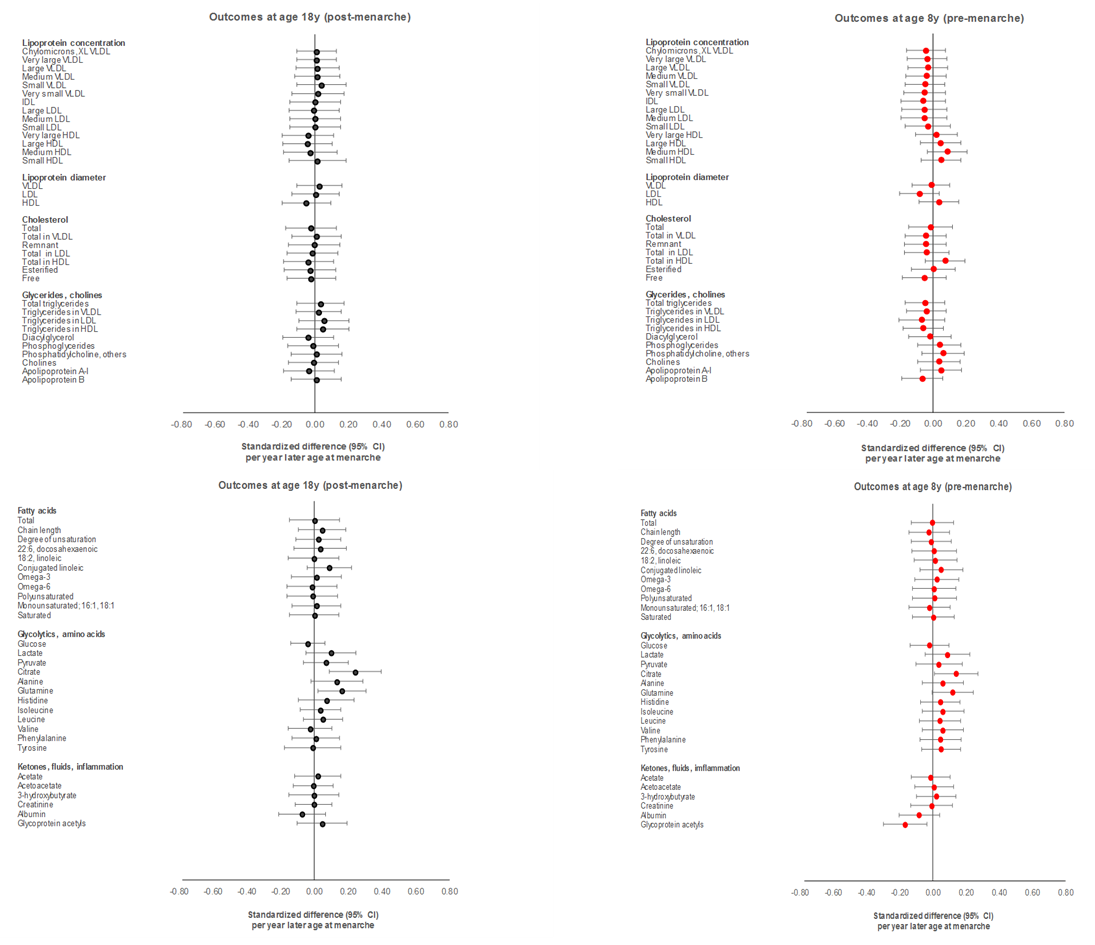

Supplement: S10 Fig — (TIF) [file pmed.1002641.s011.tif]

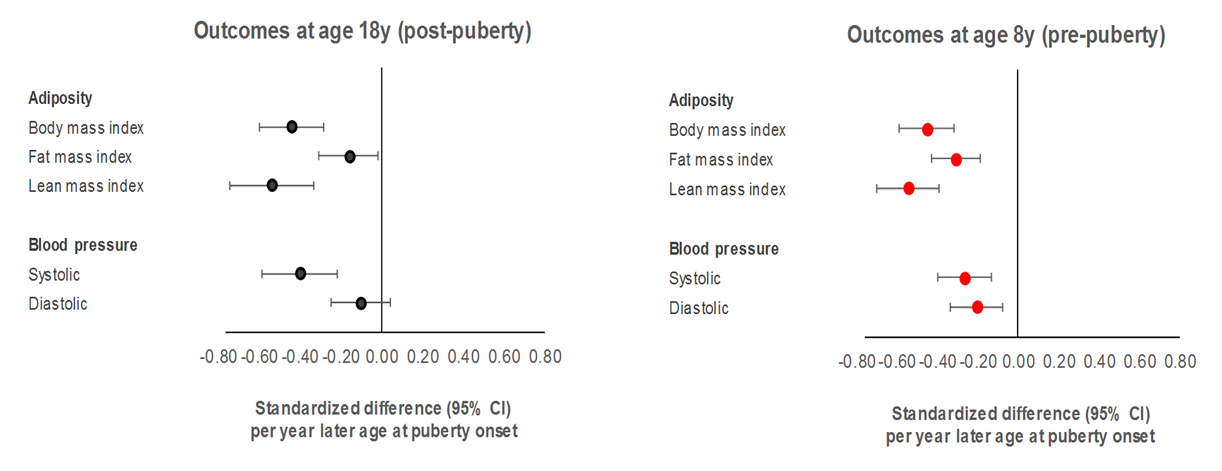

Supplement: S11 Fig — (TIF) [file pmed.1002641.s012.tif]

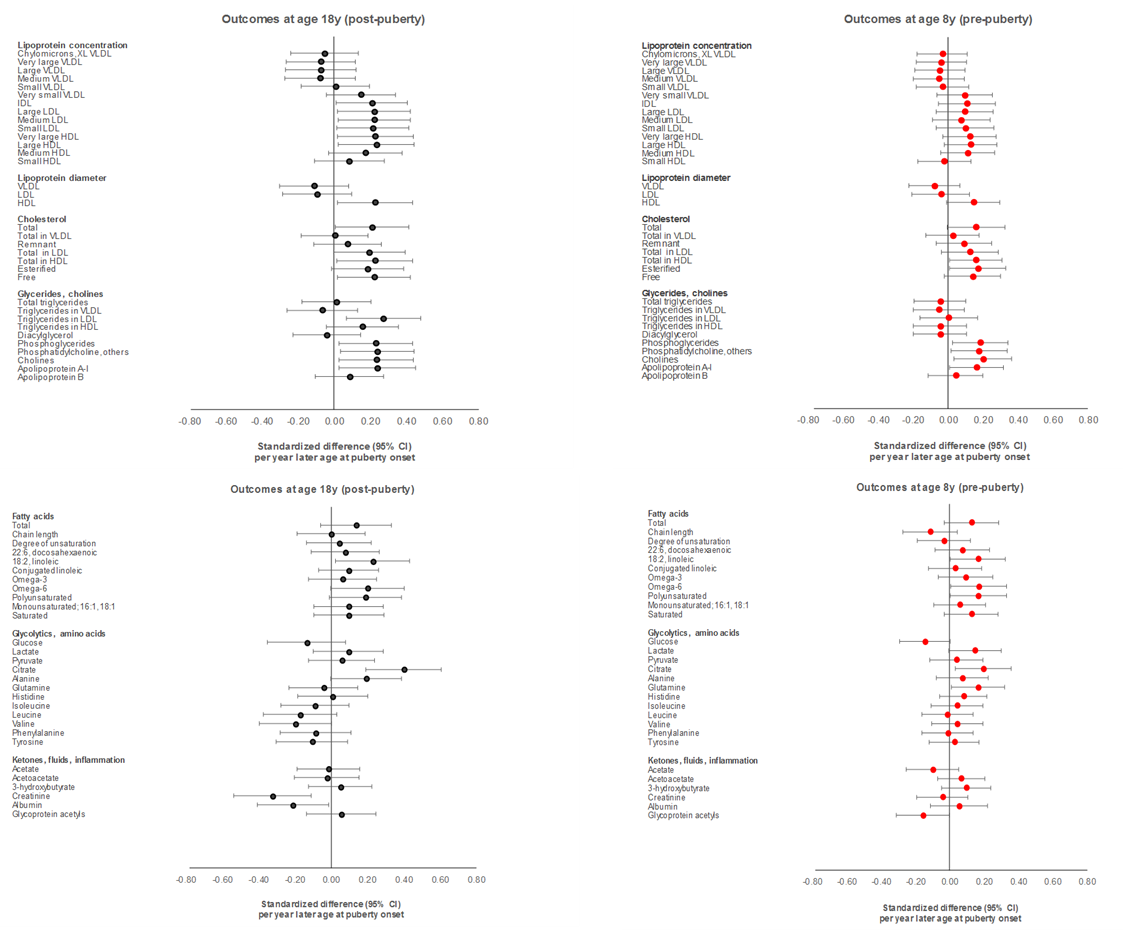

Supplement: S12 Fig — (TIF) [file pmed.1002641.s013.tif]

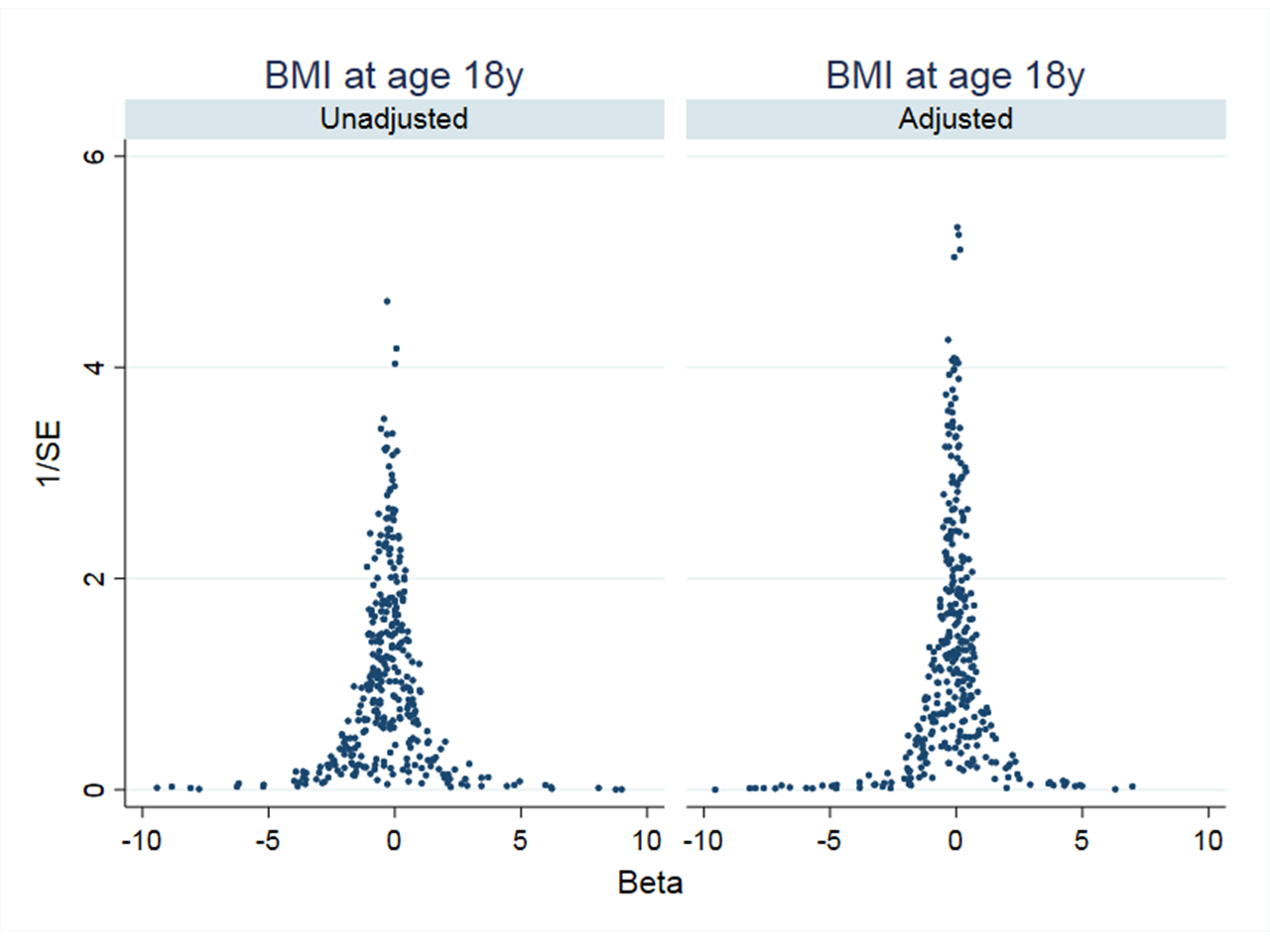

Supplement: S13 Fig — (TIF) [file pmed.1002641.s014.tif]

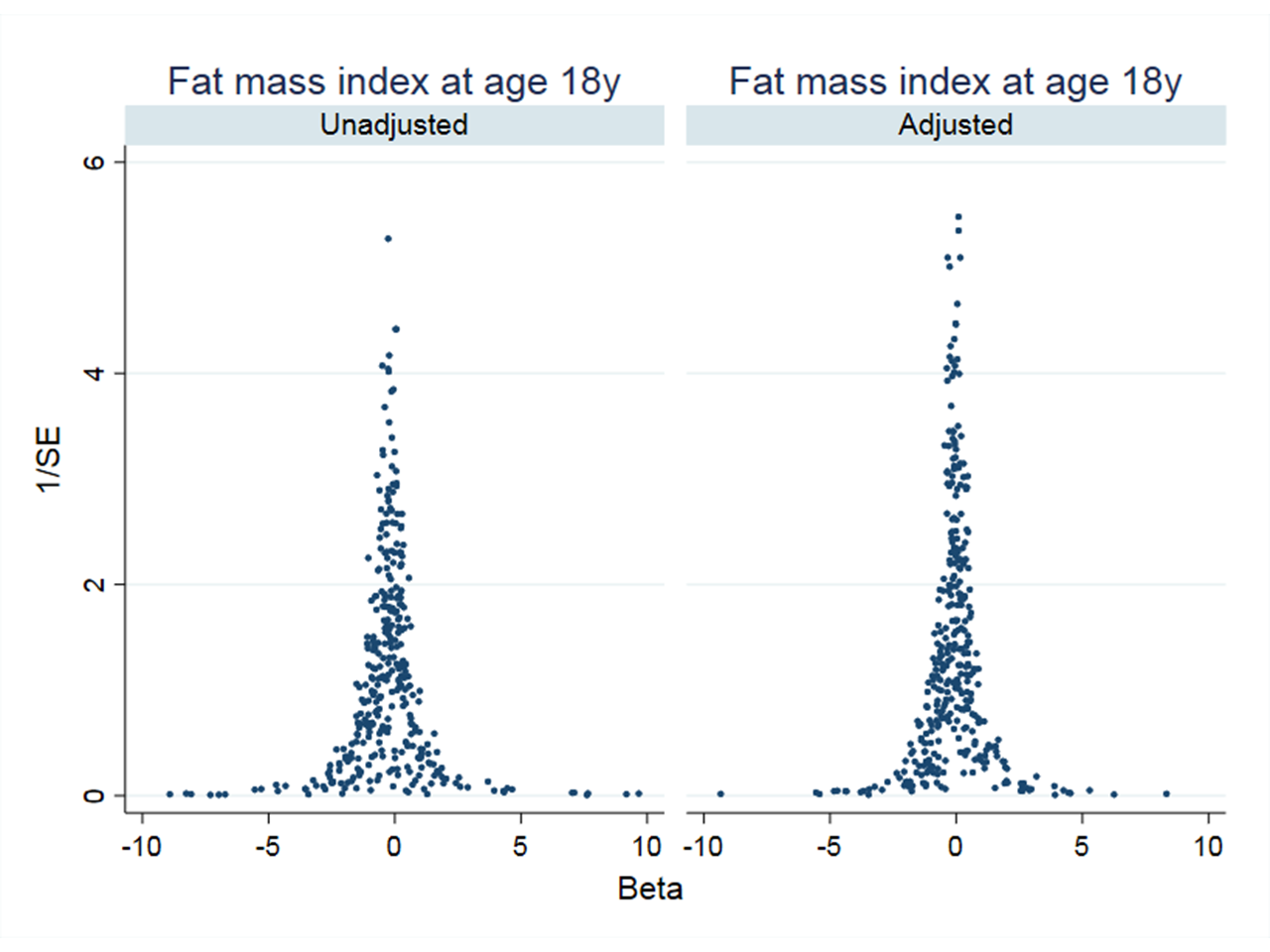

Supplement: S14 Fig — (TIF) [file pmed.1002641.s015.tif]

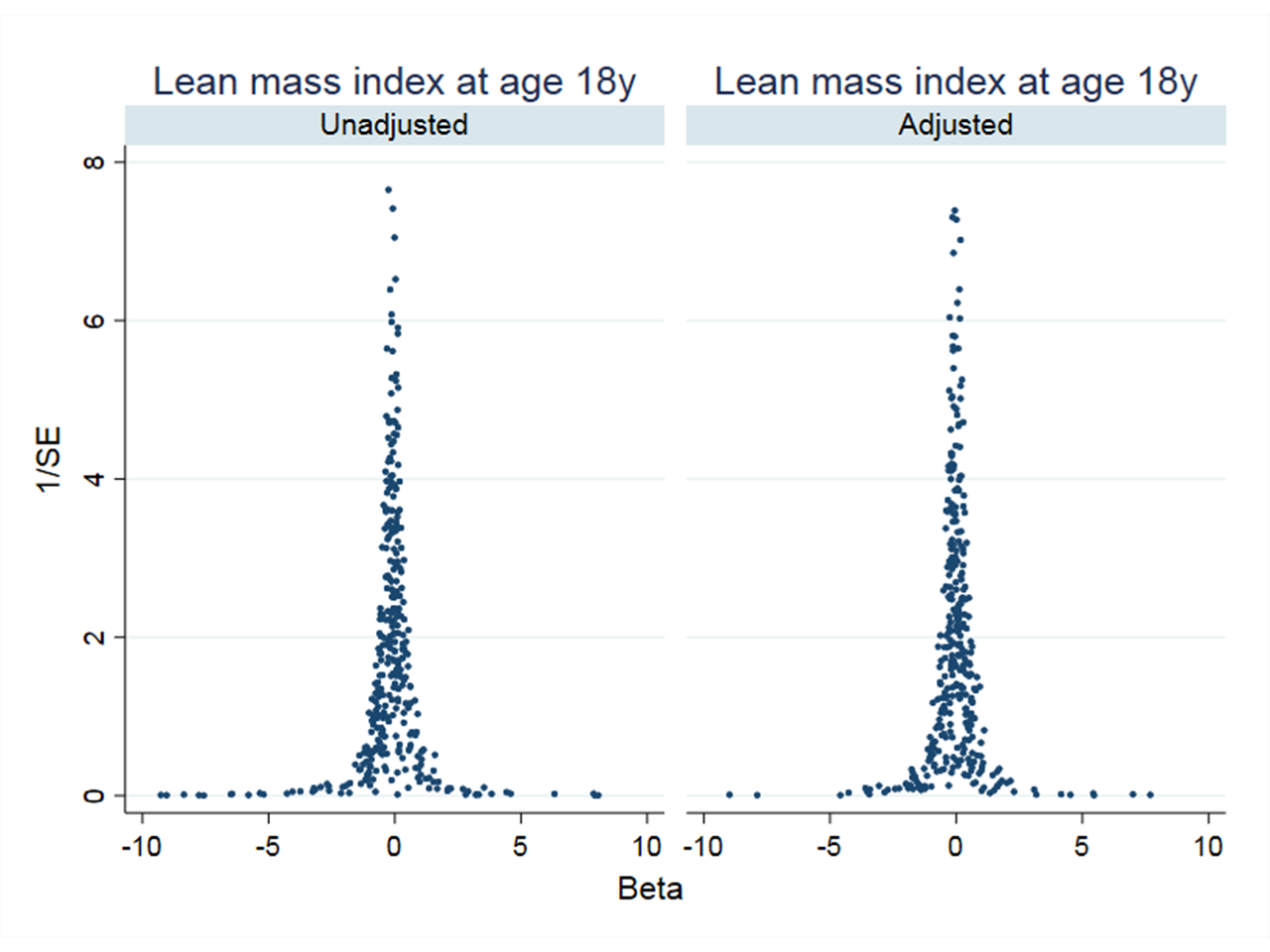

Supplement: S15 Fig — (TIF) [file pmed.1002641.s016.tif]

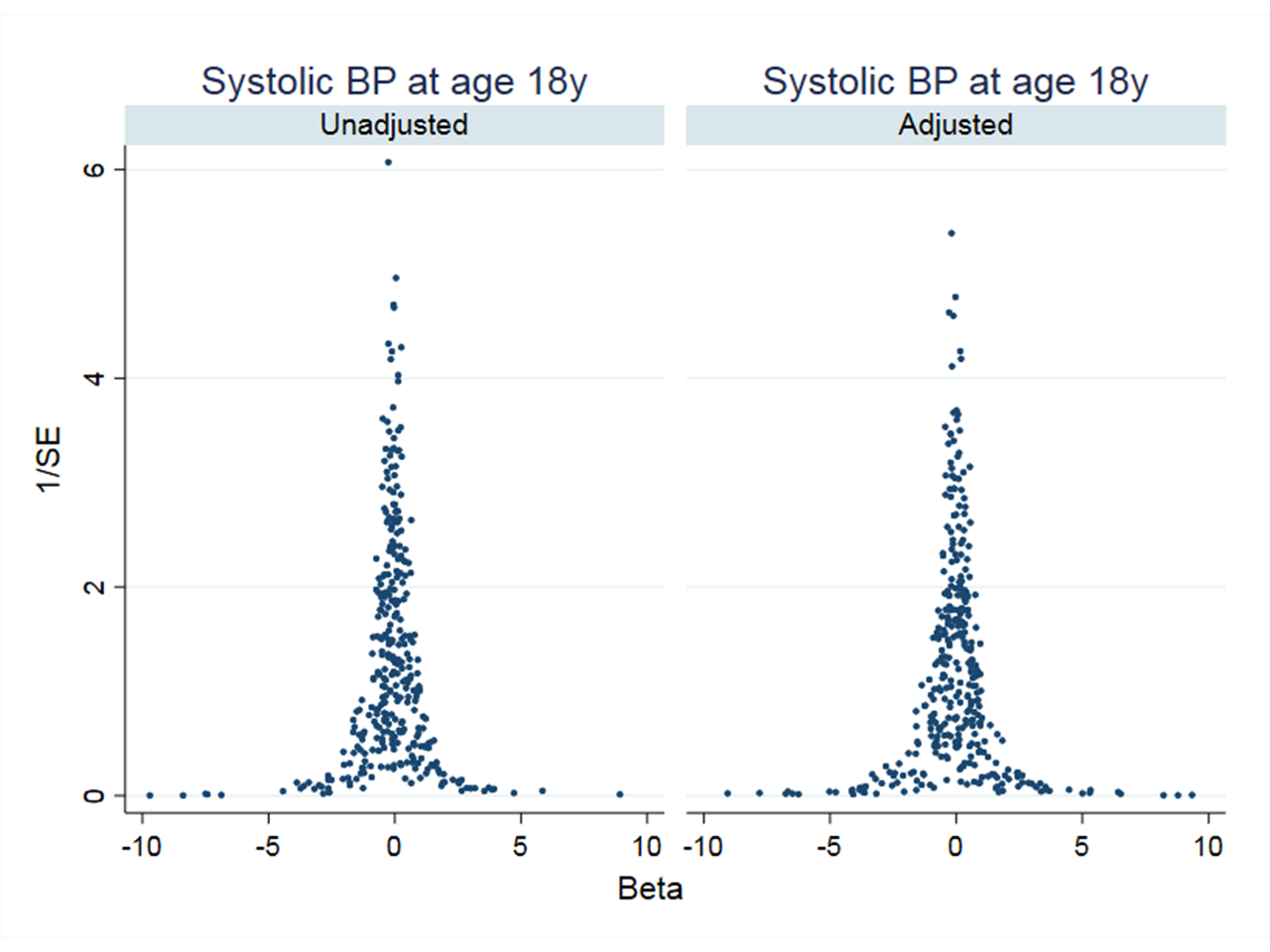

Supplement: S16 Fig — (TIF) [file pmed.1002641.s017.tif]

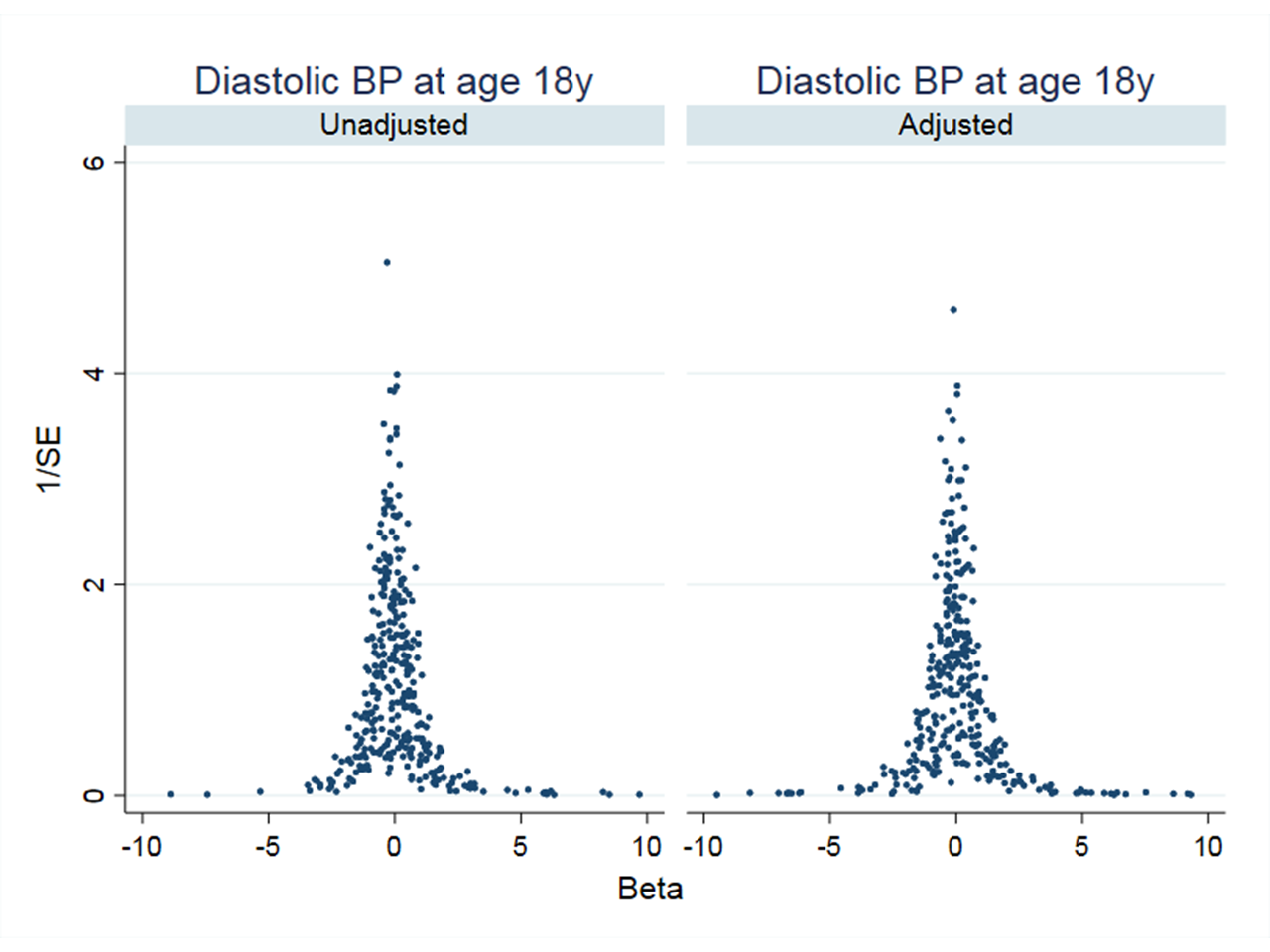

Supplement: S17 Fig — (TIF) [file pmed.1002641.s018.tif]
