# Supplementary material for: Influence of puberty timing on adiposity and cardiometabolic traits: A Mendelian randomisation study
Source: PLoS Med. 2018 Aug 28;15(8):e1002641. doi: 10.1371/journal.pmed.1002641 (PMC6112630; doi:10.1371/journal.pmed.1002641)
Supplement: S1 Protocol — (PDF) [file pmed.1002641.s019.pdf]

Submission information

Effects of puberty timing on the metabolome (B2801)

Submission date/time: 02/12/2016 02:53:24

Principal Applicant Details

Principal Applicant Title: Dr

Your name: Joshua Bell

Position Held: Research Associate, Integrative Cancer Epidemiology Programme

Affiliation: IEU, University of Bristol

Are you a 'direct access' user of ALSPAC data, or a member of the Integrative Epidemiology Unit (IEU)?: Yes

Address line 1: Oakfield House

Address line 2:

City: Bristol

Country: UK

Postcode: BS8 2BN

Email: j.bell@bristol.ac.uk

Telephone:

PhD primary supervisor (if relevant)

Co-applicant details

How many co-applicants?: 1

Co-applicant 1

Name and title: Prof George Davey Smith
Position Held and Affiliation: Professor and Director of IEU, University of Bristol
Email: kz.davey-smith@bristol.ac.uk
Role in Project: Line manager / Project supervisor
Data use: please tick this box if the co-applicant will use the data. If this field is empty, the co-applicant will not use the data: Yes

Co-applicant 2

Co-applicant 3

Co-applicant 4

Co-applicant 5

Co-applicant 6

Co-applicant 7

Co-applicant 8

Co-applicant 9

Co-applicant 10

PROJECT DETAILS

Title of project: Effects of puberty timing on the metabolome

Proposed Project Start Date: 2017-01-01

Proposed Project Finish Date: 2017-07-01

Is your project currently funded?: Yes

Current project funding: CRUK Integrative Cancer Epidemiology Programme, IEU, University of Bristol

Are you seeking funding for this project?: No

----------------Potential funding fields----------------

Have you previously had a project with us?: No

Where did you find out about ALSPAC?:

Where did you hear about ALSPAC (please specify)?:

Data buddy name (if one has been assigned to you):

Research dissemination: Peer review journal article, Conference

Other plans for disseminating research findings:

----------------Invoice details----------------
- **Name:** George Davey Smith

- **Position Held:** Professor and Director of IEU, University of Bristol

- **Address:** Oakfield House, Bristol, BS8 2BN

- **Email:** kz.davey-smith@bristol.ac.uk

- **Telephone number:**

Description of Project

Project summary for laypersons: Early puberty may lead to the development of several different types of cancer in both men and women. This project aims to find out whether this increased cancer risk is partly due to adverse effects of early puberty on metabolism. To do this, we will examine links between genetic predispositions for timing of puberty and markers of body fat, along with a wide range of detailed blood-based markers of metabolic functioning. Data on ALSPAC mothers and children are particularly useful for seeing how genetic predispositions to early puberty affect fat and lean mass differently, and how these affect metabolic functioning at different stages of sexual maturity. Data on men from a separate cancer-specific study will also be examined to see how development of cancer affects links between puberty timing and metabolism. All results will be compared with those obtained from a separate analysis of genetic predispositions for puberty timing on metabolites using larger-scale collaborative data.

Aim(s) and objective(s): The aims of this project are as follows:

1. To examine whether early puberty adversely affects metabolic functioning

2. To examine whether effects of early puberty on metabolic functioning differ by sex and stage of sexual maturity

3. To examine whether early puberty has differential effects on fat and lean mass

4. To examine whether the development of cancer in adulthood leads to differential effects of puberty timing on metabolic functioning

These aims translate into the following objectives:

1. To test associations of a genetic risk score for puberty timing (based on genome-wide significant variants for age at menarche and age at voice breaking) with body mass index, waist-hip ratio, summary metabolic traits, and levels of circulating metabolites using summary-level consortium data held in the MR-Base platform

2. To test whether a genetic risk score for puberty timing is differentially associated with fat and lean mass based on DXA scans among ALSPAC mothers and children

3. To test whether a genetic risk score for puberty timing is differentially associated with body mass index, waist-hip ratio, summary metabolic traits, and metabolites based on sex and stage of sexual maturity among ALSPAC mothers and children

4. To test whether a genetic risk score for puberty timing is differentially associated with body mass index, waist-hip ratio, summary metabolic traits, and circulating metabolites based on the presence or absence of prostate cancer among men in the ProtecT study

Methods (including an overview of statistical methods): A genetic risk score for puberty timing will be constructed based on 389 genome-wide significant SNPs for age at menarche in women discovered through genome-wide association analyses by Day et al. (BioArxiv, pre-print). This score will be refined in at least 2 ways; the first will contain the 127 age at menarche SNPs from the same source paper that were also associated with age at voice breaking in men; the second will exclude those of the 389 age at menarche SNPs that are also associated with body mass index (at $p<0.05$) based on summary data from the GIANT consortium (Locke et al. 2015, Nature).

Associations of these genetic risk scores with anthropometric and metabolic outcomes will be examined first based on summary-level consortium data using inverse variance weighted and other sensitivity models (i.e. MR-Egger) through the MR-Base platform. These association tests will then be repeated using individual-level data from ALSPAC mothers and children to examine whether the puberty timing genetic risk score affects fat and lean mass differently based on DXA scans, and whether effects also differ by sex and stage of sexual maturity (using repeated measures on children from pre-puberty/age 7 and post-puberty/age 15 or 17, sufficient data depending). Association tests of the early puberty genetic risk score on anthropometric and metabolite outcomes will also be repeated using individual-level data from the ProtecT study, to examine how effects differ among men who

were or where not subsequently diagnosed with prostate cancer.

All outcome variables would be standardised (mean=0, standard deviation=1) for models in order to allow comparability of effect sizes. Models would be adjusted for age and sex at a minimum (when not stratifying by sex), with effect modification by socioeconomic status and health behaviours potentially examined at a later stage.

Exposures, outcomes and confounders to be considered (justifying particular types of data as necessary): Exposures: Weighted and unweighted genetic risk score for puberty timing based on 389 genome-wide significant SNPs for age at menarche in women from Day et al. (2016 BioArxiv, pre-print), refined to 127 such SNPs that are also associated with age at voice breaking in men from the same source paper. This score will also be refined to exclude those of the 389 SNPs that are also associated ($p<0.05$) with body mass index based on summary data from the GIANT consortium (Locke et al. 2015, Nature).

Outcomes: Clinic measures of height, weight, BMI, waist-hip ratio, systolic and diastolic blood pressure, fasting blood glucose, fasting insulin, derived insulin sensitivity/resistance, HDL cholesterol, total cholesterol, LDL cholesterol, triglycerides, C-reactive protein, interleukin-6, DXA-derived total fat mass, DXA-derived total lean mass, and all NMR-derived blood-based metabolites.

Confounders: Age; sex; ethnicity; education level; occupation (or related socioeconomic marker); birth weight; prevalence, history, and family history of type 2 diabetes, cardiovascular disease, and cancer (all sites); smoking status; alcohol consumption; moderate-to-vigorous physical activity.

Reasons for using ALSPAC: The ALSPAC resource is highly suited for examining effects of puberty timing on metabolism, given the availability of comprehensive genotype data for the construction of genetic risk scores for puberty timing, in addition to a wide range of blood-based markers of metabolism in the form of both summary (traditional) metabolic traits and NMR-derived metabolites. Perhaps uniquely, ALSPAC also offers the chance to examine how effects differ by stage of sexual maturity by using data on mothers and on children (who have metabolite measures taken at both pre- and post-puberty). Detailed measures of body composition by way of DXA scans are also available, affording a more detailed view into differential effects of puberty timing on fat and lean mass than is afforded by BMI alone.

What do you think the likely impact of your research will be?: The immediate impact of this research will likely be at least 1 peer reviewed publication in a general medical journal (i.e. PLOS Medicine). We aim to present and discuss results in a way that is robust enough for academics but is accessible and relevant to clinicians and public health practitioners, ultimately encouraging greater attention to early life exposures and better prevention of disease.

Subject classification (please select one): Epidemiology

Other subject - please specify:

----------------Diseases/conditions (click to expand/collapse)----------------
- **Please tick all appropriate diseases/conditions:** Cancer, Diabetes, Obesity

- **Other disease/condition - please specify:**

----------------Techniques (click to expand/collapse)----------------
- **Please tick all appropriate techniques:** GWAS, Metabolomics, NMR, Statistical methods

- **Other techniques - please specify:**

----------------Keywords (click to expand/collapse)----------------
- **Please tick all appropriate keywords:** Biological samples -e.g. blood, cell lines, saliva, etc., Biomarkers - e.g. cotinine, fatty acids, haemoglobin, etc., BMI, Genetics - e.g. epigenetics, mendelian randomisation, UK10K, sequencing, etc., Metabolic - metabolism, Puberty

- **Other keyword(s) (please specify):**

- **Methods - please specify:**

Types of existing data required

Does your project involve analysing existing data?: Yes
Existing questionnaire data: Yes

Existing clinic data: Yes

Existing data from biological samples (not genetic): Yes

----------------Existing genome-wide SNP genotype data----------------
- **Existing genetics data:** Yes

- **Genome-wide 1000genomes imputed SNP data (recommended):** 1

- **Genome-wide SNP genotype cohort:**

- **Genome-wide HapMap imputed SNP data:**

- **HRC imputed data (beta):**

- **Specific genotypes:** Yes

- **SNPs:**

- **SNPs list:** 1409

- **Other variants (e.g. VNTRs):**

- **Other variants list:**

----------------Existing Methylation Data----------------
- **Methylation data:** No

----------------Linkage data----------------
- **Third party data (e.g. data provided by education/health organisations):** No

----------------Address Data----------------
- **Do you require any data linked to addresses?:** No

----------------Text data----------------
- **Text data:** No

NEW data or sample collection

Are you requesting the collection of new data and/or biosamples?: No
----------------New data/biosamples----------------
- **Please provide details of what data you want to collect:**

- **How will your data be collected?:**

Do you want new genotyping carried out by LGC?

SNP genotyping: No

----------------LGC genotyping info----------------

Do you want new genotyping or methylation data generated on the Illumina platform?

Do you require Illumina arrays to be run in the ALSPAC Laboratory?: No

----------------Illumina array info----------------

Do you require DNA samples to analyse elsewhere?

DNA analysis: No

----------------Dna samples info----------------

Other biological samples

I understand that all genotypes will be returned to ALSPAC and be made available to other researchers: Yes
Will the project require access to biological samples other than DNA?: No
1. Please confirm that...: ...you are familiar with the latest version of the ALSPAC access policy
2. Please confirm that...: ...you understand it is your responsibility to ensure that all members of your team working on this project complete confidentiality forms and that you inform ALSPAC of any changes to the team
3. Please confirm that...: ...you understand that data and samples from the ALSPAC resource cannot be used for commercial gain
4. Please confirm that...: ...you understand that you and your team must not pass on any data or samples awarded, or any derived variables or genotypes generated by this application to a third party (i.e. to anybody who is not included in the list of applicants on this form)
5. Please confirm that...: ...you aware that any third party seeking to use data, samples, or derived variables or genotypes arising from this application must approach ALSPAC to obtain access permission of their own
6. Please confirm that...: ...you understand that if a problem arises involving any misuse of the ALSPAC data or samples provided for this project that violates any of the terms and conditions specified by the Materials Transfer Agreement (MTA), Data Transfer Agreement (DTA) or confidentiality form that you (as principal applicant) have signed, you will be held responsible. This might result in you being excluded from using the ALSPAC resource in the future.
7. I understand that...: ...costs will be determined after the proposal has been approved and that I will not receive any data or samples until I have settled my invoice or provided a purchase order number.
8. I understand that...: ...all genotypes and/or data generated from biological samples will be returned to ALSPAC and be made available to other researchers.
9. I declare that...: ...I have no conflict of interest in relation to this research.
If you do have a conflict on interest, please declare it here:
Date: 2016-12-02
Print name (this will serve as your signature): Joshua Bell
