# Supplementary material for: Influence of puberty timing on adiposity and cardiometabolic traits: A Mendelian randomisation study
Source: PLoS Med. 2018 Aug 28;15(8):e1002641. doi: 10.1371/journal.pmed.1002641 (PMC6112630; doi:10.1371/journal.pmed.1002641)
Supplement: S1 Table — (PDF) [file pmed.1002641.s020.pdf]

S1 Table Characteristics of males by age at voice breaking in ALSPAC

	Age at voice breaking among males<br>Sample mean (SD) = 13.21y (1.58y)				
	**Earliest (n=598)**<br>(9.08y-12.58y)	**Intermediate (n=377)**<br>(12.67y-14.08y)	**Latest (n=524)**<br>(14.17y-16.67y)	**P-value**<br>(Intermediate vs Earliest)	**P-value**<br>(Latest vs Earliest)
Mother has no further academic education – n (%)	281 (46.99)	178 (47.21)	258 (49.24)	0.945	0.453
**After puberty onset (age 18y assessment)**					
Body mass index (kg/m^2) – mean (SD)	22.95 (3.92)	22.46 (3.74)	22.10 (3.73)	0.050	2.05E-04
Fat mass index (kg/m^2) – mean (SD)	4.52 (3.18)	4.20 (2.84)	4.10 (3.04)	0.109	0.026
Systolic blood pressure (mmHg) – mean (SD)	120.51 (9.14)	120.46 (9.04)	119.29 (8.85)	0.937	0.027
Diastolic blood pressure (mmHg) – mean (SD)	63.49 (6.59)	63.36 (5.88)	63.10 (6.62)	0.751	0.328
Triglycerides (mmol/l) – mean (SD)	0.91 (0.34)	0.91 (0.35)	0.90 (0.36)	0.978	0.834
HDL cholesterol (mmol/l) – mean (SD)	1.30 (0.19)	1.29 (0.17)	1.29 (0.18)	0.586	0.653
LDL cholesterol (mmol/l) – mean (SD)	0.95 (0.32)	0.90 (0.31)	0.89 (0.28)	0.054	0.004
Total cholesterol (mmol/l) – mean (SD)	3.33 (0.59)	3.25 (0.57)	3.22 (0.53)	0.064	0.005
Glucose (mmol/l) – mean (SD)	4.22 (0.35)	4.24 (0.44)	4.26 (0.86)	0.509	0.337
Glycoprotein acetyls (mmol/l) – mean (SD)	1.19 (0.13)	1.18 (0.12)	1.18 (0.13)	0.171	0.603
**Before puberty onset (age 8y assessment)**					
Body mass index (kg/m^2) – mean (SD)	16.23 (1.88)	16.04 (1.90)	15.74 (1.57)	0.152	4.30E-06
Fat mass index (kg/m^2) – mean (SD)	3.88 (2.20)	3.67 (2.21)	3.30 (2.06)	0.170	1.28E-05
Systolic blood pressure (mmHg) – mean (SD)	98.75 (9.05)	98.23 (9.46)	97.82 (8.56)	0.414	0.090
Diastolic blood pressure (mmHg) – mean (SD)	55.85 (6.69)	55.43 (7.08)	55.65 (6.69)	0.375	0.636
Triglycerides (mmol/l) – mean (SD)	1.04 (0.40)	0.97 (0.38)	1.03 (0.38)	0.029	0.773
HDL cholesterol (mmol/l) – mean (SD)	1.49 (0.22)	1.52 (0.20)	1.50 (0.22)	0.148	0.796
LDL cholesterol (mmol/l) – mean (SD)	1.16 (0.34)	1.17 (0.33)	1.16 (0.31)	0.585	0.830
Total cholesterol (mmol/l) – mean (SD)	3.82 (0.61)	3.84 (0.61)	3.83 (0.57)	0.664	0.736
Glucose (mmol/l) – mean (SD)	4.29 (0.57)	4.21 (0.46)	4.22 (0.51)	0.058	0.070
Glycoprotein acetyls (mmol/l) – mean (SD)	1.23 (0.14)	1.21 (0.13)	1.21 (0.13)	0.027	0.137

Sample sizes vary as participants described are those with data on age at voice breaking, covariates, at least one adiposity trait, and at least one metabolic trait.
‘No further academic education’ defined as Certificate of Secondary Education, vocational, or O-level versus A-level or degree.
