# Supplementary material for: Influence of puberty timing on adiposity and cardiometabolic traits: A Mendelian randomisation study
Source: PLoS Med. 2018 Aug 28;15(8):e1002641. doi: 10.1371/journal.pmed.1002641 (PMC6112630; doi:10.1371/journal.pmed.1002641)
Supplement: S2 Table — (PDF) [file pmed.1002641.s021.pdf]

**S2 Table** Characteristics of participants by missing data status on age at menarche/voice breaking in ALSPAC

| | **Among females** | | | **Among males** | | |
|---|---|---|---|---|---|---|
| | **Has menarche data (n=2112)** | **Missing menarche data (n=160)** | *P-value for difference* | **Has voice breaking data (n=1499)** | **Missing voice breaking data (n=314)** | *P-value for difference* |
| Mother has no further academic education – n (%) | 1126 (53.31) | 119 (74.38) | 5.54E-07 | 717 (47.83) | 188 (59.87) | 1.14E-04 |
| **After puberty onset (age 18y assessment)** | | | | | | |
| Body mass index (kg/m$^2$) – mean (SD) | 22.91 (4.23) | 22.99 (4.80) | 0.850 | 22.53 (3.82) | 22.63 (3.93) | 0.676 |
| Fat mass index (kg/m$^2$) – mean (SD) | 7.90 (3.38) | 7.78 (3.78) | 0.688 | 4.29 (3.05) | 4.57 (3.33) | 0.157 |
| Systolic blood pressure (mmHg) – mean (SD) | 109.96 (7.83) | 110.69 (9.75) | 0.384 | 120.07 (9.02) | 119.56 (9.08) | 0.376 |
| Diastolic blood pressure (mmHg) – mean (SD) | 64.98 (6.05) | 65.95 (6.63) | 0.087 | 63.32 (6.43) | 63.80 (6.51) | 0.244 |
| Triglycerides (mmol/l) – mean (SD) | 0.92 (0.31) | 0.98 (0.32) | 0.086 | 0.91 (0.35) | 0.89 (0.29) | 0.610 |
| HDL cholesterol (mmol/l) – mean (SD) | 1.49 (0.23) | 1.47 (0.23) | 0.383 | 1.30 (0.18) | 1.30 (0.19) | 0.622 |
| LDL cholesterol (mmol/l) – mean (SD) | 1.15 (0.36) | 1.09 (0.35) | 0.121 | 0.92 (0.31) | 0.95 (0.36) | 0.226 |
| Total cholesterol (mmol/l) – mean (SD) | 3.77 (0.67) | 3.67 (0.66) | 0.170 | 3.27 (0.57) | 3.32 (0.66) | 0.218 |
| Glucose (mmol/l) – mean (SD) | 4.05 (0.37) | 4.18 (0.98) | 0.220 | 4.24 (0.60) | 4.27 (0.56) | 0.481 |
| Glycoprotein acetyls (mmol/l) – mean (SD) | 1.25 (0.13) | 1.27 (0.15) | 0.087 | 1.18 (0.13) | 1.19 (0.12) | 0.842 |
| **Before puberty onset (age 8y assessment)** | | | | | | |
| Body mass index (kg/m$^2$) – mean (SD) | 16.27 (2.08) | 15.82 (1.88) | 0.022 | 16.01 (1.79) | 15.79 (1.53) | 0.071 |
| Fat mass index (kg/m$^2$) – mean (SD) | 4.86 (2.26) | 4.30 (2.14) | 0.019 | 3.63 (2.17) | 3.49 (2.16) | 0.371 |
| Systolic blood pressure (mmHg) – mean (SD) | 98.82 (9.21) | 98.67 (8.93) | 0.870 | 98.29 (8.99) | 97.39 (8.73) | 0.149 |
| Diastolic blood pressure (mmHg) – mean (SD) | 56.87 (6.51) | 58.08 (6.72) | 0.083 | 55.68 (6.79) | 55.80 (6.00) | 0.800 |
| Triglycerides (mmol/l) – mean (SD) | 1.08 (0.37) | 1.02 (0.36) | 0.209 | 1.02 (0.39) | 1.00 (0.31) | 0.398 |
| HDL cholesterol (mmol/l) – mean (SD) | 1.48 (0.20) | 1.51 (0.22) | 0.410 | 1.50 (0.21) | 1.51 (0.20) | 0.581 |
| LDL cholesterol (mmol/l) – mean (SD) | 1.27 (0.33) | 1.27 (0.37) | 0.997 | 1.16 (0.33) | 1.20 90.34) | 0.170 |
| Total cholesterol (mmol/l) – mean (SD) | 4.01 (0.60) | 3.99 (0.65) | 0.820 | 3.83 (0.59) | 3.90 (0.63) | 0.126 |
| Glucose (mmol/l) – mean (SD) | 4.13 (0.50) | 4.16 (0.46) | 0.633 | 4.24 (0.52) | 4.20 (0.51) | 0.345 |
| Glycoprotein acetyls (mmol/l) – mean (SD) | 1.25 (0.14) | 1.23 (0.13) | 0.230 | 1.22 (0.13) | 1.21 (0.15) | 0.655 |

Sample sizes vary as participants described are those with data on covariates, at least one adiposity trait, and at least one metabolic trait
'No further academic education' defined as Certificate of Secondary Education, vocational, or O-level versus A-level or degree.
