# Supplementary material for: Influence of puberty timing on adiposity and cardiometabolic traits: A Mendelian randomisation study
Source: PLoS Med. 2018 Aug 28;15(8):e1002641. doi: 10.1371/journal.pmed.1002641 (PMC6112630; doi:10.1371/journal.pmed.1002641)
Supplement: S3 Table — (PDF) [file pmed.1002641.s022.pdf]

S3 Table Observational associations of age at menarche (per year later) with adiposity and cardiometabolic traits at age 18y among females in ALSPAC

	Adj. for age, education					*Adj. for age, education, BMI at age 8y*					*Adj. for age, education, outcome value at age 8y*				
**Standardised outcome at age 18y**	**N**	**Beta**	**LCL**	**UCL**	**P-value**	**N**	**Beta**	**LCL**	**UCL**	**P-value**	**N**	**Beta**	**LCL**	**UCL**	**P-value**
Body mass index (kg/m^2)	2230	-0.20	-0.24	-0.17	5.91E-29	1976	-0.05	-0.07	-0.02	0.002	1976	-0.05	-0.07	-0.02	0.002
Fat mass index (kg/m^2)	2152	-0.18	-0.21	-0.14	1.65E-26	1917	-0.04	-0.07	-0.01	0.003	1861	-0.03	-0.05	0.00	0.066
Lean mass index (kg/m^2)	2152	-0.07	-0.09	-0.05	2.29E-10	1917	-0.01	-0.03	0.01	0.398	1861	0.02	0.00	0.04	0.028
Systolic blood pressure (mmHg)	2087	-0.06	-0.09	-0.03	4.06E-05	1859	-0.02	-0.05	0.01	0.158	1834	-0.03	-0.06	0.00	0.069
Diastolic blood pressure (mmHg)	2087	-0.10	-0.13	-0.06	1.00E-07	1859	-0.06	-0.10	-0.02	1.13E-03	1834	-0.08	-0.11	-0.04	1.36E-05
Concentration of chylomicrons and extremely large VLDL particles (mol/l)	1315	-0.01	-0.05	0.03	0.601	1163	0.01	-0.04	0.05	0.711	874	0.00	-0.04	0.05	0.879
Total lipids in chylomicrons and extremely large VLDL (mmol/l)	1315	-0.01	-0.05	0.03	0.589	1163	0.01	-0.04	0.05	0.763	874	0.00	-0.04	0.05	0.886
Phospholipids in chylomicrons and extremely large VLDL (mmol/l)	1315	-0.01	-0.05	0.03	0.612	1163	0.01	-0.04	0.05	0.798	874	0.00	-0.05	0.05	0.943
Total cholesterol in chylomicrons and extremely large VLDL (mmol/l)	1315	-0.01	-0.05	0.04	0.759	1163	0.01	-0.04	0.06	0.686	874	0.01	-0.04	0.06	0.658
Cholesterol esters in chylomicrons and extremely large VLDL (mmol/l)	1315	0.00	-0.05	0.04	0.899	1163	0.01	-0.03	0.06	0.602	874	0.02	-0.03	0.07	0.471
Free cholesterol in chylomicrons and extremely large VLDL (mmol/l)	1315	-0.01	-0.05	0.03	0.598	1163	0.01	-0.04	0.05	0.810	874	0.00	-0.05	0.05	0.933
Triglycerides in chylomicrons and extremely large VLDL (mmol/l)	1315	-0.01	-0.05	0.03	0.547	1163	0.01	-0.04	0.05	0.779	874	0.00	-0.05	0.05	0.932
Concentration of very large VLDL particles (mol/l)	1315	-0.01	-0.05	0.03	0.524	1163	0.00	-0.04	0.05	0.855	874	0.00	-0.04	0.05	0.910
Total lipids in very large VLDL (mmol/l)	1315	-0.01	-0.05	0.03	0.568	1163	0.00	-0.04	0.05	0.862	874	0.00	-0.04	0.05	0.893
Phospholipids in very large VLDL (mmol/l)	1315	-0.01	-0.05	0.03	0.628	1163	0.00	-0.04	0.05	0.851	874	0.00	-0.04	0.05	0.920
Total cholesterol in very large VLDL (mmol/l)	1315	-0.01	-0.05	0.03	0.687	1163	0.01	-0.04	0.05	0.698	874	0.01	-0.04	0.06	0.726
Cholesterol esters in very large VLDL (mmol/l)	1315	-0.01	-0.05	0.03	0.681	1163	0.01	-0.04	0.06	0.660	874	0.01	-0.04	0.06	0.629
Free cholesterol in very large VLDL (mmol/l)	1315	-0.01	-0.05	0.03	0.696	1163	0.01	-0.04	0.05	0.745	874	0.01	-0.04	0.05	0.834
Triglycerides in very large VLDL (mmol/l)	1315	-0.01	-0.05	0.03	0.520	1163	0.00	-0.04	0.05	0.921	874	0.00	-0.04	0.05	0.937
Concentration of large VLDL particles (mol/l)	1315	-0.01	-0.05	0.03	0.600	1163	0.00	-0.05	0.05	0.956	874	0.00	-0.04	0.05	0.908
Total lipids in large VLDL (mmol/l)	1315	-0.01	-0.05	0.03	0.635	1163	0.00	-0.05	0.05	0.919	874	0.00	-0.04	0.05	0.871
Phospholipids in large VLDL (mmol/l)	1315	-0.01	-0.05	0.03	0.685	1163	0.00	-0.04	0.05	0.909	874	0.00	-0.04	0.05	0.888
Total cholesterol in large VLDL (mmol/l)	1315	-0.01	-0.05	0.04	0.789	1163	0.01	-0.04	0.05	0.798	874	0.01	-0.04	0.06	0.756
Cholesterol esters in large VLDL (mmol/l)	1315	0.00	-0.05	0.04	0.868	1163	0.01	-0.04	0.06	0.717	874	0.01	-0.04	0.06	0.636
Free cholesterol in large VLDL (mmol/l)	1315	-0.01	-0.05	0.03	0.713	1163	0.00	-0.04	0.05	0.885	874	0.00	-0.04	0.05	0.873
Triglycerides in large VLDL (mmol/l)	1315	-0.01	-0.05	0.03	0.563	1163	0.00	-0.05	0.05	0.973	874	0.00	-0.04	0.05	0.913
Concentration of medium VLDL particles (mol/l)	1315	-0.01	-0.05	0.04	0.771	1163	0.01	-0.04	0.05	0.826	874	0.01	-0.04	0.06	0.776
Total lipids in medium VLDL (mmol/l)	1315	0.00	-0.05	0.04	0.829	1163	0.01	-0.04	0.06	0.785	874	0.01	-0.04	0.06	0.721
Phospholipids in medium VLDL (mmol/l)	1315	0.00	-0.04	0.04	0.993	1163	0.01	-0.04	0.06	0.732	874	0.01	-0.04	0.06	0.703
Total cholesterol in medium VLDL (mmol/l)	1315	0.01	-0.04	0.05	0.784	1163	0.01	-0.04	0.06	0.582	874	0.02	-0.03	0.07	0.514
Cholesterol esters in medium VLDL (mmol/l)	1315	0.01	-0.04	0.06	0.645	1163	0.02	-0.03	0.07	0.480	874	0.02	-0.03	0.07	0.402
Free cholesterol in medium VLDL (mmol/l)	1315	0.00	-0.04	0.05	0.977	1163	0.01	-0.04	0.06	0.741	874	0.01	-0.04	0.06	0.727
Triglycerides in medium VLDL (mmol/l)	1315	-0.01	-0.06	0.03	0.588	1163	0.00	-0.05	0.05	0.923	874	0.00	-0.04	0.05	0.855
Concentration of small VLDL particles (mol/l)	1315	0.01	-0.04	0.06	0.653	1163	0.01	-0.04	0.06	0.713	874	0.01	-0.05	0.06	0.829
Total lipids in small VLDL (mmol/l)	1315	0.01	-0.03	0.06	0.564	1163	0.01	-0.04	0.07	0.638	874	0.01	-0.05	0.06	0.810
Phospholipids in small VLDL (mmol/l)	1315	0.02	-0.04	0.07	0.552	1163	0.01	-0.05	0.06	0.839	874	0.00	-0.05	0.05	0.998
Total cholesterol in small VLDL (mmol/l)	1315	0.02	-0.03	0.07	0.375	1163	0.02	-0.03	0.08	0.422	874	0.01	-0.04	0.06	0.639
Cholesterol esters in small VLDL (mmol/l)	1315	0.02	-0.03	0.07	0.392	1163	0.03	-0.03	0.08	0.353	874	0.02	-0.04	0.07	0.550
Free cholesterol in small VLDL (mmol/l)	1315	0.02	-0.03	0.07	0.395	1163	0.01	-0.04	0.07	0.639	874	0.00	-0.05	0.06	0.861
Triglycerides in small VLDL (mmol/l)	1315	0.00	-0.04	0.05	0.837	1163	0.01	-0.05	0.06	0.818	874	0.00	-0.05	0.05	0.920
Concentration of very small VLDL particles (mol/l)	1315	0.04	-0.01	0.09	0.122	1163	0.03	-0.03	0.08	0.332	874	0.00	-0.05	0.06	0.849
Total lipids in very small VLDL (mmol/l)	1315	0.04	-0.01	0.09	0.166	1163	0.03	-0.02	0.09	0.278	874	0.01	-0.04	0.06	0.724
Phospholipids in very small VLDL (mmol/l)	1315	0.04	-0.01	0.09	0.080	1163	0.03	-0.02	0.08	0.271	874	0.00	-0.05	0.05	0.958
Total cholesterol in very small VLDL (mmol/l)	1315	0.03	-0.02	0.07	0.305	1163	0.03	-0.02	0.09	0.245	874	0.02	-0.04	0.07	0.534
Cholesterol esters in very small VLDL (mmol/l)	1315	0.02	-0.03	0.07	0.498	1163	0.03	-0.03	0.08	0.289	874	0.02	-0.04	0.07	0.536
Free cholesterol in very small VLDL (mmol/l)	1315	0.04	-0.01	0.09	0.080	1163	0.04	-0.02	0.09	0.202	874	0.02	-0.03	0.07	0.467
Triglycerides in very small VLDL (mmol/l)	1315	0.03	-0.02	0.08	0.313	1163	0.01	-0.05	0.07	0.750	874	0.00	-0.06	0.05	0.932
Concentration of IDL particles (mol/l)	1315	0.05	0.00	0.09	0.064	1163	0.03	-0.02	0.09	0.230	874	0.00	-0.05	0.05	0.983
Total lipids in IDL (mmol/l)	1315	0.04	0.00	0.09	0.072	1163	0.03	-0.02	0.09	0.232	874	0.00	-0.05	0.05	0.996
Phospholipids in IDL (mmol/l)	1315	0.04	0.00	0.09	0.077	1163	0.03	-0.02	0.08	0.285	874	0.00	-0.05	0.05	0.896

S3 Table Observational associations of age at menarche (per year later) with adiposity and cardiometabolic traits at age 18y among females in ALSPAC

	Adj. for age, education					*Adj. for age, education, BMI at age 8y*					*Adj. for age, education, outcome value at age 8y*				
**Standardised outcome at age 18y**	**N**	**Beta**	**LCL**	**UCL**	**P-value**	**N**	**Beta**	**LCL**	**UCL**	**P-value**	**N**	**Beta**	**LCL**	**UCL**	**P-value**
Total cholesterol in IDL (mmol/l)	1315	0.04	-0.01	0.09	0.082	1163	0.04	-0.02	0.09	0.192	874	0.01	-0.04	0.05	0.827
Cholesterol esters in IDL (mmol/l)	1315	0.04	-0.01	0.09	0.096	1163	0.04	-0.02	0.09	0.182	874	0.01	-0.04	0.06	0.682
Free cholesterol in IDL (mmol/l)	1315	0.05	0.00	0.10	0.060	1163	0.03	-0.02	0.09	0.230	874	0.00	-0.05	0.05	0.913
Triglycerides in IDL (mmol/l)	1315	0.04	-0.01	0.09	0.142	1163	0.01	-0.04	0.07	0.701	874	0.00	-0.06	0.05	0.895
Concentration of large LDL particles (mol/l)	1315	0.04	-0.01	0.09	0.104	1163	0.02	-0.03	0.08	0.381	874	-0.01	-0.06	0.05	0.820
Total lipids in large LDL (mmol/l)	1315	0.04	-0.01	0.09	0.098	1163	0.03	-0.03	0.08	0.339	874	-0.01	-0.06	0.04	0.777
Phospholipids in large LDL (mmol/l)	1315	0.04	-0.01	0.09	0.112	1163	0.03	-0.03	0.08	0.355	874	-0.01	-0.06	0.04	0.807
Total cholesterol in large LDL (mmol/l)	1315	0.04	-0.01	0.09	0.099	1163	0.03	-0.03	0.08	0.304	874	-0.01	-0.06	0.04	0.787
Cholesterol esters in large LDL (mmol/l)	1315	0.04	-0.01	0.09	0.107	1163	0.03	-0.03	0.08	0.310	874	-0.01	-0.06	0.04	0.794
Free cholesterol in large LDL (mmol/l)	1315	0.04	0.00	0.09	0.078	1163	0.03	-0.02	0.08	0.290	874	-0.01	-0.06	0.04	0.784
Triglycerides in large LDL (mmol/l)	1315	0.03	-0.02	0.08	0.193	1163	0.01	-0.05	0.06	0.821	874	0.00	-0.06	0.05	0.925
Concentration of medium LDL particles (mol/l)	1315	0.04	-0.01	0.08	0.140	1163	0.02	-0.03	0.07	0.480	874	-0.01	-0.06	0.05	0.793
Total lipids in medium LDL (mmol/l)	1315	0.04	-0.01	0.09	0.118	1163	0.02	-0.03	0.08	0.402	874	-0.01	-0.06	0.04	0.721
Phospholipids in medium LDL (mmol/l)	1315	0.04	-0.01	0.08	0.146	1163	0.02	-0.03	0.07	0.444	874	-0.01	-0.06	0.04	0.739
Total cholesterol in medium LDL (mmol/l)	1315	0.04	-0.01	0.09	0.114	1163	0.03	-0.03	0.08	0.353	874	-0.01	-0.06	0.04	0.738
Cholesterol esters in medium LDL (mmol/l)	1315	0.04	-0.01	0.09	0.121	1163	0.02	-0.03	0.08	0.363	874	-0.01	-0.06	0.04	0.752
Free cholesterol in medium LDL (mmol/l)	1315	0.04	-0.01	0.09	0.091	1163	0.03	-0.03	0.08	0.318	874	-0.01	-0.06	0.04	0.723
Triglycerides in medium LDL (mmol/l)	1315	0.03	-0.02	0.07	0.252	1163	0.00	-0.05	0.05	0.992	874	0.00	-0.06	0.05	0.907
Concentration of small LDL particles (mol/l)	1315	0.04	-0.01	0.08	0.141	1163	0.02	-0.04	0.07	0.511	874	-0.01	-0.06	0.04	0.753
Total lipids in small LDL (mmol/l)	1315	0.04	-0.01	0.09	0.120	1163	0.02	-0.03	0.08	0.411	874	-0.01	-0.06	0.04	0.692
Phospholipids in small LDL (mmol/l)	1315	0.04	-0.01	0.09	0.115	1163	0.02	-0.03	0.08	0.423	874	-0.01	-0.06	0.04	0.748
Total cholesterol in small LDL (mmol/l)	1315	0.04	-0.01	0.09	0.119	1163	0.02	-0.03	0.08	0.370	874	-0.01	-0.06	0.04	0.679
Cholesterol esters in small LDL (mmol/l)	1315	0.04	-0.01	0.09	0.130	1163	0.02	-0.03	0.08	0.391	874	-0.01	-0.06	0.04	0.715
Free cholesterol in small LDL (mmol/l)	1315	0.04	-0.01	0.09	0.092	1163	0.03	-0.03	0.08	0.309	874	-0.01	-0.06	0.04	0.815
Triglycerides in small LDL (mmol/l)	1315	0.02	-0.02	0.07	0.347	1163	0.00	-0.05	0.05	0.992	874	0.00	-0.06	0.05	0.880
Concentration of very large HDL particles (mol/l)	1315	0.03	-0.02	0.08	0.250	1163	0.02	-0.04	0.08	0.512	874	0.00	-0.05	0.05	0.931
Total lipids in very large HDL (mmol/l)	1315	0.03	-0.02	0.08	0.243	1163	0.02	-0.04	0.08	0.454	874	0.00	-0.05	0.05	0.983
Phospholipids in very large HDL (mmol/l)	1315	0.03	-0.02	0.08	0.237	1163	0.02	-0.04	0.07	0.567	874	-0.01	-0.06	0.04	0.799
Total cholesterol in very large HDL (mmol/l)	1315	0.03	-0.02	0.08	0.312	1163	0.02	-0.03	0.08	0.412	874	0.01	-0.05	0.06	0.822
Cholesterol esters in very large HDL (mmol/l)	1315	0.02	-0.03	0.07	0.357	1163	0.02	-0.03	0.08	0.416	874	0.01	-0.05	0.06	0.807
Free cholesterol in very large HDL (mmol/l)	1315	0.03	-0.02	0.08	0.230	1163	0.02	-0.04	0.08	0.433	874	0.00	-0.05	0.06	0.859
Triglycerides in very large HDL (mmol/l)	1315	0.03	-0.01	0.08	0.156	1163	0.03	-0.02	0.09	0.177	874	0.04	-0.02	0.09	0.166
Concentration of large HDL particles (mol/l)	1315	0.03	-0.02	0.08	0.273	1163	0.00	-0.05	0.06	0.932	874	-0.01	-0.06	0.04	0.586
Total lipids in large HDL (mmol/l)	1315	0.03	-0.02	0.08	0.240	1163	0.00	-0.05	0.06	0.871	874	-0.01	-0.06	0.04	0.612
Phospholipids in large HDL (mmol/l)	1315	0.03	-0.02	0.08	0.274	1163	0.00	-0.05	0.06	0.973	874	-0.02	-0.07	0.03	0.477
Total cholesterol in large HDL (mmol/l)	1315	0.03	-0.02	0.08	0.229	1163	0.01	-0.05	0.06	0.804	874	-0.01	-0.06	0.04	0.746
Cholesterol esters in large HDL (mmol/l)	1315	0.03	-0.02	0.08	0.243	1163	0.01	-0.05	0.06	0.831	874	-0.01	-0.06	0.04	0.744
Free cholesterol in large HDL (mmol/l)	1315	0.03	-0.02	0.08	0.183	1163	0.01	-0.05	0.07	0.708	874	-0.01	-0.06	0.04	0.763
Triglycerides in large HDL (mmol/l)	1315	0.03	-0.01	0.08	0.144	1163	0.02	-0.04	0.07	0.526	874	0.02	-0.03	0.07	0.526
Concentration of medium HDL particles (mol/l)	1315	0.01	-0.04	0.05	0.799	1163	-0.03	-0.08	0.03	0.358	874	-0.01	-0.07	0.04	0.614
Total lipids in medium HDL (mmol/l)	1315	0.01	-0.04	0.06	0.718	1163	-0.02	-0.08	0.03	0.383	874	-0.02	-0.07	0.04	0.583
Phospholipids in medium HDL (mmol/l)	1315	0.01	-0.04	0.06	0.732	1163	-0.02	-0.08	0.03	0.386	874	-0.01	-0.07	0.04	0.621
Total cholesterol in medium HDL (mmol/l)	1315	0.01	-0.04	0.06	0.670	1163	-0.02	-0.07	0.03	0.442	874	-0.02	-0.07	0.04	0.566
Cholesterol esters in medium HDL (mmol/l)	1315	0.01	-0.04	0.05	0.724	1163	-0.02	-0.07	0.03	0.416	874	-0.02	-0.07	0.04	0.541
Free cholesterol in medium HDL (mmol/l)	1315	0.02	-0.03	0.06	0.459	1163	-0.01	-0.07	0.04	0.591	874	-0.01	-0.07	0.04	0.685
Triglycerides in medium HDL (mmol/l)	1315	-0.01	-0.06	0.04	0.798	1163	-0.02	-0.07	0.04	0.541	874	-0.01	-0.07	0.04	0.622
Concentration of small HDL particles (mol/l)	1315	-0.02	-0.07	0.03	0.556	1163	-0.04	-0.10	0.01	0.142	874	-0.03	-0.09	0.03	0.279
Total lipids in small HDL (mmol/l)	1315	0.00	-0.05	0.05	0.926	1163	-0.04	-0.09	0.02	0.202	874	-0.03	-0.09	0.03	0.381
Phospholipids in small HDL (mmol/l)	1315	-0.02	-0.07	0.03	0.400	1163	-0.05	-0.10	0.01	0.114	874	-0.03	-0.09	0.03	0.298
Total cholesterol in small HDL (mmol/l)	1315	0.02	-0.03	0.07	0.401	1163	-0.02	-0.07	0.03	0.496	874	-0.02	-0.07	0.04	0.540
Cholesterol esters in small HDL (mmol/l)	1315	0.02	-0.02	0.07	0.293	1163	-0.01	-0.06	0.04	0.669	874	-0.02	-0.07	0.04	0.549

S3 Table Observational associations of age at menarche (per year later) with adiposity and cardiometabolic traits at age 18y among females in ALSPAC

	Adj. for age, education					*Adj. for age, education, BMI at age 8y*					*Adj. for age, education, outcome value at age 8y*				
**Standardised outcome at age 18y**	**N**	**Beta**	**LCL**	**UCL**	**P-value**	**N**	**Beta**	**LCL**	**UCL**	**P-value**	**N**	**Beta**	**LCL**	**UCL**	**P-value**
Free cholesterol in small HDL (mmol/l)	1315	0.00	-0.05	0.05	0.959	1163	-0.04	-0.09	0.02	0.210	874	-0.02	-0.08	0.04	0.469
Triglycerides in small HDL (mmol/l)	1315	-0.01	-0.06	0.04	0.783	1163	-0.02	-0.07	0.04	0.573	874	-0.02	-0.07	0.04	0.554
Phospholipids to total lipds ratio in chylomicrons and extremely large VLDL (%)	1315	0.01	-0.02	0.04	0.457	1163	0.01	-0.02	0.04	0.616	874	0.01	-0.03	0.04	0.722
Total cholesterol to total lipids ratio in chylomicrons and extremely large VLDL (%)	1315	0.01	-0.03	0.06	0.607	1163	0.01	-0.04	0.06	0.665	874	0.01	-0.04	0.07	0.635
Cholesterol esters to total lipids ratio in chylomicrons and extremely large VLDL (%)	1315	0.01	-0.04	0.06	0.599	1163	0.01	-0.04	0.06	0.699	874	0.01	-0.05	0.07	0.641
Free cholesterol to total lipids ratio in chylomicrons and extremely large VLDL (%)	1315	0.00	-0.04	0.04	0.971	1163	0.01	-0.04	0.05	0.829	874	0.00	-0.05	0.04	0.850
Triglycerides to total lipids ratio in chylomicrons and extremely large VLDL (%)	1315	-0.01	-0.05	0.02	0.500	1163	-0.01	-0.05	0.03	0.574	874	-0.01	-0.06	0.03	0.599
Phospholipids to total lipds ratio in very large VLDL (%)	1315	0.02	-0.02	0.06	0.328	1163	0.02	-0.02	0.07	0.291	874	0.02	-0.03	0.07	0.436
Total cholesterol to total lipids ratio in very large VLDL (%)	1315	-0.01	-0.05	0.04	0.706	1163	0.00	-0.05	0.05	0.944	874	0.01	-0.05	0.06	0.860
Cholesterol esters to total lipids ratio in very large VLDL (%)	1315	-0.01	-0.04	0.02	0.575	1163	-0.01	-0.05	0.03	0.712	874	0.00	-0.05	0.04	0.870
Free cholesterol to total lipids ratio in very large VLDL (%)	1315	0.00	-0.04	0.04	0.953	1163	0.01	-0.03	0.05	0.677	874	0.02	-0.03	0.07	0.452
Triglycerides to total lipids ratio in very large VLDL (%)	1315	0.00	-0.05	0.05	0.894	1163	-0.01	-0.06	0.05	0.865	874	-0.01	-0.08	0.05	0.669
Phospholipids to total lipds ratio in large VLDL (%)	1315	0.02	-0.02	0.06	0.379	1163	0.02	-0.03	0.07	0.398	874	0.01	-0.04	0.07	0.589
Total cholesterol to total lipids ratio in large VLDL (%)	1315	0.04	-0.01	0.08	0.122	1163	0.05	-0.01	0.10	0.077	874	0.04	-0.01	0.10	0.151
Cholesterol esters to total lipids ratio in large VLDL (%)	1315	0.03	-0.02	0.09	0.196	1163	0.05	-0.01	0.11	0.117	874	0.05	-0.02	0.12	0.185
Free cholesterol to total lipids ratio in large VLDL (%)	1315	0.01	-0.01	0.03	0.389	1163	0.01	-0.01	0.03	0.429	874	0.00	-0.02	0.03	0.706
Triglycerides to total lipids ratio in large VLDL (%)	1315	-0.03	-0.08	0.01	0.117	1163	-0.04	-0.09	0.01	0.088	874	-0.04	-0.09	0.01	0.151
Phospholipids to total lipds ratio in medium VLDL (%)	1315	0.05	0.00	0.10	0.049	1163	0.03	-0.03	0.09	0.301	874	0.03	-0.03	0.09	0.354
Total cholesterol to total lipids ratio in medium VLDL (%)	1315	0.04	0.00	0.09	0.062	1163	0.04	-0.01	0.09	0.081	874	0.04	-0.01	0.09	0.113
Cholesterol esters to total lipids ratio in medium VLDL (%)	1315	0.04	-0.01	0.08	0.111	1163	0.04	-0.01	0.09	0.113	874	0.04	-0.01	0.09	0.153
Free cholesterol to total lipids ratio in medium VLDL (%)	1315	0.04	-0.01	0.08	0.091	1163	0.03	-0.02	0.08	0.223	874	0.02	-0.03	0.07	0.423
Triglycerides to total lipids ratio in medium VLDL (%)	1315	-0.05	-0.09	0.00	0.033	1163	-0.05	-0.10	0.00	0.070	874	-0.04	-0.09	0.01	0.104
Phospholipids to total lipds ratio in small VLDL (%)	1315	-0.01	-0.05	0.04	0.740	1163	-0.04	-0.09	0.02	0.176	874	-0.03	-0.08	0.02	0.299
Total cholesterol to total lipids ratio in small VLDL (%)	1315	0.02	-0.03	0.06	0.467	1163	0.02	-0.03	0.08	0.372	874	0.01	-0.04	0.07	0.664
Cholesterol esters to total lipids ratio in small VLDL (%)	1315	0.01	-0.04	0.06	0.641	1163	0.02	-0.03	0.08	0.397	874	0.01	-0.04	0.07	0.681
Free cholesterol to total lipids ratio in small VLDL (%)	1315	0.04	-0.01	0.09	0.080	1163	0.01	-0.05	0.06	0.736	874	0.01	-0.04	0.06	0.747
Triglycerides to total lipids ratio in small VLDL (%)	1315	-0.02	-0.06	0.03	0.521	1163	-0.01	-0.06	0.04	0.650	874	0.00	-0.06	0.05	0.879
Phospholipids to total lipds ratio in very small VLDL (%)	1315	0.04	0.00	0.09	0.056	1163	0.02	-0.03	0.07	0.345	874	0.00	-0.04	0.05	0.839
Total cholesterol to total lipids ratio in very small VLDL (%)	1315	-0.02	-0.07	0.02	0.344	1163	0.00	-0.05	0.05	0.975	874	-0.01	-0.07	0.04	0.628
Cholesterol esters to total lipids ratio in very small VLDL (%)	1315	-0.03	-0.08	0.02	0.181	1163	0.00	-0.06	0.05	0.863	874	-0.02	-0.08	0.04	0.588
Free cholesterol to total lipids ratio in very small VLDL (%)	1315	0.03	-0.01	0.07	0.160	1163	0.02	-0.03	0.06	0.423	874	0.03	-0.02	0.08	0.237
Triglycerides to total lipids ratio in very small VLDL (%)	1315	0.00	-0.05	0.05	0.932	1163	-0.02	-0.07	0.04	0.534	874	0.00	-0.06	0.05	0.909
Phospholipids to total lipds ratio in IDL (%)	1315	-0.02	-0.06	0.03	0.458	1163	-0.03	-0.08	0.01	0.172	874	-0.03	-0.08	0.03	0.329
Total cholesterol to total lipids ratio in IDL (%)	1315	0.01	-0.04	0.06	0.718	1163	0.03	-0.02	0.09	0.278	874	0.02	-0.04	0.07	0.598
Cholesterol esters to total lipids ratio in IDL (%)	1315	0.00	-0.05	0.04	0.924	1163	0.02	-0.03	0.08	0.354	874	0.02	-0.04	0.07	0.575
Free cholesterol to total lipids ratio in IDL (%)	1315	0.03	-0.02	0.07	0.214	1163	0.02	-0.03	0.06	0.517	874	-0.01	-0.05	0.04	0.779
Triglycerides to total lipids ratio in IDL (%)	1315	0.00	-0.05	0.05	0.890	1163	-0.02	-0.08	0.03	0.450	874	0.00	-0.06	0.05	0.884
Phospholipids to total lipds ratio in large LDL (%)	1315	-0.03	-0.07	0.01	0.098	1163	-0.03	-0.07	0.01	0.171	874	0.00	-0.04	0.04	0.938
Total cholesterol to total lipids ratio in large LDL (%)	1315	0.03	-0.02	0.07	0.234	1163	0.03	-0.02	0.08	0.183	874	0.00	-0.04	0.04	0.977
Cholesterol esters to total lipids ratio in large LDL (%)	1315	0.03	-0.01	0.07	0.214	1163	0.03	-0.01	0.08	0.162	874	0.00	-0.04	0.04	0.989
Free cholesterol to total lipids ratio in large LDL (%)	1315	-0.01	-0.05	0.03	0.599	1163	-0.01	-0.06	0.03	0.569	874	-0.01	-0.06	0.03	0.560
Triglycerides to total lipids ratio in large LDL (%)	1315	-0.01	-0.05	0.04	0.818	1163	-0.02	-0.08	0.03	0.469	874	0.00	-0.05	0.05	0.999
Phospholipids to total lipds ratio in medium LDL (%)	1315	-0.03	-0.07	0.01	0.104	1163	-0.03	-0.07	0.01	0.160	874	0.00	-0.04	0.04	0.866
Total cholesterol to total lipids ratio in medium LDL (%)	1315	0.03	-0.01	0.07	0.172	1163	0.03	-0.01	0.08	0.147	874	0.00	-0.04	0.04	0.932
Cholesterol esters to total lipids ratio in medium LDL (%)	1315	0.03	-0.01	0.07	0.141	1163	0.03	-0.01	0.08	0.142	874	0.00	-0.04	0.04	0.924
Free cholesterol to total lipids ratio in medium LDL (%)	1315	-0.03	-0.06	0.01	0.172	1163	-0.03	-0.07	0.02	0.223	874	-0.01	-0.05	0.03	0.712
Triglycerides to total lipids ratio in medium LDL (%)	1315	0.00	-0.04	0.04	0.968	1163	-0.01	-0.06	0.03	0.567	874	0.00	-0.05	0.05	0.874
Phospholipids to total lipds ratio in small LDL (%)	1315	-0.03	-0.06	0.01	0.177	1163	-0.03	-0.07	0.02	0.220	874	0.00	-0.04	0.04	0.865
Total cholesterol to total lipids ratio in small LDL (%)	1315	0.03	-0.01	0.07	0.214	1163	0.03	-0.02	0.08	0.214	874	0.00	-0.05	0.04	0.842
Cholesterol esters to total lipids ratio in small LDL (%)	1315	0.03	-0.01	0.07	0.176	1163	0.03	-0.02	0.07	0.202	874	0.00	-0.04	0.04	0.906
Free cholesterol to total lipids ratio in small LDL (%)	1315	-0.02	-0.06	0.01	0.210	1163	-0.02	-0.06	0.02	0.297	874	-0.01	-0.05	0.03	0.669

S3 Table Observational associations of age at menarche (per year later) with adiposity and cardiometabolic traits at age 18y among females in ALSPAC

	Adj. for age, education					*Adj. for age, education, BMI at age 8y*					*Adj. for age, education, outcome value at age 8y*				
**Standardised outcome at age 18y**	**N**	**Beta**	**LCL**	**UCL**	**P-value**	**N**	**Beta**	**LCL**	**UCL**	**P-value**	**N**	**Beta**	**LCL**	**UCL**	**P-value**
Triglycerides to total lipids ratio in small LDL (%)	1315	0.00	-0.05	0.04	0.900	1163	-0.01	-0.07	0.04	0.646	874	0.00	-0.05	0.05	0.999
Phospholipids to total lipds ratio in very large HDL (%)	1315	0.02	-0.02	0.06	0.400	1163	0.00	-0.05	0.04	0.877	874	-0.01	-0.05	0.03	0.689
Total cholesterol to total lipids ratio in very large HDL (%)	1315	-0.02	-0.06	0.02	0.363	1163	0.00	-0.05	0.05	0.970	874	0.00	-0.04	0.05	0.893
Cholesterol esters to total lipids ratio in very large HDL (%)	1315	-0.02	-0.06	0.02	0.361	1163	0.00	-0.05	0.05	0.987	874	0.00	-0.04	0.05	0.949
Free cholesterol to total lipids ratio in very large HDL (%)	1315	0.01	-0.04	0.06	0.657	1163	0.01	-0.04	0.07	0.598	874	0.02	-0.04	0.07	0.490
Triglycerides to total lipids ratio in very large HDL (%)	1315	0.00	-0.04	0.05	0.889	1163	0.01	-0.03	0.06	0.555	874	0.02	-0.03	0.07	0.387
Phospholipids to total lipds ratio in large HDL (%)	1315	-0.03	-0.07	0.02	0.252	1163	-0.02	-0.07	0.03	0.414	874	-0.04	-0.09	0.02	0.170
Total cholesterol to total lipids ratio in large HDL (%)	1315	0.02	-0.02	0.06	0.363	1163	0.01	-0.04	0.06	0.661	874	0.02	-0.03	0.07	0.468
Cholesterol esters to total lipids ratio in large HDL (%)	1315	0.02	-0.03	0.06	0.485	1163	0.01	-0.04	0.06	0.816	874	0.02	-0.03	0.07	0.489
Free cholesterol to total lipids ratio in large HDL (%)	1315	0.03	-0.01	0.08	0.136	1163	0.03	-0.02	0.08	0.269	874	0.02	-0.03	0.07	0.441
Triglycerides to total lipids ratio in large HDL (%)	1315	0.00	-0.04	0.04	0.844	1163	0.01	-0.03	0.06	0.604	874	0.02	-0.03	0.07	0.392
Phospholipids to total lipds ratio in medium HDL (%)	1315	0.01	-0.04	0.05	0.753	1163	0.00	-0.05	0.05	0.897	874	0.00	-0.05	0.05	0.932
Total cholesterol to total lipids ratio in medium HDL (%)	1315	0.00	-0.05	0.04	0.888	1163	0.00	-0.05	0.05	0.956	874	0.00	-0.05	0.05	0.987
Cholesterol esters to total lipids ratio in medium HDL (%)	1315	-0.01	-0.05	0.04	0.700	1163	0.00	-0.05	0.05	0.907	874	0.00	-0.05	0.05	0.950
Free cholesterol to total lipids ratio in medium HDL (%)	1315	0.03	-0.01	0.07	0.185	1163	0.02	-0.02	0.07	0.302	874	0.01	-0.04	0.05	0.728
Triglycerides to total lipids ratio in medium HDL (%)	1315	0.00	-0.05	0.04	0.838	1163	0.00	-0.05	0.05	0.912	874	0.01	-0.05	0.06	0.848
Phospholipids to total lipds ratio in small HDL (%)	1315	-0.04	-0.08	0.00	0.055	1163	-0.02	-0.07	0.03	0.403	874	0.00	-0.05	0.04	0.950
Total cholesterol to total lipids ratio in small HDL (%)	1315	0.04	0.00	0.08	0.052	1163	0.02	-0.03	0.06	0.458	874	0.00	-0.04	0.05	0.899
Cholesterol esters to total lipids ratio in small HDL (%)	1315	0.04	0.00	0.08	0.072	1163	0.02	-0.03	0.06	0.447	874	0.00	-0.04	0.04	0.998
Free cholesterol to total lipids ratio in small HDL (%)	1315	0.00	-0.04	0.05	0.945	1163	-0.01	-0.06	0.04	0.698	874	0.02	-0.03	0.07	0.533
Triglycerides to total lipids ratio in small HDL (%)	1315	0.00	-0.05	0.04	0.861	1163	0.01	-0.04	0.06	0.798	874	0.00	-0.05	0.05	0.903
Mean diameter for VLDL particles (nm)	1315	-0.02	-0.06	0.03	0.471	1163	0.00	-0.05	0.05	0.965	874	0.00	-0.05	0.05	0.961
Mean diameter for LDL particles (nm)	1315	0.00	-0.04	0.04	0.989	1163	0.01	-0.04	0.06	0.605	874	0.02	-0.03	0.07	0.442
Mean diameter for HDL particles (nm)	1315	0.03	-0.02	0.08	0.180	1163	0.02	-0.03	0.08	0.435	874	0.01	-0.04	0.05	0.802
Serum total cholesterol (mmol/l)	1315	0.04	0.00	0.09	0.079	1163	0.03	-0.03	0.08	0.320	874	-0.01	-0.05	0.04	0.818
Total cholesterol in VLDL (mmol/l)	1315	0.01	-0.03	0.06	0.560	1163	0.02	-0.03	0.07	0.412	874	0.02	-0.03	0.07	0.435
Remnant cholesterol (non-HDL, non-LDL -cholesterol) (mmol/l)	1315	0.03	-0.02	0.08	0.228	1163	0.03	-0.02	0.08	0.256	874	0.02	-0.03	0.07	0.496
Total cholesterol in LDL (mmol/l)	1315	0.04	-0.01	0.09	0.106	1163	0.03	-0.03	0.08	0.330	874	-0.01	-0.06	0.04	0.746
Total cholesterol in HDL (mmol/l)	1315	0.03	-0.02	0.08	0.254	1163	0.00	-0.06	0.06	0.972	874	-0.02	-0.07	0.03	0.514
Total cholesterol in HDL2 (mmol/l)	1315	0.03	-0.02	0.07	0.309	1163	0.00	-0.06	0.05	0.943	874	-0.02	-0.07	0.03	0.482
Total cholesterol in HDL3 (mmol/l)	1315	0.03	-0.02	0.08	0.192	1163	0.01	-0.05	0.06	0.823	874	-0.01	-0.06	0.04	0.657
Esterified cholesterol (mmol/l)	1309	0.05	0.00	0.09	0.062	1159	0.03	-0.02	0.08	0.267	871	0.00	-0.05	0.05	0.972
Free cholesterol (mmol/l)	1308	0.03	-0.02	0.08	0.258	1158	0.01	-0.04	0.06	0.703	871	-0.01	-0.06	0.04	0.619
Serum total triglycerides (mmol/l)	1315	0.00	-0.04	0.05	0.936	1163	0.00	-0.05	0.05	0.877	874	0.00	-0.05	0.05	0.972
Triglycerides in VLDL (mmol/l)	1315	-0.01	-0.05	0.04	0.771	1163	0.00	-0.05	0.05	0.890	874	0.00	-0.05	0.05	0.898
Triglycerides in LDL (mmol/l)	1315	0.03	-0.02	0.08	0.229	1163	0.00	-0.05	0.06	0.901	874	0.00	-0.06	0.05	0.926
Triglycerides in HDL (mmol/l)	1315	0.01	-0.04	0.06	0.741	1163	0.00	-0.06	0.05	0.929	874	0.00	-0.05	0.05	0.968
Diacylglycerol (mmol/l)	1272	-0.01	-0.05	0.04	0.833	1125	-0.01	-0.06	0.05	0.804	829	-0.01	-0.07	0.05	0.807
Ratio of diacylglycerol to triglycerides	1273	-0.01	-0.06	0.04	0.664	1126	-0.01	-0.07	0.04	0.660	830	-0.01	-0.07	0.05	0.773
Total phosphoglycerides (mmol/l)	1308	0.03	-0.02	0.08	0.220	1158	0.01	-0.04	0.07	0.631	871	0.00	-0.06	0.05	0.963
Ratio of triglycerides to phosphoglycerides	1308	0.00	-0.04	0.05	0.963	1158	0.01	-0.04	0.06	0.614	871	0.01	-0.04	0.06	0.765
Phosphatidylcholine and other cholines (mmol/l)	1297	0.02	-0.02	0.07	0.343	1148	-0.01	-0.06	0.05	0.819	859	-0.02	-0.07	0.04	0.492
Total cholines (mmol/l)	1309	0.03	-0.02	0.08	0.247	1159	0.01	-0.05	0.06	0.830	871	-0.01	-0.06	0.04	0.659
Apolipoprotein A-I (g/l)	1315	0.04	-0.01	0.09	0.127	1163	0.01	-0.05	0.06	0.775	874	-0.01	-0.06	0.04	0.604
Apolipoprotein B (g/l)	1315	0.03	-0.02	0.08	0.241	1163	0.03	-0.03	0.08	0.320	874	0.01	-0.04	0.06	0.679
Ratio of apolipoprotein B to apolipoprotein A-I	1315	0.01	-0.04	0.06	0.638	1163	0.02	-0.03	0.08	0.355	874	0.02	-0.03	0.07	0.451
Total fatty acids (mmol/l)	1309	0.03	-0.02	0.08	0.195	1159	0.02	-0.03	0.07	0.489	871	0.00	-0.05	0.05	0.962
Estimated description of fatty acid chain length, not actual carbon number	1309	-0.01	-0.06	0.03	0.587	1159	-0.02	-0.07	0.03	0.430	871	-0.01	-0.06	0.04	0.729
Estimated degree of unsaturation	1310	0.01	-0.03	0.06	0.552	1160	0.00	-0.05	0.05	0.952	872	-0.01	-0.07	0.04	0.585
22:6, docosahexaenoic acid (mmol/l)	1309	0.03	-0.01	0.08	0.175	1159	0.01	-0.04	0.07	0.621	871	-0.01	-0.06	0.05	0.828
18:2, linoleic acid (mmol/l)	1308	0.05	0.00	0.09	0.057	1158	0.02	-0.03	0.07	0.425	870	-0.01	-0.06	0.05	0.845

S3 Table Observational associations of age at menarche (per year later) with adiposity and cardiometabolic traits at age 18y among females in ALSPAC

	Adj. for age, education					*Adj. for age, education, BMI at age 8y*					*Adj. for age, education, outcome value at age 8y*				
**Standardised outcome at age 18y**	**N**	**Beta**	**LCL**	**UCL**	**P-value**	**N**	**Beta**	**LCL**	**UCL**	**P-value**	**N**	**Beta**	**LCL**	**UCL**	**P-value**
Conjugated linoleic acid (mmol/l)	1309	0.01	-0.04	0.06	0.613	1159	0.02	-0.04	0.08	0.528	870	0.01	-0.05	0.08	0.692
Omega-3 fatty acids (mmol/l)	1309	0.03	-0.02	0.08	0.222	1159	0.03	-0.02	0.08	0.204	871	0.00	-0.05	0.05	0.990
Omega-6 fatty acids (mmol/l)	1309	0.04	-0.01	0.09	0.091	1159	0.02	-0.03	0.07	0.467	871	-0.01	-0.06	0.04	0.717
Polyunsaturated fatty acids (mmol/l)	1308	0.04	-0.01	0.09	0.108	1158	0.02	-0.03	0.07	0.464	870	-0.01	-0.06	0.04	0.634
Monounsaturated fatty acids; 16:1, 18:1 (mmol/l)	1309	0.02	-0.03	0.07	0.435	1159	0.01	-0.05	0.06	0.796	871	-0.01	-0.06	0.05	0.774
Saturated fatty acids (mmol/l)	1308	0.03	-0.02	0.07	0.241	1158	0.02	-0.03	0.07	0.404	870	0.01	-0.04	0.06	0.721
Ratio of 22:6 docosahexaenoic acid to total fatty acids (%)	1310	0.02	-0.03	0.06	0.430	1160	0.01	-0.04	0.05	0.817	872	0.00	-0.05	0.05	0.960
Ratio of 18:2 linoleic acid to total fatty acids (%)	1309	0.03	-0.02	0.07	0.234	1159	0.00	-0.05	0.05	0.859	871	0.00	-0.05	0.06	0.882
Ratio of conjugated linoleic acid to total fatty acids (%)	1310	0.00	-0.05	0.06	0.861	1160	0.01	-0.05	0.08	0.698	871	0.01	-0.06	0.09	0.737
Ratio of omega-3 fatty acids to total fatty acids (%)	1310	0.01	-0.04	0.06	0.667	1160	0.03	-0.02	0.08	0.281	872	0.00	-0.05	0.06	0.879
Ratio of omega-6 fatty acids to total fatty acids (%)	1310	0.01	-0.03	0.06	0.531	1160	-0.01	-0.05	0.04	0.842	872	-0.01	-0.06	0.05	0.851
Ratio of polyunsaturated fatty acids to total fatty acids (%)	1309	0.02	-0.03	0.06	0.471	1159	0.00	-0.05	0.05	0.913	871	0.00	-0.06	0.05	0.929
Ratio of monounsaturated fatty acids to total fatty acids (%)	1310	-0.01	-0.06	0.03	0.604	1160	-0.02	-0.06	0.03	0.543	872	-0.02	-0.07	0.03	0.484
Ratio of saturated fatty acids to total fatty acids (%)	1309	0.00	-0.05	0.04	0.873	1159	0.02	-0.03	0.06	0.457	871	0.03	-0.03	0.08	0.326
Glucose (mmol/l)	1314	0.00	-0.04	0.04	0.885	1162	0.02	-0.02	0.06	0.370	869	0.00	-0.06	0.05	0.865
Lactate (mmol/l)	1314	-0.02	-0.07	0.02	0.301	1162	-0.02	-0.07	0.03	0.481	873	0.00	-0.06	0.06	0.928
Pyruvate (mmol/l)	1314	-0.06	-0.11	-0.02	0.009	1162	-0.04	-0.09	0.01	0.117	871	-0.03	-0.09	0.02	0.247
Citrate (mmol/l)	1314	0.03	-0.01	0.08	0.187	1162	0.00	-0.05	0.05	0.991	871	0.02	-0.04	0.08	0.529
Alanine (mmol/l)	1314	0.01	-0.04	0.06	0.774	1162	0.01	-0.05	0.07	0.713	873	0.03	-0.03	0.10	0.325
Glutamine (mmol/l)	1314	0.06	0.02	0.11	0.008	1162	0.05	0.00	0.11	0.037	871	0.06	0.00	0.11	0.039
Histidine (mmol/l)	1314	0.00	-0.05	0.05	0.986	1162	-0.02	-0.08	0.04	0.517	872	0.00	-0.06	0.06	0.884
Isoleucine (mmol/l)	1314	-0.02	-0.07	0.02	0.375	1162	0.00	-0.05	0.05	0.931	873	0.01	-0.04	0.06	0.782
Leucine (mmol/l)	1314	0.00	-0.05	0.05	0.974	1162	0.02	-0.04	0.07	0.564	873	0.03	-0.02	0.07	0.303
Valine (mmol/l)	1314	-0.04	-0.08	0.01	0.106	1162	0.00	-0.05	0.04	0.856	873	-0.01	-0.06	0.04	0.667
Phenylalanine (mmol/l)	1313	-0.05	-0.10	-0.01	0.025	1161	-0.06	-0.11	0.00	0.048	871	-0.06	-0.12	-0.01	0.031
Tyrosine (mmol/l)	1314	-0.05	-0.10	0.01	0.075	1162	-0.02	-0.08	0.05	0.624	870	-0.03	-0.10	0.03	0.343
Acetate (mmol/l)	1313	-0.01	-0.05	0.04	0.834	1161	-0.02	-0.07	0.04	0.543	873	-0.02	-0.10	0.05	0.512
Acetoacetate (mmol/l)	1314	0.00	-0.04	0.04	0.885	1162	-0.03	-0.07	0.02	0.257	872	0.00	-0.05	0.05	0.971
3-hydroxybutyrate (mmol/l)	1312	-0.01	-0.05	0.04	0.752	1160	-0.04	-0.09	0.01	0.131	871	-0.01	-0.07	0.05	0.711
Creatinine (mmol/l)	1314	-0.03	-0.07	0.00	0.058	1162	-0.03	-0.07	0.01	0.114	871	-0.03	-0.07	0.01	0.111
Albumin (signal area)	1315	0.02	-0.03	0.07	0.398	1163	0.00	-0.05	0.05	0.941	872	0.03	-0.02	0.09	0.194
Glycoprotein acetyls, mainly a1-acid glycoprotein (mmol/l)	1314	-0.04	-0.09	0.01	0.098	1162	-0.03	-0.08	0.03	0.346	873	-0.02	-0.07	0.04	0.504

Complete case sample

	Adj. for age, education					*Adj. for age, education, BMI at age 8y*					*Adj. for age, education, outcome value at age 8y*				
**Standardised outcome at age 18y**	**N**	**Beta**	**LCL**	**UCL**	**P-value**	**N**	**Beta**	**LCL**	**UCL**	**P-value**	**N**	**Beta**	**LCL**	**UCL**	**P-value**
Body mass index (kg/m^2)	664	-0.20	-0.26	-0.14	1.25E-10	664	-0.06	-0.10	-0.01	0.027	664	-0.06	-0.10	-0.01	0.027
Fat mass index (kg/m^2)	664	-0.17	-0.23	-0.12	2.63E-09	664	-0.05	-0.10	-0.01	0.029	664	-0.03	-0.07	0.01	0.172
Lean mass index (kg/m^2)	664	-0.07	-0.11	-0.03	1.80E-04	664	-0.01	-0.05	0.03	0.536	664	0.03	0.00	0.06	0.067
Systolic blood pressure (mmHg)	664	-0.05	-0.11	0.00	0.049	664	-0.02	-0.08	0.03	0.430	664	-0.02	-0.07	0.03	0.504
Diastolic blood pressure (mmHg)	664	-0.08	-0.14	-0.02	0.008	664	-0.05	-0.11	0.01	0.127	664	-0.06	-0.12	0.00	0.042
Concentration of chylomicrons and extremely large VLDL particles (mol/l)	664	0.01	-0.05	0.06	0.852	664	0.04	-0.02	0.10	0.203	664	0.01	-0.05	0.06	0.770
Total lipids in chylomicrons and extremely large VLDL (mmol/l)	664	0.01	-0.05	0.06	0.820	664	0.04	-0.02	0.10	0.205	664	0.01	-0.04	0.06	0.747
Phospholipids in chylomicrons and extremely large VLDL (mmol/l)	664	0.01	-0.05	0.06	0.848	664	0.04	-0.02	0.10	0.225	664	0.01	-0.05	0.06	0.795
Total cholesterol in chylomicrons and extremely large VLDL (mmol/l)	664	0.01	-0.04	0.07	0.617	664	0.05	-0.02	0.11	0.149	664	0.02	-0.04	0.07	0.510
Cholesterol esters in chylomicrons and extremely large VLDL (mmol/l)	664	0.02	-0.04	0.08	0.486	664	0.05	-0.01	0.11	0.115	664	0.03	-0.03	0.08	0.356
Free cholesterol in chylomicrons and extremely large VLDL (mmol/l)	664	0.01	-0.05	0.06	0.816	664	0.04	-0.02	0.10	0.217	664	0.01	-0.05	0.06	0.747
Triglycerides in chylomicrons and extremely large VLDL (mmol/l)	664	0.00	-0.05	0.06	0.870	664	0.04	-0.02	0.10	0.220	664	0.01	-0.05	0.06	0.802
Concentration of very large VLDL particles (mol/l)	664	0.01	-0.05	0.07	0.715	664	0.04	-0.02	0.10	0.188	664	0.01	-0.04	0.07	0.631

S3 Table Observational associations of age at menarche (per year later) with adiposity and cardiometabolic traits at age 18y among females in ALSPAC

	Adj. for age, education					*Adj. for age, education, BMI at age 8y*					*Adj. for age, education, outcome value at age 8y*				
**Standardised outcome at age 18y**	**N**	**Beta**	**LCL**	**UCL**	**P-value**	**N**	**Beta**	**LCL**	**UCL**	**P-value**	**N**	**Beta**	**LCL**	**UCL**	**P-value**
Total lipids in very large VLDL (mmol/l)	664	0.01	-0.04	0.07	0.666	664	0.04	-0.02	0.10	0.179	664	0.01	-0.04	0.07	0.589
Phospholipids in very large VLDL (mmol/l)	664	0.01	-0.04	0.07	0.669	664	0.04	-0.02	0.10	0.177	664	0.01	-0.04	0.07	0.604
Total cholesterol in very large VLDL (mmol/l)	664	0.01	-0.05	0.07	0.688	664	0.04	-0.02	0.11	0.158	664	0.02	-0.04	0.07	0.572
Cholesterol esters in very large VLDL (mmol/l)	664	0.01	-0.04	0.07	0.661	664	0.05	-0.02	0.11	0.145	664	0.02	-0.04	0.07	0.512
Free cholesterol in very large VLDL (mmol/l)	664	0.01	-0.05	0.07	0.722	664	0.04	-0.02	0.10	0.177	664	0.01	-0.04	0.07	0.640
Triglycerides in very large VLDL (mmol/l)	664	0.01	-0.04	0.07	0.660	664	0.04	-0.02	0.10	0.191	664	0.01	-0.04	0.07	0.591
Concentration of large VLDL particles (mol/l)	664	0.02	-0.04	0.07	0.600	664	0.04	-0.02	0.10	0.200	664	0.02	-0.04	0.07	0.531
Total lipids in large VLDL (mmol/l)	664	0.02	-0.04	0.07	0.582	664	0.04	-0.02	0.11	0.186	664	0.02	-0.04	0.07	0.508
Phospholipids in large VLDL (mmol/l)	664	0.02	-0.04	0.07	0.576	664	0.04	-0.02	0.11	0.184	664	0.02	-0.04	0.07	0.512
Total cholesterol in large VLDL (mmol/l)	664	0.02	-0.04	0.08	0.539	664	0.05	-0.02	0.11	0.151	664	0.02	-0.03	0.08	0.444
Cholesterol esters in large VLDL (mmol/l)	664	0.02	-0.04	0.08	0.525	664	0.05	-0.02	0.11	0.137	664	0.02	-0.03	0.08	0.391
Free cholesterol in large VLDL (mmol/l)	664	0.02	-0.04	0.07	0.557	664	0.04	-0.02	0.11	0.169	664	0.02	-0.04	0.07	0.495
Triglycerides in large VLDL (mmol/l)	664	0.02	-0.04	0.07	0.605	664	0.04	-0.02	0.10	0.206	664	0.02	-0.04	0.07	0.535
Concentration of medium VLDL particles (mol/l)	664	0.02	-0.04	0.08	0.577	664	0.04	-0.02	0.11	0.186	664	0.02	-0.04	0.08	0.476
Total lipids in medium VLDL (mmol/l)	664	0.02	-0.04	0.08	0.548	664	0.05	-0.02	0.11	0.167	664	0.02	-0.03	0.08	0.437
Phospholipids in medium VLDL (mmol/l)	664	0.02	-0.04	0.08	0.501	664	0.05	-0.02	0.11	0.159	664	0.02	-0.03	0.08	0.420
Total cholesterol in medium VLDL (mmol/l)	664	0.03	-0.04	0.09	0.417	664	0.05	-0.01	0.12	0.109	664	0.03	-0.03	0.09	0.292
Cholesterol esters in medium VLDL (mmol/l)	664	0.03	-0.03	0.09	0.379	664	0.06	-0.01	0.13	0.086	664	0.04	-0.02	0.09	0.213
Free cholesterol in medium VLDL (mmol/l)	664	0.02	-0.04	0.08	0.491	664	0.05	-0.02	0.11	0.165	664	0.02	-0.03	0.08	0.427
Triglycerides in medium VLDL (mmol/l)	664	0.01	-0.05	0.07	0.654	664	0.04	-0.02	0.10	0.224	664	0.02	-0.04	0.07	0.542
Concentration of small VLDL particles (mol/l)	664	0.02	-0.05	0.08	0.628	664	0.04	-0.03	0.11	0.297	664	0.02	-0.05	0.08	0.617
Total lipids in small VLDL (mmol/l)	664	0.01	-0.05	0.08	0.685	664	0.04	-0.03	0.11	0.310	664	0.01	-0.05	0.07	0.682
Phospholipids in small VLDL (mmol/l)	664	0.01	-0.06	0.08	0.751	664	0.03	-0.05	0.10	0.468	664	0.01	-0.06	0.07	0.806
Total cholesterol in small VLDL (mmol/l)	664	0.01	-0.06	0.08	0.765	664	0.04	-0.03	0.11	0.296	664	0.01	-0.05	0.07	0.737
Cholesterol esters in small VLDL (mmol/l)	664	0.01	-0.06	0.07	0.858	664	0.04	-0.03	0.11	0.306	664	0.01	-0.05	0.07	0.765
Free cholesterol in small VLDL (mmol/l)	664	0.02	-0.05	0.08	0.617	664	0.04	-0.04	0.11	0.328	664	0.01	-0.05	0.08	0.685
Triglycerides in small VLDL (mmol/l)	664	0.02	-0.05	0.08	0.624	664	0.03	-0.03	0.10	0.321	664	0.02	-0.05	0.08	0.614
Concentration of very small VLDL particles (mol/l)	664	0.02	-0.05	0.09	0.547	664	0.04	-0.03	0.11	0.287	664	0.01	-0.05	0.07	0.748
Total lipids in very small VLDL (mmol/l)	664	0.01	-0.06	0.08	0.704	664	0.04	-0.03	0.11	0.300	664	0.01	-0.05	0.07	0.850
Phospholipids in very small VLDL (mmol/l)	664	0.02	-0.04	0.09	0.474	664	0.04	-0.03	0.12	0.233	664	0.01	-0.05	0.07	0.788
Total cholesterol in very small VLDL (mmol/l)	664	0.00	-0.07	0.07	0.958	664	0.03	-0.04	0.10	0.377	664	0.00	-0.06	0.07	0.889
Cholesterol esters in very small VLDL (mmol/l)	664	-0.01	-0.08	0.06	0.855	664	0.03	-0.04	0.10	0.448	664	0.00	-0.06	0.06	0.989
Free cholesterol in very small VLDL (mmol/l)	664	0.02	-0.05	0.09	0.539	664	0.04	-0.03	0.11	0.282	664	0.02	-0.04	0.08	0.571
Triglycerides in very small VLDL (mmol/l)	664	0.02	-0.05	0.08	0.631	664	0.02	-0.05	0.10	0.513	664	0.01	-0.06	0.08	0.780
Concentration of IDL particles (mol/l)	664	0.02	-0.04	0.09	0.482	664	0.04	-0.03	0.12	0.241	664	0.00	-0.05	0.06	0.886
Total lipids in IDL (mmol/l)	664	0.02	-0.04	0.09	0.478	664	0.05	-0.03	0.12	0.220	664	0.00	-0.05	0.06	0.888
Phospholipids in IDL (mmol/l)	664	0.02	-0.05	0.09	0.545	664	0.04	-0.03	0.11	0.297	664	0.00	-0.06	0.06	0.987
Total cholesterol in IDL (mmol/l)	664	0.03	-0.04	0.09	0.455	664	0.05	-0.02	0.12	0.165	664	0.01	-0.05	0.06	0.779
Cholesterol esters in IDL (mmol/l)	664	0.03	-0.04	0.10	0.434	664	0.06	-0.02	0.13	0.133	664	0.01	-0.04	0.07	0.671
Free cholesterol in IDL (mmol/l)	664	0.02	-0.05	0.09	0.519	664	0.04	-0.03	0.11	0.278	664	0.00	-0.06	0.06	0.983
Triglycerides in IDL (mmol/l)	664	0.02	-0.05	0.08	0.648	664	0.01	-0.06	0.08	0.763	664	0.00	-0.06	0.07	0.938
Concentration of large LDL particles (mol/l)	664	0.02	-0.05	0.08	0.608	664	0.03	-0.04	0.11	0.363	664	0.00	-0.06	0.06	0.933
Total lipids in large LDL (mmol/l)	664	0.02	-0.05	0.09	0.564	664	0.04	-0.04	0.11	0.311	664	0.00	-0.06	0.05	0.919
Phospholipids in large LDL (mmol/l)	664	0.02	-0.05	0.09	0.545	664	0.04	-0.03	0.11	0.282	664	0.00	-0.06	0.06	0.975
Total cholesterol in large LDL (mmol/l)	664	0.02	-0.05	0.09	0.559	664	0.04	-0.03	0.11	0.277	664	0.00	-0.06	0.05	0.914
Cholesterol esters in large LDL (mmol/l)	664	0.02	-0.05	0.09	0.552	664	0.04	-0.03	0.11	0.262	664	0.00	-0.06	0.05	0.928
Free cholesterol in large LDL (mmol/l)	664	0.02	-0.05	0.09	0.579	664	0.04	-0.04	0.11	0.328	664	0.00	-0.06	0.05	0.886
Triglycerides in large LDL (mmol/l)	664	0.01	-0.05	0.07	0.778	664	0.00	-0.07	0.07	0.948	664	0.00	-0.06	0.06	0.956
Concentration of medium LDL particles (mol/l)	664	0.01	-0.05	0.08	0.677	664	0.03	-0.04	0.10	0.421	664	0.00	-0.07	0.06	0.908
Total lipids in medium LDL (mmol/l)	664	0.02	-0.05	0.08	0.630	664	0.03	-0.04	0.11	0.367	664	-0.01	-0.07	0.05	0.845
Phospholipids in medium LDL (mmol/l)	664	0.02	-0.05	0.09	0.554	664	0.04	-0.03	0.11	0.296	664	0.00	-0.06	0.05	0.885

S3 Table Observational associations of age at menarche (per year later) with adiposity and cardiometabolic traits at age 18y among females in ALSPAC

	Adj. for age, education					*Adj. for age, education, BMI at age 8y*					*Adj. for age, education, outcome value at age 8y*				
**Standardised outcome at age 18y**	**N**	**Beta**	**LCL**	**UCL**	**P-value**	**N**	**Beta**	**LCL**	**UCL**	**P-value**	**N**	**Beta**	**LCL**	**UCL**	**P-value**
Total cholesterol in medium LDL (mmol/l)	664	0.02	-0.05	0.08	0.631	664	0.04	-0.04	0.11	0.332	664	-0.01	-0.06	0.05	0.851
Cholesterol esters in medium LDL (mmol/l)	664	0.02	-0.05	0.08	0.647	664	0.04	-0.04	0.11	0.340	664	0.00	-0.06	0.05	0.873
Free cholesterol in medium LDL (mmol/l)	664	0.02	-0.05	0.09	0.572	664	0.04	-0.04	0.11	0.309	664	-0.01	-0.07	0.05	0.781
Triglycerides in medium LDL (mmol/l)	664	0.00	-0.06	0.06	0.934	664	-0.01	-0.08	0.06	0.838	664	0.00	-0.07	0.06	0.887
Concentration of small LDL particles (mol/l)	664	0.01	-0.05	0.08	0.710	664	0.03	-0.05	0.10	0.462	664	-0.01	-0.07	0.05	0.789
Total lipids in small LDL (mmol/l)	664	0.02	-0.05	0.08	0.654	664	0.03	-0.04	0.11	0.378	664	-0.01	-0.07	0.05	0.774
Phospholipids in small LDL (mmol/l)	664	0.02	-0.04	0.09	0.494	664	0.04	-0.03	0.11	0.274	664	-0.01	-0.06	0.05	0.869
Total cholesterol in small LDL (mmol/l)	664	0.01	-0.05	0.08	0.696	664	0.03	-0.04	0.11	0.382	664	-0.01	-0.07	0.05	0.738
Cholesterol esters in small LDL (mmol/l)	664	0.01	-0.06	0.08	0.721	664	0.03	-0.04	0.10	0.396	664	-0.01	-0.07	0.05	0.782
Free cholesterol in small LDL (mmol/l)	664	0.02	-0.05	0.09	0.606	664	0.04	-0.04	0.11	0.340	664	-0.01	-0.07	0.05	0.719
Triglycerides in small LDL (mmol/l)	664	0.01	-0.06	0.07	0.836	664	0.01	-0.06	0.08	0.879	664	0.00	-0.06	0.06	0.970
Concentration of very large HDL particles (mol/l)	664	0.02	-0.04	0.09	0.504	664	0.02	-0.06	0.09	0.684	664	0.00	-0.06	0.05	0.884
Total lipids in very large HDL (mmol/l)	664	0.02	-0.05	0.09	0.545	664	0.01	-0.06	0.09	0.692	664	-0.01	-0.07	0.05	0.809
Phospholipids in very large HDL (mmol/l)	664	0.03	-0.04	0.09	0.430	664	0.02	-0.06	0.09	0.672	664	0.00	-0.06	0.05	0.968
Total cholesterol in very large HDL (mmol/l)	664	0.01	-0.06	0.08	0.775	664	0.01	-0.07	0.08	0.813	664	-0.02	-0.08	0.05	0.623
Cholesterol esters in very large HDL (mmol/l)	664	0.00	-0.06	0.07	0.893	664	0.00	-0.07	0.08	0.904	664	-0.02	-0.08	0.04	0.544
Free cholesterol in very large HDL (mmol/l)	664	0.02	-0.04	0.09	0.486	664	0.02	-0.05	0.10	0.584	664	0.00	-0.06	0.06	0.944
Triglycerides in very large HDL (mmol/l)	664	0.04	-0.02	0.11	0.146	664	0.06	-0.01	0.12	0.083	664	0.04	-0.01	0.10	0.143
Concentration of large HDL particles (mol/l)	664	0.03	-0.04	0.10	0.400	664	0.01	-0.07	0.08	0.884	664	0.00	-0.05	0.05	0.989
Total lipids in large HDL (mmol/l)	664	0.03	-0.03	0.10	0.336	664	0.01	-0.06	0.08	0.773	664	0.00	-0.05	0.06	0.906
Phospholipids in large HDL (mmol/l)	664	0.03	-0.04	0.10	0.394	664	0.01	-0.06	0.08	0.858	664	0.00	-0.06	0.05	0.952
Total cholesterol in large HDL (mmol/l)	664	0.03	-0.03	0.10	0.314	664	0.01	-0.06	0.08	0.742	664	0.01	-0.05	0.06	0.819
Cholesterol esters in large HDL (mmol/l)	664	0.03	-0.03	0.10	0.324	664	0.01	-0.06	0.08	0.768	664	0.01	-0.05	0.06	0.823
Free cholesterol in large HDL (mmol/l)	664	0.04	-0.03	0.11	0.280	664	0.02	-0.06	0.09	0.648	664	0.01	-0.05	0.06	0.799
Triglycerides in large HDL (mmol/l)	664	0.05	-0.01	0.11	0.122	664	0.05	-0.02	0.12	0.136	664	0.04	-0.01	0.10	0.124
Concentration of medium HDL particles (mol/l)	664	0.01	-0.05	0.08	0.676	664	-0.01	-0.08	0.06	0.796	664	0.00	-0.06	0.07	0.917
Total lipids in medium HDL (mmol/l)	664	0.02	-0.05	0.08	0.620	664	-0.01	-0.07	0.06	0.848	664	0.00	-0.06	0.06	0.919
Phospholipids in medium HDL (mmol/l)	664	0.01	-0.05	0.08	0.752	664	-0.01	-0.08	0.05	0.679	664	0.00	-0.06	0.06	0.968
Total cholesterol in medium HDL (mmol/l)	664	0.02	-0.04	0.09	0.494	664	0.00	-0.07	0.07	0.982	664	0.01	-0.06	0.07	0.856
Cholesterol esters in medium HDL (mmol/l)	664	0.02	-0.04	0.09	0.515	664	0.00	-0.07	0.07	0.974	664	0.00	-0.06	0.06	0.884
Free cholesterol in medium HDL (mmol/l)	664	0.03	-0.04	0.09	0.428	664	0.01	-0.06	0.08	0.791	664	0.01	-0.05	0.07	0.762
Triglycerides in medium HDL (mmol/l)	664	0.00	-0.06	0.07	0.916	664	0.01	-0.06	0.08	0.776	664	0.01	-0.05	0.07	0.807
Concentration of small HDL particles (mol/l)	664	-0.02	-0.08	0.05	0.666	664	-0.04	-0.11	0.03	0.314	664	-0.02	-0.09	0.05	0.534
Total lipids in small HDL (mmol/l)	664	-0.01	-0.07	0.06	0.867	664	-0.03	-0.10	0.04	0.383	664	-0.01	-0.08	0.05	0.705
Phospholipids in small HDL (mmol/l)	664	-0.02	-0.08	0.05	0.640	664	-0.04	-0.11	0.03	0.295	664	-0.02	-0.09	0.05	0.538
Total cholesterol in small HDL (mmol/l)	664	0.01	-0.06	0.07	0.821	664	-0.02	-0.09	0.05	0.594	664	0.00	-0.07	0.06	0.895
Cholesterol esters in small HDL (mmol/l)	664	0.01	-0.05	0.07	0.770	664	-0.01	-0.08	0.05	0.699	664	0.00	-0.06	0.06	0.913
Free cholesterol in small HDL (mmol/l)	664	0.00	-0.07	0.07	0.975	664	-0.03	-0.10	0.04	0.392	664	-0.01	-0.08	0.05	0.740
Triglycerides in small HDL (mmol/l)	664	-0.01	-0.08	0.06	0.818	664	-0.01	-0.08	0.06	0.818	664	-0.01	-0.07	0.06	0.822
Phospholipids to total lipds ratio in chylomicrons and extremely large VLDL (%)	664	0.00	-0.04	0.03	0.776	664	0.00	-0.04	0.03	0.855	664	-0.01	-0.05	0.02	0.499
Total cholesterol to total lipids ratio in chylomicrons and extremely large VLDL (%)	664	0.03	-0.04	0.09	0.388	664	0.03	-0.03	0.10	0.328	664	0.03	-0.03	0.10	0.293
Cholesterol esters to total lipids ratio in chylomicrons and extremely large VLDL (%)	664	0.03	-0.03	0.10	0.322	664	0.04	-0.03	0.10	0.299	664	0.04	-0.03	0.10	0.245
Free cholesterol to total lipids ratio in chylomicrons and extremely large VLDL (%)	664	-0.01	-0.07	0.04	0.612	664	0.00	-0.06	0.06	0.903	664	-0.02	-0.07	0.04	0.576
Triglycerides to total lipids ratio in chylomicrons and extremely large VLDL (%)	664	-0.02	-0.07	0.03	0.480	664	-0.03	-0.08	0.03	0.365	664	-0.02	-0.07	0.03	0.414
Phospholipids to total lipds ratio in very large VLDL (%)	664	0.02	-0.04	0.08	0.521	664	0.04	-0.02	0.10	0.179	664	0.01	-0.04	0.07	0.651
Total cholesterol to total lipids ratio in very large VLDL (%)	664	0.01	-0.06	0.07	0.806	664	0.02	-0.05	0.08	0.611	664	0.01	-0.05	0.08	0.720
Cholesterol esters to total lipids ratio in very large VLDL (%)	664	0.00	-0.04	0.05	0.864	664	0.01	-0.04	0.06	0.735	664	0.01	-0.04	0.06	0.733
Free cholesterol to total lipids ratio in very large VLDL (%)	664	0.01	-0.04	0.07	0.685	664	0.02	-0.04	0.08	0.497	664	0.01	-0.04	0.07	0.697
Triglycerides to total lipids ratio in very large VLDL (%)	664	-0.02	-0.09	0.06	0.670	664	-0.03	-0.11	0.04	0.397	664	-0.02	-0.09	0.05	0.615
Phospholipids to total lipds ratio in large VLDL (%)	664	0.01	-0.05	0.07	0.683	664	0.03	-0.04	0.09	0.431	664	0.01	-0.05	0.07	0.824
Total cholesterol to total lipids ratio in large VLDL (%)	664	0.05	-0.02	0.12	0.159	664	0.08	0.01	0.15	0.032	664	0.05	-0.02	0.11	0.169

S3 Table Observational associations of age at menarche (per year later) with adiposity and cardiometabolic traits at age 18y among females in ALSPAC

	Adj. for age, education					*Adj. for age, education, BMI at age 8y*					*Adj. for age, education, outcome value at age 8y*				
**Standardised outcome at age 18y**	**N**	**Beta**	**LCL**	**UCL**	**P-value**	**N**	**Beta**	**LCL**	**UCL**	**P-value**	**N**	**Beta**	**LCL**	**UCL**	**P-value**
Cholesterol esters to total lipids ratio in large VLDL (%)	664	0.06	-0.03	0.14	0.190	664	0.08	-0.01	0.17	0.065	664	0.05	-0.03	0.14	0.194
Free cholesterol to total lipids ratio in large VLDL (%)	664	0.01	-0.02	0.03	0.682	664	0.01	-0.02	0.04	0.356	664	0.00	-0.02	0.03	0.733
Triglycerides to total lipids ratio in large VLDL (%)	664	-0.04	-0.10	0.02	0.168	664	-0.06	-0.13	0.00	0.044	664	-0.04	-0.09	0.02	0.212
Phospholipids to total lipds ratio in medium VLDL (%)	664	0.04	-0.03	0.11	0.230	664	0.03	-0.05	0.10	0.505	664	0.02	-0.05	0.08	0.598
Total cholesterol to total lipids ratio in medium VLDL (%)	664	0.05	-0.01	0.11	0.093	664	0.07	0.01	0.13	0.030	664	0.05	0.00	0.11	0.074
Cholesterol esters to total lipids ratio in medium VLDL (%)	664	0.05	-0.01	0.11	0.126	664	0.07	0.00	0.14	0.039	664	0.05	-0.01	0.11	0.080
Free cholesterol to total lipids ratio in medium VLDL (%)	664	0.03	-0.02	0.09	0.253	664	0.04	-0.02	0.10	0.211	664	0.02	-0.04	0.08	0.535
Triglycerides to total lipids ratio in medium VLDL (%)	664	-0.05	-0.12	0.01	0.080	664	-0.07	-0.14	0.00	0.039	664	-0.05	-0.11	0.01	0.091
Phospholipids to total lipds ratio in small VLDL (%)	664	-0.02	-0.08	0.04	0.508	664	-0.06	-0.13	0.00	0.057	664	-0.03	-0.08	0.03	0.391
Total cholesterol to total lipids ratio in small VLDL (%)	664	0.00	-0.07	0.06	0.924	664	0.02	-0.05	0.09	0.644	664	0.00	-0.07	0.06	0.941
Cholesterol esters to total lipids ratio in small VLDL (%)	664	-0.01	-0.07	0.06	0.820	664	0.02	-0.05	0.08	0.649	664	0.00	-0.07	0.06	0.894
Free cholesterol to total lipids ratio in small VLDL (%)	664	0.03	-0.04	0.09	0.395	664	0.00	-0.06	0.07	0.909	664	0.01	-0.05	0.07	0.655
Triglycerides to total lipids ratio in small VLDL (%)	664	0.01	-0.06	0.08	0.763	664	0.01	-0.07	0.08	0.882	664	0.01	-0.05	0.07	0.747
Phospholipids to total lipds ratio in very small VLDL (%)	664	0.05	-0.01	0.11	0.138	664	0.05	-0.02	0.11	0.150	664	0.02	-0.03	0.08	0.404
Total cholesterol to total lipids ratio in very small VLDL (%)	664	-0.04	-0.10	0.03	0.250	664	-0.02	-0.09	0.05	0.533	664	-0.03	-0.10	0.03	0.287
Cholesterol esters to total lipids ratio in very small VLDL (%)	664	-0.05	-0.11	0.02	0.150	664	-0.03	-0.10	0.04	0.447	664	-0.04	-0.11	0.03	0.239
Free cholesterol to total lipids ratio in very small VLDL (%)	664	0.02	-0.03	0.08	0.397	664	0.01	-0.05	0.07	0.768	664	0.02	-0.03	0.08	0.394
Triglycerides to total lipids ratio in very small VLDL (%)	664	0.01	-0.05	0.08	0.678	664	-0.01	-0.08	0.06	0.844	664	0.01	-0.05	0.08	0.668
Phospholipids to total lipds ratio in IDL (%)	664	-0.03	-0.09	0.03	0.287	664	-0.07	-0.13	0.00	0.043	664	-0.03	-0.09	0.02	0.249
Total cholesterol to total lipids ratio in IDL (%)	664	0.01	-0.05	0.08	0.728	664	0.05	-0.02	0.12	0.133	664	0.01	-0.05	0.07	0.708
Cholesterol esters to total lipids ratio in IDL (%)	664	0.01	-0.05	0.07	0.687	664	0.05	-0.01	0.12	0.098	664	0.02	-0.04	0.08	0.545
Free cholesterol to total lipids ratio in IDL (%)	664	0.00	-0.06	0.06	0.977	664	0.00	-0.07	0.06	0.952	664	-0.01	-0.07	0.04	0.600
Triglycerides to total lipids ratio in IDL (%)	664	0.00	-0.07	0.07	0.992	664	-0.03	-0.10	0.04	0.348	664	0.00	-0.06	0.06	0.952
Phospholipids to total lipds ratio in large LDL (%)	664	-0.02	-0.07	0.03	0.404	664	-0.04	-0.10	0.02	0.184	664	0.00	-0.04	0.04	0.927
Total cholesterol to total lipids ratio in large LDL (%)	664	0.02	-0.04	0.08	0.591	664	0.05	-0.01	0.11	0.120	664	0.00	-0.05	0.04	0.884
Cholesterol esters to total lipids ratio in large LDL (%)	664	0.02	-0.03	0.08	0.430	664	0.05	0.00	0.11	0.067	664	0.00	-0.04	0.04	0.937
Free cholesterol to total lipids ratio in large LDL (%)	664	-0.03	-0.08	0.03	0.335	664	-0.04	-0.10	0.02	0.171	664	-0.02	-0.07	0.03	0.401
Triglycerides to total lipids ratio in large LDL (%)	664	0.00	-0.06	0.06	0.957	664	-0.04	-0.10	0.03	0.278	664	0.00	-0.06	0.06	0.947
Phospholipids to total lipds ratio in medium LDL (%)	664	-0.02	-0.07	0.03	0.477	664	-0.04	-0.09	0.02	0.220	664	0.00	-0.05	0.04	0.921
Total cholesterol to total lipids ratio in medium LDL (%)	664	0.02	-0.04	0.07	0.566	664	0.05	-0.01	0.10	0.125	664	0.00	-0.04	0.04	0.989
Cholesterol esters to total lipids ratio in medium LDL (%)	664	0.02	-0.03	0.07	0.442	664	0.05	-0.01	0.10	0.111	664	0.00	-0.04	0.04	0.925
Free cholesterol to total lipids ratio in medium LDL (%)	664	-0.02	-0.07	0.03	0.346	664	-0.04	-0.09	0.01	0.153	664	-0.01	-0.06	0.03	0.644
Triglycerides to total lipids ratio in medium LDL (%)	664	0.00	-0.05	0.05	0.970	664	-0.03	-0.09	0.03	0.296	664	0.00	-0.05	0.05	0.933
Phospholipids to total lipds ratio in small LDL (%)	664	-0.01	-0.06	0.04	0.695	664	-0.03	-0.09	0.03	0.301	664	0.01	-0.04	0.05	0.748
Total cholesterol to total lipids ratio in small LDL (%)	664	0.01	-0.05	0.06	0.806	664	0.03	-0.03	0.09	0.290	664	-0.01	-0.06	0.04	0.683
Cholesterol esters to total lipids ratio in small LDL (%)	664	0.01	-0.04	0.07	0.619	664	0.04	-0.02	0.10	0.212	664	0.00	-0.05	0.04	0.859
Free cholesterol to total lipids ratio in small LDL (%)	664	-0.02	-0.07	0.03	0.378	664	-0.04	-0.09	0.02	0.179	664	-0.01	-0.06	0.03	0.556
Triglycerides to total lipids ratio in small LDL (%)	664	0.01	-0.05	0.07	0.789	664	-0.01	-0.08	0.05	0.689	664	0.01	-0.05	0.07	0.791
Phospholipids to total lipds ratio in very large HDL (%)	664	0.03	-0.03	0.09	0.314	664	0.01	-0.05	0.07	0.811	664	0.02	-0.03	0.07	0.343
Total cholesterol to total lipids ratio in very large HDL (%)	664	-0.04	-0.09	0.02	0.216	664	-0.02	-0.08	0.04	0.577	664	-0.03	-0.08	0.02	0.184
Cholesterol esters to total lipids ratio in very large HDL (%)	664	-0.04	-0.10	0.02	0.206	664	-0.02	-0.08	0.04	0.513	664	-0.03	-0.08	0.02	0.182
Free cholesterol to total lipids ratio in very large HDL (%)	664	0.03	-0.04	0.10	0.372	664	0.04	-0.03	0.11	0.238	664	0.03	-0.04	0.09	0.397
Triglycerides to total lipids ratio in very large HDL (%)	664	0.02	-0.04	0.08	0.456	664	0.05	-0.02	0.11	0.175	664	0.03	-0.02	0.09	0.245
Phospholipids to total lipds ratio in large HDL (%)	664	-0.04	-0.10	0.03	0.234	664	-0.02	-0.09	0.05	0.524	664	-0.04	-0.10	0.02	0.181
Total cholesterol to total lipids ratio in large HDL (%)	664	0.03	-0.04	0.09	0.398	664	0.01	-0.06	0.07	0.868	664	0.02	-0.03	0.08	0.417
Cholesterol esters to total lipids ratio in large HDL (%)	664	0.02	-0.04	0.09	0.481	664	0.00	-0.07	0.07	0.985	664	0.02	-0.04	0.08	0.447
Free cholesterol to total lipids ratio in large HDL (%)	664	0.04	-0.02	0.10	0.236	664	0.03	-0.04	0.09	0.395	664	0.03	-0.03	0.08	0.358
Triglycerides to total lipids ratio in large HDL (%)	664	0.00	-0.05	0.06	0.923	664	0.03	-0.03	0.09	0.356	664	0.02	-0.03	0.07	0.496
Phospholipids to total lipds ratio in medium HDL (%)	664	-0.01	-0.07	0.04	0.621	664	-0.03	-0.09	0.03	0.356	664	-0.02	-0.07	0.04	0.559
Total cholesterol to total lipids ratio in medium HDL (%)	664	0.01	-0.05	0.07	0.702	664	0.01	-0.05	0.08	0.660	664	0.01	-0.05	0.07	0.755
Cholesterol esters to total lipids ratio in medium HDL (%)	664	0.01	-0.05	0.06	0.815	664	0.01	-0.06	0.07	0.822	664	0.01	-0.05	0.06	0.837

S3 Table Observational associations of age at menarche (per year later) with adiposity and cardiometabolic traits at age 18y among females in ALSPAC

	Adj. for age, education					*Adj. for age, education, BMI at age 8y*					*Adj. for age, education, outcome value at age 8y*				
**Standardised outcome at age 18y**	**N**	**Beta**	**LCL**	**UCL**	**P-value**	**N**	**Beta**	**LCL**	**UCL**	**P-value**	**N**	**Beta**	**LCL**	**UCL**	**P-value**
Free cholesterol to total lipids ratio in medium HDL (%)	664	0.03	-0.03	0.08	0.329	664	0.04	-0.02	0.10	0.158	664	0.02	-0.04	0.07	0.531
Triglycerides to total lipids ratio in medium HDL (%)	664	0.00	-0.06	0.06	0.982	664	0.02	-0.05	0.09	0.548	664	0.01	-0.04	0.07	0.658
Phospholipids to total lipds ratio in small HDL (%)	664	-0.02	-0.08	0.03	0.394	664	-0.01	-0.08	0.05	0.652	664	-0.01	-0.06	0.04	0.713
Total cholesterol to total lipids ratio in small HDL (%)	664	0.02	-0.03	0.08	0.403	664	0.01	-0.05	0.07	0.731	664	0.01	-0.04	0.06	0.695
Cholesterol esters to total lipids ratio in small HDL (%)	664	0.02	-0.04	0.08	0.463	664	0.01	-0.05	0.07	0.725	664	0.01	-0.04	0.06	0.782
Free cholesterol to total lipids ratio in small HDL (%)	664	0.01	-0.05	0.07	0.744	664	-0.01	-0.07	0.06	0.865	664	0.02	-0.04	0.07	0.576
Triglycerides to total lipids ratio in small HDL (%)	664	0.00	-0.06	0.06	0.946	664	0.01	-0.06	0.08	0.758	664	0.00	-0.06	0.06	0.906
Mean diameter for VLDL particles (nm)	664	0.01	-0.06	0.07	0.799	664	0.03	-0.04	0.10	0.382	664	0.01	-0.05	0.07	0.680
Mean diameter for LDL particles (nm)	664	0.01	-0.04	0.07	0.620	664	0.02	-0.04	0.08	0.552	664	0.03	-0.03	0.08	0.348
Mean diameter for HDL particles (nm)	664	0.03	-0.03	0.10	0.330	664	0.02	-0.05	0.09	0.523	664	0.01	-0.04	0.07	0.646
Serum total cholesterol (mmol/l)	664	0.03	-0.04	0.09	0.420	664	0.04	-0.03	0.11	0.235	664	0.00	-0.06	0.06	0.988
Total cholesterol in VLDL (mmol/l)	664	0.02	-0.05	0.08	0.639	664	0.05	-0.02	0.12	0.166	664	0.02	-0.04	0.08	0.486
Remnant cholesterol (non-HDL, non-LDL -cholesterol) (mmol/l)	664	0.02	-0.04	0.09	0.512	664	0.05	-0.02	0.13	0.131	664	0.02	-0.04	0.07	0.550
Total cholesterol in LDL (mmol/l)	664	0.02	-0.05	0.09	0.606	664	0.04	-0.04	0.11	0.312	664	-0.01	-0.06	0.05	0.857
Total cholesterol in HDL (mmol/l)	664	0.03	-0.04	0.09	0.451	664	0.00	-0.07	0.08	0.920	664	-0.01	-0.06	0.05	0.791
Total cholesterol in HDL2 (mmol/l)	664	0.02	-0.05	0.09	0.541	664	0.00	-0.08	0.07	0.909	664	-0.01	-0.07	0.05	0.692
Total cholesterol in HDL3 (mmol/l)	664	0.03	-0.03	0.10	0.325	664	0.02	-0.05	0.09	0.622	664	0.00	-0.05	0.06	0.954
Esterified cholesterol (mmol/l)	664	0.03	-0.03	0.10	0.345	664	0.05	-0.02	0.12	0.183	664	0.00	-0.05	0.06	0.921
Free cholesterol (mmol/l)	664	0.01	-0.05	0.08	0.661	664	0.03	-0.04	0.10	0.436	664	0.00	-0.06	0.05	0.926
Serum total triglycerides (mmol/l)	664	0.02	-0.05	0.08	0.618	664	0.04	-0.03	0.10	0.296	664	0.01	-0.04	0.07	0.620
Triglycerides in VLDL (mmol/l)	664	0.01	-0.04	0.07	0.629	664	0.04	-0.03	0.10	0.235	664	0.02	-0.04	0.07	0.568
Triglycerides in LDL (mmol/l)	664	0.01	-0.06	0.07	0.832	664	0.00	-0.07	0.07	1.000	664	0.00	-0.06	0.06	0.955
Triglycerides in HDL (mmol/l)	664	0.02	-0.05	0.08	0.603	664	0.02	-0.05	0.09	0.521	664	0.02	-0.04	0.08	0.573
Diacylglycerol (mmol/l)	664	-0.01	-0.07	0.06	0.816	664	0.00	-0.07	0.07	0.922	664	0.00	-0.07	0.06	0.980
Ratio of diacylglycerol to triglycerides	664	-0.01	-0.08	0.05	0.665	664	-0.01	-0.08	0.06	0.785	664	-0.01	-0.07	0.05	0.767
Total phosphoglycerides (mmol/l)	664	0.03	-0.04	0.09	0.440	664	0.03	-0.05	0.10	0.492	664	0.01	-0.05	0.08	0.659
Ratio of triglycerides to phosphoglycerides	664	0.02	-0.04	0.08	0.602	664	0.04	-0.03	0.10	0.286	664	0.02	-0.04	0.08	0.530
Phosphatidylcholine and other cholines (mmol/l)	664	0.01	-0.05	0.08	0.667	664	0.00	-0.07	0.07	0.907	664	0.00	-0.06	0.06	0.969
Total cholines (mmol/l)	664	0.02	-0.05	0.08	0.561	664	0.02	-0.05	0.09	0.665	664	0.00	-0.06	0.06	0.896
Apolipoprotein A-I (g/l)	664	0.04	-0.03	0.10	0.267	664	0.02	-0.05	0.09	0.510	664	0.00	-0.06	0.06	0.989
Apolipoprotein B (g/l)	664	0.02	-0.04	0.09	0.475	664	0.05	-0.02	0.12	0.141	664	0.02	-0.04	0.07	0.591
Ratio of apolipoprotein B to apolipoprotein A-I	664	0.01	-0.06	0.07	0.812	664	0.04	-0.03	0.11	0.229	664	0.02	-0.04	0.07	0.583
Total fatty acids (mmol/l)	664	0.03	-0.04	0.09	0.392	664	0.04	-0.03	0.11	0.250	664	0.01	-0.05	0.08	0.646
Estimated description of fatty acid chain length, not actual carbon number	664	-0.03	-0.08	0.03	0.380	664	-0.04	-0.10	0.02	0.189	664	-0.02	-0.08	0.03	0.434
Estimated degree of unsaturation	664	-0.02	-0.07	0.04	0.580	664	-0.03	-0.09	0.03	0.374	664	-0.02	-0.08	0.04	0.514
22:6, docosahexaenoic acid (mmol/l)	664	0.03	-0.04	0.09	0.403	664	0.03	-0.04	0.10	0.409	664	0.00	-0.06	0.07	0.906
18:2, linoleic acid (mmol/l)	664	0.04	-0.03	0.10	0.278	664	0.04	-0.03	0.11	0.260	664	0.01	-0.05	0.07	0.690
Conjugated linoleic acid (mmol/l)	664	0.01	-0.05	0.07	0.769	664	0.02	-0.04	0.09	0.501	664	0.01	-0.05	0.07	0.786
Omega-3 fatty acids (mmol/l)	664	0.04	-0.03	0.10	0.288	664	0.06	-0.01	0.13	0.098	664	0.01	-0.06	0.07	0.838
Omega-6 fatty acids (mmol/l)	664	0.03	-0.04	0.09	0.399	664	0.03	-0.04	0.10	0.340	664	0.00	-0.06	0.06	0.930
Polyunsaturated fatty acids (mmol/l)	664	0.03	-0.03	0.10	0.362	664	0.04	-0.03	0.11	0.276	664	0.00	-0.06	0.06	0.940
Monounsaturated fatty acids; 16:1, 18:1 (mmol/l)	664	0.02	-0.05	0.08	0.607	664	0.03	-0.04	0.09	0.467	664	0.01	-0.05	0.07	0.751
Saturated fatty acids (mmol/l)	664	0.03	-0.03	0.09	0.349	664	0.05	-0.02	0.12	0.172	664	0.02	-0.04	0.09	0.451
Ratio of 22:6 docosahexaenoic acid to total fatty acids (%)	664	0.01	-0.05	0.08	0.675	664	0.01	-0.06	0.07	0.809	664	0.00	-0.06	0.06	0.929
Ratio of 18:2 linoleic acid to total fatty acids (%)	664	0.01	-0.06	0.07	0.800	664	-0.01	-0.07	0.06	0.830	664	0.00	-0.06	0.07	0.903
Ratio of conjugated linoleic acid to total fatty acids (%)	664	0.01	-0.05	0.07	0.788	664	0.02	-0.05	0.09	0.600	664	0.01	-0.05	0.07	0.788
Ratio of omega-3 fatty acids to total fatty acids (%)	664	0.02	-0.04	0.09	0.538	664	0.04	-0.03	0.11	0.284	664	0.00	-0.07	0.07	0.995
Ratio of omega-6 fatty acids to total fatty acids (%)	664	-0.01	-0.07	0.06	0.824	664	-0.02	-0.09	0.04	0.495	664	-0.01	-0.07	0.05	0.717
Ratio of polyunsaturated fatty acids to total fatty acids (%)	664	0.00	-0.06	0.06	0.955	664	-0.01	-0.08	0.05	0.707	664	-0.01	-0.07	0.05	0.771
Ratio of monounsaturated fatty acids to total fatty acids (%)	664	-0.01	-0.07	0.06	0.843	664	-0.01	-0.07	0.06	0.797	664	-0.01	-0.07	0.05	0.814
Ratio of saturated fatty acids to total fatty acids (%)	664	0.01	-0.04	0.07	0.699	664	0.03	-0.03	0.08	0.349	664	0.02	-0.04	0.07	0.527

S3 Table Observational associations of age at menarche (per year later) with adiposity and cardiometabolic traits at age 18y among females in ALSPAC

	Adj. for age, education					*Adj. for age, education, BMI at age 8y*					*Adj. for age, education, outcome value at age 8y*				
**Standardised outcome at age 18y**	**N**	**Beta**	**LCL**	**UCL**	**P-value**	**N**	**Beta**	**LCL**	**UCL**	**P-value**	**N**	**Beta**	**LCL**	**UCL**	**P-value**
Glucose (mmol/l)	664	0.01	-0.06	0.07	0.859	664	0.02	-0.04	0.08	0.480	664	0.01	-0.05	0.08	0.665
Lactate (mmol/l)	664	0.03	-0.04	0.10	0.471	664	0.03	-0.05	0.10	0.495	664	0.02	-0.05	0.09	0.513
Pyruvate (mmol/l)	664	-0.01	-0.08	0.05	0.735	664	0.00	-0.07	0.07	0.988	664	-0.01	-0.08	0.05	0.697
Citrate (mmol/l)	664	0.02	-0.04	0.09	0.520	664	0.00	-0.07	0.07	0.984	664	0.01	-0.05	0.08	0.668
Alanine (mmol/l)	664	0.06	-0.02	0.13	0.129	664	0.07	-0.01	0.15	0.080	664	0.06	-0.01	0.13	0.116
Glutamine (mmol/l)	664	0.07	0.01	0.14	0.034	664	0.08	0.01	0.14	0.029	664	0.07	0.01	0.13	0.033
Histidine (mmol/l)	664	0.02	-0.05	0.09	0.611	664	0.02	-0.05	0.09	0.643	664	0.03	-0.04	0.10	0.386
Isoleucine (mmol/l)	664	0.03	-0.03	0.09	0.397	664	0.04	-0.02	0.11	0.203	664	0.03	-0.03	0.09	0.353
Leucine (mmol/l)	664	0.03	-0.02	0.09	0.245	664	0.05	-0.01	0.11	0.136	664	0.04	-0.02	0.09	0.215
Valine (mmol/l)	664	0.00	-0.06	0.06	0.973	664	0.02	-0.04	0.09	0.431	664	0.00	-0.06	0.06	0.979
Phenylalanine (mmol/l)	664	-0.06	-0.13	0.00	0.058	664	-0.05	-0.12	0.02	0.171	664	-0.06	-0.12	0.01	0.088
Tyrosine (mmol/l)	664	-0.03	-0.10	0.04	0.422	664	0.01	-0.07	0.09	0.839	664	-0.03	-0.10	0.04	0.429
Acetate (mmol/l)	664	-0.03	-0.12	0.06	0.504	664	-0.04	-0.13	0.05	0.403	664	-0.03	-0.12	0.06	0.485
Acetoacetate (mmol/l)	664	-0.01	-0.07	0.05	0.687	664	-0.03	-0.09	0.04	0.389	664	-0.01	-0.07	0.05	0.677
3-hydroxybutyrate (mmol/l)	664	-0.03	-0.10	0.04	0.433	664	-0.05	-0.13	0.02	0.145	664	-0.03	-0.10	0.04	0.415
Creatinine (mmol/l)	664	-0.06	-0.11	-0.01	0.013	664	-0.05	-0.10	0.00	0.050	664	-0.04	-0.08	0.01	0.104
Albumin (signal area)	664	0.05	-0.01	0.12	0.084	664	0.06	-0.01	0.12	0.081	664	0.06	0.00	0.12	0.044
Glycoprotein acetyls, mainly a1-acid glycoprotein (mmol/l)	664	-0.03	-0.09	0.04	0.411	664	0.00	-0.06	0.06	0.995	664	-0.01	-0.07	0.04	0.630
