# Supplementary material for: Influence of puberty timing on adiposity and cardiometabolic traits: A Mendelian randomisation study
Source: PLoS Med. 2018 Aug 28;15(8):e1002641. doi: 10.1371/journal.pmed.1002641 (PMC6112630; doi:10.1371/journal.pmed.1002641)
Supplement: S4 Table — (PDF) [file pmed.1002641.s023.pdf]

S4 Table Observational associations of age at voice breaking (per year later) with adiposity and cardiometabolic traits at age 18y among males in ALSPAC

	Adj. for age, education					*Adj. for age, education, BMI at age 8y*					*Adj. for age, education, outcome value at age 8y*				
**Standardised outcome at age 18y**	**N**	**Beta**	**LCL**	**UCL**	**P-value**	**N**	**Beta**	**LCL**	**UCL**	**P-value**	**N**	**Beta**	**LCL**	**UCL**	**P-value**
Body mass index (kg/m^2)	1554	-0.05	-0.08	-0.02	1.88E-03	1428	-0.01	-0.03	0.01	0.404	1428	-0.01	-0.03	0.01	0.404
Fat mass index (kg/m^2)	1511	-0.03	-0.05	0.00	0.066	1392	0.00	-0.02	0.03	0.764	1351	0.00	-0.02	0.02	0.756
Lean mass index (kg/m^2)	1511	-0.04	-0.06	-0.01	1.64E-03	1392	-0.02	-0.04	0.00	0.077	1351	-0.02	-0.04	0.00	0.013
Systolic blood pressure (mmHg)	1474	-0.03	-0.06	0.00	0.039	1362	-0.02	-0.05	0.01	0.157	1343	-0.02	-0.05	0.01	0.158
Diastolic blood pressure (mmHg)	1474	-0.02	-0.05	0.01	0.219	1362	-0.01	-0.05	0.02	0.517	1342	-0.01	-0.05	0.02	0.417
Concentration of chylomicrons and extremely large VLDL particles (mol/l)	1109	-0.01	-0.05	0.04	0.686	1025	0.00	-0.04	0.05	0.838	785	0.00	-0.06	0.05	0.939
Total lipids in chylomicrons and extremely large VLDL (mmol/l)	1109	-0.01	-0.05	0.03	0.673	1025	0.00	-0.04	0.05	0.837	785	0.00	-0.06	0.05	0.941
Phospholipids in chylomicrons and extremely large VLDL (mmol/l)	1109	-0.01	-0.05	0.04	0.692	1025	0.01	-0.04	0.05	0.821	785	0.00	-0.06	0.06	0.971
Total cholesterol in chylomicrons and extremely large VLDL (mmol/l)	1109	-0.01	-0.05	0.03	0.583	1025	0.00	-0.04	0.05	0.935	785	-0.01	-0.06	0.05	0.799
Cholesterol esters in chylomicrons and extremely large VLDL (mmol/l)	1109	-0.01	-0.06	0.03	0.524	1025	0.00	-0.04	0.04	0.992	785	-0.01	-0.06	0.04	0.661
Free cholesterol in chylomicrons and extremely large VLDL (mmol/l)	1109	-0.01	-0.05	0.03	0.670	1025	0.00	-0.04	0.05	0.846	785	0.00	-0.06	0.06	0.961
Triglycerides in chylomicrons and extremely large VLDL (mmol/l)	1109	-0.01	-0.05	0.04	0.696	1025	0.01	-0.04	0.05	0.814	785	0.00	-0.06	0.06	0.973
Concentration of very large VLDL particles (mol/l)	1109	-0.01	-0.05	0.04	0.688	1025	0.01	-0.04	0.05	0.802	785	0.00	-0.06	0.05	0.891
Total lipids in very large VLDL (mmol/l)	1109	-0.01	-0.05	0.03	0.662	1025	0.01	-0.04	0.05	0.820	785	0.00	-0.06	0.05	0.867
Phospholipids in very large VLDL (mmol/l)	1109	-0.01	-0.05	0.03	0.642	1025	0.00	-0.04	0.05	0.866	785	-0.01	-0.06	0.05	0.848
Total cholesterol in very large VLDL (mmol/l)	1109	-0.01	-0.05	0.03	0.642	1025	0.00	-0.04	0.05	0.862	785	-0.01	-0.06	0.05	0.855
Cholesterol esters in very large VLDL (mmol/l)	1109	-0.01	-0.05	0.03	0.645	1025	0.00	-0.04	0.05	0.847	785	-0.01	-0.06	0.05	0.837
Free cholesterol in very large VLDL (mmol/l)	1109	-0.01	-0.05	0.03	0.634	1025	0.00	-0.04	0.05	0.886	785	0.00	-0.06	0.05	0.872
Triglycerides in very large VLDL (mmol/l)	1109	-0.01	-0.05	0.03	0.676	1025	0.01	-0.04	0.05	0.796	785	0.00	-0.06	0.05	0.876
Concentration of large VLDL particles (mol/l)	1109	-0.01	-0.05	0.03	0.662	1025	0.01	-0.04	0.05	0.777	785	-0.01	-0.06	0.05	0.837
Total lipids in large VLDL (mmol/l)	1109	-0.01	-0.05	0.03	0.667	1025	0.01	-0.04	0.05	0.772	785	-0.01	-0.06	0.05	0.856
Phospholipids in large VLDL (mmol/l)	1109	-0.01	-0.05	0.04	0.798	1025	0.01	-0.03	0.06	0.646	785	0.00	-0.06	0.06	0.967
Total cholesterol in large VLDL (mmol/l)	1109	-0.01	-0.06	0.03	0.555	1025	0.00	-0.04	0.05	0.921	785	-0.01	-0.06	0.04	0.730
Cholesterol esters in large VLDL (mmol/l)	1109	-0.01	-0.06	0.03	0.507	1025	0.00	-0.04	0.04	0.979	785	-0.01	-0.06	0.04	0.673
Free cholesterol in large VLDL (mmol/l)	1109	-0.01	-0.05	0.03	0.606	1025	0.00	-0.04	0.05	0.867	785	-0.01	-0.06	0.05	0.787
Triglycerides in large VLDL (mmol/l)	1109	-0.01	-0.05	0.03	0.676	1025	0.01	-0.04	0.05	0.758	785	-0.01	-0.06	0.05	0.853
Concentration of medium VLDL particles (mol/l)	1109	-0.01	-0.05	0.03	0.646	1025	0.01	-0.04	0.05	0.773	785	-0.01	-0.06	0.05	0.823
Total lipids in medium VLDL (mmol/l)	1109	-0.01	-0.05	0.03	0.586	1025	0.00	-0.04	0.05	0.845	785	-0.01	-0.06	0.04	0.761
Phospholipids in medium VLDL (mmol/l)	1109	-0.01	-0.05	0.03	0.544	1025	0.00	-0.04	0.04	0.899	785	-0.01	-0.06	0.04	0.743
Total cholesterol in medium VLDL (mmol/l)	1109	-0.02	-0.06	0.02	0.352	1025	-0.01	-0.05	0.04	0.791	785	-0.02	-0.06	0.03	0.522
Cholesterol esters in medium VLDL (mmol/l)	1109	-0.02	-0.06	0.02	0.252	1025	-0.01	-0.05	0.03	0.582	785	-0.02	-0.06	0.03	0.388
Free cholesterol in medium VLDL (mmol/l)	1109	-0.01	-0.05	0.03	0.514	1025	0.00	-0.04	0.04	0.945	785	-0.01	-0.06	0.04	0.717
Triglycerides in medium VLDL (mmol/l)	1109	-0.01	-0.05	0.03	0.743	1025	0.01	-0.03	0.05	0.661	785	0.00	-0.06	0.05	0.893
Concentration of small VLDL particles (mol/l)	1109	-0.01	-0.05	0.03	0.531	1025	0.00	-0.04	0.04	0.970	785	-0.01	-0.06	0.03	0.572
Total lipids in small VLDL (mmol/l)	1109	-0.01	-0.05	0.02	0.495	1025	0.00	-0.04	0.04	0.949	785	-0.02	-0.06	0.03	0.458
Phospholipids in small VLDL (mmol/l)	1109	-0.01	-0.05	0.02	0.520	1025	0.00	-0.04	0.04	0.966	785	-0.02	-0.06	0.02	0.344
Total cholesterol in small VLDL (mmol/l)	1109	-0.01	-0.05	0.02	0.412	1025	-0.01	-0.04	0.03	0.690	785	-0.02	-0.06	0.01	0.237
Cholesterol esters in small VLDL (mmol/l)	1109	-0.01	-0.05	0.02	0.437	1025	-0.01	-0.05	0.03	0.649	785	-0.02	-0.06	0.02	0.273
Free cholesterol in small VLDL (mmol/l)	1109	-0.01	-0.05	0.02	0.419	1025	0.00	-0.04	0.03	0.805	785	-0.02	-0.06	0.02	0.288
Triglycerides in small VLDL (mmol/l)	1109	-0.01	-0.05	0.03	0.605	1025	0.00	-0.04	0.04	0.847	785	-0.01	-0.06	0.04	0.717
Concentration of very small VLDL particles (mol/l)	1109	-0.02	-0.05	0.02	0.320	1025	-0.01	-0.05	0.02	0.433	785	-0.02	-0.05	0.01	0.177
Total lipids in very small VLDL (mmol/l)	1109	-0.01	-0.04	0.02	0.487	1025	-0.01	-0.05	0.03	0.578	785	-0.02	-0.05	0.01	0.222
Phospholipids in very small VLDL (mmol/l)	1109	-0.02	-0.05	0.01	0.176	1025	-0.02	-0.05	0.01	0.211	785	-0.03	-0.06	0.01	0.102
Total cholesterol in very small VLDL (mmol/l)	1109	0.00	-0.04	0.03	0.886	1025	0.00	-0.04	0.03	0.848	785	-0.01	-0.05	0.03	0.577
Cholesterol esters in very small VLDL (mmol/l)	1109	0.00	-0.04	0.03	0.928	1025	0.00	-0.04	0.04	0.909	785	-0.01	-0.05	0.03	0.660
Free cholesterol in very small VLDL (mmol/l)	1109	0.00	-0.04	0.03	0.805	1025	-0.01	-0.04	0.03	0.723	785	-0.01	-0.05	0.02	0.442
Triglycerides in very small VLDL (mmol/l)	1109	-0.01	-0.05	0.02	0.419	1025	0.00	-0.04	0.03	0.787	785	-0.01	-0.05	0.03	0.523
Concentration of IDL particles (mol/l)	1109	-0.02	-0.06	0.01	0.134	1025	-0.03	-0.06	0.01	0.137	785	-0.02	-0.06	0.01	0.125
Total lipids in IDL (mmol/l)	1109	-0.02	-0.06	0.01	0.151	1025	-0.02	-0.06	0.01	0.152	785	-0.03	-0.06	0.00	0.094
Phospholipids in IDL (mmol/l)	1109	-0.03	-0.06	0.01	0.111	1025	-0.03	-0.06	0.01	0.099	785	-0.03	-0.06	0.00	0.093
Total cholesterol in IDL (mmol/l)	1109	-0.02	-0.06	0.01	0.185	1025	-0.02	-0.06	0.01	0.183	785	-0.03	-0.06	0.00	0.090
Cholesterol esters in IDL (mmol/l)	1109	-0.02	-0.05	0.01	0.207	1025	-0.02	-0.06	0.01	0.229	785	-0.03	-0.06	0.01	0.101

S4 Table Observational associations of age at voice breaking (per year later) with adiposity and cardiometabolic traits at age 18y among males in ALSPAC

	Adj. for age, education					*Adj. for age, education, BMI at age 8y*					*Adj. for age, education, outcome value at age 8y*				
**Standardised outcome at age 18y**	**N**	**Beta**	**LCL**	**UCL**	**P-value**	**N**	**Beta**	**LCL**	**UCL**	**P-value**	**N**	**Beta**	**LCL**	**UCL**	**P-value**
Free cholesterol in IDL (mmol/l)	1109	-0.02	-0.06	0.01	0.152	1025	-0.03	-0.06	0.01	0.110	785	-0.03	-0.06	0.00	0.097
Triglycerides in IDL (mmol/l)	1109	-0.02	-0.04	0.01	0.270	1025	-0.01	-0.04	0.02	0.381	785	-0.01	-0.04	0.02	0.573
Concentration of large LDL particles (mol/l)	1109	-0.03	-0.06	0.01	0.119	1025	-0.03	-0.06	0.01	0.135	785	-0.03	-0.06	0.01	0.106
Total lipids in large LDL (mmol/l)	1109	-0.02	-0.06	0.01	0.128	1025	-0.03	-0.06	0.01	0.142	785	-0.03	-0.06	0.00	0.096
Phospholipids in large LDL (mmol/l)	1109	-0.03	-0.06	0.01	0.124	1025	-0.02	-0.06	0.01	0.151	785	-0.03	-0.06	0.00	0.090
Total cholesterol in large LDL (mmol/l)	1109	-0.02	-0.06	0.01	0.137	1025	-0.03	-0.06	0.01	0.144	785	-0.03	-0.06	0.00	0.079
Cholesterol esters in large LDL (mmol/l)	1109	-0.02	-0.06	0.01	0.133	1025	-0.03	-0.06	0.01	0.151	785	-0.03	-0.06	0.00	0.078
Free cholesterol in large LDL (mmol/l)	1109	-0.02	-0.06	0.01	0.157	1025	-0.03	-0.06	0.01	0.130	785	-0.03	-0.06	0.00	0.093
Triglycerides in large LDL (mmol/l)	1109	-0.02	-0.04	0.01	0.219	1025	-0.02	-0.04	0.01	0.287	785	-0.01	-0.04	0.02	0.580
Concentration of medium LDL particles (mol/l)	1109	-0.03	-0.06	0.01	0.108	1025	-0.03	-0.06	0.01	0.145	785	-0.03	-0.06	0.01	0.108
Total lipids in medium LDL (mmol/l)	1109	-0.02	-0.06	0.01	0.144	1025	-0.02	-0.06	0.01	0.177	785	-0.03	-0.06	0.01	0.107
Phospholipids in medium LDL (mmol/l)	1109	-0.02	-0.05	0.01	0.177	1025	-0.02	-0.05	0.01	0.269	785	-0.03	-0.06	0.01	0.104
Total cholesterol in medium LDL (mmol/l)	1109	-0.02	-0.06	0.01	0.149	1025	-0.02	-0.06	0.01	0.167	785	-0.03	-0.06	0.00	0.088
Cholesterol esters in medium LDL (mmol/l)	1109	-0.02	-0.06	0.01	0.135	1025	-0.03	-0.06	0.01	0.153	785	-0.03	-0.06	0.00	0.096
Free cholesterol in medium LDL (mmol/l)	1109	-0.02	-0.05	0.01	0.238	1025	-0.02	-0.06	0.02	0.256	785	-0.03	-0.06	0.00	0.090
Triglycerides in medium LDL (mmol/l)	1109	-0.02	-0.05	0.01	0.233	1025	-0.02	-0.05	0.02	0.321	785	-0.01	-0.04	0.03	0.723
Concentration of small LDL particles (mol/l)	1109	-0.02	-0.06	0.01	0.185	1025	-0.02	-0.06	0.01	0.242	785	-0.03	-0.06	0.01	0.131
Total lipids in small LDL (mmol/l)	1109	-0.02	-0.05	0.01	0.173	1025	-0.02	-0.06	0.01	0.215	785	-0.03	-0.06	0.00	0.094
Phospholipids in small LDL (mmol/l)	1109	-0.02	-0.05	0.01	0.231	1025	-0.02	-0.05	0.02	0.342	785	-0.03	-0.06	0.01	0.115
Total cholesterol in small LDL (mmol/l)	1109	-0.02	-0.06	0.01	0.190	1025	-0.02	-0.06	0.01	0.204	785	-0.03	-0.06	0.00	0.089
Cholesterol esters in small LDL (mmol/l)	1109	-0.02	-0.06	0.01	0.154	1025	-0.02	-0.06	0.01	0.167	785	-0.03	-0.06	0.01	0.105
Free cholesterol in small LDL (mmol/l)	1109	-0.01	-0.05	0.02	0.450	1025	-0.01	-0.05	0.02	0.465	785	-0.03	-0.06	0.01	0.102
Triglycerides in small LDL (mmol/l)	1109	-0.02	-0.05	0.01	0.148	1025	-0.02	-0.05	0.02	0.311	785	-0.01	-0.05	0.02	0.435
Concentration of very large HDL particles (mol/l)	1109	-0.01	-0.04	0.02	0.481	1025	-0.02	-0.05	0.01	0.252	785	0.00	-0.03	0.03	0.898
Total lipids in very large HDL (mmol/l)	1109	-0.01	-0.04	0.03	0.691	1025	-0.01	-0.05	0.02	0.405	785	0.00	-0.03	0.04	0.814
Phospholipids in very large HDL (mmol/l)	1109	-0.01	-0.05	0.02	0.358	1025	-0.02	-0.06	0.01	0.169	785	0.00	-0.03	0.03	0.956
Total cholesterol in very large HDL (mmol/l)	1109	0.00	-0.03	0.04	0.801	1025	0.00	-0.04	0.03	0.892	785	0.01	-0.03	0.04	0.695
Cholesterol esters in very large HDL (mmol/l)	1109	0.01	-0.03	0.04	0.606	1025	0.00	-0.03	0.04	0.890	785	0.01	-0.03	0.05	0.595
Free cholesterol in very large HDL (mmol/l)	1109	-0.01	-0.04	0.02	0.614	1025	-0.02	-0.05	0.02	0.375	785	0.00	-0.03	0.03	0.981
Triglycerides in very large HDL (mmol/l)	1109	-0.02	-0.05	0.02	0.289	1025	-0.02	-0.05	0.02	0.341	785	-0.01	-0.05	0.03	0.709
Concentration of large HDL particles (mol/l)	1109	-0.02	-0.05	0.02	0.336	1025	-0.02	-0.06	0.01	0.166	785	0.00	-0.03	0.03	0.809
Total lipids in large HDL (mmol/l)	1109	-0.02	-0.05	0.02	0.336	1025	-0.02	-0.06	0.01	0.175	785	0.00	-0.04	0.03	0.789
Phospholipids in large HDL (mmol/l)	1109	-0.02	-0.05	0.01	0.252	1025	-0.03	-0.06	0.01	0.131	785	-0.01	-0.04	0.02	0.663
Total cholesterol in large HDL (mmol/l)	1109	-0.01	-0.05	0.02	0.458	1025	-0.02	-0.05	0.01	0.244	785	0.00	-0.03	0.03	0.918
Cholesterol esters in large HDL (mmol/l)	1109	-0.01	-0.04	0.02	0.468	1025	-0.02	-0.05	0.01	0.251	785	0.00	-0.03	0.03	0.940
Free cholesterol in large HDL (mmol/l)	1109	-0.01	-0.05	0.02	0.431	1025	-0.02	-0.06	0.01	0.226	785	0.00	-0.04	0.03	0.846
Triglycerides in large HDL (mmol/l)	1109	-0.04	-0.07	-0.01	0.021	1025	-0.04	-0.07	-0.01	0.022	785	-0.02	-0.06	0.01	0.223
Concentration of medium HDL particles (mol/l)	1109	-0.02	-0.05	0.01	0.273	1025	-0.02	-0.05	0.01	0.306	785	-0.02	-0.05	0.02	0.289
Total lipids in medium HDL (mmol/l)	1109	-0.01	-0.05	0.02	0.360	1025	-0.01	-0.05	0.02	0.386	785	-0.01	-0.05	0.02	0.400
Phospholipids in medium HDL (mmol/l)	1109	-0.01	-0.04	0.02	0.404	1025	-0.01	-0.04	0.02	0.413	785	-0.01	-0.04	0.02	0.446
Total cholesterol in medium HDL (mmol/l)	1109	-0.01	-0.05	0.02	0.486	1025	-0.01	-0.05	0.02	0.485	785	-0.01	-0.05	0.02	0.508
Cholesterol esters in medium HDL (mmol/l)	1109	-0.01	-0.05	0.02	0.545	1025	-0.01	-0.05	0.02	0.543	785	-0.01	-0.05	0.03	0.586
Free cholesterol in medium HDL (mmol/l)	1109	-0.02	-0.05	0.01	0.266	1025	-0.02	-0.05	0.02	0.279	785	-0.02	-0.06	0.02	0.254
Triglycerides in medium HDL (mmol/l)	1109	-0.03	-0.06	0.00	0.076	1025	-0.02	-0.05	0.01	0.219	785	-0.02	-0.06	0.01	0.201
Concentration of small HDL particles (mol/l)	1109	-0.01	-0.04	0.02	0.588	1025	0.00	-0.03	0.03	0.878	785	-0.01	-0.04	0.02	0.585
Total lipids in small HDL (mmol/l)	1109	-0.01	-0.04	0.02	0.371	1025	-0.01	-0.04	0.02	0.544	785	-0.02	-0.05	0.02	0.343
Phospholipids in small HDL (mmol/l)	1109	0.00	-0.04	0.03	0.897	1025	0.00	-0.03	0.04	0.848	785	-0.01	-0.04	0.03	0.761
Total cholesterol in small HDL (mmol/l)	1109	-0.02	-0.05	0.01	0.130	1025	-0.02	-0.05	0.01	0.154	785	-0.02	-0.05	0.01	0.166
Cholesterol esters in small HDL (mmol/l)	1109	-0.02	-0.05	0.00	0.082	1025	-0.02	-0.05	0.00	0.096	785	-0.02	-0.06	0.01	0.152
Free cholesterol in small HDL (mmol/l)	1109	0.00	-0.04	0.03	0.828	1025	0.00	-0.03	0.03	0.928	785	-0.01	-0.05	0.02	0.510
Triglycerides in small HDL (mmol/l)	1109	-0.01	-0.05	0.02	0.476	1025	0.00	-0.04	0.03	0.907	785	0.00	-0.05	0.04	0.852
Phospholipids to total lipds ratio in chylomicrons and extremely large VLDL (%)	1109	0.03	-0.03	0.10	0.320	1025	0.04	-0.03	0.11	0.280	785	0.05	-0.03	0.14	0.233

S4 Table Observational associations of age at voice breaking (per year later) with adiposity and cardiometabolic traits at age 18y among males in ALSPAC

	Adj. for age, education					*Adj. for age, education, BMI at age 8y*					*Adj. for age, education, outcome value at age 8y*				
**Standardised outcome at age 18y**	**N**	**Beta**	**LCL**	**UCL**	**P-value**	**N**	**Beta**	**LCL**	**UCL**	**P-value**	**N**	**Beta**	**LCL**	**UCL**	**P-value**
Total cholesterol to total lipids ratio in chylomicrons and extremely large VLDL (%)	1109	-0.03	-0.07	0.00	0.069	1025	-0.03	-0.07	0.01	0.115	785	-0.01	-0.06	0.03	0.516
Cholesterol esters to total lipids ratio in chylomicrons and extremely large VLDL (%)	1109	-0.03	-0.06	0.01	0.149	1025	-0.02	-0.06	0.01	0.209	785	-0.02	-0.06	0.02	0.320
Free cholesterol to total lipids ratio in chylomicrons and extremely large VLDL (%)	1109	0.00	-0.04	0.03	0.832	1025	0.00	-0.04	0.04	0.876	785	0.03	-0.02	0.08	0.250
Triglycerides to total lipids ratio in chylomicrons and extremely large VLDL (%)	1109	0.02	-0.02	0.06	0.253	1025	0.02	-0.02	0.06	0.388	785	0.01	-0.04	0.06	0.681
Phospholipids to total lipds ratio in very large VLDL (%)	1109	0.00	-0.04	0.04	0.958	1025	0.01	-0.04	0.05	0.827	785	0.03	-0.02	0.09	0.271
Total cholesterol to total lipids ratio in very large VLDL (%)	1109	0.02	-0.02	0.05	0.326	1025	0.01	-0.02	0.05	0.469	785	0.00	-0.04	0.04	0.919
Cholesterol esters to total lipids ratio in very large VLDL (%)	1109	-0.02	-0.08	0.04	0.488	1025	-0.03	-0.10	0.04	0.415	785	-0.05	-0.14	0.04	0.304
Free cholesterol to total lipids ratio in very large VLDL (%)	1109	-0.01	-0.07	0.05	0.779	1025	-0.01	-0.09	0.06	0.705	785	-0.02	-0.12	0.08	0.680
Triglycerides to total lipids ratio in very large VLDL (%)	1109	-0.02	-0.05	0.01	0.228	1025	-0.02	-0.06	0.02	0.296	785	-0.03	-0.07	0.02	0.214
Phospholipids to total lipds ratio in large VLDL (%)	1109	0.00	-0.05	0.05	0.880	1025	0.02	-0.04	0.07	0.573	785	0.04	-0.03	0.11	0.250
Total cholesterol to total lipids ratio in large VLDL (%)	1109	-0.02	-0.05	0.02	0.325	1025	-0.01	-0.05	0.03	0.557	785	0.01	-0.03	0.05	0.628
Cholesterol esters to total lipids ratio in large VLDL (%)	1109	-0.01	-0.03	0.02	0.676	1025	0.00	-0.03	0.03	0.830	785	0.00	-0.03	0.04	0.867
Free cholesterol to total lipids ratio in large VLDL (%)	1109	-0.01	-0.02	0.00	0.189	1025	-0.01	-0.02	0.01	0.399	785	0.00	-0.02	0.02	0.918
Triglycerides to total lipids ratio in large VLDL (%)	1109	0.01	-0.03	0.05	0.596	1025	0.00	-0.04	0.04	0.960	785	-0.03	-0.08	0.02	0.224
Phospholipids to total lipds ratio in medium VLDL (%)	1109	0.00	-0.03	0.02	0.773	1025	-0.01	-0.04	0.02	0.616	785	0.01	-0.03	0.04	0.711
Total cholesterol to total lipids ratio in medium VLDL (%)	1109	-0.03	-0.06	0.01	0.112	1025	-0.03	-0.07	0.01	0.133	785	-0.01	-0.05	0.03	0.572
Cholesterol esters to total lipids ratio in medium VLDL (%)	1109	-0.03	-0.06	0.01	0.149	1025	-0.03	-0.06	0.01	0.134	785	-0.01	-0.06	0.03	0.466
Free cholesterol to total lipids ratio in medium VLDL (%)	1109	-0.02	-0.06	0.01	0.215	1025	-0.02	-0.06	0.02	0.404	785	-0.01	-0.06	0.05	0.845
Triglycerides to total lipids ratio in medium VLDL (%)	1109	0.03	0.00	0.06	0.087	1025	0.03	0.00	0.06	0.094	785	0.01	-0.03	0.05	0.526
Phospholipids to total lipds ratio in small VLDL (%)	1109	0.01	-0.02	0.05	0.506	1025	0.00	-0.03	0.04	0.859	785	-0.01	-0.05	0.03	0.715
Total cholesterol to total lipids ratio in small VLDL (%)	1109	0.00	-0.04	0.04	0.877	1025	-0.01	-0.05	0.03	0.589	785	0.00	-0.05	0.04	0.911
Cholesterol esters to total lipids ratio in small VLDL (%)	1109	0.00	-0.04	0.04	0.929	1025	-0.01	-0.05	0.03	0.670	785	0.00	-0.05	0.05	0.971
Free cholesterol to total lipids ratio in small VLDL (%)	1109	-0.01	-0.04	0.02	0.461	1025	-0.02	-0.05	0.01	0.207	785	-0.03	-0.06	0.00	0.089
Triglycerides to total lipids ratio in small VLDL (%)	1109	0.00	-0.04	0.04	0.905	1025	0.01	-0.03	0.05	0.682	785	0.00	-0.04	0.05	0.917
Phospholipids to total lipds ratio in very small VLDL (%)	1109	-0.03	-0.07	0.00	0.040	1025	-0.03	-0.07	0.00	0.049	785	-0.03	-0.06	0.00	0.067
Total cholesterol to total lipids ratio in very small VLDL (%)	1109	0.02	-0.01	0.06	0.166	1025	0.02	-0.02	0.05	0.382	785	0.02	-0.02	0.06	0.395
Cholesterol esters to total lipids ratio in very small VLDL (%)	1109	0.02	-0.01	0.05	0.199	1025	0.02	-0.02	0.05	0.378	785	0.02	-0.02	0.06	0.299
Free cholesterol to total lipids ratio in very small VLDL (%)	1109	0.02	-0.02	0.06	0.322	1025	0.01	-0.03	0.06	0.639	785	0.00	-0.05	0.04	0.960
Triglycerides to total lipids ratio in very small VLDL (%)	1109	-0.01	-0.05	0.03	0.663	1025	0.00	-0.04	0.04	0.945	785	0.00	-0.05	0.05	0.938
Phospholipids to total lipds ratio in IDL (%)	1109	-0.01	-0.05	0.03	0.676	1025	-0.02	-0.06	0.03	0.414	785	0.00	-0.05	0.05	0.962
Total cholesterol to total lipids ratio in IDL (%)	1109	0.00	-0.04	0.04	0.924	1025	0.00	-0.04	0.04	0.905	785	-0.01	-0.06	0.03	0.562
Cholesterol esters to total lipids ratio in IDL (%)	1109	0.00	-0.03	0.04	0.841	1025	0.01	-0.03	0.05	0.638	785	0.00	-0.05	0.04	0.922
Free cholesterol to total lipids ratio in IDL (%)	1109	-0.01	-0.06	0.03	0.522	1025	-0.03	-0.07	0.02	0.197	785	-0.03	-0.08	0.02	0.286
Triglycerides to total lipids ratio in IDL (%)	1109	0.01	-0.03	0.04	0.784	1025	0.01	-0.03	0.05	0.628	785	0.02	-0.03	0.06	0.479
Phospholipids to total lipds ratio in large LDL (%)	1109	0.02	-0.01	0.06	0.218	1025	0.03	-0.01	0.07	0.205	785	0.04	0.00	0.08	0.082
Total cholesterol to total lipids ratio in large LDL (%)	1109	-0.02	-0.06	0.02	0.336	1025	-0.02	-0.07	0.02	0.307	785	-0.04	-0.08	0.00	0.048
Cholesterol esters to total lipids ratio in large LDL (%)	1109	-0.02	-0.07	0.02	0.265	1025	-0.02	-0.07	0.02	0.334	785	-0.04	-0.07	0.00	0.038
Free cholesterol to total lipids ratio in large LDL (%)	1109	0.02	-0.02	0.06	0.359	1025	0.01	-0.04	0.05	0.727	785	0.01	-0.04	0.06	0.777
Triglycerides to total lipids ratio in large LDL (%)	1109	0.01	-0.03	0.04	0.797	1025	0.01	-0.04	0.05	0.752	785	0.02	-0.03	0.07	0.410
Phospholipids to total lipds ratio in medium LDL (%)	1109	0.02	-0.02	0.06	0.444	1025	0.02	-0.03	0.06	0.420	785	0.02	-0.02	0.07	0.264
Total cholesterol to total lipids ratio in medium LDL (%)	1109	-0.02	-0.07	0.03	0.400	1025	-0.02	-0.08	0.03	0.369	785	-0.04	-0.10	0.01	0.099
Cholesterol esters to total lipids ratio in medium LDL (%)	1109	-0.02	-0.06	0.02	0.323	1025	-0.02	-0.06	0.02	0.353	785	-0.03	-0.07	0.01	0.099
Free cholesterol to total lipids ratio in medium LDL (%)	1109	0.02	-0.02	0.05	0.375	1025	0.01	-0.03	0.05	0.520	785	0.01	-0.04	0.06	0.589
Triglycerides to total lipids ratio in medium LDL (%)	1109	0.01	-0.04	0.07	0.673	1025	0.01	-0.05	0.08	0.639	785	0.03	-0.05	0.11	0.408
Phospholipids to total lipds ratio in small LDL (%)	1109	0.02	-0.02	0.07	0.263	1025	0.03	-0.02	0.07	0.264	785	0.04	-0.01	0.09	0.082
Total cholesterol to total lipids ratio in small LDL (%)	1109	-0.02	-0.06	0.03	0.469	1025	-0.02	-0.07	0.03	0.391	785	-0.04	-0.09	0.01	0.124
Cholesterol esters to total lipids ratio in small LDL (%)	1109	-0.02	-0.06	0.02	0.324	1025	-0.02	-0.06	0.02	0.325	785	-0.03	-0.07	0.01	0.126
Free cholesterol to total lipids ratio in small LDL (%)	1109	0.02	-0.02	0.06	0.274	1025	0.02	-0.02	0.06	0.416	785	0.01	-0.03	0.06	0.582
Triglycerides to total lipids ratio in small LDL (%)	1109	-0.02	-0.06	0.02	0.366	1025	-0.01	-0.05	0.03	0.632	785	0.00	-0.05	0.05	0.869
Phospholipids to total lipds ratio in very large HDL (%)	1109	-0.02	-0.06	0.01	0.227	1025	-0.03	-0.07	0.01	0.100	785	0.00	-0.04	0.04	0.918
Total cholesterol to total lipids ratio in very large HDL (%)	1109	0.03	-0.01	0.06	0.135	1025	0.04	0.00	0.07	0.062	785	0.00	-0.04	0.04	0.824
Cholesterol esters to total lipids ratio in very large HDL (%)	1109	0.03	-0.01	0.07	0.124	1025	0.04	0.00	0.08	0.059	785	0.01	-0.03	0.05	0.728

S4 Table Observational associations of age at voice breaking (per year later) with adiposity and cardiometabolic traits at age 18y among males in ALSPAC

	Adj. for age, education					*Adj. for age, education, BMI at age 8y*					*Adj. for age, education, outcome value at age 8y*				
**Standardised outcome at age 18y**	**N**	**Beta**	**LCL**	**UCL**	**P-value**	**N**	**Beta**	**LCL**	**UCL**	**P-value**	**N**	**Beta**	**LCL**	**UCL**	**P-value**
Free cholesterol to total lipids ratio in very large HDL (%)	1109	-0.02	-0.06	0.03	0.481	1025	-0.01	-0.06	0.03	0.566	785	-0.03	-0.08	0.02	0.225
Triglycerides to total lipids ratio in very large HDL (%)	1109	-0.02	-0.06	0.02	0.374	1025	-0.01	-0.05	0.03	0.655	785	-0.01	-0.06	0.04	0.676
Phospholipids to total lipds ratio in large HDL (%)	1109	-0.02	-0.06	0.01	0.255	1025	-0.01	-0.05	0.02	0.477	785	-0.02	-0.06	0.02	0.412
Total cholesterol to total lipids ratio in large HDL (%)	1109	0.02	-0.02	0.05	0.310	1025	0.01	-0.03	0.05	0.626	785	0.02	-0.02	0.06	0.343
Cholesterol esters to total lipids ratio in large HDL (%)	1109	0.02	-0.02	0.06	0.282	1025	0.01	-0.03	0.05	0.564	785	0.02	-0.02	0.06	0.290
Free cholesterol to total lipids ratio in large HDL (%)	1109	0.01	-0.03	0.05	0.567	1025	0.00	-0.04	0.04	0.927	785	0.01	-0.04	0.05	0.796
Triglycerides to total lipids ratio in large HDL (%)	1109	-0.01	-0.05	0.02	0.518	1025	0.00	-0.04	0.04	0.931	785	-0.01	-0.06	0.03	0.503
Phospholipids to total lipds ratio in medium HDL (%)	1109	0.00	-0.03	0.03	0.992	1025	0.00	-0.04	0.04	0.891	785	0.00	-0.04	0.04	0.955
Total cholesterol to total lipids ratio in medium HDL (%)	1109	0.01	-0.02	0.05	0.425	1025	0.01	-0.03	0.05	0.543	785	0.01	-0.03	0.05	0.640
Cholesterol esters to total lipids ratio in medium HDL (%)	1109	0.02	-0.02	0.06	0.311	1025	0.02	-0.02	0.06	0.404	785	0.02	-0.03	0.06	0.461
Free cholesterol to total lipids ratio in medium HDL (%)	1109	-0.02	-0.09	0.05	0.529	1025	-0.03	-0.10	0.05	0.511	785	-0.04	-0.14	0.06	0.421
Triglycerides to total lipids ratio in medium HDL (%)	1109	-0.03	-0.07	0.01	0.131	1025	-0.02	-0.06	0.02	0.341	785	-0.02	-0.07	0.02	0.297
Phospholipids to total lipds ratio in small HDL (%)	1109	0.03	0.00	0.06	0.086	1025	0.03	0.00	0.06	0.076	785	0.02	-0.01	0.06	0.228
Total cholesterol to total lipids ratio in small HDL (%)	1109	-0.02	-0.06	0.01	0.158	1025	-0.03	-0.06	0.01	0.102	785	-0.02	-0.06	0.02	0.264
Cholesterol esters to total lipids ratio in small HDL (%)	1109	-0.03	-0.06	0.01	0.113	1025	-0.03	-0.06	0.00	0.085	785	-0.02	-0.06	0.02	0.256
Free cholesterol to total lipids ratio in small HDL (%)	1109	0.03	-0.01	0.06	0.154	1025	0.02	-0.02	0.06	0.310	785	0.01	-0.03	0.06	0.656
Triglycerides to total lipids ratio in small HDL (%)	1109	-0.01	-0.05	0.03	0.709	1025	0.00	-0.04	0.04	0.902	785	0.00	-0.04	0.05	0.847
Mean diameter for VLDL particles (nm)	1109	-0.01	-0.05	0.03	0.690	1025	0.01	-0.03	0.05	0.765	785	0.00	-0.05	0.05	0.902
Mean diameter for LDL particles (nm)	1109	0.01	-0.04	0.05	0.730	1025	0.00	-0.05	0.05	0.965	785	0.02	-0.03	0.08	0.407
Mean diameter for HDL particles (nm)	1109	-0.01	-0.05	0.02	0.531	1025	-0.02	-0.06	0.01	0.222	785	0.01	-0.03	0.04	0.728
Serum total cholesterol (mmol/l)	1109	-0.02	-0.06	0.01	0.122	1025	-0.02	-0.06	0.01	0.138	785	-0.03	-0.06	0.00	0.068
Total cholesterol in VLDL (mmol/l)	1109	-0.01	-0.05	0.02	0.471	1025	0.00	-0.04	0.03	0.830	785	-0.02	-0.06	0.03	0.426
Remnant cholesterol (non-HDL, non-LDL -cholesterol) (mmol/l)	1109	-0.02	-0.05	0.02	0.284	1025	-0.01	-0.05	0.02	0.456	785	-0.02	-0.06	0.01	0.180
Total cholesterol in LDL (mmol/l)	1109	-0.02	-0.06	0.01	0.150	1025	-0.02	-0.06	0.01	0.161	785	-0.03	-0.06	0.00	0.083
Total cholesterol in HDL (mmol/l)	1109	-0.01	-0.04	0.02	0.442	1025	-0.02	-0.05	0.01	0.282	785	-0.01	-0.04	0.02	0.643
Total cholesterol in HDL2 (mmol/l)	1109	-0.01	-0.04	0.02	0.570	1025	-0.02	-0.05	0.02	0.340	785	-0.01	-0.04	0.03	0.760
Total cholesterol in HDL3 (mmol/l)	1109	-0.02	-0.05	0.01	0.272	1025	-0.02	-0.05	0.01	0.217	785	-0.01	-0.04	0.02	0.449
Esterified cholesterol (mmol/l)	1105	-0.02	-0.05	0.01	0.192	1021	-0.02	-0.06	0.01	0.186	781	-0.02	-0.05	0.01	0.113
Free cholesterol (mmol/l)	1104	-0.03	-0.06	0.00	0.076	1020	-0.02	-0.06	0.01	0.135	779	-0.03	-0.06	0.00	0.065
Serum total triglycerides (mmol/l)	1109	-0.01	-0.05	0.03	0.518	1025	0.00	-0.04	0.04	0.964	785	-0.01	-0.06	0.04	0.765
Triglycerides in VLDL (mmol/l)	1109	-0.01	-0.05	0.03	0.665	1025	0.01	-0.04	0.05	0.767	785	-0.01	-0.06	0.05	0.835
Triglycerides in LDL (mmol/l)	1109	-0.02	-0.05	0.01	0.195	1025	-0.02	-0.05	0.01	0.289	785	-0.01	-0.04	0.02	0.573
Triglycerides in HDL (mmol/l)	1109	-0.03	-0.06	0.01	0.099	1025	-0.02	-0.05	0.01	0.250	785	-0.02	-0.06	0.02	0.432
Diacylglycerol (mmol/l)	1085	-0.01	-0.04	0.03	0.757	1007	-0.01	-0.05	0.03	0.650	753	0.00	-0.05	0.04	0.906
Ratio of diacylglycerol to triglycerides	1085	0.01	-0.03	0.04	0.764	1007	-0.01	-0.04	0.03	0.753	753	0.01	-0.03	0.04	0.804
Total phosphoglycerides (mmol/l)	1104	-0.04	-0.06	-0.01	0.011	1020	-0.03	-0.06	0.00	0.025	779	-0.03	-0.06	0.00	0.051
Ratio of triglycerides to phosphoglycerides	1104	0.00	-0.04	0.04	0.846	1020	0.02	-0.03	0.06	0.447	779	0.01	-0.05	0.06	0.747
Phosphatidylcholine and other cholines (mmol/l)	1098	-0.02	-0.05	0.00	0.101	1014	-0.02	-0.05	0.01	0.130	772	-0.03	-0.06	0.01	0.107
Total cholines (mmol/l)	1105	-0.03	-0.06	-0.01	0.020	1021	-0.03	-0.06	0.00	0.030	781	-0.03	-0.06	0.00	0.039
Apolipoprotein A-I (g/l)	1109	-0.02	-0.05	0.01	0.180	1025	-0.02	-0.05	0.01	0.161	785	-0.02	-0.04	0.01	0.260
Apolipoprotein B (g/l)	1109	-0.02	-0.06	0.01	0.239	1025	-0.01	-0.05	0.02	0.476	785	-0.02	-0.06	0.02	0.253
Ratio of apolipoprotein B to apolipoprotein A-I	1109	-0.01	-0.05	0.02	0.474	1025	0.00	-0.04	0.04	0.823	785	-0.02	-0.06	0.02	0.404
Total fatty acids (mmol/l)	1105	-0.02	-0.05	0.01	0.210	1021	-0.01	-0.05	0.02	0.490	781	-0.02	-0.06	0.02	0.370
Estimated description of fatty acid chain length, not actual carbon number	1105	0.04	0.00	0.08	0.045	1021	0.05	0.00	0.09	0.038	780	0.04	-0.01	0.09	0.098
Estimated degree of unsaturation	1104	0.03	-0.02	0.07	0.236	1020	0.03	-0.02	0.07	0.250	780	0.03	-0.02	0.09	0.261
22:6, docosahexaenoic acid (mmol/l)	1105	0.00	-0.03	0.03	0.854	1021	0.01	-0.02	0.04	0.561	781	0.01	-0.03	0.04	0.759
18:2, linoleic acid (mmol/l)	1105	-0.02	-0.05	0.01	0.212	1021	-0.02	-0.05	0.02	0.355	781	-0.02	-0.05	0.02	0.334
Conjugated linoleic acid (mmol/l)	1104	-0.02	-0.05	0.02	0.338	1020	-0.01	-0.05	0.03	0.545	779	-0.02	-0.07	0.02	0.358
Omega-3 fatty acids (mmol/l)	1105	0.00	-0.04	0.03	0.837	1021	0.01	-0.03	0.04	0.723	781	0.00	-0.05	0.04	0.869
Omega-6 fatty acids (mmol/l)	1105	-0.02	-0.05	0.01	0.170	1021	-0.02	-0.05	0.02	0.332	781	-0.02	-0.05	0.02	0.372
Polyunsaturated fatty acids (mmol/l)	1105	-0.02	-0.05	0.01	0.208	1021	-0.01	-0.05	0.02	0.417	781	-0.01	-0.05	0.02	0.405
Monounsaturated fatty acids; 16:1, 18:1 (mmol/l)	1105	-0.02	-0.05	0.02	0.353	1021	-0.01	-0.04	0.03	0.733	780	-0.01	-0.06	0.03	0.555

S4 Table Observational associations of age at voice breaking (per year later) with adiposity and cardiometabolic traits at age 18y among males in ALSPAC

	Adj. for age, education					*Adj. for age, education, BMI at age 8y*					*Adj. for age, education, outcome value at age 8y*				
**Standardised outcome at age 18y**	**N**	**Beta**	**LCL**	**UCL**	**P-value**	**N**	**Beta**	**LCL**	**UCL**	**P-value**	**N**	**Beta**	**LCL**	**UCL**	**P-value**
Saturated fatty acids (mmol/l)	1105	-0.02	-0.06	0.01	0.235	1021	-0.01	-0.05	0.02	0.479	780	-0.02	-0.07	0.02	0.297
Ratio of 22:6 docosahexaenoic acid to total fatty acids (%)	1105	0.02	-0.02	0.06	0.275	1021	0.02	-0.01	0.06	0.221	781	0.03	-0.02	0.07	0.217
Ratio of 18:2 linoleic acid to total fatty acids (%)	1105	0.00	-0.04	0.04	0.912	1021	-0.01	-0.05	0.04	0.801	781	0.00	-0.05	0.05	0.924
Ratio of conjugated linoleic acid to total fatty acids (%)	1104	-0.01	-0.04	0.03	0.708	1020	0.00	-0.04	0.03	0.861	779	-0.01	-0.05	0.03	0.567
Ratio of omega-3 fatty acids to total fatty acids (%)	1105	0.01	-0.03	0.05	0.507	1021	0.02	-0.02	0.06	0.339	781	0.02	-0.03	0.06	0.492
Ratio of omega-6 fatty acids to total fatty acids (%)	1105	0.00	-0.04	0.05	0.899	1021	0.00	-0.05	0.04	0.855	781	0.01	-0.04	0.06	0.683
Ratio of polyunsaturated fatty acids to total fatty acids (%)	1105	0.01	-0.04	0.05	0.790	1021	0.00	-0.04	0.05	0.969	781	0.02	-0.04	0.07	0.574
Ratio of monounsaturated fatty acids to total fatty acids (%)	1105	0.00	-0.04	0.04	0.965	1021	0.01	-0.03	0.05	0.766	780	0.00	-0.04	0.05	0.879
Ratio of saturated fatty acids to total fatty acids (%)	1105	-0.01	-0.05	0.04	0.790	1021	-0.01	-0.06	0.04	0.688	780	-0.02	-0.07	0.02	0.323
Glucose (mmol/l)	1109	0.01	-0.03	0.06	0.541	1025	0.01	-0.04	0.06	0.641	780	-0.01	-0.04	0.02	0.451
Lactate (mmol/l)	1109	-0.03	-0.08	0.01	0.175	1025	-0.02	-0.07	0.02	0.324	786	-0.01	-0.06	0.03	0.622
Pyruvate (mmol/l)	1109	-0.03	-0.07	0.02	0.231	1025	-0.01	-0.05	0.03	0.567	785	-0.01	-0.06	0.03	0.624
Citrate (mmol/l)	1109	0.04	0.01	0.08	0.026	1025	0.03	-0.01	0.07	0.184	785	0.04	-0.01	0.09	0.091
Alanine (mmol/l)	1109	-0.03	-0.06	0.01	0.137	1025	-0.02	-0.06	0.02	0.247	786	-0.01	-0.06	0.03	0.521
Glutamine (mmol/l)	1109	0.01	-0.02	0.04	0.491	1025	0.01	-0.02	0.04	0.557	785	0.02	-0.02	0.06	0.285
Histidine (mmol/l)	1109	-0.01	-0.05	0.02	0.419	1025	0.00	-0.04	0.03	0.871	785	0.00	-0.05	0.04	0.823
Isoleucine (mmol/l)	1109	0.00	-0.04	0.04	0.920	1025	0.02	-0.02	0.06	0.376	786	0.03	-0.02	0.07	0.254
Leucine (mmol/l)	1109	0.01	-0.03	0.04	0.701	1025	0.02	-0.02	0.06	0.354	786	0.01	-0.03	0.06	0.500
Valine (mmol/l)	1109	0.01	-0.03	0.05	0.570	1025	0.02	-0.02	0.06	0.242	786	0.03	-0.02	0.07	0.209
Phenylalanine (mmol/l)	1109	-0.02	-0.06	0.02	0.340	1025	-0.02	-0.06	0.02	0.344	783	-0.02	-0.06	0.03	0.437
Tyrosine (mmol/l)	1109	-0.02	-0.05	0.01	0.259	1025	-0.02	-0.05	0.02	0.308	782	0.00	-0.04	0.03	0.795
Acetate (mmol/l)	1109	-0.02	-0.05	0.01	0.273	1025	-0.03	-0.06	0.01	0.160	786	0.00	-0.02	0.02	0.983
Acetoacetate (mmol/l)	1109	0.02	-0.02	0.06	0.281	1025	0.03	-0.01	0.07	0.166	786	0.01	-0.04	0.06	0.669
3-hydroxybutyrate (mmol/l)	1109	0.04	0.01	0.07	0.017	1025	0.04	0.01	0.08	0.015	786	0.04	0.00	0.08	0.072
Creatinine (mmol/l)	1109	-0.04	-0.07	0.00	0.040	1025	-0.03	-0.06	0.01	0.102	784	-0.04	-0.07	0.00	0.051
Albumin (signal area)	1109	-0.01	-0.04	0.03	0.755	1025	0.00	-0.04	0.04	0.986	785	0.01	-0.04	0.05	0.739
Glycoprotein acetyls, mainly a1-acid glycoprotein (mmol/l)	1109	-0.01	-0.04	0.03	0.712	1025	0.01	-0.02	0.05	0.514	786	0.02	-0.02	0.07	0.342

Complete case sample

	Adj. for age, education					*Adj. for age, education, BMI at age 8y*					*Adj. for age, education, outcome value at age 8y*				
**Standardised outcome at age 18y**	**N**	**Beta**	**LCL**	**UCL**	**P-value**	**N**	**Beta**	**LCL**	**UCL**	**P-value**	**N**	**Beta**	**LCL**	**UCL**	**P-value**
Body mass index (kg/m^2)	605	-0.04	-0.08	0.00	0.034	605	-0.03	-0.06	0.00	0.093	605	-0.03	-0.06	0.00	0.093
Fat mass index (kg/m^2)	605	-0.03	-0.07	0.00	0.087	605	-0.02	-0.05	0.01	0.201	605	0.00	-0.03	0.03	0.993
Lean mass index (kg/m^2)	605	-0.02	-0.05	0.01	0.176	605	-0.02	-0.05	0.02	0.349	605	-0.02	-0.05	0.00	0.109
Systolic blood pressure (mmHg)	605	-0.05	-0.09	0.00	0.049	605	-0.04	-0.09	0.00	0.076	605	-0.03	-0.07	0.01	0.179
Diastolic blood pressure (mmHg)	605	-0.05	-0.10	0.00	0.035	605	-0.05	-0.09	0.00	0.050	605	-0.04	-0.09	0.01	0.081
Concentration of chylomicrons and extremely large VLDL particles (mol/l)	605	0.00	-0.06	0.06	0.915	605	0.01	-0.05	0.07	0.768	605	0.01	-0.05	0.07	0.839
Total lipids in chylomicrons and extremely large VLDL (mmol/l)	605	0.00	-0.06	0.06	0.943	605	0.01	-0.05	0.07	0.795	605	0.01	-0.05	0.06	0.862
Phospholipids in chylomicrons and extremely large VLDL (mmol/l)	605	0.00	-0.06	0.06	0.901	605	0.01	-0.05	0.07	0.753	605	0.01	-0.05	0.07	0.827
Total cholesterol in chylomicrons and extremely large VLDL (mmol/l)	605	0.00	-0.06	0.06	0.898	605	0.00	-0.06	0.06	0.952	605	0.00	-0.06	0.06	0.979
Cholesterol esters in chylomicrons and extremely large VLDL (mmol/l)	605	-0.01	-0.07	0.05	0.744	605	0.00	-0.06	0.06	0.889	605	-0.01	-0.07	0.05	0.804
Free cholesterol in chylomicrons and extremely large VLDL (mmol/l)	605	0.00	-0.06	0.06	0.910	605	0.01	-0.05	0.07	0.762	605	0.01	-0.05	0.07	0.830
Triglycerides in chylomicrons and extremely large VLDL (mmol/l)	605	0.00	-0.06	0.06	0.908	605	0.01	-0.05	0.07	0.761	605	0.01	-0.05	0.07	0.827
Concentration of very large VLDL particles (mol/l)	605	0.00	-0.06	0.06	0.953	605	0.01	-0.05	0.07	0.800	605	0.00	-0.05	0.06	0.871
Total lipids in very large VLDL (mmol/l)	605	0.00	-0.06	0.06	0.992	605	0.01	-0.05	0.07	0.838	605	0.00	-0.06	0.06	0.912
Phospholipids in very large VLDL (mmol/l)	605	0.00	-0.06	0.06	0.966	605	0.01	-0.05	0.07	0.815	605	0.00	-0.06	0.06	0.906
Total cholesterol in very large VLDL (mmol/l)	605	0.00	-0.06	0.06	0.978	605	0.00	-0.05	0.06	0.870	605	0.00	-0.06	0.06	0.934
Cholesterol esters in very large VLDL (mmol/l)	605	0.00	-0.06	0.06	0.934	605	0.00	-0.06	0.06	0.913	605	0.00	-0.06	0.06	0.965
Free cholesterol in very large VLDL (mmol/l)	605	0.00	-0.06	0.06	0.975	605	0.01	-0.05	0.07	0.825	605	0.00	-0.06	0.06	0.901
Triglycerides in very large VLDL (mmol/l)	605	0.00	-0.06	0.06	0.990	605	0.01	-0.05	0.07	0.835	605	0.00	-0.06	0.06	0.906

S4 Table Observational associations of age at voice breaking (per year later) with adiposity and cardiometabolic traits at age 18y among males in ALSPAC

	Adj. for age, education					*Adj. for age, education, BMI at age 8y*					*Adj. for age, education, outcome value at age 8y*				
**Standardised outcome at age 18y**	**N**	**Beta**	**LCL**	**UCL**	**P-value**	**N**	**Beta**	**LCL**	**UCL**	**P-value**	**N**	**Beta**	**LCL**	**UCL**	**P-value**
Concentration of large VLDL particles (mol/l)	605	0.00	-0.06	0.05	0.890	605	0.00	-0.06	0.06	0.949	605	0.00	-0.06	0.06	0.985
Total lipids in large VLDL (mmol/l)	605	0.00	-0.06	0.06	0.927	605	0.00	-0.05	0.06	0.913	605	0.00	-0.06	0.06	0.979
Phospholipids in large VLDL (mmol/l)	605	0.01	-0.05	0.07	0.818	605	0.01	-0.05	0.07	0.674	605	0.01	-0.05	0.07	0.743
Total cholesterol in large VLDL (mmol/l)	605	-0.01	-0.06	0.05	0.857	605	0.00	-0.06	0.06	0.985	605	0.00	-0.06	0.06	0.944
Cholesterol esters in large VLDL (mmol/l)	605	-0.01	-0.07	0.05	0.789	605	0.00	-0.06	0.06	0.943	605	0.00	-0.06	0.05	0.875
Free cholesterol in large VLDL (mmol/l)	605	0.00	-0.06	0.06	0.926	605	0.00	-0.05	0.06	0.916	605	0.00	-0.06	0.06	0.994
Triglycerides in large VLDL (mmol/l)	605	0.00	-0.06	0.05	0.876	605	0.00	-0.06	0.06	0.964	605	0.00	-0.06	0.06	0.974
Concentration of medium VLDL particles (mol/l)	605	-0.01	-0.06	0.05	0.805	605	0.00	-0.06	0.05	0.964	605	0.00	-0.06	0.05	0.904
Total lipids in medium VLDL (mmol/l)	605	-0.01	-0.06	0.05	0.778	605	0.00	-0.06	0.05	0.935	605	0.00	-0.06	0.05	0.872
Phospholipids in medium VLDL (mmol/l)	605	-0.01	-0.06	0.05	0.778	605	0.00	-0.06	0.05	0.935	605	0.00	-0.06	0.05	0.862
Total cholesterol in medium VLDL (mmol/l)	605	-0.01	-0.07	0.04	0.628	605	-0.01	-0.06	0.05	0.769	605	-0.01	-0.06	0.04	0.693
Cholesterol esters in medium VLDL (mmol/l)	605	-0.02	-0.07	0.04	0.567	605	-0.01	-0.07	0.04	0.692	605	-0.01	-0.06	0.04	0.594
Free cholesterol in medium VLDL (mmol/l)	605	-0.01	-0.07	0.05	0.716	605	0.00	-0.06	0.05	0.868	605	-0.01	-0.06	0.05	0.798
Triglycerides in medium VLDL (mmol/l)	605	-0.01	-0.06	0.05	0.856	605	0.00	-0.06	0.06	0.982	605	0.00	-0.06	0.05	0.960
Concentration of small VLDL particles (mol/l)	605	-0.01	-0.06	0.04	0.740	605	0.00	-0.06	0.05	0.889	605	-0.01	-0.06	0.04	0.659
Total lipids in small VLDL (mmol/l)	605	-0.01	-0.06	0.04	0.711	605	0.00	-0.06	0.05	0.853	605	-0.01	-0.06	0.03	0.562
Phospholipids in small VLDL (mmol/l)	605	-0.01	-0.06	0.04	0.758	605	0.00	-0.05	0.05	0.903	605	-0.02	-0.06	0.03	0.480
Total cholesterol in small VLDL (mmol/l)	605	-0.01	-0.06	0.04	0.608	605	-0.01	-0.06	0.04	0.714	605	-0.02	-0.07	0.02	0.279
Cholesterol esters in small VLDL (mmol/l)	605	-0.01	-0.07	0.04	0.612	605	-0.01	-0.06	0.04	0.701	605	-0.02	-0.07	0.02	0.267
Free cholesterol in small VLDL (mmol/l)	605	-0.01	-0.06	0.04	0.644	605	-0.01	-0.06	0.04	0.776	605	-0.02	-0.06	0.03	0.402
Triglycerides in small VLDL (mmol/l)	605	-0.01	-0.06	0.05	0.824	605	0.00	-0.05	0.05	0.978	605	-0.01	-0.06	0.05	0.828
Concentration of very small VLDL particles (mol/l)	605	-0.02	-0.06	0.03	0.496	605	-0.01	-0.06	0.03	0.567	605	-0.03	-0.06	0.01	0.149
Total lipids in very small VLDL (mmol/l)	605	-0.01	-0.06	0.03	0.553	605	-0.01	-0.06	0.04	0.619	605	-0.03	-0.07	0.01	0.167
Phospholipids in very small VLDL (mmol/l)	605	-0.02	-0.07	0.02	0.365	605	-0.02	-0.06	0.03	0.407	605	-0.03	-0.07	0.00	0.068
Total cholesterol in very small VLDL (mmol/l)	605	-0.01	-0.06	0.04	0.647	605	-0.01	-0.06	0.04	0.687	605	-0.02	-0.07	0.02	0.351
Cholesterol esters in very small VLDL (mmol/l)	605	-0.01	-0.06	0.05	0.763	605	-0.01	-0.06	0.05	0.807	605	-0.02	-0.06	0.03	0.458
Free cholesterol in very small VLDL (mmol/l)	605	-0.02	-0.07	0.03	0.400	605	-0.02	-0.07	0.03	0.425	605	-0.03	-0.07	0.01	0.197
Triglycerides in very small VLDL (mmol/l)	605	0.00	-0.05	0.04	0.847	605	0.00	-0.05	0.05	0.982	605	-0.01	-0.05	0.04	0.713
Concentration of IDL particles (mol/l)	605	-0.02	-0.07	0.02	0.331	605	-0.02	-0.07	0.02	0.359	605	-0.03	-0.07	0.00	0.079
Total lipids in IDL (mmol/l)	605	-0.02	-0.07	0.02	0.322	605	-0.02	-0.07	0.02	0.347	605	-0.03	-0.07	0.00	0.059
Phospholipids in IDL (mmol/l)	605	-0.02	-0.07	0.02	0.327	605	-0.02	-0.07	0.02	0.344	605	-0.03	-0.07	0.00	0.059
Total cholesterol in IDL (mmol/l)	605	-0.03	-0.07	0.02	0.286	605	-0.03	-0.07	0.02	0.307	605	-0.04	-0.07	0.00	0.053
Cholesterol esters in IDL (mmol/l)	605	-0.03	-0.08	0.02	0.287	605	-0.03	-0.07	0.02	0.314	605	-0.03	-0.07	0.00	0.067
Free cholesterol in IDL (mmol/l)	605	-0.02	-0.07	0.02	0.304	605	-0.02	-0.07	0.02	0.308	605	-0.04	-0.07	0.00	0.047
Triglycerides in IDL (mmol/l)	605	0.00	-0.04	0.04	0.959	605	0.00	-0.04	0.04	0.961	605	0.00	-0.04	0.03	0.834
Concentration of large LDL particles (mol/l)	605	-0.02	-0.07	0.02	0.293	605	-0.02	-0.07	0.02	0.322	605	-0.03	-0.07	0.00	0.052
Total lipids in large LDL (mmol/l)	605	-0.02	-0.07	0.02	0.310	605	-0.02	-0.07	0.02	0.340	605	-0.03	-0.07	0.00	0.057
Phospholipids in large LDL (mmol/l)	605	-0.02	-0.07	0.02	0.294	605	-0.02	-0.07	0.02	0.326	605	-0.04	-0.07	0.00	0.045
Total cholesterol in large LDL (mmol/l)	605	-0.02	-0.07	0.02	0.285	605	-0.02	-0.07	0.02	0.309	605	-0.04	-0.07	0.00	0.043
Cholesterol esters in large LDL (mmol/l)	605	-0.03	-0.07	0.02	0.281	605	-0.02	-0.07	0.02	0.309	605	-0.04	-0.07	0.00	0.045
Free cholesterol in large LDL (mmol/l)	605	-0.02	-0.07	0.02	0.308	605	-0.02	-0.07	0.02	0.318	605	-0.04	-0.07	0.00	0.048
Triglycerides in large LDL (mmol/l)	605	0.00	-0.04	0.04	0.952	605	0.00	-0.04	0.04	0.978	605	0.00	-0.04	0.03	0.859
Concentration of medium LDL particles (mol/l)	605	-0.02	-0.07	0.02	0.280	605	-0.02	-0.07	0.02	0.316	605	-0.04	-0.07	0.00	0.056
Total lipids in medium LDL (mmol/l)	605	-0.02	-0.07	0.02	0.317	605	-0.02	-0.07	0.02	0.353	605	-0.03	-0.07	0.00	0.054
Phospholipids in medium LDL (mmol/l)	605	-0.02	-0.07	0.02	0.297	605	-0.02	-0.07	0.02	0.346	605	-0.03	-0.07	0.00	0.076
Total cholesterol in medium LDL (mmol/l)	605	-0.02	-0.07	0.02	0.288	605	-0.02	-0.07	0.02	0.316	605	-0.04	-0.07	0.00	0.035
Cholesterol esters in medium LDL (mmol/l)	605	-0.02	-0.07	0.02	0.310	605	-0.02	-0.07	0.02	0.340	605	-0.04	-0.07	0.00	0.041
Free cholesterol in medium LDL (mmol/l)	605	-0.03	-0.08	0.02	0.225	605	-0.03	-0.08	0.02	0.245	605	-0.04	-0.08	0.00	0.030
Triglycerides in medium LDL (mmol/l)	605	0.00	-0.04	0.04	0.849	605	0.01	-0.03	0.04	0.793	605	0.00	-0.04	0.04	0.957
Concentration of small LDL particles (mol/l)	605	-0.02	-0.07	0.02	0.287	605	-0.02	-0.07	0.02	0.325	605	-0.04	-0.07	0.00	0.059
Total lipids in small LDL (mmol/l)	605	-0.02	-0.07	0.02	0.292	605	-0.02	-0.07	0.02	0.327	605	-0.04	-0.07	0.00	0.042
Phospholipids in small LDL (mmol/l)	605	-0.03	-0.07	0.02	0.280	605	-0.02	-0.07	0.02	0.325	605	-0.03	-0.07	0.00	0.071

S4 Table Observational associations of age at voice breaking (per year later) with adiposity and cardiometabolic traits at age 18y among males in ALSPAC

	Adj. for age, education					*Adj. for age, education, BMI at age 8y*					*Adj. for age, education, outcome value at age 8y*				
**Standardised outcome at age 18y**	**N**	**Beta**	**LCL**	**UCL**	**P-value**	**N**	**Beta**	**LCL**	**UCL**	**P-value**	**N**	**Beta**	**LCL**	**UCL**	**P-value**
Total cholesterol in small LDL (mmol/l)	605	-0.02	-0.07	0.02	0.290	605	-0.02	-0.07	0.02	0.315	605	-0.04	-0.07	0.00	0.032
Cholesterol esters in small LDL (mmol/l)	605	-0.02	-0.07	0.02	0.317	605	-0.02	-0.07	0.02	0.344	605	-0.04	-0.07	0.00	0.046
Free cholesterol in small LDL (mmol/l)	605	-0.03	-0.08	0.02	0.219	605	-0.03	-0.08	0.02	0.238	605	-0.04	-0.08	0.00	0.033
Triglycerides in small LDL (mmol/l)	605	-0.01	-0.05	0.03	0.704	605	0.00	-0.05	0.04	0.815	605	-0.01	-0.05	0.03	0.618
Concentration of very large HDL particles (mol/l)	605	-0.01	-0.05	0.04	0.746	605	-0.01	-0.05	0.04	0.670	605	0.00	-0.03	0.04	0.842
Total lipids in very large HDL (mmol/l)	605	-0.01	-0.05	0.04	0.785	605	-0.01	-0.05	0.04	0.711	605	0.00	-0.03	0.04	0.804
Phospholipids in very large HDL (mmol/l)	605	-0.01	-0.05	0.04	0.778	605	-0.01	-0.05	0.04	0.690	605	0.00	-0.03	0.04	0.797
Total cholesterol in very large HDL (mmol/l)	605	-0.01	-0.05	0.04	0.829	605	-0.01	-0.06	0.04	0.770	605	0.00	-0.04	0.04	0.876
Cholesterol esters in very large HDL (mmol/l)	605	0.00	-0.05	0.04	0.859	605	-0.01	-0.05	0.04	0.808	605	0.00	-0.04	0.05	0.889
Free cholesterol in very large HDL (mmol/l)	605	-0.01	-0.05	0.04	0.749	605	-0.01	-0.06	0.04	0.675	605	0.00	-0.03	0.04	0.852
Triglycerides in very large HDL (mmol/l)	605	-0.01	-0.05	0.04	0.810	605	0.00	-0.05	0.04	0.868	605	0.00	-0.04	0.05	0.891
Concentration of large HDL particles (mol/l)	605	-0.01	-0.06	0.03	0.651	605	-0.01	-0.06	0.03	0.574	605	0.00	-0.04	0.03	0.810
Total lipids in large HDL (mmol/l)	605	-0.01	-0.05	0.04	0.712	605	-0.01	-0.06	0.03	0.627	605	0.00	-0.04	0.03	0.940
Phospholipids in large HDL (mmol/l)	605	-0.01	-0.05	0.04	0.699	605	-0.01	-0.06	0.03	0.626	605	0.00	-0.04	0.03	0.828
Total cholesterol in large HDL (mmol/l)	605	-0.01	-0.05	0.04	0.737	605	-0.01	-0.06	0.04	0.641	605	0.00	-0.04	0.04	0.952
Cholesterol esters in large HDL (mmol/l)	605	-0.01	-0.05	0.04	0.746	605	-0.01	-0.06	0.04	0.649	605	0.00	-0.04	0.04	0.941
Free cholesterol in large HDL (mmol/l)	605	-0.01	-0.06	0.04	0.711	605	-0.01	-0.06	0.03	0.615	605	0.00	-0.04	0.04	0.995
Triglycerides in large HDL (mmol/l)	605	-0.01	-0.05	0.04	0.805	605	0.00	-0.05	0.04	0.830	605	0.00	-0.04	0.04	0.885
Concentration of medium HDL particles (mol/l)	605	-0.01	-0.05	0.03	0.494	605	-0.01	-0.05	0.03	0.512	605	-0.02	-0.06	0.02	0.354
Total lipids in medium HDL (mmol/l)	605	-0.01	-0.05	0.03	0.577	605	-0.01	-0.05	0.03	0.580	605	-0.02	-0.05	0.02	0.429
Phospholipids in medium HDL (mmol/l)	605	-0.01	-0.05	0.03	0.618	605	-0.01	-0.05	0.03	0.631	605	-0.01	-0.05	0.02	0.467
Total cholesterol in medium HDL (mmol/l)	605	-0.01	-0.06	0.03	0.606	605	-0.01	-0.06	0.03	0.579	605	-0.01	-0.06	0.03	0.500
Cholesterol esters in medium HDL (mmol/l)	605	-0.01	-0.06	0.03	0.587	605	-0.01	-0.06	0.03	0.558	605	-0.02	-0.06	0.03	0.480
Free cholesterol in medium HDL (mmol/l)	605	-0.01	-0.05	0.03	0.672	605	-0.01	-0.05	0.03	0.666	605	-0.01	-0.05	0.03	0.575
Triglycerides in medium HDL (mmol/l)	605	-0.01	-0.05	0.04	0.738	605	0.00	-0.05	0.04	0.886	605	-0.02	-0.06	0.03	0.445
Concentration of small HDL particles (mol/l)	605	0.00	-0.04	0.04	0.860	605	0.00	-0.04	0.04	0.952	605	-0.01	-0.05	0.03	0.559
Total lipids in small HDL (mmol/l)	605	-0.01	-0.05	0.03	0.613	605	-0.01	-0.05	0.03	0.671	605	-0.02	-0.06	0.02	0.361
Phospholipids in small HDL (mmol/l)	605	0.00	-0.04	0.05	0.874	605	0.01	-0.04	0.05	0.803	605	0.00	-0.05	0.04	0.848
Total cholesterol in small HDL (mmol/l)	605	-0.02	-0.06	0.01	0.230	605	-0.02	-0.06	0.02	0.237	605	-0.03	-0.07	0.01	0.109
Cholesterol esters in small HDL (mmol/l)	605	-0.03	-0.06	0.01	0.196	605	-0.03	-0.06	0.01	0.201	605	-0.03	-0.07	0.01	0.097
Free cholesterol in small HDL (mmol/l)	605	-0.01	-0.05	0.03	0.662	605	-0.01	-0.05	0.03	0.679	605	-0.02	-0.06	0.02	0.404
Triglycerides in small HDL (mmol/l)	605	0.00	-0.04	0.05	0.854	605	0.01	-0.04	0.06	0.697	605	0.00	-0.04	0.05	0.902
Phospholipids to total lipids ratio in chylomicrons and extremely large VLDL (%)	605	0.06	-0.06	0.18	0.312	605	0.06	-0.05	0.18	0.295	605	0.06	-0.05	0.18	0.283
Total cholesterol to total lipids ratio in chylomicrons and extremely large VLDL (%)	605	-0.03	-0.08	0.03	0.313	605	-0.03	-0.08	0.03	0.329	605	-0.02	-0.07	0.03	0.385
Cholesterol esters to total lipids ratio in chylomicrons and extremely large VLDL (%)	605	-0.03	-0.08	0.02	0.240	605	-0.03	-0.08	0.02	0.236	605	-0.03	-0.08	0.02	0.225
Free cholesterol to total lipids ratio in chylomicrons and extremely large VLDL (%)	605	0.02	-0.04	0.07	0.524	605	0.02	-0.03	0.08	0.436	605	0.03	-0.03	0.08	0.317
Triglycerides to total lipids ratio in chylomicrons and extremely large VLDL (%)	605	0.02	-0.04	0.09	0.487	605	0.02	-0.05	0.09	0.531	605	0.02	-0.05	0.08	0.594
Phospholipids to total lipds ratio in very large VLDL (%)	605	0.03	-0.04	0.10	0.456	605	0.03	-0.04	0.10	0.394	605	0.03	-0.03	0.10	0.307
Total cholesterol to total lipids ratio in very large VLDL (%)	605	0.03	-0.01	0.08	0.185	605	0.03	-0.02	0.07	0.212	605	0.03	-0.02	0.07	0.247
Cholesterol esters to total lipids ratio in very large VLDL (%)	605	0.01	-0.03	0.04	0.633	605	0.01	-0.03	0.04	0.710	605	0.00	-0.03	0.04	0.791
Free cholesterol to total lipids ratio in very large VLDL (%)	605	0.04	-0.02	0.10	0.179	605	0.04	-0.02	0.10	0.182	605	0.04	-0.02	0.10	0.196
Triglycerides to total lipids ratio in very large VLDL (%)	605	-0.05	-0.10	0.01	0.082	605	-0.05	-0.10	0.01	0.090	605	-0.04	-0.10	0.01	0.102
Phospholipids to total lipds ratio in large VLDL (%)	605	0.03	-0.06	0.12	0.554	605	0.03	-0.06	0.12	0.518	605	0.04	-0.05	0.12	0.399
Total cholesterol to total lipids ratio in large VLDL (%)	605	-0.01	-0.06	0.04	0.780	605	0.00	-0.05	0.04	0.845	605	0.01	-0.04	0.05	0.818
Cholesterol esters to total lipids ratio in large VLDL (%)	605	0.00	-0.04	0.04	0.910	605	0.00	-0.04	0.04	0.904	605	0.01	-0.03	0.05	0.765
Free cholesterol to total lipids ratio in large VLDL (%)	605	-0.01	-0.03	0.01	0.553	605	0.00	-0.03	0.02	0.645	605	0.00	-0.02	0.02	0.864
Triglycerides to total lipids ratio in large VLDL (%)	605	-0.01	-0.06	0.05	0.826	605	-0.01	-0.06	0.05	0.754	605	-0.02	-0.07	0.03	0.420
Phospholipids to total lipds ratio in medium VLDL (%)	605	0.01	-0.03	0.05	0.663	605	0.01	-0.03	0.05	0.706	605	0.01	-0.03	0.04	0.737
Total cholesterol to total lipids ratio in medium VLDL (%)	605	-0.03	-0.08	0.02	0.272	605	-0.03	-0.08	0.02	0.283	605	-0.02	-0.07	0.02	0.357
Cholesterol esters to total lipids ratio in medium VLDL (%)	605	-0.02	-0.07	0.03	0.413	605	-0.02	-0.07	0.03	0.409	605	-0.02	-0.06	0.03	0.473
Free cholesterol to total lipids ratio in medium VLDL (%)	605	-0.04	-0.10	0.02	0.155	605	-0.04	-0.10	0.02	0.183	605	-0.03	-0.08	0.02	0.221
Triglycerides to total lipids ratio in medium VLDL (%)	605	0.02	-0.02	0.07	0.336	605	0.02	-0.02	0.07	0.342	605	0.02	-0.02	0.06	0.392

S4 Table Observational associations of age at voice breaking (per year later) with adiposity and cardiometabolic traits at age 18y among males in ALSPAC

	Adj. for age, education					*Adj. for age, education, BMI at age 8y*					*Adj. for age, education, outcome value at age 8y*				
**Standardised outcome at age 18y**	**N**	**Beta**	**LCL**	**UCL**	**P-value**	**N**	**Beta**	**LCL**	**UCL**	**P-value**	**N**	**Beta**	**LCL**	**UCL**	**P-value**
Phospholipids to total lipds ratio in small VLDL (%)	605	0.02	-0.03	0.06	0.536	605	0.01	-0.04	0.06	0.610	605	0.00	-0.05	0.05	0.997
Total cholesterol to total lipids ratio in small VLDL (%)	605	0.00	-0.06	0.05	0.868	605	-0.01	-0.06	0.05	0.813	605	-0.01	-0.06	0.04	0.718
Cholesterol esters to total lipids ratio in small VLDL (%)	605	0.00	-0.06	0.05	0.932	605	0.00	-0.06	0.05	0.883	605	-0.01	-0.06	0.05	0.827
Free cholesterol to total lipids ratio in small VLDL (%)	605	-0.01	-0.06	0.03	0.528	605	-0.02	-0.06	0.03	0.461	605	-0.03	-0.07	0.01	0.194
Triglycerides to total lipids ratio in small VLDL (%)	605	0.00	-0.05	0.05	1.000	605	0.00	-0.05	0.06	0.917	605	0.01	-0.05	0.06	0.800
Phospholipids to total lipds ratio in very small VLDL (%)	605	-0.03	-0.07	0.02	0.241	605	-0.03	-0.07	0.02	0.245	605	-0.03	-0.07	0.01	0.126
Total cholesterol to total lipids ratio in very small VLDL (%)	605	0.01	-0.04	0.06	0.727	605	0.01	-0.04	0.05	0.790	605	0.01	-0.04	0.05	0.742
Cholesterol esters to total lipids ratio in very small VLDL (%)	605	0.01	-0.03	0.06	0.550	605	0.01	-0.03	0.06	0.585	605	0.01	-0.03	0.06	0.552
Free cholesterol to total lipids ratio in very small VLDL (%)	605	-0.02	-0.06	0.03	0.459	605	-0.02	-0.06	0.02	0.375	605	-0.02	-0.06	0.03	0.450
Triglycerides to total lipids ratio in very small VLDL (%)	605	0.01	-0.04	0.06	0.750	605	0.01	-0.04	0.06	0.681	605	0.01	-0.04	0.06	0.701
Phospholipids to total lipds ratio in IDL (%)	605	0.02	-0.04	0.08	0.513	605	0.02	-0.04	0.07	0.580	605	0.01	-0.04	0.07	0.630
Total cholesterol to total lipids ratio in IDL (%)	605	-0.03	-0.09	0.02	0.253	605	-0.03	-0.09	0.02	0.256	605	-0.03	-0.08	0.02	0.257
Cholesterol esters to total lipids ratio in IDL (%)	605	-0.02	-0.08	0.03	0.399	605	-0.02	-0.07	0.03	0.438	605	-0.02	-0.07	0.03	0.485
Free cholesterol to total lipids ratio in IDL (%)	605	-0.02	-0.08	0.03	0.429	605	-0.03	-0.09	0.03	0.360	605	-0.03	-0.09	0.02	0.242
Triglycerides to total lipids ratio in IDL (%)	605	0.03	-0.02	0.08	0.289	605	0.03	-0.02	0.08	0.272	605	0.03	-0.02	0.08	0.248
Phospholipids to total lipds ratio in large LDL (%)	605	0.04	-0.02	0.10	0.204	605	0.04	-0.02	0.10	0.215	605	0.03	-0.01	0.08	0.129
Total cholesterol to total lipids ratio in large LDL (%)	605	-0.05	-0.12	0.02	0.135	605	-0.05	-0.12	0.02	0.138	605	-0.05	-0.10	0.00	0.038
Cholesterol esters to total lipids ratio in large LDL (%)	605	-0.05	-0.12	0.02	0.131	605	-0.05	-0.11	0.02	0.145	605	-0.04	-0.08	0.00	0.041
Free cholesterol to total lipids ratio in large LDL (%)	605	0.03	-0.03	0.08	0.385	605	0.02	-0.04	0.08	0.469	605	0.02	-0.04	0.07	0.531
Triglycerides to total lipids ratio in large LDL (%)	605	0.04	-0.02	0.09	0.209	605	0.04	-0.02	0.09	0.204	605	0.04	-0.01	0.09	0.153
Phospholipids to total lipds ratio in medium LDL (%)	605	0.03	-0.03	0.09	0.294	605	0.03	-0.03	0.09	0.304	605	0.04	-0.01	0.09	0.098
Total cholesterol to total lipids ratio in medium LDL (%)	605	-0.05	-0.13	0.02	0.150	605	-0.05	-0.13	0.02	0.153	605	-0.07	-0.13	-0.01	0.029
Cholesterol esters to total lipids ratio in medium LDL (%)	605	-0.04	-0.10	0.02	0.212	605	-0.04	-0.10	0.02	0.228	605	-0.05	-0.09	0.00	0.039
Free cholesterol to total lipids ratio in medium LDL (%)	605	0.01	-0.05	0.07	0.815	605	0.01	-0.05	0.07	0.865	605	0.01	-0.05	0.07	0.739
Triglycerides to total lipids ratio in medium LDL (%)	605	0.06	-0.04	0.16	0.259	605	0.06	-0.04	0.16	0.255	605	0.06	-0.04	0.16	0.255
Phospholipids to total lipds ratio in small LDL (%)	605	0.05	-0.02	0.12	0.196	605	0.04	-0.02	0.11	0.205	605	0.06	0.00	0.11	0.033
Total cholesterol to total lipids ratio in small LDL (%)	605	-0.04	-0.11	0.02	0.209	605	-0.04	-0.11	0.02	0.209	605	-0.06	-0.11	0.00	0.045
Cholesterol esters to total lipids ratio in small LDL (%)	605	-0.03	-0.09	0.02	0.248	605	-0.03	-0.09	0.02	0.260	605	-0.05	-0.09	0.00	0.050
Free cholesterol to total lipids ratio in small LDL (%)	605	0.01	-0.04	0.06	0.717	605	0.01	-0.05	0.06	0.768	605	0.01	-0.04	0.07	0.631
Triglycerides to total lipids ratio in small LDL (%)	605	0.00	-0.05	0.06	0.900	605	0.01	-0.05	0.06	0.820	605	0.01	-0.05	0.06	0.834
Phospholipids to total lipds ratio in very large HDL (%)	605	0.00	-0.05	0.06	0.915	605	0.00	-0.05	0.05	0.973	605	0.01	-0.04	0.06	0.678
Total cholesterol to total lipids ratio in very large HDL (%)	605	0.00	-0.05	0.05	0.946	605	0.00	-0.05	0.05	0.964	605	-0.01	-0.06	0.03	0.643
Cholesterol esters to total lipids ratio in very large HDL (%)	605	0.00	-0.05	0.05	0.997	605	0.00	-0.05	0.05	0.911	605	-0.01	-0.05	0.03	0.664
Free cholesterol to total lipids ratio in very large HDL (%)	605	-0.02	-0.07	0.03	0.482	605	-0.02	-0.07	0.03	0.438	605	-0.02	-0.07	0.04	0.551
Triglycerides to total lipids ratio in very large HDL (%)	605	0.00	-0.06	0.05	0.910	605	0.00	-0.05	0.06	0.952	605	0.00	-0.05	0.05	0.949
Phospholipids to total lipds ratio in large HDL (%)	605	-0.01	-0.06	0.04	0.672	605	0.00	-0.05	0.04	0.851	605	-0.02	-0.07	0.02	0.294
Total cholesterol to total lipids ratio in large HDL (%)	605	0.01	-0.04	0.06	0.777	605	0.00	-0.05	0.05	0.968	605	0.02	-0.02	0.07	0.307
Cholesterol esters to total lipids ratio in large HDL (%)	605	0.01	-0.04	0.06	0.723	605	0.00	-0.05	0.05	0.896	605	0.02	-0.02	0.07	0.293
Free cholesterol to total lipids ratio in large HDL (%)	605	0.00	-0.05	0.05	0.939	605	-0.01	-0.06	0.04	0.763	605	0.01	-0.04	0.06	0.670
Triglycerides to total lipids ratio in large HDL (%)	605	0.00	-0.05	0.06	0.858	605	0.01	-0.04	0.06	0.712	605	0.00	-0.05	0.05	0.911
Phospholipids to total lipds ratio in medium HDL (%)	605	0.00	-0.05	0.05	0.956	605	0.00	-0.05	0.05	0.907	605	0.00	-0.05	0.05	0.964
Total cholesterol to total lipids ratio in medium HDL (%)	605	0.00	-0.05	0.05	0.927	605	0.00	-0.05	0.05	0.954	605	0.01	-0.04	0.05	0.795
Cholesterol esters to total lipids ratio in medium HDL (%)	605	0.00	-0.05	0.05	0.997	605	0.00	-0.05	0.04	0.880	605	0.00	-0.04	0.05	0.879
Free cholesterol to total lipids ratio in medium HDL (%)	605	0.01	-0.05	0.07	0.809	605	0.01	-0.06	0.07	0.839	605	0.01	-0.05	0.07	0.753
Triglycerides to total lipids ratio in medium HDL (%)	605	0.00	-0.06	0.05	0.879	605	0.00	-0.05	0.05	0.967	605	-0.01	-0.06	0.03	0.581
Phospholipids to total lipds ratio in small HDL (%)	605	0.03	-0.01	0.07	0.181	605	0.03	-0.01	0.07	0.178	605	0.03	-0.01	0.07	0.130
Total cholesterol to total lipids ratio in small HDL (%)	605	-0.03	-0.08	0.01	0.156	605	-0.04	-0.08	0.01	0.140	605	-0.04	-0.08	0.01	0.114
Cholesterol esters to total lipids ratio in small HDL (%)	605	-0.03	-0.08	0.02	0.187	605	-0.03	-0.08	0.01	0.175	605	-0.03	-0.08	0.01	0.139
Free cholesterol to total lipids ratio in small HDL (%)	605	0.00	-0.06	0.05	0.857	605	-0.01	-0.06	0.04	0.766	605	0.00	-0.05	0.05	0.976
Triglycerides to total lipids ratio in small HDL (%)	605	0.01	-0.04	0.07	0.624	605	0.02	-0.04	0.08	0.510	605	0.02	-0.04	0.07	0.584
Mean diameter for VLDL particles (nm)	605	-0.01	-0.06	0.05	0.806	605	0.00	-0.06	0.05	0.943	605	0.00	-0.05	0.05	0.965
Mean diameter for LDL particles (nm)	605	0.04	-0.02	0.11	0.187	605	0.04	-0.02	0.10	0.209	605	0.05	-0.01	0.11	0.101

S4 Table Observational associations of age at voice breaking (per year later) with adiposity and cardiometabolic traits at age 18y among males in ALSPAC

	Adj. for age, education					*Adj. for age, education, BMI at age 8y*					*Adj. for age, education, outcome value at age 8y*				
**Standardised outcome at age 18y**	**N**	**Beta**	**LCL**	**UCL**	**P-value**	**N**	**Beta**	**LCL**	**UCL**	**P-value**	**N**	**Beta**	**LCL**	**UCL**	**P-value**
Mean diameter for HDL particles (nm)	605	-0.01	-0.06	0.04	0.732	605	-0.01	-0.06	0.04	0.628	605	0.01	-0.03	0.04	0.720
Serum total cholesterol (mmol/l)	605	-0.03	-0.07	0.02	0.250	605	-0.02	-0.07	0.02	0.276	605	-0.03	-0.07	0.00	0.040
Total cholesterol in VLDL (mmol/l)	605	-0.01	-0.07	0.04	0.649	605	-0.01	-0.06	0.05	0.773	605	-0.01	-0.06	0.03	0.556
Remnant cholesterol (non-HDL, non-LDL -cholesterol) (mmol/l)	605	-0.02	-0.07	0.03	0.435	605	-0.02	-0.07	0.03	0.514	605	-0.03	-0.07	0.02	0.213
Total cholesterol in LDL (mmol/l)	605	-0.02	-0.07	0.02	0.286	605	-0.02	-0.07	0.02	0.312	605	-0.04	-0.07	0.00	0.037
Total cholesterol in HDL (mmol/l)	605	-0.01	-0.06	0.03	0.543	605	-0.02	-0.06	0.03	0.486	605	-0.01	-0.05	0.02	0.539
Total cholesterol in HDL2 (mmol/l)	605	-0.01	-0.06	0.03	0.610	605	-0.01	-0.06	0.03	0.534	605	-0.01	-0.05	0.03	0.600
Total cholesterol in HDL3 (mmol/l)	605	-0.02	-0.06	0.03	0.450	605	-0.02	-0.06	0.03	0.427	605	-0.01	-0.04	0.02	0.462
Esterified cholesterol (mmol/l)	605	-0.03	-0.07	0.02	0.270	605	-0.02	-0.07	0.02	0.294	605	-0.03	-0.07	0.00	0.054
Free cholesterol (mmol/l)	605	-0.02	-0.07	0.02	0.262	605	-0.02	-0.07	0.02	0.291	605	-0.03	-0.07	0.00	0.066
Serum total triglycerides (mmol/l)	605	0.00	-0.06	0.05	0.882	605	0.00	-0.05	0.06	0.957	605	0.00	-0.06	0.05	0.926
Triglycerides in VLDL (mmol/l)	605	0.00	-0.06	0.05	0.876	605	0.00	-0.05	0.06	0.963	605	0.00	-0.06	0.05	0.952
Triglycerides in LDL (mmol/l)	605	0.00	-0.04	0.04	0.962	605	0.00	-0.04	0.04	0.960	605	0.00	-0.04	0.03	0.858
Triglycerides in HDL (mmol/l)	605	0.00	-0.05	0.04	0.880	605	0.00	-0.04	0.05	0.973	605	0.00	-0.05	0.04	0.865
Diacylglycerol (mmol/l)	605	-0.01	-0.07	0.04	0.561	605	-0.01	-0.06	0.04	0.651	605	-0.01	-0.06	0.04	0.676
Ratio of diacylglycerol to triglycerides	605	0.00	-0.05	0.04	0.888	605	0.00	-0.05	0.04	0.942	605	0.00	-0.05	0.04	0.955
Total phosphoglycerides (mmol/l)	605	-0.03	-0.07	0.01	0.153	605	-0.03	-0.07	0.01	0.187	605	-0.03	-0.07	0.00	0.081
Ratio of triglycerides to phosphoglycerides	605	0.00	-0.05	0.06	0.862	605	0.01	-0.05	0.07	0.726	605	0.01	-0.04	0.07	0.697
Phosphatidylcholine and other cholines (mmol/l)	605	-0.02	-0.05	0.02	0.442	605	-0.01	-0.05	0.02	0.460	605	-0.02	-0.06	0.01	0.178
Total cholines (mmol/l)	605	-0.03	-0.07	0.01	0.162	605	-0.03	-0.07	0.01	0.187	605	-0.04	-0.07	0.00	0.035
Apolipoprotein A-I (g/l)	605	-0.02	-0.06	0.02	0.310	605	-0.02	-0.06	0.02	0.303	605	-0.02	-0.05	0.01	0.209
Apolipoprotein B (g/l)	605	-0.02	-0.07	0.03	0.447	605	-0.02	-0.07	0.03	0.544	605	-0.02	-0.07	0.02	0.290
Ratio of apolipoprotein B to apolipoprotein A-I	605	-0.01	-0.07	0.04	0.682	605	-0.01	-0.06	0.05	0.804	605	-0.02	-0.06	0.03	0.495
Total fatty acids (mmol/l)	605	-0.02	-0.07	0.03	0.399	605	-0.02	-0.06	0.03	0.497	605	-0.02	-0.06	0.02	0.370
Estimated description of fatty acid chain length, not actual carbon number	605	0.06	0.01	0.11	0.016	605	0.06	0.01	0.11	0.014	605	0.06	0.01	0.11	0.016
Estimated degree of unsaturation	605	0.04	-0.01	0.10	0.100	605	0.04	-0.01	0.10	0.118	605	0.04	-0.02	0.09	0.186
22:6, docosahexaenoic acid (mmol/l)	605	0.01	-0.04	0.05	0.776	605	0.01	-0.03	0.05	0.680	605	-0.01	-0.05	0.03	0.774
18:2, linoleic acid (mmol/l)	605	-0.03	-0.07	0.02	0.262	605	-0.02	-0.07	0.02	0.280	605	-0.03	-0.06	0.01	0.176
Conjugated linoleic acid (mmol/l)	605	-0.02	-0.07	0.02	0.338	605	-0.02	-0.07	0.03	0.414	605	-0.02	-0.07	0.03	0.356
Omega-3 fatty acids (mmol/l)	605	0.00	-0.05	0.05	0.956	605	0.00	-0.05	0.05	0.967	605	-0.01	-0.06	0.04	0.613
Omega-6 fatty acids (mmol/l)	605	-0.02	-0.07	0.02	0.348	605	-0.02	-0.06	0.03	0.391	605	-0.02	-0.06	0.01	0.199
Polyunsaturated fatty acids (mmol/l)	605	-0.02	-0.06	0.03	0.401	605	-0.02	-0.06	0.03	0.451	605	-0.02	-0.06	0.01	0.216
Monounsaturated fatty acids; 16:1, 18:1 (mmol/l)	605	-0.01	-0.06	0.04	0.699	605	0.00	-0.05	0.04	0.851	605	-0.01	-0.06	0.04	0.739
Saturated fatty acids (mmol/l)	605	-0.03	-0.07	0.02	0.280	605	-0.02	-0.07	0.03	0.360	605	-0.03	-0.07	0.02	0.277
Ratio of 22:6 docosahexaenoic acid to total fatty acids (%)	605	0.03	-0.02	0.08	0.262	605	0.03	-0.02	0.08	0.242	605	0.01	-0.03	0.06	0.541
Ratio of 18:2 linoleic acid to total fatty acids (%)	605	-0.01	-0.06	0.04	0.671	605	-0.02	-0.07	0.04	0.529	605	-0.01	-0.06	0.04	0.668
Ratio of conjugated linoleic acid to total fatty acids (%)	605	-0.02	-0.06	0.03	0.469	605	-0.01	-0.06	0.03	0.541	605	-0.02	-0.06	0.03	0.471
Ratio of omega-3 fatty acids to total fatty acids (%)	605	0.02	-0.03	0.07	0.431	605	0.02	-0.03	0.07	0.442	605	0.01	-0.04	0.06	0.684
Ratio of omega-6 fatty acids to total fatty acids (%)	605	0.00	-0.05	0.06	0.930	605	0.00	-0.06	0.05	0.930	605	0.00	-0.05	0.06	0.980
Ratio of polyunsaturated fatty acids to total fatty acids (%)	605	0.01	-0.05	0.06	0.794	605	0.00	-0.05	0.06	0.928	605	0.00	-0.05	0.06	0.883
Ratio of monounsaturated fatty acids to total fatty acids (%)	605	0.01	-0.04	0.06	0.708	605	0.01	-0.04	0.07	0.592	605	0.01	-0.04	0.07	0.607
Ratio of saturated fatty acids to total fatty acids (%)	605	-0.02	-0.07	0.03	0.364	605	-0.02	-0.07	0.03	0.366	605	-0.02	-0.07	0.03	0.373
Glucose (mmol/l)	605	-0.02	-0.06	0.01	0.170	605	-0.02	-0.06	0.01	0.190	605	-0.02	-0.06	0.01	0.194
Lactate (mmol/l)	605	-0.02	-0.07	0.02	0.319	605	-0.02	-0.07	0.02	0.338	605	-0.02	-0.07	0.02	0.323
Pyruvate (mmol/l)	605	-0.03	-0.08	0.02	0.214	605	-0.03	-0.08	0.02	0.259	605	-0.03	-0.08	0.02	0.210
Citrate (mmol/l)	605	0.04	-0.02	0.09	0.157	605	0.04	-0.02	0.09	0.196	605	0.04	-0.01	0.09	0.144
Alanine (mmol/l)	605	-0.02	-0.07	0.03	0.386	605	-0.02	-0.07	0.03	0.438	605	-0.02	-0.07	0.03	0.415
Glutamine (mmol/l)	605	0.00	-0.04	0.04	1.000	605	0.00	-0.04	0.04	0.936	605	0.00	-0.04	0.05	0.884
Histidine (mmol/l)	605	-0.01	-0.06	0.04	0.755	605	-0.01	-0.06	0.04	0.759	605	-0.01	-0.06	0.04	0.692
Isoleucine (mmol/l)	605	0.04	-0.01	0.09	0.159	605	0.04	-0.01	0.10	0.116	605	0.04	-0.02	0.09	0.165
Leucine (mmol/l)	605	0.03	-0.02	0.07	0.287	605	0.03	-0.02	0.08	0.221	605	0.03	-0.02	0.07	0.284
Valine (mmol/l)	605	0.04	-0.01	0.09	0.148	605	0.04	-0.01	0.09	0.112	605	0.03	-0.01	0.08	0.169

S4 Table Observational associations of age at voice breaking (per year later) with adiposity and cardiometabolic traits at age 18y among males in ALSPAC

	Adj. for age, education					*Adj. for age, education, BMI at age 8y*					*Adj. for age, education, outcome value at age 8y*				
**Standardised outcome at age 18y**	**N**	**Beta**	**LCL**	**UCL**	**P-value**	**N**	**Beta**	**LCL**	**UCL**	**P-value**	**N**	**Beta**	**LCL**	**UCL**	**P-value**
Phenylalanine (mmol/l)	605	0.00	-0.05	0.05	0.922	605	0.01	-0.05	0.06	0.843	605	0.00	-0.05	0.05	0.888
Tyrosine (mmol/l)	605	0.00	-0.04	0.04	0.975	605	0.00	-0.04	0.04	0.914	605	0.00	-0.04	0.04	0.976
Acetate (mmol/l)	605	0.01	-0.01	0.03	0.189	605	0.01	-0.01	0.03	0.201	605	0.01	-0.01	0.03	0.193
Acetoacetate (mmol/l)	605	0.01	-0.05	0.07	0.680	605	0.02	-0.04	0.07	0.606	605	0.01	-0.05	0.07	0.679
3-hydroxybutyrate (mmol/l)	605	0.04	-0.01	0.09	0.146	605	0.04	-0.01	0.09	0.131	605	0.04	-0.01	0.09	0.146
Creatinine (mmol/l)	605	-0.05	-0.09	0.00	0.056	605	-0.04	-0.09	0.00	0.072	605	-0.03	-0.08	0.01	0.121
Albumin (signal area)	605	0.00	-0.05	0.05	0.969	605	0.00	-0.05	0.05	0.973	605	0.00	-0.05	0.05	0.904
Glycoprotein acetyls, mainly a1-acid glycoprotein (mmol/l)	605	0.02	-0.04	0.07	0.545	605	0.02	-0.03	0.07	0.441	605	0.02	-0.03	0.08	0.377
