# Supplementary material for: Influence of puberty timing on adiposity and cardiometabolic traits: A Mendelian randomisation study
Source: PLoS Med. 2018 Aug 28;15(8):e1002641. doi: 10.1371/journal.pmed.1002641 (PMC6112630; doi:10.1371/journal.pmed.1002641)
Supplement: S5 Table — (PDF) [file pmed.1002641.s024.pdf]

S5 Table Observational associations of age at puberty onset (per year later) with adiposity and cardiometabolic traits at age 18y among females and males in ALSPAC

	Adj. for age, sex, education					*Adj. for age, sex, education, BMI at age 8y*					*Adj. for age, sex, education, outcome value at age 8y*				
**Standardised outcome at age 18y**	**N**	**Beta**	**LCL**	**UCL**	**P-value**	**N**	**Beta**	**LCL**	**UCL**	**P-value**	**N**	**Beta**	**LCL**	**UCL**	**P-value**
Body mass index (kg/m^2)	3784	-0.11	-0.14	-0.09	2.02E-21	3404	-0.02	-0.04	0.00	0.015	3404	-0.02	-0.04	0.00	0.015
Fat mass index (kg/m^2)	3663	-0.09	-0.11	-0.07	3.50E-16	3309	-0.01	-0.03	0.00	0.122	3212	-0.01	-0.02	0.01	0.376
Lean mass index (kg/m^2)	3663	-0.05	-0.07	-0.04	2.38E-10	3309	-0.01	-0.03	0.00	0.091	3212	0.00	-0.01	0.01	0.721
Systolic blood pressure (mmHg)	3561	-0.04	-0.07	-0.02	4.66E-05	3221	-0.02	-0.04	0.00	0.064	3177	-0.02	-0.05	0.00	0.029
Diastolic blood pressure (mmHg)	3561	-0.05	-0.08	-0.03	2.27E-05	3221	-0.03	-0.06	0.00	0.023	3176	-0.04	-0.06	-0.02	1.43E-03
Concentration of chylomicrons and extremely large VLDL particles (mol/l)	2424	-0.01	-0.04	0.02	0.501	2188	0.01	-0.02	0.04	0.589	1659	0.00	-0.04	0.04	0.951
Total lipids in chylomicrons and extremely large VLDL (mmol/l)	2424	-0.01	-0.04	0.02	0.487	2188	0.01	-0.02	0.04	0.607	1659	0.00	-0.04	0.04	0.953
Phospholipids in chylomicrons and extremely large VLDL (mmol/l)	2424	-0.01	-0.04	0.02	0.513	2188	0.01	-0.02	0.04	0.606	1659	0.00	-0.04	0.04	0.956
Total cholesterol in chylomicrons and extremely large VLDL (mmol/l)	2424	-0.01	-0.04	0.02	0.492	2188	0.01	-0.02	0.04	0.639	1659	0.00	-0.04	0.04	0.947
Cholesterol esters in chylomicrons and extremely large VLDL (mmol/l)	2424	-0.01	-0.04	0.02	0.508	2188	0.01	-0.02	0.04	0.651	1659	0.00	-0.04	0.04	0.947
Free cholesterol in chylomicrons and extremely large VLDL (mmol/l)	2424	-0.01	-0.04	0.02	0.490	2188	0.01	-0.03	0.04	0.630	1659	0.00	-0.04	0.04	0.954
Triglycerides in chylomicrons and extremely large VLDL (mmol/l)	2424	-0.01	-0.04	0.02	0.485	2188	0.01	-0.02	0.04	0.599	1659	0.00	-0.04	0.04	0.960
Concentration of very large VLDL particles (mol/l)	2424	-0.01	-0.04	0.02	0.470	2188	0.01	-0.02	0.04	0.610	1659	0.00	-0.04	0.04	0.906
Total lipids in very large VLDL (mmol/l)	2424	-0.01	-0.04	0.02	0.472	2188	0.01	-0.02	0.04	0.624	1659	0.00	-0.04	0.04	0.896
Phospholipids in very large VLDL (mmol/l)	2424	-0.01	-0.04	0.02	0.484	2188	0.01	-0.03	0.04	0.652	1659	0.00	-0.04	0.04	0.863
Total cholesterol in very large VLDL (mmol/l)	2424	-0.01	-0.04	0.02	0.505	2188	0.01	-0.02	0.04	0.592	1659	0.00	-0.04	0.04	0.960
Cholesterol esters in very large VLDL (mmol/l)	2424	-0.01	-0.04	0.02	0.504	2188	0.01	-0.02	0.04	0.566	1659	0.00	-0.04	0.04	0.999
Free cholesterol in very large VLDL (mmol/l)	2424	-0.01	-0.04	0.02	0.505	2188	0.01	-0.02	0.04	0.627	1659	0.00	-0.04	0.04	0.921
Triglycerides in very large VLDL (mmol/l)	2424	-0.01	-0.04	0.02	0.460	2188	0.01	-0.02	0.04	0.629	1659	0.00	-0.04	0.04	0.888
Concentration of large VLDL particles (mol/l)	2424	-0.01	-0.04	0.02	0.486	2188	0.01	-0.02	0.04	0.625	1659	0.00	-0.04	0.04	0.886
Total lipids in large VLDL (mmol/l)	2424	-0.01	-0.04	0.02	0.506	2188	0.01	-0.02	0.04	0.605	1659	0.00	-0.04	0.04	0.921
Phospholipids in large VLDL (mmol/l)	2424	-0.01	-0.04	0.02	0.640	2188	0.01	-0.02	0.04	0.500	1659	0.00	-0.04	0.04	0.926
Total cholesterol in large VLDL (mmol/l)	2424	-0.01	-0.04	0.02	0.487	2188	0.01	-0.03	0.04	0.666	1659	0.00	-0.04	0.03	0.865
Cholesterol esters in large VLDL (mmol/l)	2424	-0.01	-0.04	0.02	0.483	2188	0.01	-0.03	0.04	0.676	1659	0.00	-0.04	0.03	0.889
Free cholesterol in large VLDL (mmol/l)	2424	-0.01	-0.04	0.02	0.494	2188	0.01	-0.03	0.04	0.658	1659	0.00	-0.04	0.04	0.852
Triglycerides in large VLDL (mmol/l)	2424	-0.01	-0.04	0.02	0.479	2188	0.01	-0.02	0.04	0.619	1659	0.00	-0.04	0.04	0.897
Concentration of medium VLDL particles (mol/l)	2424	-0.01	-0.04	0.02	0.551	2188	0.01	-0.02	0.04	0.567	1659	0.00	-0.04	0.04	0.958
Total lipids in medium VLDL (mmol/l)	2424	-0.01	-0.04	0.02	0.531	2188	0.01	-0.02	0.04	0.602	1659	0.00	-0.04	0.04	0.936
Phospholipids in medium VLDL (mmol/l)	2424	-0.01	-0.04	0.02	0.576	2188	0.01	-0.02	0.04	0.610	1659	0.00	-0.04	0.04	0.936
Total cholesterol in medium VLDL (mmol/l)	2424	-0.01	-0.04	0.02	0.515	2188	0.00	-0.03	0.04	0.765	1659	0.00	-0.04	0.03	0.867
Cholesterol esters in medium VLDL (mmol/l)	2424	-0.01	-0.04	0.02	0.489	2188	0.00	-0.03	0.03	0.879	1659	0.00	-0.04	0.03	0.843
Free cholesterol in medium VLDL (mmol/l)	2424	-0.01	-0.04	0.02	0.566	2188	0.01	-0.02	0.04	0.647	1659	0.00	-0.04	0.03	0.899
Triglycerides in medium VLDL (mmol/l)	2424	-0.01	-0.04	0.02	0.534	2188	0.01	-0.02	0.04	0.531	1659	0.00	-0.04	0.04	0.974
Concentration of small VLDL particles (mol/l)	2424	0.00	-0.03	0.03	0.790	2188	0.01	-0.02	0.04	0.636	1659	-0.01	-0.04	0.03	0.749
Total lipids in small VLDL (mmol/l)	2424	0.00	-0.03	0.03	0.829	2188	0.01	-0.02	0.04	0.650	1659	-0.01	-0.04	0.03	0.674
Phospholipids in small VLDL (mmol/l)	2424	0.00	-0.03	0.03	0.889	2188	0.01	-0.03	0.04	0.739	1659	-0.01	-0.04	0.02	0.485
Total cholesterol in small VLDL (mmol/l)	2424	0.00	-0.03	0.03	0.929	2188	0.01	-0.02	0.04	0.716	1659	-0.01	-0.04	0.02	0.595
Cholesterol esters in small VLDL (mmol/l)	2424	0.00	-0.03	0.03	0.934	2188	0.01	-0.02	0.04	0.725	1659	-0.01	-0.04	0.02	0.703
Free cholesterol in small VLDL (mmol/l)	2424	0.00	-0.03	0.03	0.927	2188	0.01	-0.03	0.04	0.735	1659	-0.01	-0.04	0.02	0.505
Triglycerides in small VLDL (mmol/l)	2424	-0.01	-0.04	0.02	0.734	2188	0.01	-0.02	0.04	0.610	1659	0.00	-0.04	0.03	0.815
Concentration of very small VLDL particles (mol/l)	2424	0.00	-0.02	0.03	0.725	2188	0.00	-0.03	0.03	0.811	1659	-0.01	-0.04	0.02	0.450
Total lipids in very small VLDL (mmol/l)	2424	0.01	-0.02	0.03	0.685	2188	0.01	-0.02	0.04	0.673	1659	-0.01	-0.04	0.02	0.558
Phospholipids in very small VLDL (mmol/l)	2424	0.00	-0.02	0.03	0.812	2188	0.00	-0.03	0.03	0.962	1659	-0.01	-0.04	0.01	0.294
Total cholesterol in very small VLDL (mmol/l)	2424	0.01	-0.02	0.04	0.598	2188	0.01	-0.02	0.04	0.526	1659	0.00	-0.03	0.03	0.995
Cholesterol esters in very small VLDL (mmol/l)	2424	0.00	-0.02	0.03	0.742	2188	0.01	-0.02	0.04	0.544	1659	0.00	-0.03	0.03	0.951
Free cholesterol in very small VLDL (mmol/l)	2424	0.01	-0.01	0.04	0.325	2188	0.01	-0.02	0.04	0.518	1659	0.00	-0.03	0.03	0.971
Triglycerides in very small VLDL (mmol/l)	2424	0.00	-0.03	0.03	0.938	2188	0.00	-0.03	0.03	0.806	1659	-0.01	-0.04	0.02	0.612
Concentration of IDL particles (mol/l)	2424	0.00	-0.02	0.03	0.874	2188	0.00	-0.03	0.03	0.827	1659	-0.01	-0.04	0.01	0.299
Total lipids in IDL (mmol/l)	2424	0.00	-0.02	0.03	0.870	2188	0.00	-0.03	0.03	0.855	1659	-0.02	-0.04	0.01	0.253

S5 Table Observational associations of age at puberty onset (per year later) with adiposity and cardiometabolic traits at age 18y among females and males in ALSPAC

	Adj. for age, sex, education					*Adj. for age, sex, education, BMI at age 8y*					*Adj. for age, sex, education, outcome value at age 8y*				
**Standardised outcome at age 18y**	**N**	**Beta**	**LCL**	**UCL**	**P-value**	**N**	**Beta**	**LCL**	**UCL**	**P-value**	**N**	**Beta**	**LCL**	**UCL**	**P-value**
Phospholipids in IDL (mmol/l)	2424	0.00	-0.03	0.03	0.979	2188	-0.01	-0.04	0.02	0.654	1659	-0.02	-0.04	0.01	0.212
Total cholesterol in IDL (mmol/l)	2424	0.00	-0.03	0.03	0.870	2188	0.00	-0.03	0.03	0.934	1659	-0.01	-0.04	0.01	0.306
Cholesterol esters in IDL (mmol/l)	2424	0.00	-0.03	0.03	0.887	2188	0.00	-0.03	0.03	0.978	1659	-0.01	-0.04	0.01	0.389
Free cholesterol in IDL (mmol/l)	2424	0.00	-0.02	0.03	0.834	2188	-0.01	-0.03	0.02	0.721	1659	-0.02	-0.04	0.01	0.223
Triglycerides in IDL (mmol/l)	2424	0.00	-0.02	0.03	0.700	2188	0.00	-0.03	0.03	0.907	1659	-0.01	-0.03	0.02	0.714
Concentration of large LDL particles (mol/l)	2424	0.00	-0.03	0.03	0.952	2188	-0.01	-0.04	0.02	0.660	1659	-0.02	-0.05	0.01	0.199
Total lipids in large LDL (mmol/l)	2424	0.00	-0.03	0.03	0.994	2188	-0.01	-0.03	0.02	0.727	1659	-0.02	-0.04	0.01	0.183
Phospholipids in large LDL (mmol/l)	2424	0.00	-0.03	0.03	0.940	2188	-0.01	-0.03	0.02	0.720	1659	-0.02	-0.05	0.01	0.181
Total cholesterol in large LDL (mmol/l)	2424	0.00	-0.03	0.03	0.995	2188	0.00	-0.03	0.02	0.741	1659	-0.02	-0.05	0.01	0.163
Cholesterol esters in large LDL (mmol/l)	2424	0.00	-0.03	0.03	0.972	2188	0.00	-0.03	0.02	0.754	1659	-0.02	-0.05	0.01	0.163
Free cholesterol in large LDL (mmol/l)	2424	0.00	-0.03	0.03	0.888	2188	-0.01	-0.03	0.02	0.710	1659	-0.02	-0.05	0.01	0.178
Triglycerides in large LDL (mmol/l)	2424	0.00	-0.02	0.03	0.864	2188	-0.01	-0.03	0.02	0.712	1659	-0.01	-0.03	0.02	0.730
Concentration of medium LDL particles (mol/l)	2424	0.00	-0.03	0.02	0.822	2188	-0.01	-0.04	0.02	0.603	1659	-0.02	-0.05	0.01	0.187
Total lipids in medium LDL (mmol/l)	2424	0.00	-0.03	0.03	0.986	2188	0.00	-0.03	0.02	0.743	1659	-0.02	-0.05	0.01	0.174
Phospholipids in medium LDL (mmol/l)	2424	0.00	-0.03	0.03	0.975	2188	0.00	-0.03	0.03	0.869	1659	-0.02	-0.05	0.01	0.180
Total cholesterol in medium LDL (mmol/l)	2424	0.00	-0.03	0.03	0.985	2188	0.00	-0.03	0.02	0.739	1659	-0.02	-0.05	0.01	0.154
Cholesterol esters in medium LDL (mmol/l)	2424	0.00	-0.03	0.03	0.941	2188	-0.01	-0.03	0.02	0.705	1659	-0.02	-0.05	0.01	0.166
Free cholesterol in medium LDL (mmol/l)	2424	0.00	-0.02	0.03	0.829	2188	0.00	-0.03	0.03	0.896	1659	-0.02	-0.05	0.01	0.155
Triglycerides in medium LDL (mmol/l)	2424	0.00	-0.03	0.03	0.994	2188	-0.01	-0.03	0.02	0.597	1659	0.00	-0.03	0.03	0.780
Concentration of small LDL particles (mol/l)	2424	0.00	-0.03	0.03	0.982	2188	0.00	-0.03	0.02	0.750	1659	-0.02	-0.05	0.01	0.197
Total lipids in small LDL (mmol/l)	2424	0.00	-0.03	0.03	0.977	2188	0.00	-0.03	0.03	0.790	1659	-0.02	-0.05	0.01	0.149
Phospholipids in small LDL (mmol/l)	2424	0.00	-0.03	0.03	0.876	2188	0.00	-0.03	0.03	0.981	1659	-0.02	-0.05	0.01	0.192
Total cholesterol in small LDL (mmol/l)	2424	0.00	-0.03	0.03	0.957	2188	0.00	-0.03	0.03	0.777	1659	-0.02	-0.05	0.01	0.137
Cholesterol esters in small LDL (mmol/l)	2424	0.00	-0.03	0.03	0.947	2188	-0.01	-0.04	0.02	0.688	1659	-0.02	-0.05	0.01	0.161
Free cholesterol in small LDL (mmol/l)	2424	0.01	-0.02	0.04	0.594	2188	0.00	-0.03	0.03	0.839	1659	-0.02	-0.05	0.01	0.196
Triglycerides in small LDL (mmol/l)	2424	-0.01	-0.03	0.02	0.672	2188	-0.01	-0.04	0.02	0.604	1659	-0.01	-0.04	0.02	0.511
Concentration of very large HDL particles (mol/l)	2424	0.01	-0.02	0.03	0.713	2188	-0.01	-0.04	0.02	0.686	1659	0.00	-0.03	0.03	0.880
Total lipids in very large HDL (mmol/l)	2424	0.01	-0.02	0.04	0.553	2188	0.00	-0.03	0.03	0.904	1659	0.00	-0.02	0.03	0.780
Phospholipids in very large HDL (mmol/l)	2424	0.00	-0.02	0.03	0.817	2188	-0.01	-0.04	0.02	0.522	1659	0.00	-0.03	0.03	0.993
Total cholesterol in very large HDL (mmol/l)	2424	0.01	-0.01	0.04	0.352	2188	0.01	-0.02	0.04	0.676	1659	0.01	-0.02	0.04	0.631
Cholesterol esters in very large HDL (mmol/l)	2424	0.02	-0.01	0.04	0.289	2188	0.01	-0.02	0.04	0.539	1659	0.01	-0.02	0.04	0.561
Free cholesterol in very large HDL (mmol/l)	2424	0.01	-0.02	0.04	0.598	2188	0.00	-0.03	0.03	0.887	1659	0.00	-0.03	0.03	0.839
Triglycerides in very large HDL (mmol/l)	2424	0.00	-0.03	0.03	0.907	2188	0.00	-0.03	0.03	0.839	1659	0.01	-0.02	0.04	0.526
Concentration of large HDL particles (mol/l)	2424	0.00	-0.03	0.03	0.896	2188	-0.01	-0.04	0.01	0.342	1659	-0.01	-0.03	0.02	0.678
Total lipids in large HDL (mmol/l)	2424	0.00	-0.02	0.03	0.866	2188	-0.01	-0.04	0.02	0.372	1659	-0.01	-0.03	0.02	0.697
Phospholipids in large HDL (mmol/l)	2424	0.00	-0.03	0.03	0.988	2188	-0.02	-0.04	0.01	0.287	1659	-0.01	-0.04	0.02	0.513
Total cholesterol in large HDL (mmol/l)	2424	0.00	-0.02	0.03	0.733	2188	-0.01	-0.04	0.02	0.469	1659	0.00	-0.03	0.03	0.876
Cholesterol esters in large HDL (mmol/l)	2424	0.00	-0.02	0.03	0.741	2188	-0.01	-0.04	0.02	0.464	1659	0.00	-0.03	0.03	0.888
Free cholesterol in large HDL (mmol/l)	2424	0.01	-0.02	0.03	0.699	2188	-0.01	-0.04	0.02	0.500	1659	0.00	-0.03	0.02	0.848
Triglycerides in large HDL (mmol/l)	2424	-0.01	-0.04	0.02	0.474	2188	-0.02	-0.05	0.01	0.233	1659	-0.01	-0.04	0.02	0.686
Concentration of medium HDL particles (mol/l)	2424	-0.01	-0.03	0.02	0.569	2188	-0.02	-0.04	0.01	0.234	1659	-0.02	-0.04	0.01	0.301
Total lipids in medium HDL (mmol/l)	2424	-0.01	-0.03	0.02	0.705	2188	-0.02	-0.04	0.01	0.285	1659	-0.01	-0.04	0.02	0.364
Phospholipids in medium HDL (mmol/l)	2424	0.00	-0.03	0.02	0.777	2188	-0.01	-0.04	0.01	0.311	1659	-0.01	-0.04	0.02	0.411
Total cholesterol in medium HDL (mmol/l)	2424	0.00	-0.03	0.02	0.818	2188	-0.01	-0.04	0.02	0.350	1659	-0.01	-0.04	0.02	0.435
Cholesterol esters in medium HDL (mmol/l)	2424	0.00	-0.03	0.02	0.831	2188	-0.01	-0.04	0.02	0.369	1659	-0.01	-0.04	0.02	0.472
Free cholesterol in medium HDL (mmol/l)	2424	0.00	-0.03	0.02	0.730	2188	-0.02	-0.04	0.01	0.291	1659	-0.02	-0.05	0.01	0.304
Triglycerides in medium HDL (mmol/l)	2424	-0.02	-0.05	0.01	0.129	2188	-0.02	-0.04	0.01	0.280	1659	-0.02	-0.05	0.01	0.226
Concentration of small HDL particles (mol/l)	2424	-0.01	-0.04	0.02	0.431	2188	-0.01	-0.04	0.02	0.361	1659	-0.02	-0.05	0.01	0.253
Total lipids in small HDL (mmol/l)	2424	-0.01	-0.03	0.02	0.513	2188	-0.02	-0.04	0.01	0.263	1659	-0.02	-0.05	0.01	0.212
Phospholipids in small HDL (mmol/l)	2424	-0.01	-0.04	0.02	0.512	2188	-0.01	-0.04	0.02	0.457	1659	-0.02	-0.05	0.02	0.343

S5 Table Observational associations of age at puberty onset (per year later) with adiposity and cardiometabolic traits at age 18y among females and males in ALSPAC

	Adj. for age, sex, education					*Adj. for age, sex, education, BMI at age 8y*					*Adj. for age, sex, education, outcome value at age 8y*				
**Standardised outcome at age 18y**	**N**	**Beta**	**LCL**	**UCL**	**P-value**	**N**	**Beta**	**LCL**	**UCL**	**P-value**	**N**	**Beta**	**LCL**	**UCL**	**P-value**
Total cholesterol in small HDL (mmol/l)	2424	0.00	-0.03	0.02	0.702	2188	-0.02	-0.04	0.01	0.188	1659	-0.02	-0.05	0.01	0.163
Cholesterol esters in small HDL (mmol/l)	2424	0.00	-0.03	0.02	0.679	2188	-0.02	-0.04	0.01	0.181	1659	-0.02	-0.05	0.01	0.151
Free cholesterol in small HDL (mmol/l)	2424	0.00	-0.03	0.03	0.884	2188	-0.01	-0.04	0.02	0.450	1659	-0.01	-0.05	0.02	0.356
Triglycerides in small HDL (mmol/l)	2424	-0.01	-0.04	0.02	0.477	2188	0.00	-0.03	0.03	0.845	1659	-0.01	-0.04	0.03	0.615
Phospholipids to total lipds ratio in chylomicrons and extremely large VLDL (%)	2424	0.02	-0.02	0.06	0.257	2188	0.03	-0.02	0.07	0.238	1659	0.03	-0.02	0.09	0.234
Total cholesterol to total lipids ratio in chylomicrons and extremely large VLDL (%)	2424	-0.02	-0.04	0.01	0.288	2188	-0.01	-0.04	0.02	0.368	1659	0.00	-0.04	0.03	0.907
Cholesterol esters to total lipids ratio in chylomicrons and extremely large VLDL (%)	2424	-0.01	-0.04	0.02	0.464	2188	-0.01	-0.04	0.02	0.490	1659	-0.01	-0.04	0.03	0.751
Free cholesterol to total lipids ratio in chylomicrons and extremely large VLDL (%)	2424	0.00	-0.03	0.03	0.856	2188	0.00	-0.03	0.03	0.752	1659	0.02	-0.02	0.05	0.359
Triglycerides to total lipids ratio in chylomicrons and extremely large VLDL (%)	2424	0.01	-0.02	0.04	0.520	2188	0.01	-0.02	0.04	0.697	1659	0.00	-0.03	0.04	0.970
Phospholipids to total lipds ratio in very large VLDL (%)	2424	0.01	-0.02	0.04	0.681	2188	0.01	-0.02	0.05	0.431	1659	0.03	-0.01	0.07	0.173
Total cholesterol to total lipids ratio in very large VLDL (%)	2424	0.01	-0.02	0.03	0.613	2188	0.01	-0.02	0.04	0.626	1659	0.00	-0.03	0.04	0.901
Cholesterol esters to total lipids ratio in very large VLDL (%)	2424	-0.02	-0.06	0.02	0.402	2188	-0.02	-0.06	0.02	0.372	1659	-0.03	-0.09	0.03	0.289
Free cholesterol to total lipids ratio in very large VLDL (%)	2424	0.00	-0.05	0.04	0.834	2188	0.00	-0.05	0.04	0.843	1659	-0.01	-0.07	0.06	0.866
Triglycerides to total lipids ratio in very large VLDL (%)	2424	-0.01	-0.04	0.02	0.445	2188	-0.01	-0.05	0.02	0.378	1659	-0.02	-0.06	0.02	0.247
Phospholipids to total lipds ratio in large VLDL (%)	2424	0.01	-0.03	0.05	0.583	2188	0.02	-0.02	0.06	0.362	1659	0.03	-0.02	0.08	0.197
Total cholesterol to total lipids ratio in large VLDL (%)	2424	0.00	-0.02	0.03	0.839	2188	0.01	-0.02	0.04	0.514	1659	0.02	-0.01	0.06	0.192
Cholesterol esters to total lipids ratio in large VLDL (%)	2424	0.01	-0.02	0.04	0.500	2188	0.01	-0.01	0.04	0.334	1659	0.02	-0.01	0.05	0.243
Free cholesterol to total lipids ratio in large VLDL (%)	2424	0.00	-0.01	0.01	0.633	2188	0.00	-0.01	0.01	0.951	1659	0.00	-0.01	0.02	0.713
Triglycerides to total lipids ratio in large VLDL (%)	2424	-0.01	-0.03	0.02	0.663	2188	-0.01	-0.04	0.02	0.361	1659	-0.03	-0.07	0.00	0.069
Phospholipids to total lipds ratio in medium VLDL (%)	2424	0.02	-0.01	0.04	0.193	2188	0.01	-0.02	0.04	0.638	1659	0.02	-0.01	0.05	0.263
Total cholesterol to total lipids ratio in medium VLDL (%)	2424	0.00	-0.03	0.03	0.896	2188	0.00	-0.03	0.03	0.893	1659	0.01	-0.02	0.04	0.582
Cholesterol esters to total lipids ratio in medium VLDL (%)	2424	0.00	-0.03	0.03	0.905	2188	0.00	-0.03	0.03	0.860	1659	0.01	-0.03	0.04	0.673
Free cholesterol to total lipids ratio in medium VLDL (%)	2424	0.00	-0.03	0.03	0.938	2188	0.00	-0.03	0.03	0.951	1659	0.01	-0.03	0.04	0.784
Triglycerides to total lipids ratio in medium VLDL (%)	2424	0.00	-0.03	0.03	0.968	2188	0.00	-0.03	0.03	0.900	1659	-0.01	-0.04	0.02	0.531
Phospholipids to total lipds ratio in small VLDL (%)	2424	0.00	-0.02	0.03	0.737	2188	-0.01	-0.04	0.02	0.453	1659	-0.02	-0.05	0.02	0.353
Total cholesterol to total lipids ratio in small VLDL (%)	2424	0.00	-0.02	0.03	0.752	2188	0.00	-0.03	0.03	0.990	1659	0.00	-0.03	0.04	0.861
Cholesterol esters to total lipids ratio in small VLDL (%)	2424	0.00	-0.03	0.03	0.833	2188	0.00	-0.03	0.03	0.941	1659	0.00	-0.03	0.04	0.788
Free cholesterol to total lipids ratio in small VLDL (%)	2424	0.01	-0.02	0.04	0.457	2188	-0.01	-0.04	0.02	0.547	1659	-0.01	-0.04	0.02	0.359
Triglycerides to total lipids ratio in small VLDL (%)	2424	-0.01	-0.04	0.02	0.621	2188	0.00	-0.03	0.03	0.879	1659	0.00	-0.04	0.04	0.997
Phospholipids to total lipds ratio in very small VLDL (%)	2424	0.00	-0.03	0.02	0.752	2188	-0.01	-0.04	0.02	0.390	1659	-0.02	-0.04	0.01	0.249
Total cholesterol to total lipids ratio in very small VLDL (%)	2424	0.01	-0.02	0.03	0.642	2188	0.01	-0.02	0.04	0.595	1659	0.01	-0.03	0.04	0.744
Cholesterol esters to total lipids ratio in very small VLDL (%)	2424	0.00	-0.03	0.03	0.980	2188	0.01	-0.02	0.04	0.699	1659	0.01	-0.03	0.04	0.735
Free cholesterol to total lipids ratio in very small VLDL (%)	2424	0.02	-0.01	0.05	0.110	2188	0.01	-0.02	0.04	0.488	1659	0.01	-0.02	0.04	0.514
Triglycerides to total lipids ratio in very small VLDL (%)	2424	-0.01	-0.04	0.02	0.690	2188	0.00	-0.04	0.03	0.872	1659	0.00	-0.04	0.04	0.914
Phospholipids to total lipds ratio in IDL (%)	2424	-0.01	-0.04	0.02	0.467	2188	-0.03	-0.06	0.01	0.129	1659	-0.01	-0.05	0.03	0.539
Total cholesterol to total lipids ratio in IDL (%)	2424	0.00	-0.03	0.03	0.923	2188	0.01	-0.02	0.04	0.623	1659	0.00	-0.04	0.03	0.865
Cholesterol esters to total lipids ratio in IDL (%)	2424	0.00	-0.03	0.03	0.970	2188	0.01	-0.02	0.05	0.359	1659	0.00	-0.03	0.04	0.826
Free cholesterol to total lipids ratio in IDL (%)	2424	0.00	-0.03	0.03	0.899	2188	-0.01	-0.05	0.02	0.374	1659	-0.02	-0.06	0.02	0.287
Triglycerides to total lipids ratio in IDL (%)	2424	0.00	-0.03	0.03	0.860	2188	0.00	-0.03	0.03	0.971	1659	0.01	-0.03	0.05	0.586
Phospholipids to total lipds ratio in large LDL (%)	2424	0.00	-0.03	0.03	0.839	2188	0.01	-0.03	0.04	0.716	1659	0.02	-0.01	0.05	0.111
Total cholesterol to total lipids ratio in large LDL (%)	2424	0.00	-0.03	0.03	0.810	2188	0.00	-0.04	0.03	0.826	1659	-0.03	-0.06	0.00	0.091
Cholesterol esters to total lipids ratio in large LDL (%)	2424	-0.01	-0.04	0.02	0.704	2188	0.00	-0.04	0.03	0.897	1659	-0.02	-0.05	0.00	0.083
Free cholesterol to total lipids ratio in large LDL (%)	2424	0.01	-0.02	0.04	0.590	2188	0.00	-0.04	0.03	0.871	1659	0.00	-0.04	0.04	0.965
Triglycerides to total lipids ratio in large LDL (%)	2424	0.00	-0.03	0.03	0.886	2188	0.00	-0.03	0.03	0.948	1659	0.01	-0.02	0.05	0.453
Phospholipids to total lipds ratio in medium LDL (%)	2424	0.00	-0.03	0.03	0.885	2188	0.00	-0.03	0.03	0.996	1659	0.01	-0.02	0.05	0.353
Total cholesterol to total lipids ratio in medium LDL (%)	2424	0.00	-0.04	0.03	0.871	2188	0.00	-0.04	0.03	0.860	1659	-0.03	-0.06	0.01	0.144
Cholesterol esters to total lipids ratio in medium LDL (%)	2424	0.00	-0.03	0.03	0.900	2188	0.00	-0.03	0.03	0.956	1659	-0.02	-0.05	0.01	0.162
Free cholesterol to total lipids ratio in medium LDL (%)	2424	0.00	-0.03	0.03	0.957	2188	0.00	-0.03	0.03	0.893	1659	0.01	-0.03	0.04	0.778
Triglycerides to total lipids ratio in medium LDL (%)	2424	0.01	-0.03	0.05	0.641	2188	0.01	-0.04	0.05	0.783	1659	0.02	-0.03	0.08	0.440
Phospholipids to total lipds ratio in small LDL (%)	2424	0.01	-0.03	0.04	0.706	2188	0.01	-0.03	0.04	0.679	1659	0.03	-0.01	0.06	0.101

S5 Table Observational associations of age at puberty onset (per year later) with adiposity and cardiometabolic traits at age 18y among females and males in ALSPAC

	Adj. for age, sex, education					*Adj. for age, sex, education, BMI at age 8y*					*Adj. for age, sex, education, outcome value at age 8y*				
**Standardised outcome at age 18y**	**N**	**Beta**	**LCL**	**UCL**	**P-value**	**N**	**Beta**	**LCL**	**UCL**	**P-value**	**N**	**Beta**	**LCL**	**UCL**	**P-value**
Total cholesterol to total lipids ratio in small LDL (%)	2424	0.00	-0.03	0.03	0.954	2188	0.00	-0.04	0.03	0.850	1659	-0.03	-0.06	0.01	0.140
Cholesterol esters to total lipids ratio in small LDL (%)	2424	0.00	-0.03	0.03	0.869	2188	0.00	-0.03	0.03	0.847	1659	-0.02	-0.05	0.01	0.162
Free cholesterol to total lipids ratio in small LDL (%)	2424	0.00	-0.02	0.03	0.786	2188	0.00	-0.03	0.03	0.932	1659	0.00	-0.03	0.04	0.800
Triglycerides to total lipids ratio in small LDL (%)	2424	-0.01	-0.04	0.02	0.435	2188	-0.01	-0.04	0.02	0.630	1659	0.00	-0.04	0.04	0.922
Phospholipids to total lipds ratio in very large HDL (%)	2424	-0.01	-0.04	0.02	0.610	2188	-0.02	-0.05	0.01	0.112	1659	-0.01	-0.04	0.02	0.693
Total cholesterol to total lipids ratio in very large HDL (%)	2424	0.01	-0.02	0.04	0.497	2188	0.03	0.00	0.05	0.098	1659	0.00	-0.02	0.03	0.754
Cholesterol esters to total lipids ratio in very large HDL (%)	2424	0.01	-0.02	0.04	0.473	2188	0.03	0.00	0.06	0.100	1659	0.01	-0.02	0.04	0.686
Free cholesterol to total lipids ratio in very large HDL (%)	2424	-0.01	-0.04	0.02	0.678	2188	-0.01	-0.04	0.03	0.761	1659	-0.01	-0.05	0.02	0.482
Triglycerides to total lipids ratio in very large HDL (%)	2424	-0.01	-0.04	0.02	0.523	2188	0.00	-0.03	0.03	0.888	1659	0.00	-0.03	0.04	0.841
Phospholipids to total lipds ratio in large HDL (%)	2424	-0.02	-0.05	0.00	0.101	2188	-0.01	-0.04	0.02	0.417	1659	-0.02	-0.06	0.01	0.129
Total cholesterol to total lipids ratio in large HDL (%)	2424	0.02	-0.01	0.05	0.162	2188	0.01	-0.02	0.04	0.686	1659	0.02	-0.01	0.05	0.271
Cholesterol esters to total lipids ratio in large HDL (%)	2424	0.02	-0.01	0.05	0.184	2188	0.01	-0.02	0.04	0.703	1659	0.02	-0.01	0.05	0.239
Free cholesterol to total lipids ratio in large HDL (%)	2424	0.02	-0.01	0.05	0.182	2188	0.01	-0.02	0.04	0.651	1659	0.01	-0.02	0.04	0.544
Triglycerides to total lipids ratio in large HDL (%)	2424	-0.01	-0.04	0.02	0.499	2188	0.01	-0.02	0.03	0.693	1659	0.00	-0.03	0.03	0.871
Phospholipids to total lipds ratio in medium HDL (%)	2424	0.00	-0.02	0.03	0.778	2188	0.00	-0.03	0.03	0.975	1659	0.00	-0.03	0.03	0.935
Total cholesterol to total lipids ratio in medium HDL (%)	2424	0.01	-0.02	0.03	0.621	2188	0.00	-0.03	0.03	0.762	1659	0.01	-0.03	0.04	0.724
Cholesterol esters to total lipids ratio in medium HDL (%)	2424	0.01	-0.02	0.04	0.581	2188	0.01	-0.02	0.04	0.679	1659	0.01	-0.02	0.04	0.578
Free cholesterol to total lipids ratio in medium HDL (%)	2424	0.00	-0.05	0.04	0.847	2188	-0.01	-0.06	0.04	0.712	1659	-0.02	-0.09	0.04	0.529
Triglycerides to total lipids ratio in medium HDL (%)	2424	-0.02	-0.05	0.01	0.166	2188	-0.01	-0.04	0.02	0.598	1659	-0.01	-0.04	0.02	0.521
Phospholipids to total lipds ratio in small HDL (%)	2424	0.00	-0.02	0.03	0.972	2188	0.01	-0.02	0.04	0.424	1659	0.01	-0.02	0.04	0.388
Total cholesterol to total lipids ratio in small HDL (%)	2424	0.00	-0.02	0.03	0.906	2188	-0.01	-0.04	0.02	0.385	1659	-0.01	-0.04	0.02	0.418
Cholesterol esters to total lipids ratio in small HDL (%)	2424	0.00	-0.03	0.02	0.922	2188	-0.01	-0.04	0.01	0.364	1659	-0.01	-0.04	0.02	0.372
Free cholesterol to total lipids ratio in small HDL (%)	2424	0.02	-0.01	0.05	0.218	2188	0.01	-0.02	0.04	0.589	1659	0.01	-0.02	0.05	0.450
Triglycerides to total lipids ratio in small HDL (%)	2424	-0.01	-0.04	0.02	0.671	2188	0.01	-0.02	0.04	0.670	1659	0.00	-0.03	0.04	0.825
Mean diameter for VLDL particles (nm)	2424	-0.01	-0.04	0.02	0.425	2188	0.01	-0.03	0.04	0.732	1659	0.00	-0.03	0.04	0.925
Mean diameter for LDL particles (nm)	2424	0.01	-0.03	0.04	0.723	2188	0.00	-0.03	0.04	0.855	1659	0.02	-0.02	0.06	0.247
Mean diameter for HDL particles (nm)	2424	0.01	-0.02	0.03	0.646	2188	-0.01	-0.04	0.02	0.615	1659	0.01	-0.02	0.03	0.664
Serum total cholesterol (mmol/l)	2424	0.00	-0.03	0.03	0.941	2188	0.00	-0.03	0.02	0.759	1659	-0.02	-0.04	0.01	0.171
Total cholesterol in VLDL (mmol/l)	2424	0.00	-0.03	0.03	0.794	2188	0.01	-0.02	0.04	0.618	1659	0.00	-0.03	0.03	0.875
Remnant cholesterol (non-HDL, non-LDL -cholesterol) (mmol/l)	2424	0.00	-0.03	0.03	0.935	2188	0.00	-0.03	0.03	0.782	1659	-0.01	-0.04	0.02	0.592
Total cholesterol in LDL (mmol/l)	2424	0.00	-0.03	0.03	0.993	2188	0.00	-0.03	0.02	0.748	1659	-0.02	-0.05	0.01	0.152
Total cholesterol in HDL (mmol/l)	2424	0.00	-0.02	0.03	0.759	2188	-0.01	-0.04	0.02	0.472	1659	-0.01	-0.04	0.02	0.487
Total cholesterol in HDL2 (mmol/l)	2424	0.00	-0.02	0.03	0.728	2188	-0.01	-0.04	0.02	0.457	1659	-0.01	-0.04	0.02	0.524
Total cholesterol in HDL3 (mmol/l)	2424	0.00	-0.02	0.03	0.835	2188	-0.01	-0.04	0.02	0.527	1659	-0.01	-0.04	0.02	0.479
Esterified cholesterol (mmol/l)	2414	0.00	-0.02	0.03	0.759	2180	0.00	-0.03	0.03	0.901	1652	-0.01	-0.04	0.01	0.275
Free cholesterol (mmol/l)	2412	-0.01	-0.03	0.02	0.599	2178	-0.01	-0.04	0.02	0.458	1650	-0.02	-0.05	0.01	0.114
Serum total triglycerides (mmol/l)	2424	-0.01	-0.04	0.02	0.599	2188	0.01	-0.03	0.04	0.719	1659	0.00	-0.04	0.03	0.815
Triglycerides in VLDL (mmol/l)	2424	-0.01	-0.04	0.02	0.566	2188	0.01	-0.02	0.04	0.589	1659	0.00	-0.04	0.04	0.899
Triglycerides in LDL (mmol/l)	2424	0.00	-0.02	0.03	0.992	2188	-0.01	-0.03	0.02	0.650	1659	-0.01	-0.03	0.02	0.697
Triglycerides in HDL (mmol/l)	2424	-0.01	-0.04	0.01	0.317	2188	-0.01	-0.04	0.02	0.505	1659	-0.01	-0.04	0.02	0.558
Diacylglycerol (mmol/l)	2357	-0.01	-0.04	0.02	0.648	2132	-0.01	-0.04	0.02	0.681	1582	0.00	-0.04	0.03	0.801
Ratio of diacylglycerol to triglycerides	2358	0.00	-0.03	0.03	0.909	2133	-0.01	-0.04	0.02	0.634	1583	0.00	-0.03	0.03	0.969
Total phosphoglycerides (mmol/l)	2412	-0.01	-0.03	0.02	0.435	2178	-0.01	-0.04	0.01	0.356	1650	-0.02	-0.05	0.01	0.199
Ratio of triglycerides to phosphoglycerides	2412	0.00	-0.03	0.03	0.902	2178	0.02	-0.02	0.05	0.317	1650	0.01	-0.03	0.05	0.681
Phosphatidylcholine and other cholines (mmol/l)	2395	-0.01	-0.03	0.02	0.688	2162	-0.01	-0.04	0.01	0.290	1631	-0.02	-0.05	0.01	0.115
Total cholines (mmol/l)	2414	-0.01	-0.03	0.02	0.475	2180	-0.02	-0.04	0.01	0.253	1652	-0.02	-0.05	0.00	0.094
Apolipoprotein A-I (g/l)	2424	0.00	-0.02	0.03	0.863	2188	-0.01	-0.04	0.02	0.501	1659	-0.01	-0.04	0.01	0.324
Apolipoprotein B (g/l)	2424	0.00	-0.03	0.03	0.839	2188	0.00	-0.03	0.03	0.815	1659	-0.01	-0.04	0.02	0.542
Ratio of apolipoprotein B to apolipoprotein A-I	2424	-0.01	-0.03	0.02	0.738	2188	0.01	-0.02	0.04	0.612	1659	0.00	-0.03	0.03	0.857
Total fatty acids (mmol/l)	2414	0.00	-0.03	0.03	0.924	2180	0.00	-0.03	0.03	0.864	1652	-0.01	-0.04	0.02	0.515

S5 Table Observational associations of age at puberty onset (per year later) with adiposity and cardiometabolic traits at age 18y among females and males in ALSPAC

	Adj. for age, sex, education					*Adj. for age, sex, education, BMI at age 8y*					*Adj. for age, sex, education, outcome value at age 8y*				
**Standardised outcome at age 18y**	**N**	**Beta**	**LCL**	**UCL**	**P-value**	**N**	**Beta**	**LCL**	**UCL**	**P-value**	**N**	**Beta**	**LCL**	**UCL**	**P-value**
Estimated description of fatty acid chain length, not actual carbon number	2414	0.02	-0.01	0.05	0.181	2180	0.02	-0.01	0.06	0.162	1651	0.02	-0.02	0.06	0.280
Estimated degree of unsaturation	2414	0.02	-0.01	0.05	0.213	2180	0.01	-0.02	0.05	0.413	1652	0.01	-0.03	0.05	0.565
22:6, docosahexaenoic acid (mmol/l)	2414	0.01	-0.01	0.04	0.283	2180	0.01	-0.01	0.04	0.338	1652	0.00	-0.03	0.03	0.908
18:2, linoleic acid (mmol/l)	2413	0.01	-0.02	0.03	0.688	2179	0.00	-0.03	0.03	0.977	1651	-0.01	-0.04	0.02	0.465
Conjugated linoleic acid (mmol/l)	2413	-0.01	-0.03	0.02	0.704	2179	0.00	-0.03	0.03	0.956	1649	-0.01	-0.05	0.03	0.683
Omega-3 fatty acids (mmol/l)	2414	0.01	-0.02	0.04	0.508	2180	0.02	-0.01	0.05	0.224	1652	0.00	-0.03	0.03	0.951
Omega-6 fatty acids (mmol/l)	2414	0.00	-0.02	0.03	0.884	2180	0.00	-0.03	0.03	0.965	1652	-0.01	-0.04	0.02	0.418
Polyunsaturated fatty acids (mmol/l)	2413	0.00	-0.02	0.03	0.868	2179	0.00	-0.03	0.03	0.942	1651	-0.01	-0.04	0.02	0.396
Monounsaturated fatty acids; 16:1, 18:1 (mmol/l)	2414	0.00	-0.03	0.02	0.805	2180	0.00	-0.03	0.03	0.886	1651	-0.01	-0.04	0.02	0.544
Saturated fatty acids (mmol/l)	2413	0.00	-0.03	0.03	0.848	2179	0.00	-0.03	0.03	0.854	1650	-0.01	-0.04	0.02	0.583
Ratio of 22:6 docosahexaenoic acid to total fatty acids (%)	2415	0.02	-0.01	0.05	0.189	2181	0.02	-0.01	0.05	0.232	1653	0.02	-0.02	0.05	0.347
Ratio of 18:2 linoleic acid to total fatty acids (%)	2414	0.01	-0.02	0.04	0.404	2180	0.00	-0.04	0.03	0.834	1652	0.00	-0.03	0.04	0.850
Ratio of conjugated linoleic acid to total fatty acids (%)	2414	0.00	-0.03	0.03	0.871	2180	0.00	-0.03	0.03	0.871	1650	0.00	-0.04	0.04	0.891
Ratio of omega-3 fatty acids to total fatty acids (%)	2415	0.01	-0.02	0.04	0.402	2181	0.02	-0.01	0.05	0.169	1653	0.01	-0.02	0.05	0.493
Ratio of omega-6 fatty acids to total fatty acids (%)	2415	0.01	-0.02	0.04	0.656	2181	-0.01	-0.04	0.03	0.701	1653	0.00	-0.03	0.04	0.805
Ratio of polyunsaturated fatty acids to total fatty acids (%)	2414	0.01	-0.02	0.04	0.530	2180	0.00	-0.03	0.03	0.976	1652	0.01	-0.03	0.05	0.658
Ratio of monounsaturated fatty acids to total fatty acids (%)	2415	-0.01	-0.04	0.02	0.710	2181	0.00	-0.03	0.03	0.970	1652	0.00	-0.04	0.03	0.790
Ratio of saturated fatty acids to total fatty acids (%)	2414	0.00	-0.04	0.03	0.783	2180	0.00	-0.03	0.03	0.996	1651	-0.01	-0.04	0.03	0.776
Glucose (mmol/l)	2423	0.01	-0.02	0.04	0.546	2187	0.01	-0.02	0.05	0.412	1649	-0.01	-0.04	0.02	0.573
Lactate (mmol/l)	2423	-0.03	-0.06	0.00	0.079	2187	-0.02	-0.06	0.01	0.203	1659	-0.01	-0.04	0.03	0.658
Pyruvate (mmol/l)	2423	-0.04	-0.07	-0.01	0.011	2187	-0.02	-0.06	0.01	0.169	1656	-0.02	-0.06	0.01	0.231
Citrate (mmol/l)	2423	0.04	0.01	0.07	0.007	2187	0.02	-0.01	0.05	0.263	1656	0.03	0.00	0.07	0.069
Alanine (mmol/l)	2423	-0.01	-0.04	0.02	0.338	2187	-0.01	-0.04	0.02	0.534	1659	0.00	-0.03	0.04	0.874
Glutamine (mmol/l)	2423	0.03	0.00	0.06	0.020	2187	0.03	0.00	0.05	0.072	1656	0.03	0.00	0.07	0.028
Histidine (mmol/l)	2423	-0.01	-0.04	0.02	0.564	2187	-0.01	-0.04	0.02	0.642	1657	0.00	-0.04	0.03	0.962
Isoleucine (mmol/l)	2423	-0.01	-0.04	0.02	0.688	2187	0.02	-0.02	0.05	0.352	1659	0.02	-0.02	0.06	0.265
Leucine (mmol/l)	2423	0.01	-0.02	0.03	0.732	2187	0.02	-0.01	0.05	0.193	1659	0.02	-0.01	0.05	0.246
Valine (mmol/l)	2423	-0.01	-0.04	0.02	0.659	2187	0.02	-0.02	0.05	0.325	1659	0.01	-0.02	0.05	0.444
Phenylalanine (mmol/l)	2422	-0.03	-0.06	0.00	0.041	2186	-0.03	-0.06	0.00	0.080	1654	-0.03	-0.07	0.00	0.061
Tyrosine (mmol/l)	2423	-0.03	-0.06	0.00	0.040	2187	-0.01	-0.04	0.01	0.327	1652	-0.01	-0.05	0.02	0.413
Acetate (mmol/l)	2422	-0.01	-0.04	0.02	0.416	2186	-0.02	-0.05	0.01	0.181	1659	-0.01	-0.04	0.02	0.581
Acetoacetate (mmol/l)	2423	0.01	-0.02	0.04	0.423	2187	0.01	-0.02	0.04	0.491	1658	0.00	-0.03	0.04	0.792
3-hydroxybutyrate (mmol/l)	2421	0.02	0.00	0.05	0.115	2185	0.01	-0.01	0.04	0.309	1657	0.02	-0.02	0.05	0.300
Creatinine (mmol/l)	2423	-0.03	-0.06	-0.01	0.008	2187	-0.03	-0.05	0.00	0.051	1655	-0.03	-0.06	-0.01	0.018
Albumin (signal area)	2424	0.00	-0.02	0.03	0.754	2188	0.00	-0.03	0.03	0.931	1657	0.02	-0.01	0.05	0.274
Glycoprotein acetyls, mainly a1-acid glycoprotein (mmol/l)	2423	-0.02	-0.05	0.01	0.163	2187	0.00	-0.03	0.03	0.987	1659	0.01	-0.03	0.04	0.702

Complete case sample

	Adj. for age, education					*Adj. for age, education, BMI at age 8y*					*Adj. for age, sex, education, outcome value at age 8y*				
**Standardised outcome at age 18y**	**N**	**Beta**	**LCL**	**UCL**	**P-value**	**N**	**Beta**	**LCL**	**UCL**	**P-value**	**N**	**Beta**	**LCL**	**UCL**	**P-value**
Body mass index (kg/m^2)	1269	-0.11	-0.14	-0.07	1.90E-09	1269	-0.04	-0.07	-0.01	0.006	1269	-0.04	-0.07	-0.01	0.006
Fat mass index (kg/m^2)	1269	-0.09	-0.12	-0.06	6.79E-08	1269	-0.03	-0.06	-0.01	0.012	1269	-0.01	-0.04	0.01	0.309
Lean mass index (kg/m^2)	1269	-0.04	-0.07	-0.02	9.35E-04	1269	-0.01	-0.04	0.01	0.316	1269	0.00	-0.02	0.02	0.969
Systolic blood pressure (mmHg)	1269	-0.05	-0.09	-0.02	0.004	1269	-0.03	-0.07	0.00	0.056	1269	-0.03	-0.06	0.01	0.123
Diastolic blood pressure (mmHg)	1269	-0.06	-0.10	-0.02	1.16E-03	1269	-0.05	-0.08	-0.01	0.018	1269	-0.05	-0.08	-0.01	0.010
Concentration of chylomicrons and extremely large VLDL particles (mol/l)	1269	0.00	-0.04	0.04	0.931	1269	0.02	-0.02	0.06	0.329	1269	0.00	-0.04	0.05	0.825
Total lipids in chylomicrons and extremely large VLDL (mmol/l)	1269	0.00	-0.04	0.04	0.935	1269	0.02	-0.02	0.06	0.338	1269	0.00	-0.04	0.05	0.829
Phospholipids in chylomicrons and extremely large VLDL (mmol/l)	1269	0.00	-0.04	0.04	0.914	1269	0.02	-0.02	0.06	0.328	1269	0.00	-0.04	0.05	0.824

S5 Table Observational associations of age at puberty onset (per year later) with adiposity and cardiometabolic traits at age 18y among females and males in ALSPAC

	Adj. for age, sex, education					*Adj. for age, sex, education, BMI at age 8y*					*Adj. for age, sex, education, outcome value at age 8y*				
**Standardised outcome at age 18y**	**N**	**Beta**	**LCL**	**UCL**	**P-value**	**N**	**Beta**	**LCL**	**UCL**	**P-value**	**N**	**Beta**	**LCL**	**UCL**	**P-value**
Total cholesterol in chylomicrons and extremely large VLDL (mmol/l)	1269	0.00	-0.04	0.04	0.953	1269	0.02	-0.02	0.06	0.366	1269	0.00	-0.04	0.05	0.826
Cholesterol esters in chylomicrons and extremely large VLDL (mmol/l)	1269	0.00	-0.04	0.04	0.996	1269	0.02	-0.03	0.06	0.415	1269	0.00	-0.04	0.04	0.858
Free cholesterol in chylomicrons and extremely large VLDL (mmol/l)	1269	0.00	-0.04	0.04	0.902	1269	0.02	-0.02	0.06	0.323	1269	0.01	-0.04	0.05	0.799
Triglycerides in chylomicrons and extremely large VLDL (mmol/l)	1269	0.00	-0.04	0.04	0.930	1269	0.02	-0.02	0.06	0.332	1269	0.00	-0.04	0.05	0.827
Concentration of very large VLDL particles (mol/l)	1269	0.00	-0.04	0.04	0.872	1269	0.02	-0.02	0.07	0.310	1269	0.01	-0.03	0.05	0.763
Total lipids in very large VLDL (mmol/l)	1269	0.00	-0.04	0.04	0.876	1269	0.02	-0.02	0.06	0.317	1269	0.01	-0.03	0.05	0.772
Phospholipids in very large VLDL (mmol/l)	1269	0.00	-0.04	0.04	0.863	1269	0.02	-0.02	0.06	0.311	1269	0.01	-0.04	0.05	0.782
Total cholesterol in very large VLDL (mmol/l)	1269	0.00	-0.04	0.04	0.920	1269	0.02	-0.02	0.06	0.330	1269	0.01	-0.04	0.05	0.788
Cholesterol esters in very large VLDL (mmol/l)	1269	0.00	-0.04	0.04	0.934	1269	0.02	-0.02	0.06	0.337	1269	0.01	-0.04	0.05	0.773
Free cholesterol in very large VLDL (mmol/l)	1269	0.00	-0.04	0.04	0.907	1269	0.02	-0.02	0.06	0.326	1269	0.01	-0.04	0.05	0.803
Triglycerides in very large VLDL (mmol/l)	1269	0.00	-0.04	0.04	0.865	1269	0.02	-0.02	0.06	0.317	1269	0.01	-0.03	0.05	0.763
Concentration of large VLDL particles (mol/l)	1269	0.00	-0.04	0.04	0.908	1269	0.02	-0.02	0.06	0.354	1269	0.01	-0.04	0.05	0.796
Total lipids in large VLDL (mmol/l)	1269	0.00	-0.04	0.04	0.861	1269	0.02	-0.02	0.06	0.324	1269	0.01	-0.03	0.05	0.748
Phospholipids in large VLDL (mmol/l)	1269	0.01	-0.03	0.05	0.640	1269	0.03	-0.02	0.07	0.216	1269	0.01	-0.03	0.05	0.554
Total cholesterol in large VLDL (mmol/l)	1269	0.00	-0.04	0.04	0.899	1269	0.02	-0.02	0.06	0.340	1269	0.01	-0.03	0.05	0.776
Cholesterol esters in large VLDL (mmol/l)	1269	0.00	-0.04	0.04	0.938	1269	0.02	-0.02	0.06	0.363	1269	0.01	-0.03	0.05	0.785
Free cholesterol in large VLDL (mmol/l)	1269	0.00	-0.04	0.04	0.860	1269	0.02	-0.02	0.06	0.322	1269	0.01	-0.03	0.05	0.763
Triglycerides in large VLDL (mmol/l)	1269	0.00	-0.04	0.04	0.922	1269	0.02	-0.02	0.06	0.367	1269	0.01	-0.04	0.05	0.808
Concentration of medium VLDL particles (mol/l)	1269	0.00	-0.04	0.04	0.939	1269	0.02	-0.02	0.06	0.371	1269	0.01	-0.03	0.04	0.801
Total lipids in medium VLDL (mmol/l)	1269	0.00	-0.04	0.04	0.941	1269	0.02	-0.02	0.06	0.371	1269	0.01	-0.03	0.04	0.797
Phospholipids in medium VLDL (mmol/l)	1269	0.00	-0.04	0.04	0.904	1269	0.02	-0.02	0.06	0.356	1269	0.01	-0.03	0.05	0.785
Total cholesterol in medium VLDL (mmol/l)	1269	0.00	-0.04	0.04	0.966	1269	0.02	-0.02	0.06	0.402	1269	0.00	-0.03	0.04	0.811
Cholesterol esters in medium VLDL (mmol/l)	1269	0.00	-0.04	0.04	0.976	1269	0.02	-0.02	0.06	0.421	1269	0.01	-0.03	0.04	0.791
Free cholesterol in medium VLDL (mmol/l)	1269	0.00	-0.04	0.04	0.956	1269	0.02	-0.02	0.06	0.401	1269	0.00	-0.04	0.04	0.848
Triglycerides in medium VLDL (mmol/l)	1269	0.00	-0.04	0.04	0.945	1269	0.02	-0.02	0.06	0.373	1269	0.01	-0.03	0.04	0.800
Concentration of small VLDL particles (mol/l)	1269	0.00	-0.04	0.04	0.995	1269	0.02	-0.03	0.06	0.470	1269	0.00	-0.04	0.04	0.945
Total lipids in small VLDL (mmol/l)	1269	0.00	-0.04	0.04	0.949	1269	0.01	-0.03	0.05	0.512	1269	0.00	-0.04	0.03	0.832
Phospholipids in small VLDL (mmol/l)	1269	0.00	-0.04	0.04	0.951	1269	0.01	-0.03	0.05	0.569	1269	-0.01	-0.04	0.03	0.700
Total cholesterol in small VLDL (mmol/l)	1269	0.00	-0.05	0.04	0.813	1269	0.01	-0.03	0.05	0.661	1269	-0.01	-0.04	0.02	0.563
Cholesterol esters in small VLDL (mmol/l)	1269	-0.01	-0.05	0.03	0.755	1269	0.01	-0.03	0.05	0.731	1269	-0.01	-0.05	0.02	0.538
Free cholesterol in small VLDL (mmol/l)	1269	0.00	-0.04	0.04	0.958	1269	0.01	-0.03	0.05	0.558	1269	-0.01	-0.04	0.03	0.712
Triglycerides in small VLDL (mmol/l)	1269	0.00	-0.04	0.04	0.921	1269	0.02	-0.02	0.06	0.425	1269	0.00	-0.04	0.04	0.917
Concentration of very small VLDL particles (mol/l)	1269	0.00	-0.04	0.04	0.927	1269	0.01	-0.03	0.05	0.712	1269	-0.01	-0.04	0.02	0.469
Total lipids in very small VLDL (mmol/l)	1269	0.00	-0.04	0.04	0.845	1269	0.01	-0.03	0.05	0.751	1269	-0.01	-0.05	0.02	0.423
Phospholipids in very small VLDL (mmol/l)	1269	0.00	-0.04	0.03	0.865	1269	0.00	-0.03	0.04	0.827	1269	-0.02	-0.05	0.02	0.322
Total cholesterol in very small VLDL (mmol/l)	1269	-0.01	-0.05	0.03	0.752	1269	0.00	-0.04	0.05	0.871	1269	-0.01	-0.05	0.03	0.564
Cholesterol esters in very small VLDL (mmol/l)	1269	-0.01	-0.05	0.03	0.720	1269	0.00	-0.04	0.05	0.862	1269	-0.01	-0.05	0.03	0.581
Free cholesterol in very small VLDL (mmol/l)	1269	0.00	-0.04	0.04	0.855	1269	0.00	-0.04	0.04	0.906	1269	-0.01	-0.04	0.03	0.621
Triglycerides in very small VLDL (mmol/l)	1269	0.00	-0.03	0.04	0.864	1269	0.01	-0.03	0.05	0.516	1269	0.00	-0.04	0.04	0.931
Concentration of IDL particles (mol/l)	1269	0.00	-0.04	0.03	0.798	1269	0.00	-0.04	0.04	0.937	1269	-0.02	-0.05	0.01	0.277
Total lipids in IDL (mmol/l)	1269	-0.01	-0.04	0.03	0.787	1269	0.00	-0.04	0.04	0.941	1269	-0.02	-0.05	0.01	0.242
Phospholipids in IDL (mmol/l)	1269	-0.01	-0.04	0.03	0.740	1269	0.00	-0.04	0.04	0.954	1269	-0.02	-0.05	0.01	0.203
Total cholesterol in IDL (mmol/l)	1269	-0.01	-0.05	0.03	0.750	1269	0.00	-0.04	0.04	0.953	1269	-0.02	-0.05	0.01	0.258
Cholesterol esters in IDL (mmol/l)	1269	-0.01	-0.05	0.03	0.760	1269	0.00	-0.04	0.04	0.893	1269	-0.02	-0.05	0.02	0.326
Free cholesterol in IDL (mmol/l)	1269	-0.01	-0.04	0.03	0.736	1269	0.00	-0.04	0.04	0.899	1269	-0.02	-0.05	0.01	0.184
Triglycerides in IDL (mmol/l)	1269	0.01	-0.03	0.04	0.772	1269	0.01	-0.03	0.04	0.668	1269	0.00	-0.03	0.03	0.950
Concentration of large LDL particles (mol/l)	1269	-0.01	-0.05	0.03	0.644	1269	0.00	-0.04	0.04	0.888	1269	-0.02	-0.05	0.01	0.161
Total lipids in large LDL (mmol/l)	1269	-0.01	-0.04	0.03	0.700	1269	0.00	-0.04	0.04	0.959	1269	-0.02	-0.05	0.01	0.173
Phospholipids in large LDL (mmol/l)	1269	-0.01	-0.05	0.03	0.689	1269	0.00	-0.04	0.04	0.972	1269	-0.02	-0.05	0.01	0.165
Total cholesterol in large LDL (mmol/l)	1269	-0.01	-0.05	0.03	0.668	1269	0.00	-0.04	0.04	0.931	1269	-0.02	-0.05	0.01	0.146

S5 Table Observational associations of age at puberty onset (per year later) with adiposity and cardiometabolic traits at age 18y among females and males in ALSPAC

Standardised outcome at age 18y	*Adj. for age, sex, education* N	Beta	LCL	UCL	P-value	*Adj. for age, sex, education, BMI at age 8y* N	Beta	LCL	UCL	P-value	*Adj. for age, sex, education, outcome value at age 8y* N	Beta	LCL	UCL	P-value
Cholesterol esters in large LDL (mmol/l)	1269	-0.01	-0.05	0.03	0.665	1269	0.00	-0.04	0.04	0.954	1269	-0.02	-0.05	0.01	0.151
Free cholesterol in large LDL (mmol/l)	1269	-0.01	-0.05	0.03	0.686	1269	0.00	-0.04	0.04	0.867	1269	-0.02	-0.05	0.01	0.147
Triglycerides in large LDL (mmol/l)	1269	0.00	-0.03	0.04	0.889	1269	0.00	-0.03	0.04	0.826	1269	0.00	-0.03	0.03	0.872
Concentration of medium LDL particles (mol/l)	1269	-0.01	-0.05	0.03	0.578	1269	0.00	-0.04	0.03	0.844	1269	-0.02	-0.06	0.01	0.155
Total lipids in medium LDL (mmol/l)	1269	-0.01	-0.05	0.03	0.660	1269	0.00	-0.04	0.04	0.935	1269	-0.02	-0.05	0.01	0.146
Phospholipids in medium LDL (mmol/l)	1269	-0.01	-0.05	0.03	0.670	1269	0.00	-0.04	0.04	0.991	1269	-0.02	-0.05	0.01	0.183
Total cholesterol in medium LDL (mmol/l)	1269	-0.01	-0.05	0.03	0.622	1269	0.00	-0.04	0.04	0.893	1269	-0.03	-0.06	0.01	0.114
Cholesterol esters in medium LDL (mmol/l)	1269	-0.01	-0.05	0.03	0.641	1269	0.00	-0.04	0.04	0.921	1269	-0.02	-0.06	0.01	0.130
Free cholesterol in medium LDL (mmol/l)	1269	-0.01	-0.05	0.03	0.555	1269	-0.01	-0.05	0.03	0.782	1269	-0.03	-0.06	0.00	0.087
Triglycerides in medium LDL (mmol/l)	1269	0.00	-0.03	0.04	0.875	1269	0.00	-0.03	0.04	0.863	1269	0.00	-0.03	0.03	0.927
Concentration of small LDL particles (mol/l)	1269	-0.01	-0.05	0.03	0.555	1269	0.00	-0.04	0.03	0.816	1269	-0.03	-0.06	0.01	0.126
Total lipids in small LDL (mmol/l)	1269	-0.01	-0.05	0.03	0.606	1269	0.00	-0.04	0.04	0.882	1269	-0.03	-0.06	0.01	0.108
Phospholipids in small LDL (mmol/l)	1269	-0.01	-0.05	0.03	0.694	1269	0.00	-0.04	0.04	0.988	1269	-0.02	-0.05	0.01	0.167
Total cholesterol in small LDL (mmol/l)	1269	-0.01	-0.05	0.03	0.576	1269	0.00	-0.04	0.03	0.829	1269	-0.03	-0.06	0.00	0.085
Cholesterol esters in small LDL (mmol/l)	1269	-0.01	-0.05	0.03	0.595	1269	0.00	-0.04	0.03	0.854	1269	-0.03	-0.06	0.01	0.113
Free cholesterol in small LDL (mmol/l)	1269	-0.01	-0.05	0.03	0.519	1269	-0.01	-0.05	0.03	0.733	1269	-0.03	-0.06	0.00	0.077
Triglycerides in small LDL (mmol/l)	1269	0.00	-0.04	0.03	0.836	1269	0.00	-0.03	0.04	0.914	1269	-0.01	-0.04	0.03	0.685
Concentration of very large HDL particles (mol/l)	1269	0.00	-0.03	0.04	0.841	1269	0.00	-0.04	0.04	0.873	1269	0.00	-0.03	0.03	0.931
Total lipids in very large HDL (mmol/l)	1269	0.00	-0.03	0.04	0.854	1269	0.00	-0.04	0.04	0.888	1269	0.00	-0.03	0.03	0.972
Phospholipids in very large HDL (mmol/l)	1269	0.01	-0.03	0.04	0.745	1269	0.00	-0.04	0.04	0.906	1269	0.00	-0.03	0.03	0.825
Total cholesterol in very large HDL (mmol/l)	1269	0.00	-0.04	0.04	0.980	1269	0.00	-0.04	0.04	0.820	1269	0.00	-0.04	0.03	0.814
Cholesterol esters in very large HDL (mmol/l)	1269	0.00	-0.04	0.04	0.916	1269	-0.01	-0.05	0.03	0.790	1269	-0.01	-0.04	0.03	0.743
Free cholesterol in very large HDL (mmol/l)	1269	0.00	-0.03	0.04	0.832	1269	0.00	-0.04	0.04	0.928	1269	0.00	-0.03	0.03	0.921
Triglycerides in very large HDL (mmol/l)	1269	0.01	-0.02	0.05	0.452	1269	0.02	-0.02	0.06	0.302	1269	0.02	-0.02	0.06	0.283
Concentration of large HDL particles (mol/l)	1269	0.00	-0.03	0.04	0.804	1269	-0.01	-0.04	0.03	0.732	1269	0.00	-0.03	0.03	0.925
Total lipids in large HDL (mmol/l)	1269	0.01	-0.03	0.05	0.697	1269	0.00	-0.04	0.03	0.840	1269	0.00	-0.03	0.03	0.918
Phospholipids in large HDL (mmol/l)	1269	0.01	-0.03	0.04	0.765	1269	0.00	-0.04	0.03	0.798	1269	0.00	-0.03	0.03	0.898
Total cholesterol in large HDL (mmol/l)	1269	0.01	-0.03	0.05	0.658	1269	0.00	-0.04	0.04	0.854	1269	0.00	-0.03	0.03	0.786
Cholesterol esters in large HDL (mmol/l)	1269	0.01	-0.03	0.05	0.663	1269	0.00	-0.04	0.03	0.845	1269	0.00	-0.03	0.03	0.783
Free cholesterol in large HDL (mmol/l)	1269	0.01	-0.03	0.05	0.638	1269	0.00	-0.04	0.04	0.891	1269	0.00	-0.03	0.03	0.806
Triglycerides in large HDL (mmol/l)	1269	0.02	-0.02	0.05	0.357	1269	0.02	-0.02	0.05	0.343	1269	0.02	-0.02	0.05	0.319
Concentration of medium HDL particles (mol/l)	1269	0.00	-0.04	0.03	0.831	1269	-0.01	-0.04	0.03	0.630	1269	-0.01	-0.04	0.02	0.555
Total lipids in medium HDL (mmol/l)	1269	0.00	-0.04	0.03	0.943	1269	-0.01	-0.04	0.03	0.692	1269	-0.01	-0.04	0.02	0.620
Phospholipids in medium HDL (mmol/l)	1269	0.00	-0.04	0.03	0.886	1269	-0.01	-0.04	0.03	0.641	1269	-0.01	-0.04	0.02	0.585
Total cholesterol in medium HDL (mmol/l)	1269	0.00	-0.04	0.04	0.956	1269	-0.01	-0.04	0.03	0.729	1269	-0.01	-0.04	0.03	0.709
Cholesterol esters in medium HDL (mmol/l)	1269	0.00	-0.04	0.04	0.994	1269	-0.01	-0.05	0.03	0.679	1269	-0.01	-0.04	0.03	0.671
Free cholesterol in medium HDL (mmol/l)	1269	0.00	-0.03	0.04	0.824	1269	0.00	-0.04	0.04	0.958	1269	0.00	-0.04	0.03	0.853
Triglycerides in medium HDL (mmol/l)	1269	0.00	-0.04	0.03	0.845	1269	0.01	-0.03	0.04	0.741	1269	-0.01	-0.04	0.03	0.714
Concentration of small HDL particles (mol/l)	1269	-0.01	-0.04	0.03	0.616	1269	-0.01	-0.05	0.03	0.599	1269	-0.02	-0.05	0.02	0.368
Total lipids in small HDL (mmol/l)	1269	-0.01	-0.04	0.03	0.602	1269	-0.01	-0.05	0.02	0.487	1269	-0.02	-0.05	0.02	0.354
Phospholipids in small HDL (mmol/l)	1269	0.00	-0.04	0.03	0.794	1269	-0.01	-0.04	0.03	0.743	1269	-0.01	-0.05	0.02	0.536
Total cholesterol in small HDL (mmol/l)	1269	-0.01	-0.05	0.02	0.464	1269	-0.02	-0.05	0.02	0.288	1269	-0.02	-0.05	0.01	0.211
Cholesterol esters in small HDL (mmol/l)	1269	-0.01	-0.05	0.02	0.440	1269	-0.02	-0.05	0.02	0.289	1269	-0.02	-0.05	0.01	0.195
Free cholesterol in small HDL (mmol/l)	1269	-0.01	-0.04	0.03	0.723	1269	-0.01	-0.05	0.02	0.489	1269	-0.02	-0.05	0.02	0.399
Triglycerides in small HDL (mmol/l)	1269	0.00	-0.04	0.04	0.982	1269	0.01	-0.03	0.05	0.648	1269	0.00	-0.04	0.04	0.944
Phospholipids to total lipds ratio in chylomicrons and extremely large VLDL (%)	1269	0.03	-0.04	0.10	0.385	1269	0.04	-0.04	0.11	0.317	1269	0.03	-0.04	0.10	0.423
Total cholesterol to total lipids ratio in chylomicrons and extremely large VLDL (%)	1269	0.00	-0.04	0.04	0.839	1269	0.00	-0.04	0.04	0.912	1269	0.00	-0.04	0.04	0.975
Cholesterol esters to total lipids ratio in chylomicrons and extremely large VLDL (%)	1269	0.00	-0.04	0.04	0.842	1269	-0.01	-0.05	0.04	0.806	1269	0.00	-0.04	0.04	0.947
Free cholesterol to total lipids ratio in chylomicrons and extremely large VLDL (%)	1269	0.01	-0.03	0.05	0.797	1269	0.02	-0.03	0.06	0.450	1269	0.01	-0.03	0.05	0.646
Triglycerides to total lipids ratio in chylomicrons and extremely large VLDL (%)	1269	0.01	-0.04	0.05	0.782	1269	0.00	-0.04	0.05	0.977	1269	0.00	-0.04	0.04	0.933

S5 Table Observational associations of age at puberty onset (per year later) with adiposity and cardiometabolic traits at age 18y among females and males in ALSPAC

	Adj. for age, sex, education					*Adj. for age, sex, education, BMI at age 8y*					*Adj. for age, sex, education, outcome value at age 8y*				
**Standardised outcome at age 18y**	**N**	**Beta**	**LCL**	**UCL**	**P-value**	**N**	**Beta**	**LCL**	**UCL**	**P-value**	**N**	**Beta**	**LCL**	**UCL**	**P-value**
Phospholipids to total lipds ratio in very large VLDL (%)	1269	0.02	-0.03	0.07	0.368	1269	0.04	-0.01	0.08	0.163	1269	0.03	-0.02	0.07	0.278
Total cholesterol to total lipids ratio in very large VLDL (%)	1269	0.02	-0.02	0.06	0.246	1269	0.02	-0.02	0.06	0.273	1269	0.02	-0.02	0.06	0.275
Cholesterol esters to total lipids ratio in very large VLDL (%)	1269	0.01	-0.02	0.03	0.618	1269	0.00	-0.02	0.03	0.745	1269	0.01	-0.02	0.03	0.683
Free cholesterol to total lipids ratio in very large VLDL (%)	1269	0.03	-0.01	0.07	0.183	1269	0.03	-0.01	0.07	0.162	1269	0.03	-0.01	0.07	0.193
Triglycerides to total lipids ratio in very large VLDL (%)	1269	-0.04	-0.08	0.01	0.115	1269	-0.04	-0.08	0.01	0.095	1269	-0.03	-0.08	0.01	0.118
Phospholipids to total lipds ratio in large VLDL (%)	1269	0.02	-0.04	0.09	0.457	1269	0.03	-0.03	0.09	0.326	1269	0.03	-0.03	0.09	0.378
Total cholesterol to total lipids ratio in large VLDL (%)	1269	0.01	-0.03	0.05	0.471	1269	0.02	-0.02	0.07	0.229	1269	0.02	-0.02	0.06	0.267
Cholesterol esters to total lipids ratio in large VLDL (%)	1269	0.02	-0.02	0.06	0.249	1269	0.03	-0.01	0.07	0.157	1269	0.03	-0.01	0.07	0.195
Free cholesterol to total lipids ratio in large VLDL (%)	1269	0.00	-0.02	0.01	0.830	1269	0.00	-0.01	0.02	0.749	1269	0.00	-0.02	0.02	0.937
Triglycerides to total lipids ratio in large VLDL (%)	1269	-0.02	-0.06	0.02	0.324	1269	-0.03	-0.07	0.01	0.148	1269	-0.03	-0.07	0.01	0.162
Phospholipids to total lipds ratio in medium VLDL (%)	1269	0.02	-0.01	0.06	0.241	1269	0.02	-0.02	0.05	0.438	1269	0.01	-0.02	0.05	0.464
Total cholesterol to total lipids ratio in medium VLDL (%)	1269	0.00	-0.04	0.04	0.877	1269	0.01	-0.03	0.05	0.694	1269	0.01	-0.03	0.04	0.664
Cholesterol esters to total lipids ratio in medium VLDL (%)	1269	0.01	-0.03	0.05	0.701	1269	0.01	-0.03	0.05	0.573	1269	0.01	-0.02	0.05	0.510
Free cholesterol to total lipids ratio in medium VLDL (%)	1269	-0.01	-0.06	0.03	0.553	1269	-0.01	-0.05	0.04	0.750	1269	-0.01	-0.05	0.03	0.504
Triglycerides to total lipids ratio in medium VLDL (%)	1269	-0.01	-0.05	0.03	0.699	1269	-0.01	-0.05	0.03	0.595	1269	-0.01	-0.04	0.02	0.598
Phospholipids to total lipds ratio in small VLDL (%)	1269	0.00	-0.04	0.04	0.976	1269	-0.01	-0.05	0.02	0.456	1269	-0.01	-0.05	0.03	0.561
Total cholesterol to total lipids ratio in small VLDL (%)	1269	0.00	-0.05	0.04	0.857	1269	0.00	-0.05	0.04	0.896	1269	-0.01	-0.05	0.03	0.771
Cholesterol esters to total lipids ratio in small VLDL (%)	1269	0.00	-0.05	0.04	0.843	1269	0.00	-0.04	0.04	0.941	1269	0.00	-0.04	0.04	0.826
Free cholesterol to total lipids ratio in small VLDL (%)	1269	0.00	-0.03	0.04	0.896	1269	-0.01	-0.05	0.03	0.654	1269	-0.01	-0.04	0.02	0.553
Triglycerides to total lipids ratio in small VLDL (%)	1269	0.00	-0.04	0.05	0.854	1269	0.01	-0.03	0.05	0.710	1269	0.01	-0.03	0.05	0.701
Phospholipids to total lipds ratio in very small VLDL (%)	1269	0.00	-0.04	0.04	0.961	1269	0.00	-0.04	0.04	0.973	1269	-0.01	-0.04	0.02	0.552
Total cholesterol to total lipids ratio in very small VLDL (%)	1269	-0.01	-0.05	0.03	0.641	1269	-0.01	-0.05	0.03	0.667	1269	-0.01	-0.05	0.03	0.668
Cholesterol esters to total lipids ratio in very small VLDL (%)	1269	-0.01	-0.05	0.03	0.606	1269	-0.01	-0.05	0.03	0.747	1269	-0.01	-0.04	0.03	0.703
Free cholesterol to total lipids ratio in very small VLDL (%)	1269	0.00	-0.03	0.03	0.978	1269	-0.01	-0.04	0.02	0.577	1269	0.00	-0.03	0.03	0.986
Triglycerides to total lipids ratio in very small VLDL (%)	1269	0.01	-0.03	0.05	0.624	1269	0.01	-0.03	0.05	0.646	1269	0.01	-0.03	0.05	0.587
Phospholipids to total lipds ratio in IDL (%)	1269	0.00	-0.04	0.04	0.910	1269	-0.02	-0.06	0.03	0.465	1269	-0.01	-0.05	0.03	0.757
Total cholesterol to total lipids ratio in IDL (%)	1269	-0.01	-0.06	0.03	0.477	1269	-0.01	-0.05	0.04	0.796	1269	-0.01	-0.05	0.03	0.504
Cholesterol esters to total lipids ratio in IDL (%)	1269	-0.01	-0.05	0.03	0.657	1269	0.00	-0.04	0.04	0.842	1269	0.00	-0.04	0.03	0.847
Free cholesterol to total lipids ratio in IDL (%)	1269	-0.01	-0.06	0.03	0.490	1269	-0.02	-0.07	0.02	0.309	1269	-0.03	-0.06	0.01	0.206
Triglycerides to total lipids ratio in IDL (%)	1269	0.02	-0.02	0.06	0.383	1269	0.01	-0.03	0.05	0.555	1269	0.02	-0.02	0.06	0.328
Phospholipids to total lipds ratio in large LDL (%)	1269	0.02	-0.03	0.06	0.449	1269	0.01	-0.03	0.06	0.609	1269	0.02	-0.01	0.06	0.136
Total cholesterol to total lipids ratio in large LDL (%)	1269	-0.03	-0.07	0.02	0.284	1269	-0.02	-0.07	0.03	0.469	1269	-0.03	-0.07	0.00	0.062
Cholesterol esters to total lipids ratio in large LDL (%)	1269	-0.02	-0.07	0.02	0.317	1269	-0.01	-0.06	0.03	0.584	1269	-0.03	-0.06	0.00	0.063
Free cholesterol to total lipids ratio in large LDL (%)	1269	0.01	-0.03	0.05	0.749	1269	0.00	-0.05	0.04	0.830	1269	0.00	-0.03	0.04	0.852
Triglycerides to total lipids ratio in large LDL (%)	1269	0.02	-0.02	0.07	0.297	1269	0.02	-0.03	0.06	0.485	1269	0.03	-0.01	0.06	0.208
Phospholipids to total lipds ratio in medium LDL (%)	1269	0.01	-0.03	0.05	0.553	1269	0.01	-0.03	0.05	0.717	1269	0.03	-0.01	0.06	0.149
Total cholesterol to total lipids ratio in medium LDL (%)	1269	-0.03	-0.08	0.02	0.275	1269	-0.02	-0.07	0.03	0.423	1269	-0.04	-0.08	0.00	0.049
Cholesterol esters to total lipids ratio in medium LDL (%)	1269	-0.02	-0.06	0.03	0.453	1269	-0.01	-0.05	0.04	0.710	1269	-0.03	-0.06	0.00	0.073
Free cholesterol to total lipids ratio in medium LDL (%)	1269	0.00	-0.05	0.04	0.825	1269	-0.01	-0.06	0.03	0.587	1269	0.00	-0.04	0.05	0.914
Triglycerides to total lipids ratio in medium LDL (%)	1269	0.04	-0.03	0.11	0.282	1269	0.03	-0.04	0.10	0.379	1269	0.04	-0.03	0.11	0.292
Phospholipids to total lipds ratio in small LDL (%)	1269	0.03	-0.02	0.07	0.295	1269	0.02	-0.03	0.07	0.431	1269	0.04	0.00	0.08	0.042
Total cholesterol to total lipids ratio in small LDL (%)	1269	-0.03	-0.07	0.02	0.297	1269	-0.02	-0.07	0.03	0.424	1269	-0.04	-0.08	0.00	0.050
Cholesterol esters to total lipids ratio in small LDL (%)	1269	-0.02	-0.06	0.02	0.429	1269	-0.01	-0.05	0.03	0.641	1269	-0.03	-0.06	0.00	0.072
Free cholesterol to total lipids ratio in small LDL (%)	1269	0.00	-0.04	0.04	0.909	1269	-0.01	-0.05	0.03	0.652	1269	0.00	-0.04	0.04	0.876
Triglycerides to total lipids ratio in small LDL (%)	1269	0.00	-0.04	0.05	0.821	1269	0.01	-0.04	0.05	0.815	1269	0.01	-0.03	0.05	0.766
Phospholipids to total lipds ratio in very large HDL (%)	1269	0.01	-0.03	0.05	0.528	1269	0.00	-0.04	0.04	0.983	1269	0.01	-0.02	0.05	0.413
Total cholesterol to total lipids ratio in very large HDL (%)	1269	-0.01	-0.05	0.02	0.452	1269	0.00	-0.04	0.03	0.840	1269	-0.02	-0.05	0.01	0.273
Cholesterol esters to total lipids ratio in very large HDL (%)	1269	-0.01	-0.05	0.02	0.475	1269	0.00	-0.04	0.04	0.858	1269	-0.02	-0.05	0.02	0.284
Free cholesterol to total lipids ratio in very large HDL (%)	1269	0.00	-0.04	0.04	0.920	1269	0.00	-0.04	0.04	0.866	1269	0.00	-0.04	0.04	0.969
Triglycerides to total lipids ratio in very large HDL (%)	1269	0.01	-0.03	0.05	0.671	1269	0.02	-0.02	0.06	0.278	1269	0.02	-0.02	0.05	0.432

S5 Table Observational associations of age at puberty onset (per year later) with adiposity and cardiometabolic traits at age 18y among females and males in ALSPAC

	Adj. for age, sex, education					*Adj. for age, sex, education, BMI at age 8y*					*Adj. for age, sex, education, outcome value at age 8y*				
**Standardised outcome at age 18y**	**N**	**Beta**	**LCL**	**UCL**	**P-value**	**N**	**Beta**	**LCL**	**UCL**	**P-value**	**N**	**Beta**	**LCL**	**UCL**	**P-value**
Phospholipids to total lipds ratio in large HDL (%)	1269	-0.02	-0.06	0.02	0.260	1269	-0.01	-0.04	0.03	0.748	1269	-0.03	-0.06	0.01	0.099
Total cholesterol to total lipids ratio in large HDL (%)	1269	0.01	-0.02	0.05	0.458	1269	0.00	-0.04	0.04	0.891	1269	0.02	-0.01	0.06	0.231
Cholesterol esters to total lipids ratio in large HDL (%)	1269	0.01	-0.03	0.05	0.481	1269	0.00	-0.04	0.04	0.886	1269	0.02	-0.01	0.06	0.231
Free cholesterol to total lipids ratio in large HDL (%)	1269	0.01	-0.03	0.05	0.522	1269	0.00	-0.04	0.04	0.957	1269	0.01	-0.02	0.05	0.415
Triglycerides to total lipids ratio in large HDL (%)	1269	0.01	-0.03	0.04	0.785	1269	0.02	-0.02	0.06	0.284	1269	0.01	-0.03	0.04	0.654
Phospholipids to total lipds ratio in medium HDL (%)	1269	-0.01	-0.04	0.03	0.789	1269	-0.01	-0.04	0.03	0.770	1269	-0.01	-0.04	0.03	0.743
Total cholesterol to total lipids ratio in medium HDL (%)	1269	0.01	-0.03	0.04	0.744	1269	0.00	-0.04	0.04	0.983	1269	0.01	-0.03	0.04	0.688
Cholesterol esters to total lipids ratio in medium HDL (%)	1269	0.00	-0.03	0.04	0.872	1269	0.00	-0.04	0.03	0.821	1269	0.00	-0.03	0.04	0.803
Free cholesterol to total lipids ratio in medium HDL (%)	1269	0.02	-0.03	0.06	0.470	1269	0.02	-0.03	0.06	0.444	1269	0.02	-0.03	0.06	0.489
Triglycerides to total lipids ratio in medium HDL (%)	1269	0.00	-0.04	0.04	0.901	1269	0.01	-0.03	0.05	0.551	1269	0.00	-0.04	0.03	0.901
Phospholipids to total lipds ratio in small HDL (%)	1269	0.01	-0.03	0.04	0.593	1269	0.01	-0.02	0.05	0.470	1269	0.02	-0.02	0.05	0.347
Total cholesterol to total lipids ratio in small HDL (%)	1269	-0.01	-0.05	0.02	0.524	1269	-0.02	-0.06	0.02	0.320	1269	-0.02	-0.05	0.02	0.304
Cholesterol esters to total lipids ratio in small HDL (%)	1269	-0.01	-0.05	0.02	0.533	1269	-0.02	-0.05	0.02	0.381	1269	-0.02	-0.05	0.02	0.313
Free cholesterol to total lipids ratio in small HDL (%)	1269	0.00	-0.04	0.04	0.904	1269	-0.01	-0.05	0.03	0.702	1269	0.01	-0.03	0.04	0.754
Triglycerides to total lipids ratio in small HDL (%)	1269	0.01	-0.03	0.05	0.713	1269	0.02	-0.02	0.06	0.339	1269	0.01	-0.03	0.05	0.607
Mean diameter for VLDL particles (nm)	1269	0.00	-0.04	0.04	0.922	1269	0.01	-0.03	0.05	0.556	1269	0.00	-0.04	0.04	0.837
Mean diameter for LDL particles (nm)	1269	0.04	-0.01	0.08	0.129	1269	0.03	-0.01	0.08	0.175	1269	0.04	0.00	0.09	0.045
Mean diameter for HDL particles (nm)	1269	0.01	-0.03	0.05	0.705	1269	0.00	-0.04	0.04	0.909	1269	0.01	-0.02	0.04	0.568
Serum total cholesterol (mmol/l)	1269	-0.01	-0.04	0.03	0.734	1269	0.00	-0.04	0.04	0.972	1269	-0.02	-0.05	0.01	0.172
Total cholesterol in VLDL (mmol/l)	1269	0.00	-0.04	0.04	0.907	1269	0.01	-0.03	0.06	0.501	1269	0.00	-0.04	0.04	0.942
Remnant cholesterol (non-HDL, non-LDL -cholesterol) (mmol/l)	1269	0.00	-0.04	0.04	0.825	1269	0.01	-0.03	0.05	0.656	1269	-0.01	-0.04	0.02	0.586
Total cholesterol in LDL (mmol/l)	1269	-0.01	-0.05	0.03	0.636	1269	0.00	-0.04	0.04	0.900	1269	-0.02	-0.06	0.01	0.120
Total cholesterol in HDL (mmol/l)	1269	0.00	-0.04	0.04	0.963	1269	-0.01	-0.05	0.03	0.634	1269	-0.01	-0.04	0.02	0.548
Total cholesterol in HDL2 (mmol/l)	1269	0.00	-0.04	0.04	0.994	1269	-0.01	-0.05	0.03	0.551	1269	-0.01	-0.04	0.02	0.520
Total cholesterol in HDL3 (mmol/l)	1269	0.00	-0.03	0.04	0.905	1269	0.00	-0.04	0.03	0.828	1269	-0.01	-0.04	0.02	0.670
Esterified cholesterol (mmol/l)	1269	0.00	-0.04	0.03	0.810	1269	0.00	-0.04	0.04	0.960	1269	-0.02	-0.05	0.01	0.208
Free cholesterol (mmol/l)	1269	-0.01	-0.05	0.03	0.587	1269	0.00	-0.04	0.03	0.815	1269	-0.02	-0.05	0.01	0.188
Serum total triglycerides (mmol/l)	1269	0.00	-0.04	0.04	0.897	1269	0.02	-0.02	0.06	0.388	1269	0.00	-0.04	0.04	0.866
Triglycerides in VLDL (mmol/l)	1269	0.00	-0.04	0.04	0.920	1269	0.02	-0.02	0.06	0.370	1269	0.00	-0.04	0.04	0.828
Triglycerides in LDL (mmol/l)	1269	0.00	-0.03	0.03	0.934	1269	0.00	-0.03	0.04	0.850	1269	0.00	-0.04	0.03	0.849
Triglycerides in HDL (mmol/l)	1269	0.00	-0.03	0.04	0.808	1269	0.01	-0.02	0.05	0.470	1269	0.00	-0.03	0.04	0.800
Diacylglycerol (mmol/l)	1269	-0.01	-0.05	0.03	0.551	1269	0.00	-0.04	0.04	0.892	1269	-0.01	-0.05	0.03	0.733
Ratio of diacylglycerol to triglycerides	1269	-0.01	-0.04	0.03	0.731	1269	0.00	-0.04	0.03	0.912	1269	0.00	-0.04	0.03	0.854
Total phosphoglycerides (mmol/l)	1269	-0.01	-0.04	0.03	0.655	1269	-0.01	-0.04	0.03	0.785	1269	-0.01	-0.05	0.02	0.389
Ratio of triglycerides to phosphoglycerides	1269	0.01	-0.03	0.05	0.650	1269	0.02	-0.02	0.07	0.268	1269	0.01	-0.03	0.05	0.504
Phosphatidylcholine and other cholines (mmol/l)	1269	0.00	-0.04	0.03	0.813	1269	-0.01	-0.04	0.03	0.740	1269	-0.01	-0.05	0.02	0.368
Total cholines (mmol/l)	1269	-0.01	-0.04	0.02	0.566	1269	-0.01	-0.04	0.03	0.616	1269	-0.02	-0.05	0.01	0.182
Apolipoprotein A-I (g/l)	1269	0.00	-0.04	0.04	0.993	1269	0.00	-0.04	0.03	0.797	1269	-0.01	-0.04	0.02	0.428
Apolipoprotein B (g/l)	1269	0.00	-0.04	0.04	0.864	1269	0.01	-0.03	0.05	0.598	1269	-0.01	-0.04	0.03	0.637
Ratio of apolipoprotein B to apolipoprotein A-I	1269	0.00	-0.05	0.04	0.848	1269	0.01	-0.03	0.05	0.545	1269	0.00	-0.04	0.03	0.838
Total fatty acids (mmol/l)	1269	0.00	-0.04	0.04	0.907	1269	0.01	-0.03	0.05	0.685	1269	-0.01	-0.04	0.03	0.698
Estimated description of fatty acid chain length, not actual carbon number	1269	0.03	-0.01	0.07	0.159	1269	0.03	-0.01	0.07	0.155	1269	0.03	-0.01	0.07	0.147
Estimated degree of unsaturation	1269	0.02	-0.02	0.06	0.310	1269	0.01	-0.03	0.06	0.472	1269	0.01	-0.02	0.05	0.477
22:6, docosahexaenoic acid (mmol/l)	1269	0.01	-0.02	0.05	0.430	1269	0.02	-0.02	0.06	0.293	1269	0.00	-0.04	0.03	0.896
18:2, linoleic acid (mmol/l)	1269	0.00	-0.04	0.04	0.931	1269	0.00	-0.04	0.04	0.990	1269	-0.01	-0.04	0.02	0.532
Conjugated linoleic acid (mmol/l)	1269	-0.01	-0.05	0.03	0.577	1269	0.00	-0.04	0.04	0.957	1269	-0.01	-0.05	0.03	0.598
Omega-3 fatty acids (mmol/l)	1269	0.01	-0.03	0.05	0.483	1269	0.02	-0.02	0.06	0.249	1269	0.00	-0.04	0.03	0.857
Omega-6 fatty acids (mmol/l)	1269	0.00	-0.04	0.03	0.891	1269	0.00	-0.04	0.04	0.926	1269	-0.01	-0.05	0.02	0.407
Polyunsaturated fatty acids (mmol/l)	1269	0.00	-0.04	0.04	0.985	1269	0.00	-0.03	0.04	0.800	1269	-0.01	-0.05	0.02	0.419
Monounsaturated fatty acids; 16:1, 18:1 (mmol/l)	1269	0.00	-0.04	0.04	0.983	1269	0.01	-0.03	0.05	0.575	1269	0.00	-0.04	0.04	0.926

S5 Table Observational associations of age at puberty onset (per year later) with adiposity and cardiometabolic traits at age 18y among females and males in ALSPAC

	Adj. for age, sex, education					*Adj. for age, sex, education, BMI at age 8y*					*Adj. for age, sex, education, outcome value at age 8y*				
**Standardised outcome at age 18y**	**N**	**Beta**	**LCL**	**UCL**	**P-value**	**N**	**Beta**	**LCL**	**UCL**	**P-value**	**N**	**Beta**	**LCL**	**UCL**	**P-value**
Saturated fatty acids (mmol/l)	1269	0.00	-0.04	0.03	0.793	1269	0.01	-0.03	0.04	0.760	1269	-0.01	-0.04	0.03	0.711
Ratio of 22:6 docosahexaenoic acid to total fatty acids (%)	1269	0.02	-0.02	0.06	0.257	1269	0.02	-0.02	0.06	0.237	1269	0.01	-0.03	0.04	0.698
Ratio of 18:2 linoleic acid to total fatty acids (%)	1269	0.00	-0.04	0.04	0.902	1269	-0.02	-0.06	0.02	0.402	1269	0.00	-0.04	0.04	0.847
Ratio of conjugated linoleic acid to total fatty acids (%)	1269	-0.01	-0.04	0.03	0.709	1269	0.00	-0.04	0.04	0.995	1269	-0.01	-0.04	0.03	0.729
Ratio of omega-3 fatty acids to total fatty acids (%)	1269	0.02	-0.02	0.06	0.271	1269	0.03	-0.02	0.07	0.219	1269	0.01	-0.03	0.05	0.678
Ratio of omega-6 fatty acids to total fatty acids (%)	1269	0.00	-0.04	0.04	0.975	1269	-0.01	-0.06	0.03	0.514	1269	0.00	-0.04	0.04	0.885
Ratio of polyunsaturated fatty acids to total fatty acids (%)	1269	0.00	-0.04	0.05	0.819	1269	-0.01	-0.05	0.03	0.742	1269	0.00	-0.04	0.04	0.984
Ratio of monounsaturated fatty acids to total fatty acids (%)	1269	0.00	-0.04	0.04	0.875	1269	0.01	-0.03	0.05	0.591	1269	0.00	-0.03	0.04	0.810
Ratio of saturated fatty acids to total fatty acids (%)	1269	-0.01	-0.05	0.03	0.581	1269	-0.01	-0.04	0.03	0.724	1269	-0.01	-0.04	0.03	0.696
Glucose (mmol/l)	1269	-0.01	-0.04	0.02	0.487	1269	-0.01	-0.04	0.03	0.705	1269	-0.01	-0.04	0.03	0.678
Lactate (mmol/l)	1269	-0.01	-0.05	0.03	0.764	1269	-0.01	-0.05	0.04	0.804	1269	-0.01	-0.05	0.03	0.720
Pyruvate (mmol/l)	1269	-0.03	-0.07	0.01	0.205	1269	-0.02	-0.06	0.02	0.397	1269	-0.03	-0.07	0.01	0.189
Citrate (mmol/l)	1269	0.03	-0.01	0.08	0.113	1269	0.02	-0.02	0.06	0.306	1269	0.03	-0.01	0.07	0.129
Alanine (mmol/l)	1269	0.01	-0.03	0.05	0.663	1269	0.02	-0.03	0.06	0.492	1269	0.01	-0.03	0.05	0.617
Glutamine (mmol/l)	1269	0.03	-0.01	0.07	0.128	1269	0.03	-0.01	0.06	0.174	1269	0.03	-0.01	0.07	0.106
Histidine (mmol/l)	1269	0.00	-0.04	0.04	0.884	1269	0.00	-0.04	0.04	0.897	1269	0.00	-0.04	0.04	0.822
Isoleucine (mmol/l)	1269	0.03	-0.01	0.07	0.103	1269	0.05	0.01	0.09	0.027	1269	0.03	-0.01	0.07	0.101
Leucine (mmol/l)	1269	0.03	-0.01	0.06	0.122	1269	0.04	0.00	0.08	0.039	1269	0.03	-0.01	0.07	0.112
Valine (mmol/l)	1269	0.02	-0.02	0.06	0.251	1269	0.04	0.00	0.07	0.063	1269	0.02	-0.02	0.06	0.276
Phenylalanine (mmol/l)	1269	-0.02	-0.06	0.02	0.286	1269	-0.01	-0.05	0.03	0.561	1269	-0.02	-0.06	0.02	0.270
Tyrosine (mmol/l)	1269	-0.01	-0.05	0.03	0.584	1269	0.00	-0.03	0.04	0.800	1269	-0.01	-0.05	0.03	0.585
Acetate (mmol/l)	1269	0.00	-0.04	0.03	0.869	1269	0.00	-0.04	0.03	0.782	1269	0.00	-0.04	0.03	0.832
Acetoacetate (mmol/l)	1269	0.00	-0.04	0.04	0.993	1269	0.00	-0.04	0.05	0.913	1269	0.00	-0.04	0.04	0.997
3-hydroxybutyrate (mmol/l)	1269	0.01	-0.03	0.05	0.664	1269	0.01	-0.04	0.05	0.733	1269	0.01	-0.03	0.05	0.685
Creatinine (mmol/l)	1269	-0.05	-0.08	-0.01	0.005	1269	-0.04	-0.08	-0.01	0.021	1269	-0.03	-0.06	0.00	0.047
Albumin (signal area)	1269	0.02	-0.02	0.06	0.232	1269	0.02	-0.02	0.06	0.241	1269	0.02	-0.02	0.06	0.239
Glycoprotein acetyls, mainly a1-acid glycoprotein (mmol/l)	1269	0.00	-0.04	0.04	0.956	1269	0.01	-0.03	0.05	0.492	1269	0.01	-0.03	0.05	0.692
