# Supplementary material for: Influence of puberty timing on adiposity and cardiometabolic traits: A Mendelian randomisation study
Source: PLoS Med. 2018 Aug 28;15(8):e1002641. doi: 10.1371/journal.pmed.1002641 (PMC6112630; doi:10.1371/journal.pmed.1002641)
Supplement: S6 Table — (PDF) [file pmed.1002641.s025.pdf]

S6 Table One-sample MR estimates of associations of age at menarche (per year later) with adiposity and cardiometabolic traits at age 18y among females in ALSPAC, using a full GRS of 351 SNPs for age at menarche

	Unadj.					*Adj. for measured BMI at age 8y*					*Adj. for measured outcome value at age 8y*				
**Standardised outcome at age 18y**	**N**	**Beta (2SLS)**	**LCL**	**UCL**	**P-value**	**N**	**Beta (2SLS)**	**LCL**	**UCL**	**P-value**	**N**	**Beta (2SLS)**	**LCL**	**UCL**	**P-value**
Body mass index (kg/m^2)	2053	-0.34	-0.46	-0.23	9.77E-09	1838	-0.03	-0.13	0.07	0.554	1838	-0.03	-0.13	0.07	0.554
Fat mass index (kg/m^2)	1976	-0.30	-0.41	-0.19	2.76E-08	1779	-0.04	-0.14	0.05	0.361	1730	-0.06	-0.15	0.04	0.251
Lean mass index (kg/m^2)	1976	-0.13	-0.19	-0.06	2.60E-04	1779	-0.01	-0.08	0.06	0.817	1730	0.02	-0.04	0.07	0.547
Systolic blood pressure (mmHg)	1933	-0.10	-0.19	0.00	0.049	1739	-0.04	-0.15	0.06	0.448	1721	-0.05	-0.14	0.04	0.294
Diastolic blood pressure (mmHg)	1933	-0.09	-0.20	0.02	0.109	1739	-0.02	-0.14	0.10	0.709	1721	-0.04	-0.15	0.06	0.420
Concentration of chylomicrons and extremely large VLDL particles (mol/l)	1268	0.01	-0.11	0.13	0.870	1126	0.06	-0.08	0.21	0.380	873	0.04	-0.09	0.17	0.554
Total lipids in chylomicrons and extremely large VLDL (mmol/l)	1268	0.01	-0.11	0.13	0.887	1126	0.06	-0.08	0.21	0.395	873	0.04	-0.09	0.17	0.532
Phospholipids in chylomicrons and extremely large VLDL (mmol/l)	1268	0.01	-0.12	0.13	0.931	1126	0.06	-0.09	0.20	0.434	873	0.04	-0.10	0.17	0.586
Total cholesterol in chylomicrons and extremely large VLDL (mmol/l)	1268	0.02	-0.11	0.15	0.772	1126	0.08	-0.08	0.23	0.320	873	0.06	-0.08	0.19	0.409
Cholesterol esters in chylomicrons and extremely large VLDL (mmol/l)	1268	0.03	-0.11	0.16	0.698	1126	0.09	-0.07	0.25	0.285	873	0.07	-0.07	0.21	0.318
Free cholesterol in chylomicrons and extremely large VLDL (mmol/l)	1268	0.01	-0.11	0.13	0.885	1126	0.06	-0.08	0.21	0.392	873	0.04	-0.09	0.17	0.560
Triglycerides in chylomicrons and extremely large VLDL (mmol/l)	1268	0.01	-0.11	0.13	0.911	1126	0.06	-0.08	0.20	0.412	873	0.04	-0.09	0.17	0.559
Concentration of very large VLDL particles (mol/l)	1268	0.01	-0.11	0.13	0.866	1126	0.07	-0.08	0.21	0.362	873	0.04	-0.09	0.17	0.554
Total lipids in very large VLDL (mmol/l)	1268	0.01	-0.11	0.13	0.859	1126	0.07	-0.08	0.21	0.363	873	0.04	-0.09	0.17	0.527
Phospholipids in very large VLDL (mmol/l)	1268	0.01	-0.11	0.13	0.901	1126	0.06	-0.08	0.21	0.395	873	0.04	-0.10	0.17	0.583
Total cholesterol in very large VLDL (mmol/l)	1268	0.01	-0.11	0.14	0.846	1126	0.07	-0.08	0.22	0.355	873	0.05	-0.09	0.18	0.500
Cholesterol esters in very large VLDL (mmol/l)	1268	0.01	-0.11	0.14	0.822	1126	0.07	-0.08	0.23	0.335	873	0.05	-0.08	0.18	0.465
Free cholesterol in very large VLDL (mmol/l)	1268	0.01	-0.11	0.13	0.874	1126	0.07	-0.08	0.21	0.383	873	0.04	-0.09	0.18	0.543
Triglycerides in very large VLDL (mmol/l)	1268	0.01	-0.11	0.13	0.853	1126	0.07	-0.08	0.21	0.362	873	0.04	-0.09	0.17	0.526
Concentration of large VLDL particles (mol/l)	1268	0.02	-0.11	0.15	0.796	1126	0.08	-0.08	0.23	0.336	873	0.06	-0.08	0.19	0.413
Total lipids in large VLDL (mmol/l)	1268	0.02	-0.11	0.14	0.811	1126	0.07	-0.08	0.23	0.341	873	0.06	-0.08	0.19	0.415
Phospholipids in large VLDL (mmol/l)	1268	0.02	-0.11	0.14	0.814	1126	0.07	-0.08	0.23	0.345	873	0.06	-0.08	0.19	0.410
Total cholesterol in large VLDL (mmol/l)	1268	0.02	-0.12	0.15	0.809	1126	0.08	-0.08	0.23	0.332	873	0.06	-0.08	0.20	0.388
Cholesterol esters in large VLDL (mmol/l)	1268	0.02	-0.12	0.15	0.792	1126	0.08	-0.08	0.24	0.313	873	0.07	-0.07	0.21	0.336
Free cholesterol in large VLDL (mmol/l)	1268	0.01	-0.11	0.14	0.829	1126	0.07	-0.08	0.23	0.358	873	0.05	-0.08	0.19	0.448
Triglycerides in large VLDL (mmol/l)	1268	0.02	-0.11	0.14	0.812	1126	0.07	-0.08	0.23	0.347	873	0.05	-0.08	0.19	0.432
Concentration of medium VLDL particles (mol/l)	1268	0.02	-0.12	0.15	0.819	1126	0.08	-0.09	0.24	0.356	873	0.07	-0.07	0.20	0.350
Total lipids in medium VLDL (mmol/l)	1268	0.01	-0.12	0.15	0.833	1126	0.08	-0.09	0.24	0.362	873	0.07	-0.07	0.21	0.343
Phospholipids in medium VLDL (mmol/l)	1268	0.02	-0.12	0.15	0.821	1126	0.08	-0.09	0.24	0.364	873	0.07	-0.07	0.21	0.321
Total cholesterol in medium VLDL (mmol/l)	1268	0.01	-0.13	0.15	0.861	1126	0.07	-0.09	0.24	0.391	873	0.07	-0.07	0.22	0.306
Cholesterol esters in medium VLDL (mmol/l)	1268	0.01	-0.14	0.15	0.917	1126	0.07	-0.10	0.24	0.446	873	0.07	-0.07	0.22	0.327
Free cholesterol in medium VLDL (mmol/l)	1268	0.02	-0.12	0.16	0.797	1126	0.08	-0.09	0.24	0.347	873	0.07	-0.07	0.21	0.313
Triglycerides in medium VLDL (mmol/l)	1268	0.01	-0.12	0.15	0.828	1126	0.07	-0.08	0.23	0.360	873	0.06	-0.08	0.20	0.387
Concentration of small VLDL particles (mol/l)	1268	0.04	-0.11	0.19	0.603	1126	0.10	-0.08	0.27	0.274	873	0.11	-0.04	0.26	0.158
Total lipids in small VLDL (mmol/l)	1268	0.04	-0.11	0.19	0.625	1126	0.09	-0.09	0.27	0.307	873	0.12	-0.04	0.27	0.133
Phospholipids in small VLDL (mmol/l)	1268	0.05	-0.11	0.20	0.543	1126	0.10	-0.08	0.29	0.274	873	0.12	-0.04	0.28	0.140
Total cholesterol in small VLDL (mmol/l)	1268	0.02	-0.13	0.18	0.773	1126	0.07	-0.12	0.25	0.486	873	0.12	-0.03	0.27	0.129
Cholesterol esters in small VLDL (mmol/l)	1268	0.01	-0.14	0.17	0.881	1126	0.05	-0.14	0.23	0.622	873	0.11	-0.04	0.27	0.158
Free cholesterol in small VLDL (mmol/l)	1268	0.04	-0.11	0.20	0.601	1126	0.09	-0.09	0.28	0.311	873	0.12	-0.04	0.27	0.139
Triglycerides in small VLDL (mmol/l)	1268	0.04	-0.11	0.19	0.585	1126	0.10	-0.07	0.27	0.252	873	0.10	-0.05	0.25	0.191
Concentration of very small VLDL particles (mol/l)	1268	0.02	-0.14	0.17	0.816	1126	0.05	-0.13	0.23	0.578	873	0.11	-0.04	0.27	0.144
Total lipids in very small VLDL (mmol/l)	1268	0.01	-0.15	0.17	0.900	1126	0.04	-0.15	0.22	0.711	873	0.11	-0.05	0.26	0.168
Phospholipids in very small VLDL (mmol/l)	1268	0.00	-0.15	0.16	0.971	1126	0.02	-0.16	0.20	0.820	873	0.10	-0.05	0.24	0.193
Total cholesterol in very small VLDL (mmol/l)	1268	-0.01	-0.17	0.15	0.906	1126	0.00	-0.19	0.19	0.979	873	0.07	-0.10	0.24	0.395
Cholesterol esters in very small VLDL (mmol/l)	1268	-0.01	-0.17	0.14	0.860	1126	0.00	-0.19	0.19	0.993	873	0.07	-0.10	0.24	0.435
Free cholesterol in very small VLDL (mmol/l)	1268	0.00	-0.15	0.16	0.979	1126	0.01	-0.17	0.19	0.918	873	0.08	-0.09	0.24	0.372
Triglycerides in very small VLDL (mmol/l)	1268	0.06	-0.10	0.21	0.467	1126	0.11	-0.07	0.29	0.243	873	0.13	-0.04	0.29	0.130
Concentration of IDL particles (mol/l)	1268	0.00	-0.15	0.15	0.988	1126	0.02	-0.16	0.19	0.867	873	0.08	-0.07	0.23	0.288
Total lipids in IDL (mmol/l)	1268	-0.01	-0.17	0.14	0.881	1126	0.00	-0.18	0.18	0.966	873	0.07	-0.08	0.21	0.375
Phospholipids in IDL (mmol/l)	1268	-0.01	-0.16	0.14	0.881	1126	-0.01	-0.18	0.17	0.942	873	0.06	-0.09	0.21	0.426
Total cholesterol in IDL (mmol/l)	1268	-0.02	-0.18	0.13	0.775	1126	-0.02	-0.20	0.16	0.839	873	0.05	-0.09	0.20	0.480
Cholesterol esters in IDL (mmol/l)	1268	-0.02	-0.18	0.13	0.763	1126	-0.02	-0.20	0.17	0.849	873	0.05	-0.09	0.19	0.491
Free cholesterol in IDL (mmol/l)	1268	-0.02	-0.17	0.13	0.813	1126	-0.02	-0.20	0.16	0.820	873	0.05	-0.10	0.20	0.489
Triglycerides in IDL (mmol/l)	1268	0.05	-0.10	0.20	0.492	1126	0.09	-0.09	0.26	0.335	873	0.12	-0.04	0.28	0.148
Concentration of large LDL particles (mol/l)	1268	0.00	-0.15	0.15	0.960	1126	0.01	-0.17	0.18	0.944	873	0.07	-0.08	0.23	0.365
Total lipids in large LDL (mmol/l)	1268	-0.01	-0.16	0.14	0.892	1126	0.00	-0.18	0.18	0.981	873	0.06	-0.09	0.21	0.402

S6 Table One-sample MR estimates of associations of age at menarche (per year later) with adiposity and cardiometabolic traits at age 18y among females in ALSPAC, using a full GRS of 351 SNPs for age at menarche

	Unadj.					*Adj. for measured BMI at age 8y*					*Adj. for measured outcome value at age 8y*				
**Standardised outcome at age 18y**	**N**	**Beta (2SLS)**	**LCL**	**UCL**	**P-value**	**N**	**Beta (2SLS)**	**LCL**	**UCL**	**P-value**	**N**	**Beta (2SLS)**	**LCL**	**UCL**	**P-value**
Phospholipids in large LDL (mmol/l)	1268	-0.02	-0.17	0.14	0.837	1126	-0.01	-0.19	0.17	0.931	873	0.06	-0.09	0.21	0.436
Total cholesterol in large LDL (mmol/l)	1268	-0.02	-0.17	0.14	0.827	1126	-0.01	-0.19	0.17	0.895	873	0.06	-0.09	0.20	0.454
Cholesterol esters in large LDL (mmol/l)	1268	-0.02	-0.17	0.14	0.830	1126	-0.01	-0.19	0.17	0.918	873	0.06	-0.09	0.21	0.439
Free cholesterol in large LDL (mmol/l)	1268	-0.02	-0.17	0.13	0.820	1126	-0.02	-0.20	0.16	0.828	873	0.05	-0.10	0.20	0.504
Triglycerides in large LDL (mmol/l)	1268	0.05	-0.10	0.20	0.517	1126	0.08	-0.10	0.25	0.376	873	0.11	-0.05	0.27	0.191
Concentration of medium LDL particles (mol/l)	1268	0.00	-0.15	0.15	0.974	1126	0.01	-0.16	0.19	0.869	873	0.08	-0.08	0.24	0.341
Total lipids in medium LDL (mmol/l)	1268	0.00	-0.16	0.15	0.961	1126	0.01	-0.17	0.18	0.947	873	0.07	-0.08	0.23	0.355
Phospholipids in medium LDL (mmol/l)	1268	-0.01	-0.17	0.14	0.848	1126	0.00	-0.18	0.18	0.976	873	0.05	-0.10	0.20	0.482
Total cholesterol in medium LDL (mmol/l)	1268	-0.01	-0.16	0.14	0.904	1126	0.00	-0.18	0.18	0.973	873	0.07	-0.08	0.22	0.383
Cholesterol esters in medium LDL (mmol/l)	1268	-0.01	-0.16	0.15	0.925	1126	0.00	-0.18	0.18	0.994	873	0.07	-0.08	0.23	0.354
Free cholesterol in medium LDL (mmol/l)	1268	-0.02	-0.17	0.14	0.820	1126	-0.02	-0.20	0.16	0.841	873	0.04	-0.10	0.19	0.559
Triglycerides in medium LDL (mmol/l)	1268	0.06	-0.09	0.21	0.411	1126	0.09	-0.08	0.26	0.309	873	0.12	-0.04	0.29	0.150
Concentration of small LDL particles (mol/l)	1268	0.00	-0.15	0.16	0.970	1126	0.01	-0.16	0.19	0.888	873	0.07	-0.09	0.23	0.399
Total lipids in small LDL (mmol/l)	1268	0.00	-0.16	0.15	0.963	1126	0.01	-0.17	0.18	0.955	873	0.07	-0.09	0.22	0.379
Phospholipids in small LDL (mmol/l)	1268	-0.01	-0.17	0.14	0.880	1126	0.00	-0.18	0.18	0.998	873	0.05	-0.10	0.20	0.536
Total cholesterol in small LDL (mmol/l)	1268	-0.01	-0.16	0.14	0.908	1126	-0.01	-0.18	0.17	0.952	873	0.07	-0.09	0.22	0.403
Cholesterol esters in small LDL (mmol/l)	1268	-0.01	-0.16	0.14	0.917	1126	0.00	-0.18	0.17	0.978	873	0.07	-0.09	0.23	0.382
Free cholesterol in small LDL (mmol/l)	1268	-0.01	-0.17	0.14	0.877	1126	-0.02	-0.20	0.16	0.847	873	0.04	-0.11	0.19	0.600
Triglycerides in small LDL (mmol/l)	1268	0.06	-0.09	0.21	0.443	1126	0.10	-0.08	0.27	0.276	873	0.12	-0.04	0.29	0.143
Concentration of very large HDL particles (mol/l)	1268	-0.04	-0.19	0.11	0.600	1126	-0.05	-0.23	0.13	0.597	873	-0.05	-0.21	0.11	0.525
Total lipids in very large HDL (mmol/l)	1268	-0.04	-0.19	0.12	0.645	1126	-0.05	-0.23	0.14	0.634	873	-0.05	-0.21	0.11	0.559
Phospholipids in very large HDL (mmol/l)	1268	-0.04	-0.19	0.11	0.564	1126	-0.06	-0.23	0.12	0.544	873	-0.06	-0.21	0.09	0.435
Total cholesterol in very large HDL (mmol/l)	1268	-0.03	-0.19	0.13	0.732	1126	-0.04	-0.23	0.15	0.711	873	-0.04	-0.21	0.14	0.688
Cholesterol esters in very large HDL (mmol/l)	1268	-0.02	-0.18	0.14	0.811	1126	-0.03	-0.22	0.16	0.783	873	-0.02	-0.20	0.16	0.800
Free cholesterol in very large HDL (mmol/l)	1268	-0.05	-0.21	0.11	0.534	1126	-0.06	-0.25	0.13	0.534	873	-0.07	-0.23	0.10	0.415
Triglycerides in very large HDL (mmol/l)	1268	0.02	-0.13	0.16	0.820	1126	0.06	-0.11	0.23	0.511	873	0.05	-0.10	0.21	0.515
Concentration of large HDL particles (mol/l)	1268	-0.04	-0.19	0.10	0.561	1126	-0.06	-0.24	0.11	0.476	873	-0.08	-0.23	0.07	0.314
Total lipids in large HDL (mmol/l)	1268	-0.05	-0.20	0.10	0.494	1126	-0.07	-0.25	0.10	0.413	873	-0.09	-0.24	0.06	0.243
Phospholipids in large HDL (mmol/l)	1268	-0.05	-0.19	0.10	0.529	1126	-0.07	-0.24	0.11	0.447	873	-0.08	-0.23	0.07	0.313
Total cholesterol in large HDL (mmol/l)	1268	-0.05	-0.20	0.09	0.474	1126	-0.08	-0.26	0.10	0.384	873	-0.10	-0.25	0.05	0.192
Cholesterol esters in large HDL (mmol/l)	1268	-0.05	-0.20	0.10	0.486	1126	-0.08	-0.26	0.10	0.399	873	-0.10	-0.25	0.05	0.195
Free cholesterol in large HDL (mmol/l)	1268	-0.06	-0.21	0.09	0.431	1126	-0.09	-0.27	0.09	0.334	873	-0.10	-0.25	0.05	0.185
Triglycerides in large HDL (mmol/l)	1268	-0.04	-0.18	0.11	0.644	1126	0.00	-0.17	0.17	0.976	873	0.01	-0.14	0.16	0.896
Concentration of medium HDL particles (mol/l)	1268	-0.03	-0.19	0.13	0.759	1126	-0.03	-0.22	0.16	0.735	873	-0.05	-0.23	0.13	0.603
Total lipids in medium HDL (mmol/l)	1268	-0.04	-0.20	0.12	0.652	1126	-0.05	-0.24	0.14	0.611	873	-0.07	-0.25	0.12	0.469
Phospholipids in medium HDL (mmol/l)	1268	-0.01	-0.17	0.15	0.866	1126	-0.02	-0.21	0.17	0.840	873	-0.03	-0.22	0.15	0.705
Total cholesterol in medium HDL (mmol/l)	1268	-0.07	-0.22	0.09	0.400	1126	-0.10	-0.28	0.09	0.312	873	-0.12	-0.30	0.06	0.203
Cholesterol esters in medium HDL (mmol/l)	1268	-0.07	-0.23	0.09	0.393	1126	-0.10	-0.29	0.09	0.298	873	-0.12	-0.31	0.06	0.183
Free cholesterol in medium HDL (mmol/l)	1268	-0.06	-0.21	0.10	0.456	1126	-0.08	-0.26	0.11	0.406	873	-0.09	-0.27	0.09	0.324
Triglycerides in medium HDL (mmol/l)	1268	0.04	-0.13	0.20	0.671	1126	0.09	-0.09	0.28	0.323	873	0.09	-0.08	0.25	0.299
Concentration of small HDL particles (mol/l)	1268	0.02	-0.15	0.19	0.849	1126	0.02	-0.18	0.22	0.842	873	0.00	-0.19	0.19	0.972
Total lipids in small HDL (mmol/l)	1268	0.03	-0.14	0.19	0.762	1126	0.03	-0.17	0.22	0.799	873	0.02	-0.17	0.21	0.826
Phospholipids in small HDL (mmol/l)	1268	-0.01	-0.18	0.16	0.910	1126	-0.01	-0.21	0.19	0.896	873	-0.05	-0.24	0.14	0.602
Total cholesterol in small HDL (mmol/l)	1268	0.04	-0.11	0.19	0.563	1126	0.04	-0.14	0.22	0.673	873	0.06	-0.11	0.23	0.458
Cholesterol esters in small HDL (mmol/l)	1268	0.06	-0.09	0.20	0.451	1126	0.05	-0.12	0.22	0.558	873	0.09	-0.07	0.26	0.275
Free cholesterol in small HDL (mmol/l)	1268	-0.01	-0.18	0.16	0.909	1126	-0.02	-0.22	0.18	0.852	873	-0.05	-0.24	0.14	0.587
Triglycerides in small HDL (mmol/l)	1268	0.09	-0.07	0.25	0.269	1126	0.15	-0.04	0.34	0.119	873	0.15	-0.02	0.32	0.085
Phospholipids to total lipds ratio in chylomicrons and extremely large VLDL (%)	1268	-0.04	-0.12	0.04	0.359	1126	-0.04	-0.14	0.05	0.362	873	-0.04	-0.14	0.05	0.339
Total cholesterol to total lipids ratio in chylomicrons and extremely large VLDL (%)	1268	0.12	-0.02	0.26	0.097	1126	0.18	0.02	0.35	0.029	873	0.17	0.01	0.33	0.042
Cholesterol esters to total lipids ratio in chylomicrons and extremely large VLDL (%)	1268	0.13	-0.01	0.28	0.068	1126	0.19	0.03	0.36	0.023	873	0.17	0.01	0.34	0.038
Free cholesterol to total lipids ratio in chylomicrons and extremely large VLDL (%)	1268	-0.02	-0.15	0.11	0.752	1126	0.00	-0.14	0.14	0.973	873	0.00	-0.14	0.14	0.961
Triglycerides to total lipids ratio in chylomicrons and extremely large VLDL (%)	1268	-0.08	-0.20	0.03	0.156	1126	-0.14	-0.27	0.00	0.048	873	-0.12	-0.25	0.02	0.084
Phospholipids to total lipds ratio in very large VLDL (%)	1268	-0.02	-0.15	0.11	0.766	1126	0.00	-0.15	0.15	0.987	873	0.04	-0.10	0.18	0.569
Total cholesterol to total lipids ratio in very large VLDL (%)	1268	-0.02	-0.14	0.11	0.789	1126	-0.03	-0.18	0.12	0.692	873	-0.05	-0.20	0.10	0.496
Cholesterol esters to total lipids ratio in very large VLDL (%)	1268	0.00	-0.10	0.09	0.936	1126	-0.01	-0.12	0.10	0.803	873	-0.04	-0.15	0.07	0.446
Free cholesterol to total lipids ratio in very large VLDL (%)	1268	-0.03	-0.15	0.09	0.616	1126	-0.04	-0.18	0.10	0.570	873	-0.02	-0.14	0.11	0.770

S6 Table One-sample MR estimates of associations of age at menarche (per year later) with adiposity and cardiometabolic traits at age 18y among females in ALSPAC, using a full GRS of 351 SNPs for age at menarche

	Unadj.					*Adj. for measured BMI at age 8y*					*Adj. for measured outcome value at age 8y*				
**Standardised outcome at age 18y**	**N**	**Beta (2SLS)**	**LCL**	**UCL**	**P-value**	**N**	**Beta (2SLS)**	**LCL**	**UCL**	**P-value**	**N**	**Beta (2SLS)**	**LCL**	**UCL**	**P-value**
Triglycerides to total lipids ratio in very large VLDL (%)	1268	0.03	-0.12	0.18	0.667	1126	0.04	-0.13	0.21	0.633	873	0.04	-0.12	0.21	0.610
Phospholipids to total lipids ratio in large VLDL (%)	1268	0.02	-0.12	0.16	0.792	1126	0.05	-0.10	0.21	0.499	873	0.09	-0.06	0.24	0.241
Total cholesterol to total lipids ratio in large VLDL (%)	1268	0.02	-0.12	0.17	0.750	1126	0.07	-0.09	0.23	0.405	873	0.13	-0.03	0.29	0.103
Cholesterol esters to total lipids ratio in large VLDL (%)	1268	0.01	-0.14	0.16	0.898	1126	0.04	-0.13	0.21	0.658	873	0.09	-0.08	0.26	0.288
Free cholesterol to total lipids ratio in large VLDL (%)	1268	0.01	-0.05	0.07	0.700	1126	0.03	-0.04	0.10	0.394	873	0.04	-0.02	0.11	0.210
Triglycerides to total lipids ratio in large VLDL (%)	1268	-0.03	-0.17	0.11	0.671	1126	-0.07	-0.23	0.08	0.350	873	-0.14	-0.29	0.01	0.071
Phospholipids to total lipds ratio in medium VLDL (%)	1268	-0.04	-0.19	0.11	0.606	1126	-0.07	-0.25	0.11	0.453	873	-0.01	-0.17	0.14	0.856
Total cholesterol to total lipids ratio in medium VLDL (%)	1268	0.01	-0.13	0.16	0.848	1126	0.05	-0.12	0.22	0.578	873	0.10	-0.05	0.24	0.198
Cholesterol esters to total lipids ratio in medium VLDL (%)	1268	0.01	-0.14	0.16	0.886	1126	0.04	-0.14	0.21	0.673	873	0.08	-0.08	0.23	0.331
Free cholesterol to total lipids ratio in medium VLDL (%)	1268	0.02	-0.12	0.16	0.791	1126	0.06	-0.10	0.22	0.478	873	0.09	-0.06	0.23	0.257
Triglycerides to total lipids ratio in medium VLDL (%)	1268	-0.01	-0.15	0.14	0.945	1126	-0.03	-0.19	0.14	0.723	873	-0.08	-0.23	0.06	0.249
Phospholipids to total lipds ratio in small VLDL (%)	1268	-0.01	-0.15	0.14	0.941	1126	-0.04	-0.22	0.13	0.630	873	-0.11	-0.27	0.05	0.164
Total cholesterol to total lipids ratio in small VLDL (%)	1268	-0.03	-0.18	0.12	0.701	1126	-0.06	-0.24	0.13	0.555	873	0.01	-0.16	0.18	0.933
Cholesterol esters to total lipids ratio in small VLDL (%)	1268	-0.03	-0.19	0.13	0.715	1126	-0.05	-0.24	0.13	0.583	873	0.02	-0.16	0.19	0.861
Free cholesterol to total lipids ratio in small VLDL (%)	1268	-0.01	-0.16	0.13	0.852	1126	-0.03	-0.20	0.14	0.736	873	-0.05	-0.21	0.11	0.547
Triglycerides to total lipids ratio in small VLDL (%)	1268	0.03	-0.12	0.18	0.669	1126	0.07	-0.10	0.25	0.428	873	0.03	-0.13	0.20	0.703
Phospholipids to total lipds ratio in very small VLDL (%)	1268	-0.02	-0.16	0.11	0.714	1126	-0.02	-0.18	0.13	0.761	873	0.02	-0.12	0.16	0.782
Total cholesterol to total lipids ratio in very small VLDL (%)	1268	-0.04	-0.18	0.11	0.620	1126	-0.07	-0.24	0.10	0.432	873	-0.06	-0.22	0.10	0.453
Cholesterol esters to total lipids ratio in very small VLDL (%)	1268	-0.03	-0.18	0.12	0.661	1126	-0.05	-0.23	0.12	0.541	873	-0.06	-0.22	0.11	0.508
Free cholesterol to total lipids ratio in very small VLDL (%)	1268	-0.03	-0.15	0.10	0.687	1126	-0.07	-0.21	0.07	0.335	873	-0.05	-0.20	0.09	0.460
Triglycerides to total lipids ratio in very small VLDL (%)	1268	0.06	-0.09	0.22	0.431	1126	0.10	-0.08	0.28	0.282	873	0.07	-0.10	0.24	0.423
Phospholipids to total lipds ratio in IDL (%)	1268	-0.01	-0.15	0.12	0.847	1126	-0.05	-0.21	0.11	0.556	873	-0.05	-0.20	0.10	0.526
Total cholesterol to total lipids ratio in IDL (%)	1268	-0.06	-0.21	0.09	0.439	1126	-0.08	-0.26	0.10	0.406	873	-0.06	-0.22	0.11	0.492
Cholesterol esters to total lipids ratio in IDL (%)	1268	-0.04	-0.18	0.10	0.580	1126	-0.04	-0.21	0.13	0.648	873	-0.04	-0.19	0.12	0.626
Free cholesterol to total lipids ratio in IDL (%)	1268	-0.05	-0.18	0.08	0.428	1126	-0.09	-0.25	0.06	0.226	873	-0.05	-0.18	0.09	0.489
Triglycerides to total lipids ratio in IDL (%)	1268	0.08	-0.08	0.23	0.338	1126	0.11	-0.07	0.30	0.242	873	0.09	-0.07	0.26	0.275
Phospholipids to total lipds ratio in large LDL (%)	1268	0.01	-0.12	0.13	0.900	1126	0.00	-0.15	0.14	0.977	873	-0.06	-0.18	0.07	0.380
Total cholesterol to total lipids ratio in large LDL (%)	1268	-0.05	-0.19	0.09	0.460	1126	-0.07	-0.23	0.10	0.441	873	-0.02	-0.15	0.12	0.819
Cholesterol esters to total lipids ratio in large LDL (%)	1268	-0.04	-0.17	0.09	0.574	1126	-0.03	-0.19	0.12	0.675	873	0.02	-0.10	0.14	0.733
Free cholesterol to total lipids ratio in large LDL (%)	1268	-0.02	-0.15	0.10	0.747	1126	-0.07	-0.21	0.08	0.361	873	-0.09	-0.22	0.05	0.199
Triglycerides to total lipids ratio in large LDL (%)	1268	0.08	-0.08	0.23	0.331	1126	0.11	-0.08	0.29	0.248	873	0.09	-0.07	0.25	0.288
Phospholipids to total lipds ratio in medium LDL (%)	1268	0.00	-0.12	0.12	0.984	1126	0.00	-0.14	0.14	0.967	873	-0.07	-0.20	0.05	0.270
Total cholesterol to total lipids ratio in medium LDL (%)	1268	-0.03	-0.16	0.10	0.616	1126	-0.04	-0.19	0.11	0.597	873	0.02	-0.10	0.15	0.712
Cholesterol esters to total lipids ratio in medium LDL (%)	1268	-0.02	-0.14	0.11	0.768	1126	-0.01	-0.16	0.13	0.842	873	0.06	-0.07	0.18	0.369
Free cholesterol to total lipids ratio in medium LDL (%)	1268	0.00	-0.12	0.11	0.938	1126	-0.03	-0.16	0.11	0.712	873	-0.08	-0.21	0.04	0.186
Triglycerides to total lipids ratio in medium LDL (%)	1268	0.07	-0.06	0.20	0.297	1126	0.09	-0.06	0.25	0.236	873	0.09	-0.05	0.23	0.226
Phospholipids to total lipds ratio in small LDL (%)	1268	0.00	-0.12	0.13	0.953	1126	0.00	-0.14	0.14	0.982	873	-0.07	-0.20	0.05	0.260
Total cholesterol to total lipids ratio in small LDL (%)	1268	-0.03	-0.16	0.10	0.634	1126	-0.05	-0.20	0.11	0.561	873	0.03	-0.10	0.15	0.686
Cholesterol esters to total lipids ratio in small LDL (%)	1268	-0.02	-0.15	0.10	0.733	1126	-0.02	-0.17	0.12	0.765	873	0.05	-0.07	0.18	0.401
Free cholesterol to total lipids ratio in small LDL (%)	1268	0.00	-0.12	0.12	0.994	1126	-0.02	-0.16	0.11	0.740	873	-0.07	-0.20	0.06	0.275
Triglycerides to total lipids ratio in small LDL (%)	1268	0.08	-0.06	0.23	0.264	1126	0.13	-0.04	0.30	0.138	873	0.11	-0.05	0.27	0.168
Phospholipids to total lipds ratio in very large HDL (%)	1268	-0.03	-0.14	0.09	0.653	1126	-0.05	-0.19	0.09	0.477	873	-0.06	-0.17	0.06	0.353
Total cholesterol to total lipids ratio in very large HDL (%)	1268	0.02	-0.09	0.14	0.692	1126	0.04	-0.10	0.17	0.592	873	0.05	-0.07	0.16	0.437
Cholesterol esters to total lipids ratio in very large HDL (%)	1268	0.03	-0.09	0.15	0.592	1126	0.05	-0.09	0.19	0.511	873	0.06	-0.06	0.18	0.333
Free cholesterol to total lipids ratio in very large HDL (%)	1268	-0.10	-0.25	0.04	0.165	1126	-0.12	-0.29	0.05	0.184	873	-0.15	-0.32	0.02	0.081
Triglycerides to total lipids ratio in very large HDL (%)	1268	0.02	-0.11	0.15	0.756	1126	0.07	-0.08	0.23	0.347	873	0.06	-0.09	0.20	0.448
Phospholipids to total lipds ratio in large HDL (%)	1268	0.04	-0.10	0.18	0.600	1126	0.07	-0.10	0.23	0.438	873	0.11	-0.05	0.26	0.170
Total cholesterol to total lipids ratio in large HDL (%)	1268	-0.02	-0.16	0.11	0.748	1126	-0.06	-0.22	0.10	0.467	873	-0.09	-0.24	0.05	0.196
Cholesterol esters to total lipids ratio in large HDL (%)	1268	-0.02	-0.15	0.12	0.820	1126	-0.05	-0.21	0.11	0.553	873	-0.09	-0.24	0.05	0.211
Free cholesterol to total lipids ratio in large HDL (%)	1268	-0.04	-0.18	0.10	0.542	1126	-0.09	-0.25	0.07	0.286	873	-0.08	-0.22	0.06	0.263
Triglycerides to total lipids ratio in large HDL (%)	1268	-0.01	-0.14	0.11	0.853	1126	0.04	-0.11	0.18	0.630	873	0.05	-0.09	0.18	0.504
Phospholipids to total lipds ratio in medium HDL (%)	1268	0.07	-0.08	0.22	0.353	1126	0.09	-0.09	0.26	0.341	873	0.09	-0.08	0.26	0.308
Total cholesterol to total lipids ratio in medium HDL (%)	1268	-0.07	-0.22	0.08	0.352	1126	-0.11	-0.29	0.06	0.212	873	-0.11	-0.28	0.05	0.182
Cholesterol esters to total lipids ratio in medium HDL (%)	1268	-0.06	-0.21	0.08	0.393	1126	-0.11	-0.28	0.07	0.232	873	-0.11	-0.28	0.05	0.189
Free cholesterol to total lipids ratio in medium HDL (%)	1268	-0.06	-0.19	0.06	0.309	1126	-0.08	-0.22	0.06	0.275	873	-0.07	-0.20	0.06	0.307
Triglycerides to total lipids ratio in medium HDL (%)	1268	0.03	-0.11	0.17	0.666	1126	0.09	-0.07	0.26	0.278	873	0.09	-0.06	0.25	0.220

S6 Table One-sample MR estimates of associations of age at menarche (per year later) with adiposity and cardiometabolic traits at age 18y among females in ALSPAC, using a full GRS of 351 SNPs for age at menarche

	Unadj.					*Adj. for measured BMI at age 8y*					*Adj. for measured outcome value at age 8y*				
**Standardised outcome at age 18y**	**N**	**Beta (2SLS)**	**LCL**	**UCL**	**P-value**	**N**	**Beta (2SLS)**	**LCL**	**UCL**	**P-value**	**N**	**Beta (2SLS)**	**LCL**	**UCL**	**P-value**
Phospholipids to total lipids ratio in small HDL (%)	1268	-0.06	-0.20	0.07	0.363	1126	-0.07	-0.23	0.09	0.407	873	-0.13	-0.28	0.02	0.083
Total cholesterol to total lipids ratio in small HDL (%)	1268	0.04	-0.09	0.17	0.529	1126	0.03	-0.12	0.18	0.698	873	0.09	-0.05	0.23	0.219
Cholesterol esters to total lipids ratio in small HDL (%)	1268	0.05	-0.08	0.18	0.455	1126	0.04	-0.11	0.20	0.589	873	0.11	-0.04	0.26	0.141
Free cholesterol to total lipids ratio in small HDL (%)	1268	-0.07	-0.22	0.07	0.318	1126	-0.10	-0.27	0.08	0.279	873	-0.18	-0.35	-0.01	0.036
Triglycerides to total lipids ratio in small HDL (%)	1268	0.07	-0.07	0.21	0.338	1126	0.13	-0.04	0.29	0.127	873	0.13	-0.02	0.29	0.095
Mean diameter for VLDL particles (nm)	1268	0.03	-0.11	0.16	0.683	1126	0.08	-0.08	0.24	0.317	873	0.06	-0.08	0.21	0.403
Mean diameter for LDL particles (nm)	1268	0.00	-0.14	0.15	0.945	1126	0.03	-0.14	0.19	0.767	873	0.02	-0.14	0.18	0.828
Mean diameter for HDL particles (nm)	1268	-0.05	-0.19	0.09	0.500	1126	-0.07	-0.24	0.10	0.422	873	-0.07	-0.21	0.07	0.337
Serum total cholesterol (mmol/l)	1268	-0.02	-0.17	0.13	0.781	1126	-0.01	-0.19	0.16	0.883	873	0.04	-0.10	0.19	0.571
Total cholesterol in VLDL (mmol/l)	1268	0.01	-0.14	0.16	0.876	1126	0.06	-0.11	0.24	0.493	873	0.09	-0.05	0.24	0.216
Remnant cholesterol (non-HDL, non-LDL -cholesterol) (mmol/l)	1268	0.00	-0.16	0.15	0.965	1126	0.03	-0.15	0.21	0.757	873	0.08	-0.06	0.23	0.261
Total cholesterol in LDL (mmol/l)	1268	-0.01	-0.17	0.14	0.866	1126	-0.01	-0.19	0.17	0.930	873	0.06	-0.09	0.21	0.421
Total cholesterol in HDL (mmol/l)	1268	-0.04	-0.19	0.11	0.620	1126	-0.06	-0.24	0.12	0.514	873	-0.07	-0.23	0.09	0.397
Total cholesterol in HDL2 (mmol/l)	1268	-0.04	-0.19	0.11	0.606	1126	-0.07	-0.25	0.11	0.448	873	-0.08	-0.24	0.08	0.330
Total cholesterol in HDL3 (mmol/l)	1268	-0.03	-0.19	0.12	0.663	1126	-0.04	-0.22	0.14	0.663	873	-0.05	-0.21	0.11	0.567
Esterified cholesterol (mmol/l)	1262	-0.03	-0.18	0.12	0.712	1122	-0.03	-0.21	0.15	0.774	870	0.03	-0.13	0.18	0.742
Free cholesterol (mmol/l)	1262	-0.02	-0.17	0.12	0.772	1122	-0.01	-0.18	0.16	0.921	870	0.05	-0.09	0.19	0.467
Serum total triglycerides (mmol/l)	1268	0.03	-0.11	0.18	0.633	1126	0.10	-0.07	0.26	0.259	873	0.09	-0.06	0.24	0.223
Triglycerides in VLDL (mmol/l)	1268	0.02	-0.11	0.16	0.738	1126	0.08	-0.08	0.24	0.311	873	0.07	-0.07	0.21	0.325
Triglycerides in LDL (mmol/l)	1268	0.06	-0.09	0.21	0.467	1126	0.09	-0.09	0.26	0.332	873	0.12	-0.05	0.28	0.167
Triglycerides in HDL (mmol/l)	1268	0.05	-0.11	0.20	0.545	1126	0.11	-0.07	0.29	0.235	873	0.11	-0.05	0.27	0.189
Diacylglycerol (mmol/l)	1227	-0.04	-0.19	0.11	0.604	1090	0.01	-0.17	0.18	0.947	831	-0.03	-0.20	0.15	0.776
Ratio of diacylglycerol to triglycerides	1228	-0.04	-0.19	0.11	0.572	1091	-0.02	-0.20	0.16	0.839	832	-0.08	-0.26	0.10	0.377
Total phosphoglycerides (mmol/l)	1262	-0.01	-0.16	0.14	0.898	1122	0.01	-0.17	0.19	0.939	870	0.05	-0.11	0.22	0.528
Ratio of triglycerides to phosphoglycerides	1262	0.04	-0.09	0.17	0.538	1122	0.10	-0.05	0.26	0.188	870	0.07	-0.08	0.21	0.379
Phosphatidylcholine and other cholines (mmol/l)	1250	0.01	-0.14	0.16	0.885	1111	0.02	-0.16	0.20	0.819	858	0.02	-0.15	0.18	0.860
Total cholines (mmol/l)	1262	-0.01	-0.16	0.14	0.926	1122	0.00	-0.18	0.17	0.992	870	0.03	-0.13	0.19	0.690
Apolipoprotein A-I (g/l)	1268	-0.04	-0.19	0.12	0.641	1126	-0.04	-0.22	0.14	0.639	873	-0.04	-0.20	0.12	0.640
Apolipoprotein B (g/l)	1268	0.01	-0.14	0.16	0.896	1126	0.05	-0.13	0.23	0.575	873	0.10	-0.05	0.24	0.200
Ratio of apolipoprotein B to apolipoprotein A-I	1268	0.02	-0.13	0.16	0.835	1126	0.06	-0.11	0.23	0.504	873	0.10	-0.04	0.25	0.164
Total fatty acids (mmol/l)	1262	0.00	-0.14	0.15	0.979	1122	0.03	-0.14	0.20	0.714	870	0.05	-0.11	0.21	0.522
Estimated description of fatty acid chain length, not actual carbon number	1262	0.05	-0.09	0.19	0.500	1122	0.09	-0.08	0.25	0.295	870	0.10	-0.06	0.27	0.232
Estimated degree of unsaturation	1263	0.02	-0.11	0.16	0.721	1123	0.03	-0.13	0.19	0.716	871	0.00	-0.16	0.16	0.994
22:6, docosahexaenoic acid (mmol/l)	1262	0.04	-0.12	0.19	0.641	1122	0.06	-0.12	0.24	0.525	870	0.07	-0.10	0.24	0.405
18:2, linoleic acid (mmol/l)	1261	0.00	-0.15	0.15	0.967	1121	0.00	-0.18	0.17	0.978	869	0.04	-0.11	0.19	0.604
Conjugated linoleic acid (mmol/l)	1262	0.09	-0.04	0.22	0.177	1122	0.17	0.02	0.32	0.026	869	0.16	0.01	0.31	0.036
Omega-3 fatty acids (mmol/l)	1262	0.01	-0.13	0.16	0.854	1122	0.07	-0.10	0.24	0.441	870	0.02	-0.14	0.19	0.801
Omega-6 fatty acids (mmol/l)	1262	-0.01	-0.16	0.14	0.868	1122	-0.01	-0.18	0.17	0.924	870	0.03	-0.12	0.18	0.718
Polyunsaturated fatty acids (mmol/l)	1261	-0.01	-0.16	0.14	0.890	1121	0.00	-0.17	0.17	0.994	869	0.03	-0.13	0.18	0.735
Monounsaturated fatty acids; 16:1, 18:1 (mmol/l)	1262	0.01	-0.13	0.16	0.851	1122	0.05	-0.12	0.22	0.568	870	0.07	-0.08	0.23	0.359
Saturated fatty acids (mmol/l)	1261	0.00	-0.15	0.15	0.997	1121	0.04	-0.14	0.21	0.686	869	0.04	-0.12	0.20	0.634
Ratio of 22:6 docosahexaenoic acid to total fatty acids (%)	1263	0.04	-0.11	0.19	0.592	1123	0.05	-0.12	0.22	0.546	871	0.05	-0.12	0.22	0.545
Ratio of 18:2 linoleic acid to total fatty acids (%)	1262	-0.01	-0.16	0.13	0.868	1122	-0.07	-0.23	0.09	0.401	870	-0.03	-0.20	0.13	0.688
Ratio of conjugated linoleic acid to total fatty acids (%)	1263	0.10	-0.03	0.23	0.132	1123	0.18	0.03	0.32	0.020	870	0.18	0.03	0.33	0.022
Ratio of omega-3 fatty acids to total fatty acids (%)	1263	0.02	-0.12	0.17	0.755	1123	0.07	-0.10	0.25	0.410	871	0.00	-0.18	0.17	0.979
Ratio of omega-6 fatty acids to total fatty acids (%)	1263	-0.03	-0.17	0.11	0.662	1123	-0.09	-0.25	0.07	0.265	871	-0.07	-0.23	0.09	0.386
Ratio of polyunsaturated fatty acids to total fatty acids (%)	1262	-0.02	-0.17	0.12	0.744	1122	-0.07	-0.23	0.10	0.414	870	-0.06	-0.23	0.10	0.433
Ratio of monounsaturated fatty acids to total fatty acids (%)	1263	0.03	-0.12	0.17	0.725	1123	0.05	-0.12	0.21	0.589	871	0.08	-0.08	0.25	0.306
Ratio of saturated fatty acids to total fatty acids (%)	1262	0.00	-0.14	0.13	0.953	1122	0.02	-0.13	0.18	0.765	870	-0.04	-0.19	0.12	0.628
Glucose (mmol/l)	1267	-0.04	-0.14	0.06	0.459	1125	-0.02	-0.13	0.10	0.798	867	-0.07	-0.19	0.05	0.280
Lactate (mmol/l)	1267	0.10	-0.05	0.25	0.183	1125	0.14	-0.04	0.32	0.116	872	0.07	-0.10	0.24	0.421
Pyruvate (mmol/l)	1267	0.07	-0.06	0.20	0.294	1125	0.12	-0.04	0.28	0.131	870	0.05	-0.10	0.21	0.480
Citrate (mmol/l)	1267	0.24	0.09	0.40	1.77E-03	1125	0.19	0.01	0.36	0.033	870	0.17	0.00	0.34	0.045
Alanine (mmol/l)	1267	0.13	-0.02	0.29	0.087	1125	0.15	-0.03	0.32	0.108	872	0.04	-0.14	0.22	0.659
Glutamine (mmol/l)	1267	0.16	0.02	0.31	0.023	1125	0.16	0.00	0.33	0.054	870	0.06	-0.11	0.22	0.484
Histidine (mmol/l)	1267	0.07	-0.09	0.24	0.389	1125	0.09	-0.11	0.28	0.383	871	0.04	-0.13	0.21	0.658

S6 Table One-sample MR estimates of associations of age at menarche (per year later) with adiposity and cardiometabolic traits at age 18y among females in ALSPAC, using a full GRS of 351 SNPs for age at menarche

	Unadj.					*Adj. for measured BMI at age 8y*					*Adj. for measured outcome value at age 8y*				
**Standardised outcome at age 18y**	**N**	**Beta (2SLS)**	**LCL**	**UCL**	**P-value**	**N**	**Beta (2SLS)**	**LCL**	**UCL**	**P-value**	**N**	**Beta (2SLS)**	**LCL**	**UCL**	**P-value**
Isoleucine (mmol/l)	1267	0.04	-0.08	0.16	0.542	1125	0.06	-0.08	0.20	0.379	872	0.00	-0.14	0.14	0.980
Leucine (mmol/l)	1267	0.05	-0.06	0.17	0.373	1125	0.06	-0.08	0.19	0.410	872	0.00	-0.14	0.13	0.969
Valine (mmol/l)	1267	-0.02	-0.15	0.10	0.707	1125	0.01	-0.14	0.16	0.932	872	-0.09	-0.24	0.06	0.250
Phenylalanine (mmol/l)	1266	0.01	-0.13	0.15	0.894	1124	0.02	-0.14	0.18	0.801	870	-0.06	-0.22	0.11	0.502
Tyrosine (mmol/l)	1267	-0.01	-0.18	0.16	0.908	1125	0.05	-0.14	0.25	0.581	869	-0.11	-0.31	0.08	0.257
Acetate (mmol/l)	1266	0.02	-0.12	0.16	0.768	1124	0.02	-0.14	0.18	0.789	872	0.02	-0.17	0.21	0.824
Acetoacetate (mmol/l)	1267	0.00	-0.12	0.11	0.948	1125	-0.05	-0.18	0.09	0.514	871	0.01	-0.13	0.15	0.889
3-hydroxybutyrate (mmol/l)	1265	0.00	-0.15	0.15	0.984	1123	-0.06	-0.23	0.10	0.456	869	0.01	-0.17	0.19	0.891
Creatinine (mmol/l)	1267	0.00	-0.11	0.11	0.955	1125	-0.01	-0.14	0.11	0.820	870	0.02	-0.10	0.14	0.761
Albumin (signal area)	1268	-0.07	-0.21	0.07	0.310	1126	-0.15	-0.31	0.01	0.065	871	-0.17	-0.33	-0.01	0.033
Glycoprotein acetyls, mainly a1-acid glycoprotein (mmol/l)	1267	0.05	-0.10	0.20	0.535	1125	0.13	-0.05	0.30	0.155	872	0.11	-0.05	0.28	0.180

Complete case sample

	Unadj.					*Adj. for measured BMI at age 8y*					*Adj. for measured outcome value at age 8y*				
**Standardised outcome at age 18y**	**N**	**Beta (2SLS)**	**LCL**	**UCL**	**P-value**	**N**	**Beta (2SLS)**	**LCL**	**UCL**	**P-value**	**N**	**Beta (2SLS)**	**LCL**	**UCL**	**P-value**
Body mass index (kg/m^2)	629	-0.29	-0.48	-0.10	0.002	629	-0.13	-0.29	0.04	0.127	629	-0.13	-0.29	0.04	0.127
Fat mass index (kg/m^2)	629	-0.22	-0.39	-0.05	0.011	629	-0.08	-0.23	0.08	0.325	629	-0.05	-0.19	0.09	0.483
Lean mass index (kg/m^2)	629	-0.17	-0.28	-0.05	0.004	629	-0.11	-0.22	0.01	0.062	629	0.00	-0.09	0.09	0.941
Systolic blood pressure (mmHg)	629	-0.07	-0.22	0.09	0.401	629	-0.03	-0.20	0.14	0.740	629	-0.03	-0.18	0.13	0.731
Diastolic blood pressure (mmHg)	629	-0.01	-0.18	0.15	0.868	629	0.04	-0.15	0.22	0.692	629	0.02	-0.14	0.19	0.789
Concentration of chylomicrons and extremely large VLDL particles (mol/l)	629	0.09	-0.06	0.23	0.231	629	0.14	-0.02	0.30	0.088	629	0.09	-0.05	0.22	0.219
Total lipids in chylomicrons and extremely large VLDL (mmol/l)	629	0.09	-0.06	0.23	0.237	629	0.14	-0.02	0.30	0.095	629	0.08	-0.05	0.22	0.229
Phospholipids in chylomicrons and extremely large VLDL (mmol/l)	629	0.09	-0.06	0.23	0.236	629	0.14	-0.02	0.30	0.097	629	0.08	-0.05	0.22	0.232
Total cholesterol in chylomicrons and extremely large VLDL (mmol/l)	629	0.10	-0.05	0.25	0.204	629	0.15	-0.02	0.32	0.088	629	0.09	-0.05	0.24	0.195
Cholesterol esters in chylomicrons and extremely large VLDL (mmol/l)	629	0.10	-0.05	0.26	0.198	629	0.15	-0.02	0.33	0.092	629	0.10	-0.05	0.25	0.185
Free cholesterol in chylomicrons and extremely large VLDL (mmol/l)	629	0.09	-0.06	0.23	0.233	629	0.14	-0.02	0.30	0.096	629	0.09	-0.05	0.22	0.229
Triglycerides in chylomicrons and extremely large VLDL (mmol/l)	629	0.08	-0.06	0.23	0.249	629	0.13	-0.02	0.29	0.098	629	0.08	-0.05	0.22	0.241
Concentration of very large VLDL particles (mol/l)	629	0.08	-0.07	0.22	0.286	629	0.12	-0.03	0.28	0.125	629	0.07	-0.06	0.21	0.285
Total lipids in very large VLDL (mmol/l)	629	0.08	-0.06	0.23	0.275	629	0.13	-0.03	0.28	0.125	629	0.08	-0.06	0.21	0.274
Phospholipids in very large VLDL (mmol/l)	629	0.08	-0.06	0.23	0.266	629	0.13	-0.03	0.29	0.121	629	0.08	-0.06	0.22	0.267
Total cholesterol in very large VLDL (mmol/l)	629	0.09	-0.06	0.24	0.249	629	0.14	-0.03	0.30	0.103	629	0.08	-0.05	0.22	0.236
Cholesterol esters in very large VLDL (mmol/l)	629	0.09	-0.06	0.24	0.266	629	0.13	-0.03	0.30	0.110	629	0.08	-0.06	0.22	0.248
Free cholesterol in very large VLDL (mmol/l)	629	0.09	-0.06	0.24	0.235	629	0.14	-0.03	0.30	0.099	629	0.09	-0.05	0.23	0.227
Triglycerides in very large VLDL (mmol/l)	629	0.08	-0.07	0.22	0.292	629	0.12	-0.04	0.28	0.139	629	0.07	-0.06	0.21	0.295
Concentration of large VLDL particles (mol/l)	629	0.08	-0.07	0.23	0.306	629	0.12	-0.05	0.28	0.163	629	0.07	-0.07	0.22	0.305
Total lipids in large VLDL (mmol/l)	629	0.08	-0.07	0.23	0.307	629	0.12	-0.05	0.29	0.162	629	0.07	-0.07	0.22	0.304
Phospholipids in large VLDL (mmol/l)	629	0.08	-0.07	0.23	0.292	629	0.12	-0.05	0.29	0.154	629	0.08	-0.07	0.22	0.289
Total cholesterol in large VLDL (mmol/l)	629	0.09	-0.07	0.24	0.276	629	0.13	-0.04	0.30	0.138	629	0.08	-0.06	0.23	0.267
Cholesterol esters in large VLDL (mmol/l)	629	0.09	-0.07	0.25	0.271	629	0.13	-0.04	0.31	0.133	629	0.09	-0.06	0.23	0.251
Free cholesterol in large VLDL (mmol/l)	629	0.08	-0.07	0.23	0.285	629	0.12	-0.04	0.29	0.148	629	0.08	-0.06	0.22	0.286
Triglycerides in large VLDL (mmol/l)	629	0.08	-0.08	0.23	0.329	629	0.11	-0.05	0.28	0.179	629	0.07	-0.07	0.21	0.330
Concentration of medium VLDL particles (mol/l)	629	0.08	-0.08	0.24	0.340	629	0.12	-0.06	0.29	0.186	629	0.08	-0.07	0.22	0.314
Total lipids in medium VLDL (mmol/l)	629	0.08	-0.08	0.24	0.337	629	0.12	-0.06	0.30	0.182	629	0.08	-0.07	0.22	0.309
Phospholipids in medium VLDL (mmol/l)	629	0.09	-0.08	0.25	0.302	629	0.13	-0.05	0.31	0.168	629	0.08	-0.07	0.24	0.272
Total cholesterol in medium VLDL (mmol/l)	629	0.09	-0.08	0.26	0.294	629	0.13	-0.05	0.32	0.159	629	0.09	-0.06	0.24	0.259
Cholesterol esters in medium VLDL (mmol/l)	629	0.09	-0.09	0.26	0.322	629	0.13	-0.06	0.33	0.176	629	0.08	-0.07	0.24	0.287
Free cholesterol in medium VLDL (mmol/l)	629	0.09	-0.07	0.25	0.283	629	0.13	-0.05	0.31	0.161	629	0.09	-0.06	0.24	0.257
Triglycerides in medium VLDL (mmol/l)	629	0.07	-0.09	0.22	0.394	629	0.11	-0.06	0.28	0.220	629	0.07	-0.08	0.21	0.371
Concentration of small VLDL particles (mol/l)	629	0.12	-0.06	0.30	0.184	629	0.16	-0.04	0.36	0.113	629	0.13	-0.04	0.29	0.133
Total lipids in small VLDL (mmol/l)	629	0.13	-0.06	0.31	0.186	629	0.17	-0.04	0.37	0.111	629	0.13	-0.03	0.30	0.117
Phospholipids in small VLDL (mmol/l)	629	0.13	-0.06	0.33	0.173	629	0.17	-0.05	0.39	0.121	629	0.14	-0.04	0.32	0.123
Total cholesterol in small VLDL (mmol/l)	629	0.12	-0.08	0.32	0.225	629	0.17	-0.05	0.39	0.132	629	0.14	-0.03	0.32	0.106
Cholesterol esters in small VLDL (mmol/l)	629	0.11	-0.09	0.32	0.280	629	0.16	-0.06	0.39	0.161	629	0.14	-0.04	0.31	0.129
Free cholesterol in small VLDL (mmol/l)	629	0.13	-0.06	0.32	0.179	629	0.17	-0.05	0.38	0.122	629	0.14	-0.03	0.31	0.116
Triglycerides in small VLDL (mmol/l)	629	0.11	-0.07	0.28	0.229	629	0.14	-0.05	0.33	0.149	629	0.11	-0.05	0.27	0.185

S6 Table One-sample MR estimates of associations of age at menarche (per year later) with adiposity and cardiometabolic traits at age 18y among females in ALSPAC, using a full GRS of 351 SNPs for age at menarche

	Unadj.					*Adj. for measured BMI at age 8y*					*Adj. for measured outcome value at age 8y*				
**Standardised outcome at age 18y**	**N**	**Beta (2SLS)**	**LCL**	**UCL**	**P-value**	**N**	**Beta (2SLS)**	**LCL**	**UCL**	**P-value**	**N**	**Beta (2SLS)**	**LCL**	**UCL**	**P-value**
Concentration of very small VLDL particles (mol/l)	629	0.10	-0.09	0.30	0.304	629	0.14	-0.09	0.36	0.232	629	0.13	-0.05	0.30	0.154
Total lipids in very small VLDL (mmol/l)	629	0.09	-0.11	0.30	0.366	629	0.14	-0.10	0.37	0.253	629	0.12	-0.06	0.30	0.179
Phospholipids in very small VLDL (mmol/l)	629	0.08	-0.12	0.28	0.418	629	0.11	-0.11	0.34	0.328	629	0.10	-0.07	0.27	0.235
Total cholesterol in very small VLDL (mmol/l)	629	0.06	-0.15	0.27	0.589	629	0.10	-0.13	0.34	0.401	629	0.08	-0.11	0.27	0.397
Cholesterol esters in very small VLDL (mmol/l)	629	0.06	-0.15	0.27	0.592	629	0.11	-0.13	0.34	0.379	629	0.08	-0.11	0.27	0.392
Free cholesterol in very small VLDL (mmol/l)	629	0.05	-0.15	0.25	0.617	629	0.08	-0.15	0.31	0.507	629	0.07	-0.12	0.26	0.470
Triglycerides in very small VLDL (mmol/l)	629	0.14	-0.05	0.34	0.147	629	0.17	-0.05	0.39	0.127	629	0.15	-0.03	0.34	0.109
Concentration of IDL particles (mol/l)	629	0.09	-0.11	0.29	0.377	629	0.12	-0.11	0.34	0.302	629	0.10	-0.07	0.28	0.244
Total lipids in IDL (mmol/l)	629	0.07	-0.13	0.27	0.499	629	0.10	-0.13	0.32	0.395	629	0.08	-0.09	0.25	0.347
Phospholipids in IDL (mmol/l)	629	0.07	-0.12	0.27	0.469	629	0.10	-0.12	0.32	0.386	629	0.08	-0.10	0.26	0.369
Total cholesterol in IDL (mmol/l)	629	0.05	-0.15	0.25	0.624	629	0.08	-0.14	0.31	0.478	629	0.06	-0.11	0.23	0.468
Cholesterol esters in IDL (mmol/l)	629	0.05	-0.15	0.25	0.646	629	0.08	-0.15	0.31	0.479	629	0.06	-0.11	0.23	0.481
Free cholesterol in IDL (mmol/l)	629	0.06	-0.14	0.25	0.577	629	0.08	-0.14	0.30	0.484	629	0.07	-0.11	0.24	0.454
Triglycerides in IDL (mmol/l)	629	0.14	-0.06	0.33	0.168	629	0.15	-0.07	0.36	0.194	629	0.15	-0.04	0.33	0.120
Concentration of large LDL particles (mol/l)	629	0.08	-0.11	0.28	0.416	629	0.11	-0.12	0.33	0.346	629	0.09	-0.09	0.27	0.335
Total lipids in large LDL (mmol/l)	629	0.07	-0.13	0.27	0.481	629	0.10	-0.13	0.32	0.395	629	0.08	-0.10	0.25	0.386
Phospholipids in large LDL (mmol/l)	629	0.06	-0.13	0.26	0.520	629	0.09	-0.13	0.32	0.421	629	0.07	-0.11	0.24	0.436
Total cholesterol in large LDL (mmol/l)	629	0.06	-0.14	0.26	0.545	629	0.09	-0.14	0.31	0.438	629	0.07	-0.11	0.24	0.449
Cholesterol esters in large LDL (mmol/l)	629	0.06	-0.13	0.26	0.532	629	0.09	-0.13	0.32	0.421	629	0.07	-0.10	0.24	0.440
Free cholesterol in large LDL (mmol/l)	629	0.05	-0.14	0.25	0.589	629	0.08	-0.15	0.30	0.496	629	0.06	-0.11	0.24	0.481
Triglycerides in large LDL (mmol/l)	629	0.13	-0.06	0.32	0.184	629	0.14	-0.08	0.36	0.221	629	0.14	-0.05	0.33	0.151
Concentration of medium LDL particles (mol/l)	629	0.09	-0.10	0.29	0.360	629	0.12	-0.10	0.34	0.297	629	0.10	-0.09	0.28	0.305
Total lipids in medium LDL (mmol/l)	629	0.08	-0.12	0.28	0.437	629	0.11	-0.12	0.33	0.358	629	0.09	-0.09	0.27	0.355
Phospholipids in medium LDL (mmol/l)	629	0.06	-0.14	0.26	0.561	629	0.09	-0.14	0.31	0.456	629	0.06	-0.11	0.24	0.494
Total cholesterol in medium LDL (mmol/l)	629	0.07	-0.13	0.27	0.480	629	0.10	-0.13	0.33	0.383	629	0.08	-0.10	0.26	0.389
Cholesterol esters in medium LDL (mmol/l)	629	0.08	-0.12	0.28	0.441	629	0.11	-0.12	0.33	0.349	629	0.08	-0.10	0.26	0.359
Free cholesterol in medium LDL (mmol/l)	629	0.04	-0.16	0.24	0.667	629	0.07	-0.16	0.30	0.558	629	0.05	-0.12	0.23	0.549
Triglycerides in medium LDL (mmol/l)	629	0.15	-0.05	0.34	0.136	629	0.15	-0.07	0.37	0.173	629	0.15	-0.04	0.34	0.125
Concentration of small LDL particles (mol/l)	629	0.09	-0.11	0.29	0.395	629	0.11	-0.12	0.34	0.333	629	0.09	-0.10	0.28	0.351
Total lipids in small LDL (mmol/l)	629	0.08	-0.12	0.28	0.443	629	0.11	-0.12	0.33	0.362	629	0.08	-0.10	0.27	0.372
Phospholipids in small LDL (mmol/l)	629	0.06	-0.14	0.26	0.549	629	0.09	-0.14	0.31	0.461	629	0.06	-0.12	0.24	0.512
Total cholesterol in small LDL (mmol/l)	629	0.07	-0.13	0.27	0.494	629	0.10	-0.13	0.33	0.397	629	0.08	-0.10	0.26	0.409
Cholesterol esters in small LDL (mmol/l)	629	0.08	-0.12	0.27	0.448	629	0.11	-0.12	0.33	0.356	629	0.08	-0.10	0.26	0.378
Free cholesterol in small LDL (mmol/l)	629	0.04	-0.17	0.24	0.727	629	0.06	-0.17	0.29	0.617	629	0.05	-0.13	0.22	0.607
Triglycerides in small LDL (mmol/l)	629	0.16	-0.04	0.35	0.111	629	0.18	-0.05	0.40	0.120	629	0.16	-0.03	0.35	0.106
Concentration of very large HDL particles (mol/l)	629	-0.02	-0.23	0.18	0.823	629	-0.04	-0.27	0.18	0.701	629	-0.04	-0.21	0.14	0.666
Total lipids in very large HDL (mmol/l)	629	-0.02	-0.23	0.19	0.854	629	-0.04	-0.27	0.20	0.746	629	-0.03	-0.22	0.15	0.735
Phospholipids in very large HDL (mmol/l)	629	-0.03	-0.23	0.16	0.733	629	-0.06	-0.28	0.16	0.583	629	-0.05	-0.22	0.11	0.518
Total cholesterol in very large HDL (mmol/l)	629	-0.01	-0.23	0.21	0.934	629	-0.02	-0.26	0.22	0.877	629	-0.01	-0.21	0.19	0.906
Cholesterol esters in very large HDL (mmol/l)	629	0.00	-0.22	0.22	0.995	629	-0.01	-0.25	0.24	0.962	629	0.00	-0.20	0.20	0.991
Free cholesterol in very large HDL (mmol/l)	629	-0.04	-0.25	0.17	0.733	629	-0.06	-0.29	0.18	0.642	629	-0.05	-0.23	0.14	0.607
Triglycerides in very large HDL (mmol/l)	629	0.08	-0.10	0.27	0.371	629	0.10	-0.11	0.31	0.347	629	0.08	-0.09	0.26	0.341
Concentration of large HDL particles (mol/l)	629	-0.04	-0.24	0.15	0.656	629	-0.08	-0.30	0.13	0.445	629	-0.08	-0.25	0.08	0.314
Total lipids in large HDL (mmol/l)	629	-0.06	-0.25	0.14	0.559	629	-0.10	-0.31	0.11	0.365	629	-0.10	-0.26	0.07	0.244
Phospholipids in large HDL (mmol/l)	629	-0.04	-0.23	0.15	0.654	629	-0.08	-0.29	0.13	0.447	629	-0.09	-0.25	0.08	0.304
Total cholesterol in large HDL (mmol/l)	629	-0.07	-0.27	0.12	0.473	629	-0.12	-0.33	0.10	0.295	629	-0.11	-0.27	0.06	0.204
Cholesterol esters in large HDL (mmol/l)	629	-0.07	-0.27	0.13	0.484	629	-0.11	-0.33	0.10	0.302	629	-0.10	-0.27	0.06	0.211
Free cholesterol in large HDL (mmol/l)	629	-0.08	-0.27	0.12	0.435	629	-0.12	-0.33	0.09	0.271	629	-0.11	-0.27	0.05	0.180
Triglycerides in large HDL (mmol/l)	629	0.06	-0.12	0.24	0.511	629	0.06	-0.14	0.26	0.556	629	0.03	-0.13	0.20	0.708
Concentration of medium HDL particles (mol/l)	629	-0.01	-0.22	0.19	0.905	629	-0.04	-0.27	0.19	0.740	629	-0.04	-0.25	0.16	0.685
Total lipids in medium HDL (mmol/l)	629	-0.03	-0.23	0.18	0.785	629	-0.06	-0.28	0.17	0.618	629	-0.06	-0.26	0.14	0.545
Phospholipids in medium HDL (mmol/l)	629	0.00	-0.21	0.20	0.981	629	-0.03	-0.26	0.20	0.799	629	-0.03	-0.23	0.17	0.781
Total cholesterol in medium HDL (mmol/l)	629	-0.08	-0.28	0.13	0.460	629	-0.11	-0.34	0.11	0.329	629	-0.12	-0.32	0.08	0.235
Cholesterol esters in medium HDL (mmol/l)	629	-0.08	-0.29	0.12	0.438	629	-0.12	-0.34	0.11	0.308	629	-0.13	-0.33	0.07	0.212
Free cholesterol in medium HDL (mmol/l)	629	-0.05	-0.25	0.14	0.590	629	-0.08	-0.30	0.14	0.465	629	-0.09	-0.29	0.11	0.367
Triglycerides in medium HDL (mmol/l)	629	0.13	-0.07	0.32	0.199	629	0.15	-0.06	0.37	0.161	629	0.11	-0.07	0.30	0.235

S6 Table One-sample MR estimates of associations of age at menarche (per year later) with adiposity and cardiometabolic traits at age 18y among females in ALSPAC, using a full GRS of 351 SNPs for age at menarche

	Unadj.					*Adj. for measured BMI at age 8y*					*Adj. for measured outcome value at age 8y*				
**Standardised outcome at age 18y**	**N**	**Beta (2SLS)**	**LCL**	**UCL**	**P-value**	**N**	**Beta (2SLS)**	**LCL**	**UCL**	**P-value**	**N**	**Beta (2SLS)**	**LCL**	**UCL**	**P-value**
Concentration of small HDL particles (mol/l)	629	0.04	-0.18	0.25	0.729	629	0.03	-0.21	0.26	0.830	629	0.02	-0.19	0.23	0.863
Total lipids in small HDL (mmol/l)	629	0.04	-0.17	0.25	0.697	629	0.02	-0.21	0.26	0.839	629	0.03	-0.18	0.24	0.784
Phospholipids in small HDL (mmol/l)	629	0.00	-0.22	0.21	0.967	629	-0.02	-0.26	0.21	0.856	629	-0.03	-0.24	0.18	0.777
Total cholesterol in small HDL (mmol/l)	629	0.06	-0.14	0.25	0.560	629	0.04	-0.18	0.26	0.725	629	0.05	-0.14	0.24	0.594
Cholesterol esters in small HDL (mmol/l)	629	0.08	-0.12	0.27	0.440	629	0.06	-0.15	0.27	0.572	629	0.07	-0.11	0.26	0.444
Free cholesterol in small HDL (mmol/l)	629	-0.02	-0.23	0.19	0.867	629	-0.05	-0.29	0.19	0.678	629	-0.04	-0.25	0.17	0.689
Triglycerides in small HDL (mmol/l)	629	0.16	-0.03	0.36	0.103	629	0.19	-0.03	0.41	0.095	629	0.16	-0.03	0.36	0.094
Phospholipids to total lipds ratio in chylomicrons and extremely large VLDL (%)	629	-0.01	-0.11	0.09	0.894	629	0.00	-0.12	0.11	0.959	629	-0.01	-0.11	0.09	0.854
Total cholesterol to total lipids ratio in chylomicrons and extremely large VLDL (%)	629	0.18	0.00	0.35	0.049	629	0.20	0.00	0.40	0.046	629	0.17	0.00	0.34	0.053
Cholesterol esters to total lipids ratio in chylomicrons and extremely large VLDL (%)	629	0.17	0.00	0.35	0.056	629	0.19	-0.01	0.39	0.059	629	0.17	-0.01	0.34	0.059
Free cholesterol to total lipids ratio in chylomicrons and extremely large VLDL (%)	629	0.06	-0.11	0.22	0.489	629	0.08	-0.10	0.26	0.387	629	0.05	-0.11	0.22	0.521
Triglycerides to total lipids ratio in chylomicrons and extremely large VLDL (%)	629	-0.14	-0.29	0.00	0.056	629	-0.17	-0.33	0.00	0.048	629	-0.14	-0.28	0.01	0.062
Phospholipids to total lipids ratio in very large VLDL (%)	629	0.07	-0.10	0.24	0.412	629	0.10	-0.09	0.29	0.282	629	0.06	-0.11	0.22	0.499
Total cholesterol to total lipids ratio in very large VLDL (%)	629	-0.05	-0.21	0.10	0.509	629	-0.05	-0.23	0.12	0.564	629	-0.07	-0.22	0.08	0.386
Cholesterol esters to total lipids ratio in very large VLDL (%)	629	-0.05	-0.17	0.08	0.450	629	-0.05	-0.19	0.09	0.474	629	-0.05	-0.17	0.06	0.372
Free cholesterol to total lipids ratio in very large VLDL (%)	629	-0.03	-0.16	0.11	0.709	629	-0.02	-0.17	0.13	0.789	629	-0.04	-0.17	0.09	0.541
Triglycerides to total lipids ratio in very large VLDL (%)	629	0.05	-0.14	0.23	0.624	629	0.03	-0.17	0.23	0.742	629	0.07	-0.10	0.24	0.438
Phospholipids to total lipds ratio in large VLDL (%)	629	0.12	-0.06	0.29	0.194	629	0.14	-0.05	0.34	0.146	629	0.12	-0.05	0.29	0.173
Total cholesterol to total lipids ratio in large VLDL (%)	629	0.16	-0.02	0.33	0.081	629	0.20	0.01	0.40	0.041	629	0.15	-0.02	0.31	0.089
Cholesterol esters to total lipids ratio in large VLDL (%)	629	0.12	-0.06	0.29	0.198	629	0.15	-0.04	0.34	0.121	629	0.11	-0.07	0.28	0.227
Free cholesterol to total lipids ratio in large VLDL (%)	629	0.05	-0.02	0.13	0.175	629	0.07	-0.02	0.16	0.121	629	0.05	-0.02	0.13	0.151
Triglycerides to total lipids ratio in large VLDL (%)	629	-0.16	-0.33	0.01	0.072	629	-0.20	-0.39	-0.01	0.041	629	-0.15	-0.32	0.01	0.073
Phospholipids to total lipds ratio in medium VLDL (%)	629	0.02	-0.16	0.20	0.822	629	0.00	-0.20	0.20	0.974	629	0.02	-0.15	0.19	0.805
Total cholesterol to total lipids ratio in medium VLDL (%)	629	0.10	-0.06	0.27	0.225	629	0.13	-0.05	0.32	0.166	629	0.10	-0.05	0.26	0.199
Cholesterol esters to total lipids ratio in medium VLDL (%)	629	0.08	-0.09	0.25	0.337	629	0.11	-0.08	0.30	0.251	629	0.08	-0.08	0.24	0.321
Free cholesterol to total lipids ratio in medium VLDL (%)	629	0.12	-0.06	0.30	0.193	629	0.14	-0.06	0.34	0.183	629	0.12	-0.05	0.29	0.165
Triglycerides to total lipids ratio in medium VLDL (%)	629	-0.10	-0.27	0.07	0.256	629	-0.12	-0.31	0.07	0.215	629	-0.10	-0.25	0.06	0.226
Phospholipids to total lipds ratio in small VLDL (%)	629	-0.07	-0.25	0.11	0.453	629	-0.12	-0.32	0.07	0.223	629	-0.09	-0.26	0.07	0.266
Total cholesterol to total lipids ratio in small VLDL (%)	629	-0.01	-0.20	0.19	0.959	629	0.02	-0.20	0.23	0.880	629	0.01	-0.18	0.19	0.951
Cholesterol esters to total lipids ratio in small VLDL (%)	629	-0.01	-0.20	0.19	0.960	629	0.02	-0.20	0.24	0.843	629	0.01	-0.18	0.20	0.929
Free cholesterol to total lipids ratio in small VLDL (%)	629	0.00	-0.18	0.17	0.962	629	-0.04	-0.23	0.16	0.712	629	-0.01	-0.18	0.15	0.867
Triglycerides to total lipids ratio in small VLDL (%)	629	0.03	-0.16	0.22	0.763	629	0.03	-0.18	0.23	0.813	629	0.03	-0.15	0.20	0.782
Phospholipids to total lipds ratio in very small VLDL (%)	629	0.03	-0.13	0.18	0.729	629	0.03	-0.15	0.20	0.773	629	0.02	-0.13	0.17	0.772
Total cholesterol to total lipids ratio in very small VLDL (%)	629	-0.09	-0.28	0.09	0.327	629	-0.08	-0.29	0.13	0.446	629	-0.09	-0.28	0.09	0.319
Cholesterol esters to total lipids ratio in very small VLDL (%)	629	-0.07	-0.26	0.12	0.466	629	-0.05	-0.26	0.16	0.659	629	-0.07	-0.26	0.11	0.437
Free cholesterol to total lipids ratio in very small VLDL (%)	629	-0.11	-0.26	0.04	0.156	629	-0.14	-0.32	0.03	0.101	629	-0.11	-0.26	0.04	0.154
Triglycerides to total lipids ratio in very small VLDL (%)	629	0.09	-0.10	0.29	0.341	629	0.08	-0.13	0.30	0.462	629	0.10	-0.09	0.29	0.312
Phospholipids to total lipds ratio in IDL (%)	629	-0.01	-0.17	0.16	0.920	629	-0.05	-0.23	0.13	0.620	629	-0.01	-0.17	0.15	0.905
Total cholesterol to total lipids ratio in IDL (%)	629	-0.08	-0.26	0.11	0.434	629	-0.04	-0.25	0.17	0.698	629	-0.09	-0.27	0.09	0.340
Cholesterol esters to total lipids ratio in IDL (%)	629	-0.07	-0.24	0.11	0.455	629	-0.03	-0.22	0.17	0.772	629	-0.08	-0.25	0.09	0.379
Free cholesterol to total lipids ratio in IDL (%)	629	-0.02	-0.17	0.12	0.755	629	-0.03	-0.20	0.13	0.703	629	-0.03	-0.17	0.11	0.646
Triglycerides to total lipids ratio in IDL (%)	629	0.09	-0.10	0.29	0.348	629	0.07	-0.15	0.28	0.537	629	0.11	-0.07	0.30	0.226
Phospholipids to total lipds ratio in large LDL (%)	629	-0.08	-0.22	0.06	0.275	629	-0.10	-0.26	0.06	0.209	629	-0.09	-0.22	0.04	0.190
Total cholesterol to total lipids ratio in large LDL (%)	629	0.00	-0.16	0.16	0.989	629	0.03	-0.15	0.21	0.726	629	-0.01	-0.15	0.12	0.840
Cholesterol esters to total lipids ratio in large LDL (%)	629	0.04	-0.12	0.19	0.655	629	0.07	-0.10	0.25	0.413	629	0.02	-0.10	0.15	0.705
Free cholesterol to total lipids ratio in large LDL (%)	629	-0.11	-0.26	0.05	0.170	629	-0.13	-0.31	0.04	0.126	629	-0.11	-0.26	0.04	0.145
Triglycerides to total lipids ratio in large LDL (%)	629	0.09	-0.10	0.28	0.348	629	0.06	-0.15	0.27	0.560	629	0.11	-0.06	0.28	0.218
Phospholipids to total lipds ratio in medium LDL (%)	629	-0.09	-0.22	0.05	0.198	629	-0.11	-0.27	0.04	0.145	629	-0.10	-0.23	0.03	0.140
Total cholesterol to total lipids ratio in medium LDL (%)	629	0.03	-0.11	0.18	0.666	629	0.07	-0.10	0.23	0.433	629	0.03	-0.10	0.16	0.627
Cholesterol esters to total lipids ratio in medium LDL (%)	629	0.07	-0.07	0.21	0.345	629	0.10	-0.06	0.26	0.214	629	0.07	-0.06	0.20	0.275
Free cholesterol to total lipids ratio in medium LDL (%)	629	-0.11	-0.25	0.03	0.130	629	-0.13	-0.29	0.02	0.093	629	-0.11	-0.25	0.02	0.098
Triglycerides to total lipids ratio in medium LDL (%)	629	0.10	-0.06	0.27	0.215	629	0.08	-0.10	0.26	0.385	629	0.12	-0.04	0.27	0.149
Phospholipids to total lipds ratio in small LDL (%)	629	-0.08	-0.22	0.06	0.276	629	-0.11	-0.27	0.05	0.188	629	-0.09	-0.22	0.04	0.193
Total cholesterol to total lipids ratio in small LDL (%)	629	0.03	-0.12	0.18	0.725	629	0.06	-0.11	0.22	0.511	629	0.03	-0.10	0.17	0.647
Cholesterol esters to total lipids ratio in small LDL (%)	629	0.06	-0.08	0.21	0.391	629	0.09	-0.07	0.26	0.259	629	0.07	-0.07	0.20	0.318
Free cholesterol to total lipids ratio in small LDL (%)	629	-0.11	-0.25	0.03	0.115	629	-0.14	-0.30	0.02	0.086	629	-0.11	-0.25	0.02	0.101

S6 Table One-sample MR estimates of associations of age at menarche (per year later) with adiposity and cardiometabolic traits at age 18y among females in ALSPAC, using a full GRS of 351 SNPs for age at menarche

	Unadj.					*Adj. for measured BMI at age 8y*					*Adj. for measured outcome value at age 8y*				
**Standardised outcome at age 18y**	**N**	**Beta (2SLS)**	**LCL**	**UCL**	**P-value**	**N**	**Beta (2SLS)**	**LCL**	**UCL**	**P-value**	**N**	**Beta (2SLS)**	**LCL**	**UCL**	**P-value**
Triglycerides to total lipids ratio in small LDL (%)	629	0.14	-0.04	0.32	0.134	629	0.13	-0.07	0.33	0.200	629	0.14	-0.03	0.32	0.111
Phospholipids to total lipds ratio in very large HDL (%)	629	-0.04	-0.18	0.10	0.602	629	-0.07	-0.23	0.08	0.350	629	-0.05	-0.17	0.08	0.472
Total cholesterol to total lipids ratio in very large HDL (%)	629	0.02	-0.12	0.16	0.746	629	0.05	-0.10	0.21	0.488	629	0.03	-0.09	0.15	0.619
Cholesterol esters to total lipids ratio in very large HDL (%)	629	0.03	-0.11	0.18	0.643	629	0.07	-0.09	0.22	0.419	629	0.04	-0.09	0.17	0.525
Free cholesterol to total lipids ratio in very large HDL (%)	629	-0.12	-0.32	0.07	0.216	629	-0.14	-0.35	0.08	0.224	629	-0.12	-0.32	0.07	0.218
Triglycerides to total lipids ratio in very large HDL (%)	629	0.08	-0.09	0.25	0.366	629	0.11	-0.07	0.30	0.230	629	0.08	-0.08	0.24	0.324
Phospholipids to total lipds ratio in large HDL (%)	629	0.14	-0.05	0.33	0.155	629	0.19	-0.03	0.40	0.091	629	0.13	-0.06	0.31	0.175
Total cholesterol to total lipids ratio in large HDL (%)	629	-0.13	-0.32	0.05	0.165	629	-0.18	-0.39	0.03	0.086	629	-0.12	-0.28	0.05	0.169
Cholesterol esters to total lipids ratio in large HDL (%)	629	-0.13	-0.32	0.07	0.197	629	-0.17	-0.39	0.04	0.105	629	-0.11	-0.28	0.06	0.200
Free cholesterol to total lipids ratio in large HDL (%)	629	-0.12	-0.29	0.04	0.148	629	-0.16	-0.34	0.03	0.092	629	-0.11	-0.27	0.04	0.150
Triglycerides to total lipids ratio in large HDL (%)	629	0.08	-0.08	0.24	0.303	629	0.13	-0.05	0.30	0.155	629	0.08	-0.06	0.22	0.262
Phospholipids to total lipds ratio in medium HDL (%)	629	0.10	-0.10	0.29	0.345	629	0.09	-0.13	0.32	0.416	629	0.10	-0.10	0.29	0.332
Total cholesterol to total lipids ratio in medium HDL (%)	629	-0.14	-0.34	0.05	0.150	629	-0.16	-0.38	0.06	0.152	629	-0.14	-0.33	0.05	0.139
Cholesterol esters to total lipids ratio in medium HDL (%)	629	-0.14	-0.33	0.05	0.159	629	-0.16	-0.38	0.06	0.156	629	-0.14	-0.32	0.05	0.149
Free cholesterol to total lipids ratio in medium HDL (%)	629	-0.08	-0.24	0.07	0.300	629	-0.08	-0.25	0.09	0.361	629	-0.09	-0.23	0.06	0.247
Triglycerides to total lipids ratio in medium HDL (%)	629	0.14	-0.04	0.32	0.138	629	0.18	-0.02	0.38	0.078	629	0.13	-0.03	0.30	0.106
Phospholipids to total lipds ratio in small HDL (%)	629	-0.09	-0.27	0.08	0.291	629	-0.09	-0.29	0.10	0.361	629	-0.10	-0.27	0.06	0.209
Total cholesterol to total lipids ratio in small HDL (%)	629	0.05	-0.11	0.21	0.559	629	0.04	-0.15	0.22	0.691	629	0.05	-0.10	0.21	0.481
Cholesterol esters to total lipids ratio in small HDL (%)	629	0.07	-0.10	0.24	0.425	629	0.06	-0.12	0.25	0.509	629	0.07	-0.08	0.23	0.350
Free cholesterol to total lipids ratio in small HDL (%)	629	-0.15	-0.35	0.05	0.153	629	-0.19	-0.41	0.04	0.106	629	-0.16	-0.35	0.03	0.102
Triglycerides to total lipids ratio in small HDL (%)	629	0.15	-0.02	0.32	0.092	629	0.18	-0.01	0.38	0.063	629	0.16	-0.01	0.33	0.064
Mean diameter for VLDL particles (nm)	629	0.08	-0.08	0.25	0.330	629	0.12	-0.06	0.30	0.201	629	0.08	-0.08	0.23	0.342
Mean diameter for LDL particles (nm)	629	-0.02	-0.20	0.16	0.854	629	-0.02	-0.22	0.19	0.852	629	-0.01	-0.19	0.17	0.934
Mean diameter for HDL particles (nm)	629	-0.05	-0.25	0.14	0.577	629	-0.08	-0.30	0.13	0.440	629	-0.07	-0.23	0.09	0.380
Serum total cholesterol (mmol/l)	629	0.06	-0.14	0.25	0.574	629	0.08	-0.15	0.30	0.494	629	0.05	-0.12	0.23	0.547
Total cholesterol in VLDL (mmol/l)	629	0.10	-0.08	0.29	0.279	629	0.15	-0.05	0.36	0.145	629	0.11	-0.05	0.27	0.175
Remnant cholesterol (non-HDL, non-LDL -cholesterol) (mmol/l)	629	0.09	-0.11	0.28	0.386	629	0.13	-0.09	0.35	0.238	629	0.10	-0.07	0.26	0.238
Total cholesterol in LDL (mmol/l)	629	0.07	-0.13	0.26	0.515	629	0.09	-0.13	0.32	0.413	629	0.07	-0.10	0.25	0.421
Total cholesterol in HDL (mmol/l)	629	-0.04	-0.24	0.16	0.702	629	-0.07	-0.30	0.15	0.508	629	-0.07	-0.25	0.10	0.416
Total cholesterol in HDL2 (mmol/l)	629	-0.05	-0.25	0.15	0.617	629	-0.09	-0.31	0.13	0.415	629	-0.08	-0.26	0.09	0.339
Total cholesterol in HDL3 (mmol/l)	629	-0.02	-0.22	0.19	0.875	629	-0.04	-0.27	0.18	0.711	629	-0.05	-0.23	0.13	0.610
Esterified cholesterol (mmol/l)	629	0.06	-0.14	0.27	0.534	629	0.09	-0.14	0.32	0.463	629	0.05	-0.13	0.23	0.599
Free cholesterol (mmol/l)	629	0.03	-0.15	0.22	0.728	629	0.05	-0.16	0.26	0.633	629	0.06	-0.10	0.22	0.471
Serum total triglycerides (mmol/l)	629	0.11	-0.05	0.28	0.185	629	0.15	-0.04	0.33	0.114	629	0.11	-0.05	0.27	0.166
Triglycerides in VLDL (mmol/l)	629	0.09	-0.07	0.25	0.283	629	0.13	-0.05	0.30	0.157	629	0.09	-0.06	0.23	0.262
Triglycerides in LDL (mmol/l)	629	0.14	-0.05	0.34	0.151	629	0.15	-0.07	0.37	0.182	629	0.15	-0.04	0.34	0.133
Triglycerides in HDL (mmol/l)	629	0.14	-0.04	0.33	0.128	629	0.17	-0.04	0.38	0.115	629	0.14	-0.04	0.32	0.136
Diacylglycerol (mmol/l)	629	0.02	-0.18	0.21	0.857	629	0.03	-0.18	0.25	0.757	629	0.01	-0.18	0.21	0.882
Ratio of diacylglycerol to triglycerides	629	-0.02	-0.21	0.18	0.868	629	-0.01	-0.23	0.20	0.902	629	-0.02	-0.21	0.18	0.860
Total phosphoglycerides (mmol/l)	629	0.09	-0.10	0.29	0.347	629	0.10	-0.12	0.32	0.374	629	0.09	-0.10	0.27	0.362
Ratio of triglycerides to phosphoglycerides	629	0.08	-0.08	0.24	0.337	629	0.11	-0.07	0.29	0.223	629	0.07	-0.09	0.22	0.393
Phosphatidylcholine and other cholines (mmol/l)	629	0.11	-0.08	0.31	0.257	629	0.11	-0.11	0.33	0.319	629	0.09	-0.10	0.28	0.360
Total cholines (mmol/l)	629	0.08	-0.12	0.27	0.452	629	0.08	-0.15	0.30	0.501	629	0.07	-0.12	0.25	0.482
Apolipoprotein A-I (g/l)	629	0.00	-0.21	0.20	0.962	629	-0.03	-0.25	0.20	0.812	629	-0.04	-0.22	0.14	0.679
Apolipoprotein B (g/l)	629	0.11	-0.08	0.30	0.274	629	0.15	-0.06	0.37	0.167	629	0.12	-0.05	0.28	0.168
Ratio of apolipoprotein B to apolipoprotein A-I	629	0.10	-0.08	0.29	0.275	629	0.16	-0.05	0.37	0.136	629	0.13	-0.03	0.29	0.104
Total fatty acids (mmol/l)	629	0.09	-0.10	0.28	0.360	629	0.11	-0.10	0.32	0.312	629	0.08	-0.10	0.26	0.378
Estimated description of fatty acid chain length, not actual carbon number	629	0.13	-0.04	0.30	0.140	629	0.13	-0.06	0.33	0.175	629	0.13	-0.04	0.30	0.141
Estimated degree of unsaturation	629	0.09	-0.07	0.26	0.266	629	0.09	-0.09	0.28	0.330	629	0.08	-0.08	0.24	0.345
22:6, docosahexaenoic acid (mmol/l)	629	0.12	-0.09	0.32	0.268	629	0.12	-0.11	0.35	0.298	629	0.09	-0.11	0.28	0.372
18:2, linoleic acid (mmol/l)	629	0.09	-0.10	0.28	0.343	629	0.10	-0.12	0.32	0.355	629	0.08	-0.10	0.25	0.381
Conjugated linoleic acid (mmol/l)	629	0.14	-0.02	0.30	0.096	629	0.17	-0.01	0.35	0.062	629	0.13	-0.03	0.29	0.106
Omega-3 fatty acids (mmol/l)	629	0.11	-0.09	0.31	0.292	629	0.14	-0.09	0.36	0.236	629	0.06	-0.13	0.26	0.527
Omega-6 fatty acids (mmol/l)	629	0.08	-0.12	0.27	0.437	629	0.09	-0.13	0.31	0.430	629	0.06	-0.12	0.23	0.503
Polyunsaturated fatty acids (mmol/l)	629	0.08	-0.11	0.27	0.394	629	0.10	-0.12	0.32	0.378	629	0.06	-0.11	0.24	0.492
Monounsaturated fatty acids; 16:1, 18:1 (mmol/l)	629	0.11	-0.08	0.29	0.247	629	0.13	-0.07	0.34	0.208	629	0.11	-0.07	0.29	0.234

S6 Table One-sample MR estimates of associations of age at menarche (per year later) with adiposity and cardiometabolic traits at age 18y among females in ALSPAC, using a full GRS of 351 SNPs for age at menarche

	Unadj.					*Adj. for measured BMI at age 8y*					*Adj. for measured outcome value at age 8y*				
**Standardised outcome at age 18y**	**N**	**Beta (2SLS)**	**LCL**	**UCL**	**P-value**	**N**	**Beta (2SLS)**	**LCL**	**UCL**	**P-value**	**N**	**Beta (2SLS)**	**LCL**	**UCL**	**P-value**
Saturated fatty acids (mmol/l)	629	0.05	-0.14	0.25	0.574	629	0.08	-0.14	0.29	0.484	629	0.05	-0.13	0.24	0.589
Ratio of 22:6 docosahexaenoic acid to total fatty acids (%)	629	0.10	-0.10	0.29	0.335	629	0.09	-0.13	0.31	0.416	629	0.06	-0.13	0.25	0.522
Ratio of 18:2 linoleic acid to total fatty acids (%)	629	-0.01	-0.20	0.17	0.902	629	-0.04	-0.24	0.17	0.732	629	-0.02	-0.20	0.16	0.842
Ratio of conjugated linoleic acid to total fatty acids (%)	629	0.12	-0.02	0.26	0.095	629	0.15	-0.01	0.30	0.066	629	0.12	-0.02	0.26	0.102
Ratio of omega-3 fatty acids to total fatty acids (%)	629	0.08	-0.14	0.29	0.485	629	0.09	-0.14	0.33	0.448	629	0.03	-0.18	0.24	0.800
Ratio of omega-6 fatty acids to total fatty acids (%)	629	-0.05	-0.23	0.13	0.587	629	-0.08	-0.27	0.12	0.449	629	-0.06	-0.23	0.12	0.533
Ratio of polyunsaturated fatty acids to total fatty acids (%)	629	-0.03	-0.21	0.15	0.761	629	-0.05	-0.25	0.15	0.629	629	-0.04	-0.22	0.14	0.650
Ratio of monounsaturated fatty acids to total fatty acids (%)	629	0.10	-0.09	0.29	0.301	629	0.12	-0.09	0.33	0.277	629	0.11	-0.08	0.30	0.245
Ratio of saturated fatty acids to total fatty acids (%)	629	-0.10	-0.28	0.08	0.261	629	-0.10	-0.30	0.10	0.334	629	-0.10	-0.28	0.08	0.273
Glucose (mmol/l)	629	-0.07	-0.22	0.08	0.363	629	-0.06	-0.23	0.11	0.491	629	-0.07	-0.21	0.08	0.356
Lactate (mmol/l)	629	0.08	-0.13	0.29	0.445	629	0.09	-0.14	0.32	0.448	629	0.08	-0.13	0.28	0.467
Pyruvate (mmol/l)	629	0.06	-0.12	0.23	0.533	629	0.08	-0.12	0.28	0.411	629	0.05	-0.13	0.23	0.570
Citrate (mmol/l)	629	0.14	-0.06	0.33	0.164	629	0.12	-0.09	0.33	0.270	629	0.14	-0.05	0.32	0.151
Alanine (mmol/l)	629	0.04	-0.17	0.25	0.700	629	0.05	-0.18	0.28	0.659	629	0.03	-0.17	0.24	0.743
Glutamine (mmol/l)	629	0.04	-0.15	0.22	0.696	629	0.03	-0.17	0.23	0.736	629	0.02	-0.16	0.20	0.826
Histidine (mmol/l)	629	0.04	-0.15	0.24	0.659	629	0.05	-0.17	0.27	0.651	629	0.05	-0.15	0.24	0.648
Isoleucine (mmol/l)	629	0.04	-0.11	0.20	0.586	629	0.06	-0.11	0.23	0.482	629	0.02	-0.14	0.18	0.817
Leucine (mmol/l)	629	0.04	-0.11	0.19	0.632	629	0.05	-0.12	0.22	0.568	629	0.02	-0.13	0.17	0.805
Valine (mmol/l)	629	-0.02	-0.19	0.15	0.813	629	0.01	-0.18	0.19	0.958	629	-0.05	-0.22	0.12	0.545
Phenylalanine (mmol/l)	629	-0.06	-0.26	0.13	0.519	629	-0.04	-0.26	0.17	0.685	629	-0.09	-0.29	0.10	0.352
Tyrosine (mmol/l)	629	-0.15	-0.36	0.05	0.144	629	-0.13	-0.36	0.11	0.287	629	-0.17	-0.38	0.04	0.106
Acetate (mmol/l)	629	-0.07	-0.21	0.06	0.301	629	-0.09	-0.23	0.05	0.218	629	-0.08	-0.21	0.06	0.286
Acetoacetate (mmol/l)	629	0.00	-0.17	0.16	0.974	629	-0.02	-0.20	0.17	0.858	629	0.00	-0.17	0.16	0.980
3-hydroxybutyrate (mmol/l)	629	-0.03	-0.24	0.18	0.755	629	-0.06	-0.29	0.17	0.606	629	-0.03	-0.24	0.18	0.753
Creatinine (mmol/l)	629	-0.03	-0.17	0.10	0.651	629	-0.02	-0.17	0.13	0.806	629	0.00	-0.13	0.12	0.940
Albumin (signal area)	629	-0.11	-0.29	0.06	0.204	629	-0.13	-0.32	0.07	0.207	629	-0.10	-0.28	0.07	0.241
Glycoprotein acetyls, mainly a1-acid glycoprotein (mmol/l)	629	0.15	-0.05	0.34	0.140	629	0.20	-0.01	0.41	0.063	629	0.13	-0.06	0.33	0.166
