# Supplementary material for: Influence of puberty timing on adiposity and cardiometabolic traits: A Mendelian randomisation study
Source: PLoS Med. 2018 Aug 28;15(8):e1002641. doi: 10.1371/journal.pmed.1002641 (PMC6112630; doi:10.1371/journal.pmed.1002641)
Supplement: S7 Table — (PDF) [file pmed.1002641.s026.pdf]

S7 Table One-sample MR estimates of associations of puberty timing (per year later) with adiposity and cardiometabolic traits at age 18y among males and females in ALSPAC, using a full GRS of 351 SNPs for age at menarche

	Unadj.					*Adj. for measured BMI at age 8y*					*Adj. for measured outcome value at age 8y*				
**Standardised outcome at age 18y**	**N**	**Beta (2SLS)**	**LCL**	**UCL**	**P-value**	**N**	**Beta (2SLS)**	**LCL**	**UCL**	**P-value**	**N**	**Beta (2SLS)**	**LCL**	**UCL**	**P-value**
Body mass index (kg/m^2)	3498	-0.44	-0.60	-0.28	3.96E-08	3174	-0.08	-0.20	0.05	0.221	3174	-0.08	-0.20	0.05	0.221
Fat mass index (kg/m^2)	3383	-0.16	-0.30	-0.02	0.028	3081	0.14	-0.01	0.29	0.067	2995	0.14	0.01	0.28	0.033
Lean mass index (kg/m^2)	3383	-0.54	-0.74	-0.33	2.47E-07	3081	-0.41	-0.63	-0.20	1.49E-04	2995	-0.08	-0.19	0.04	0.181
Systolic blood pressure (mmHg)	3303	-0.40	-0.59	-0.22	2.00E-05	3012	-0.33	-0.53	-0.13	1.14E-03	2978	-0.31	-0.48	-0.13	6.73E-04
Diastolic blood pressure (mmHg)	3303	-0.10	-0.25	0.05	0.177	3012	-0.01	-0.17	0.16	0.933	2977	-0.06	-0.20	0.09	0.449
Concentration of chylomicrons and extremely large VLDL particles (mol/l)	2333	-0.05	-0.24	0.13	0.576	2110	0.05	-0.17	0.26	0.678	1647	-0.05	-0.27	0.17	0.658
Total lipids in chylomicrons and extremely large VLDL (mmol/l)	2333	-0.05	-0.24	0.13	0.577	2110	0.05	-0.17	0.27	0.677	1647	-0.04	-0.26	0.18	0.703
Phospholipids in chylomicrons and extremely large VLDL (mmol/l)	2333	-0.05	-0.24	0.14	0.606	2110	0.05	-0.17	0.27	0.660	1647	-0.04	-0.26	0.18	0.695
Total cholesterol in chylomicrons and extremely large VLDL (mmol/l)	2333	-0.03	-0.22	0.16	0.743	2110	0.07	-0.15	0.29	0.527	1647	-0.02	-0.23	0.19	0.855
Cholesterol esters in chylomicrons and extremely large VLDL (mmol/l)	2333	-0.02	-0.21	0.17	0.839	2110	0.08	-0.14	0.31	0.464	1647	0.00	-0.21	0.21	0.971
Free cholesterol in chylomicrons and extremely large VLDL (mmol/l)	2333	-0.05	-0.23	0.14	0.637	2110	0.05	-0.16	0.27	0.626	1647	-0.04	-0.26	0.18	0.719
Triglycerides in chylomicrons and extremely large VLDL (mmol/l)	2333	-0.06	-0.25	0.13	0.532	2110	0.04	-0.18	0.26	0.725	1647	-0.05	-0.27	0.17	0.665
Concentration of very large VLDL particles (mol/l)	2333	-0.07	-0.26	0.12	0.455	2110	0.02	-0.20	0.25	0.830	1647	-0.06	-0.29	0.16	0.574
Total lipids in very large VLDL (mmol/l)	2333	-0.07	-0.26	0.12	0.488	2110	0.03	-0.19	0.25	0.797	1647	-0.06	-0.28	0.16	0.610
Phospholipids in very large VLDL (mmol/l)	2333	-0.05	-0.24	0.14	0.602	2110	0.05	-0.18	0.27	0.684	1647	-0.05	-0.27	0.17	0.640
Total cholesterol in very large VLDL (mmol/l)	2333	-0.05	-0.24	0.14	0.586	2110	0.05	-0.17	0.27	0.663	1647	-0.04	-0.26	0.17	0.686
Cholesterol esters in very large VLDL (mmol/l)	2333	-0.06	-0.25	0.13	0.512	2110	0.04	-0.18	0.26	0.731	1647	-0.05	-0.26	0.17	0.664
Free cholesterol in very large VLDL (mmol/l)	2333	-0.04	-0.23	0.15	0.681	2110	0.06	-0.16	0.28	0.589	1647	-0.04	-0.26	0.18	0.720
Triglycerides in very large VLDL (mmol/l)	2333	-0.08	-0.27	0.12	0.436	2110	0.02	-0.20	0.24	0.873	1647	-0.06	-0.29	0.16	0.580
Concentration of large VLDL particles (mol/l)	2333	-0.07	-0.27	0.12	0.455	2110	0.02	-0.21	0.24	0.880	1647	-0.05	-0.28	0.17	0.636
Total lipids in large VLDL (mmol/l)	2333	-0.07	-0.27	0.12	0.479	2110	0.02	-0.20	0.25	0.844	1647	-0.05	-0.27	0.17	0.657
Phospholipids in large VLDL (mmol/l)	2333	-0.06	-0.25	0.13	0.547	2110	0.03	-0.19	0.26	0.764	1647	-0.04	-0.26	0.18	0.704
Total cholesterol in large VLDL (mmol/l)	2333	-0.05	-0.24	0.14	0.614	2110	0.05	-0.17	0.27	0.669	1647	-0.03	-0.25	0.18	0.767
Cholesterol esters in large VLDL (mmol/l)	2333	-0.04	-0.23	0.15	0.668	2110	0.06	-0.16	0.28	0.600	1647	-0.02	-0.23	0.19	0.856
Free cholesterol in large VLDL (mmol/l)	2333	-0.06	-0.25	0.14	0.568	2110	0.04	-0.19	0.26	0.741	1647	-0.05	-0.27	0.18	0.685
Triglycerides in large VLDL (mmol/l)	2333	-0.08	-0.28	0.11	0.415	2110	0.01	-0.22	0.23	0.940	1647	-0.06	-0.28	0.17	0.604
Concentration of medium VLDL particles (mol/l)	2333	-0.08	-0.27	0.12	0.438	2110	0.01	-0.21	0.24	0.918	1647	-0.05	-0.27	0.17	0.655
Total lipids in medium VLDL (mmol/l)	2333	-0.07	-0.26	0.13	0.485	2110	0.02	-0.20	0.25	0.847	1647	-0.04	-0.26	0.17	0.703
Phospholipids in medium VLDL (mmol/l)	2333	-0.05	-0.24	0.14	0.617	2110	0.04	-0.18	0.27	0.714	1647	-0.03	-0.24	0.18	0.799
Total cholesterol in medium VLDL (mmol/l)	2333	-0.01	-0.20	0.18	0.915	2110	0.09	-0.13	0.31	0.441	1647	0.01	-0.20	0.21	0.959
Cholesterol esters in medium VLDL (mmol/l)	2333	0.01	-0.18	0.20	0.906	2110	0.11	-0.11	0.33	0.332	1647	0.02	-0.18	0.22	0.853
Free cholesterol in medium VLDL (mmol/l)	2333	-0.04	-0.23	0.16	0.716	2110	0.06	-0.17	0.28	0.621	1647	-0.02	-0.23	0.20	0.888
Triglycerides in medium VLDL (mmol/l)	2333	-0.10	-0.30	0.09	0.304	2110	-0.02	-0.24	0.21	0.876	1647	-0.07	-0.29	0.15	0.528
Concentration of small VLDL particles (mol/l)	2333	0.01	-0.18	0.20	0.937	2110	0.10	-0.13	0.32	0.400	1647	0.02	-0.18	0.22	0.844
Total lipids in small VLDL (mmol/l)	2333	0.02	-0.17	0.21	0.850	2110	0.10	-0.12	0.32	0.358	1647	0.02	-0.17	0.21	0.824
Phospholipids in small VLDL (mmol/l)	2333	0.05	-0.14	0.24	0.617	2110	0.13	-0.09	0.35	0.251	1647	0.04	-0.15	0.23	0.702
Total cholesterol in small VLDL (mmol/l)	2333	0.05	-0.14	0.23	0.628	2110	0.12	-0.10	0.34	0.273	1647	0.02	-0.16	0.20	0.862
Cholesterol esters in small VLDL (mmol/l)	2333	0.04	-0.15	0.22	0.707	2110	0.10	-0.11	0.32	0.348	1647	0.00	-0.18	0.19	0.962
Free cholesterol in small VLDL (mmol/l)	2333	0.06	-0.13	0.25	0.525	2110	0.14	-0.08	0.37	0.202	1647	0.04	-0.15	0.23	0.672
Triglycerides in small VLDL (mmol/l)	2333	-0.02	-0.22	0.17	0.817	2110	0.06	-0.16	0.28	0.599	1647	0.00	-0.21	0.21	0.982
Concentration of very small VLDL particles (mol/l)	2333	0.15	-0.04	0.34	0.128	2110	0.21	-0.02	0.44	0.068	1647	0.06	-0.11	0.24	0.478
Total lipids in very small VLDL (mmol/l)	2333	0.10	-0.09	0.29	0.304	2110	0.15	-0.07	0.38	0.172	1647	0.02	-0.15	0.20	0.810
Phospholipids in very small VLDL (mmol/l)	2333	0.17	-0.02	0.37	0.081	2110	0.22	-0.01	0.45	0.059	1647	0.06	-0.11	0.23	0.473
Total cholesterol in very small VLDL (mmol/l)	2333	0.02	-0.17	0.21	0.840	2110	0.06	-0.16	0.28	0.595	1647	-0.01	-0.20	0.19	0.934
Cholesterol esters in very small VLDL (mmol/l)	2333	-0.02	-0.21	0.17	0.862	2110	0.03	-0.19	0.25	0.812	1647	-0.04	-0.24	0.16	0.697
Free cholesterol in very small VLDL (mmol/l)	2333	0.10	-0.08	0.29	0.283	2110	0.13	-0.09	0.35	0.244	1647	0.07	-0.13	0.27	0.476
Triglycerides in very small VLDL (mmol/l)	2333	0.12	-0.07	0.32	0.209	2110	0.19	-0.03	0.42	0.093	1647	0.10	-0.10	0.31	0.320
Concentration of IDL particles (mol/l)	2333	0.21	0.01	0.41	0.039	2110	0.26	0.03	0.50	0.028	1647	0.10	-0.08	0.28	0.284
Total lipids in IDL (mmol/l)	2333	0.19	-0.01	0.38	0.064	2110	0.23	0.00	0.46	0.051	1647	0.05	-0.12	0.23	0.543
Phospholipids in IDL (mmol/l)	2333	0.22	0.02	0.42	0.035	2110	0.26	0.02	0.49	0.032	1647	0.10	-0.08	0.29	0.284
Total cholesterol in IDL (mmol/l)	2333	0.15	-0.04	0.35	0.127	2110	0.19	-0.04	0.42	0.098	1647	0.02	-0.15	0.19	0.823
Cholesterol esters in IDL (mmol/l)	2333	0.13	-0.06	0.32	0.193	2110	0.17	-0.05	0.40	0.134	1647	0.00	-0.17	0.17	0.987
Free cholesterol in IDL (mmol/l)	2333	0.20	0.00	0.40	0.046	2110	0.23	0.00	0.47	0.049	1647	0.08	-0.11	0.26	0.413
Triglycerides in IDL (mmol/l)	2333	0.24	0.04	0.44	0.018	2110	0.29	0.06	0.53	0.015	1647	0.19	-0.02	0.40	0.070
Concentration of large LDL particles (mol/l)	2333	0.22	0.02	0.42	0.030	2110	0.27	0.03	0.51	0.025	1647	0.12	-0.07	0.32	0.205
Total lipids in large LDL (mmol/l)	2333	0.21	0.01	0.41	0.042	2110	0.25	0.02	0.49	0.035	1647	0.08	-0.10	0.26	0.369

S7 Table One-sample MR estimates of associations of puberty timing (per year later) with adiposity and cardiometabolic traits at age 18y among males and females in ALSPAC, using a full GRS of 351 SNPs for age at menarche

	Unadj.					*Adj. for measured BMI at age 8y*					*Adj. for measured outcome value at age 8y*				
**Standardised outcome at age 18y**	**N**	**Beta (2SLS)**	**LCL**	**UCL**	**P-value**	**N**	**Beta (2SLS)**	**LCL**	**UCL**	**P-value**	**N**	**Beta (2SLS)**	**LCL**	**UCL**	**P-value**
Phospholipids in large LDL (mmol/l)	2333	0.20	0.00	0.40	0.054	2110	0.24	0.01	0.48	0.043	1647	0.07	-0.11	0.25	0.422
Total cholesterol in large LDL (mmol/l)	2333	0.19	0.00	0.39	0.056	2110	0.24	0.00	0.47	0.047	1647	0.06	-0.11	0.24	0.481
Cholesterol esters in large LDL (mmol/l)	2333	0.19	-0.01	0.39	0.061	2110	0.23	0.00	0.47	0.048	1647	0.06	-0.11	0.24	0.496
Free cholesterol in large LDL (mmol/l)	2333	0.21	0.01	0.41	0.044	2110	0.24	0.00	0.47	0.047	1647	0.07	-0.11	0.25	0.432
Triglycerides in large LDL (mmol/l)	2333	0.27	0.06	0.47	0.011	2110	0.32	0.08	0.56	0.010	1647	0.23	0.01	0.44	0.042
Concentration of medium LDL particles (mol/l)	2333	0.22	0.02	0.42	0.030	2110	0.27	0.04	0.51	0.023	1647	0.14	-0.06	0.34	0.158
Total lipids in medium LDL (mmol/l)	2333	0.21	0.01	0.41	0.041	2110	0.25	0.02	0.49	0.033	1647	0.10	-0.09	0.29	0.296
Phospholipids in medium LDL (mmol/l)	2333	0.17	-0.02	0.37	0.085	2110	0.22	-0.01	0.45	0.062	1647	0.03	-0.14	0.21	0.696
Total cholesterol in medium LDL (mmol/l)	2333	0.20	0.00	0.40	0.051	2110	0.24	0.01	0.47	0.042	1647	0.09	-0.10	0.27	0.346
Cholesterol esters in medium LDL (mmol/l)	2333	0.20	0.00	0.40	0.047	2110	0.25	0.01	0.48	0.038	1647	0.10	-0.08	0.29	0.275
Free cholesterol in medium LDL (mmol/l)	2333	0.18	-0.02	0.38	0.071	2110	0.22	-0.01	0.45	0.067	1647	0.03	-0.15	0.20	0.775
Triglycerides in medium LDL (mmol/l)	2333	0.30	0.09	0.51	0.006	2110	0.35	0.10	0.60	0.006	1647	0.28	0.04	0.51	0.020
Concentration of small LDL particles (mol/l)	2333	0.22	0.02	0.42	0.034	2110	0.27	0.03	0.50	0.027	1647	0.13	-0.07	0.33	0.207
Total lipids in small LDL (mmol/l)	2333	0.20	0.01	0.40	0.044	2110	0.25	0.02	0.48	0.036	1647	0.10	-0.09	0.28	0.317
Phospholipids in small LDL (mmol/l)	2333	0.18	-0.02	0.38	0.075	2110	0.23	0.00	0.46	0.054	1647	0.05	-0.13	0.23	0.600
Total cholesterol in small LDL (mmol/l)	2333	0.20	0.00	0.39	0.052	2110	0.24	0.00	0.47	0.045	1647	0.09	-0.10	0.27	0.355
Cholesterol esters in small LDL (mmol/l)	2333	0.21	0.01	0.40	0.042	2110	0.25	0.02	0.48	0.036	1647	0.12	-0.07	0.31	0.232
Free cholesterol in small LDL (mmol/l)	2333	0.15	-0.04	0.34	0.131	2110	0.18	-0.05	0.40	0.128	1647	0.00	-0.18	0.17	0.967
Triglycerides in small LDL (mmol/l)	2333	0.24	0.04	0.44	0.020	2110	0.31	0.07	0.55	0.011	1647	0.23	0.00	0.45	0.048
Concentration of very large HDL particles (mol/l)	2333	0.23	0.02	0.44	0.032	2110	0.24	0.00	0.49	0.054	1647	0.15	-0.05	0.36	0.147
Total lipids in very large HDL (mmol/l)	2333	0.21	0.01	0.42	0.044	2110	0.23	-0.02	0.47	0.067	1647	0.14	-0.07	0.35	0.183
Phospholipids in very large HDL (mmol/l)	2333	0.24	0.03	0.45	0.027	2110	0.24	-0.01	0.48	0.055	1647	0.15	-0.05	0.36	0.134
Total cholesterol in very large HDL (mmol/l)	2333	0.16	-0.04	0.37	0.112	2110	0.18	-0.05	0.42	0.131	1647	0.11	-0.10	0.32	0.315
Cholesterol esters in very large HDL (mmol/l)	2333	0.15	-0.05	0.35	0.150	2110	0.17	-0.07	0.40	0.163	1647	0.10	-0.11	0.32	0.349
Free cholesterol in very large HDL (mmol/l)	2333	0.20	-0.01	0.40	0.059	2110	0.21	-0.03	0.45	0.087	1647	0.12	-0.08	0.32	0.252
Triglycerides in very large HDL (mmol/l)	2333	0.18	-0.02	0.38	0.073	2110	0.27	0.03	0.50	0.029	1647	0.17	-0.04	0.37	0.117
Concentration of large HDL particles (mol/l)	2333	0.23	0.02	0.44	0.029	2110	0.22	-0.03	0.46	0.080	1647	0.14	-0.07	0.35	0.178
Total lipids in large HDL (mmol/l)	2333	0.22	0.01	0.43	0.039	2110	0.20	-0.04	0.44	0.107	1647	0.12	-0.08	0.32	0.241
Phospholipids in large HDL (mmol/l)	2333	0.23	0.02	0.44	0.029	2110	0.22	-0.03	0.46	0.080	1647	0.14	-0.07	0.35	0.203
Total cholesterol in large HDL (mmol/l)	2333	0.20	0.00	0.40	0.055	2110	0.17	-0.06	0.41	0.153	1647	0.11	-0.09	0.30	0.297
Cholesterol esters in large HDL (mmol/l)	2333	0.20	0.00	0.40	0.055	2110	0.17	-0.06	0.41	0.152	1647	0.11	-0.09	0.31	0.286
Free cholesterol in large HDL (mmol/l)	2333	0.20	-0.01	0.40	0.057	2110	0.17	-0.07	0.40	0.161	1647	0.09	-0.10	0.29	0.345
Triglycerides in large HDL (mmol/l)	2333	0.20	0.00	0.41	0.055	2110	0.26	0.01	0.50	0.038	1647	0.17	-0.04	0.39	0.116
Concentration of medium HDL particles (mol/l)	2333	0.18	-0.03	0.38	0.091	2110	0.17	-0.06	0.41	0.153	1647	0.11	-0.11	0.33	0.336
Total lipids in medium HDL (mmol/l)	2333	0.17	-0.03	0.37	0.101	2110	0.16	-0.08	0.39	0.185	1647	0.10	-0.13	0.32	0.389
Phospholipids in medium HDL (mmol/l)	2333	0.20	-0.01	0.40	0.063	2110	0.19	-0.05	0.43	0.114	1647	0.13	-0.10	0.36	0.258
Total cholesterol in medium HDL (mmol/l)	2333	0.12	-0.07	0.32	0.216	2110	0.09	-0.14	0.32	0.440	1647	0.04	-0.18	0.26	0.703
Cholesterol esters in medium HDL (mmol/l)	2333	0.12	-0.08	0.32	0.231	2110	0.08	-0.14	0.31	0.474	1647	0.04	-0.18	0.26	0.710
Free cholesterol in medium HDL (mmol/l)	2333	0.14	-0.06	0.34	0.180	2110	0.12	-0.11	0.35	0.324	1647	0.05	-0.18	0.27	0.689
Triglycerides in medium HDL (mmol/l)	2333	0.10	-0.09	0.30	0.301	2110	0.19	-0.04	0.42	0.103	1647	0.12	-0.09	0.32	0.261
Concentration of small HDL particles (mol/l)	2333	0.08	-0.11	0.28	0.393	2110	0.09	-0.13	0.31	0.434	1647	0.05	-0.17	0.26	0.672
Total lipids in small HDL (mmol/l)	2333	0.15	-0.05	0.35	0.138	2110	0.15	-0.08	0.38	0.204	1647	0.10	-0.12	0.32	0.374
Phospholipids in small HDL (mmol/l)	2333	0.02	-0.17	0.21	0.802	2110	0.02	-0.20	0.24	0.883	1647	-0.02	-0.23	0.19	0.864
Total cholesterol in small HDL (mmol/l)	2333	0.25	0.03	0.46	0.023	2110	0.24	-0.01	0.49	0.057	1647	0.19	-0.04	0.42	0.109
Cholesterol esters in small HDL (mmol/l)	2333	0.27	0.05	0.48	0.015	2110	0.26	0.01	0.51	0.040	1647	0.21	-0.02	0.45	0.075
Free cholesterol in small HDL (mmol/l)	2333	0.10	-0.09	0.29	0.308	2110	0.08	-0.14	0.31	0.473	1647	0.04	-0.18	0.26	0.718
Triglycerides in small HDL (mmol/l)	2333	0.10	-0.09	0.30	0.309	2110	0.18	-0.05	0.41	0.117	1647	0.12	-0.09	0.33	0.268
Phospholipids to total lipds ratio in chylomicrons and extremely large VLDL (%)	2333	-0.09	-0.28	0.10	0.364	2110	-0.11	-0.34	0.12	0.362	1647	-0.12	-0.38	0.13	0.351
Total cholesterol to total lipids ratio in chylomicrons and extremely large VLDL (%)	2333	0.16	-0.03	0.34	0.106	2110	0.24	0.02	0.47	0.035	1647	0.17	-0.04	0.39	0.109
Cholesterol esters to total lipids ratio in chylomicrons and extremely large VLDL (%)	2333	0.14	-0.04	0.33	0.126	2110	0.22	0.00	0.44	0.051	1647	0.14	-0.06	0.35	0.173
Free cholesterol to total lipids ratio in chylomicrons and extremely large VLDL (%)	2333	0.07	-0.11	0.26	0.436	2110	0.14	-0.08	0.35	0.212	1647	0.13	-0.08	0.35	0.221
Triglycerides to total lipids ratio in chylomicrons and extremely large VLDL (%)	2333	-0.15	-0.35	0.04	0.128	2110	-0.24	-0.47	0.00	0.055	1647	-0.17	-0.40	0.06	0.154
Phospholipids to total lipds ratio in very large VLDL (%)	2333	0.02	-0.16	0.21	0.800	2110	0.08	-0.14	0.30	0.474	1647	0.06	-0.15	0.28	0.567
Total cholesterol to total lipids ratio in very large VLDL (%)	2333	0.07	-0.08	0.22	0.345	2110	0.07	-0.11	0.24	0.451	1647	0.02	-0.16	0.20	0.803
Cholesterol esters to total lipids ratio in very large VLDL (%)	2333	0.05	-0.07	0.17	0.394	2110	0.05	-0.09	0.19	0.490	1647	0.01	-0.13	0.15	0.924
Free cholesterol to total lipids ratio in very large VLDL (%)	2333	0.07	-0.06	0.21	0.289	2110	0.07	-0.09	0.23	0.381	1647	0.06	-0.09	0.21	0.433

S7 Table One-sample MR estimates of associations of puberty timing (per year later) with adiposity and cardiometabolic traits at age 18y among males and females in ALSPAC, using a full GRS of 351 SNPs for age at menarche

	Unadj.					*Adj. for measured BMI at age 8y*					*Adj. for measured outcome value at age 8y*				
**Standardised outcome at age 18y**	**N**	**Beta (2SLS)**	**LCL**	**UCL**	**P-value**	**N**	**Beta (2SLS)**	**LCL**	**UCL**	**P-value**	**N**	**Beta (2SLS)**	**LCL**	**UCL**	**P-value**
Triglycerides to total lipids ratio in very large VLDL (%)	2333	-0.10	-0.28	0.08	0.289	2110	-0.11	-0.32	0.10	0.308	1647	-0.05	-0.26	0.16	0.618
Phospholipids to total lipds ratio in large VLDL (%)	2333	0.05	-0.12	0.23	0.549	2110	0.13	-0.08	0.33	0.215	1647	0.13	-0.07	0.34	0.201
Total cholesterol to total lipids ratio in large VLDL (%)	2333	0.12	-0.07	0.30	0.228	2110	0.21	-0.01	0.43	0.064	1647	0.20	-0.01	0.42	0.063
Cholesterol esters to total lipids ratio in large VLDL (%)	2333	0.15	-0.03	0.33	0.109	2110	0.21	-0.01	0.43	0.056	1647	0.22	0.00	0.44	0.053
Free cholesterol to total lipids ratio in large VLDL (%)	2333	0.01	-0.07	0.08	0.850	2110	0.04	-0.04	0.13	0.328	1647	0.04	-0.04	0.13	0.314
Triglycerides to total lipids ratio in large VLDL (%)	2333	-0.09	-0.27	0.09	0.342	2110	-0.18	-0.38	0.03	0.101	1647	-0.18	-0.39	0.03	0.091
Phospholipids to total lipds ratio in medium VLDL (%)	2333	0.19	-0.01	0.38	0.062	2110	0.16	-0.07	0.39	0.165	1647	0.09	-0.11	0.30	0.367
Total cholesterol to total lipids ratio in medium VLDL (%)	2333	0.22	0.01	0.42	0.035	2110	0.30	0.05	0.54	0.016	1647	0.22	0.01	0.43	0.044
Cholesterol esters to total lipids ratio in medium VLDL (%)	2333	0.21	0.01	0.41	0.042	2110	0.28	0.04	0.52	0.024	1647	0.22	0.00	0.43	0.052
Free cholesterol to total lipids ratio in medium VLDL (%)	2333	0.14	-0.05	0.33	0.137	2110	0.22	0.00	0.44	0.055	1647	0.17	-0.04	0.38	0.120
Triglycerides to total lipids ratio in medium VLDL (%)	2333	-0.23	-0.43	-0.03	0.024	2110	-0.30	-0.54	-0.06	0.015	1647	-0.20	-0.41	0.00	0.055
Phospholipids to total lipds ratio in small VLDL (%)	2333	0.06	-0.13	0.25	0.531	2110	0.00	-0.22	0.21	0.979	1647	-0.02	-0.23	0.18	0.835
Total cholesterol to total lipids ratio in small VLDL (%)	2333	0.08	-0.11	0.28	0.407	2110	0.08	-0.15	0.30	0.515	1647	0.05	-0.17	0.26	0.671
Cholesterol esters to total lipids ratio in small VLDL (%)	2333	0.05	-0.14	0.25	0.611	2110	0.04	-0.18	0.27	0.701	1647	0.03	-0.19	0.25	0.789
Free cholesterol to total lipids ratio in small VLDL (%)	2333	0.23	0.03	0.43	0.024	2110	0.22	-0.01	0.45	0.065	1647	0.11	-0.10	0.33	0.297
Triglycerides to total lipids ratio in small VLDL (%)	2333	-0.11	-0.30	0.09	0.294	2110	-0.08	-0.30	0.15	0.508	1647	-0.06	-0.28	0.17	0.614
Phospholipids to total lipds ratio in very small VLDL (%)	2333	0.27	0.06	0.48	0.011	2110	0.30	0.06	0.55	0.016	1647	0.16	-0.04	0.37	0.119
Total cholesterol to total lipids ratio in very small VLDL (%)	2333	-0.20	-0.40	0.00	0.051	2110	-0.24	-0.48	0.00	0.047	1647	-0.16	-0.39	0.06	0.160
Cholesterol esters to total lipids ratio in very small VLDL (%)	2333	-0.22	-0.43	-0.02	0.033	2110	-0.24	-0.48	-0.01	0.045	1647	-0.17	-0.40	0.05	0.131
Free cholesterol to total lipids ratio in very small VLDL (%)	2333	0.01	-0.17	0.18	0.942	2110	-0.06	-0.27	0.14	0.545	1647	-0.01	-0.22	0.20	0.910
Triglycerides to total lipids ratio in very small VLDL (%)	2333	0.06	-0.14	0.25	0.563	2110	0.08	-0.14	0.31	0.476	1647	0.04	-0.18	0.27	0.693
Phospholipids to total lipds ratio in IDL (%)	2333	0.11	-0.08	0.30	0.275	2110	0.06	-0.16	0.28	0.608	1647	0.08	-0.15	0.30	0.501
Total cholesterol to total lipids ratio in IDL (%)	2333	-0.11	-0.31	0.09	0.278	2110	-0.09	-0.32	0.14	0.445	1647	-0.09	-0.31	0.13	0.404
Cholesterol esters to total lipids ratio in IDL (%)	2333	-0.18	-0.37	0.02	0.082	2110	-0.14	-0.37	0.08	0.215	1647	-0.14	-0.37	0.08	0.213
Free cholesterol to total lipids ratio in IDL (%)	2333	0.15	-0.05	0.35	0.149	2110	0.12	-0.11	0.36	0.305	1647	0.10	-0.13	0.32	0.404
Triglycerides to total lipids ratio in IDL (%)	2333	0.09	-0.11	0.28	0.392	2110	0.08	-0.15	0.31	0.478	1647	0.09	-0.13	0.30	0.433
Phospholipids to total lipds ratio in large LDL (%)	2333	-0.22	-0.42	-0.03	0.026	2110	-0.28	-0.52	-0.05	0.018	1647	-0.08	-0.26	0.11	0.421
Total cholesterol to total lipids ratio in large LDL (%)	2333	0.09	-0.10	0.28	0.363	2110	0.13	-0.10	0.36	0.261	1647	-0.02	-0.20	0.16	0.842
Cholesterol esters to total lipids ratio in large LDL (%)	2333	0.14	-0.06	0.33	0.164	2110	0.20	-0.03	0.44	0.085	1647	0.00	-0.16	0.16	0.999
Free cholesterol to total lipids ratio in large LDL (%)	2333	-0.19	-0.39	0.02	0.070	2110	-0.28	-0.52	-0.04	0.022	1647	-0.17	-0.38	0.05	0.128
Triglycerides to total lipids ratio in large LDL (%)	2333	0.11	-0.09	0.31	0.284	2110	0.11	-0.13	0.34	0.364	1647	0.11	-0.10	0.32	0.315
Phospholipids to total lipds ratio in medium LDL (%)	2333	-0.24	-0.44	-0.03	0.023	2110	-0.30	-0.54	-0.05	0.018	1647	-0.17	-0.38	0.04	0.107
Total cholesterol to total lipids ratio in medium LDL (%)	2333	0.12	-0.07	0.31	0.218	2110	0.17	-0.06	0.39	0.152	1647	0.05	-0.14	0.24	0.598
Cholesterol esters to total lipids ratio in medium LDL (%)	2333	0.18	-0.01	0.38	0.070	2110	0.24	0.01	0.48	0.042	1647	0.10	-0.09	0.29	0.296
Free cholesterol to total lipids ratio in medium LDL (%)	2333	-0.24	-0.44	-0.04	0.021	2110	-0.31	-0.55	-0.07	0.011	1647	-0.18	-0.38	0.03	0.092
Triglycerides to total lipids ratio in medium LDL (%)	2333	0.20	0.02	0.39	0.032	2110	0.22	0.00	0.44	0.050	1647	0.19	-0.02	0.39	0.076
Phospholipids to total lipds ratio in small LDL (%)	2333	-0.21	-0.41	-0.02	0.035	2110	-0.27	-0.50	-0.03	0.026	1647	-0.13	-0.33	0.06	0.178
Total cholesterol to total lipids ratio in small LDL (%)	2333	0.13	-0.06	0.33	0.171	2110	0.17	-0.06	0.40	0.151	1647	0.06	-0.13	0.25	0.558
Cholesterol esters to total lipids ratio in small LDL (%)	2333	0.20	0.00	0.40	0.049	2110	0.25	0.02	0.49	0.036	1647	0.13	-0.07	0.32	0.203
Free cholesterol to total lipids ratio in small LDL (%)	2333	-0.27	-0.48	-0.06	0.011	2110	-0.35	-0.59	-0.10	0.006	1647	-0.24	-0.46	-0.02	0.036
Triglycerides to total lipids ratio in small LDL (%)	2333	0.18	-0.02	0.38	0.083	2110	0.23	-0.01	0.47	0.060	1647	0.17	-0.05	0.40	0.131
Phospholipids to total lipds ratio in very large HDL (%)	2333	0.26	0.05	0.47	0.015	2110	0.24	-0.01	0.48	0.056	1647	0.21	-0.01	0.43	0.057
Total cholesterol to total lipids ratio in very large HDL (%)	2333	-0.27	-0.49	-0.06	0.012	2110	-0.26	-0.51	-0.02	0.037	1647	-0.22	-0.45	0.00	0.047
Cholesterol esters to total lipids ratio in very large HDL (%)	2333	-0.26	-0.48	-0.05	0.015	2110	-0.25	-0.50	-0.01	0.043	1647	-0.21	-0.42	0.01	0.063
Free cholesterol to total lipids ratio in very large HDL (%)	2333	-0.01	-0.21	0.19	0.913	2110	-0.02	-0.25	0.21	0.875	1647	-0.10	-0.33	0.13	0.377
Triglycerides to total lipids ratio in very large HDL (%)	2333	-0.01	-0.20	0.17	0.876	2110	0.07	-0.15	0.28	0.539	1647	-0.01	-0.21	0.20	0.961
Phospholipids to total lipds ratio in large HDL (%)	2333	0.01	-0.17	0.19	0.909	2110	0.08	-0.13	0.29	0.463	1647	0.02	-0.17	0.22	0.811
Total cholesterol to total lipids ratio in large HDL (%)	2333	0.05	-0.13	0.22	0.621	2110	-0.03	-0.24	0.17	0.756	1647	0.02	-0.17	0.20	0.856
Cholesterol esters to total lipids ratio in large HDL (%)	2333	0.05	-0.13	0.23	0.610	2110	-0.03	-0.24	0.18	0.804	1647	0.03	-0.15	0.22	0.722
Free cholesterol to total lipids ratio in large HDL (%)	2333	0.02	-0.15	0.20	0.799	2110	-0.06	-0.26	0.15	0.600	1647	-0.05	-0.23	0.14	0.631
Triglycerides to total lipids ratio in large HDL (%)	2333	-0.14	-0.33	0.04	0.134	2110	-0.06	-0.27	0.15	0.555	1647	-0.09	-0.29	0.11	0.371
Phospholipids to total lipds ratio in medium HDL (%)	2333	0.17	-0.03	0.37	0.089	2110	0.20	-0.03	0.44	0.090	1647	0.17	-0.06	0.40	0.152
Total cholesterol to total lipids ratio in medium HDL (%)	2333	-0.13	-0.32	0.06	0.179	2110	-0.21	-0.44	0.02	0.079	1647	-0.15	-0.37	0.07	0.178
Cholesterol esters to total lipids ratio in medium HDL (%)	2333	-0.12	-0.32	0.07	0.221	2110	-0.20	-0.43	0.04	0.104	1647	-0.12	-0.35	0.10	0.275
Free cholesterol to total lipids ratio in medium HDL (%)	2333	-0.10	-0.35	0.14	0.410	2110	-0.14	-0.44	0.15	0.335	1647	-0.19	-0.51	0.13	0.241
Triglycerides to total lipids ratio in medium HDL (%)	2333	-0.02	-0.20	0.17	0.863	2110	0.08	-0.13	0.30	0.447	1647	0.05	-0.15	0.24	0.625

S7 Table One-sample MR estimates of associations of puberty timing (per year later) with adiposity and cardiometabolic traits at age 18y among males and females in ALSPAC, using a full GRS of 351 SNPs for age at menarche

	Unadj.					*Adj. for measured BMI at age 8y*					*Adj. for measured outcome value at age 8y*				
**Standardised outcome at age 18y**	**N**	**Beta (2SLS)**	**LCL**	**UCL**	**P-value**	**N**	**Beta (2SLS)**	**LCL**	**UCL**	**P-value**	**N**	**Beta (2SLS)**	**LCL**	**UCL**	**P-value**
Phospholipids to total lipids ratio in small HDL (%)	2333	-0.27	-0.48	-0.06	0.012	2110	-0.28	-0.53	-0.03	0.026	1647	-0.23	-0.46	-0.01	0.044
Total cholesterol to total lipids ratio in small HDL (%)	2333	0.26	0.05	0.47	0.015	2110	0.25	0.00	0.49	0.047	1647	0.22	-0.01	0.45	0.059
Cholesterol esters to total lipids ratio in small HDL (%)	2333	0.26	0.05	0.47	0.017	2110	0.25	0.01	0.49	0.045	1647	0.22	-0.01	0.45	0.058
Free cholesterol to total lipids ratio in small HDL (%)	2333	-0.09	-0.28	0.10	0.363	2110	-0.14	-0.36	0.09	0.239	1647	-0.12	-0.33	0.10	0.279
Triglycerides to total lipids ratio in small HDL (%)	2333	0.01	-0.18	0.19	0.948	2110	0.10	-0.12	0.31	0.379	1647	0.06	-0.15	0.27	0.574
Mean diameter for VLDL particles (nm)	2333	-0.11	-0.30	0.08	0.256	2110	-0.03	-0.25	0.19	0.788	1647	-0.03	-0.25	0.18	0.748
Mean diameter for LDL particles (nm)	2333	-0.09	-0.29	0.10	0.340	2110	-0.09	-0.32	0.13	0.416	1647	-0.02	-0.24	0.20	0.876
Mean diameter for HDL particles (nm)	2333	0.23	0.02	0.44	0.032	2110	0.22	-0.02	0.46	0.076	1647	0.14	-0.05	0.34	0.153
Serum total cholesterol (mmol/l)	2333	0.21	0.01	0.41	0.041	2110	0.26	0.02	0.50	0.034	1647	0.07	-0.11	0.25	0.429
Total cholesterol in VLDL (mmol/l)	2333	0.00	-0.18	0.19	0.972	2110	0.09	-0.13	0.31	0.410	1647	0.00	-0.19	0.18	0.960
Remnant cholesterol (non-HDL, non-LDL -cholesterol) (mmol/l)	2333	0.07	-0.11	0.26	0.435	2110	0.15	-0.07	0.37	0.189	1647	0.00	-0.17	0.18	0.983
Total cholesterol in LDL (mmol/l)	2333	0.20	0.00	0.39	0.053	2110	0.24	0.01	0.47	0.045	1647	0.08	-0.10	0.26	0.412
Total cholesterol in HDL (mmol/l)	2333	0.23	0.02	0.44	0.035	2110	0.21	-0.03	0.45	0.092	1647	0.12	-0.09	0.33	0.246
Total cholesterol in HDL2 (mmol/l)	2333	0.21	0.00	0.42	0.046	2110	0.18	-0.06	0.42	0.140	1647	0.11	-0.10	0.32	0.291
Total cholesterol in HDL3 (mmol/l)	2333	0.24	0.03	0.45	0.026	2110	0.25	0.00	0.50	0.050	1647	0.14	-0.07	0.35	0.193
Esterified cholesterol (mmol/l)	2323	0.19	-0.01	0.39	0.065	2102	0.23	-0.01	0.46	0.057	1640	0.06	-0.12	0.25	0.513
Free cholesterol (mmol/l)	2322	0.22	0.02	0.42	0.030	2101	0.27	0.04	0.50	0.024	1638	0.08	-0.09	0.25	0.356
Serum total triglycerides (mmol/l)	2333	0.01	-0.18	0.21	0.896	2110	0.11	-0.12	0.33	0.354	1647	0.02	-0.19	0.24	0.829
Triglycerides in VLDL (mmol/l)	2333	-0.06	-0.26	0.13	0.521	2110	0.03	-0.20	0.25	0.824	1647	-0.04	-0.26	0.18	0.707
Triglycerides in LDL (mmol/l)	2333	0.27	0.07	0.48	0.009	2110	0.33	0.09	0.57	0.008	1647	0.24	0.02	0.47	0.032
Triglycerides in HDL (mmol/l)	2333	0.16	-0.04	0.36	0.125	2110	0.25	0.01	0.49	0.037	1647	0.16	-0.05	0.37	0.132
Diacylglycerol (mmol/l)	2270	-0.04	-0.23	0.15	0.663	2058	0.01	-0.21	0.23	0.949	1574	-0.07	-0.30	0.16	0.533
Ratio of diacylglycerol to triglycerides	2271	-0.03	-0.20	0.15	0.753	2059	0.00	-0.21	0.20	0.967	1575	-0.07	-0.28	0.14	0.503
Total phosphoglycerides (mmol/l)	2322	0.23	0.03	0.44	0.026	2101	0.28	0.04	0.52	0.022	1638	0.18	-0.03	0.40	0.090
Ratio of triglycerides to phosphoglycerides	2322	-0.09	-0.28	0.11	0.382	2101	0.00	-0.22	0.22	0.991	1638	-0.05	-0.27	0.18	0.682
Phosphatidylcholine and other cholines (mmol/l)	2305	0.24	0.03	0.44	0.022	2085	0.28	0.04	0.53	0.021	1620	0.16	-0.06	0.37	0.152
Total cholines (mmol/l)	2323	0.23	0.03	0.44	0.026	2102	0.28	0.04	0.52	0.024	1640	0.16	-0.05	0.37	0.142
Apolipoprotein A-I (g/l)	2333	0.24	0.03	0.45	0.026	2110	0.25	0.00	0.50	0.048	1647	0.13	-0.08	0.33	0.229
Apolipoprotein B (g/l)	2333	0.09	-0.10	0.28	0.370	2110	0.17	-0.06	0.39	0.140	1647	0.04	-0.14	0.22	0.667
Ratio of apolipoprotein B to apolipoprotein A-I	2333	-0.03	-0.22	0.16	0.741	2110	0.05	-0.17	0.27	0.654	1647	-0.02	-0.21	0.17	0.826
Total fatty acids (mmol/l)	2323	0.14	-0.06	0.33	0.169	2102	0.21	-0.02	0.44	0.071	1640	0.09	-0.11	0.29	0.366
Estimated description of fatty acid chain length, not actual carbon number	2323	0.00	-0.19	0.19	0.995	2102	0.00	-0.21	0.22	0.976	1639	0.04	-0.18	0.26	0.753
Estimated degree of unsaturation	2323	0.04	-0.13	0.22	0.626	2102	0.02	-0.19	0.23	0.853	1640	0.02	-0.20	0.24	0.848
22:6, docosahexaenoic acid (mmol/l)	2323	0.08	-0.11	0.26	0.416	2102	0.09	-0.12	0.31	0.407	1640	0.07	-0.13	0.28	0.498
18:2, linoleic acid (mmol/l)	2322	0.23	0.02	0.43	0.028	2101	0.28	0.04	0.53	0.023	1639	0.16	-0.04	0.36	0.114
Conjugated linoleic acid (mmol/l)	2322	0.10	-0.07	0.26	0.251	2101	0.15	-0.04	0.34	0.127	1637	0.07	-0.12	0.27	0.460
Omega-3 fatty acids (mmol/l)	2323	0.06	-0.13	0.25	0.509	2102	0.11	-0.10	0.33	0.305	1640	0.04	-0.17	0.24	0.720
Omega-6 fatty acids (mmol/l)	2323	0.20	0.00	0.40	0.050	2102	0.26	0.02	0.49	0.034	1640	0.13	-0.07	0.32	0.195
Polyunsaturated fatty acids (mmol/l)	2322	0.19	-0.01	0.39	0.063	2101	0.25	0.01	0.48	0.040	1639	0.12	-0.08	0.31	0.234
Monounsaturated fatty acids; 16:1, 18:1 (mmol/l)	2323	0.10	-0.10	0.29	0.327	2102	0.16	-0.06	0.39	0.156	1639	0.07	-0.14	0.27	0.507
Saturated fatty acids (mmol/l)	2322	0.10	-0.09	0.29	0.317	2101	0.18	-0.05	0.41	0.118	1638	0.07	-0.13	0.28	0.490
Ratio of 22:6 docosahexaenoic acid to total fatty acids (%)	2324	-0.01	-0.20	0.17	0.894	2103	-0.04	-0.25	0.17	0.718	1641	0.01	-0.20	0.22	0.941
Ratio of 18:2 linoleic acid to total fatty acids (%)	2323	0.19	-0.01	0.39	0.060	2102	0.15	-0.07	0.38	0.189	1640	0.17	-0.06	0.40	0.144
Ratio of conjugated linoleic acid to total fatty acids (%)	2323	0.08	-0.09	0.24	0.379	2102	0.11	-0.08	0.31	0.256	1638	0.06	-0.14	0.25	0.569
Ratio of omega-3 fatty acids to total fatty acids (%)	2324	-0.05	-0.24	0.13	0.575	2103	-0.06	-0.28	0.16	0.593	1641	-0.03	-0.25	0.19	0.779
Ratio of omega-6 fatty acids to total fatty acids (%)	2324	0.13	-0.07	0.33	0.191	2103	0.09	-0.14	0.31	0.449	1641	0.12	-0.11	0.34	0.309
Ratio of polyunsaturated fatty acids to total fatty acids (%)	2323	0.11	-0.08	0.31	0.267	2102	0.07	-0.16	0.29	0.553	1640	0.10	-0.12	0.33	0.375
Ratio of monounsaturated fatty acids to total fatty acids (%)	2324	-0.01	-0.20	0.17	0.887	2103	0.00	-0.22	0.21	0.990	1640	-0.01	-0.23	0.21	0.931
Ratio of saturated fatty acids to total fatty acids (%)	2323	-0.11	-0.30	0.07	0.232	2102	-0.08	-0.29	0.14	0.476	1639	-0.11	-0.31	0.10	0.314
Glucose (mmol/l)	2332	-0.13	-0.35	0.08	0.222	2109	-0.11	-0.37	0.15	0.406	1636	-0.04	-0.20	0.11	0.610
Lactate (mmol/l)	2332	0.09	-0.10	0.29	0.332	2109	0.16	-0.07	0.39	0.168	1647	0.10	-0.12	0.33	0.370
Pyruvate (mmol/l)	2332	0.06	-0.12	0.24	0.534	2109	0.14	-0.07	0.36	0.185	1644	0.09	-0.13	0.30	0.419
Citrate (mmol/l)	2332	0.40	0.19	0.61	1.55E-04	2109	0.33	0.10	0.56	0.004	1644	0.33	0.10	0.56	0.004
Alanine (mmol/l)	2332	0.19	0.00	0.39	0.050	2109	0.24	0.02	0.47	0.032	1647	0.17	-0.05	0.39	0.131
Glutamine (mmol/l)	2332	-0.04	-0.23	0.15	0.667	2109	-0.06	-0.28	0.15	0.573	1644	-0.09	-0.31	0.13	0.400
Histidine (mmol/l)	2332	0.01	-0.19	0.20	0.938	2109	0.04	-0.18	0.26	0.720	1645	0.00	-0.22	0.21	0.996

S7 Table One-sample MR estimates of associations of puberty timing (per year later) with adiposity and cardiometabolic traits at age 18y among males and females in ALSPAC, using a full GRS of 351 SNPs for age at menarche

	Unadj.					*Adj. for measured BMI at age 8y*					*Adj. for measured outcome value at age 8y*				
**Standardised outcome at age 18y**	**N**	**Beta (2SLS)**	**LCL**	**UCL**	**P-value**	**N**	**Beta (2SLS)**	**LCL**	**UCL**	**P-value**	**N**	**Beta (2SLS)**	**LCL**	**UCL**	**P-value**
Isoleucine (mmol/l)	2332	-0.09	-0.28	0.10	0.344	2109	-0.01	-0.23	0.20	0.896	1647	-0.09	-0.30	0.13	0.436
Leucine (mmol/l)	2332	-0.17	-0.37	0.03	0.099	2109	-0.16	-0.39	0.08	0.192	1647	-0.18	-0.42	0.06	0.135
Valine (mmol/l)	2332	-0.20	-0.40	0.00	0.053	2109	-0.17	-0.40	0.06	0.148	1647	-0.24	-0.48	0.00	0.047
Phenylalanine (mmol/l)	2331	-0.08	-0.28	0.11	0.396	2108	-0.10	-0.33	0.12	0.370	1642	-0.16	-0.40	0.08	0.193
Tyrosine (mmol/l)	2332	-0.11	-0.30	0.09	0.293	2109	-0.01	-0.23	0.22	0.949	1640	-0.16	-0.40	0.07	0.164
Acetate (mmol/l)	2330	-0.02	-0.19	0.16	0.863	2107	-0.01	-0.22	0.19	0.890	1647	-0.01	-0.21	0.19	0.898
Acetoacetate (mmol/l)	2332	-0.02	-0.20	0.15	0.791	2109	-0.08	-0.29	0.13	0.463	1646	-0.02	-0.23	0.19	0.844
3-hydroxybutyrate (mmol/l)	2330	0.05	-0.12	0.23	0.567	2107	-0.01	-0.21	0.18	0.902	1644	0.03	-0.18	0.25	0.758
Creatinine (mmol/l)	2332	-0.32	-0.54	-0.11	0.003	2109	-0.37	-0.61	-0.12	0.004	1643	-0.33	-0.58	-0.09	0.007
Albumin (signal area)	2333	-0.21	-0.41	-0.01	0.036	2110	-0.31	-0.54	-0.07	0.011	1645	-0.30	-0.54	-0.06	0.013
Glycoprotein acetyls, mainly a1-acid glycoprotein (mmol/l)	2332	0.05	-0.14	0.25	0.576	2109	0.15	-0.08	0.37	0.197	1647	0.10	-0.11	0.32	0.348

Complete case sample

	Unadj.					*Adj. for measured BMI at age 8y*					*Adj. for measured outcome value at age 8y*				
**Standardised outcome at age 18y**	**N**	**Beta (2SLS)**	**LCL**	**UCL**	**P-value**	**N**	**Beta (2SLS)**	**LCL**	**UCL**	**P-value**	**N**	**Beta (2SLS)**	**LCL**	**UCL**	**P-value**
Body mass index (kg/m^2)	1193	-0.28	-0.49	-0.08	0.008	1193	-0.12	-0.29	0.05	0.167	1193	-0.12	-0.29	0.05	0.167
Fat mass index (kg/m^2)	1193	-0.08	-0.29	0.12	0.430	1193	0.07	-0.12	0.27	0.458	1193	0.11	-0.06	0.28	0.205
Lean mass index (kg/m^2)	1193	-0.39	-0.65	-0.13	0.003	1193	-0.36	-0.64	-0.09	0.010	1193	-0.08	-0.24	0.08	0.315
Systolic blood pressure (mmHg)	1193	-0.27	-0.50	-0.03	0.027	1193	-0.25	-0.50	0.00	0.051	1193	-0.22	-0.45	0.01	0.060
Diastolic blood pressure (mmHg)	1193	0.02	-0.18	0.23	0.827	1193	0.08	-0.14	0.30	0.480	1193	0.06	-0.15	0.26	0.591
Concentration of chylomicrons and extremely large VLDL particles (mol/l)	1193	0.05	-0.14	0.25	0.589	1193	0.11	-0.10	0.32	0.321	1193	0.06	-0.13	0.24	0.539
Total lipids in chylomicrons and extremely large VLDL (mmol/l)	1193	0.06	-0.13	0.25	0.538	1193	0.11	-0.10	0.32	0.291	1193	0.07	-0.12	0.25	0.489
Phospholipids in chylomicrons and extremely large VLDL (mmol/l)	1193	0.07	-0.12	0.26	0.494	1193	0.12	-0.09	0.33	0.261	1193	0.07	-0.11	0.26	0.452
Total cholesterol in chylomicrons and extremely large VLDL (mmol/l)	1193	0.06	-0.14	0.26	0.557	1193	0.11	-0.10	0.33	0.314	1193	0.07	-0.12	0.26	0.496
Cholesterol esters in chylomicrons and extremely large VLDL (mmol/l)	1193	0.05	-0.15	0.25	0.628	1193	0.10	-0.12	0.32	0.377	1193	0.06	-0.13	0.25	0.549
Free cholesterol in chylomicrons and extremely large VLDL (mmol/l)	1193	0.07	-0.12	0.26	0.487	1193	0.12	-0.09	0.33	0.257	1193	0.07	-0.11	0.26	0.445
Triglycerides in chylomicrons and extremely large VLDL (mmol/l)	1193	0.06	-0.13	0.25	0.549	1193	0.11	-0.10	0.32	0.297	1193	0.06	-0.12	0.25	0.501
Concentration of very large VLDL particles (mol/l)	1193	0.04	-0.15	0.23	0.697	1193	0.09	-0.12	0.29	0.412	1193	0.04	-0.14	0.23	0.638
Total lipids in very large VLDL (mmol/l)	1193	0.04	-0.15	0.23	0.681	1193	0.09	-0.12	0.29	0.404	1193	0.05	-0.14	0.23	0.620
Phospholipids in very large VLDL (mmol/l)	1193	0.05	-0.14	0.24	0.588	1193	0.10	-0.10	0.31	0.332	1193	0.06	-0.13	0.24	0.541
Total cholesterol in very large VLDL (mmol/l)	1193	0.04	-0.15	0.24	0.680	1193	0.09	-0.12	0.30	0.395	1193	0.05	-0.14	0.23	0.619
Cholesterol esters in very large VLDL (mmol/l)	1193	0.03	-0.17	0.22	0.797	1193	0.08	-0.14	0.29	0.489	1193	0.03	-0.15	0.22	0.724
Free cholesterol in very large VLDL (mmol/l)	1193	0.06	-0.14	0.25	0.554	1193	0.11	-0.10	0.32	0.303	1193	0.06	-0.12	0.25	0.507
Triglycerides in very large VLDL (mmol/l)	1193	0.04	-0.15	0.23	0.709	1193	0.08	-0.12	0.29	0.432	1193	0.04	-0.14	0.23	0.645
Concentration of large VLDL particles (mol/l)	1193	0.02	-0.17	0.22	0.804	1193	0.07	-0.14	0.28	0.519	1193	0.03	-0.15	0.22	0.718
Total lipids in large VLDL (mmol/l)	1193	0.03	-0.17	0.22	0.800	1193	0.07	-0.14	0.28	0.514	1193	0.03	-0.15	0.22	0.715
Phospholipids in large VLDL (mmol/l)	1193	0.03	-0.16	0.23	0.737	1193	0.08	-0.13	0.29	0.460	1193	0.04	-0.14	0.23	0.661
Total cholesterol in large VLDL (mmol/l)	1193	0.03	-0.16	0.23	0.743	1193	0.08	-0.13	0.29	0.461	1193	0.04	-0.14	0.23	0.671
Cholesterol esters in large VLDL (mmol/l)	1193	0.03	-0.17	0.23	0.789	1193	0.07	-0.14	0.29	0.498	1193	0.04	-0.15	0.22	0.709
Free cholesterol in large VLDL (mmol/l)	1193	0.04	-0.15	0.23	0.700	1193	0.08	-0.12	0.29	0.428	1193	0.04	-0.14	0.23	0.636
Triglycerides in large VLDL (mmol/l)	1193	0.02	-0.17	0.21	0.841	1193	0.06	-0.15	0.27	0.555	1193	0.03	-0.16	0.22	0.750
Concentration of medium VLDL particles (mol/l)	1193	0.00	-0.20	0.20	0.996	1193	0.04	-0.17	0.25	0.700	1193	0.01	-0.17	0.20	0.893
Total lipids in medium VLDL (mmol/l)	1193	0.00	-0.20	0.20	0.983	1193	0.04	-0.17	0.26	0.678	1193	0.01	-0.17	0.20	0.876
Phospholipids in medium VLDL (mmol/l)	1193	0.02	-0.18	0.22	0.855	1193	0.06	-0.15	0.27	0.565	1193	0.03	-0.16	0.21	0.759
Total cholesterol in medium VLDL (mmol/l)	1193	0.03	-0.17	0.23	0.759	1193	0.08	-0.14	0.29	0.483	1193	0.03	-0.15	0.22	0.711
Cholesterol esters in medium VLDL (mmol/l)	1193	0.03	-0.18	0.24	0.785	1193	0.07	-0.15	0.30	0.512	1193	0.02	-0.16	0.21	0.805
Free cholesterol in medium VLDL (mmol/l)	1193	0.03	-0.16	0.23	0.735	1193	0.08	-0.13	0.29	0.468	1193	0.04	-0.14	0.23	0.643
Triglycerides in medium VLDL (mmol/l)	1193	-0.02	-0.22	0.18	0.857	1193	0.02	-0.19	0.23	0.843	1193	0.00	-0.19	0.18	0.984
Concentration of small VLDL particles (mol/l)	1193	0.05	-0.15	0.26	0.614	1193	0.10	-0.12	0.31	0.392	1193	0.06	-0.12	0.25	0.519
Total lipids in small VLDL (mmol/l)	1193	0.05	-0.15	0.26	0.619	1193	0.10	-0.13	0.32	0.395	1193	0.06	-0.13	0.24	0.533
Phospholipids in small VLDL (mmol/l)	1193	0.07	-0.14	0.28	0.486	1193	0.12	-0.11	0.35	0.313	1193	0.07	-0.11	0.26	0.439
Total cholesterol in small VLDL (mmol/l)	1193	0.05	-0.17	0.27	0.650	1193	0.09	-0.14	0.33	0.434	1193	0.04	-0.14	0.22	0.678
Cholesterol esters in small VLDL (mmol/l)	1193	0.03	-0.19	0.26	0.776	1193	0.07	-0.17	0.32	0.550	1193	0.02	-0.17	0.20	0.859
Free cholesterol in small VLDL (mmol/l)	1193	0.08	-0.13	0.29	0.445	1193	0.13	-0.10	0.35	0.280	1193	0.08	-0.11	0.26	0.416
Triglycerides in small VLDL (mmol/l)	1193	0.04	-0.16	0.24	0.719	1193	0.08	-0.14	0.29	0.486	1193	0.05	-0.14	0.24	0.597

S7 Table One-sample MR estimates of associations of puberty timing (per year later) with adiposity and cardiometabolic traits at age 18y among males and females in ALSPAC, using a full GRS of 351 SNPs for age at menarche

	Unadj.					*Adj. for measured BMI at age 8y*					*Adj. for measured outcome value at age 8y*				
**Standardised outcome at age 18y**	**N**	**Beta (2SLS)**	**LCL**	**UCL**	**P-value**	**N**	**Beta (2SLS)**	**LCL**	**UCL**	**P-value**	**N**	**Beta (2SLS)**	**LCL**	**UCL**	**P-value**
Concentration of very small VLDL particles (mol/l)	1193	0.11	-0.11	0.33	0.329	1193	0.15	-0.09	0.39	0.224	1193	0.07	-0.11	0.25	0.452
Total lipids in very small VLDL (mmol/l)	1193	0.07	-0.16	0.29	0.564	1193	0.10	-0.14	0.35	0.401	1193	0.03	-0.16	0.21	0.767
Phospholipids in very small VLDL (mmol/l)	1193	0.10	-0.12	0.33	0.360	1193	0.14	-0.10	0.39	0.257	1193	0.05	-0.13	0.23	0.556
Total cholesterol in very small VLDL (mmol/l)	1193	-0.01	-0.24	0.22	0.937	1193	0.02	-0.23	0.27	0.880	1193	-0.04	-0.24	0.17	0.716
Cholesterol esters in very small VLDL (mmol/l)	1193	-0.03	-0.26	0.21	0.822	1193	0.00	-0.25	0.25	0.987	1193	-0.05	-0.26	0.15	0.612
Free cholesterol in very small VLDL (mmol/l)	1193	0.03	-0.19	0.25	0.775	1193	0.06	-0.18	0.30	0.645	1193	0.01	-0.19	0.21	0.952
Triglycerides in very small VLDL (mmol/l)	1193	0.15	-0.07	0.36	0.175	1193	0.19	-0.04	0.42	0.111	1193	0.15	-0.06	0.35	0.157
Concentration of IDL particles (mol/l)	1193	0.15	-0.08	0.38	0.194	1193	0.19	-0.06	0.44	0.139	1193	0.10	-0.10	0.29	0.329
Total lipids in IDL (mmol/l)	1193	0.12	-0.11	0.34	0.316	1193	0.15	-0.10	0.40	0.231	1193	0.05	-0.13	0.23	0.605
Phospholipids in IDL (mmol/l)	1193	0.14	-0.08	0.37	0.213	1193	0.18	-0.07	0.43	0.159	1193	0.08	-0.11	0.28	0.398
Total cholesterol in IDL (mmol/l)	1193	0.07	-0.15	0.30	0.518	1193	0.11	-0.14	0.35	0.392	1193	0.00	-0.17	0.18	0.964
Cholesterol esters in IDL (mmol/l)	1193	0.05	-0.17	0.28	0.637	1193	0.09	-0.16	0.33	0.479	1193	-0.01	-0.19	0.16	0.883
Free cholesterol in IDL (mmol/l)	1193	0.12	-0.11	0.34	0.302	1193	0.15	-0.10	0.40	0.240	1193	0.05	-0.14	0.24	0.596
Triglycerides in IDL (mmol/l)	1193	0.24	0.01	0.47	0.042	1193	0.28	0.02	0.53	0.034	1193	0.22	0.00	0.45	0.047
Concentration of large LDL particles (mol/l)	1193	0.15	-0.07	0.38	0.188	1193	0.19	-0.06	0.44	0.135	1193	0.10	-0.10	0.30	0.314
Total lipids in large LDL (mmol/l)	1193	0.14	-0.09	0.36	0.236	1193	0.18	-0.07	0.43	0.170	1193	0.07	-0.12	0.27	0.449
Phospholipids in large LDL (mmol/l)	1193	0.12	-0.11	0.35	0.302	1193	0.16	-0.09	0.41	0.216	1193	0.06	-0.13	0.25	0.567
Total cholesterol in large LDL (mmol/l)	1193	0.12	-0.11	0.34	0.305	1193	0.15	-0.09	0.40	0.222	1193	0.05	-0.14	0.24	0.594
Cholesterol esters in large LDL (mmol/l)	1193	0.12	-0.11	0.34	0.307	1193	0.16	-0.09	0.40	0.219	1193	0.05	-0.13	0.24	0.590
Free cholesterol in large LDL (mmol/l)	1193	0.12	-0.11	0.35	0.301	1193	0.15	-0.10	0.40	0.234	1193	0.05	-0.14	0.24	0.602
Triglycerides in large LDL (mmol/l)	1193	0.27	0.03	0.51	0.026	1193	0.31	0.04	0.57	0.022	1193	0.25	0.02	0.49	0.031
Concentration of medium LDL particles (mol/l)	1193	0.16	-0.07	0.39	0.170	1193	0.20	-0.05	0.45	0.119	1193	0.12	-0.09	0.33	0.258
Total lipids in medium LDL (mmol/l)	1193	0.14	-0.09	0.37	0.225	1193	0.18	-0.07	0.43	0.159	1193	0.09	-0.11	0.28	0.390
Phospholipids in medium LDL (mmol/l)	1193	0.11	-0.12	0.33	0.351	1193	0.15	-0.10	0.40	0.242	1193	0.04	-0.15	0.23	0.668
Total cholesterol in medium LDL (mmol/l)	1193	0.12	-0.10	0.35	0.285	1193	0.16	-0.09	0.41	0.205	1193	0.07	-0.13	0.26	0.498
Cholesterol esters in medium LDL (mmol/l)	1193	0.13	-0.10	0.36	0.257	1193	0.17	-0.08	0.42	0.182	1193	0.08	-0.12	0.27	0.426
Free cholesterol in medium LDL (mmol/l)	1193	0.09	-0.14	0.32	0.433	1193	0.12	-0.12	0.37	0.329	1193	0.01	-0.17	0.20	0.879
Triglycerides in medium LDL (mmol/l)	1193	0.30	0.05	0.54	0.017	1193	0.34	0.06	0.61	0.016	1193	0.29	0.05	0.53	0.019
Concentration of small LDL particles (mol/l)	1193	0.16	-0.07	0.39	0.171	1193	0.20	-0.05	0.45	0.120	1193	0.12	-0.09	0.33	0.271
Total lipids in small LDL (mmol/l)	1193	0.14	-0.09	0.37	0.229	1193	0.18	-0.07	0.43	0.162	1193	0.09	-0.11	0.28	0.395
Phospholipids in small LDL (mmol/l)	1193	0.12	-0.11	0.35	0.296	1193	0.16	-0.09	0.41	0.208	1193	0.06	-0.13	0.25	0.543
Total cholesterol in small LDL (mmol/l)	1193	0.12	-0.10	0.35	0.290	1193	0.16	-0.09	0.41	0.211	1193	0.07	-0.13	0.26	0.506
Cholesterol esters in small LDL (mmol/l)	1193	0.14	-0.09	0.36	0.240	1193	0.17	-0.08	0.42	0.172	1193	0.09	-0.11	0.29	0.390
Free cholesterol in small LDL (mmol/l)	1193	0.06	-0.16	0.29	0.585	1193	0.09	-0.16	0.34	0.464	1193	-0.01	-0.20	0.18	0.905
Triglycerides in small LDL (mmol/l)	1193	0.26	0.03	0.50	0.026	1193	0.31	0.05	0.57	0.019	1193	0.26	0.03	0.49	0.028
Concentration of very large HDL particles (mol/l)	1193	0.23	-0.02	0.48	0.067	1193	0.24	-0.03	0.50	0.082	1193	0.18	-0.03	0.39	0.089
Total lipids in very large HDL (mmol/l)	1193	0.23	-0.02	0.47	0.071	1193	0.23	-0.03	0.50	0.086	1193	0.18	-0.03	0.40	0.092
Phospholipids in very large HDL (mmol/l)	1193	0.22	-0.02	0.46	0.074	1193	0.22	-0.04	0.49	0.095	1193	0.17	-0.03	0.37	0.099
Total cholesterol in very large HDL (mmol/l)	1193	0.20	-0.04	0.45	0.103	1193	0.21	-0.05	0.48	0.118	1193	0.17	-0.05	0.40	0.130
Cholesterol esters in very large HDL (mmol/l)	1193	0.20	-0.05	0.44	0.118	1193	0.21	-0.06	0.47	0.131	1193	0.17	-0.06	0.40	0.144
Free cholesterol in very large HDL (mmol/l)	1193	0.21	-0.03	0.45	0.090	1193	0.22	-0.05	0.48	0.108	1193	0.17	-0.04	0.38	0.120
Triglycerides in very large HDL (mmol/l)	1193	0.25	0.02	0.48	0.034	1193	0.29	0.03	0.54	0.027	1193	0.23	0.02	0.45	0.032
Concentration of large HDL particles (mol/l)	1193	0.19	-0.05	0.43	0.117	1193	0.19	-0.07	0.44	0.158	1193	0.13	-0.07	0.33	0.214
Total lipids in large HDL (mmol/l)	1193	0.17	-0.06	0.41	0.147	1193	0.17	-0.09	0.42	0.197	1193	0.11	-0.08	0.31	0.264
Phospholipids in large HDL (mmol/l)	1193	0.19	-0.05	0.42	0.122	1193	0.18	-0.07	0.44	0.158	1193	0.12	-0.08	0.33	0.233
Total cholesterol in large HDL (mmol/l)	1193	0.16	-0.08	0.39	0.187	1193	0.14	-0.11	0.39	0.258	1193	0.10	-0.09	0.29	0.315
Cholesterol esters in large HDL (mmol/l)	1193	0.16	-0.07	0.39	0.180	1193	0.15	-0.10	0.40	0.251	1193	0.10	-0.09	0.29	0.299
Free cholesterol in large HDL (mmol/l)	1193	0.14	-0.09	0.37	0.219	1193	0.13	-0.12	0.38	0.295	1193	0.08	-0.11	0.27	0.393
Triglycerides in large HDL (mmol/l)	1193	0.22	-0.01	0.44	0.060	1193	0.24	-0.01	0.49	0.055	1193	0.18	-0.03	0.38	0.090
Concentration of medium HDL particles (mol/l)	1193	0.12	-0.10	0.35	0.295	1193	0.13	-0.12	0.38	0.301	1193	0.09	-0.13	0.31	0.437
Total lipids in medium HDL (mmol/l)	1193	0.11	-0.12	0.33	0.360	1193	0.11	-0.13	0.35	0.379	1193	0.07	-0.15	0.29	0.537
Phospholipids in medium HDL (mmol/l)	1193	0.15	-0.08	0.37	0.213	1193	0.16	-0.10	0.41	0.224	1193	0.11	-0.11	0.33	0.322
Total cholesterol in medium HDL (mmol/l)	1193	0.04	-0.18	0.26	0.719	1193	0.03	-0.21	0.27	0.786	1193	0.00	-0.22	0.22	0.996
Cholesterol esters in medium HDL (mmol/l)	1193	0.03	-0.19	0.26	0.763	1193	0.02	-0.21	0.26	0.841	1193	-0.01	-0.22	0.21	0.959
Free cholesterol in medium HDL (mmol/l)	1193	0.07	-0.15	0.29	0.535	1193	0.07	-0.17	0.32	0.552	1193	0.03	-0.19	0.25	0.799
Triglycerides in medium HDL (mmol/l)	1193	0.12	-0.10	0.33	0.282	1193	0.16	-0.07	0.40	0.179	1193	0.11	-0.09	0.32	0.268

S7 Table One-sample MR estimates of associations of puberty timing (per year later) with adiposity and cardiometabolic traits at age 18y among males and females in ALSPAC, using a full GRS of 351 SNPs for age at menarche

	Unadj.					*Adj. for measured BMI at age 8y*					*Adj. for measured outcome value at age 8y*				
**Standardised outcome at age 18y**	**N**	**Beta (2SLS)**	**LCL**	**UCL**	**P-value**	**N**	**Beta (2SLS)**	**LCL**	**UCL**	**P-value**	**N**	**Beta (2SLS)**	**LCL**	**UCL**	**P-value**
Concentration of small HDL particles (mol/l)	1193	0.07	-0.15	0.29	0.556	1193	0.08	-0.16	0.32	0.511	1193	0.05	-0.16	0.27	0.630
Total lipids in small HDL (mmol/l)	1193	0.10	-0.12	0.33	0.373	1193	0.12	-0.13	0.36	0.358	1193	0.09	-0.14	0.31	0.442
Phospholipids in small HDL (mmol/l)	1193	0.02	-0.19	0.24	0.830	1193	0.03	-0.21	0.27	0.808	1193	0.01	-0.20	0.22	0.929
Total cholesterol in small HDL (mmol/l)	1193	0.14	-0.09	0.37	0.224	1193	0.16	-0.10	0.41	0.228	1193	0.12	-0.10	0.35	0.276
Cholesterol esters in small HDL (mmol/l)	1193	0.15	-0.08	0.39	0.197	1193	0.17	-0.09	0.42	0.195	1193	0.14	-0.09	0.36	0.237
Free cholesterol in small HDL (mmol/l)	1193	0.06	-0.16	0.28	0.580	1193	0.06	-0.18	0.30	0.627	1193	0.04	-0.18	0.26	0.714
Triglycerides in small HDL (mmol/l)	1193	0.17	-0.04	0.39	0.121	1193	0.21	-0.02	0.45	0.074	1193	0.18	-0.03	0.39	0.100
Phospholipids to total lipds ratio in chylomicrons and extremely large VLDL (%)	1193	-0.10	-0.39	0.19	0.499	1193	-0.09	-0.41	0.22	0.558	1193	-0.10	-0.40	0.19	0.482
Total cholesterol to total lipids ratio in chylomicrons and extremely large VLDL (%)	1193	0.12	-0.09	0.34	0.260	1193	0.14	-0.09	0.38	0.238	1193	0.11	-0.10	0.32	0.298
Cholesterol esters to total lipids ratio in chylomicrons and extremely large VLDL (%)	1193	0.08	-0.13	0.29	0.455	1193	0.09	-0.14	0.32	0.461	1193	0.07	-0.13	0.28	0.498
Free cholesterol to total lipids ratio in chylomicrons and extremely large VLDL (%)	1193	0.17	-0.05	0.39	0.121	1193	0.22	-0.02	0.45	0.073	1193	0.16	-0.05	0.37	0.131
Triglycerides to total lipids ratio in chylomicrons and extremely large VLDL (%)	1193	-0.16	-0.41	0.10	0.234	1193	-0.19	-0.48	0.10	0.198	1193	-0.14	-0.39	0.10	0.251
Phospholipids to total lipds ratio in very large VLDL (%)	1193	0.09	-0.15	0.33	0.452	1193	0.13	-0.12	0.39	0.308	1193	0.07	-0.15	0.29	0.536
Total cholesterol to total lipids ratio in very large VLDL (%)	1193	0.02	-0.15	0.20	0.803	1193	0.02	-0.17	0.21	0.807	1193	0.00	-0.17	0.18	0.956
Cholesterol esters to total lipids ratio in very large VLDL (%)	1193	0.00	-0.14	0.14	0.969	1193	-0.01	-0.16	0.14	0.909	1193	-0.01	-0.15	0.12	0.843
Free cholesterol to total lipids ratio in very large VLDL (%)	1193	0.08	-0.07	0.23	0.307	1193	0.09	-0.07	0.26	0.263	1193	0.06	-0.08	0.21	0.403
Triglycerides to total lipids ratio in very large VLDL (%)	1193	-0.06	-0.27	0.15	0.557	1193	-0.08	-0.30	0.15	0.505	1193	-0.03	-0.23	0.17	0.762
Phospholipids to total lipds ratio in large VLDL (%)	1193	0.13	-0.08	0.34	0.240	1193	0.16	-0.07	0.39	0.174	1193	0.12	-0.08	0.32	0.257
Total cholesterol to total lipids ratio in large VLDL (%)	1193	0.21	-0.01	0.43	0.064	1193	0.25	0.01	0.49	0.039	1193	0.17	-0.03	0.38	0.098
Cholesterol esters to total lipids ratio in large VLDL (%)	1193	0.21	0.00	0.42	0.050	1193	0.25	0.02	0.48	0.036	1193	0.19	-0.01	0.39	0.069
Free cholesterol to total lipids ratio in large VLDL (%)	1193	0.04	-0.05	0.13	0.330	1193	0.06	-0.04	0.16	0.231	1193	0.04	-0.04	0.13	0.350
Triglycerides to total lipids ratio in large VLDL (%)	1193	-0.17	-0.39	0.04	0.121	1193	-0.21	-0.45	0.02	0.076	1193	-0.14	-0.35	0.06	0.165
Phospholipids to total lipds ratio in medium VLDL (%)	1193	0.17	-0.05	0.39	0.123	1193	0.18	-0.06	0.42	0.142	1193	0.12	-0.08	0.33	0.245
Total cholesterol to total lipids ratio in medium VLDL (%)	1193	0.19	-0.03	0.41	0.085	1193	0.23	-0.01	0.47	0.064	1193	0.15	-0.05	0.35	0.140
Cholesterol esters to total lipids ratio in medium VLDL (%)	1193	0.16	-0.06	0.38	0.145	1193	0.19	-0.05	0.43	0.116	1193	0.13	-0.07	0.33	0.204
Free cholesterol to total lipids ratio in medium VLDL (%)	1193	0.21	-0.01	0.43	0.065	1193	0.24	0.00	0.48	0.047	1193	0.18	-0.02	0.39	0.078
Triglycerides to total lipids ratio in medium VLDL (%)	1193	-0.21	-0.43	0.01	0.067	1193	-0.24	-0.49	0.00	0.054	1193	-0.15	-0.35	0.05	0.135
Phospholipids to total lipds ratio in small VLDL (%)	1193	0.00	-0.21	0.21	0.964	1193	-0.03	-0.25	0.20	0.801	1193	-0.01	-0.21	0.19	0.900
Total cholesterol to total lipids ratio in small VLDL (%)	1193	0.01	-0.22	0.23	0.961	1193	0.01	-0.23	0.26	0.912	1193	-0.02	-0.24	0.19	0.850
Cholesterol esters to total lipids ratio in small VLDL (%)	1193	-0.02	-0.25	0.21	0.893	1193	-0.01	-0.25	0.24	0.959	1193	-0.04	-0.25	0.18	0.739
Free cholesterol to total lipids ratio in small VLDL (%)	1193	0.15	-0.07	0.37	0.186	1193	0.14	-0.10	0.38	0.242	1193	0.10	-0.11	0.31	0.347
Triglycerides to total lipids ratio in small VLDL (%)	1193	-0.01	-0.23	0.21	0.936	1193	-0.01	-0.24	0.23	0.962	1193	0.01	-0.20	0.23	0.892
Phospholipids to total lipds ratio in very small VLDL (%)	1193	0.15	-0.07	0.37	0.177	1193	0.17	-0.07	0.42	0.158	1193	0.11	-0.09	0.31	0.293
Total cholesterol to total lipids ratio in very small VLDL (%)	1193	-0.21	-0.44	0.02	0.072	1193	-0.23	-0.49	0.02	0.069	1193	-0.21	-0.44	0.02	0.069
Cholesterol esters to total lipids ratio in very small VLDL (%)	1193	-0.20	-0.43	0.03	0.093	1193	-0.21	-0.47	0.04	0.099	1193	-0.20	-0.42	0.03	0.089
Free cholesterol to total lipids ratio in very small VLDL (%)	1193	-0.11	-0.29	0.07	0.223	1193	-0.15	-0.34	0.05	0.141	1193	-0.11	-0.29	0.07	0.218
Triglycerides to total lipids ratio in very small VLDL (%)	1193	0.15	-0.07	0.37	0.182	1193	0.16	-0.08	0.40	0.184	1193	0.16	-0.06	0.38	0.148
Phospholipids to total lipds ratio in IDL (%)	1193	0.13	-0.08	0.34	0.238	1193	0.11	-0.12	0.33	0.347	1193	0.13	-0.08	0.34	0.219
Total cholesterol to total lipids ratio in IDL (%)	1193	-0.20	-0.43	0.04	0.100	1193	-0.19	-0.44	0.06	0.135	1193	-0.21	-0.44	0.01	0.067
Cholesterol esters to total lipids ratio in IDL (%)	1193	-0.21	-0.45	0.02	0.074	1193	-0.21	-0.46	0.05	0.112	1193	-0.22	-0.45	0.01	0.060
Free cholesterol to total lipids ratio in IDL (%)	1193	0.03	-0.19	0.24	0.810	1193	0.02	-0.21	0.24	0.894	1193	0.00	-0.20	0.20	0.988
Triglycerides to total lipids ratio in IDL (%)	1193	0.18	-0.05	0.41	0.123	1193	0.19	-0.06	0.43	0.146	1193	0.20	-0.02	0.42	0.074
Phospholipids to total lipds ratio in large LDL (%)	1193	-0.17	-0.39	0.06	0.150	1193	-0.20	-0.45	0.05	0.111	1193	-0.10	-0.29	0.09	0.281
Total cholesterol to total lipids ratio in large LDL (%)	1193	-0.02	-0.23	0.20	0.887	1193	0.01	-0.23	0.24	0.951	1193	-0.07	-0.25	0.11	0.432
Cholesterol esters to total lipids ratio in large LDL (%)	1193	0.05	-0.16	0.27	0.633	1193	0.09	-0.15	0.33	0.458	1193	-0.01	-0.18	0.15	0.874
Free cholesterol to total lipids ratio in large LDL (%)	1193	-0.20	-0.42	0.02	0.072	1193	-0.25	-0.49	-0.01	0.039	1193	-0.19	-0.39	0.01	0.066
Triglycerides to total lipids ratio in large LDL (%)	1193	0.21	-0.02	0.44	0.073	1193	0.21	-0.04	0.47	0.094	1193	0.23	0.01	0.45	0.041
Phospholipids to total lipds ratio in medium LDL (%)	1193	-0.14	-0.37	0.09	0.223	1193	-0.17	-0.42	0.08	0.173	1193	-0.12	-0.32	0.09	0.267
Total cholesterol to total lipids ratio in medium LDL (%)	1193	0.01	-0.21	0.22	0.947	1193	0.03	-0.20	0.26	0.795	1193	-0.03	-0.20	0.15	0.753
Cholesterol esters to total lipids ratio in medium LDL (%)	1193	0.09	-0.13	0.31	0.417	1193	0.13	-0.12	0.37	0.305	1193	0.05	-0.13	0.24	0.564
Free cholesterol to total lipids ratio in medium LDL (%)	1193	-0.20	-0.42	0.03	0.095	1193	-0.24	-0.49	0.01	0.062	1193	-0.17	-0.38	0.04	0.106
Triglycerides to total lipids ratio in medium LDL (%)	1193	0.26	0.04	0.49	0.022	1193	0.27	0.03	0.52	0.027	1193	0.27	0.05	0.49	0.016
Phospholipids to total lipds ratio in small LDL (%)	1193	-0.12	-0.34	0.10	0.277	1193	-0.16	-0.40	0.09	0.205	1193	-0.09	-0.28	0.10	0.362
Total cholesterol to total lipids ratio in small LDL (%)	1193	0.02	-0.20	0.23	0.877	1193	0.04	-0.20	0.27	0.748	1193	-0.02	-0.20	0.17	0.844
Cholesterol esters to total lipids ratio in small LDL (%)	1193	0.10	-0.12	0.33	0.363	1193	0.14	-0.11	0.38	0.271	1193	0.07	-0.12	0.26	0.484
Free cholesterol to total lipids ratio in small LDL (%)	1193	-0.23	-0.46	0.00	0.053	1193	-0.27	-0.53	-0.02	0.035	1193	-0.21	-0.43	0.01	0.057

S7 Table One-sample MR estimates of associations of puberty timing (per year later) with adiposity and cardiometabolic traits at age 18y among males and females in ALSPAC, using a full GRS of 351 SNPs for age at menarche

	Unadj.					*Adj. for measured BMI at age 8y*					*Adj. for measured outcome value at age 8y*				
**Standardised outcome at age 18y**	**N**	**Beta (2SLS)**	**LCL**	**UCL**	**P-value**	**N**	**Beta (2SLS)**	**LCL**	**UCL**	**P-value**	**N**	**Beta (2SLS)**	**LCL**	**UCL**	**P-value**
Triglycerides to total lipids ratio in small LDL (%)	1193	0.27	0.04	0.51	0.023	1193	0.30	0.05	0.56	0.021	1193	0.28	0.05	0.51	0.018
Phospholipids to total lipds ratio in very large HDL (%)	1193	0.20	-0.03	0.43	0.091	1193	0.19	-0.06	0.44	0.132	1193	0.18	-0.02	0.39	0.083
Total cholesterol to total lipids ratio in very large HDL (%)	1193	-0.22	-0.46	0.02	0.068	1193	-0.22	-0.48	0.04	0.092	1193	-0.20	-0.41	0.01	0.061
Cholesterol esters to total lipids ratio in very large HDL (%)	1193	-0.21	-0.45	0.02	0.076	1193	-0.21	-0.47	0.04	0.102	1193	-0.19	-0.40	0.02	0.070
Free cholesterol to total lipids ratio in very large HDL (%)	1193	0.00	-0.23	0.23	0.978	1193	-0.01	-0.26	0.23	0.928	1193	-0.01	-0.23	0.21	0.933
Triglycerides to total lipids ratio in very large HDL (%)	1193	0.05	-0.15	0.25	0.626	1193	0.09	-0.12	0.31	0.407	1193	0.05	-0.14	0.23	0.623
Phospholipids to total lipds ratio in large HDL (%)	1193	0.01	-0.20	0.22	0.937	1193	0.06	-0.17	0.28	0.623	1193	0.00	-0.19	0.20	0.989
Total cholesterol to total lipids ratio in large HDL (%)	1193	0.01	-0.19	0.22	0.895	1193	-0.03	-0.26	0.19	0.764	1193	0.02	-0.17	0.20	0.863
Cholesterol esters to total lipids ratio in large HDL (%)	1193	0.03	-0.18	0.25	0.760	1193	-0.01	-0.24	0.21	0.911	1193	0.04	-0.14	0.22	0.666
Free cholesterol to total lipids ratio in large HDL (%)	1193	-0.07	-0.27	0.13	0.520	1193	-0.11	-0.33	0.11	0.319	1193	-0.08	-0.27	0.11	0.394
Triglycerides to total lipids ratio in large HDL (%)	1193	-0.05	-0.26	0.15	0.617	1193	-0.02	-0.23	0.20	0.877	1193	-0.04	-0.22	0.14	0.642
Phospholipids to total lipds ratio in medium HDL (%)	1193	0.22	-0.03	0.46	0.080	1193	0.24	-0.03	0.51	0.076	1193	0.21	-0.03	0.45	0.085
Total cholesterol to total lipids ratio in medium HDL (%)	1193	-0.21	-0.44	0.03	0.087	1193	-0.25	-0.51	0.01	0.061	1193	-0.20	-0.43	0.03	0.082
Cholesterol esters to total lipids ratio in medium HDL (%)	1193	-0.20	-0.44	0.03	0.087	1193	-0.25	-0.51	0.01	0.059	1193	-0.20	-0.42	0.03	0.086
Free cholesterol to total lipids ratio in medium HDL (%)	1193	-0.10	-0.30	0.10	0.318	1193	-0.11	-0.32	0.11	0.330	1193	-0.12	-0.30	0.07	0.232
Triglycerides to total lipids ratio in medium HDL (%)	1193	0.06	-0.15	0.27	0.576	1193	0.10	-0.12	0.33	0.363	1193	0.08	-0.11	0.27	0.407
Phospholipids to total lipds ratio in small HDL (%)	1193	-0.17	-0.40	0.06	0.154	1193	-0.19	-0.44	0.07	0.151	1193	-0.15	-0.37	0.06	0.165
Total cholesterol to total lipids ratio in small HDL (%)	1193	0.13	-0.09	0.35	0.255	1193	0.13	-0.11	0.37	0.276	1193	0.11	-0.10	0.32	0.291
Cholesterol esters to total lipids ratio in small HDL (%)	1193	0.13	-0.09	0.35	0.250	1193	0.14	-0.10	0.39	0.252	1193	0.12	-0.09	0.33	0.279
Free cholesterol to total lipids ratio in small HDL (%)	1193	-0.08	-0.32	0.15	0.478	1193	-0.13	-0.38	0.13	0.328	1193	-0.09	-0.31	0.13	0.403
Triglycerides to total lipids ratio in small HDL (%)	1193	0.13	-0.07	0.33	0.204	1193	0.17	-0.05	0.39	0.120	1193	0.15	-0.05	0.34	0.148
Mean diameter for VLDL particles (nm)	1193	0.01	-0.19	0.22	0.888	1193	0.05	-0.17	0.27	0.674	1193	0.03	-0.17	0.22	0.772
Mean diameter for LDL particles (nm)	1193	-0.05	-0.27	0.16	0.622	1193	-0.07	-0.31	0.16	0.540	1193	-0.04	-0.26	0.17	0.693
Mean diameter for HDL particles (nm)	1193	0.20	-0.04	0.44	0.100	1193	0.20	-0.06	0.45	0.134	1193	0.14	-0.05	0.34	0.147
Serum total cholesterol (mmol/l)	1193	0.15	-0.08	0.37	0.214	1193	0.18	-0.07	0.43	0.157	1193	0.07	-0.12	0.26	0.453
Total cholesterol in VLDL (mmol/l)	1193	0.03	-0.18	0.24	0.774	1193	0.08	-0.15	0.31	0.503	1193	0.03	-0.16	0.21	0.786
Remnant cholesterol (non-HDL, non-LDL -cholesterol) (mmol/l)	1193	0.05	-0.17	0.27	0.628	1193	0.10	-0.14	0.34	0.418	1193	0.02	-0.16	0.19	0.854
Total cholesterol in LDL (mmol/l)	1193	0.12	-0.11	0.35	0.295	1193	0.16	-0.09	0.41	0.214	1193	0.06	-0.13	0.25	0.546
Total cholesterol in HDL (mmol/l)	1193	0.17	-0.06	0.41	0.151	1193	0.17	-0.08	0.43	0.189	1193	0.12	-0.09	0.32	0.270
Total cholesterol in HDL2 (mmol/l)	1193	0.15	-0.08	0.39	0.194	1193	0.15	-0.10	0.40	0.255	1193	0.10	-0.10	0.30	0.343
Total cholesterol in HDL3 (mmol/l)	1193	0.20	-0.04	0.44	0.104	1193	0.21	-0.05	0.47	0.118	1193	0.14	-0.07	0.35	0.180
Esterified cholesterol (mmol/l)	1193	0.14	-0.09	0.37	0.242	1193	0.17	-0.08	0.43	0.184	1193	0.07	-0.13	0.27	0.496
Free cholesterol (mmol/l)	1193	0.15	-0.07	0.37	0.194	1193	0.18	-0.06	0.43	0.138	1193	0.08	-0.10	0.26	0.379
Serum total triglycerides (mmol/l)	1193	0.09	-0.11	0.29	0.368	1193	0.14	-0.08	0.35	0.205	1193	0.10	-0.09	0.29	0.312
Triglycerides in VLDL (mmol/l)	1193	0.02	-0.17	0.22	0.817	1193	0.07	-0.14	0.28	0.536	1193	0.03	-0.15	0.22	0.712
Triglycerides in LDL (mmol/l)	1193	0.28	0.04	0.52	0.022	1193	0.32	0.05	0.59	0.019	1193	0.27	0.03	0.51	0.025
Triglycerides in HDL (mmol/l)	1193	0.21	-0.01	0.43	0.063	1193	0.26	0.01	0.50	0.038	1193	0.20	-0.01	0.41	0.060
Diacylglycerol (mmol/l)	1193	0.00	-0.21	0.21	0.992	1193	0.03	-0.20	0.25	0.820	1193	0.00	-0.20	0.20	0.990
Ratio of diacylglycerol to triglycerides	1193	-0.03	-0.24	0.18	0.768	1193	-0.02	-0.25	0.20	0.842	1193	-0.03	-0.24	0.17	0.759
Total phosphoglycerides (mmol/l)	1193	0.24	0.00	0.47	0.051	1193	0.27	0.01	0.54	0.041	1193	0.19	-0.04	0.41	0.099
Ratio of triglycerides to phosphoglycerides	1193	0.01	-0.19	0.22	0.893	1193	0.05	-0.17	0.26	0.679	1193	0.03	-0.17	0.22	0.766
Phosphatidylcholine and other cholines (mmol/l)	1193	0.25	0.02	0.49	0.037	1193	0.28	0.02	0.54	0.037	1193	0.21	-0.01	0.43	0.065
Total cholines (mmol/l)	1193	0.22	-0.02	0.46	0.070	1193	0.25	-0.01	0.52	0.062	1193	0.17	-0.05	0.39	0.136
Apolipoprotein A-I (g/l)	1193	0.18	-0.05	0.42	0.128	1193	0.20	-0.06	0.45	0.136	1193	0.12	-0.09	0.33	0.252
Apolipoprotein B (g/l)	1193	0.08	-0.13	0.30	0.458	1193	0.13	-0.10	0.36	0.279	1193	0.06	-0.12	0.24	0.510
Ratio of apolipoprotein B to apolipoprotein A-I	1193	0.00	-0.22	0.21	0.985	1193	0.04	-0.19	0.27	0.730	1193	0.01	-0.17	0.19	0.945
Total fatty acids (mmol/l)	1193	0.15	-0.07	0.37	0.168	1193	0.20	-0.04	0.44	0.105	1193	0.13	-0.08	0.33	0.226
Estimated description of fatty acid chain length, not actual carbon number	1193	0.15	-0.06	0.35	0.163	1193	0.16	-0.06	0.38	0.165	1193	0.17	-0.04	0.37	0.111
Estimated degree of unsaturation	1193	0.06	-0.14	0.25	0.575	1193	0.05	-0.17	0.26	0.669	1193	0.07	-0.12	0.26	0.487
22:6, docosahexaenoic acid (mmol/l)	1193	0.08	-0.15	0.30	0.502	1193	0.10	-0.14	0.34	0.421	1193	0.08	-0.13	0.29	0.439
18:2, linoleic acid (mmol/l)	1193	0.21	-0.03	0.44	0.084	1193	0.24	-0.02	0.50	0.074	1193	0.17	-0.04	0.37	0.115
Conjugated linoleic acid (mmol/l)	1193	0.05	-0.13	0.23	0.583	1193	0.08	-0.11	0.28	0.412	1193	0.05	-0.13	0.23	0.572
Omega-3 fatty acids (mmol/l)	1193	0.05	-0.17	0.27	0.677	1193	0.08	-0.17	0.32	0.537	1193	0.02	-0.19	0.22	0.865
Omega-6 fatty acids (mmol/l)	1193	0.19	-0.04	0.42	0.105	1193	0.23	-0.03	0.48	0.082	1193	0.14	-0.06	0.34	0.171
Polyunsaturated fatty acids (mmol/l)	1193	0.18	-0.05	0.41	0.127	1193	0.22	-0.04	0.47	0.096	1193	0.13	-0.07	0.33	0.211
Monounsaturated fatty acids; 16:1, 18:1 (mmol/l)	1193	0.14	-0.07	0.36	0.182	1193	0.19	-0.04	0.43	0.104	1193	0.14	-0.06	0.34	0.179

S7 Table One-sample MR estimates of associations of puberty timing (per year later) with adiposity and cardiometabolic traits at age 18y among males and females in ALSPAC, using a full GRS of 351 SNPs for age at menarche

	Unadj.					*Adj. for measured BMI at age 8y*					*Adj. for measured outcome value at age 8y*				
**Standardised outcome at age 18y**	**N**	**Beta (2SLS)**	**LCL**	**UCL**	**P-value**	**N**	**Beta (2SLS)**	**LCL**	**UCL**	**P-value**	**N**	**Beta (2SLS)**	**LCL**	**UCL**	**P-value**
Saturated fatty acids (mmol/l)	1193	0.11	-0.11	0.33	0.318	1193	0.15	-0.09	0.39	0.209	1193	0.09	-0.12	0.29	0.423
Ratio of 22:6 docosahexaenoic acid to total fatty acids (%)	1193	-0.03	-0.24	0.19	0.817	1193	-0.03	-0.26	0.21	0.831	1193	0.00	-0.20	0.20	0.996
Ratio of 18:2 linoleic acid to total fatty acids (%)	1193	0.12	-0.10	0.34	0.300	1193	0.09	-0.15	0.33	0.461	1193	0.11	-0.11	0.33	0.322
Ratio of conjugated linoleic acid to total fatty acids (%)	1193	0.00	-0.17	0.17	0.972	1193	0.02	-0.16	0.21	0.814	1193	0.00	-0.17	0.17	0.961
Ratio of omega-3 fatty acids to total fatty acids (%)	1193	-0.09	-0.32	0.13	0.417	1193	-0.10	-0.35	0.15	0.418	1193	-0.10	-0.32	0.12	0.372
Ratio of omega-6 fatty acids to total fatty acids (%)	1193	0.08	-0.14	0.29	0.477	1193	0.05	-0.18	0.28	0.659	1193	0.07	-0.14	0.28	0.520
Ratio of polyunsaturated fatty acids to total fatty acids (%)	1193	0.05	-0.16	0.26	0.645	1193	0.02	-0.20	0.25	0.839	1193	0.04	-0.17	0.25	0.702
Ratio of monounsaturated fatty acids to total fatty acids (%)	1193	0.07	-0.15	0.29	0.542	1193	0.10	-0.14	0.33	0.421	1193	0.09	-0.12	0.30	0.412
Ratio of saturated fatty acids to total fatty acids (%)	1193	-0.15	-0.37	0.06	0.163	1193	-0.16	-0.40	0.08	0.181	1193	-0.16	-0.38	0.05	0.138
Glucose (mmol/l)	1193	-0.07	-0.23	0.09	0.386	1193	-0.07	-0.24	0.11	0.458	1193	-0.04	-0.20	0.11	0.602
Lactate (mmol/l)	1193	0.12	-0.12	0.35	0.337	1193	0.13	-0.13	0.38	0.328	1193	0.10	-0.13	0.34	0.384
Pyruvate (mmol/l)	1193	0.11	-0.11	0.33	0.320	1193	0.14	-0.09	0.38	0.238	1193	0.10	-0.12	0.31	0.373
Citrate (mmol/l)	1193	0.27	0.04	0.50	0.021	1193	0.26	0.01	0.50	0.039	1193	0.26	0.04	0.48	0.020
Alanine (mmol/l)	1193	0.17	-0.05	0.39	0.136	1193	0.20	-0.05	0.44	0.115	1193	0.16	-0.06	0.39	0.146
Glutamine (mmol/l)	1193	-0.13	-0.36	0.09	0.247	1193	-0.17	-0.41	0.08	0.187	1193	-0.14	-0.37	0.08	0.218
Histidine (mmol/l)	1193	0.01	-0.22	0.23	0.959	1193	0.01	-0.23	0.25	0.964	1193	0.01	-0.21	0.23	0.956
Isoleucine (mmol/l)	1193	-0.01	-0.22	0.19	0.910	1193	0.01	-0.21	0.23	0.946	1193	-0.02	-0.23	0.19	0.859
Leucine (mmol/l)	1193	-0.13	-0.35	0.10	0.266	1193	-0.13	-0.37	0.12	0.306	1193	-0.13	-0.36	0.10	0.260
Valine (mmol/l)	1193	-0.18	-0.40	0.05	0.129	1193	-0.17	-0.41	0.08	0.185	1193	-0.18	-0.41	0.04	0.112
Phenylalanine (mmol/l)	1193	-0.17	-0.42	0.07	0.167	1193	-0.16	-0.43	0.10	0.232	1193	-0.19	-0.43	0.06	0.133
Tyrosine (mmol/l)	1193	-0.14	-0.36	0.08	0.199	1193	-0.12	-0.36	0.12	0.321	1193	-0.15	-0.37	0.07	0.179
Acetate (mmol/l)	1193	-0.07	-0.20	0.05	0.234	1193	-0.09	-0.22	0.04	0.181	1193	-0.07	-0.20	0.05	0.232
Acetoacetate (mmol/l)	1193	-0.05	-0.28	0.18	0.699	1193	-0.04	-0.29	0.21	0.743	1193	-0.04	-0.27	0.19	0.706
3-hydroxybutyrate (mmol/l)	1193	0.02	-0.21	0.24	0.888	1193	0.02	-0.22	0.26	0.872	1193	0.02	-0.21	0.24	0.884
Creatinine (mmol/l)	1193	-0.37	-0.62	-0.12	0.004	1193	-0.39	-0.67	-0.11	0.006	1193	-0.32	-0.56	-0.08	0.009
Albumin (signal area)	1193	-0.23	-0.47	0.01	0.057	1193	-0.26	-0.51	0.00	0.054	1193	-0.24	-0.48	-0.01	0.044
Glycoprotein acetyls, mainly a1-acid glycoprotein (mmol/l)	1193	0.13	-0.09	0.36	0.235	1193	0.19	-0.05	0.43	0.118	1193	0.13	-0.09	0.34	0.253
