# Supplementary material for: Influence of puberty timing on adiposity and cardiometabolic traits: A Mendelian randomisation study
Source: PLoS Med. 2018 Aug 28;15(8):e1002641. doi: 10.1371/journal.pmed.1002641 (PMC6112630; doi:10.1371/journal.pmed.1002641)
Supplement: S8 Table — (PDF) [file pmed.1002641.s027.pdf]

S8 Table One-sample MR estimates of associations of age at voice breaking (per year later) with adiposity and cardiometabolic traits at age 18y among males in ALSPAC, using a refined GRS of 115 SNPs for age at menarche/voice breaking

	Unadj.					*Adj. for measured BMI at age 8y*					*Adj. for measured outcome value at age 8y*				
**Standardised outcome at age 18y**	**N**	**Beta (2SLS)**	**LCL**	**UCL**	**P-value**	**N**	**Beta (2SLS)**	**LCL**	**UCL**	**P-value**	**N**	**Beta (2SLS)**	**LCL**	**UCL**	**P-value**
Body mass index (kg/m^2)	1446	-0.20	-0.57	0.17	0.281	1337	-0.07	-0.38	0.23	0.633	1337	-0.07	-0.38	0.23	0.633
Fat mass index (kg/m^2)	1408	-0.02	-0.36	0.32	0.895	1303	0.05	-0.25	0.36	0.725	1266	0.13	-0.10	0.37	0.275
Lean mass index (kg/m^2)	1408	-0.25	-0.58	0.09	0.146	1303	-0.17	-0.49	0.15	0.302	1266	-0.16	-0.38	0.07	0.172
Systolic blood pressure (mmHg)	1371	-0.28	-0.72	0.16	0.214	1274	-0.25	-0.73	0.22	0.294	1258	-0.33	-0.78	0.12	0.155
Diastolic blood pressure (mmHg)	1371	-0.07	-0.48	0.35	0.756	1274	-0.08	-0.53	0.38	0.744	1257	-0.15	-0.57	0.28	0.498
Concentration of chylomicrons and extremely large VLDL particles (mol/l)	1066	0.12	-0.40	0.65	0.647	985	0.19	-0.38	0.77	0.511	775	0.13	-0.44	0.70	0.651
Total lipids in chylomicrons and extremely large VLDL (mmol/l)	1066	0.11	-0.41	0.64	0.672	985	0.18	-0.39	0.76	0.534	775	0.12	-0.44	0.69	0.672
Phospholipids in chylomicrons and extremely large VLDL (mmol/l)	1066	0.12	-0.40	0.65	0.647	985	0.19	-0.39	0.76	0.521	775	0.13	-0.43	0.70	0.648
Total cholesterol in chylomicrons and extremely large VLDL (mmol/l)	1066	0.11	-0.41	0.63	0.683	985	0.17	-0.40	0.74	0.558	775	0.10	-0.45	0.65	0.732
Cholesterol esters in chylomicrons and extremely large VLDL (mmol/l)	1066	0.10	-0.41	0.61	0.709	985	0.15	-0.41	0.72	0.591	775	0.06	-0.47	0.59	0.812
Free cholesterol in chylomicrons and extremely large VLDL (mmol/l)	1066	0.12	-0.41	0.64	0.658	985	0.19	-0.39	0.76	0.526	775	0.13	-0.44	0.70	0.652
Triglycerides in chylomicrons and extremely large VLDL (mmol/l)	1066	0.11	-0.41	0.64	0.674	985	0.18	-0.39	0.76	0.532	775	0.13	-0.44	0.70	0.661
Concentration of very large VLDL particles (mol/l)	1066	0.09	-0.45	0.62	0.749	985	0.15	-0.43	0.73	0.607	775	0.10	-0.47	0.67	0.738
Total lipids in very large VLDL (mmol/l)	1066	0.09	-0.44	0.62	0.746	985	0.15	-0.43	0.73	0.613	775	0.09	-0.48	0.66	0.757
Phospholipids in very large VLDL (mmol/l)	1066	0.11	-0.42	0.65	0.675	985	0.17	-0.41	0.75	0.567	775	0.10	-0.47	0.66	0.740
Total cholesterol in very large VLDL (mmol/l)	1066	0.09	-0.43	0.62	0.724	985	0.16	-0.41	0.73	0.584	775	0.09	-0.46	0.64	0.757
Cholesterol esters in very large VLDL (mmol/l)	1066	0.07	-0.45	0.59	0.792	985	0.14	-0.43	0.70	0.634	775	0.07	-0.48	0.61	0.805
Free cholesterol in very large VLDL (mmol/l)	1066	0.12	-0.40	0.65	0.652	985	0.18	-0.39	0.76	0.534	775	0.11	-0.45	0.67	0.706
Triglycerides in very large VLDL (mmol/l)	1066	0.08	-0.46	0.61	0.774	985	0.14	-0.44	0.72	0.637	775	0.09	-0.48	0.66	0.764
Concentration of large VLDL particles (mol/l)	1066	0.05	-0.48	0.57	0.863	985	0.10	-0.47	0.66	0.741	775	0.04	-0.52	0.59	0.894
Total lipids in large VLDL (mmol/l)	1066	0.05	-0.48	0.58	0.855	985	0.10	-0.47	0.66	0.733	775	0.04	-0.52	0.59	0.898
Phospholipids in large VLDL (mmol/l)	1066	0.06	-0.46	0.58	0.820	985	0.11	-0.45	0.67	0.704	775	0.04	-0.51	0.59	0.880
Total cholesterol in large VLDL (mmol/l)	1066	0.06	-0.46	0.57	0.830	985	0.11	-0.45	0.67	0.702	775	0.03	-0.51	0.57	0.912
Cholesterol esters in large VLDL (mmol/l)	1066	0.05	-0.46	0.55	0.859	985	0.10	-0.45	0.65	0.717	775	0.01	-0.51	0.53	0.964
Free cholesterol in large VLDL (mmol/l)	1066	0.07	-0.46	0.59	0.802	985	0.12	-0.45	0.69	0.689	775	0.05	-0.51	0.60	0.868
Triglycerides in large VLDL (mmol/l)	1066	0.04	-0.49	0.57	0.874	985	0.09	-0.48	0.66	0.754	775	0.04	-0.52	0.59	0.898
Concentration of medium VLDL particles (mol/l)	1066	0.01	-0.50	0.52	0.968	985	0.05	-0.50	0.60	0.861	775	-0.02	-0.54	0.51	0.955
Total lipids in medium VLDL (mmol/l)	1066	0.01	-0.50	0.52	0.969	985	0.05	-0.49	0.59	0.857	775	-0.02	-0.54	0.49	0.932
Phospholipids in medium VLDL (mmol/l)	1066	0.02	-0.48	0.52	0.929	985	0.06	-0.48	0.60	0.829	775	-0.02	-0.54	0.49	0.925
Total cholesterol in medium VLDL (mmol/l)	1066	0.04	-0.45	0.52	0.877	985	0.09	-0.44	0.61	0.747	775	-0.04	-0.52	0.44	0.882
Cholesterol esters in medium VLDL (mmol/l)	1066	0.03	-0.43	0.50	0.893	985	0.09	-0.42	0.59	0.741	775	-0.07	-0.52	0.38	0.766
Free cholesterol in medium VLDL (mmol/l)	1066	0.04	-0.46	0.55	0.865	985	0.08	-0.46	0.62	0.765	775	0.00	-0.51	0.52	0.997
Triglycerides in medium VLDL (mmol/l)	1066	-0.01	-0.52	0.50	0.971	985	0.03	-0.52	0.57	0.925	775	-0.02	-0.55	0.51	0.950
Concentration of small VLDL particles (mol/l)	1066	0.05	-0.42	0.51	0.835	985	0.08	-0.42	0.58	0.761	775	-0.04	-0.50	0.42	0.855
Total lipids in small VLDL (mmol/l)	1066	0.03	-0.42	0.47	0.905	985	0.05	-0.43	0.53	0.843	775	-0.09	-0.53	0.34	0.673
Phospholipids in small VLDL (mmol/l)	1066	0.04	-0.39	0.46	0.869	985	0.05	-0.41	0.51	0.828	775	-0.11	-0.52	0.31	0.616
Total cholesterol in small VLDL (mmol/l)	1066	0.01	-0.41	0.43	0.950	985	0.03	-0.44	0.49	0.914	775	-0.17	-0.57	0.22	0.386
Cholesterol esters in small VLDL (mmol/l)	1066	-0.01	-0.43	0.41	0.975	985	0.00	-0.46	0.46	0.993	775	-0.19	-0.58	0.21	0.350
Free cholesterol in small VLDL (mmol/l)	1066	0.05	-0.37	0.48	0.802	985	0.07	-0.39	0.54	0.765	775	-0.12	-0.53	0.30	0.582
Triglycerides in small VLDL (mmol/l)	1066	0.03	-0.45	0.52	0.896	985	0.06	-0.45	0.58	0.811	775	-0.03	-0.52	0.47	0.918
Concentration of very small VLDL particles (mol/l)	1066	0.11	-0.28	0.50	0.582	985	0.11	-0.32	0.54	0.608	775	-0.17	-0.54	0.20	0.361
Total lipids in very small VLDL (mmol/l)	1066	0.03	-0.37	0.43	0.872	985	0.02	-0.42	0.47	0.913	775	-0.24	-0.64	0.15	0.229
Phospholipids in very small VLDL (mmol/l)	1066	0.15	-0.24	0.54	0.449	985	0.13	-0.29	0.56	0.540	775	-0.14	-0.49	0.21	0.432
Total cholesterol in very small VLDL (mmol/l)	1066	-0.10	-0.53	0.33	0.638	985	-0.12	-0.59	0.36	0.627	775	-0.28	-0.74	0.17	0.222
Cholesterol esters in very small VLDL (mmol/l)	1066	-0.11	-0.54	0.32	0.629	985	-0.11	-0.58	0.36	0.651	775	-0.27	-0.72	0.18	0.235
Free cholesterol in very small VLDL (mmol/l)	1066	-0.08	-0.49	0.32	0.682	985	-0.12	-0.57	0.33	0.589	775	-0.28	-0.74	0.17	0.219
Triglycerides in very small VLDL (mmol/l)	1066	0.17	-0.25	0.58	0.432	985	0.19	-0.27	0.64	0.415	775	0.03	-0.39	0.45	0.896
Concentration of IDL particles (mol/l)	1066	0.26	-0.15	0.68	0.218	985	0.26	-0.20	0.71	0.268	775	0.00	-0.35	0.35	0.988
Total lipids in IDL (mmol/l)	1066	0.21	-0.20	0.61	0.319	985	0.19	-0.25	0.63	0.394	775	-0.08	-0.43	0.26	0.630
Phospholipids in IDL (mmol/l)	1066	0.27	-0.15	0.68	0.208	985	0.25	-0.20	0.70	0.272	775	0.00	-0.34	0.35	0.978
Total cholesterol in IDL (mmol/l)	1066	0.16	-0.25	0.57	0.450	985	0.14	-0.31	0.59	0.537	775	-0.13	-0.49	0.22	0.466
Cholesterol esters in IDL (mmol/l)	1066	0.13	-0.28	0.54	0.534	985	0.12	-0.33	0.57	0.612	775	-0.16	-0.52	0.21	0.397
Free cholesterol in IDL (mmol/l)	1066	0.22	-0.20	0.63	0.300	985	0.19	-0.25	0.64	0.395	775	-0.06	-0.41	0.29	0.741
Triglycerides in IDL (mmol/l)	1066	0.26	-0.10	0.63	0.157	985	0.27	-0.13	0.68	0.185	775	0.11	-0.24	0.46	0.532
Concentration of large LDL particles (mol/l)	1066	0.30	-0.12	0.72	0.165	985	0.28	-0.17	0.73	0.228	775	0.05	-0.30	0.41	0.763
Total lipids in large LDL (mmol/l)	1066	0.26	-0.15	0.66	0.218	985	0.24	-0.20	0.68	0.292	775	-0.02	-0.36	0.32	0.906

S8 Table One-sample MR estimates of associations of age at voice breaking (per year later) with adiposity and cardiometabolic traits at age 18y among males in ALSPAC, using a refined GRS of 115 SNPs for age at menarche/voice breaking

	Unadj.					*Adj. for measured BMI at age 8y*					*Adj. for measured outcome value at age 8y*				
**Standardised outcome at age 18y**	**N**	**Beta (2SLS)**	**LCL**	**UCL**	**P-value**	**N**	**Beta (2SLS)**	**LCL**	**UCL**	**P-value**	**N**	**Beta (2SLS)**	**LCL**	**UCL**	**P-value**
Phospholipids in large LDL (mmol/l)	1066	0.25	-0.16	0.66	0.236	985	0.23	-0.22	0.67	0.314	775	-0.02	-0.37	0.32	0.888
Total cholesterol in large LDL (mmol/l)	1066	0.24	-0.17	0.65	0.251	985	0.22	-0.23	0.66	0.334	775	-0.04	-0.38	0.30	0.810
Cholesterol esters in large LDL (mmol/l)	1066	0.24	-0.17	0.65	0.251	985	0.22	-0.22	0.67	0.330	775	-0.04	-0.38	0.30	0.823
Free cholesterol in large LDL (mmol/l)	1066	0.24	-0.17	0.65	0.258	985	0.21	-0.23	0.65	0.353	775	-0.05	-0.39	0.30	0.795
Triglycerides in large LDL (mmol/l)	1066	0.31	-0.07	0.68	0.106	985	0.32	-0.10	0.73	0.132	775	0.16	-0.18	0.51	0.353
Concentration of medium LDL particles (mol/l)	1066	0.32	-0.10	0.75	0.138	985	0.31	-0.16	0.77	0.195	775	0.10	-0.27	0.47	0.608
Total lipids in medium LDL (mmol/l)	1066	0.27	-0.14	0.68	0.196	985	0.25	-0.19	0.69	0.272	775	0.01	-0.34	0.36	0.955
Phospholipids in medium LDL (mmol/l)	1066	0.19	-0.20	0.59	0.338	985	0.16	-0.26	0.59	0.450	775	-0.09	-0.42	0.24	0.594
Total cholesterol in medium LDL (mmol/l)	1066	0.27	-0.15	0.68	0.213	985	0.24	-0.21	0.69	0.297	775	0.00	-0.35	0.36	0.995
Cholesterol esters in medium LDL (mmol/l)	1066	0.28	-0.14	0.70	0.197	985	0.25	-0.20	0.71	0.272	775	0.03	-0.33	0.39	0.889
Free cholesterol in medium LDL (mmol/l)	1066	0.21	-0.20	0.62	0.309	985	0.18	-0.27	0.62	0.436	775	-0.10	-0.45	0.25	0.572
Triglycerides in medium LDL (mmol/l)	1066	0.38	-0.02	0.79	0.063	985	0.40	-0.05	0.85	0.083	775	0.26	-0.13	0.64	0.190
Concentration of small LDL particles (mol/l)	1066	0.31	-0.11	0.72	0.154	985	0.27	-0.17	0.72	0.231	775	0.07	-0.30	0.43	0.713
Total lipids in small LDL (mmol/l)	1066	0.27	-0.15	0.68	0.205	985	0.23	-0.21	0.67	0.297	775	0.00	-0.35	0.35	0.984
Phospholipids in small LDL (mmol/l)	1066	0.20	-0.19	0.60	0.312	985	0.16	-0.26	0.59	0.452	775	-0.08	-0.41	0.26	0.664
Total cholesterol in small LDL (mmol/l)	1066	0.26	-0.16	0.68	0.222	985	0.23	-0.22	0.68	0.321	775	0.00	-0.36	0.35	0.993
Cholesterol esters in small LDL (mmol/l)	1066	0.30	-0.13	0.72	0.176	985	0.27	-0.19	0.73	0.252	775	0.06	-0.31	0.42	0.764
Free cholesterol in small LDL (mmol/l)	1066	0.10	-0.30	0.49	0.633	985	0.04	-0.39	0.46	0.862	775	-0.22	-0.60	0.16	0.249
Triglycerides in small LDL (mmol/l)	1066	0.36	-0.07	0.78	0.101	985	0.38	-0.09	0.84	0.115	775	0.23	-0.19	0.64	0.281
Concentration of very large HDL particles (mol/l)	1066	0.29	-0.14	0.71	0.191	985	0.29	-0.18	0.76	0.225	775	0.18	-0.16	0.52	0.302
Total lipids in very large HDL (mmol/l)	1066	0.27	-0.16	0.70	0.216	985	0.27	-0.20	0.74	0.260	775	0.16	-0.19	0.51	0.368
Phospholipids in very large HDL (mmol/l)	1066	0.30	-0.13	0.73	0.168	985	0.31	-0.16	0.78	0.198	775	0.20	-0.13	0.52	0.239
Total cholesterol in very large HDL (mmol/l)	1066	0.21	-0.22	0.65	0.341	985	0.19	-0.28	0.66	0.422	775	0.11	-0.28	0.50	0.586
Cholesterol esters in very large HDL (mmol/l)	1066	0.18	-0.26	0.62	0.414	985	0.16	-0.31	0.63	0.509	775	0.09	-0.32	0.51	0.652
Free cholesterol in very large HDL (mmol/l)	1066	0.27	-0.16	0.71	0.213	985	0.27	-0.20	0.74	0.261	775	0.14	-0.21	0.48	0.432
Triglycerides in very large HDL (mmol/l)	1066	0.21	-0.25	0.68	0.368	985	0.29	-0.24	0.82	0.283	775	0.11	-0.32	0.53	0.629
Concentration of large HDL particles (mol/l)	1066	0.20	-0.18	0.59	0.304	985	0.18	-0.24	0.59	0.405	775	0.12	-0.18	0.41	0.443
Total lipids in large HDL (mmol/l)	1066	0.19	-0.20	0.58	0.329	985	0.16	-0.25	0.58	0.444	775	0.09	-0.21	0.39	0.568
Phospholipids in large HDL (mmol/l)	1066	0.21	-0.17	0.60	0.281	985	0.18	-0.23	0.60	0.390	775	0.11	-0.19	0.42	0.468
Total cholesterol in large HDL (mmol/l)	1066	0.18	-0.22	0.57	0.378	985	0.14	-0.27	0.56	0.499	775	0.07	-0.23	0.37	0.647
Cholesterol esters in large HDL (mmol/l)	1066	0.17	-0.22	0.57	0.382	985	0.14	-0.27	0.56	0.499	775	0.08	-0.22	0.38	0.612
Free cholesterol in large HDL (mmol/l)	1066	0.18	-0.21	0.57	0.366	985	0.14	-0.27	0.56	0.502	775	0.04	-0.26	0.34	0.799
Triglycerides in large HDL (mmol/l)	1066	0.13	-0.27	0.54	0.519	985	0.15	-0.30	0.59	0.521	775	-0.02	-0.41	0.38	0.931
Concentration of medium HDL particles (mol/l)	1066	0.05	-0.28	0.37	0.782	985	0.00	-0.35	0.34	0.977	775	-0.01	-0.32	0.30	0.945
Total lipids in medium HDL (mmol/l)	1066	0.07	-0.27	0.41	0.687	985	0.01	-0.35	0.37	0.948	775	0.02	-0.31	0.35	0.903
Phospholipids in medium HDL (mmol/l)	1066	0.06	-0.26	0.38	0.720	985	0.01	-0.33	0.35	0.956	775	0.01	-0.30	0.32	0.958
Total cholesterol in medium HDL (mmol/l)	1066	0.07	-0.30	0.44	0.704	985	0.00	-0.40	0.40	0.997	775	0.03	-0.33	0.40	0.852
Cholesterol esters in medium HDL (mmol/l)	1066	0.08	-0.31	0.47	0.685	985	0.01	-0.41	0.43	0.962	775	0.06	-0.32	0.44	0.757
Free cholesterol in medium HDL (mmol/l)	1066	0.02	-0.35	0.39	0.914	985	-0.06	-0.46	0.33	0.764	775	-0.10	-0.48	0.29	0.619
Triglycerides in medium HDL (mmol/l)	1066	0.08	-0.31	0.47	0.682	985	0.12	-0.29	0.54	0.564	775	0.09	-0.30	0.48	0.656
Concentration of small HDL particles (mol/l)	1066	-0.02	-0.35	0.31	0.914	985	-0.07	-0.42	0.28	0.691	775	-0.03	-0.36	0.30	0.865
Total lipids in small HDL (mmol/l)	1066	0.02	-0.29	0.33	0.918	985	-0.04	-0.37	0.28	0.793	775	-0.05	-0.36	0.26	0.760
Phospholipids in small HDL (mmol/l)	1066	-0.05	-0.41	0.31	0.797	985	-0.10	-0.49	0.28	0.596	775	-0.04	-0.40	0.31	0.813
Total cholesterol in small HDL (mmol/l)	1066	0.07	-0.24	0.37	0.672	985	0.01	-0.32	0.33	0.974	775	-0.05	-0.35	0.25	0.728
Cholesterol esters in small HDL (mmol/l)	1066	0.08	-0.24	0.39	0.630	985	0.03	-0.31	0.36	0.873	775	-0.05	-0.36	0.26	0.755
Free cholesterol in small HDL (mmol/l)	1066	0.00	-0.36	0.35	0.984	985	-0.08	-0.46	0.30	0.682	775	-0.05	-0.39	0.29	0.773
Triglycerides in small HDL (mmol/l)	1066	0.09	-0.32	0.50	0.668	985	0.13	-0.31	0.58	0.555	775	0.07	-0.34	0.48	0.741
Phospholipids to total lipids ratio in chylomicrons and extremely large VLDL (%)	1066	-0.11	-0.55	0.34	0.639	985	-0.14	-0.64	0.36	0.585	775	-0.07	-0.61	0.46	0.785
Total cholesterol to total lipids ratio in chylomicrons and extremely large VLDL (%)	1066	-0.15	-0.58	0.28	0.492	985	-0.11	-0.57	0.36	0.653	775	-0.11	-0.56	0.34	0.637
Cholesterol esters to total lipids ratio in chylomicrons and extremely large VLDL (%)	1066	-0.19	-0.61	0.23	0.372	985	-0.18	-0.64	0.28	0.442	775	-0.24	-0.70	0.23	0.318
Free cholesterol to total lipids ratio in chylomicrons and extremely large VLDL (%)	1066	0.08	-0.39	0.54	0.747	985	0.20	-0.32	0.72	0.448	775	0.33	-0.24	0.90	0.257
Triglycerides to total lipids ratio in chylomicrons and extremely large VLDL (%)	1066	0.23	-0.18	0.64	0.267	985	0.21	-0.24	0.66	0.356	775	0.25	-0.22	0.72	0.295
Phospholipids to total lipids ratio in very large VLDL (%)	1066	-0.17	-0.63	0.28	0.457	985	-0.13	-0.63	0.36	0.598	775	-0.07	-0.57	0.42	0.773
Total cholesterol to total lipids ratio in very large VLDL (%)	1066	0.09	-0.24	0.41	0.592	985	0.07	-0.29	0.42	0.705	775	0.10	-0.27	0.48	0.583
Cholesterol esters to total lipids ratio in very large VLDL (%)	1066	0.09	-0.19	0.36	0.545	985	0.08	-0.23	0.38	0.625	775	0.08	-0.25	0.40	0.641
Free cholesterol to total lipids ratio in very large VLDL (%)	1066	0.00	-0.25	0.24	0.978	985	-0.03	-0.30	0.24	0.824	775	0.04	-0.24	0.31	0.797
Triglycerides to total lipids ratio in very large VLDL (%)	1066	-0.06	-0.43	0.32	0.770	985	-0.04	-0.45	0.36	0.830	775	-0.10	-0.52	0.32	0.636

S8 Table One-sample MR estimates of associations of age at voice breaking (per year later) with adiposity and cardiometabolic traits at age 18y among males in ALSPAC, using a refined GRS of 115 SNPs for age at menarche/voice breaking

	Unadj.					*Adj. for measured BMI at age 8y*					*Adj. for measured outcome value at age 8y*				
**Standardised outcome at age 18y**	**N**	**Beta (2SLS)**	**LCL**	**UCL**	**P-value**	**N**	**Beta (2SLS)**	**LCL**	**UCL**	**P-value**	**N**	**Beta (2SLS)**	**LCL**	**UCL**	**P-value**
Phospholipids to total lipids ratio in large VLDL (%)	1066	0.04	-0.38	0.45	0.859	985	0.13	-0.33	0.58	0.584	775	0.08	-0.37	0.53	0.725
Total cholesterol to total lipids ratio in large VLDL (%)	1066	0.02	-0.40	0.43	0.943	985	0.11	-0.35	0.57	0.643	775	0.06	-0.39	0.50	0.801
Cholesterol esters to total lipids ratio in large VLDL (%)	1066	0.09	-0.25	0.44	0.592	985	0.16	-0.23	0.54	0.419	775	0.10	-0.27	0.47	0.601
Free cholesterol to total lipids ratio in large VLDL (%)	1066	-0.03	-0.21	0.14	0.703	985	0.00	-0.19	0.19	0.996	775	0.00	-0.18	0.19	0.962
Triglycerides to total lipids ratio in large VLDL (%)	1066	0.01	-0.42	0.44	0.969	985	-0.08	-0.55	0.38	0.723	775	-0.06	-0.52	0.40	0.793
Phospholipids to total lipds ratio in medium VLDL (%)	1066	0.11	-0.22	0.44	0.531	985	0.05	-0.31	0.41	0.778	775	-0.05	-0.37	0.28	0.774
Total cholesterol to total lipids ratio in medium VLDL (%)	1066	0.05	-0.38	0.48	0.823	985	0.11	-0.37	0.58	0.665	775	-0.03	-0.46	0.40	0.900
Cholesterol esters to total lipids ratio in medium VLDL (%)	1066	0.03	-0.37	0.44	0.868	985	0.08	-0.36	0.53	0.714	775	-0.05	-0.46	0.35	0.805
Free cholesterol to total lipids ratio in medium VLDL (%)	1066	0.06	-0.42	0.55	0.802	985	0.11	-0.43	0.65	0.689	775	0.06	-0.48	0.60	0.828
Triglycerides to total lipids ratio in medium VLDL (%)	1066	-0.07	-0.48	0.34	0.734	985	-0.11	-0.56	0.34	0.627	775	0.04	-0.37	0.44	0.859
Phospholipids to total lipds ratio in small VLDL (%)	1066	0.11	-0.34	0.56	0.628	985	0.08	-0.41	0.57	0.751	775	0.08	-0.38	0.53	0.743
Total cholesterol to total lipids ratio in small VLDL (%)	1066	-0.02	-0.48	0.43	0.922	985	-0.06	-0.56	0.43	0.799	775	-0.11	-0.58	0.35	0.639
Cholesterol esters to total lipids ratio in small VLDL (%)	1066	-0.05	-0.51	0.41	0.831	985	-0.09	-0.58	0.41	0.736	775	-0.10	-0.57	0.36	0.657
Free cholesterol to total lipids ratio in small VLDL (%)	1066	0.20	-0.17	0.57	0.297	985	0.15	-0.24	0.55	0.450	775	-0.06	-0.40	0.28	0.720
Triglycerides to total lipids ratio in small VLDL (%)	1066	-0.01	-0.48	0.46	0.957	985	0.04	-0.47	0.55	0.877	775	0.08	-0.40	0.56	0.742
Phospholipids to total lipds ratio in very small VLDL (%)	1066	0.34	-0.11	0.79	0.141	985	0.31	-0.17	0.80	0.205	775	0.12	-0.26	0.50	0.538
Total cholesterol to total lipids ratio in very small VLDL (%)	1066	-0.37	-0.84	0.11	0.130	985	-0.38	-0.89	0.13	0.146	775	-0.25	-0.72	0.21	0.289
Cholesterol esters to total lipids ratio in very small VLDL (%)	1066	-0.30	-0.73	0.13	0.167	985	-0.29	-0.74	0.17	0.212	775	-0.16	-0.57	0.25	0.447
Free cholesterol to total lipids ratio in very small VLDL (%)	1066	-0.34	-0.83	0.16	0.184	985	-0.42	-0.98	0.14	0.142	775	-0.41	-0.97	0.15	0.153
Triglycerides to total lipids ratio in very small VLDL (%)	1066	0.22	-0.27	0.70	0.380	985	0.25	-0.27	0.77	0.353	775	0.21	-0.28	0.71	0.400
Phospholipids to total lipds ratio in IDL (%)	1066	0.35	-0.23	0.94	0.235	985	0.36	-0.29	1.02	0.279	775	0.35	-0.30	1.01	0.289
Total cholesterol to total lipids ratio in IDL (%)	1066	-0.21	-0.68	0.26	0.388	985	-0.21	-0.72	0.31	0.435	775	-0.22	-0.70	0.27	0.382
Cholesterol esters to total lipids ratio in IDL (%)	1066	-0.25	-0.71	0.21	0.294	985	-0.23	-0.74	0.28	0.374	775	-0.20	-0.69	0.28	0.408
Free cholesterol to total lipids ratio in IDL (%)	1066	0.07	-0.51	0.66	0.802	985	0.04	-0.59	0.67	0.908	775	-0.06	-0.65	0.54	0.856
Triglycerides to total lipids ratio in IDL (%)	1066	0.10	-0.35	0.55	0.672	985	0.09	-0.40	0.59	0.711	775	0.10	-0.34	0.54	0.662
Phospholipids to total lipds ratio in large LDL (%)	1066	-0.16	-0.66	0.33	0.519	985	-0.14	-0.68	0.41	0.623	775	0.17	-0.30	0.64	0.471
Total cholesterol to total lipids ratio in large LDL (%)	1066	0.05	-0.47	0.57	0.857	985	0.03	-0.54	0.60	0.921	775	-0.19	-0.65	0.27	0.419
Cholesterol esters to total lipids ratio in large LDL (%)	1066	0.15	-0.38	0.68	0.576	985	0.14	-0.45	0.72	0.647	775	-0.14	-0.56	0.27	0.490
Free cholesterol to total lipids ratio in large LDL (%)	1066	-0.33	-0.92	0.26	0.271	985	-0.33	-0.96	0.30	0.303	775	-0.19	-0.75	0.37	0.500
Triglycerides to total lipids ratio in large LDL (%)	1066	0.11	-0.37	0.59	0.649	985	0.11	-0.41	0.64	0.676	775	0.11	-0.35	0.57	0.630
Phospholipids to total lipds ratio in medium LDL (%)	1066	-0.28	-0.86	0.30	0.349	985	-0.27	-0.91	0.38	0.418	775	-0.09	-0.62	0.43	0.722
Total cholesterol to total lipids ratio in medium LDL (%)	1066	0.11	-0.46	0.69	0.705	985	0.10	-0.54	0.74	0.765	775	-0.05	-0.56	0.45	0.831
Cholesterol esters to total lipids ratio in medium LDL (%)	1066	0.18	-0.37	0.73	0.524	985	0.16	-0.45	0.76	0.606	775	-0.03	-0.50	0.44	0.910
Free cholesterol to total lipids ratio in medium LDL (%)	1066	-0.24	-0.74	0.26	0.341	985	-0.22	-0.77	0.33	0.428	775	-0.05	-0.52	0.43	0.853
Triglycerides to total lipids ratio in medium LDL (%)	1066	0.30	-0.15	0.75	0.191	985	0.31	-0.19	0.81	0.225	775	0.25	-0.20	0.70	0.272
Phospholipids to total lipds ratio in small LDL (%)	1066	-0.24	-0.78	0.29	0.372	985	-0.22	-0.81	0.36	0.454	775	-0.04	-0.51	0.43	0.865
Total cholesterol to total lipids ratio in small LDL (%)	1066	0.11	-0.43	0.65	0.694	985	0.08	-0.52	0.68	0.790	775	-0.06	-0.55	0.43	0.800
Cholesterol esters to total lipids ratio in small LDL (%)	1066	0.25	-0.30	0.79	0.376	985	0.23	-0.38	0.83	0.462	775	0.06	-0.41	0.54	0.799
Free cholesterol to total lipids ratio in small LDL (%)	1066	-0.43	-0.99	0.13	0.129	985	-0.43	-1.04	0.18	0.170	775	-0.27	-0.79	0.25	0.303
Triglycerides to total lipids ratio in small LDL (%)	1066	0.34	-0.19	0.87	0.207	985	0.37	-0.21	0.95	0.208	775	0.30	-0.22	0.82	0.263
Phospholipids to total lipds ratio in very large HDL (%)	1066	0.30	-0.20	0.79	0.239	985	0.33	-0.22	0.89	0.241	775	0.33	-0.15	0.81	0.182
Total cholesterol to total lipids ratio in very large HDL (%)	1066	-0.32	-0.81	0.18	0.207	985	-0.37	-0.94	0.19	0.196	775	-0.33	-0.81	0.15	0.176
Cholesterol esters to total lipids ratio in very large HDL (%)	1066	-0.32	-0.82	0.18	0.207	985	-0.37	-0.94	0.20	0.200	775	-0.31	-0.78	0.16	0.200
Free cholesterol to total lipids ratio in very large HDL (%)	1066	0.13	-0.43	0.69	0.646	985	0.11	-0.51	0.73	0.724	775	-0.13	-0.73	0.48	0.682
Triglycerides to total lipids ratio in very large HDL (%)	1066	0.05	-0.44	0.55	0.830	985	0.13	-0.41	0.68	0.628	775	-0.01	-0.50	0.49	0.983
Phospholipids to total lipds ratio in large HDL (%)	1066	0.13	-0.28	0.53	0.535	985	0.14	-0.29	0.56	0.532	775	0.14	-0.27	0.55	0.504
Total cholesterol to total lipids ratio in large HDL (%)	1066	-0.07	-0.49	0.35	0.734	985	-0.10	-0.55	0.34	0.649	775	-0.08	-0.48	0.33	0.713
Cholesterol esters to total lipids ratio in large HDL (%)	1066	-0.08	-0.51	0.35	0.724	985	-0.10	-0.56	0.36	0.669	775	-0.01	-0.43	0.41	0.957
Free cholesterol to total lipids ratio in large HDL (%)	1066	-0.04	-0.46	0.38	0.862	985	-0.09	-0.53	0.35	0.687	775	-0.27	-0.73	0.20	0.260
Triglycerides to total lipids ratio in large HDL (%)	1066	-0.02	-0.48	0.44	0.936	985	0.05	-0.45	0.55	0.840	775	-0.07	-0.50	0.36	0.755
Phospholipids to total lipds ratio in medium HDL (%)	1066	-0.02	-0.45	0.41	0.929	985	0.00	-0.48	0.47	0.989	775	-0.03	-0.49	0.43	0.898
Total cholesterol to total lipids ratio in medium HDL (%)	1066	-0.01	-0.43	0.40	0.944	985	-0.07	-0.54	0.40	0.765	775	-0.02	-0.47	0.43	0.937
Cholesterol esters to total lipids ratio in medium HDL (%)	1066	0.03	-0.45	0.51	0.890	985	0.00	-0.54	0.53	0.987	775	0.08	-0.46	0.62	0.765
Free cholesterol to total lipids ratio in medium HDL (%)	1066	-0.29	-1.33	0.75	0.582	985	-0.41	-1.59	0.77	0.498	775	-0.56	-1.85	0.73	0.393
Triglycerides to total lipids ratio in medium HDL (%)	1066	0.08	-0.36	0.53	0.706	985	0.18	-0.31	0.66	0.477	775	0.13	-0.31	0.57	0.564
Phospholipids to total lipds ratio in small HDL (%)	1066	-0.15	-0.53	0.23	0.449	985	-0.12	-0.53	0.28	0.552	775	0.03	-0.34	0.39	0.887
Total cholesterol to total lipids ratio in small HDL (%)	1066	0.11	-0.29	0.50	0.602	985	0.06	-0.37	0.48	0.795	775	-0.06	-0.45	0.32	0.752

S8 Table One-sample MR estimates of associations of age at voice breaking (per year later) with adiposity and cardiometabolic traits at age 18y among males in ALSPAC, using a refined GRS of 115 SNPs for age at menarche/voice breaking

	Unadj.					*Adj. for measured BMI at age 8y*					*Adj. for measured outcome value at age 8y*				
**Standardised outcome at age 18y**	**N**	**Beta (2SLS)**	**LCL**	**UCL**	**P-value**	**N**	**Beta (2SLS)**	**LCL**	**UCL**	**P-value**	**N**	**Beta (2SLS)**	**LCL**	**UCL**	**P-value**
Cholesterol esters to total lipids ratio in small HDL (%)	1066	0.11	-0.29	0.50	0.601	985	0.07	-0.36	0.49	0.752	775	-0.05	-0.44	0.34	0.801
Free cholesterol to total lipids ratio in small HDL (%)	1066	-0.07	-0.54	0.39	0.752	985	-0.13	-0.65	0.38	0.615	775	-0.05	-0.54	0.44	0.845
Triglycerides to total lipids ratio in small HDL (%)	1066	0.12	-0.36	0.60	0.624	985	0.22	-0.32	0.75	0.427	775	0.14	-0.35	0.63	0.571
Mean diameter for VLDL particles (nm)	1066	-0.07	-0.55	0.41	0.774	985	0.00	-0.52	0.51	0.986	775	0.05	-0.43	0.54	0.830
Mean diameter for LDL particles (nm)	1066	-0.26	-0.76	0.24	0.306	985	-0.16	-0.68	0.36	0.554	775	-0.10	-0.60	0.39	0.677
Mean diameter for HDL particles (nm)	1066	0.25	-0.19	0.69	0.260	985	0.24	-0.23	0.71	0.318	775	0.13	-0.20	0.47	0.428
Serum total cholesterol (mmol/l)	1066	0.22	-0.17	0.62	0.265	985	0.20	-0.23	0.62	0.364	775	-0.05	-0.36	0.26	0.737
Total cholesterol in VLDL (mmol/l)	1066	0.00	-0.45	0.45	0.988	985	0.03	-0.46	0.52	0.900	775	-0.14	-0.57	0.29	0.528
Remnant cholesterol (non-HDL, non-LDL -cholesterol) (mmol/l)	1066	0.08	-0.35	0.50	0.725	985	0.08	-0.38	0.55	0.722	775	-0.17	-0.56	0.22	0.393
Total cholesterol in LDL (mmol/l)	1066	0.25	-0.16	0.67	0.232	985	0.23	-0.22	0.67	0.319	775	-0.02	-0.37	0.33	0.905
Total cholesterol in HDL (mmol/l)	1066	0.17	-0.20	0.55	0.366	985	0.12	-0.27	0.51	0.549	775	0.07	-0.21	0.35	0.637
Total cholesterol in HDL2 (mmol/l)	1066	0.15	-0.24	0.53	0.449	985	0.09	-0.32	0.49	0.667	775	0.07	-0.24	0.38	0.653
Total cholesterol in HDL3 (mmol/l)	1066	0.21	-0.16	0.57	0.271	985	0.17	-0.22	0.55	0.391	775	0.06	-0.20	0.32	0.651
Esterified cholesterol (mmol/l)	1062	0.21	-0.18	0.60	0.298	981	0.18	-0.24	0.61	0.398	771	-0.05	-0.37	0.28	0.783
Free cholesterol (mmol/l)	1061	0.24	-0.15	0.63	0.223	980	0.21	-0.20	0.62	0.318	769	-0.06	-0.36	0.24	0.690
Serum total triglycerides (mmol/l)	1066	0.11	-0.39	0.61	0.663	985	0.15	-0.38	0.69	0.579	775	0.05	-0.46	0.57	0.841
Triglycerides in VLDL (mmol/l)	1066	0.04	-0.47	0.55	0.882	985	0.08	-0.47	0.63	0.773	775	0.01	-0.52	0.55	0.958
Triglycerides in LDL (mmol/l)	1066	0.34	-0.05	0.74	0.086	985	0.36	-0.08	0.79	0.107	775	0.21	-0.16	0.57	0.270
Triglycerides in HDL (mmol/l)	1066	0.14	-0.27	0.55	0.510	985	0.19	-0.26	0.64	0.406	775	0.08	-0.33	0.48	0.709
Diacylglycerol (mmol/l)	1044	0.19	-0.21	0.59	0.353	969	0.27	-0.22	0.77	0.281	744	0.15	-0.29	0.59	0.512
Ratio of diacylglycerol to triglycerides	1044	0.22	-0.14	0.58	0.236	969	0.27	-0.17	0.72	0.228	744	0.22	-0.18	0.62	0.284
Total phosphoglycerides (mmol/l)	1061	0.29	-0.09	0.67	0.136	980	0.32	-0.10	0.74	0.138	769	0.12	-0.19	0.44	0.445
Ratio of triglycerides to phosphoglycerides	1061	-0.12	-0.64	0.40	0.656	980	-0.07	-0.63	0.49	0.808	769	-0.10	-0.64	0.44	0.718
Phosphatidylcholine and other cholines (mmol/l)	1056	0.22	-0.15	0.58	0.243	975	0.23	-0.16	0.63	0.251	763	0.13	-0.20	0.46	0.450
Total cholines (mmol/l)	1062	0.20	-0.16	0.56	0.284	981	0.22	-0.18	0.61	0.283	771	-0.01	-0.32	0.31	0.968
Apolipoprotein A-I (g/l)	1066	0.20	-0.15	0.56	0.266	985	0.15	-0.22	0.52	0.434	775	0.05	-0.20	0.31	0.697
Apolipoprotein B (g/l)	1066	0.15	-0.30	0.59	0.518	985	0.16	-0.32	0.65	0.508	775	-0.06	-0.47	0.35	0.786
Ratio of apolipoprotein B to apolipoprotein A-I	1066	0.07	-0.39	0.53	0.771	985	0.12	-0.39	0.62	0.651	775	-0.08	-0.50	0.35	0.729
Total fatty acids (mmol/l)	1062	0.13	-0.29	0.54	0.547	981	0.16	-0.29	0.62	0.478	771	-0.04	-0.44	0.36	0.847
Estimated description of fatty acid chain length, not actual carbon number	1062	0.00	-0.47	0.48	0.988	981	0.05	-0.46	0.57	0.838	770	0.18	-0.32	0.67	0.482
Estimated degree of unsaturation	1061	0.13	-0.36	0.61	0.610	980	0.11	-0.41	0.63	0.680	770	0.22	-0.32	0.75	0.427
22:6, docosahexaenoic acid (mmol/l)	1062	-0.13	-0.56	0.31	0.572	981	-0.16	-0.65	0.33	0.523	771	-0.05	-0.49	0.39	0.831
18:2, linoleic acid (mmol/l)	1062	0.20	-0.19	0.60	0.311	981	0.25	-0.19	0.69	0.268	771	0.12	-0.24	0.48	0.529
Conjugated linoleic acid (mmol/l)	1061	0.13	-0.28	0.54	0.527	980	0.13	-0.33	0.58	0.586	769	0.11	-0.34	0.56	0.642
Omega-3 fatty acids (mmol/l)	1062	0.02	-0.51	0.54	0.953	981	-0.06	-0.65	0.52	0.831	771	-0.12	-0.69	0.45	0.680
Omega-6 fatty acids (mmol/l)	1062	0.21	-0.18	0.61	0.287	981	0.25	-0.19	0.69	0.261	771	0.09	-0.26	0.44	0.610
Polyunsaturated fatty acids (mmol/l)	1062	0.19	-0.21	0.60	0.344	981	0.22	-0.23	0.66	0.337	771	0.06	-0.30	0.43	0.730
Monounsaturated fatty acids; 16:1, 18:1 (mmol/l)	1062	0.17	-0.29	0.63	0.464	981	0.22	-0.28	0.72	0.382	770	-0.02	-0.48	0.43	0.918
Saturated fatty acids (mmol/l)	1062	0.00	-0.42	0.42	0.989	981	0.03	-0.43	0.50	0.890	770	-0.12	-0.54	0.30	0.581
Ratio of 22:6 docosahexaenoic acid to total fatty acids (%)	1062	-0.32	-0.84	0.20	0.223	981	-0.40	-1.00	0.20	0.188	771	-0.13	-0.61	0.35	0.590
Ratio of 18:2 linoleic acid to total fatty acids (%)	1062	0.27	-0.24	0.77	0.300	981	0.31	-0.26	0.87	0.284	771	0.32	-0.23	0.87	0.253
Ratio of conjugated linoleic acid to total fatty acids (%)	1061	0.08	-0.33	0.50	0.698	980	0.06	-0.41	0.52	0.811	769	0.07	-0.38	0.52	0.761
Ratio of omega-3 fatty acids to total fatty acids (%)	1062	-0.16	-0.71	0.39	0.560	981	-0.33	-0.97	0.31	0.313	771	-0.20	-0.81	0.41	0.523
Ratio of omega-6 fatty acids to total fatty acids (%)	1062	0.28	-0.25	0.81	0.302	981	0.30	-0.29	0.88	0.320	771	0.31	-0.25	0.86	0.277
Ratio of polyunsaturated fatty acids to total fatty acids (%)	1062	0.23	-0.30	0.75	0.397	981	0.20	-0.36	0.77	0.482	771	0.25	-0.30	0.79	0.377
Ratio of monounsaturated fatty acids to total fatty acids (%)	1062	0.10	-0.39	0.59	0.683	981	0.15	-0.37	0.67	0.567	770	-0.02	-0.54	0.50	0.946
Ratio of saturated fatty acids to total fatty acids (%)	1062	-0.41	-0.97	0.14	0.145	981	-0.46	-1.07	0.16	0.147	770	-0.33	-0.85	0.19	0.215
Glucose (mmol/l)	1066	-0.31	-1.20	0.58	0.495	985	-0.37	-1.39	0.66	0.480	770	0.01	-0.33	0.35	0.942
Lactate (mmol/l)	1066	0.06	-0.39	0.52	0.783	985	0.02	-0.47	0.51	0.940	776	0.19	-0.33	0.70	0.478
Pyruvate (mmol/l)	1066	0.06	-0.41	0.54	0.790	985	0.05	-0.46	0.55	0.854	775	0.07	-0.44	0.57	0.797
Citrate (mmol/l)	1066	0.36	-0.13	0.85	0.149	985	0.43	-0.14	1.00	0.137	775	0.38	-0.15	0.91	0.162
Alanine (mmol/l)	1066	0.43	-0.07	0.92	0.091	985	0.48	-0.06	1.02	0.082	776	0.44	-0.07	0.94	0.091
Glutamine (mmol/l)	1066	-0.09	-0.45	0.26	0.605	985	-0.11	-0.50	0.27	0.560	775	0.00	-0.33	0.33	0.988
Histidine (mmol/l)	1066	0.04	-0.36	0.44	0.833	985	0.11	-0.33	0.55	0.636	775	0.12	-0.30	0.55	0.569
Isoleucine (mmol/l)	1066	0.33	-0.15	0.82	0.177	985	0.40	-0.13	0.94	0.140	776	0.35	-0.13	0.84	0.153
Leucine (mmol/l)	1066	0.14	-0.27	0.55	0.510	985	0.16	-0.28	0.61	0.471	776	0.19	-0.24	0.62	0.392
Valine (mmol/l)	1066	0.05	-0.38	0.48	0.805	985	0.06	-0.40	0.53	0.785	776	0.10	-0.33	0.53	0.650

S8 Table One-sample MR estimates of associations of age at voice breaking (per year later) with adiposity and cardiometabolic traits at age 18y among males in ALSPAC, using a refined GRS of 115 SNPs for age at menarche/voice breaking

	Unadj.					*Adj. for measured BMI at age 8y*					*Adj. for measured outcome value at age 8y*				
**Standardised outcome at age 18y**	**N**	**Beta (2SLS)**	**LCL**	**UCL**	**P-value**	**N**	**Beta (2SLS)**	**LCL**	**UCL**	**P-value**	**N**	**Beta (2SLS)**	**LCL**	**UCL**	**P-value**
Phenylalanine (mmol/l)	1066	-0.04	-0.49	0.42	0.871	985	-0.01	-0.52	0.49	0.958	773	0.10	-0.39	0.60	0.677
Tyrosine (mmol/l)	1066	0.00	-0.34	0.35	0.988	985	0.07	-0.31	0.45	0.707	772	-0.01	-0.36	0.33	0.937
Acetate (mmol/l)	1065	-0.06	-0.39	0.27	0.729	984	-0.06	-0.44	0.32	0.750	776	0.03	-0.15	0.21	0.755
Acetoacetate (mmol/l)	1066	0.05	-0.49	0.59	0.861	985	0.05	-0.56	0.65	0.884	776	0.22	-0.36	0.79	0.462
3-hydroxybutyrate (mmol/l)	1066	0.13	-0.27	0.54	0.516	985	0.15	-0.30	0.59	0.513	776	0.22	-0.27	0.71	0.371
Creatinine (mmol/l)	1066	-0.41	-0.88	0.06	0.085	985	-0.49	-1.04	0.05	0.074	774	-0.51	-1.01	0.00	0.048
Albumin (signal area)	1066	-0.30	-0.75	0.15	0.187	985	-0.32	-0.81	0.18	0.213	775	-0.18	-0.63	0.28	0.443
Glycoprotein acetyls, mainly a1-acid glycoprotein (mmol/l)	1066	0.05	-0.38	0.47	0.835	985	0.12	-0.36	0.59	0.627	776	0.07	-0.37	0.51	0.755

Complete case sample

	Unadj.					*Adj. for measured BMI at age 8y*					*Adj. for measured outcome value at age 8y*				
**Standardised outcome at age 18y**	**N**	**Beta (2SLS)**	**LCL**	**UCL**	**P-value**	**N**	**Beta (2SLS)**	**LCL**	**UCL**	**P-value**	**N**	**Beta (2SLS)**	**LCL**	**UCL**	**P-value**
Body mass index (kg/m^2)	564	-0.09	-0.47	0.29	0.649	564	-0.04	-0.32	0.23	0.745	564	-0.04	-0.32	0.23	0.745
Fat mass index (kg/m^2)	564	-0.03	-0.37	0.31	0.851	564	0.00	-0.27	0.27	0.994	564	0.04	-0.18	0.26	0.715
Lean mass index (kg/m^2)	564	-0.10	-0.41	0.21	0.532	564	-0.08	-0.36	0.20	0.587	564	-0.11	-0.35	0.12	0.340
Systolic blood pressure (mmHg)	564	-0.29	-0.73	0.14	0.185	564	-0.28	-0.71	0.15	0.199	564	-0.34	-0.76	0.07	0.107
Diastolic blood pressure (mmHg)	564	0.09	-0.36	0.54	0.686	564	0.11	-0.34	0.55	0.641	564	0.04	-0.38	0.45	0.857
Concentration of chylomicrons and extremely large VLDL particles (mol/l)	564	0.15	-0.38	0.67	0.582	564	0.16	-0.36	0.68	0.540	564	0.16	-0.36	0.67	0.549
Total lipids in chylomicrons and extremely large VLDL (mmol/l)	564	0.16	-0.36	0.67	0.553	564	0.17	-0.34	0.69	0.512	564	0.17	-0.35	0.68	0.520
Phospholipids in chylomicrons and extremely large VLDL (mmol/l)	564	0.17	-0.35	0.68	0.524	564	0.18	-0.33	0.70	0.484	564	0.18	-0.33	0.69	0.489
Total cholesterol in chylomicrons and extremely large VLDL (mmol/l)	564	0.10	-0.41	0.62	0.699	564	0.12	-0.40	0.63	0.654	564	0.12	-0.39	0.63	0.645
Cholesterol esters in chylomicrons and extremely large VLDL (mmol/l)	564	0.05	-0.47	0.56	0.860	564	0.06	-0.45	0.57	0.814	564	0.06	-0.43	0.56	0.800
Free cholesterol in chylomicrons and extremely large VLDL (mmol/l)	564	0.17	-0.35	0.68	0.529	564	0.18	-0.33	0.70	0.488	564	0.18	-0.33	0.69	0.491
Triglycerides in chylomicrons and extremely large VLDL (mmol/l)	564	0.17	-0.35	0.69	0.526	564	0.18	-0.33	0.70	0.486	564	0.18	-0.34	0.69	0.497
Concentration of very large VLDL particles (mol/l)	564	0.14	-0.37	0.65	0.595	564	0.16	-0.35	0.66	0.551	564	0.16	-0.35	0.66	0.548
Total lipids in very large VLDL (mmol/l)	564	0.13	-0.38	0.64	0.615	564	0.15	-0.36	0.65	0.569	564	0.15	-0.35	0.65	0.563
Phospholipids in very large VLDL (mmol/l)	564	0.13	-0.38	0.64	0.606	564	0.15	-0.36	0.66	0.562	564	0.15	-0.36	0.65	0.565
Total cholesterol in very large VLDL (mmol/l)	564	0.10	-0.41	0.61	0.700	564	0.12	-0.39	0.62	0.653	564	0.12	-0.39	0.62	0.652
Cholesterol esters in very large VLDL (mmol/l)	564	0.07	-0.43	0.58	0.772	564	0.09	-0.41	0.59	0.723	564	0.09	-0.40	0.58	0.722
Free cholesterol in very large VLDL (mmol/l)	564	0.13	-0.38	0.64	0.625	564	0.14	-0.37	0.66	0.580	564	0.14	-0.36	0.65	0.582
Triglycerides in very large VLDL (mmol/l)	564	0.14	-0.37	0.64	0.592	564	0.15	-0.35	0.66	0.548	564	0.16	-0.34	0.66	0.538
Concentration of large VLDL particles (mol/l)	564	0.08	-0.40	0.57	0.734	564	0.10	-0.38	0.59	0.683	564	0.11	-0.37	0.59	0.662
Total lipids in large VLDL (mmol/l)	564	0.08	-0.41	0.57	0.748	564	0.10	-0.39	0.58	0.697	564	0.10	-0.38	0.58	0.678
Phospholipids in large VLDL (mmol/l)	564	0.08	-0.40	0.57	0.735	564	0.10	-0.38	0.58	0.684	564	0.10	-0.37	0.58	0.670
Total cholesterol in large VLDL (mmol/l)	564	0.06	-0.43	0.55	0.813	564	0.07	-0.41	0.56	0.762	564	0.08	-0.40	0.55	0.751
Cholesterol esters in large VLDL (mmol/l)	564	0.02	-0.46	0.51	0.923	564	0.04	-0.44	0.52	0.873	564	0.04	-0.42	0.50	0.868
Free cholesterol in large VLDL (mmol/l)	564	0.09	-0.40	0.58	0.712	564	0.11	-0.38	0.60	0.662	564	0.11	-0.37	0.59	0.651
Triglycerides in large VLDL (mmol/l)	564	0.09	-0.40	0.57	0.726	564	0.10	-0.38	0.59	0.675	564	0.11	-0.37	0.59	0.653
Concentration of medium VLDL particles (mol/l)	564	0.00	-0.47	0.47	0.992	564	0.01	-0.45	0.48	0.956	564	0.02	-0.43	0.47	0.941
Total lipids in medium VLDL (mmol/l)	564	-0.01	-0.48	0.46	0.959	564	0.00	-0.46	0.46	0.990	564	0.01	-0.44	0.45	0.975
Phospholipids in medium VLDL (mmol/l)	564	-0.01	-0.47	0.45	0.962	564	0.00	-0.45	0.46	0.987	564	0.00	-0.44	0.45	0.983
Total cholesterol in medium VLDL (mmol/l)	564	-0.06	-0.52	0.41	0.812	564	-0.04	-0.50	0.41	0.856	564	-0.05	-0.48	0.37	0.811
Cholesterol esters in medium VLDL (mmol/l)	564	-0.11	-0.57	0.35	0.642	564	-0.10	-0.55	0.36	0.676	564	-0.12	-0.53	0.29	0.566
Free cholesterol in medium VLDL (mmol/l)	564	0.01	-0.45	0.47	0.959	564	0.03	-0.43	0.48	0.907	564	0.03	-0.41	0.47	0.900
Triglycerides in medium VLDL (mmol/l)	564	0.01	-0.46	0.48	0.970	564	0.02	-0.44	0.49	0.917	564	0.03	-0.42	0.49	0.882
Concentration of small VLDL particles (mol/l)	564	-0.04	-0.47	0.40	0.863	564	-0.02	-0.45	0.40	0.909	564	-0.04	-0.44	0.36	0.836
Total lipids in small VLDL (mmol/l)	564	-0.08	-0.51	0.36	0.730	564	-0.06	-0.49	0.36	0.770	564	-0.09	-0.48	0.29	0.636
Phospholipids in small VLDL (mmol/l)	564	-0.06	-0.47	0.36	0.785	564	-0.05	-0.45	0.36	0.827	564	-0.07	-0.44	0.29	0.695
Total cholesterol in small VLDL (mmol/l)	564	-0.17	-0.62	0.28	0.468	564	-0.16	-0.60	0.29	0.489	564	-0.24	-0.61	0.13	0.209
Cholesterol esters in small VLDL (mmol/l)	564	-0.21	-0.67	0.26	0.389	564	-0.20	-0.66	0.27	0.405	564	-0.29	-0.68	0.09	0.133
Free cholesterol in small VLDL (mmol/l)	564	-0.07	-0.49	0.35	0.749	564	-0.06	-0.47	0.36	0.787	564	-0.10	-0.46	0.27	0.600
Triglycerides in small VLDL (mmol/l)	564	0.01	-0.44	0.45	0.977	564	0.02	-0.42	0.46	0.926	564	0.02	-0.41	0.44	0.941
Concentration of very small VLDL particles (mol/l)	564	-0.12	-0.52	0.28	0.560	564	-0.11	-0.51	0.29	0.579	564	-0.24	-0.58	0.09	0.155
Total lipids in very small VLDL (mmol/l)	564	-0.19	-0.63	0.25	0.403	564	-0.18	-0.62	0.26	0.416	564	-0.33	-0.71	0.04	0.083
Phospholipids in very small VLDL (mmol/l)	564	-0.12	-0.52	0.28	0.558	564	-0.11	-0.51	0.28	0.572	564	-0.23	-0.54	0.09	0.164

S8 Table One-sample MR estimates of associations of age at voice breaking (per year later) with adiposity and cardiometabolic traits at age 18y among males in ALSPAC, using a refined GRS of 115 SNPs for age at menarche/voice breaking

	Unadj.					*Adj. for measured BMI at age 8y*					*Adj. for measured outcome value at age 8y*				
**Standardised outcome at age 18y**	**N**	**Beta (2SLS)**	**LCL**	**UCL**	**P-value**	**N**	**Beta (2SLS)**	**LCL**	**UCL**	**P-value**	**N**	**Beta (2SLS)**	**LCL**	**UCL**	**P-value**
Total cholesterol in very small VLDL (mmol/l)	564	-0.31	-0.81	0.19	0.227	564	-0.30	-0.80	0.19	0.232	564	-0.43	-0.90	0.03	0.069
Cholesterol esters in very small VLDL (mmol/l)	564	-0.29	-0.80	0.21	0.251	564	-0.29	-0.79	0.21	0.256	564	-0.42	-0.88	0.04	0.074
Free cholesterol in very small VLDL (mmol/l)	564	-0.30	-0.76	0.15	0.192	564	-0.30	-0.76	0.15	0.195	564	-0.40	-0.84	0.04	0.077
Triglycerides in very small VLDL (mmol/l)	564	0.09	-0.30	0.47	0.653	564	0.10	-0.28	0.48	0.609	564	0.06	-0.30	0.43	0.740
Concentration of IDL particles (mol/l)	564	-0.03	-0.43	0.37	0.895	564	-0.02	-0.43	0.38	0.908	564	-0.11	-0.43	0.21	0.514
Total lipids in IDL (mmol/l)	564	-0.08	-0.49	0.33	0.696	564	-0.08	-0.49	0.33	0.707	564	-0.18	-0.50	0.14	0.281
Phospholipids in IDL (mmol/l)	564	-0.02	-0.42	0.37	0.905	564	-0.02	-0.42	0.38	0.914	564	-0.09	-0.41	0.23	0.593
Total cholesterol in IDL (mmol/l)	564	-0.14	-0.57	0.28	0.509	564	-0.14	-0.57	0.29	0.518	564	-0.25	-0.59	0.10	0.160
Cholesterol esters in IDL (mmol/l)	564	-0.17	-0.60	0.27	0.455	564	-0.16	-0.60	0.27	0.465	564	-0.27	-0.62	0.08	0.131
Free cholesterol in IDL (mmol/l)	564	-0.09	-0.50	0.32	0.677	564	-0.09	-0.50	0.33	0.679	564	-0.17	-0.51	0.16	0.311
Triglycerides in IDL (mmol/l)	564	0.17	-0.18	0.52	0.338	564	0.18	-0.17	0.52	0.322	564	0.13	-0.19	0.46	0.427
Concentration of large LDL particles (mol/l)	564	0.00	-0.38	0.39	0.991	564	0.01	-0.38	0.39	0.975	564	-0.04	-0.36	0.27	0.795
Total lipids in large LDL (mmol/l)	564	-0.02	-0.41	0.37	0.903	564	-0.02	-0.41	0.37	0.918	564	-0.09	-0.40	0.22	0.559
Phospholipids in large LDL (mmol/l)	564	-0.05	-0.44	0.34	0.796	564	-0.05	-0.44	0.34	0.812	564	-0.11	-0.42	0.19	0.468
Total cholesterol in large LDL (mmol/l)	564	-0.05	-0.45	0.35	0.803	564	-0.05	-0.45	0.35	0.816	564	-0.12	-0.43	0.19	0.442
Cholesterol esters in large LDL (mmol/l)	564	-0.05	-0.44	0.35	0.817	564	-0.04	-0.44	0.36	0.833	564	-0.11	-0.42	0.20	0.470
Free cholesterol in large LDL (mmol/l)	564	-0.06	-0.46	0.34	0.769	564	-0.06	-0.46	0.34	0.775	564	-0.14	-0.46	0.18	0.388
Triglycerides in large LDL (mmol/l)	564	0.23	-0.13	0.59	0.219	564	0.23	-0.13	0.59	0.211	564	0.21	-0.14	0.55	0.238
Concentration of medium LDL particles (mol/l)	564	0.03	-0.35	0.41	0.876	564	0.04	-0.35	0.42	0.856	564	0.01	-0.32	0.33	0.975
Total lipids in medium LDL (mmol/l)	564	-0.01	-0.39	0.38	0.969	564	0.00	-0.39	0.38	0.988	564	-0.06	-0.37	0.25	0.699
Phospholipids in medium LDL (mmol/l)	564	-0.05	-0.44	0.33	0.791	564	-0.05	-0.43	0.34	0.816	564	-0.12	-0.42	0.19	0.456
Total cholesterol in medium LDL (mmol/l)	564	-0.04	-0.43	0.36	0.861	564	-0.03	-0.43	0.36	0.876	564	-0.09	-0.41	0.22	0.565
Cholesterol esters in medium LDL (mmol/l)	564	-0.02	-0.41	0.37	0.915	564	-0.02	-0.41	0.38	0.931	564	-0.07	-0.39	0.25	0.665
Free cholesterol in medium LDL (mmol/l)	564	-0.09	-0.49	0.31	0.653	564	-0.09	-0.49	0.31	0.665	564	-0.19	-0.51	0.13	0.254
Triglycerides in medium LDL (mmol/l)	564	0.29	-0.10	0.68	0.141	564	0.30	-0.09	0.68	0.136	564	0.28	-0.09	0.66	0.140
Concentration of small LDL particles (mol/l)	564	0.04	-0.34	0.42	0.846	564	0.04	-0.34	0.42	0.825	564	0.01	-0.31	0.34	0.930
Total lipids in small LDL (mmol/l)	564	-0.01	-0.39	0.38	0.975	564	0.00	-0.39	0.38	0.994	564	-0.05	-0.37	0.26	0.739
Phospholipids in small LDL (mmol/l)	564	-0.04	-0.42	0.34	0.836	564	-0.03	-0.41	0.34	0.861	564	-0.08	-0.39	0.22	0.594
Total cholesterol in small LDL (mmol/l)	564	-0.03	-0.43	0.37	0.880	564	-0.03	-0.42	0.37	0.894	564	-0.09	-0.40	0.23	0.602
Cholesterol esters in small LDL (mmol/l)	564	0.00	-0.39	0.39	0.999	564	0.00	-0.39	0.40	0.985	564	-0.04	-0.36	0.29	0.818
Free cholesterol in small LDL (mmol/l)	564	-0.16	-0.58	0.25	0.449	564	-0.16	-0.57	0.26	0.459	564	-0.27	-0.63	0.08	0.133
Triglycerides in small LDL (mmol/l)	564	0.26	-0.14	0.66	0.197	564	0.27	-0.13	0.67	0.183	564	0.26	-0.13	0.65	0.194
Concentration of very large HDL particles (mol/l)	564	0.27	-0.16	0.70	0.214	564	0.27	-0.16	0.69	0.217	564	0.26	-0.08	0.60	0.131
Total lipids in very large HDL (mmol/l)	564	0.28	-0.16	0.71	0.216	564	0.27	-0.16	0.70	0.219	564	0.27	-0.09	0.63	0.143
Phospholipids in very large HDL (mmol/l)	564	0.28	-0.14	0.70	0.198	564	0.27	-0.14	0.69	0.201	564	0.28	-0.05	0.60	0.095
Total cholesterol in very large HDL (mmol/l)	564	0.25	-0.21	0.70	0.288	564	0.24	-0.21	0.70	0.292	564	0.24	-0.17	0.64	0.254
Cholesterol esters in very large HDL (mmol/l)	564	0.23	-0.23	0.69	0.331	564	0.23	-0.23	0.69	0.336	564	0.22	-0.20	0.64	0.312
Free cholesterol in very large HDL (mmol/l)	564	0.27	-0.16	0.70	0.213	564	0.27	-0.16	0.69	0.216	564	0.27	-0.09	0.62	0.139
Triglycerides in very large HDL (mmol/l)	564	0.21	-0.24	0.66	0.357	564	0.22	-0.24	0.67	0.350	564	0.18	-0.24	0.61	0.400
Concentration of large HDL particles (mol/l)	564	0.16	-0.22	0.54	0.405	564	0.15	-0.22	0.53	0.417	564	0.19	-0.10	0.48	0.202
Total lipids in large HDL (mmol/l)	564	0.17	-0.21	0.55	0.389	564	0.16	-0.22	0.54	0.401	564	0.19	-0.10	0.48	0.195
Phospholipids in large HDL (mmol/l)	564	0.17	-0.21	0.55	0.371	564	0.17	-0.21	0.54	0.381	564	0.21	-0.09	0.50	0.169
Total cholesterol in large HDL (mmol/l)	564	0.17	-0.22	0.56	0.400	564	0.16	-0.22	0.54	0.414	564	0.18	-0.11	0.46	0.226
Cholesterol esters in large HDL (mmol/l)	564	0.17	-0.22	0.57	0.381	564	0.17	-0.22	0.55	0.394	564	0.19	-0.10	0.47	0.206
Free cholesterol in large HDL (mmol/l)	564	0.14	-0.24	0.52	0.482	564	0.13	-0.25	0.50	0.500	564	0.14	-0.14	0.41	0.339
Triglycerides in large HDL (mmol/l)	564	0.05	-0.29	0.40	0.768	564	0.05	-0.29	0.40	0.763	564	0.06	-0.26	0.38	0.723
Concentration of medium HDL particles (mol/l)	564	0.03	-0.28	0.34	0.836	564	0.03	-0.28	0.35	0.831	564	0.10	-0.20	0.40	0.528
Total lipids in medium HDL (mmol/l)	564	0.03	-0.30	0.35	0.874	564	0.03	-0.30	0.35	0.875	564	0.10	-0.21	0.41	0.539
Phospholipids in medium HDL (mmol/l)	564	0.08	-0.24	0.39	0.633	564	0.08	-0.24	0.39	0.631	564	0.13	-0.17	0.43	0.392
Total cholesterol in medium HDL (mmol/l)	564	-0.03	-0.39	0.33	0.878	564	-0.03	-0.39	0.33	0.866	564	0.06	-0.28	0.40	0.732
Cholesterol esters in medium HDL (mmol/l)	564	-0.04	-0.41	0.33	0.829	564	-0.04	-0.41	0.32	0.816	564	0.06	-0.29	0.40	0.751
Free cholesterol in medium HDL (mmol/l)	564	0.01	-0.33	0.36	0.933	564	0.01	-0.33	0.36	0.936	564	0.06	-0.27	0.40	0.720
Triglycerides in medium HDL (mmol/l)	564	0.01	-0.36	0.39	0.941	564	0.03	-0.34	0.39	0.892	564	0.08	-0.27	0.44	0.653
Concentration of small HDL particles (mol/l)	564	0.04	-0.29	0.36	0.825	564	0.04	-0.28	0.37	0.797	564	0.09	-0.23	0.41	0.585
Total lipids in small HDL (mmol/l)	564	0.04	-0.27	0.35	0.781	564	0.05	-0.26	0.36	0.761	564	0.08	-0.23	0.39	0.608
Phospholipids in small HDL (mmol/l)	564	0.06	-0.30	0.43	0.736	564	0.07	-0.30	0.44	0.718	564	0.12	-0.23	0.48	0.494
Total cholesterol in small HDL (mmol/l)	564	-0.01	-0.31	0.29	0.929	564	-0.01	-0.31	0.29	0.933	564	0.00	-0.29	0.29	1.000

S8 Table One-sample MR estimates of associations of age at voice breaking (per year later) with adiposity and cardiometabolic traits at age 18y among males in ALSPAC, using a refined GRS of 115 SNPs for age at menarche/voice breaking

	Unadj.					*Adj. for measured BMI at age 8y*					*Adj. for measured outcome value at age 8y*				
**Standardised outcome at age 18y**	**N**	**Beta (2SLS)**	**LCL**	**UCL**	**P-value**	**N**	**Beta (2SLS)**	**LCL**	**UCL**	**P-value**	**N**	**Beta (2SLS)**	**LCL**	**UCL**	**P-value**
Cholesterol esters in small HDL (mmol/l)	564	-0.03	-0.35	0.28	0.843	564	-0.03	-0.35	0.28	0.847	564	-0.03	-0.34	0.28	0.854
Free cholesterol in small HDL (mmol/l)	564	0.05	-0.31	0.40	0.799	564	0.05	-0.31	0.40	0.796	564	0.10	-0.24	0.44	0.564
Triglycerides in small HDL (mmol/l)	564	0.17	-0.22	0.57	0.392	564	0.19	-0.21	0.58	0.352	564	0.18	-0.21	0.57	0.365
Phospholipids to total lipds ratio in chylomicrons and extremely large VLDL (%)	564	-0.06	-0.61	0.50	0.843	564	-0.05	-0.60	0.50	0.860	564	-0.03	-0.59	0.53	0.912
Total cholesterol to total lipids ratio in chylomicrons and extremely large VLDL (%)	564	-0.19	-0.64	0.26	0.402	564	-0.19	-0.64	0.26	0.405	564	-0.16	-0.59	0.26	0.452
Cholesterol esters to total lipids ratio in chylomicrons and extremely large VLDL (%)	564	-0.31	-0.79	0.17	0.210	564	-0.31	-0.80	0.17	0.210	564	-0.30	-0.76	0.17	0.213
Free cholesterol to total lipids ratio in chylomicrons and extremely large VLDL (%)	564	0.32	-0.17	0.81	0.203	564	0.33	-0.16	0.83	0.188	564	0.36	-0.16	0.88	0.170
Triglycerides to total lipids ratio in chylomicrons and extremely large VLDL (%)	564	0.27	-0.17	0.72	0.228	564	0.27	-0.18	0.72	0.236	564	0.21	-0.22	0.64	0.332
Phospholipids to total lipds ratio in very large VLDL (%)	564	-0.03	-0.50	0.44	0.902	564	-0.02	-0.49	0.45	0.934	564	-0.01	-0.48	0.47	0.973
Total cholesterol to total lipids ratio in very large VLDL (%)	564	0.04	-0.25	0.33	0.774	564	0.04	-0.25	0.33	0.797	564	0.05	-0.24	0.33	0.753
Cholesterol esters to total lipids ratio in very large VLDL (%)	564	0.00	-0.23	0.22	0.976	564	-0.01	-0.24	0.22	0.943	564	-0.01	-0.24	0.22	0.914
Free cholesterol to total lipids ratio in very large VLDL (%)	564	0.05	-0.21	0.31	0.681	564	0.05	-0.21	0.32	0.685	564	0.09	-0.16	0.33	0.483
Triglycerides to total lipids ratio in very large VLDL (%)	564	-0.06	-0.42	0.30	0.745	564	-0.06	-0.41	0.30	0.757	564	-0.07	-0.42	0.27	0.683
Phospholipids to total lipds ratio in large VLDL (%)	564	0.06	-0.34	0.47	0.756	564	0.07	-0.34	0.48	0.730	564	0.06	-0.33	0.45	0.763
Total cholesterol to total lipids ratio in large VLDL (%)	564	0.08	-0.33	0.49	0.706	564	0.08	-0.33	0.49	0.688	564	0.09	-0.31	0.50	0.649
Cholesterol esters to total lipids ratio in large VLDL (%)	564	0.07	-0.25	0.40	0.656	564	0.07	-0.25	0.40	0.657	564	0.09	-0.24	0.41	0.607
Free cholesterol to total lipids ratio in large VLDL (%)	564	0.02	-0.15	0.20	0.783	564	0.03	-0.15	0.20	0.750	564	0.02	-0.15	0.19	0.838
Triglycerides to total lipids ratio in large VLDL (%)	564	-0.03	-0.46	0.40	0.893	564	-0.04	-0.47	0.39	0.868	564	-0.06	-0.48	0.36	0.776
Phospholipids to total lipds ratio in medium VLDL (%)	564	0.07	-0.29	0.43	0.697	564	0.07	-0.29	0.43	0.707	564	0.07	-0.24	0.37	0.676
Total cholesterol to total lipids ratio in medium VLDL (%)	564	-0.11	-0.52	0.31	0.613	564	-0.11	-0.52	0.31	0.616	564	-0.11	-0.47	0.26	0.566
Cholesterol esters to total lipids ratio in medium VLDL (%)	564	-0.16	-0.57	0.26	0.465	564	-0.16	-0.58	0.26	0.462	564	-0.15	-0.53	0.23	0.444
Free cholesterol to total lipids ratio in medium VLDL (%)	564	0.08	-0.32	0.48	0.683	564	0.09	-0.31	0.49	0.658	564	0.07	-0.31	0.45	0.721
Triglycerides to total lipids ratio in medium VLDL (%)	564	0.08	-0.32	0.47	0.707	564	0.08	-0.32	0.48	0.708	564	0.09	-0.27	0.44	0.633
Phospholipids to total lipds ratio in small VLDL (%)	564	0.16	-0.31	0.62	0.512	564	0.15	-0.32	0.61	0.531	564	0.19	-0.25	0.63	0.404
Total cholesterol to total lipids ratio in small VLDL (%)	564	-0.25	-0.75	0.26	0.337	564	-0.25	-0.76	0.25	0.326	564	-0.28	-0.77	0.20	0.249
Cholesterol esters to total lipids ratio in small VLDL (%)	564	-0.26	-0.76	0.25	0.320	564	-0.26	-0.77	0.24	0.311	564	-0.29	-0.78	0.19	0.232
Free cholesterol to total lipids ratio in small VLDL (%)	564	0.06	-0.29	0.40	0.744	564	0.05	-0.30	0.40	0.771	564	0.04	-0.29	0.36	0.828
Triglycerides to total lipids ratio in small VLDL (%)	564	0.20	-0.29	0.69	0.422	564	0.21	-0.28	0.69	0.403	564	0.22	-0.25	0.70	0.362
Phospholipids to total lipds ratio in very small VLDL (%)	564	0.05	-0.35	0.45	0.813	564	0.05	-0.35	0.45	0.811	564	0.06	-0.28	0.40	0.726
Total cholesterol to total lipids ratio in very small VLDL (%)	564	-0.35	-0.80	0.10	0.125	564	-0.36	-0.80	0.09	0.117	564	-0.36	-0.81	0.09	0.115
Cholesterol esters to total lipids ratio in very small VLDL (%)	564	-0.28	-0.69	0.13	0.182	564	-0.28	-0.69	0.13	0.176	564	-0.30	-0.70	0.11	0.158
Free cholesterol to total lipids ratio in very small VLDL (%)	564	-0.35	-0.80	0.10	0.131	564	-0.36	-0.81	0.09	0.117	564	-0.35	-0.81	0.10	0.126
Triglycerides to total lipids ratio in very small VLDL (%)	564	0.40	-0.11	0.91	0.129	564	0.40	-0.11	0.91	0.122	564	0.41	-0.10	0.92	0.113
Phospholipids to total lipds ratio in IDL (%)	564	0.44	-0.13	1.02	0.130	564	0.44	-0.13	1.01	0.134	564	0.49	-0.08	1.06	0.094
Total cholesterol to total lipids ratio in IDL (%)	564	-0.46	-0.99	0.07	0.086	564	-0.46	-0.99	0.07	0.086	564	-0.48	-0.99	0.03	0.066
Cholesterol esters to total lipids ratio in IDL (%)	564	-0.38	-0.86	0.10	0.120	564	-0.38	-0.85	0.10	0.123	564	-0.39	-0.85	0.06	0.092
Free cholesterol to total lipids ratio in IDL (%)	564	-0.24	-0.85	0.37	0.439	564	-0.25	-0.87	0.36	0.420	564	-0.24	-0.81	0.34	0.421
Triglycerides to total lipids ratio in IDL (%)	564	0.36	-0.12	0.85	0.139	564	0.37	-0.12	0.85	0.137	564	0.35	-0.11	0.81	0.133
Phospholipids to total lipds ratio in large LDL (%)	564	0.09	-0.45	0.64	0.739	564	0.09	-0.46	0.64	0.746	564	0.10	-0.31	0.51	0.624
Total cholesterol to total lipids ratio in large LDL (%)	564	-0.32	-0.90	0.27	0.289	564	-0.32	-0.90	0.27	0.290	564	-0.32	-0.77	0.14	0.176
Cholesterol esters to total lipids ratio in large LDL (%)	564	-0.21	-0.76	0.34	0.455	564	-0.21	-0.76	0.35	0.465	564	-0.18	-0.56	0.20	0.350
Free cholesterol to total lipids ratio in large LDL (%)	564	-0.17	-0.64	0.31	0.491	564	-0.18	-0.65	0.30	0.460	564	-0.20	-0.66	0.26	0.401
Triglycerides to total lipids ratio in large LDL (%)	564	0.41	-0.10	0.92	0.113	564	0.41	-0.10	0.92	0.113	564	0.41	-0.07	0.89	0.094
Phospholipids to total lipds ratio in medium LDL (%)	564	0.12	-0.45	0.70	0.674	564	0.12	-0.45	0.70	0.678	564	0.09	-0.36	0.55	0.690
Total cholesterol to total lipids ratio in medium LDL (%)	564	-0.32	-0.93	0.29	0.305	564	-0.32	-0.94	0.29	0.306	564	-0.30	-0.78	0.18	0.215
Cholesterol esters to total lipids ratio in medium LDL (%)	564	-0.19	-0.77	0.38	0.513	564	-0.19	-0.77	0.39	0.520	564	-0.16	-0.59	0.27	0.473
Free cholesterol to total lipids ratio in medium LDL (%)	564	-0.03	-0.53	0.48	0.916	564	-0.03	-0.54	0.47	0.898	564	-0.07	-0.50	0.36	0.737
Triglycerides to total lipids ratio in medium LDL (%)	564	0.45	-0.05	0.95	0.077	564	0.45	-0.05	0.96	0.077	564	0.45	-0.03	0.94	0.068
Phospholipids to total lipds ratio in small LDL (%)	564	0.10	-0.45	0.64	0.726	564	0.10	-0.45	0.64	0.732	564	0.09	-0.34	0.52	0.684
Total cholesterol to total lipids ratio in small LDL (%)	564	-0.26	-0.85	0.33	0.391	564	-0.26	-0.85	0.33	0.390	564	-0.26	-0.74	0.22	0.286
Cholesterol esters to total lipids ratio in small LDL (%)	564	-0.12	-0.67	0.44	0.681	564	-0.11	-0.67	0.44	0.686	564	-0.09	-0.52	0.34	0.692
Free cholesterol to total lipids ratio in small LDL (%)	564	-0.15	-0.64	0.34	0.550	564	-0.16	-0.65	0.34	0.534	564	-0.20	-0.65	0.25	0.388
Triglycerides to total lipids ratio in small LDL (%)	564	0.48	-0.07	1.03	0.088	564	0.49	-0.07	1.05	0.084	564	0.49	-0.06	1.04	0.083
Phospholipids to total lipds ratio in very large HDL (%)	564	0.31	-0.19	0.81	0.221	564	0.30	-0.19	0.80	0.228	564	0.39	-0.06	0.85	0.089
Total cholesterol to total lipids ratio in very large HDL (%)	564	-0.32	-0.82	0.19	0.215	564	-0.31	-0.81	0.19	0.221	564	-0.39	-0.84	0.07	0.097
Cholesterol esters to total lipids ratio in very large HDL (%)	564	-0.33	-0.83	0.18	0.208	564	-0.32	-0.82	0.18	0.214	564	-0.39	-0.85	0.06	0.090
Free cholesterol to total lipids ratio in very large HDL (%)	564	0.16	-0.40	0.73	0.567	564	0.16	-0.40	0.72	0.579	564	0.17	-0.38	0.72	0.552

S8 Table One-sample MR estimates of associations of age at voice breaking (per year later) with adiposity and cardiometabolic traits at age 18y among males in ALSPAC, using a refined GRS of 115 SNPs for age at menarche/voice breaking

	Unadj.					*Adj. for measured BMI at age 8y*					*Adj. for measured outcome value at age 8y*				
**Standardised outcome at age 18y**	**N**	**Beta (2SLS)**	**LCL**	**UCL**	**P-value**	**N**	**Beta (2SLS)**	**LCL**	**UCL**	**P-value**	**N**	**Beta (2SLS)**	**LCL**	**UCL**	**P-value**
Triglycerides to total lipids ratio in very large HDL (%)	564	-0.02	-0.49	0.46	0.942	564	0.00	-0.47	0.46	0.985	564	-0.08	-0.52	0.36	0.726
Phospholipids to total lipds ratio in large HDL (%)	564	-0.07	-0.47	0.33	0.747	564	-0.05	-0.44	0.34	0.798	564	-0.01	-0.37	0.35	0.950
Total cholesterol to total lipids ratio in large HDL (%)	564	0.10	-0.32	0.53	0.633	564	0.09	-0.32	0.49	0.676	564	0.07	-0.28	0.42	0.714
Cholesterol esters to total lipids ratio in large HDL (%)	564	0.16	-0.28	0.60	0.479	564	0.14	-0.28	0.56	0.507	564	0.14	-0.21	0.49	0.434
Free cholesterol to total lipids ratio in large HDL (%)	564	-0.12	-0.56	0.31	0.573	564	-0.14	-0.57	0.29	0.517	564	-0.22	-0.64	0.21	0.317
Triglycerides to total lipids ratio in large HDL (%)	564	-0.10	-0.56	0.36	0.667	564	-0.09	-0.54	0.36	0.704	564	-0.17	-0.55	0.21	0.389
Phospholipids to total lipds ratio in medium HDL (%)	564	0.26	-0.23	0.74	0.302	564	0.26	-0.23	0.76	0.298	564	0.25	-0.23	0.73	0.306
Total cholesterol to total lipids ratio in medium HDL (%)	564	-0.22	-0.69	0.26	0.369	564	-0.23	-0.71	0.25	0.351	564	-0.22	-0.68	0.25	0.362
Cholesterol esters to total lipids ratio in medium HDL (%)	564	-0.24	-0.71	0.24	0.327	564	-0.25	-0.73	0.23	0.309	564	-0.22	-0.69	0.24	0.344
Free cholesterol to total lipids ratio in medium HDL (%)	564	-0.05	-0.53	0.44	0.844	564	-0.05	-0.54	0.44	0.838	564	-0.10	-0.59	0.40	0.700
Triglycerides to total lipids ratio in medium HDL (%)	564	0.05	-0.40	0.49	0.838	564	0.06	-0.37	0.50	0.785	564	0.09	-0.31	0.48	0.670
Phospholipids to total lipds ratio in small HDL (%)	564	0.03	-0.35	0.41	0.875	564	0.03	-0.35	0.41	0.872	564	0.07	-0.28	0.43	0.691
Total cholesterol to total lipids ratio in small HDL (%)	564	-0.09	-0.48	0.29	0.634	564	-0.10	-0.49	0.29	0.619	564	-0.12	-0.49	0.25	0.512
Cholesterol esters to total lipids ratio in small HDL (%)	564	-0.09	-0.49	0.31	0.665	564	-0.09	-0.49	0.31	0.656	564	-0.12	-0.50	0.26	0.543
Free cholesterol to total lipids ratio in small HDL (%)	564	-0.02	-0.49	0.45	0.938	564	-0.03	-0.50	0.44	0.906	564	0.01	-0.42	0.44	0.963
Triglycerides to total lipids ratio in small HDL (%)	564	0.21	-0.27	0.70	0.390	564	0.23	-0.26	0.71	0.358	564	0.20	-0.27	0.67	0.411
Mean diameter for VLDL particles (nm)	564	0.09	-0.36	0.55	0.695	564	0.10	-0.35	0.56	0.652	564	0.12	-0.33	0.56	0.600
Mean diameter for LDL particles (nm)	564	-0.13	-0.59	0.33	0.572	564	-0.14	-0.60	0.32	0.556	564	-0.18	-0.62	0.26	0.425
Mean diameter for HDL particles (nm)	564	0.21	-0.22	0.63	0.342	564	0.20	-0.22	0.61	0.351	564	0.19	-0.13	0.51	0.241
Serum total cholesterol (mmol/l)	564	-0.04	-0.41	0.33	0.839	564	-0.04	-0.41	0.34	0.854	564	-0.09	-0.38	0.20	0.545
Total cholesterol in VLDL (mmol/l)	564	-0.13	-0.60	0.33	0.569	564	-0.12	-0.58	0.34	0.599	564	-0.18	-0.58	0.22	0.388
Remnant cholesterol (non-HDL, non-LDL -cholesterol) (mmol/l)	564	-0.15	-0.60	0.30	0.502	564	-0.15	-0.59	0.30	0.523	564	-0.24	-0.60	0.13	0.207
Total cholesterol in LDL (mmol/l)	564	-0.04	-0.44	0.35	0.836	564	-0.04	-0.44	0.36	0.850	564	-0.11	-0.42	0.21	0.507
Total cholesterol in HDL (mmol/l)	564	0.13	-0.23	0.50	0.466	564	0.13	-0.23	0.49	0.477	564	0.17	-0.11	0.45	0.235
Total cholesterol in HDL2 (mmol/l)	564	0.12	-0.25	0.50	0.521	564	0.12	-0.25	0.48	0.537	564	0.16	-0.14	0.45	0.288
Total cholesterol in HDL3 (mmol/l)	564	0.15	-0.20	0.49	0.399	564	0.15	-0.20	0.49	0.403	564	0.18	-0.09	0.45	0.198
Esterified cholesterol (mmol/l)	564	-0.06	-0.44	0.32	0.752	564	-0.06	-0.44	0.32	0.766	564	-0.09	-0.39	0.22	0.580
Free cholesterol (mmol/l)	564	0.02	-0.35	0.39	0.924	564	0.02	-0.35	0.39	0.906	564	-0.08	-0.37	0.20	0.567
Serum total triglycerides (mmol/l)	564	0.10	-0.35	0.56	0.656	564	0.12	-0.33	0.57	0.606	564	0.11	-0.33	0.56	0.615
Triglycerides in VLDL (mmol/l)	564	0.05	-0.42	0.53	0.822	564	0.07	-0.40	0.54	0.769	564	0.07	-0.39	0.53	0.756
Triglycerides in LDL (mmol/l)	564	0.25	-0.12	0.63	0.182	564	0.26	-0.12	0.64	0.174	564	0.24	-0.12	0.60	0.187
Triglycerides in HDL (mmol/l)	564	0.13	-0.25	0.51	0.499	564	0.14	-0.24	0.52	0.462	564	0.15	-0.23	0.52	0.440
Diacylglycerol (mmol/l)	564	0.10	-0.31	0.51	0.623	564	0.11	-0.30	0.52	0.594	564	0.10	-0.30	0.50	0.638
Ratio of diacylglycerol to triglycerides	564	0.11	-0.28	0.50	0.578	564	0.12	-0.28	0.51	0.563	564	0.11	-0.28	0.50	0.578
Total phosphoglycerides (mmol/l)	564	0.17	-0.17	0.52	0.324	564	0.18	-0.17	0.53	0.315	564	0.16	-0.17	0.48	0.341
Ratio of triglycerides to phosphoglycerides	564	-0.08	-0.57	0.40	0.738	564	-0.07	-0.55	0.41	0.772	564	-0.06	-0.53	0.40	0.788
Phosphatidylcholine and other cholines (mmol/l)	564	0.09	-0.23	0.41	0.575	564	0.09	-0.23	0.41	0.571	564	0.10	-0.20	0.40	0.505
Total cholines (mmol/l)	564	0.10	-0.24	0.43	0.570	564	0.10	-0.24	0.44	0.561	564	0.07	-0.24	0.38	0.671
Apolipoprotein A-I (g/l)	564	0.08	-0.24	0.40	0.632	564	0.08	-0.24	0.40	0.636	564	0.11	-0.14	0.36	0.383
Apolipoprotein B (g/l)	564	-0.07	-0.50	0.36	0.749	564	-0.06	-0.49	0.37	0.783	564	-0.12	-0.48	0.25	0.538
Ratio of apolipoprotein B to apolipoprotein A-I	564	-0.10	-0.57	0.38	0.691	564	-0.08	-0.55	0.38	0.722	564	-0.17	-0.55	0.22	0.400
Total fatty acids (mmol/l)	564	0.04	-0.34	0.42	0.833	564	0.05	-0.33	0.43	0.791	564	0.02	-0.34	0.38	0.893
Estimated description of fatty acid chain length, not actual carbon number	564	0.16	-0.29	0.60	0.493	564	0.16	-0.28	0.60	0.481	564	0.17	-0.27	0.62	0.441
Estimated degree of unsaturation	564	-0.04	-0.48	0.40	0.863	564	-0.04	-0.49	0.40	0.847	564	0.03	-0.38	0.43	0.903
22:6, docosahexaenoic acid (mmol/l)	564	-0.21	-0.58	0.16	0.273	564	-0.20	-0.57	0.17	0.285	564	-0.09	-0.39	0.21	0.560
18:2, linoleic acid (mmol/l)	564	0.04	-0.33	0.41	0.831	564	0.04	-0.33	0.41	0.824	564	0.08	-0.24	0.40	0.629
Conjugated linoleic acid (mmol/l)	564	0.04	-0.38	0.46	0.860	564	0.05	-0.38	0.47	0.827	564	0.04	-0.38	0.47	0.845
Omega-3 fatty acids (mmol/l)	564	-0.32	-0.77	0.14	0.172	564	-0.31	-0.77	0.14	0.177	564	-0.30	-0.71	0.10	0.145
Omega-6 fatty acids (mmol/l)	564	0.08	-0.28	0.45	0.646	564	0.09	-0.27	0.45	0.628	564	0.10	-0.21	0.42	0.524
Polyunsaturated fatty acids (mmol/l)	564	0.03	-0.33	0.39	0.860	564	0.04	-0.33	0.40	0.838	564	0.05	-0.26	0.36	0.748
Monounsaturated fatty acids; 16:1, 18:1 (mmol/l)	564	0.10	-0.33	0.53	0.648	564	0.11	-0.31	0.54	0.598	564	0.06	-0.35	0.48	0.760
Saturated fatty acids (mmol/l)	564	-0.01	-0.40	0.37	0.944	564	0.00	-0.39	0.38	0.984	564	-0.02	-0.40	0.35	0.913
Ratio of 22:6 docosahexaenoic acid to total fatty acids (%)	564	-0.36	-0.82	0.09	0.121	564	-0.36	-0.82	0.10	0.123	564	-0.18	-0.54	0.17	0.311
Ratio of 18:2 linoleic acid to total fatty acids (%)	564	0.10	-0.38	0.58	0.683	564	0.08	-0.38	0.55	0.722	564	0.15	-0.32	0.62	0.526
Ratio of conjugated linoleic acid to total fatty acids (%)	564	0.01	-0.41	0.43	0.973	564	0.01	-0.41	0.44	0.949	564	0.01	-0.42	0.43	0.979
Ratio of omega-3 fatty acids to total fatty acids (%)	564	-0.55	-1.11	0.02	0.058	564	-0.55	-1.12	0.02	0.058	564	-0.51	-1.04	0.01	0.056
Ratio of omega-6 fatty acids to total fatty acids (%)	564	0.18	-0.30	0.67	0.453	564	0.17	-0.30	0.65	0.479	564	0.21	-0.27	0.68	0.392

S8 Table One-sample MR estimates of associations of age at voice breaking (per year later) with adiposity and cardiometabolic traits at age 18y among males in ALSPAC, using a refined GRS of 115 SNPs for age at menarche/voice breaking

	Unadj.					*Adj. for measured BMI at age 8y*					*Adj. for measured outcome value at age 8y*				
**Standardised outcome at age 18y**	**N**	**Beta (2SLS)**	**LCL**	**UCL**	**P-value**	**N**	**Beta (2SLS)**	**LCL**	**UCL**	**P-value**	**N**	**Beta (2SLS)**	**LCL**	**UCL**	**P-value**
Ratio of polyunsaturated fatty acids to total fatty acids (%)	564	0.04	-0.42	0.51	0.858	564	0.03	-0.43	0.49	0.899	564	0.07	-0.38	0.53	0.757
Ratio of monounsaturated fatty acids to total fatty acids (%)	564	0.14	-0.35	0.63	0.572	564	0.15	-0.33	0.64	0.532	564	0.09	-0.38	0.56	0.703
Ratio of saturated fatty acids to total fatty acids (%)	564	-0.25	-0.74	0.25	0.330	564	-0.25	-0.74	0.25	0.329	564	-0.24	-0.72	0.24	0.330
Glucose (mmol/l)	564	0.05	-0.27	0.37	0.756	564	0.05	-0.27	0.38	0.744	564	0.07	-0.26	0.41	0.678
Lactate (mmol/l)	564	0.24	-0.23	0.72	0.314	564	0.25	-0.23	0.73	0.310	564	0.25	-0.23	0.72	0.309
Pyruvate (mmol/l)	564	0.20	-0.27	0.67	0.402	564	0.21	-0.26	0.68	0.385	564	0.19	-0.28	0.66	0.427
Citrate (mmol/l)	564	0.41	-0.10	0.91	0.114	564	0.40	-0.10	0.91	0.119	564	0.34	-0.15	0.83	0.173
Alanine (mmol/l)	564	0.47	-0.01	0.96	0.057	564	0.48	-0.01	0.97	0.054	564	0.48	0.00	0.97	0.052
Glutamine (mmol/l)	564	-0.02	-0.35	0.32	0.920	564	-0.02	-0.36	0.31	0.899	564	0.02	-0.30	0.34	0.885
Histidine (mmol/l)	564	0.23	-0.20	0.66	0.292	564	0.23	-0.20	0.66	0.289	564	0.25	-0.17	0.67	0.240
Isoleucine (mmol/l)	564	0.38	-0.07	0.83	0.099	564	0.39	-0.05	0.84	0.085	564	0.38	-0.06	0.83	0.089
Leucine (mmol/l)	564	0.22	-0.17	0.62	0.273	564	0.23	-0.16	0.62	0.241	564	0.24	-0.16	0.63	0.237
Valine (mmol/l)	564	0.05	-0.34	0.44	0.808	564	0.06	-0.33	0.45	0.763	564	0.08	-0.30	0.45	0.688
Phenylalanine (mmol/l)	564	-0.03	-0.50	0.44	0.891	564	-0.02	-0.49	0.44	0.919	564	0.07	-0.36	0.51	0.735
Tyrosine (mmol/l)	564	0.15	-0.19	0.49	0.397	564	0.16	-0.18	0.50	0.367	564	0.16	-0.18	0.51	0.360
Acetate (mmol/l)	564	0.00	-0.16	0.16	0.972	564	0.00	-0.16	0.16	0.977	564	0.02	-0.13	0.17	0.795
Acetoacetate (mmol/l)	564	0.17	-0.42	0.77	0.572	564	0.18	-0.41	0.78	0.550	564	0.18	-0.42	0.77	0.556
3-hydroxybutyrate (mmol/l)	564	0.23	-0.27	0.73	0.377	564	0.23	-0.27	0.73	0.361	564	0.23	-0.27	0.73	0.370
Creatinine (mmol/l)	564	-0.42	-0.89	0.05	0.082	564	-0.41	-0.88	0.06	0.085	564	-0.45	-0.92	0.02	0.061
Albumin (signal area)	564	-0.19	-0.63	0.24	0.382	564	-0.19	-0.63	0.24	0.383	564	-0.19	-0.62	0.24	0.387
Glycoprotein acetyls, mainly a1-acid glycoprotein (mmol/l)	564	0.07	-0.33	0.47	0.743	564	0.08	-0.33	0.48	0.703	564	0.05	-0.34	0.43	0.807
