# Supplementary material for: Influence of puberty timing on adiposity and cardiometabolic traits: A Mendelian randomisation study
Source: PLoS Med. 2018 Aug 28;15(8):e1002641. doi: 10.1371/journal.pmed.1002641 (PMC6112630; doi:10.1371/journal.pmed.1002641)
Supplement: S9 Table — (PDF) [file pmed.1002641.s028.pdf]

S9 Table One-sample multivariate Mendelian randomization estimates of genetically predicted age at menarche and childhood body mass index with adulthood adiposity and blood pressure among females in ALSPAC, for comparisons with Fig 2

		**Unadjusted**			**Adjusted for genetically predicted body mass index at age 8y**	
	**N**	**Standardized beta (95% CI) per year later genetically predicted age at menarche**	**P-value**	**N**	**Standardized beta (95% CI) per year later genetically predicted age at menarche**	**P-value**
**Standardised outcome at age 18y**						
Body mass index	3957	-0.28 (-0.36, -0.19)	2.92E-10	3957	-0.001 (-0.10, 0.10)	0.985
Fat mass index	3805	-0.11 (-0.20, -0.02)	0.013	3805	0.13 (0.02, 0.23)	0.020
Lean mass index	3805	-0.32 (-0.41, -0.23)	1.81E-12	3805	-0.24 (-0.35, -0.13)	1.65E-05
Systolic blood pressure	3693	-0.25 (-0.34, -0.16)	5.39E-08	3693	-0.22 (-0.33, -0.11)	1.33E-04
Diastolic blood pressure	3693	-0.07 (-0.16, 0.02)	0.146	3693	-0.04 (-0.15, 0.07)	0.481

		**Unadjusted**			**Adjusted for genetically predicted age at menarche**	
	**N**	**Standardized beta (95% CI) per SD higher genetically predicted body mass index at age 8y**	**P-value**	**N**	**Standardized beta (95% CI) per year later genetically predicted body mass index at age 8y**	**P-value**
**Standardised outcome at age 18y**						
Body mass index	3957	1.11 (0.94, 1.29)	9.21E-35	3957	1.11 (0.91, 1.32)	1.80E-26
Fat mass index	3805	0.90 (0.72, 1.09)	9.35E-22	3805	0.98 (0.77, 1.19)	3.19E-19
Lean mass index	3805	0.47 (0.29, 0.66)	6.72E-07	3805	0.34 (0.12, 0.55)	0.002
Systolic blood pressure	3693	0.27 (0.08, 0.47)	0.005	3693	0.16 (-0.06, 0.38)	0.164
Diastolic blood pressure	3693	0.14 (-0.05, 0.33)	0.159	3693	0.12 (-0.10, 0.34)	0.304

For unadjusted models, genetically predicted age at menarche and childhood body mass index were first derived by regressing each exposure trait with its externally weighted GRS as an independent variable. Predicted values from first-stage regressions were then used as independent variables in relation to outcome traits in second-stage models. For multivariate adjusted models, genetically predicted age at menarche and childhood body mass index were first derived by regressing each exposure trait with *both* externally weighted GRSs as two independent variables. Predicted values from first-stage regressions were then used as independent variables in relation to outcome traits in second-stage models.
