# Supplementary material for: Influence of puberty timing on adiposity and cardiometabolic traits: A Mendelian randomisation study
Source: PLoS Med. 2018 Aug 28;15(8):e1002641. doi: 10.1371/journal.pmed.1002641 (PMC6112630; doi:10.1371/journal.pmed.1002641)
Supplement: S10 Table — (PDF) [file pmed.1002641.s029.pdf]

S10 Table Negative control one-sample MR estimates of associations of age at menarche (per year later) with adiposity and cardiometabolic traits at age 8y among females in ALSPAC, using a full GRS of 351 SNPs for age at menarche

	Unadj.				
**Standardised outcome at age 8y**	**N**	**Beta (2SLS)**	**LCL**	**UCL**	**P-value**
Body mass index (kg/m^2)	2648	-0.39	-0.50	-0.29	6.28E-13
Fat mass index (kg/m^2)	2569	-0.33	-0.44	-0.23	2.75E-10
Lean mass index (kg/m^2)	2569	-0.34	-0.44	-0.24	1.26E-11
Systolic blood pressure (mmHg)	2618	-0.19	-0.30	-0.08	4.94E-04
Diastolic blood pressure (mmHg)	2619	-0.17	-0.27	-0.07	1.25E-03
Concentration of chylomicrons and extremely large VLDL particles (mol/l)	1946	-0.04	-0.16	0.08	0.492
Total lipids in chylomicrons and extremely large VLDL (mmol/l)	1946	-0.04	-0.16	0.08	0.551
Phospholipids in chylomicrons and extremely large VLDL (mmol/l)	1946	-0.04	-0.16	0.08	0.542
Total cholesterol in chylomicrons and extremely large VLDL (mmol/l)	1946	-0.03	-0.16	0.09	0.570
Cholesterol esters in chylomicrons and extremely large VLDL (mmol/l)	1946	-0.03	-0.15	0.09	0.642
Free cholesterol in chylomicrons and extremely large VLDL (mmol/l)	1946	-0.04	-0.16	0.08	0.525
Triglycerides in chylomicrons and extremely large VLDL (mmol/l)	1946	-0.04	-0.15	0.08	0.551
Concentration of very large VLDL particles (mol/l)	1946	-0.04	-0.15	0.08	0.561
Total lipids in very large VLDL (mmol/l)	1946	-0.03	-0.15	0.09	0.579
Phospholipids in very large VLDL (mmol/l)	1946	-0.04	-0.16	0.08	0.556
Total cholesterol in very large VLDL (mmol/l)	1946	-0.04	-0.16	0.08	0.535
Cholesterol esters in very large VLDL (mmol/l)	1946	-0.04	-0.16	0.09	0.568
Free cholesterol in very large VLDL (mmol/l)	1946	-0.04	-0.16	0.08	0.507
Triglycerides in very large VLDL (mmol/l)	1946	-0.03	-0.15	0.09	0.601
Concentration of large VLDL particles (mol/l)	1946	-0.03	-0.15	0.09	0.601
Total lipids in large VLDL (mmol/l)	1946	-0.03	-0.15	0.09	0.605
Phospholipids in large VLDL (mmol/l)	1946	-0.03	-0.15	0.09	0.573
Total cholesterol in large VLDL (mmol/l)	1946	-0.03	-0.15	0.09	0.599
Cholesterol esters in large VLDL (mmol/l)	1946	-0.03	-0.15	0.09	0.609
Free cholesterol in large VLDL (mmol/l)	1946	-0.03	-0.15	0.09	0.596
Triglycerides in large VLDL (mmol/l)	1946	-0.03	-0.15	0.09	0.618
Concentration of medium VLDL particles (mol/l)	1946	-0.04	-0.16	0.08	0.505
Total lipids in medium VLDL (mmol/l)	1946	-0.04	-0.16	0.08	0.515
Phospholipids in medium VLDL (mmol/l)	1946	-0.04	-0.16	0.08	0.486
Total cholesterol in medium VLDL (mmol/l)	1946	-0.04	-0.16	0.08	0.530
Cholesterol esters in medium VLDL (mmol/l)	1946	-0.03	-0.16	0.09	0.607
Free cholesterol in medium VLDL (mmol/l)	1946	-0.04	-0.16	0.08	0.484
Triglycerides in medium VLDL (mmol/l)	1946	-0.04	-0.16	0.08	0.529
Concentration of small VLDL particles (mol/l)	1946	-0.05	-0.17	0.07	0.455
Total lipids in small VLDL (mmol/l)	1946	-0.05	-0.17	0.07	0.434
Phospholipids in small VLDL (mmol/l)	1946	-0.03	-0.15	0.09	0.651
Total cholesterol in small VLDL (mmol/l)	1946	-0.05	-0.18	0.07	0.397
Cholesterol esters in small VLDL (mmol/l)	1946	-0.06	-0.19	0.07	0.358
Free cholesterol in small VLDL (mmol/l)	1946	-0.04	-0.16	0.08	0.552
Triglycerides in small VLDL (mmol/l)	1946	-0.05	-0.17	0.08	0.458
Concentration of very small VLDL particles (mol/l)	1946	-0.05	-0.18	0.08	0.434
Total lipids in very small VLDL (mmol/l)	1946	-0.05	-0.17	0.08	0.479
Phospholipids in very small VLDL (mmol/l)	1946	-0.05	-0.18	0.08	0.421
Total cholesterol in very small VLDL (mmol/l)	1946	-0.02	-0.15	0.11	0.776
Cholesterol esters in very small VLDL (mmol/l)	1946	-0.03	-0.16	0.10	0.606
Free cholesterol in very small VLDL (mmol/l)	1946	0.01	-0.12	0.14	0.850
Triglycerides in very small VLDL (mmol/l)	1946	-0.06	-0.18	0.06	0.331
Concentration of IDL particles (mol/l)	1946	-0.06	-0.19	0.08	0.397
Total lipids in IDL (mmol/l)	1946	-0.04	-0.18	0.09	0.511
Phospholipids in IDL (mmol/l)	1946	-0.05	-0.19	0.09	0.464
Total cholesterol in IDL (mmol/l)	1946	-0.03	-0.16	0.10	0.627
Cholesterol esters in IDL (mmol/l)	1946	-0.03	-0.16	0.10	0.663
Free cholesterol in IDL (mmol/l)	1946	-0.04	-0.17	0.10	0.576
Triglycerides in IDL (mmol/l)	1946	-0.08	-0.21	0.06	0.262
Concentration of large LDL particles (mol/l)	1946	-0.05	-0.19	0.09	0.470
Total lipids in large LDL (mmol/l)	1946	-0.04	-0.18	0.09	0.542
Phospholipids in large LDL (mmol/l)	1946	-0.04	-0.18	0.09	0.548
Total cholesterol in large LDL (mmol/l)	1946	-0.04	-0.17	0.10	0.594
Cholesterol esters in large LDL (mmol/l)	1946	-0.04	-0.17	0.10	0.583
Free cholesterol in large LDL (mmol/l)	1946	-0.03	-0.17	0.10	0.633
Triglycerides in large LDL (mmol/l)	1946	-0.07	-0.21	0.07	0.323
Concentration of medium LDL particles (mol/l)	1946	-0.05	-0.19	0.09	0.449
Total lipids in medium LDL (mmol/l)	1946	-0.04	-0.18	0.09	0.540
Phospholipids in medium LDL (mmol/l)	1946	-0.02	-0.15	0.11	0.769
Total cholesterol in medium LDL (mmol/l)	1946	-0.04	-0.18	0.09	0.539
Cholesterol esters in medium LDL (mmol/l)	1946	-0.05	-0.19	0.09	0.472
Free cholesterol in medium LDL (mmol/l)	1946	-0.01	-0.14	0.13	0.918

S10 Table Negative control one-sample MR estimates of associations of age at menarche (per year later) with adiposity and cardiometabolic traits at age 8y among females in ALSPAC, using a full GRS of 351 SNPs for age at menarche

	Unadj.				
**Standardised outcome at age 8y**	**N**	**Beta (2SLS)**	**LCL**	**UCL**	**P-value**
Triglycerides in medium LDL (mmol/l)	1946	-0.06	-0.21	0.08	0.384
Concentration of small LDL particles (mol/l)	1946	-0.03	-0.17	0.11	0.663
Total lipids in small LDL (mmol/l)	1946	-0.03	-0.17	0.10	0.642
Phospholipids in small LDL (mmol/l)	1946	0.00	-0.14	0.13	0.959
Total cholesterol in small LDL (mmol/l)	1946	-0.03	-0.17	0.10	0.626
Cholesterol esters in small LDL (mmol/l)	1946	-0.05	-0.19	0.09	0.506
Free cholesterol in small LDL (mmol/l)	1946	0.03	-0.10	0.16	0.632
Triglycerides in small LDL (mmol/l)	1946	-0.06	-0.20	0.08	0.375
Concentration of very large HDL particles (mol/l)	1946	0.02	-0.10	0.15	0.728
Total lipids in very large HDL (mmol/l)	1946	0.04	-0.09	0.16	0.583
Phospholipids in very large HDL (mmol/l)	1946	0.02	-0.11	0.14	0.813
Total cholesterol in very large HDL (mmol/l)	1946	0.06	-0.07	0.18	0.360
Cholesterol esters in very large HDL (mmol/l)	1946	0.07	-0.06	0.19	0.300
Free cholesterol in very large HDL (mmol/l)	1946	0.04	-0.09	0.16	0.563
Triglycerides in very large HDL (mmol/l)	1946	-0.02	-0.14	0.10	0.721
Concentration of large HDL particles (mol/l)	1946	0.05	-0.07	0.17	0.447
Total lipids in large HDL (mmol/l)	1946	0.05	-0.07	0.17	0.434
Phospholipids in large HDL (mmol/l)	1946	0.04	-0.08	0.17	0.512
Total cholesterol in large HDL (mmol/l)	1946	0.06	-0.06	0.18	0.363
Cholesterol esters in large HDL (mmol/l)	1946	0.06	-0.06	0.18	0.365
Free cholesterol in large HDL (mmol/l)	1946	0.06	-0.06	0.18	0.366
Triglycerides in large HDL (mmol/l)	1946	-0.03	-0.16	0.10	0.625
Concentration of medium HDL particles (mol/l)	1946	0.09	-0.03	0.21	0.160
Total lipids in medium HDL (mmol/l)	1946	0.09	-0.03	0.21	0.150
Phospholipids in medium HDL (mmol/l)	1946	0.09	-0.03	0.21	0.154
Total cholesterol in medium HDL (mmol/l)	1946	0.10	-0.02	0.22	0.113
Cholesterol esters in medium HDL (mmol/l)	1946	0.10	-0.02	0.22	0.110
Free cholesterol in medium HDL (mmol/l)	1946	0.09	-0.03	0.21	0.142
Triglycerides in medium HDL (mmol/l)	1946	-0.06	-0.18	0.07	0.355
Concentration of small HDL particles (mol/l)	1946	0.05	-0.07	0.17	0.415
Total lipids in small HDL (mmol/l)	1946	0.07	-0.05	0.19	0.261
Phospholipids in small HDL (mmol/l)	1946	0.07	-0.05	0.19	0.253
Total cholesterol in small HDL (mmol/l)	1946	0.05	-0.07	0.17	0.403
Cholesterol esters in small HDL (mmol/l)	1946	0.03	-0.09	0.15	0.659
Free cholesterol in small HDL (mmol/l)	1946	0.13	0.00	0.25	0.043
Triglycerides in small HDL (mmol/l)	1946	-0.06	-0.19	0.06	0.308
Phospholipids to total lipds ratio in chylomicrons and extremely large VLDL (%)	1946	0.01	-0.08	0.10	0.841
Total cholesterol to total lipids ratio in chylomicrons and extremely large VLDL (%)	1946	-0.02	-0.14	0.11	0.802
Cholesterol esters to total lipids ratio in chylomicrons and extremely large VLDL (%)	1946	0.00	-0.13	0.12	0.959
Free cholesterol to total lipids ratio in chylomicrons and extremely large VLDL (%)	1946	-0.03	-0.14	0.08	0.563
Triglycerides to total lipids ratio in chylomicrons and extremely large VLDL (%)	1946	0.00	-0.04	0.04	0.942
Phospholipids to total lipds ratio in very large VLDL (%)	1946	-0.06	-0.18	0.05	0.278
Total cholesterol to total lipids ratio in very large VLDL (%)	1946	0.00	-0.03	0.03	0.975
Cholesterol esters to total lipids ratio in very large VLDL (%)	1946	0.02	-0.06	0.11	0.559
Free cholesterol to total lipids ratio in very large VLDL (%)	1946	-0.01	-0.05	0.04	0.736
Triglycerides to total lipids ratio in very large VLDL (%)	1946	0.00	-0.10	0.10	0.995
Phospholipids to total lipds ratio in large VLDL (%)	1946	-0.06	-0.16	0.04	0.265
Total cholesterol to total lipids ratio in large VLDL (%)	1946	-0.02	-0.14	0.09	0.701
Cholesterol esters to total lipids ratio in large VLDL (%)	1946	0.00	-0.08	0.08	0.984
Free cholesterol to total lipids ratio in large VLDL (%)	1946	-0.02	-0.05	0.02	0.409
Triglycerides to total lipids ratio in large VLDL (%)	1946	0.03	-0.05	0.12	0.424
Phospholipids to total lipds ratio in medium VLDL (%)	1946	-0.03	-0.15	0.09	0.608
Total cholesterol to total lipids ratio in medium VLDL (%)	1946	-0.04	-0.17	0.09	0.572
Cholesterol esters to total lipids ratio in medium VLDL (%)	1946	-0.02	-0.16	0.11	0.720
Free cholesterol to total lipids ratio in medium VLDL (%)	1946	-0.05	-0.17	0.07	0.389
Triglycerides to total lipids ratio in medium VLDL (%)	1946	0.04	-0.09	0.17	0.558
Phospholipids to total lipds ratio in small VLDL (%)	1946	0.12	0.00	0.23	0.048
Total cholesterol to total lipids ratio in small VLDL (%)	1946	-0.02	-0.14	0.11	0.779
Cholesterol esters to total lipids ratio in small VLDL (%)	1946	-0.03	-0.16	0.10	0.631
Free cholesterol to total lipids ratio in small VLDL (%)	1946	0.08	-0.04	0.20	0.194
Triglycerides to total lipids ratio in small VLDL (%)	1946	-0.01	-0.14	0.11	0.814
Phospholipids to total lipds ratio in very small VLDL (%)	1946	-0.03	-0.16	0.09	0.607
Total cholesterol to total lipids ratio in very small VLDL (%)	1946	0.06	-0.07	0.19	0.370
Cholesterol esters to total lipids ratio in very small VLDL (%)	1946	0.03	-0.10	0.15	0.670
Free cholesterol to total lipids ratio in very small VLDL (%)	1946	0.08	-0.04	0.21	0.198
Triglycerides to total lipids ratio in very small VLDL (%)	1946	-0.05	-0.17	0.08	0.454
Phospholipids to total lipds ratio in IDL (%)	1946	-0.02	-0.15	0.11	0.725
Total cholesterol to total lipids ratio in IDL (%)	1946	0.06	-0.07	0.19	0.392

S10 Table Negative control one-sample MR estimates of associations of age at menarche (per year later) with adiposity and cardiometabolic traits at age 8y among females in ALSPAC, using a full GRS of 351 SNPs for age at menarche

	Unadj.				
**Standardised outcome at age 8y**	**N**	**Beta (2SLS)**	**LCL**	**UCL**	**P-value**
Cholesterol esters to total lipids ratio in IDL (%)	1946	0.04	-0.09	0.18	0.519
Free cholesterol to total lipids ratio in IDL (%)	1946	0.03	-0.09	0.15	0.625
Triglycerides to total lipids ratio in IDL (%)	1946	-0.06	-0.19	0.06	0.322
Phospholipids to total lipds ratio in large LDL (%)	1946	0.01	-0.11	0.13	0.871
Total cholesterol to total lipids ratio in large LDL (%)	1946	0.03	-0.09	0.15	0.606
Cholesterol esters to total lipids ratio in large LDL (%)	1946	0.01	-0.11	0.13	0.888
Free cholesterol to total lipids ratio in large LDL (%)	1946	0.06	-0.06	0.18	0.339
Triglycerides to total lipids ratio in large LDL (%)	1946	-0.06	-0.18	0.07	0.359
Phospholipids to total lipds ratio in medium LDL (%)	1946	0.03	-0.09	0.15	0.581
Total cholesterol to total lipids ratio in medium LDL (%)	1946	0.00	-0.12	0.11	0.956
Cholesterol esters to total lipids ratio in medium LDL (%)	1946	-0.03	-0.15	0.09	0.646
Free cholesterol to total lipids ratio in medium LDL (%)	1946	0.06	-0.06	0.18	0.329
Triglycerides to total lipids ratio in medium LDL (%)	1946	-0.05	-0.18	0.07	0.402
Phospholipids to total lipds ratio in small LDL (%)	1946	0.03	-0.09	0.16	0.588
Total cholesterol to total lipids ratio in small LDL (%)	1946	0.00	-0.12	0.12	0.986
Cholesterol esters to total lipids ratio in small LDL (%)	1946	-0.03	-0.16	0.09	0.586
Free cholesterol to total lipids ratio in small LDL (%)	1946	0.08	-0.05	0.21	0.237
Triglycerides to total lipids ratio in small LDL (%)	1946	-0.07	-0.19	0.05	0.282
Phospholipids to total lipds ratio in very large HDL (%)	1946	-0.01	-0.13	0.11	0.890
Total cholesterol to total lipids ratio in very large HDL (%)	1946	0.03	-0.09	0.15	0.659
Cholesterol esters to total lipids ratio in very large HDL (%)	1946	0.02	-0.10	0.14	0.734
Free cholesterol to total lipids ratio in very large HDL (%)	1946	0.05	-0.07	0.18	0.404
Triglycerides to total lipids ratio in very large HDL (%)	1946	-0.08	-0.20	0.05	0.223
Phospholipids to total lipds ratio in large HDL (%)	1946	-0.09	-0.21	0.04	0.178
Total cholesterol to total lipids ratio in large HDL (%)	1946	0.10	-0.02	0.23	0.111
Cholesterol esters to total lipids ratio in large HDL (%)	1946	0.09	-0.03	0.22	0.148
Free cholesterol to total lipids ratio in large HDL (%)	1946	0.10	-0.02	0.23	0.106
Triglycerides to total lipids ratio in large HDL (%)	1946	-0.10	-0.23	0.03	0.131
Phospholipids to total lipds ratio in medium HDL (%)	1946	0.05	-0.07	0.18	0.405
Total cholesterol to total lipids ratio in medium HDL (%)	1946	0.02	-0.11	0.15	0.747
Cholesterol esters to total lipids ratio in medium HDL (%)	1946	0.02	-0.11	0.15	0.785
Free cholesterol to total lipids ratio in medium HDL (%)	1946	0.03	-0.09	0.16	0.601
Triglycerides to total lipids ratio in medium HDL (%)	1946	-0.09	-0.22	0.03	0.133
Phospholipids to total lipds ratio in small HDL (%)	1946	0.00	-0.12	0.12	0.972
Total cholesterol to total lipids ratio in small HDL (%)	1946	0.02	-0.10	0.14	0.714
Cholesterol esters to total lipids ratio in small HDL (%)	1946	0.00	-0.12	0.12	0.946
Free cholesterol to total lipids ratio in small HDL (%)	1946	0.14	0.01	0.27	0.042
Triglycerides to total lipids ratio in small HDL (%)	1946	-0.09	-0.21	0.03	0.142
Mean diameter for VLDL particles (nm)	1946	-0.01	-0.13	0.10	0.847
Mean diameter for LDL particles (nm)	1946	-0.08	-0.20	0.04	0.184
Mean diameter for HDL particles (nm)	1946	0.04	-0.08	0.16	0.559
Serum total cholesterol (mmol/l)	1946	-0.01	-0.15	0.12	0.843
Total cholesterol in VLDL (mmol/l)	1946	-0.04	-0.17	0.08	0.486
Remnant cholesterol (non-HDL, non-LDL -cholesterol) (mmol/l)	1946	-0.04	-0.17	0.08	0.492
Total cholesterol in LDL (mmol/l)	1946	-0.04	-0.17	0.10	0.583
Total cholesterol in HDL (mmol/l)	1946	0.07	-0.05	0.20	0.231
Total cholesterol in HDL2 (mmol/l)	1946	0.07	-0.05	0.20	0.238
Total cholesterol in HDL3 (mmol/l)	1946	0.07	-0.05	0.19	0.252
Esterified cholesterol (mmol/l)	1946	0.00	-0.13	0.14	0.969
Free cholesterol (mmol/l)	1946	-0.05	-0.18	0.08	0.456
Serum total triglycerides (mmol/l)	1946	-0.05	-0.17	0.07	0.425
Triglycerides in VLDL (mmol/l)	1946	-0.04	-0.16	0.08	0.526
Triglycerides in LDL (mmol/l)	1946	-0.07	-0.21	0.07	0.347
Triglycerides in HDL (mmol/l)	1946	-0.06	-0.18	0.06	0.344
Diacylglycerol (mmol/l)	1908	-0.02	-0.15	0.11	0.771
Ratio of diacylglycerol to triglycerides	1908	0.00	-0.13	0.13	0.963
Total phosphoglycerides (mmol/l)	1946	0.04	-0.09	0.17	0.551
Ratio of triglycerides to phosphoglycerides	1946	-0.04	-0.16	0.08	0.515
Phosphatidylcholine and other cholines (mmol/l)	1940	0.06	-0.07	0.19	0.347
Total cholines (mmol/l)	1946	0.04	-0.09	0.17	0.577
Apolipoprotein A-I (g/l)	1946	0.05	-0.07	0.18	0.429
Apolipoprotein B (g/l)	1946	-0.06	-0.19	0.06	0.317
Ratio of apolipoprotein B to apolipoprotein A-I	1946	-0.09	-0.21	0.03	0.160
Total fatty acids (mmol/l)	1946	0.00	-0.13	0.13	0.988
Estimated description of fatty acid chain length, not actual carbon number	1946	-0.02	-0.14	0.10	0.727
Estimated degree of unsaturation	1946	-0.01	-0.13	0.11	0.897
22:6, docosahexaenoic acid (mmol/l)	1946	0.01	-0.13	0.14	0.908
18:2, linoleic acid (mmol/l)	1946	0.02	-0.11	0.15	0.793

S10 Table Negative control one-sample MR estimates of associations of age at menarche (per year later) with adiposity and cardiometabolic traits at age 8y among females in ALSPAC, using a full GRS of 351 SNPs for age at menarche

	Unadj.				
**Standardised outcome at age 8y**	**N**	**Beta (2SLS)**	**LCL**	**UCL**	**P-value**
Conjugated linoleic acid (mmol/l)	1945	0.05	-0.08	0.18	0.434
Omega-3 fatty acids (mmol/l)	1946	0.02	-0.11	0.16	0.710
Omega-6 fatty acids (mmol/l)	1946	0.01	-0.12	0.14	0.898
Polyunsaturated fatty acids (mmol/l)	1946	0.01	-0.12	0.14	0.869
Monounsaturated fatty acids; 16:1, 18:1 (mmol/l)	1946	-0.02	-0.14	0.10	0.768
Saturated fatty acids (mmol/l)	1946	0.00	-0.12	0.13	0.944
Ratio of 22:6 docosahexaenoic acid to total fatty acids (%)	1946	0.01	-0.11	0.13	0.884
Ratio of 18:2 linoleic acid to total fatty acids (%)	1946	0.02	-0.10	0.14	0.730
Ratio of conjugated linoleic acid to total fatty acids (%)	1945	0.06	-0.06	0.18	0.339
Ratio of omega-3 fatty acids to total fatty acids (%)	1946	0.03	-0.09	0.15	0.661
Ratio of omega-6 fatty acids to total fatty acids (%)	1946	0.01	-0.11	0.13	0.923
Ratio of polyunsaturated fatty acids to total fatty acids (%)	1946	0.01	-0.11	0.13	0.861
Ratio of monounsaturated fatty acids to total fatty acids (%)	1946	-0.03	-0.15	0.10	0.673
Ratio of saturated fatty acids to total fatty acids (%)	1946	0.02	-0.10	0.14	0.709
Glucose (mmol/l)	1936	-0.02	-0.14	0.10	0.762
Lactate (mmol/l)	1947	0.09	-0.04	0.23	0.190
Pyruvate (mmol/l)	1942	0.04	-0.10	0.18	0.603
Citrate (mmol/l)	1942	0.14	0.01	0.27	0.033
Alanine (mmol/l)	1947	0.06	-0.06	0.18	0.326
Glutamine (mmol/l)	1942	0.12	-0.01	0.25	0.060
Histidine (mmol/l)	1943	0.05	-0.07	0.16	0.447
Isoleucine (mmol/l)	1946	0.06	-0.06	0.19	0.335
Leucine (mmol/l)	1947	0.04	-0.08	0.17	0.501
Valine (mmol/l)	1947	0.06	-0.06	0.18	0.324
Phenylalanine (mmol/l)	1941	0.05	-0.08	0.17	0.456
Tyrosine (mmol/l)	1940	0.05	-0.07	0.17	0.395
Acetate (mmol/l)	1947	-0.01	-0.13	0.10	0.826
Acetoacetate (mmol/l)	1943	0.01	-0.11	0.12	0.881
3-hydroxybutyrate (mmol/l)	1945	0.02	-0.10	0.14	0.722
Creatinine (mmol/l)	1943	-0.01	-0.13	0.12	0.925
Albumin (signal area)	1941	-0.08	-0.20	0.04	0.198
Glycoprotein acetyls, mainly a1-acid glycoprotein (mmol/l)	1947	-0.16	-0.30	-0.03	0.013

Complete case sample

	Unadj.				
**Standardised outcome at age 8y**	**N**	**Beta (2SLS)**	**LCL**	**UCL**	**P-value**
Body mass index (kg/m^2)	629	-0.27	-0.44	-0.10	1.89E-03
Fat mass index (kg/m^2)	629	-0.26	-0.43	-0.09	0.002
Lean mass index (kg/m^2)	629	-0.37	-0.54	-0.19	3.49E-05
Systolic blood pressure (mmHg)	629	-0.14	-0.33	0.05	0.143
Diastolic blood pressure (mmHg)	629	-0.15	-0.33	0.03	0.098
Concentration of chylomicrons and extremely large VLDL particles (mol/l)	629	0.01	-0.17	0.19	0.901
Total lipids in chylomicrons and extremely large VLDL (mmol/l)	629	0.01	-0.16	0.19	0.871
Phospholipids in chylomicrons and extremely large VLDL (mmol/l)	629	0.02	-0.16	0.19	0.868
Total cholesterol in chylomicrons and extremely large VLDL (mmol/l)	629	0.02	-0.16	0.20	0.858
Cholesterol esters in chylomicrons and extremely large VLDL (mmol/l)	629	0.02	-0.17	0.20	0.863
Free cholesterol in chylomicrons and extremely large VLDL (mmol/l)	629	0.02	-0.16	0.20	0.861
Triglycerides in chylomicrons and extremely large VLDL (mmol/l)	629	0.01	-0.16	0.19	0.874
Concentration of very large VLDL particles (mol/l)	629	0.02	-0.16	0.20	0.843
Total lipids in very large VLDL (mmol/l)	629	0.02	-0.16	0.20	0.840
Phospholipids in very large VLDL (mmol/l)	629	0.02	-0.16	0.20	0.842
Total cholesterol in very large VLDL (mmol/l)	629	0.01	-0.17	0.19	0.879
Cholesterol esters in very large VLDL (mmol/l)	629	0.01	-0.17	0.19	0.885
Free cholesterol in very large VLDL (mmol/l)	629	0.01	-0.17	0.19	0.875
Triglycerides in very large VLDL (mmol/l)	629	0.02	-0.16	0.20	0.828
Concentration of large VLDL particles (mol/l)	629	0.02	-0.16	0.19	0.858
Total lipids in large VLDL (mmol/l)	629	0.02	-0.16	0.19	0.859
Phospholipids in large VLDL (mmol/l)	629	0.02	-0.16	0.19	0.860
Total cholesterol in large VLDL (mmol/l)	629	0.02	-0.16	0.19	0.867
Cholesterol esters in large VLDL (mmol/l)	629	0.01	-0.17	0.19	0.899
Free cholesterol in large VLDL (mmol/l)	629	0.02	-0.16	0.20	0.842
Triglycerides in large VLDL (mmol/l)	629	0.02	-0.16	0.19	0.857
Concentration of medium VLDL particles (mol/l)	629	0.01	-0.17	0.18	0.954
Total lipids in medium VLDL (mmol/l)	629	0.01	-0.17	0.18	0.949
Phospholipids in medium VLDL (mmol/l)	629	0.00	-0.17	0.18	0.966
Total cholesterol in medium VLDL (mmol/l)	629	0.01	-0.18	0.19	0.941

S10 Table Negative control one-sample MR estimates of associations of age at menarche (per year later) with adiposity and cardiometabolic traits at age 8y among females in ALSPAC, using a full GRS of 351 SNPs for age at menarche

	Unadj.				
**Standardised outcome at age 8y**	**N**	**Beta (2SLS)**	**LCL**	**UCL**	**P-value**
Cholesterol esters in medium VLDL (mmol/l)	629	0.01	-0.18	0.20	0.926
Free cholesterol in medium VLDL (mmol/l)	629	0.00	-0.17	0.18	0.963
Triglycerides in medium VLDL (mmol/l)	629	0.01	-0.17	0.18	0.948
Concentration of small VLDL particles (mol/l)	629	-0.01	-0.19	0.16	0.871
Total lipids in small VLDL (mmol/l)	629	-0.02	-0.20	0.16	0.809
Phospholipids in small VLDL (mmol/l)	629	-0.01	-0.19	0.17	0.877
Total cholesterol in small VLDL (mmol/l)	629	-0.04	-0.23	0.15	0.701
Cholesterol esters in small VLDL (mmol/l)	629	-0.04	-0.24	0.15	0.661
Free cholesterol in small VLDL (mmol/l)	629	-0.02	-0.20	0.16	0.821
Triglycerides in small VLDL (mmol/l)	629	-0.01	-0.19	0.17	0.900
Concentration of very small VLDL particles (mol/l)	629	-0.05	-0.24	0.15	0.632
Total lipids in very small VLDL (mmol/l)	629	-0.05	-0.25	0.15	0.601
Phospholipids in very small VLDL (mmol/l)	629	-0.04	-0.24	0.16	0.680
Total cholesterol in very small VLDL (mmol/l)	629	-0.05	-0.25	0.15	0.626
Cholesterol esters in very small VLDL (mmol/l)	629	-0.05	-0.25	0.15	0.633
Free cholesterol in very small VLDL (mmol/l)	629	-0.05	-0.25	0.15	0.642
Triglycerides in very small VLDL (mmol/l)	629	-0.03	-0.21	0.15	0.730
Concentration of IDL particles (mol/l)	629	-0.03	-0.23	0.16	0.752
Total lipids in IDL (mmol/l)	629	-0.02	-0.22	0.17	0.805
Phospholipids in IDL (mmol/l)	629	-0.02	-0.21	0.18	0.865
Total cholesterol in IDL (mmol/l)	629	-0.02	-0.22	0.18	0.837
Cholesterol esters in IDL (mmol/l)	629	-0.02	-0.22	0.18	0.834
Free cholesterol in IDL (mmol/l)	629	-0.02	-0.21	0.18	0.852
Triglycerides in IDL (mmol/l)	629	-0.05	-0.23	0.13	0.591
Concentration of large LDL particles (mol/l)	629	-0.02	-0.22	0.18	0.861
Total lipids in large LDL (mmol/l)	629	-0.01	-0.21	0.18	0.891
Phospholipids in large LDL (mmol/l)	629	-0.01	-0.21	0.19	0.923
Total cholesterol in large LDL (mmol/l)	629	-0.01	-0.21	0.19	0.911
Cholesterol esters in large LDL (mmol/l)	629	-0.01	-0.21	0.19	0.925
Free cholesterol in large LDL (mmol/l)	629	-0.02	-0.21	0.18	0.874
Triglycerides in large LDL (mmol/l)	629	-0.04	-0.22	0.15	0.703
Concentration of medium LDL particles (mol/l)	629	-0.01	-0.21	0.19	0.894
Total lipids in medium LDL (mmol/l)	629	-0.01	-0.21	0.18	0.889
Phospholipids in medium LDL (mmol/l)	629	0.00	-0.20	0.19	0.966
Total cholesterol in medium LDL (mmol/l)	629	-0.01	-0.21	0.18	0.890
Cholesterol esters in medium LDL (mmol/l)	629	-0.01	-0.21	0.19	0.897
Free cholesterol in medium LDL (mmol/l)	629	-0.02	-0.22	0.18	0.867
Triglycerides in medium LDL (mmol/l)	629	-0.03	-0.22	0.17	0.796
Concentration of small LDL particles (mol/l)	629	-0.01	-0.21	0.19	0.934
Total lipids in small LDL (mmol/l)	629	-0.01	-0.21	0.19	0.915
Phospholipids in small LDL (mmol/l)	629	0.00	-0.19	0.20	0.982
Total cholesterol in small LDL (mmol/l)	629	-0.01	-0.21	0.19	0.895
Cholesterol esters in small LDL (mmol/l)	629	-0.01	-0.21	0.19	0.907
Free cholesterol in small LDL (mmol/l)	629	-0.02	-0.22	0.18	0.857
Triglycerides in small LDL (mmol/l)	629	-0.01	-0.20	0.18	0.907
Concentration of very large HDL particles (mol/l)	629	0.03	-0.15	0.20	0.766
Total lipids in very large HDL (mmol/l)	629	0.02	-0.16	0.20	0.818
Phospholipids in very large HDL (mmol/l)	629	0.03	-0.14	0.21	0.703
Total cholesterol in very large HDL (mmol/l)	629	0.01	-0.18	0.19	0.952
Cholesterol esters in very large HDL (mmol/l)	629	0.00	-0.19	0.18	0.992
Free cholesterol in very large HDL (mmol/l)	629	0.02	-0.16	0.20	0.814
Triglycerides in very large HDL (mmol/l)	629	0.00	-0.18	0.18	0.993
Concentration of large HDL particles (mol/l)	629	0.07	-0.11	0.25	0.433
Total lipids in large HDL (mmol/l)	629	0.07	-0.11	0.24	0.454
Phospholipids in large HDL (mmol/l)	629	0.08	-0.10	0.25	0.390
Total cholesterol in large HDL (mmol/l)	629	0.06	-0.12	0.24	0.530
Cholesterol esters in large HDL (mmol/l)	629	0.06	-0.12	0.24	0.529
Free cholesterol in large HDL (mmol/l)	629	0.06	-0.12	0.24	0.535
Triglycerides in large HDL (mmol/l)	629	0.08	-0.10	0.27	0.366
Concentration of medium HDL particles (mol/l)	629	0.14	-0.04	0.32	0.121
Total lipids in medium HDL (mmol/l)	629	0.14	-0.04	0.32	0.127
Phospholipids in medium HDL (mmol/l)	629	0.12	-0.06	0.30	0.202
Total cholesterol in medium HDL (mmol/l)	629	0.15	-0.03	0.33	0.111
Cholesterol esters in medium HDL (mmol/l)	629	0.15	-0.03	0.33	0.110
Free cholesterol in medium HDL (mmol/l)	629	0.14	-0.04	0.32	0.139
Triglycerides in medium HDL (mmol/l)	629	0.05	-0.13	0.24	0.570
Concentration of small HDL particles (mol/l)	629	0.11	-0.07	0.29	0.229
Total lipids in small HDL (mmol/l)	629	0.10	-0.08	0.28	0.277

S10 Table Negative control one-sample MR estimates of associations of age at menarche (per year later) with adiposity and cardiometabolic traits at age 8y among females in ALSPAC, using a full GRS of 351 SNPs for age at menarche

	Unadj.				
**Standardised outcome at age 8y**	**N**	**Beta (2SLS)**	**LCL**	**UCL**	**P-value**
Phospholipids in small HDL (mmol/l)	629	0.12	-0.06	0.30	0.198
Total cholesterol in small HDL (mmol/l)	629	0.04	-0.15	0.22	0.700
Cholesterol esters in small HDL (mmol/l)	629	0.02	-0.17	0.21	0.863
Free cholesterol in small HDL (mmol/l)	629	0.10	-0.08	0.28	0.258
Triglycerides in small HDL (mmol/l)	629	0.00	-0.18	0.18	0.979
Phospholipids to total lipds ratio in chylomicrons and extremely large VLDL (%)	629	0.02	-0.09	0.14	0.698
Total cholesterol to total lipids ratio in chylomicrons and extremely large VLDL (%)	629	0.06	-0.14	0.26	0.566
Cholesterol esters to total lipids ratio in chylomicrons and extremely large VLDL (%)	629	0.05	-0.16	0.26	0.650
Free cholesterol to total lipids ratio in chylomicrons and extremely large VLDL (%)	629	0.05	-0.11	0.21	0.534
Triglycerides to total lipids ratio in chylomicrons and extremely large VLDL (%)	629	-0.02	-0.09	0.04	0.482
Phospholipids to total lipds ratio in very large VLDL (%)	629	0.06	-0.10	0.22	0.462
Total cholesterol to total lipids ratio in very large VLDL (%)	629	0.01	-0.02	0.05	0.449
Cholesterol esters to total lipids ratio in very large VLDL (%)	629	0.03	-0.08	0.14	0.546
Free cholesterol to total lipids ratio in very large VLDL (%)	629	0.03	-0.04	0.10	0.377
Triglycerides to total lipids ratio in very large VLDL (%)	629	-0.08	-0.23	0.07	0.321
Phospholipids to total lipds ratio in large VLDL (%)	629	-0.01	-0.14	0.12	0.842
Total cholesterol to total lipids ratio in large VLDL (%)	629	0.03	-0.11	0.18	0.644
Cholesterol esters to total lipids ratio in large VLDL (%)	629	0.03	-0.06	0.11	0.582
Free cholesterol to total lipids ratio in large VLDL (%)	629	0.00	-0.06	0.05	0.961
Triglycerides to total lipids ratio in large VLDL (%)	629	-0.01	-0.11	0.09	0.781
Phospholipids to total lipds ratio in medium VLDL (%)	629	0.00	-0.17	0.17	0.993
Total cholesterol to total lipids ratio in medium VLDL (%)	629	0.00	-0.18	0.19	0.978
Cholesterol esters to total lipids ratio in medium VLDL (%)	629	0.00	-0.19	0.20	0.964
Free cholesterol to total lipids ratio in medium VLDL (%)	629	-0.01	-0.16	0.14	0.934
Triglycerides to total lipids ratio in medium VLDL (%)	629	0.00	-0.19	0.18	0.981
Phospholipids to total lipds ratio in small VLDL (%)	629	0.06	-0.12	0.24	0.490
Total cholesterol to total lipids ratio in small VLDL (%)	629	-0.03	-0.22	0.15	0.748
Cholesterol esters to total lipids ratio in small VLDL (%)	629	-0.04	-0.22	0.15	0.694
Free cholesterol to total lipids ratio in small VLDL (%)	629	0.03	-0.15	0.21	0.746
Triglycerides to total lipids ratio in small VLDL (%)	629	0.01	-0.17	0.19	0.903
Phospholipids to total lipds ratio in very small VLDL (%)	629	0.02	-0.17	0.20	0.860
Total cholesterol to total lipids ratio in very small VLDL (%)	629	0.02	-0.17	0.21	0.866
Cholesterol esters to total lipids ratio in very small VLDL (%)	629	0.02	-0.16	0.20	0.857
Free cholesterol to total lipids ratio in very small VLDL (%)	629	0.01	-0.18	0.20	0.921
Triglycerides to total lipids ratio in very small VLDL (%)	629	-0.03	-0.22	0.16	0.789
Phospholipids to total lipds ratio in IDL (%)	629	0.01	-0.19	0.21	0.941
Total cholesterol to total lipids ratio in IDL (%)	629	0.05	-0.15	0.24	0.644
Cholesterol esters to total lipids ratio in IDL (%)	629	0.03	-0.16	0.22	0.734
Free cholesterol to total lipids ratio in IDL (%)	629	0.03	-0.14	0.20	0.760
Triglycerides to total lipids ratio in IDL (%)	629	-0.06	-0.25	0.12	0.493
Phospholipids to total lipds ratio in large LDL (%)	629	0.02	-0.15	0.20	0.807
Total cholesterol to total lipids ratio in large LDL (%)	629	0.03	-0.16	0.21	0.780
Cholesterol esters to total lipids ratio in large LDL (%)	629	0.02	-0.16	0.20	0.836
Free cholesterol to total lipids ratio in large LDL (%)	629	0.01	-0.16	0.18	0.870
Triglycerides to total lipids ratio in large LDL (%)	629	-0.05	-0.24	0.13	0.553
Phospholipids to total lipds ratio in medium LDL (%)	629	0.03	-0.15	0.21	0.758
Total cholesterol to total lipids ratio in medium LDL (%)	629	0.00	-0.18	0.18	1.000
Cholesterol esters to total lipids ratio in medium LDL (%)	629	-0.01	-0.19	0.17	0.931
Free cholesterol to total lipids ratio in medium LDL (%)	629	0.02	-0.16	0.20	0.839
Triglycerides to total lipids ratio in medium LDL (%)	629	-0.05	-0.24	0.13	0.570
Phospholipids to total lipds ratio in small LDL (%)	629	0.03	-0.16	0.21	0.779
Total cholesterol to total lipids ratio in small LDL (%)	629	-0.01	-0.19	0.17	0.907
Cholesterol esters to total lipids ratio in small LDL (%)	629	-0.01	-0.20	0.17	0.895
Free cholesterol to total lipids ratio in small LDL (%)	629	0.01	-0.18	0.20	0.903
Triglycerides to total lipids ratio in small LDL (%)	629	-0.03	-0.21	0.15	0.755
Phospholipids to total lipds ratio in very large HDL (%)	629	0.02	-0.15	0.19	0.832
Total cholesterol to total lipids ratio in very large HDL (%)	629	-0.02	-0.19	0.15	0.824
Cholesterol esters to total lipids ratio in very large HDL (%)	629	-0.02	-0.19	0.15	0.838
Free cholesterol to total lipids ratio in very large HDL (%)	629	-0.01	-0.20	0.18	0.917
Triglycerides to total lipids ratio in very large HDL (%)	629	0.00	-0.19	0.19	0.979
Phospholipids to total lipds ratio in large HDL (%)	629	0.04	-0.15	0.23	0.659
Total cholesterol to total lipids ratio in large HDL (%)	629	-0.03	-0.22	0.15	0.715
Cholesterol esters to total lipids ratio in large HDL (%)	629	-0.03	-0.22	0.15	0.715
Free cholesterol to total lipids ratio in large HDL (%)	629	-0.03	-0.21	0.16	0.781
Triglycerides to total lipids ratio in large HDL (%)	629	0.01	-0.18	0.19	0.926
Phospholipids to total lipds ratio in medium HDL (%)	629	0.00	-0.19	0.19	0.987
Total cholesterol to total lipids ratio in medium HDL (%)	629	0.00	-0.19	0.19	0.995
Cholesterol esters to total lipids ratio in medium HDL (%)	629	0.00	-0.19	0.19	0.973

S10 Table Negative control one-sample MR estimates of associations of age at menarche (per year later) with adiposity and cardiometabolic traits at age 8y among females in ALSPAC, using a full GRS of 351 SNPs for age at menarche

	Unadj.				
**Standardised outcome at age 8y**	**N**	**Beta (2SLS)**	**LCL**	**UCL**	**P-value**
Free cholesterol to total lipids ratio in medium HDL (%)	629	0.01	-0.18	0.21	0.905
Triglycerides to total lipids ratio in medium HDL (%)	629	0.00	-0.19	0.19	0.972
Phospholipids to total lipds ratio in small HDL (%)	629	0.03	-0.16	0.23	0.727
Total cholesterol to total lipids ratio in small HDL (%)	629	-0.02	-0.21	0.17	0.832
Cholesterol esters to total lipids ratio in small HDL (%)	629	-0.02	-0.22	0.17	0.813
Free cholesterol to total lipids ratio in small HDL (%)	629	0.03	-0.17	0.23	0.784
Triglycerides to total lipids ratio in small HDL (%)	629	-0.03	-0.22	0.15	0.722
Mean diameter for VLDL particles (nm)	629	0.02	-0.15	0.20	0.798
Mean diameter for LDL particles (nm)	629	-0.04	-0.22	0.14	0.636
Mean diameter for HDL particles (nm)	629	0.03	-0.15	0.21	0.735
Serum total cholesterol (mmol/l)	629	0.01	-0.19	0.20	0.946
Total cholesterol in VLDL (mmol/l)	629	-0.02	-0.21	0.17	0.855
Remnant cholesterol (non-HDL, non-LDL -cholesterol) (mmol/l)	629	-0.02	-0.22	0.18	0.830
Total cholesterol in LDL (mmol/l)	629	-0.01	-0.21	0.19	0.901
Total cholesterol in HDL (mmol/l)	629	0.07	-0.12	0.25	0.483
Total cholesterol in HDL2 (mmol/l)	629	0.06	-0.12	0.25	0.492
Total cholesterol in HDL3 (mmol/l)	629	0.06	-0.12	0.25	0.495
Esterified cholesterol (mmol/l)	629	0.03	-0.17	0.23	0.751
Free cholesterol (mmol/l)	629	-0.05	-0.25	0.14	0.596
Serum total triglycerides (mmol/l)	629	0.00	-0.18	0.18	0.995
Triglycerides in VLDL (mmol/l)	629	0.01	-0.17	0.18	0.950
Triglycerides in LDL (mmol/l)	629	-0.03	-0.22	0.16	0.771
Triglycerides in HDL (mmol/l)	629	0.03	-0.15	0.22	0.719
Diacylglycerol (mmol/l)	629	0.03	-0.17	0.22	0.796
Ratio of diacylglycerol to triglycerides	629	0.01	-0.19	0.22	0.893
Total phosphoglycerides (mmol/l)	629	0.03	-0.16	0.22	0.781
Ratio of triglycerides to phosphoglycerides	629	0.05	-0.13	0.22	0.602
Phosphatidylcholine and other cholines (mmol/l)	629	0.09	-0.10	0.28	0.370
Total cholines (mmol/l)	629	0.03	-0.16	0.21	0.764
Apolipoprotein A-I (g/l)	629	0.07	-0.11	0.25	0.460
Apolipoprotein B (g/l)	629	-0.02	-0.21	0.17	0.812
Ratio of apolipoprotein B to apolipoprotein A-I	629	-0.05	-0.24	0.14	0.589
Total fatty acids (mmol/l)	629	0.03	-0.16	0.21	0.781
Estimated description of fatty acid chain length, not actual carbon number	629	0.01	-0.17	0.19	0.911
Estimated degree of unsaturation	629	0.09	-0.10	0.27	0.343
22:6, docosahexaenoic acid (mmol/l)	629	0.09	-0.09	0.27	0.341
18:2, linoleic acid (mmol/l)	629	0.04	-0.15	0.22	0.705
Conjugated linoleic acid (mmol/l)	629	0.08	-0.13	0.29	0.473
Omega-3 fatty acids (mmol/l)	629	0.15	-0.03	0.33	0.108
Omega-6 fatty acids (mmol/l)	629	0.04	-0.15	0.23	0.668
Polyunsaturated fatty acids (mmol/l)	629	0.06	-0.13	0.25	0.555
Monounsaturated fatty acids; 16:1, 18:1 (mmol/l)	629	0.00	-0.18	0.18	0.970
Saturated fatty acids (mmol/l)	629	0.02	-0.17	0.21	0.874
Ratio of 22:6 docosahexaenoic acid to total fatty acids (%)	629	0.11	-0.07	0.29	0.242
Ratio of 18:2 linoleic acid to total fatty acids (%)	629	0.03	-0.14	0.20	0.702
Ratio of conjugated linoleic acid to total fatty acids (%)	629	0.06	-0.13	0.26	0.529
Ratio of omega-3 fatty acids to total fatty acids (%)	629	0.17	0.00	0.34	0.052
Ratio of omega-6 fatty acids to total fatty acids (%)	629	0.03	-0.15	0.21	0.709
Ratio of polyunsaturated fatty acids to total fatty acids (%)	629	0.07	-0.12	0.25	0.479
Ratio of monounsaturated fatty acids to total fatty acids (%)	629	-0.04	-0.21	0.12	0.616
Ratio of saturated fatty acids to total fatty acids (%)	629	-0.04	-0.23	0.16	0.716
Glucose (mmol/l)	629	0.00	-0.18	0.17	0.966
Lactate (mmol/l)	629	0.04	-0.16	0.25	0.673
Pyruvate (mmol/l)	629	0.06	-0.16	0.28	0.607
Citrate (mmol/l)	629	0.00	-0.18	0.19	0.988
Alanine (mmol/l)	629	0.03	-0.16	0.22	0.736
Glutamine (mmol/l)	629	0.06	-0.11	0.24	0.480
Histidine (mmol/l)	629	0.00	-0.16	0.15	0.951
Isoleucine (mmol/l)	629	0.22	0.03	0.41	0.027
Leucine (mmol/l)	629	0.15	-0.03	0.34	0.100
Valine (mmol/l)	629	0.21	0.03	0.38	0.021
Phenylalanine (mmol/l)	629	0.20	0.01	0.39	0.042
Tyrosine (mmol/l)	629	0.12	-0.06	0.30	0.188
Acetate (mmol/l)	629	0.11	-0.08	0.30	0.272
Acetoacetate (mmol/l)	629	-0.03	-0.18	0.13	0.756
3-hydroxybutyrate (mmol/l)	629	0.01	-0.15	0.17	0.902
Creatinine (mmol/l)	629	-0.09	-0.24	0.07	0.286
Albumin (signal area)	629	-0.05	-0.22	0.12	0.578

S10 Table Negative control one-sample MR estimates of associations of age at menarche (per year later) with adiposity and cardiometabolic traits at age 8y among females in ALSPAC, using a full GRS of 351 SNPs for age at menarche

	Unadj.				
**Standardised outcome at age 8y**	**N**	**Beta (2SLS)**	**LCL**	**UCL**	**P-value**
Glycoprotein acetyls, mainly a1-acid glycoprotein (mmol/l)	629	0.05	-0.13	0.23	0.568
