# Supplementary material for: Influence of puberty timing on adiposity and cardiometabolic traits: A Mendelian randomisation study
Source: PLoS Med. 2018 Aug 28;15(8):e1002641. doi: 10.1371/journal.pmed.1002641 (PMC6112630; doi:10.1371/journal.pmed.1002641)
Supplement: S11 Table — (PDF) [file pmed.1002641.s030.pdf]

S11 Table Negative control one-sample MR estimates of associations of puberty timing (per year later) with adiposity and cardiometabolic traits at age 8y among males and females in ALSPAC, using a full GRS of 351 SNPs for age at menarche

	Unadj.				
**Standardised outcome at age 8y**	**N**	**Beta (2SLS)**	**LCL**	**UCL**	**P-value**
Body mass index (kg/m^2)	4795	-0.45	-0.59	-0.32	3.85E-11
Fat mass index (kg/m^2)	4590	-0.31	-0.43	-0.19	6.90E-07
Lean mass index (kg/m^2)	4590	-0.54	-0.70	-0.39	5.17E-12
Systolic blood pressure (mmHg)	4733	-0.27	-0.40	-0.13	7.76E-05
Diastolic blood pressure (mmHg)	4732	-0.21	-0.34	-0.07	0.002
Concentration of chylomicrons and extremely large VLDL particles (mol/l)	3543	-0.03	-0.18	0.11	0.646
Total lipids in chylomicrons and extremely large VLDL (mmol/l)	3543	-0.03	-0.18	0.11	0.664
Phospholipids in chylomicrons and extremely large VLDL (mmol/l)	3543	-0.03	-0.18	0.11	0.673
Total cholesterol in chylomicrons and extremely large VLDL (mmol/l)	3543	-0.03	-0.17	0.12	0.707
Cholesterol esters in chylomicrons and extremely large VLDL (mmol/l)	3543	-0.02	-0.17	0.13	0.792
Free cholesterol in chylomicrons and extremely large VLDL (mmol/l)	3543	-0.03	-0.18	0.11	0.644
Triglycerides in chylomicrons and extremely large VLDL (mmol/l)	3543	-0.03	-0.18	0.11	0.655
Concentration of very large VLDL particles (mol/l)	3543	-0.04	-0.18	0.10	0.594
Total lipids in very large VLDL (mmol/l)	3543	-0.04	-0.18	0.11	0.606
Phospholipids in very large VLDL (mmol/l)	3543	-0.03	-0.18	0.11	0.670
Total cholesterol in very large VLDL (mmol/l)	3543	-0.03	-0.17	0.11	0.683
Cholesterol esters in very large VLDL (mmol/l)	3543	-0.03	-0.17	0.11	0.681
Free cholesterol in very large VLDL (mmol/l)	3543	-0.03	-0.17	0.12	0.689
Triglycerides in very large VLDL (mmol/l)	3543	-0.04	-0.19	0.10	0.569
Concentration of large VLDL particles (mol/l)	3543	-0.05	-0.19	0.10	0.524
Total lipids in large VLDL (mmol/l)	3543	-0.04	-0.19	0.10	0.554
Phospholipids in large VLDL (mmol/l)	3543	-0.04	-0.19	0.10	0.578
Total cholesterol in large VLDL (mmol/l)	3543	-0.03	-0.18	0.11	0.680
Cholesterol esters in large VLDL (mmol/l)	3543	-0.03	-0.17	0.12	0.723
Free cholesterol in large VLDL (mmol/l)	3543	-0.03	-0.18	0.11	0.647
Triglycerides in large VLDL (mmol/l)	3543	-0.05	-0.19	0.10	0.504
Concentration of medium VLDL particles (mol/l)	3543	-0.05	-0.20	0.09	0.489
Total lipids in medium VLDL (mmol/l)	3543	-0.05	-0.19	0.10	0.538
Phospholipids in medium VLDL (mmol/l)	3543	-0.04	-0.19	0.11	0.588
Total cholesterol in medium VLDL (mmol/l)	3543	-0.01	-0.16	0.14	0.879
Cholesterol esters in medium VLDL (mmol/l)	3543	0.02	-0.14	0.17	0.837
Free cholesterol in medium VLDL (mmol/l)	3543	-0.04	-0.19	0.11	0.585
Triglycerides in medium VLDL (mmol/l)	3543	-0.06	-0.21	0.08	0.399
Concentration of small VLDL particles (mol/l)	3543	-0.03	-0.18	0.12	0.663
Total lipids in small VLDL (mmol/l)	3543	-0.02	-0.17	0.13	0.820
Phospholipids in small VLDL (mmol/l)	3543	0.00	-0.15	0.15	0.971
Total cholesterol in small VLDL (mmol/l)	3543	0.03	-0.12	0.19	0.683
Cholesterol esters in small VLDL (mmol/l)	3543	0.04	-0.11	0.20	0.599
Free cholesterol in small VLDL (mmol/l)	3543	0.01	-0.14	0.16	0.876
Triglycerides in small VLDL (mmol/l)	3543	-0.06	-0.21	0.09	0.450
Concentration of very small VLDL particles (mol/l)	3543	0.09	-0.06	0.25	0.246
Total lipids in very small VLDL (mmol/l)	3543	0.10	-0.06	0.26	0.224
Phospholipids in very small VLDL (mmol/l)	3543	0.11	-0.05	0.27	0.178
Total cholesterol in very small VLDL (mmol/l)	3543	0.11	-0.05	0.27	0.178
Cholesterol esters in very small VLDL (mmol/l)	3543	0.09	-0.07	0.24	0.265
Free cholesterol in very small VLDL (mmol/l)	3543	0.13	-0.03	0.29	0.099
Triglycerides in very small VLDL (mmol/l)	3543	-0.02	-0.17	0.14	0.824
Concentration of IDL particles (mol/l)	3543	0.11	-0.06	0.27	0.200
Total lipids in IDL (mmol/l)	3543	0.13	-0.03	0.30	0.105
Phospholipids in IDL (mmol/l)	3543	0.11	-0.05	0.27	0.176
Total cholesterol in IDL (mmol/l)	3543	0.15	-0.01	0.31	0.070
Cholesterol esters in IDL (mmol/l)	3543	0.15	-0.01	0.31	0.067
Free cholesterol in IDL (mmol/l)	3543	0.13	-0.03	0.30	0.100
Triglycerides in IDL (mmol/l)	3543	0.02	-0.14	0.18	0.820
Concentration of large LDL particles (mol/l)	3543	0.10	-0.07	0.26	0.249
Total lipids in large LDL (mmol/l)	3543	0.13	-0.04	0.29	0.133
Phospholipids in large LDL (mmol/l)	3543	0.12	-0.04	0.29	0.135
Total cholesterol in large LDL (mmol/l)	3543	0.13	-0.03	0.30	0.105
Cholesterol esters in large LDL (mmol/l)	3543	0.13	-0.03	0.29	0.112
Free cholesterol in large LDL (mmol/l)	3543	0.14	-0.02	0.30	0.089
Triglycerides in large LDL (mmol/l)	3543	0.02	-0.15	0.18	0.845
Concentration of medium LDL particles (mol/l)	3543	0.07	-0.09	0.24	0.372
Total lipids in medium LDL (mmol/l)	3543	0.11	-0.05	0.27	0.185
Phospholipids in medium LDL (mmol/l)	3543	0.15	-0.01	0.31	0.074
Total cholesterol in medium LDL (mmol/l)	3543	0.11	-0.05	0.27	0.177
Cholesterol esters in medium LDL (mmol/l)	3543	0.10	-0.07	0.26	0.245
Free cholesterol in medium LDL (mmol/l)	3543	0.18	0.01	0.34	0.034

S11 Table Negative control one-sample MR estimates of associations of puberty timing (per year later) with adiposity and cardiometabolic traits at age 8y among males and females in ALSPAC, using a full GRS of 351 SNPs for age at menarche

	Unadj.				
**Standardised outcome at age 8y**	**N**	**Beta (2SLS)**	**LCL**	**UCL**	**P-value**
Triglycerides in medium LDL (mmol/l)	3543	-0.01	-0.17	0.16	0.926
Concentration of small LDL particles (mol/l)	3543	0.10	-0.07	0.26	0.248
Total lipids in small LDL (mmol/l)	3543	0.12	-0.05	0.28	0.165
Phospholipids in small LDL (mmol/l)	3543	0.15	-0.01	0.32	0.068
Total cholesterol in small LDL (mmol/l)	3543	0.12	-0.05	0.28	0.158
Cholesterol esters in small LDL (mmol/l)	3543	0.09	-0.07	0.25	0.266
Free cholesterol in small LDL (mmol/l)	3543	0.21	0.04	0.37	0.013
Triglycerides in small LDL (mmol/l)	3543	-0.02	-0.18	0.15	0.854
Concentration of very large HDL particles (mol/l)	3543	0.12	-0.03	0.28	0.114
Total lipids in very large HDL (mmol/l)	3543	0.14	-0.02	0.29	0.082
Phospholipids in very large HDL (mmol/l)	3543	0.11	-0.04	0.26	0.151
Total cholesterol in very large HDL (mmol/l)	3543	0.15	0.00	0.31	0.050
Cholesterol esters in very large HDL (mmol/l)	3543	0.16	0.00	0.31	0.044
Free cholesterol in very large HDL (mmol/l)	3543	0.14	-0.02	0.29	0.080
Triglycerides in very large HDL (mmol/l)	3543	0.08	-0.08	0.23	0.328
Concentration of large HDL particles (mol/l)	3543	0.13	-0.02	0.28	0.095
Total lipids in large HDL (mmol/l)	3543	0.13	-0.02	0.28	0.082
Phospholipids in large HDL (mmol/l)	3543	0.13	-0.02	0.28	0.097
Total cholesterol in large HDL (mmol/l)	3543	0.14	-0.01	0.28	0.074
Cholesterol esters in large HDL (mmol/l)	3543	0.13	-0.02	0.28	0.083
Free cholesterol in large HDL (mmol/l)	3543	0.15	0.00	0.30	0.051
Triglycerides in large HDL (mmol/l)	3543	0.06	-0.10	0.21	0.459
Concentration of medium HDL particles (mol/l)	3543	0.11	-0.04	0.27	0.153
Total lipids in medium HDL (mmol/l)	3543	0.12	-0.03	0.27	0.129
Phospholipids in medium HDL (mmol/l)	3543	0.11	-0.04	0.27	0.146
Total cholesterol in medium HDL (mmol/l)	3543	0.14	-0.02	0.29	0.080
Cholesterol esters in medium HDL (mmol/l)	3543	0.13	-0.02	0.28	0.099
Free cholesterol in medium HDL (mmol/l)	3543	0.17	0.01	0.33	0.035
Triglycerides in medium HDL (mmol/l)	3543	-0.09	-0.24	0.07	0.265
Concentration of small HDL particles (mol/l)	3543	-0.02	-0.17	0.13	0.781
Total lipids in small HDL (mmol/l)	3543	0.03	-0.12	0.18	0.675
Phospholipids in small HDL (mmol/l)	3543	-0.02	-0.17	0.13	0.769
Total cholesterol in small HDL (mmol/l)	3543	0.09	-0.05	0.24	0.217
Cholesterol esters in small HDL (mmol/l)	3543	0.09	-0.06	0.24	0.242
Free cholesterol in small HDL (mmol/l)	3543	0.06	-0.09	0.21	0.398
Triglycerides in small HDL (mmol/l)	3543	-0.10	-0.25	0.05	0.202
Phospholipids to total lipds ratio in chylomicrons and extremely large VLDL (%)	3543	0.03	-0.09	0.14	0.650
Total cholesterol to total lipids ratio in chylomicrons and extremely large VLDL (%)	3543	-0.01	-0.15	0.14	0.946
Cholesterol esters to total lipids ratio in chylomicrons and extremely large VLDL (%)	3543	0.01	-0.14	0.16	0.890
Free cholesterol to total lipids ratio in chylomicrons and extremely large VLDL (%)	3543	-0.04	-0.18	0.10	0.604
Triglycerides to total lipids ratio in chylomicrons and extremely large VLDL (%)	3543	-0.01	-0.06	0.05	0.827
Phospholipids to total lipds ratio in very large VLDL (%)	3543	0.01	-0.14	0.16	0.932
Total cholesterol to total lipids ratio in very large VLDL (%)	3543	0.02	-0.01	0.06	0.205
Cholesterol esters to total lipids ratio in very large VLDL (%)	3543	0.11	-0.03	0.24	0.113
Free cholesterol to total lipids ratio in very large VLDL (%)	3543	0.03	-0.04	0.09	0.396
Triglycerides to total lipids ratio in very large VLDL (%)	3543	-0.11	-0.25	0.03	0.116
Phospholipids to total lipds ratio in large VLDL (%)	3543	-0.04	-0.20	0.11	0.584
Total cholesterol to total lipids ratio in large VLDL (%)	3543	0.05	-0.09	0.20	0.456
Cholesterol esters to total lipids ratio in large VLDL (%)	3543	0.05	-0.04	0.13	0.302
Free cholesterol to total lipids ratio in large VLDL (%)	3543	-0.01	-0.06	0.05	0.795
Triglycerides to total lipids ratio in large VLDL (%)	3543	0.02	-0.11	0.15	0.740
Phospholipids to total lipds ratio in medium VLDL (%)	3543	0.08	-0.07	0.24	0.298
Total cholesterol to total lipids ratio in medium VLDL (%)	3543	0.07	-0.10	0.23	0.422
Cholesterol esters to total lipids ratio in medium VLDL (%)	3543	0.08	-0.09	0.24	0.359
Free cholesterol to total lipids ratio in medium VLDL (%)	3543	-0.02	-0.19	0.15	0.813
Triglycerides to total lipids ratio in medium VLDL (%)	3543	-0.09	-0.25	0.08	0.300
Phospholipids to total lipds ratio in small VLDL (%)	3543	0.06	-0.09	0.21	0.431
Total cholesterol to total lipids ratio in small VLDL (%)	3543	0.11	-0.04	0.26	0.159
Cholesterol esters to total lipids ratio in small VLDL (%)	3543	0.09	-0.06	0.25	0.222
Free cholesterol to total lipids ratio in small VLDL (%)	3543	0.17	0.02	0.32	0.030
Triglycerides to total lipids ratio in small VLDL (%)	3543	-0.12	-0.27	0.03	0.130
Phospholipids to total lipds ratio in very small VLDL (%)	3543	0.10	-0.06	0.26	0.203
Total cholesterol to total lipids ratio in very small VLDL (%)	3543	0.06	-0.10	0.22	0.446
Cholesterol esters to total lipids ratio in very small VLDL (%)	3543	0.01	-0.14	0.17	0.881
Free cholesterol to total lipids ratio in very small VLDL (%)	3543	0.11	-0.05	0.27	0.179
Triglycerides to total lipids ratio in very small VLDL (%)	3543	-0.12	-0.27	0.03	0.131
Phospholipids to total lipds ratio in IDL (%)	3543	-0.08	-0.24	0.08	0.305
Total cholesterol to total lipids ratio in IDL (%)	3543	0.14	-0.02	0.30	0.086

S11 Table Negative control one-sample MR estimates of associations of puberty timing (per year later) with adiposity and cardiometabolic traits at age 8y among males and females in ALSPAC, using a full GRS of 351 SNPs for age at menarche

	Unadj.				
**Standardised outcome at age 8y**	**N**	**Beta (2SLS)**	**LCL**	**UCL**	**P-value**
Cholesterol esters to total lipids ratio in IDL (%)	3543	0.09	-0.07	0.25	0.263
Free cholesterol to total lipids ratio in IDL (%)	3543	0.11	-0.04	0.25	0.146
Triglycerides to total lipids ratio in IDL (%)	3543	-0.14	-0.29	0.02	0.079
Phospholipids to total lipds ratio in large LDL (%)	3543	-0.16	-0.33	0.02	0.076
Total cholesterol to total lipids ratio in large LDL (%)	3543	0.18	0.01	0.34	0.037
Cholesterol esters to total lipids ratio in large LDL (%)	3543	0.16	-0.02	0.34	0.076
Free cholesterol to total lipids ratio in large LDL (%)	3543	0.02	-0.15	0.18	0.844
Triglycerides to total lipids ratio in large LDL (%)	3543	-0.12	-0.28	0.03	0.117
Phospholipids to total lipds ratio in medium LDL (%)	3543	-0.07	-0.22	0.09	0.408
Total cholesterol to total lipids ratio in medium LDL (%)	3543	0.11	-0.04	0.27	0.152
Cholesterol esters to total lipids ratio in medium LDL (%)	3543	0.09	-0.08	0.25	0.304
Free cholesterol to total lipids ratio in medium LDL (%)	3543	-0.02	-0.20	0.15	0.785
Triglycerides to total lipids ratio in medium LDL (%)	3543	-0.10	-0.26	0.05	0.187
Phospholipids to total lipds ratio in small LDL (%)	3543	-0.08	-0.24	0.09	0.367
Total cholesterol to total lipids ratio in small LDL (%)	3543	0.12	-0.04	0.27	0.142
Cholesterol esters to total lipids ratio in small LDL (%)	3543	0.08	-0.08	0.24	0.345
Free cholesterol to total lipids ratio in small LDL (%)	3543	0.01	-0.16	0.18	0.918
Triglycerides to total lipids ratio in small LDL (%)	3543	-0.12	-0.27	0.03	0.111
Phospholipids to total lipds ratio in very large HDL (%)	3543	0.05	-0.09	0.20	0.470
Total cholesterol to total lipids ratio in very large HDL (%)	3543	-0.05	-0.19	0.10	0.518
Cholesterol esters to total lipids ratio in very large HDL (%)	3543	-0.06	-0.21	0.09	0.432
Free cholesterol to total lipids ratio in very large HDL (%)	3543	0.11	-0.04	0.26	0.158
Triglycerides to total lipids ratio in very large HDL (%)	3543	-0.04	-0.19	0.11	0.586
Phospholipids to total lipds ratio in large HDL (%)	3543	-0.13	-0.28	0.03	0.108
Total cholesterol to total lipids ratio in large HDL (%)	3543	0.13	-0.02	0.29	0.083
Cholesterol esters to total lipids ratio in large HDL (%)	3543	0.10	-0.05	0.25	0.201
Free cholesterol to total lipids ratio in large HDL (%)	3543	0.22	0.07	0.38	0.005
Triglycerides to total lipids ratio in large HDL (%)	3543	-0.10	-0.25	0.04	0.158
Phospholipids to total lipds ratio in medium HDL (%)	3543	0.04	-0.11	0.19	0.607
Total cholesterol to total lipids ratio in medium HDL (%)	3543	0.06	-0.10	0.21	0.458
Cholesterol esters to total lipids ratio in medium HDL (%)	3543	0.03	-0.13	0.18	0.742
Free cholesterol to total lipids ratio in medium HDL (%)	3543	0.20	0.04	0.36	0.016
Triglycerides to total lipids ratio in medium HDL (%)	3543	-0.14	-0.29	0.02	0.080
Phospholipids to total lipds ratio in small HDL (%)	3543	-0.09	-0.24	0.06	0.225
Total cholesterol to total lipids ratio in small HDL (%)	3543	0.11	-0.04	0.25	0.151
Cholesterol esters to total lipids ratio in small HDL (%)	3543	0.09	-0.05	0.24	0.214
Free cholesterol to total lipids ratio in small HDL (%)	3543	0.07	-0.08	0.23	0.350
Triglycerides to total lipids ratio in small HDL (%)	3543	-0.11	-0.26	0.04	0.146
Mean diameter for VLDL particles (nm)	3543	-0.08	-0.22	0.07	0.299
Mean diameter for LDL particles (nm)	3543	-0.04	-0.21	0.12	0.614
Mean diameter for HDL particles (nm)	3543	0.14	-0.01	0.30	0.062
Serum total cholesterol (mmol/l)	3543	0.16	0.00	0.32	0.055
Total cholesterol in VLDL (mmol/l)	3543	0.03	-0.13	0.18	0.735
Remnant cholesterol (non-HDL, non-LDL -cholesterol) (mmol/l)	3543	0.09	-0.07	0.25	0.262
Total cholesterol in LDL (mmol/l)	3543	0.12	-0.04	0.29	0.133
Total cholesterol in HDL (mmol/l)	3543	0.16	0.01	0.31	0.039
Total cholesterol in HDL2 (mmol/l)	3543	0.14	0.00	0.29	0.058
Total cholesterol in HDL3 (mmol/l)	3543	0.17	0.02	0.33	0.027
Esterified cholesterol (mmol/l)	3542	0.17	0.01	0.33	0.041
Free cholesterol (mmol/l)	3541	0.14	-0.02	0.30	0.090
Serum total triglycerides (mmol/l)	3543	-0.05	-0.19	0.10	0.536
Triglycerides in VLDL (mmol/l)	3543	-0.05	-0.20	0.09	0.478
Triglycerides in LDL (mmol/l)	3543	0.00	-0.16	0.17	0.977
Triglycerides in HDL (mmol/l)	3543	-0.05	-0.20	0.11	0.555
Diacylglycerol (mmol/l)	3467	-0.05	-0.20	0.11	0.551
Ratio of diacylglycerol to triglycerides	3467	-0.06	-0.22	0.09	0.441
Total phosphoglycerides (mmol/l)	3541	0.18	0.02	0.34	0.024
Ratio of triglycerides to phosphoglycerides	3541	-0.08	-0.22	0.06	0.274
Phosphatidylcholine and other cholines (mmol/l)	3531	0.18	0.02	0.34	0.032
Total cholines (mmol/l)	3542	0.20	0.03	0.36	0.018
Apolipoprotein A-I (g/l)	3543	0.16	0.01	0.32	0.040
Apolipoprotein B (g/l)	3543	0.04	-0.11	0.20	0.591
Ratio of apolipoprotein B to apolipoprotein A-I	3543	-0.03	-0.18	0.12	0.686
Total fatty acids (mmol/l)	3542	0.13	-0.03	0.29	0.114
Estimated description of fatty acid chain length, not actual carbon number	3541	-0.11	-0.27	0.05	0.163
Estimated degree of unsaturation	3541	-0.03	-0.19	0.12	0.665
22:6, docosahexaenoic acid (mmol/l)	3542	0.07	-0.09	0.23	0.370
18:2, linoleic acid (mmol/l)	3542	0.16	0.00	0.33	0.046

S11 Table Negative control one-sample MR estimates of associations of puberty timing (per year later) with adiposity and cardiometabolic traits at age 8y among males and females in ALSPAC, using a full GRS of 351 SNPs for age at menarche

		Unadj.			
**Standardised outcome at age 8y**	**N**	**Beta (2SLS)**	**LCL**	**UCL**	**P-value**
Conjugated linoleic acid (mmol/l)	3540	0.03	-0.12	0.19	0.686
Omega-3 fatty acids (mmol/l)	3542	0.09	-0.07	0.25	0.251
Omega-6 fatty acids (mmol/l)	3542	0.17	0.01	0.33	0.043
Polyunsaturated fatty acids (mmol/l)	3542	0.17	0.00	0.33	0.046
Monounsaturated fatty acids; 16:1, 18:1 (mmol/l)	3541	0.06	-0.09	0.21	0.459
Saturated fatty acids (mmol/l)	3541	0.13	-0.03	0.28	0.120
Ratio of 22:6 docosahexaenoic acid to total fatty acids (%)	3542	0.00	-0.15	0.15	1.000
Ratio of 18:2 linoleic acid to total fatty acids (%)	3542	0.07	-0.08	0.21	0.372
Ratio of conjugated linoleic acid to total fatty acids (%)	3540	0.01	-0.15	0.16	0.923
Ratio of omega-3 fatty acids to total fatty acids (%)	3542	0.00	-0.15	0.15	0.954
Ratio of omega-6 fatty acids to total fatty acids (%)	3542	0.06	-0.09	0.21	0.420
Ratio of polyunsaturated fatty acids to total fatty acids (%)	3542	0.06	-0.09	0.20	0.450
Ratio of monounsaturated fatty acids to total fatty acids (%)	3541	-0.06	-0.21	0.09	0.461
Ratio of saturated fatty acids to total fatty acids (%)	3541	0.00	-0.15	0.15	0.989
Glucose (mmol/l)	3528	-0.15	-0.29	0.00	0.051
Lactate (mmol/l)	3545	0.15	-0.01	0.30	0.061
Pyruvate (mmol/l)	3538	0.04	-0.12	0.19	0.623
Citrate (mmol/l)	3538	0.20	0.03	0.36	0.019
Alanine (mmol/l)	3544	0.07	-0.08	0.22	0.342
Glutamine (mmol/l)	3538	0.16	0.01	0.32	0.039
Histidine (mmol/l)	3540	0.08	-0.06	0.22	0.257
Isoleucine (mmol/l)	3544	0.04	-0.11	0.19	0.575
Leucine (mmol/l)	3544	-0.01	-0.16	0.13	0.851
Valine (mmol/l)	3545	0.04	-0.11	0.19	0.568
Phenylalanine (mmol/l)	3536	-0.01	-0.16	0.14	0.877
Tyrosine (mmol/l)	3534	0.03	-0.12	0.17	0.733
Acetate (mmol/l)	3545	-0.10	-0.25	0.05	0.201
Acetoacetate (mmol/l)	3540	0.07	-0.07	0.20	0.345
3-hydroxybutyrate (mmol/l)	3542	0.10	-0.05	0.24	0.184
Creatinine (mmol/l)	3538	-0.04	-0.19	0.11	0.579
Albumin (signal area)	3538	0.05	-0.11	0.22	0.533
Glycoprotein acetyls, mainly a1-acid glycoprotein (mmol/l)	3544	-0.16	-0.31	0.00	0.045

Complete case sample

		Unadj.			
**Standardised outcome at age 8y**	**N**	**Beta (2SLS)**	**LCL**	**UCL**	**P-value**
Body mass index (kg/m^2)	1193	-0.28	-0.48	-0.08	0.006
Fat mass index (kg/m^2)	1193	-0.25	-0.45	-0.05	0.012
Lean mass index (kg/m^2)	1193	-0.42	-0.65	-0.18	4.55E-04
Systolic blood pressure (mmHg)	1193	-0.18	-0.39	0.03	0.088
Diastolic blood pressure (mmHg)	1193	-0.12	-0.33	0.08	0.238
Concentration of chylomicrons and extremely large VLDL particles (mol/l)	1193	-0.02	-0.21	0.18	0.852
Total lipids in chylomicrons and extremely large VLDL (mmol/l)	1193	-0.02	-0.21	0.18	0.857
Phospholipids in chylomicrons and extremely large VLDL (mmol/l)	1193	-0.01	-0.21	0.18	0.887
Total cholesterol in chylomicrons and extremely large VLDL (mmol/l)	1193	-0.02	-0.21	0.18	0.854
Cholesterol esters in chylomicrons and extremely large VLDL (mmol/l)	1193	-0.02	-0.22	0.17	0.827
Free cholesterol in chylomicrons and extremely large VLDL (mmol/l)	1193	-0.01	-0.21	0.18	0.889
Triglycerides in chylomicrons and extremely large VLDL (mmol/l)	1193	-0.02	-0.21	0.18	0.854
Concentration of very large VLDL particles (mol/l)	1193	-0.02	-0.22	0.17	0.816
Total lipids in very large VLDL (mmol/l)	1193	-0.02	-0.22	0.17	0.815
Phospholipids in very large VLDL (mmol/l)	1193	-0.02	-0.21	0.18	0.873
Total cholesterol in very large VLDL (mmol/l)	1193	-0.02	-0.21	0.18	0.853
Cholesterol esters in very large VLDL (mmol/l)	1193	-0.02	-0.22	0.17	0.818
Free cholesterol in very large VLDL (mmol/l)	1193	-0.01	-0.21	0.18	0.890
Triglycerides in very large VLDL (mmol/l)	1193	-0.03	-0.22	0.17	0.790
Concentration of large VLDL particles (mol/l)	1193	-0.03	-0.22	0.16	0.735
Total lipids in large VLDL (mmol/l)	1193	-0.03	-0.22	0.16	0.749
Phospholipids in large VLDL (mmol/l)	1193	-0.03	-0.22	0.16	0.782
Total cholesterol in large VLDL (mmol/l)	1193	-0.02	-0.21	0.17	0.833
Cholesterol esters in large VLDL (mmol/l)	1193	-0.02	-0.21	0.18	0.842
Free cholesterol in large VLDL (mmol/l)	1193	-0.02	-0.21	0.17	0.828
Triglycerides in large VLDL (mmol/l)	1193	-0.04	-0.23	0.15	0.709
Concentration of medium VLDL particles (mol/l)	1193	-0.04	-0.23	0.15	0.697
Total lipids in medium VLDL (mmol/l)	1193	-0.03	-0.23	0.16	0.731
Phospholipids in medium VLDL (mmol/l)	1193	-0.03	-0.22	0.17	0.788
Total cholesterol in medium VLDL (mmol/l)	1193	0.00	-0.20	0.20	0.979

S11 Table Negative control one-sample MR estimates of associations of puberty timing (per year later) with adiposity and cardiometabolic traits at age 8y among males and females in ALSPAC, using a full GRS of 351 SNPs for age at menarche

	Unadj.				
**Standardised outcome at age 8y**	**N**	**Beta (2SLS)**	**LCL**	**UCL**	**P-value**
Cholesterol esters in medium VLDL (mmol/l)	1193	0.02	-0.19	0.23	0.870
Free cholesterol in medium VLDL (mmol/l)	1193	-0.03	-0.22	0.17	0.800
Triglycerides in medium VLDL (mmol/l)	1193	-0.05	-0.24	0.14	0.608
Concentration of small VLDL particles (mol/l)	1193	-0.02	-0.22	0.19	0.864
Total lipids in small VLDL (mmol/l)	1193	-0.01	-0.22	0.20	0.938
Phospholipids in small VLDL (mmol/l)	1193	0.01	-0.20	0.22	0.949
Total cholesterol in small VLDL (mmol/l)	1193	0.03	-0.19	0.25	0.805
Cholesterol esters in small VLDL (mmol/l)	1193	0.03	-0.19	0.26	0.777
Free cholesterol in small VLDL (mmol/l)	1193	0.02	-0.19	0.23	0.865
Triglycerides in small VLDL (mmol/l)	1193	-0.04	-0.24	0.16	0.697
Concentration of very small VLDL particles (mol/l)	1193	0.08	-0.14	0.30	0.470
Total lipids in very small VLDL (mmol/l)	1193	0.07	-0.15	0.30	0.516
Phospholipids in very small VLDL (mmol/l)	1193	0.10	-0.12	0.32	0.367
Total cholesterol in very small VLDL (mmol/l)	1193	0.06	-0.17	0.29	0.604
Cholesterol esters in very small VLDL (mmol/l)	1193	0.05	-0.17	0.28	0.651
Free cholesterol in very small VLDL (mmol/l)	1193	0.07	-0.16	0.30	0.551
Triglycerides in very small VLDL (mmol/l)	1193	0.02	-0.19	0.22	0.880
Concentration of IDL particles (mol/l)	1193	0.12	-0.10	0.34	0.295
Total lipids in IDL (mmol/l)	1193	0.13	-0.09	0.35	0.245
Phospholipids in IDL (mmol/l)	1193	0.13	-0.09	0.35	0.243
Total cholesterol in IDL (mmol/l)	1193	0.13	-0.09	0.35	0.251
Cholesterol esters in IDL (mmol/l)	1193	0.12	-0.10	0.34	0.281
Free cholesterol in IDL (mmol/l)	1193	0.14	-0.08	0.35	0.216
Triglycerides in IDL (mmol/l)	1193	0.06	-0.15	0.27	0.557
Concentration of large LDL particles (mol/l)	1193	0.12	-0.10	0.34	0.291
Total lipids in large LDL (mmol/l)	1193	0.13	-0.09	0.35	0.235
Phospholipids in large LDL (mmol/l)	1193	0.13	-0.09	0.35	0.237
Total cholesterol in large LDL (mmol/l)	1193	0.13	-0.08	0.35	0.228
Cholesterol esters in large LDL (mmol/l)	1193	0.13	-0.09	0.35	0.237
Free cholesterol in large LDL (mmol/l)	1193	0.14	-0.08	0.35	0.208
Triglycerides in large LDL (mmol/l)	1193	0.07	-0.14	0.29	0.509
Concentration of medium LDL particles (mol/l)	1193	0.11	-0.11	0.33	0.343
Total lipids in medium LDL (mmol/l)	1193	0.12	-0.10	0.34	0.275
Phospholipids in medium LDL (mmol/l)	1193	0.14	-0.08	0.36	0.225
Total cholesterol in medium LDL (mmol/l)	1193	0.12	-0.10	0.34	0.274
Cholesterol esters in medium LDL (mmol/l)	1193	0.12	-0.10	0.34	0.301
Free cholesterol in medium LDL (mmol/l)	1193	0.15	-0.07	0.36	0.189
Triglycerides in medium LDL (mmol/l)	1193	0.05	-0.17	0.27	0.639
Concentration of small LDL particles (mol/l)	1193	0.11	-0.11	0.34	0.312
Total lipids in small LDL (mmol/l)	1193	0.12	-0.10	0.34	0.278
Phospholipids in small LDL (mmol/l)	1193	0.13	-0.09	0.35	0.235
Total cholesterol in small LDL (mmol/l)	1193	0.12	-0.10	0.34	0.274
Cholesterol esters in small LDL (mmol/l)	1193	0.11	-0.11	0.33	0.316
Free cholesterol in small LDL (mmol/l)	1193	0.14	-0.08	0.36	0.206
Triglycerides in small LDL (mmol/l)	1193	0.04	-0.17	0.26	0.697
Concentration of very large HDL particles (mol/l)	1193	0.11	-0.11	0.32	0.334
Total lipids in very large HDL (mmol/l)	1193	0.10	-0.11	0.31	0.360
Phospholipids in very large HDL (mmol/l)	1193	0.10	-0.11	0.32	0.338
Total cholesterol in very large HDL (mmol/l)	1193	0.09	-0.13	0.30	0.420
Cholesterol esters in very large HDL (mmol/l)	1193	0.08	-0.13	0.30	0.453
Free cholesterol in very large HDL (mmol/l)	1193	0.10	-0.12	0.32	0.364
Triglycerides in very large HDL (mmol/l)	1193	0.06	-0.15	0.27	0.585
Concentration of large HDL particles (mol/l)	1193	0.14	-0.07	0.35	0.205
Total lipids in large HDL (mmol/l)	1193	0.13	-0.08	0.34	0.228
Phospholipids in large HDL (mmol/l)	1193	0.14	-0.07	0.35	0.187
Total cholesterol in large HDL (mmol/l)	1193	0.12	-0.10	0.33	0.278
Cholesterol esters in large HDL (mmol/l)	1193	0.12	-0.10	0.33	0.283
Free cholesterol in large HDL (mmol/l)	1193	0.12	-0.09	0.34	0.252
Triglycerides in large HDL (mmol/l)	1193	0.13	-0.09	0.34	0.253
Concentration of medium HDL particles (mol/l)	1193	0.16	-0.05	0.37	0.127
Total lipids in medium HDL (mmol/l)	1193	0.16	-0.05	0.37	0.135
Phospholipids in medium HDL (mmol/l)	1193	0.15	-0.06	0.36	0.152
Total cholesterol in medium HDL (mmol/l)	1193	0.15	-0.06	0.37	0.156
Cholesterol esters in medium HDL (mmol/l)	1193	0.15	-0.06	0.36	0.171
Free cholesterol in medium HDL (mmol/l)	1193	0.17	-0.04	0.39	0.112
Triglycerides in medium HDL (mmol/l)	1193	0.02	-0.19	0.22	0.865
Concentration of small HDL particles (mol/l)	1193	0.08	-0.12	0.28	0.453
Total lipids in small HDL (mmol/l)	1193	0.10	-0.10	0.31	0.313

S11 Table Negative control one-sample MR estimates of associations of puberty timing (per year later) with adiposity and cardiometabolic traits at age 8y among males and females in ALSPAC, using a full GRS of 351 SNPs for age at menarche

	Unadj.				
**Standardised outcome at age 8y**	**N**	**Beta (2SLS)**	**LCL**	**UCL**	**P-value**
Phospholipids in small HDL (mmol/l)	1193	0.08	-0.13	0.28	0.466
Total cholesterol in small HDL (mmol/l)	1193	0.09	-0.12	0.30	0.396
Cholesterol esters in small HDL (mmol/l)	1193	0.07	-0.14	0.29	0.488
Free cholesterol in small HDL (mmol/l)	1193	0.11	-0.09	0.31	0.293
Triglycerides in small HDL (mmol/l)	1193	-0.01	-0.21	0.19	0.886
Phospholipids to total lipds ratio in chylomicrons and extremely large VLDL (%)	1193	0.04	-0.11	0.19	0.596
Total cholesterol to total lipids ratio in chylomicrons and extremely large VLDL (%)	1193	0.07	-0.15	0.30	0.533
Cholesterol esters to total lipids ratio in chylomicrons and extremely large VLDL (%)	1193	0.06	-0.17	0.29	0.591
Free cholesterol to total lipids ratio in chylomicrons and extremely large VLDL (%)	1193	0.05	-0.15	0.25	0.642
Triglycerides to total lipids ratio in chylomicrons and extremely large VLDL (%)	1193	-0.03	-0.11	0.05	0.533
Phospholipids to total lipds ratio in very large VLDL (%)	1193	0.09	-0.11	0.30	0.375
Total cholesterol to total lipids ratio in very large VLDL (%)	1193	0.02	-0.02	0.07	0.291
Cholesterol esters to total lipids ratio in very large VLDL (%)	1193	0.08	-0.08	0.23	0.333
Free cholesterol to total lipids ratio in very large VLDL (%)	1193	0.05	-0.04	0.15	0.294
Triglycerides to total lipids ratio in very large VLDL (%)	1193	-0.14	-0.34	0.06	0.169
Phospholipids to total lipds ratio in large VLDL (%)	1193	0.04	-0.17	0.26	0.685
Total cholesterol to total lipids ratio in large VLDL (%)	1193	0.12	-0.08	0.32	0.235
Cholesterol esters to total lipids ratio in large VLDL (%)	1193	0.07	-0.04	0.18	0.221
Free cholesterol to total lipids ratio in large VLDL (%)	1193	0.01	-0.06	0.09	0.713
Triglycerides to total lipids ratio in large VLDL (%)	1193	-0.07	-0.22	0.09	0.404
Phospholipids to total lipds ratio in medium VLDL (%)	1193	0.15	-0.05	0.36	0.141
Total cholesterol to total lipids ratio in medium VLDL (%)	1193	0.11	-0.12	0.33	0.344
Cholesterol esters to total lipids ratio in medium VLDL (%)	1193	0.09	-0.14	0.32	0.461
Free cholesterol to total lipids ratio in medium VLDL (%)	1193	0.09	-0.12	0.29	0.404
Triglycerides to total lipids ratio in medium VLDL (%)	1193	-0.14	-0.36	0.08	0.213
Phospholipids to total lipds ratio in small VLDL (%)	1193	0.05	-0.17	0.27	0.664
Total cholesterol to total lipids ratio in small VLDL (%)	1193	0.08	-0.13	0.28	0.475
Cholesterol esters to total lipids ratio in small VLDL (%)	1193	0.06	-0.15	0.27	0.576
Free cholesterol to total lipids ratio in small VLDL (%)	1193	0.17	-0.05	0.38	0.132
Triglycerides to total lipids ratio in small VLDL (%)	1193	-0.08	-0.29	0.13	0.435
Phospholipids to total lipds ratio in very small VLDL (%)	1193	0.13	-0.09	0.34	0.250
Total cholesterol to total lipids ratio in very small VLDL (%)	1193	-0.01	-0.23	0.21	0.948
Cholesterol esters to total lipids ratio in very small VLDL (%)	1193	-0.03	-0.24	0.18	0.781
Free cholesterol to total lipids ratio in very small VLDL (%)	1193	0.03	-0.20	0.25	0.819
Triglycerides to total lipids ratio in very small VLDL (%)	1193	-0.05	-0.26	0.15	0.612
Phospholipids to total lipds ratio in IDL (%)	1193	-0.01	-0.24	0.22	0.932
Total cholesterol to total lipids ratio in IDL (%)	1193	0.06	-0.16	0.28	0.611
Cholesterol esters to total lipids ratio in IDL (%)	1193	0.02	-0.20	0.24	0.863
Free cholesterol to total lipids ratio in IDL (%)	1193	0.09	-0.10	0.27	0.373
Triglycerides to total lipids ratio in IDL (%)	1193	-0.07	-0.28	0.14	0.501
Phospholipids to total lipds ratio in large LDL (%)	1193	-0.12	-0.33	0.09	0.259
Total cholesterol to total lipids ratio in large LDL (%)	1193	0.11	-0.10	0.31	0.317
Cholesterol esters to total lipids ratio in large LDL (%)	1193	0.11	-0.10	0.33	0.293
Free cholesterol to total lipids ratio in large LDL (%)	1193	-0.04	-0.24	0.16	0.702
Triglycerides to total lipids ratio in large LDL (%)	1193	-0.05	-0.26	0.16	0.631
Phospholipids to total lipds ratio in medium LDL (%)	1193	-0.07	-0.28	0.14	0.528
Total cholesterol to total lipids ratio in medium LDL (%)	1193	0.08	-0.13	0.28	0.452
Cholesterol esters to total lipids ratio in medium LDL (%)	1193	0.08	-0.13	0.29	0.447
Free cholesterol to total lipids ratio in medium LDL (%)	1193	-0.07	-0.29	0.15	0.540
Triglycerides to total lipids ratio in medium LDL (%)	1193	-0.03	-0.25	0.18	0.766
Phospholipids to total lipds ratio in small LDL (%)	1193	-0.08	-0.29	0.14	0.476
Total cholesterol to total lipids ratio in small LDL (%)	1193	0.08	-0.12	0.28	0.447
Cholesterol esters to total lipids ratio in small LDL (%)	1193	0.08	-0.13	0.29	0.463
Free cholesterol to total lipids ratio in small LDL (%)	1193	-0.06	-0.28	0.17	0.616
Triglycerides to total lipids ratio in small LDL (%)	1193	-0.03	-0.23	0.18	0.783
Phospholipids to total lipds ratio in very large HDL (%)	1193	0.05	-0.16	0.25	0.664
Total cholesterol to total lipids ratio in very large HDL (%)	1193	-0.05	-0.26	0.16	0.633
Cholesterol esters to total lipids ratio in very large HDL (%)	1193	-0.05	-0.26	0.16	0.613
Free cholesterol to total lipids ratio in very large HDL (%)	1193	0.04	-0.17	0.25	0.721
Triglycerides to total lipids ratio in very large HDL (%)	1193	0.01	-0.20	0.22	0.926
Phospholipids to total lipds ratio in large HDL (%)	1193	0.03	-0.19	0.24	0.812
Total cholesterol to total lipids ratio in large HDL (%)	1193	-0.01	-0.22	0.20	0.936
Cholesterol esters to total lipids ratio in large HDL (%)	1193	-0.02	-0.23	0.19	0.862
Free cholesterol to total lipids ratio in large HDL (%)	1193	0.03	-0.17	0.24	0.741
Triglycerides to total lipids ratio in large HDL (%)	1193	-0.02	-0.23	0.19	0.831
Phospholipids to total lipds ratio in medium HDL (%)	1193	0.06	-0.15	0.28	0.574
Total cholesterol to total lipids ratio in medium HDL (%)	1193	-0.02	-0.23	0.19	0.830
Cholesterol esters to total lipids ratio in medium HDL (%)	1193	-0.04	-0.25	0.17	0.732

S11 Table Negative control one-sample MR estimates of associations of puberty timing (per year later) with adiposity and cardiometabolic traits at age 8y among males and females in ALSPAC, using a full GRS of 351 SNPs for age at menarche

	Unadj.				
**Standardised outcome at age 8y**	**N**	**Beta (2SLS)**	**LCL**	**UCL**	**P-value**
Free cholesterol to total lipids ratio in medium HDL (%)	1193	0.07	-0.14	0.28	0.527
Triglycerides to total lipids ratio in medium HDL (%)	1193	-0.04	-0.26	0.17	0.678
Phospholipids to total lipds ratio in small HDL (%)	1193	-0.04	-0.26	0.17	0.682
Total cholesterol to total lipids ratio in small HDL (%)	1193	0.05	-0.16	0.26	0.635
Cholesterol esters to total lipids ratio in small HDL (%)	1193	0.04	-0.17	0.26	0.677
Free cholesterol to total lipids ratio in small HDL (%)	1193	0.03	-0.19	0.25	0.791
Triglycerides to total lipids ratio in small HDL (%)	1193	-0.05	-0.26	0.15	0.617
Mean diameter for VLDL particles (nm)	1193	-0.05	-0.25	0.15	0.631
Mean diameter for LDL particles (nm)	1193	-0.06	-0.27	0.15	0.568
Mean diameter for HDL particles (nm)	1193	0.11	-0.10	0.33	0.308
Serum total cholesterol (mmol/l)	1193	0.15	-0.07	0.37	0.189
Total cholesterol in VLDL (mmol/l)	1193	0.02	-0.20	0.23	0.874
Remnant cholesterol (non-HDL, non-LDL -cholesterol) (mmol/l)	1193	0.07	-0.15	0.30	0.512
Total cholesterol in LDL (mmol/l)	1193	0.13	-0.09	0.35	0.249
Total cholesterol in HDL (mmol/l)	1193	0.13	-0.08	0.34	0.224
Total cholesterol in HDL2 (mmol/l)	1193	0.13	-0.09	0.34	0.244
Total cholesterol in HDL3 (mmol/l)	1193	0.13	-0.08	0.35	0.218
Esterified cholesterol (mmol/l)	1193	0.15	-0.07	0.37	0.186
Free cholesterol (mmol/l)	1193	0.14	-0.08	0.35	0.223
Serum total triglycerides (mmol/l)	1193	-0.02	-0.22	0.18	0.848
Triglycerides in VLDL (mmol/l)	1193	-0.04	-0.23	0.16	0.705
Triglycerides in LDL (mmol/l)	1193	0.06	-0.16	0.28	0.580
Triglycerides in HDL (mmol/l)	1193	0.04	-0.17	0.24	0.723
Diacylglycerol (mmol/l)	1193	0.02	-0.19	0.23	0.856
Ratio of diacylglycerol to triglycerides	1193	0.02	-0.20	0.24	0.863
Total phosphoglycerides (mmol/l)	1193	0.17	-0.05	0.39	0.123
Ratio of triglycerides to phosphoglycerides	1193	-0.06	-0.26	0.13	0.515
Phosphatidylcholine and other cholines (mmol/l)	1193	0.15	-0.07	0.37	0.192
Total cholines (mmol/l)	1193	0.16	-0.06	0.38	0.150
Apolipoprotein A-I (g/l)	1193	0.15	-0.06	0.36	0.170
Apolipoprotein B (g/l)	1193	0.05	-0.17	0.27	0.641
Ratio of apolipoprotein B to apolipoprotein A-I	1193	-0.01	-0.23	0.20	0.909
Total fatty acids (mmol/l)	1193	0.10	-0.11	0.32	0.358
Estimated description of fatty acid chain length, not actual carbon number	1193	-0.18	-0.39	0.04	0.111
Estimated degree of unsaturation	1193	-0.07	-0.28	0.15	0.544
22:6, docosahexaenoic acid (mmol/l)	1193	0.00	-0.20	0.21	0.986
18:2, linoleic acid (mmol/l)	1193	0.10	-0.12	0.32	0.373
Conjugated linoleic acid (mmol/l)	1193	-0.03	-0.27	0.21	0.802
Omega-3 fatty acids (mmol/l)	1193	0.11	-0.10	0.32	0.309
Omega-6 fatty acids (mmol/l)	1193	0.12	-0.10	0.35	0.267
Polyunsaturated fatty acids (mmol/l)	1193	0.13	-0.09	0.35	0.253
Monounsaturated fatty acids; 16:1, 18:1 (mmol/l)	1193	0.03	-0.17	0.23	0.769
Saturated fatty acids (mmol/l)	1193	0.11	-0.11	0.33	0.311
Ratio of 22:6 docosahexaenoic acid to total fatty acids (%)	1193	-0.06	-0.28	0.15	0.549
Ratio of 18:2 linoleic acid to total fatty acids (%)	1193	0.03	-0.18	0.23	0.792
Ratio of conjugated linoleic acid to total fatty acids (%)	1193	-0.07	-0.31	0.18	0.586
Ratio of omega-3 fatty acids to total fatty acids (%)	1193	0.04	-0.17	0.24	0.730
Ratio of omega-6 fatty acids to total fatty acids (%)	1193	0.04	-0.16	0.25	0.673
Ratio of polyunsaturated fatty acids to total fatty acids (%)	1193	0.05	-0.15	0.25	0.635
Ratio of monounsaturated fatty acids to total fatty acids (%)	1193	-0.09	-0.28	0.10	0.358
Ratio of saturated fatty acids to total fatty acids (%)	1193	0.06	-0.16	0.28	0.577
Glucose (mmol/l)	1193	-0.20	-0.41	0.01	0.065
Lactate (mmol/l)	1193	0.10	-0.11	0.31	0.346
Pyruvate (mmol/l)	1193	0.15	-0.08	0.37	0.203
Citrate (mmol/l)	1193	0.02	-0.19	0.23	0.868
Alanine (mmol/l)	1193	0.05	-0.17	0.27	0.665
Glutamine (mmol/l)	1193	0.06	-0.15	0.28	0.554
Histidine (mmol/l)	1193	0.00	-0.17	0.17	0.997
Isoleucine (mmol/l)	1193	0.10	-0.11	0.31	0.359
Leucine (mmol/l)	1193	0.03	-0.18	0.24	0.791
Valine (mmol/l)	1193	0.09	-0.12	0.30	0.410
Phenylalanine (mmol/l)	1193	0.08	-0.14	0.30	0.473
Tyrosine (mmol/l)	1193	0.06	-0.15	0.27	0.586
Acetate (mmol/l)	1193	-0.01	-0.24	0.22	0.943
Acetoacetate (mmol/l)	1193	-0.04	-0.23	0.15	0.700
3-hydroxybutyrate (mmol/l)	1193	-0.02	-0.22	0.19	0.874
Creatinine (mmol/l)	1193	-0.14	-0.34	0.05	0.150
Albumin (signal area)	1193	0.09	-0.12	0.30	0.410

S11 Table Negative control one-sample MR estimates of associations of puberty timing (per year later) with adiposity and cardiometabolic traits at age 8y among males and females in ALSPAC, using a full GRS of 351 SNPs for age at menarche

	Unadj.				
**Standardised outcome at age 8y**	**N**	**Beta (2SLS)**	**LCL**	**UCL**	**P-value**
Glycoprotein acetyls, mainly a1-acid glycoprotein (mmol/l)	1193	0.05	-0.16	0.25	0.664
