# Supplementary material for: Influence of puberty timing on adiposity and cardiometabolic traits: A Mendelian randomisation study
Source: PLoS Med. 2018 Aug 28;15(8):e1002641. doi: 10.1371/journal.pmed.1002641 (PMC6112630; doi:10.1371/journal.pmed.1002641)
Supplement: S12 Table — (PDF) [file pmed.1002641.s031.pdf]

S12 Table Negative control one-sample MR estimates of associations of age at voice breaking (per year later) with adiposity and cardiometabolic traits at age 8y among males in ALSPAC, using a refined GRS of 115 SNPs for age at menarche/voice breaking

	Unadj.				
**Standardised outcome at age 8y**	**N**	**Beta (2SLS)**	**LCL**	**UCL**	**P-value**
Body mass index (kg/m^2)	2150	-0.36	-0.72	0.00	0.048
Fat mass index (kg/m^2)	2024	-0.26	-0.56	0.05	0.100
Lean mass index (kg/m^2)	2024	-0.11	-0.38	0.16	0.418
Systolic blood pressure (mmHg)	2118	-0.17	-0.53	0.18	0.335
Diastolic blood pressure (mmHg)	2116	-0.27	-0.65	0.11	0.164
Concentration of chylomicrons and extremely large VLDL particles (mol/l)	1599	-0.10	-0.46	0.26	0.595
Total lipids in chylomicrons and extremely large VLDL (mmol/l)	1599	-0.10	-0.46	0.27	0.597
Phospholipids in chylomicrons and extremely large VLDL (mmol/l)	1599	-0.11	-0.47	0.26	0.562
Total cholesterol in chylomicrons and extremely large VLDL (mmol/l)	1599	-0.10	-0.46	0.26	0.595
Cholesterol esters in chylomicrons and extremely large VLDL (mmol/l)	1599	-0.08	-0.44	0.28	0.670
Free cholesterol in chylomicrons and extremely large VLDL (mmol/l)	1599	-0.11	-0.48	0.25	0.544
Triglycerides in chylomicrons and extremely large VLDL (mmol/l)	1599	-0.10	-0.46	0.27	0.604
Concentration of very large VLDL particles (mol/l)	1599	-0.10	-0.47	0.26	0.573
Total lipids in very large VLDL (mmol/l)	1599	-0.11	-0.47	0.26	0.569
Phospholipids in very large VLDL (mmol/l)	1599	-0.09	-0.45	0.27	0.616
Total cholesterol in very large VLDL (mmol/l)	1599	-0.10	-0.46	0.26	0.594
Cholesterol esters in very large VLDL (mmol/l)	1599	-0.10	-0.46	0.26	0.594
Free cholesterol in very large VLDL (mmol/l)	1599	-0.10	-0.46	0.26	0.602
Triglycerides in very large VLDL (mmol/l)	1599	-0.11	-0.47	0.25	0.552
Concentration of large VLDL particles (mol/l)	1599	-0.13	-0.49	0.24	0.495
Total lipids in large VLDL (mmol/l)	1599	-0.12	-0.49	0.24	0.510
Phospholipids in large VLDL (mmol/l)	1599	-0.12	-0.48	0.25	0.524
Total cholesterol in large VLDL (mmol/l)	1599	-0.11	-0.47	0.25	0.557
Cholesterol esters in large VLDL (mmol/l)	1599	-0.11	-0.47	0.25	0.547
Free cholesterol in large VLDL (mmol/l)	1599	-0.10	-0.47	0.26	0.572
Triglycerides in large VLDL (mmol/l)	1599	-0.13	-0.49	0.24	0.491
Concentration of medium VLDL particles (mol/l)	1599	-0.14	-0.51	0.22	0.443
Total lipids in medium VLDL (mmol/l)	1599	-0.14	-0.50	0.23	0.455
Phospholipids in medium VLDL (mmol/l)	1599	-0.14	-0.50	0.23	0.465
Total cholesterol in medium VLDL (mmol/l)	1599	-0.10	-0.46	0.27	0.608
Cholesterol esters in medium VLDL (mmol/l)	1599	-0.05	-0.41	0.31	0.805
Free cholesterol in medium VLDL (mmol/l)	1599	-0.14	-0.51	0.23	0.448
Triglycerides in medium VLDL (mmol/l)	1599	-0.16	-0.52	0.21	0.399
Concentration of small VLDL particles (mol/l)	1599	-0.16	-0.54	0.22	0.410
Total lipids in small VLDL (mmol/l)	1599	-0.14	-0.52	0.24	0.473
Phospholipids in small VLDL (mmol/l)	1599	-0.13	-0.52	0.25	0.492
Total cholesterol in small VLDL (mmol/l)	1599	-0.05	-0.41	0.32	0.810
Cholesterol esters in small VLDL (mmol/l)	1599	-0.01	-0.37	0.36	0.972
Free cholesterol in small VLDL (mmol/l)	1599	-0.10	-0.48	0.27	0.591
Triglycerides in small VLDL (mmol/l)	1599	-0.19	-0.57	0.19	0.331
Concentration of very small VLDL particles (mol/l)	1599	0.08	-0.28	0.43	0.680
Total lipids in very small VLDL (mmol/l)	1599	0.11	-0.25	0.47	0.556
Phospholipids in very small VLDL (mmol/l)	1599	0.11	-0.24	0.47	0.531
Total cholesterol in very small VLDL (mmol/l)	1599	0.18	-0.18	0.54	0.328
Cholesterol esters in very small VLDL (mmol/l)	1599	0.15	-0.20	0.51	0.396
Free cholesterol in very small VLDL (mmol/l)	1599	0.20	-0.16	0.57	0.274
Triglycerides in very small VLDL (mmol/l)	1599	-0.15	-0.53	0.24	0.449
Concentration of IDL particles (mol/l)	1599	0.09	-0.26	0.45	0.602
Total lipids in IDL (mmol/l)	1599	0.16	-0.20	0.51	0.389
Phospholipids in IDL (mmol/l)	1599	0.11	-0.25	0.47	0.547
Total cholesterol in IDL (mmol/l)	1599	0.20	-0.16	0.56	0.286
Cholesterol esters in IDL (mmol/l)	1599	0.20	-0.16	0.57	0.268
Free cholesterol in IDL (mmol/l)	1599	0.17	-0.19	0.52	0.362
Triglycerides in IDL (mmol/l)	1599	-0.05	-0.42	0.31	0.768
Concentration of large LDL particles (mol/l)	1599	0.06	-0.29	0.41	0.739
Total lipids in large LDL (mmol/l)	1599	0.13	-0.23	0.48	0.480
Phospholipids in large LDL (mmol/l)	1599	0.13	-0.23	0.48	0.488
Total cholesterol in large LDL (mmol/l)	1599	0.15	-0.21	0.51	0.411
Cholesterol esters in large LDL (mmol/l)	1599	0.14	-0.22	0.50	0.439
Free cholesterol in large LDL (mmol/l)	1599	0.17	-0.19	0.53	0.349
Triglycerides in large LDL (mmol/l)	1599	-0.06	-0.43	0.30	0.744
Concentration of medium LDL particles (mol/l)	1599	0.01	-0.34	0.37	0.939
Total lipids in medium LDL (mmol/l)	1599	0.09	-0.26	0.44	0.616
Phospholipids in medium LDL (mmol/l)	1599	0.15	-0.20	0.51	0.400
Total cholesterol in medium LDL (mmol/l)	1599	0.10	-0.25	0.45	0.580
Cholesterol esters in medium LDL (mmol/l)	1599	0.07	-0.28	0.43	0.690
Free cholesterol in medium LDL (mmol/l)	1599	0.22	-0.14	0.59	0.232

S12 Table Negative control one-sample MR estimates of associations of age at voice breaking (per year later) with adiposity and cardiometabolic traits at age 8y among males in ALSPAC, using a refined GRS of 115 SNPs for age at menarche/voice breaking

	Unadj.				
**Standardised outcome at age 8y**	**N**	**Beta (2SLS)**	**LCL**	**UCL**	**P-value**
Triglycerides in medium LDL (mmol/l)	1599	-0.11	-0.48	0.26	0.553
Concentration of small LDL particles (mol/l)	1599	0.03	-0.32	0.38	0.862
Total lipids in small LDL (mmol/l)	1599	0.09	-0.27	0.44	0.629
Phospholipids in small LDL (mmol/l)	1599	0.14	-0.22	0.50	0.441
Total cholesterol in small LDL (mmol/l)	1599	0.10	-0.25	0.45	0.584
Cholesterol esters in small LDL (mmol/l)	1599	0.05	-0.30	0.41	0.761
Free cholesterol in small LDL (mmol/l)	1599	0.28	-0.09	0.65	0.142
Triglycerides in small LDL (mmol/l)	1599	-0.13	-0.51	0.24	0.482
Concentration of very large HDL particles (mol/l)	1599	0.17	-0.20	0.54	0.370
Total lipids in very large HDL (mmol/l)	1599	0.19	-0.19	0.56	0.327
Phospholipids in very large HDL (mmol/l)	1599	0.16	-0.20	0.53	0.382
Total cholesterol in very large HDL (mmol/l)	1599	0.20	-0.18	0.57	0.305
Cholesterol esters in very large HDL (mmol/l)	1599	0.20	-0.18	0.58	0.298
Free cholesterol in very large HDL (mmol/l)	1599	0.18	-0.19	0.55	0.344
Triglycerides in very large HDL (mmol/l)	1599	0.12	-0.24	0.49	0.507
Concentration of large HDL particles (mol/l)	1599	0.17	-0.19	0.54	0.354
Total lipids in large HDL (mmol/l)	1599	0.19	-0.18	0.57	0.308
Phospholipids in large HDL (mmol/l)	1599	0.17	-0.20	0.54	0.365
Total cholesterol in large HDL (mmol/l)	1599	0.21	-0.17	0.59	0.270
Cholesterol esters in large HDL (mmol/l)	1599	0.20	-0.17	0.58	0.288
Free cholesterol in large HDL (mmol/l)	1599	0.25	-0.14	0.63	0.212
Triglycerides in large HDL (mmol/l)	1599	0.02	-0.34	0.37	0.929
Concentration of medium HDL particles (mol/l)	1599	0.02	-0.34	0.39	0.912
Total lipids in medium HDL (mmol/l)	1599	0.04	-0.33	0.41	0.833
Phospholipids in medium HDL (mmol/l)	1599	0.04	-0.32	0.40	0.829
Total cholesterol in medium HDL (mmol/l)	1599	0.10	-0.28	0.47	0.622
Cholesterol esters in medium HDL (mmol/l)	1599	0.08	-0.30	0.45	0.689
Free cholesterol in medium HDL (mmol/l)	1599	0.18	-0.21	0.57	0.370
Triglycerides in medium HDL (mmol/l)	1599	-0.30	-0.73	0.12	0.156
Concentration of small HDL particles (mol/l)	1599	-0.22	-0.60	0.15	0.242
Total lipids in small HDL (mmol/l)	1599	-0.10	-0.46	0.25	0.570
Phospholipids in small HDL (mmol/l)	1599	-0.23	-0.61	0.14	0.227
Total cholesterol in small HDL (mmol/l)	1599	0.12	-0.24	0.47	0.524
Cholesterol esters in small HDL (mmol/l)	1599	0.15	-0.21	0.51	0.410
Free cholesterol in small HDL (mmol/l)	1599	-0.10	-0.46	0.25	0.566
Triglycerides in small HDL (mmol/l)	1599	-0.23	-0.63	0.17	0.254
Phospholipids to total lipds ratio in chylomicrons and extremely large VLDL (%)	1599	-0.31	-0.74	0.12	0.158
Total cholesterol to total lipids ratio in chylomicrons and extremely large VLDL (%)	1599	-0.19	-0.56	0.17	0.300
Cholesterol esters to total lipids ratio in chylomicrons and extremely large VLDL (%)	1599	-0.06	-0.40	0.28	0.723
Free cholesterol to total lipids ratio in chylomicrons and extremely large VLDL (%)	1599	-0.46	-0.93	0.01	0.056
Triglycerides to total lipids ratio in chylomicrons and extremely large VLDL (%)	1599	0.05	-0.13	0.24	0.569
Phospholipids to total lipds ratio in very large VLDL (%)	1599	-0.18	-0.57	0.20	0.356
Total cholesterol to total lipids ratio in very large VLDL (%)	1599	0.08	-0.02	0.18	0.127
Cholesterol esters to total lipids ratio in very large VLDL (%)	1599	0.41	-0.02	0.84	0.061
Free cholesterol to total lipids ratio in very large VLDL (%)	1599	0.09	-0.10	0.27	0.352
Triglycerides to total lipids ratio in very large VLDL (%)	1599	-0.30	-0.69	0.10	0.143
Phospholipids to total lipds ratio in large VLDL (%)	1599	-0.28	-0.74	0.18	0.231
Total cholesterol to total lipids ratio in large VLDL (%)	1599	-0.12	-0.49	0.25	0.531
Cholesterol esters to total lipids ratio in large VLDL (%)	1599	-0.03	-0.24	0.18	0.770
Free cholesterol to total lipids ratio in large VLDL (%)	1599	-0.08	-0.23	0.07	0.287
Triglycerides to total lipids ratio in large VLDL (%)	1599	0.48	-0.13	1.09	0.121
Phospholipids to total lipds ratio in medium VLDL (%)	1599	0.19	-0.20	0.58	0.350
Total cholesterol to total lipids ratio in medium VLDL (%)	1599	0.05	-0.32	0.42	0.792
Cholesterol esters to total lipids ratio in medium VLDL (%)	1599	0.11	-0.25	0.48	0.549
Free cholesterol to total lipids ratio in medium VLDL (%)	1599	-0.20	-0.61	0.22	0.352
Triglycerides to total lipids ratio in medium VLDL (%)	1599	-0.11	-0.48	0.27	0.575
Phospholipids to total lipds ratio in small VLDL (%)	1599	0.12	-0.26	0.50	0.529
Total cholesterol to total lipids ratio in small VLDL (%)	1599	0.25	-0.14	0.64	0.211
Cholesterol esters to total lipids ratio in small VLDL (%)	1599	0.24	-0.15	0.63	0.229
Free cholesterol to total lipids ratio in small VLDL (%)	1599	0.28	-0.12	0.67	0.167
Triglycerides to total lipids ratio in small VLDL (%)	1599	-0.26	-0.66	0.14	0.203
Phospholipids to total lipds ratio in very small VLDL (%)	1599	0.11	-0.28	0.50	0.574
Total cholesterol to total lipids ratio in very small VLDL (%)	1599	0.20	-0.19	0.58	0.318
Cholesterol esters to total lipids ratio in very small VLDL (%)	1599	0.12	-0.25	0.49	0.523
Free cholesterol to total lipids ratio in very small VLDL (%)	1599	0.23	-0.16	0.61	0.254
Triglycerides to total lipids ratio in very small VLDL (%)	1599	-0.27	-0.66	0.13	0.183
Phospholipids to total lipds ratio in IDL (%)	1599	-0.23	-0.61	0.16	0.247
Total cholesterol to total lipids ratio in IDL (%)	1599	0.28	-0.12	0.68	0.166

S12 Table Negative control one-sample MR estimates of associations of age at voice breaking (per year later) with adiposity and cardiometabolic traits at age 8y among males in ALSPAC, using a refined GRS of 115 SNPs for age at menarche/voice breaking

	Unadj.				
**Standardised outcome at age 8y**	**N**	**Beta (2SLS)**	**LCL**	**UCL**	**P-value**
Cholesterol esters to total lipids ratio in IDL (%)	1599	0.22	-0.16	0.60	0.259
Free cholesterol to total lipids ratio in IDL (%)	1599	0.14	-0.24	0.52	0.478
Triglycerides to total lipids ratio in IDL (%)	1599	-0.24	-0.64	0.15	0.230
Phospholipids to total lipds ratio in large LDL (%)	1599	-0.22	-0.70	0.26	0.373
Total cholesterol to total lipids ratio in large LDL (%)	1599	0.30	-0.14	0.75	0.183
Cholesterol esters to total lipids ratio in large LDL (%)	1599	0.25	-0.21	0.71	0.291
Free cholesterol to total lipids ratio in large LDL (%)	1599	0.09	-0.33	0.50	0.685
Triglycerides to total lipids ratio in large LDL (%)	1599	-0.25	-0.64	0.14	0.214
Phospholipids to total lipds ratio in medium LDL (%)	1599	-0.05	-0.46	0.36	0.813
Total cholesterol to total lipids ratio in medium LDL (%)	1599	0.17	-0.25	0.58	0.430
Cholesterol esters to total lipids ratio in medium LDL (%)	1599	0.10	-0.32	0.52	0.640
Free cholesterol to total lipids ratio in medium LDL (%)	1599	0.03	-0.40	0.46	0.893
Triglycerides to total lipids ratio in medium LDL (%)	1599	-0.25	-0.64	0.15	0.221
Phospholipids to total lipds ratio in small LDL (%)	1599	-0.06	-0.47	0.35	0.778
Total cholesterol to total lipids ratio in small LDL (%)	1599	0.15	-0.25	0.56	0.453
Cholesterol esters to total lipids ratio in small LDL (%)	1599	0.06	-0.34	0.47	0.753
Free cholesterol to total lipids ratio in small LDL (%)	1599	0.11	-0.30	0.52	0.604
Triglycerides to total lipids ratio in small LDL (%)	1599	-0.25	-0.66	0.16	0.225
Phospholipids to total lipds ratio in very large HDL (%)	1599	0.01	-0.34	0.36	0.956
Total cholesterol to total lipids ratio in very large HDL (%)	1599	-0.02	-0.36	0.33	0.925
Cholesterol esters to total lipids ratio in very large HDL (%)	1599	-0.02	-0.37	0.33	0.913
Free cholesterol to total lipids ratio in very large HDL (%)	1599	0.03	-0.33	0.38	0.878
Triglycerides to total lipids ratio in very large HDL (%)	1599	0.02	-0.35	0.39	0.909
Phospholipids to total lipds ratio in large HDL (%)	1599	-0.27	-0.68	0.14	0.198
Total cholesterol to total lipids ratio in large HDL (%)	1599	0.23	-0.17	0.64	0.255
Cholesterol esters to total lipids ratio in large HDL (%)	1599	0.16	-0.23	0.55	0.432
Free cholesterol to total lipids ratio in large HDL (%)	1599	0.44	0.00	0.89	0.051
Triglycerides to total lipids ratio in large HDL (%)	1599	-0.10	-0.46	0.26	0.592
Phospholipids to total lipds ratio in medium HDL (%)	1599	0.00	-0.33	0.33	0.992
Total cholesterol to total lipids ratio in medium HDL (%)	1599	0.20	-0.18	0.57	0.299
Cholesterol esters to total lipids ratio in medium HDL (%)	1599	0.12	-0.24	0.48	0.511
Free cholesterol to total lipids ratio in medium HDL (%)	1599	0.49	0.03	0.95	0.037
Triglycerides to total lipids ratio in medium HDL (%)	1599	-0.31	-0.73	0.11	0.143
Phospholipids to total lipds ratio in small HDL (%)	1599	-0.22	-0.59	0.15	0.241
Total cholesterol to total lipids ratio in small HDL (%)	1599	0.24	-0.14	0.62	0.212
Cholesterol esters to total lipids ratio in small HDL (%)	1599	0.23	-0.15	0.61	0.228
Free cholesterol to total lipids ratio in small HDL (%)	1599	-0.03	-0.41	0.34	0.855
Triglycerides to total lipids ratio in small HDL (%)	1599	-0.19	-0.58	0.20	0.333
Mean diameter for VLDL particles (nm)	1599	-0.24	-0.63	0.16	0.243
Mean diameter for LDL particles (nm)	1599	0.11	-0.27	0.49	0.576
Mean diameter for HDL particles (nm)	1599	0.22	-0.16	0.59	0.263
Serum total cholesterol (mmol/l)	1599	0.17	-0.19	0.53	0.349
Total cholesterol in VLDL (mmol/l)	1599	-0.03	-0.39	0.33	0.883
Remnant cholesterol (non-HDL, non-LDL -cholesterol) (mmol/l)	1599	0.08	-0.28	0.43	0.668
Total cholesterol in LDL (mmol/l)	1599	0.12	-0.23	0.48	0.490
Total cholesterol in HDL (mmol/l)	1599	0.20	-0.17	0.58	0.290
Total cholesterol in HDL2 (mmol/l)	1599	0.20	-0.18	0.57	0.299
Total cholesterol in HDL3 (mmol/l)	1599	0.20	-0.18	0.57	0.303
Esterified cholesterol (mmol/l)	1598	0.14	-0.21	0.49	0.422
Free cholesterol (mmol/l)	1597	0.29	-0.08	0.66	0.122
Serum total triglycerides (mmol/l)	1599	-0.15	-0.53	0.22	0.423
Triglycerides in VLDL (mmol/l)	1599	-0.15	-0.52	0.22	0.430
Triglycerides in LDL (mmol/l)	1599	-0.09	-0.46	0.27	0.621
Triglycerides in HDL (mmol/l)	1599	-0.18	-0.57	0.21	0.369
Diacylglycerol (mmol/l)	1561	-0.12	-0.45	0.20	0.462
Ratio of diacylglycerol to triglycerides	1561	-0.18	-0.52	0.15	0.274
Total phosphoglycerides (mmol/l)	1597	0.13	-0.22	0.47	0.478
Ratio of triglycerides to phosphoglycerides	1597	-0.18	-0.55	0.18	0.325
Phosphatidylcholine and other cholines (mmol/l)	1593	0.12	-0.24	0.47	0.513
Total cholines (mmol/l)	1598	0.19	-0.17	0.54	0.300
Apolipoprotein A-I (g/l)	1599	0.17	-0.20	0.54	0.370
Apolipoprotein B (g/l)	1599	-0.03	-0.39	0.34	0.885
Ratio of apolipoprotein B to apolipoprotein A-I	1599	-0.09	-0.46	0.28	0.627
Total fatty acids (mmol/l)	1598	0.07	-0.28	0.42	0.698
Estimated description of fatty acid chain length, not actual carbon number	1597	-0.26	-0.67	0.16	0.226
Estimated degree of unsaturation	1597	-0.20	-0.61	0.22	0.353
22:6, docosahexaenoic acid (mmol/l)	1598	-0.15	-0.52	0.21	0.414
18:2, linoleic acid (mmol/l)	1598	0.00	-0.35	0.35	0.998

S12 Table Negative control one-sample MR estimates of associations of age at voice breaking (per year later) with adiposity and cardiometabolic traits at age 8y among males in ALSPAC, using a refined GRS of 115 SNPs for age at menarche/voice breaking

Unadj.

Standardised outcome at age 8y	N	Beta (2SLS)	LCL	UCL	P-value
Conjugated linoleic acid (mmol/l)	1597	-0.11	-0.47	0.24	0.524
Omega-3 fatty acids (mmol/l)	1598	-0.08	-0.43	0.28	0.680
Omega-6 fatty acids (mmol/l)	1598	0.05	-0.30	0.40	0.786
Polyunsaturated fatty acids (mmol/l)	1598	0.03	-0.31	0.38	0.845
Monounsaturated fatty acids; 16:1, 18:1 (mmol/l)	1597	0.06	-0.29	0.42	0.732
Saturated fatty acids (mmol/l)	1597	0.09	-0.28	0.47	0.616
Ratio of 22:6 docosahexaenoic acid to total fatty acids (%)	1598	-0.20	-0.59	0.20	0.325
Ratio of 18:2 linoleic acid to total fatty acids (%)	1598	-0.08	-0.45	0.30	0.687
Ratio of conjugated linoleic acid to total fatty acids (%)	1597	-0.15	-0.52	0.22	0.429
Ratio of omega-3 fatty acids to total fatty acids (%)	1598	-0.14	-0.53	0.24	0.469
Ratio of omega-6 fatty acids to total fatty acids (%)	1598	0.01	-0.36	0.37	0.977
Ratio of polyunsaturated fatty acids to total fatty acids (%)	1598	-0.02	-0.39	0.35	0.908
Ratio of monounsaturated fatty acids to total fatty acids (%)	1597	0.01	-0.36	0.37	0.976
Ratio of saturated fatty acids to total fatty acids (%)	1597	0.05	-0.34	0.43	0.820
Glucose (mmol/l)	1594	-0.25	-0.63	0.13	0.193
Lactate (mmol/l)	1600	0.00	-0.30	0.31	0.980
Pyruvate (mmol/l)	1598	-0.05	-0.39	0.29	0.768
Citrate (mmol/l)	1598	0.19	-0.19	0.56	0.325
Alanine (mmol/l)	1599	-0.03	-0.40	0.34	0.887
Glutamine (mmol/l)	1598	-0.15	-0.51	0.22	0.425
Histidine (mmol/l)	1599	-0.12	-0.47	0.22	0.490
Isoleucine (mmol/l)	1600	-0.15	-0.50	0.20	0.395
Leucine (mmol/l)	1599	-0.20	-0.57	0.16	0.267
Valine (mmol/l)	1600	-0.13	-0.49	0.23	0.478
Phenylalanine (mmol/l)	1597	-0.35	-0.79	0.09	0.119
Tyrosine (mmol/l)	1596	0.00	-0.35	0.35	0.999
Acetate (mmol/l)	1600	-0.41	-0.87	0.04	0.077
Acetoacetate (mmol/l)	1599	0.07	-0.28	0.42	0.682
3-hydroxybutyrate (mmol/l)	1599	0.20	-0.15	0.54	0.268
Creatinine (mmol/l)	1597	0.06	-0.27	0.40	0.712
Albumin (signal area)	1599	0.12	-0.31	0.55	0.580
Glycoprotein acetyls, mainly a1-acid glycoprotein (mmol/l)	1599	-0.11	-0.46	0.23	0.526

Complete case sample

Unadj.

Standardised outcome at age 8y	N	Beta (2SLS)	LCL	UCL	P-value
Body mass index (kg/m^2)	564	-0.07	-0.44	0.31	0.730
Fat mass index (kg/m^2)	564	-0.11	-0.50	0.28	0.571
Lean mass index (kg/m^2)	564	0.03	-0.32	0.38	0.872
Systolic blood pressure (mmHg)	564	0.16	-0.29	0.61	0.497
Diastolic blood pressure (mmHg)	564	0.19	-0.26	0.64	0.411
Concentration of chylomicrons and extremely large VLDL particles (mol/l)	564	-0.05	-0.45	0.35	0.805
Total lipids in chylomicrons and extremely large VLDL (mmol/l)	564	-0.05	-0.46	0.35	0.796
Phospholipids in chylomicrons and extremely large VLDL (mmol/l)	564	-0.06	-0.47	0.34	0.756
Total cholesterol in chylomicrons and extremely large VLDL (mmol/l)	564	-0.06	-0.46	0.34	0.758
Cholesterol esters in chylomicrons and extremely large VLDL (mmol/l)	564	-0.06	-0.46	0.35	0.789
Free cholesterol in chylomicrons and extremely large VLDL (mmol/l)	564	-0.07	-0.47	0.33	0.741
Triglycerides in chylomicrons and extremely large VLDL (mmol/l)	564	-0.05	-0.46	0.36	0.812
Concentration of very large VLDL particles (mol/l)	564	-0.07	-0.47	0.33	0.726
Total lipids in very large VLDL (mmol/l)	564	-0.07	-0.48	0.33	0.716
Phospholipids in very large VLDL (mmol/l)	564	-0.06	-0.46	0.34	0.757
Total cholesterol in very large VLDL (mmol/l)	564	-0.05	-0.46	0.35	0.790
Cholesterol esters in very large VLDL (mmol/l)	564	-0.05	-0.45	0.36	0.814
Free cholesterol in very large VLDL (mmol/l)	564	-0.06	-0.46	0.34	0.768
Triglycerides in very large VLDL (mmol/l)	564	-0.08	-0.48	0.32	0.684
Concentration of large VLDL particles (mol/l)	564	-0.08	-0.48	0.32	0.683
Total lipids in large VLDL (mmol/l)	564	-0.08	-0.48	0.32	0.702
Phospholipids in large VLDL (mmol/l)	564	-0.07	-0.47	0.32	0.715
Total cholesterol in large VLDL (mmol/l)	564	-0.06	-0.46	0.34	0.765
Cholesterol esters in large VLDL (mmol/l)	564	-0.05	-0.45	0.36	0.827
Free cholesterol in large VLDL (mmol/l)	564	-0.07	-0.47	0.32	0.716
Triglycerides in large VLDL (mmol/l)	564	-0.09	-0.49	0.32	0.676
Concentration of medium VLDL particles (mol/l)	564	-0.06	-0.46	0.34	0.768
Total lipids in medium VLDL (mmol/l)	564	-0.06	-0.46	0.35	0.783
Phospholipids in medium VLDL (mmol/l)	564	-0.05	-0.45	0.36	0.815
Total cholesterol in medium VLDL (mmol/l)	564	-0.01	-0.43	0.41	0.961

S12 Table Negative control one-sample MR estimates of associations of age at voice breaking (per year later) with adiposity and cardiometabolic traits at age 8y among males in ALSPAC, using a refined GRS of 115 SNPs for age at menarche/voice breaking

	Unadj.				
**Standardised outcome at age 8y**	**N**	**Beta (2SLS)**	**LCL**	**UCL**	**P-value**
Cholesterol esters in medium VLDL (mmol/l)	564	0.03	-0.41	0.46	0.908
Free cholesterol in medium VLDL (mmol/l)	564	-0.05	-0.45	0.35	0.802
Triglycerides in medium VLDL (mmol/l)	564	-0.08	-0.48	0.32	0.697
Concentration of small VLDL particles (mol/l)	564	0.01	-0.41	0.43	0.962
Total lipids in small VLDL (mmol/l)	564	0.04	-0.40	0.47	0.869
Phospholipids in small VLDL (mmol/l)	564	0.03	-0.40	0.47	0.876
Total cholesterol in small VLDL (mmol/l)	564	0.12	-0.34	0.58	0.618
Cholesterol esters in small VLDL (mmol/l)	564	0.14	-0.32	0.61	0.549
Free cholesterol in small VLDL (mmol/l)	564	0.06	-0.37	0.50	0.773
Triglycerides in small VLDL (mmol/l)	564	-0.03	-0.44	0.38	0.893
Concentration of very small VLDL particles (mol/l)	564	0.23	-0.22	0.67	0.321
Total lipids in very small VLDL (mmol/l)	564	0.24	-0.21	0.69	0.298
Phospholipids in very small VLDL (mmol/l)	564	0.19	-0.25	0.63	0.393
Total cholesterol in very small VLDL (mmol/l)	564	0.25	-0.20	0.70	0.279
Cholesterol esters in very small VLDL (mmol/l)	564	0.24	-0.21	0.69	0.290
Free cholesterol in very small VLDL (mmol/l)	564	0.23	-0.21	0.68	0.302
Triglycerides in very small VLDL (mmol/l)	564	0.09	-0.32	0.51	0.654
Concentration of IDL particles (mol/l)	564	0.16	-0.26	0.58	0.459
Total lipids in IDL (mmol/l)	564	0.17	-0.25	0.59	0.422
Phospholipids in IDL (mmol/l)	564	0.13	-0.29	0.54	0.551
Total cholesterol in IDL (mmol/l)	564	0.17	-0.25	0.60	0.419
Cholesterol esters in IDL (mmol/l)	564	0.18	-0.25	0.61	0.417
Free cholesterol in IDL (mmol/l)	564	0.16	-0.26	0.58	0.453
Triglycerides in IDL (mmol/l)	564	0.16	-0.24	0.56	0.432
Concentration of large LDL particles (mol/l)	564	0.09	-0.31	0.50	0.650
Total lipids in large LDL (mmol/l)	564	0.13	-0.28	0.54	0.530
Phospholipids in large LDL (mmol/l)	564	0.12	-0.29	0.53	0.569
Total cholesterol in large LDL (mmol/l)	564	0.13	-0.28	0.54	0.527
Cholesterol esters in large LDL (mmol/l)	564	0.12	-0.29	0.53	0.554
Free cholesterol in large LDL (mmol/l)	564	0.15	-0.26	0.56	0.461
Triglycerides in large LDL (mmol/l)	564	0.10	-0.30	0.50	0.631
Concentration of medium LDL particles (mol/l)	564	0.06	-0.34	0.46	0.772
Total lipids in medium LDL (mmol/l)	564	0.11	-0.30	0.51	0.600
Phospholipids in medium LDL (mmol/l)	564	0.12	-0.29	0.53	0.562
Total cholesterol in medium LDL (mmol/l)	564	0.11	-0.30	0.52	0.593
Cholesterol esters in medium LDL (mmol/l)	564	0.10	-0.31	0.51	0.639
Free cholesterol in medium LDL (mmol/l)	564	0.17	-0.24	0.58	0.416
Triglycerides in medium LDL (mmol/l)	564	0.03	-0.36	0.43	0.862
Concentration of small LDL particles (mol/l)	564	0.05	-0.34	0.45	0.786
Total lipids in small LDL (mmol/l)	564	0.10	-0.31	0.50	0.641
Phospholipids in small LDL (mmol/l)	564	0.09	-0.31	0.48	0.670
Total cholesterol in small LDL (mmol/l)	564	0.11	-0.30	0.51	0.607
Cholesterol esters in small LDL (mmol/l)	564	0.08	-0.32	0.48	0.697
Free cholesterol in small LDL (mmol/l)	564	0.20	-0.22	0.62	0.346
Triglycerides in small LDL (mmol/l)	564	0.00	-0.39	0.39	0.988
Concentration of very large HDL particles (mol/l)	564	0.01	-0.45	0.48	0.952
Total lipids in very large HDL (mmol/l)	564	0.02	-0.46	0.49	0.944
Phospholipids in very large HDL (mmol/l)	564	0.00	-0.47	0.47	1.000
Total cholesterol in very large HDL (mmol/l)	564	0.03	-0.44	0.50	0.906
Cholesterol esters in very large HDL (mmol/l)	564	0.03	-0.44	0.51	0.895
Free cholesterol in very large HDL (mmol/l)	564	0.02	-0.45	0.48	0.945
Triglycerides in very large HDL (mmol/l)	564	0.11	-0.32	0.54	0.612
Concentration of large HDL particles (mol/l)	564	-0.05	-0.52	0.41	0.825
Total lipids in large HDL (mmol/l)	564	-0.04	-0.50	0.42	0.861
Phospholipids in large HDL (mmol/l)	564	-0.07	-0.53	0.39	0.769
Total cholesterol in large HDL (mmol/l)	564	-0.02	-0.48	0.45	0.942
Cholesterol esters in large HDL (mmol/l)	564	-0.02	-0.49	0.45	0.931
Free cholesterol in large HDL (mmol/l)	564	0.00	-0.46	0.47	0.989
Triglycerides in large HDL (mmol/l)	564	-0.02	-0.43	0.40	0.931
Concentration of medium HDL particles (mol/l)	564	-0.28	-0.74	0.18	0.232
Total lipids in medium HDL (mmol/l)	564	-0.28	-0.75	0.18	0.231
Phospholipids in medium HDL (mmol/l)	564	-0.22	-0.66	0.22	0.321
Total cholesterol in medium HDL (mmol/l)	564	-0.31	-0.80	0.18	0.213
Cholesterol esters in medium HDL (mmol/l)	564	-0.33	-0.82	0.16	0.189
Free cholesterol in medium HDL (mmol/l)	564	-0.18	-0.64	0.28	0.438
Triglycerides in medium HDL (mmol/l)	564	-0.19	-0.67	0.28	0.421
Concentration of small HDL particles (mol/l)	564	-0.32	-0.77	0.13	0.165
Total lipids in small HDL (mmol/l)	564	-0.25	-0.69	0.19	0.262

S12 Table Negative control one-sample MR estimates of associations of age at voice breaking (per year later) with adiposity and cardiometabolic traits at age 8y among males in ALSPAC, using a refined GRS of 115 SNPs for age at menarche/voice breaking

	Unadj.				
**Standardised outcome at age 8y**	**N**	**Beta (2SLS)**	**LCL**	**UCL**	**P-value**
Phospholipids in small HDL (mmol/l)	564	-0.33	-0.78	0.12	0.152
Total cholesterol in small HDL (mmol/l)	564	-0.06	-0.50	0.37	0.770
Cholesterol esters in small HDL (mmol/l)	564	-0.01	-0.45	0.42	0.954
Free cholesterol in small HDL (mmol/l)	564	-0.25	-0.69	0.18	0.254
Triglycerides in small HDL (mmol/l)	564	-0.03	-0.44	0.37	0.877
Phospholipids to total lipds ratio in chylomicrons and extremely large VLDL (%)	564	-0.23	-0.59	0.13	0.213
Total cholesterol to total lipids ratio in chylomicrons and extremely large VLDL (%)	564	-0.09	-0.53	0.34	0.671
Cholesterol esters to total lipids ratio in chylomicrons and extremely large VLDL (%)	564	-0.05	-0.48	0.38	0.831
Free cholesterol to total lipids ratio in chylomicrons and extremely large VLDL (%)	564	-0.17	-0.60	0.26	0.434
Triglycerides to total lipids ratio in chylomicrons and extremely large VLDL (%)	564	0.09	-0.09	0.27	0.344
Phospholipids to total lipds ratio in very large VLDL (%)	564	-0.12	-0.57	0.32	0.591
Total cholesterol to total lipids ratio in very large VLDL (%)	564	-0.01	-0.10	0.09	0.910
Cholesterol esters to total lipids ratio in very large VLDL (%)	564	0.08	-0.25	0.42	0.625
Free cholesterol to total lipids ratio in very large VLDL (%)	564	-0.11	-0.34	0.12	0.359
Triglycerides to total lipids ratio in very large VLDL (%)	564	0.06	-0.37	0.50	0.773
Phospholipids to total lipds ratio in large VLDL (%)	564	0.01	-0.46	0.49	0.951
Total cholesterol to total lipids ratio in large VLDL (%)	564	-0.06	-0.53	0.42	0.809
Cholesterol esters to total lipids ratio in large VLDL (%)	564	-0.05	-0.36	0.25	0.735
Free cholesterol to total lipids ratio in large VLDL (%)	564	0.02	-0.14	0.18	0.792
Triglycerides to total lipids ratio in large VLDL (%)	564	0.08	-0.30	0.45	0.687
Phospholipids to total lipds ratio in medium VLDL (%)	564	0.02	-0.48	0.52	0.940
Total cholesterol to total lipids ratio in medium VLDL (%)	564	0.00	-0.44	0.44	1.000
Cholesterol esters to total lipids ratio in medium VLDL (%)	564	-0.02	-0.46	0.41	0.915
Free cholesterol to total lipids ratio in medium VLDL (%)	564	0.05	-0.38	0.47	0.833
Triglycerides to total lipids ratio in medium VLDL (%)	564	-0.02	-0.48	0.43	0.917
Phospholipids to total lipds ratio in small VLDL (%)	564	-0.11	-0.58	0.36	0.648
Total cholesterol to total lipids ratio in small VLDL (%)	564	0.11	-0.30	0.52	0.599
Cholesterol esters to total lipids ratio in small VLDL (%)	564	0.12	-0.29	0.53	0.574
Free cholesterol to total lipids ratio in small VLDL (%)	564	0.07	-0.37	0.52	0.749
Triglycerides to total lipids ratio in small VLDL (%)	564	-0.08	-0.49	0.33	0.709
Phospholipids to total lipds ratio in very small VLDL (%)	564	-0.03	-0.50	0.44	0.903
Total cholesterol to total lipids ratio in very small VLDL (%)	564	0.10	-0.32	0.52	0.634
Cholesterol esters to total lipids ratio in very small VLDL (%)	564	0.08	-0.33	0.49	0.700
Free cholesterol to total lipids ratio in very small VLDL (%)	564	0.10	-0.34	0.54	0.664
Triglycerides to total lipids ratio in very small VLDL (%)	564	-0.09	-0.51	0.33	0.671
Phospholipids to total lipds ratio in IDL (%)	564	-0.21	-0.66	0.23	0.343
Total cholesterol to total lipids ratio in IDL (%)	564	0.05	-0.37	0.48	0.801
Cholesterol esters to total lipids ratio in IDL (%)	564	0.06	-0.36	0.48	0.778
Free cholesterol to total lipids ratio in IDL (%)	564	-0.01	-0.50	0.47	0.957
Triglycerides to total lipids ratio in IDL (%)	564	0.04	-0.40	0.49	0.843
Phospholipids to total lipds ratio in large LDL (%)	564	-0.01	-0.52	0.49	0.954
Total cholesterol to total lipids ratio in large LDL (%)	564	0.00	-0.47	0.48	0.995
Cholesterol esters to total lipids ratio in large LDL (%)	564	-0.04	-0.53	0.45	0.878
Free cholesterol to total lipids ratio in large LDL (%)	564	0.10	-0.37	0.57	0.668
Triglycerides to total lipids ratio in large LDL (%)	564	0.00	-0.43	0.44	0.983
Phospholipids to total lipds ratio in medium LDL (%)	564	0.05	-0.46	0.56	0.837
Total cholesterol to total lipids ratio in medium LDL (%)	564	-0.03	-0.55	0.49	0.911
Cholesterol esters to total lipids ratio in medium LDL (%)	564	-0.05	-0.57	0.46	0.835
Free cholesterol to total lipids ratio in medium LDL (%)	564	0.09	-0.41	0.59	0.724
Triglycerides to total lipids ratio in medium LDL (%)	564	-0.02	-0.46	0.41	0.923
Phospholipids to total lipds ratio in small LDL (%)	564	0.01	-0.48	0.51	0.953
Total cholesterol to total lipids ratio in small LDL (%)	564	0.00	-0.49	0.50	0.987
Cholesterol esters to total lipids ratio in small LDL (%)	564	-0.05	-0.54	0.44	0.845
Free cholesterol to total lipids ratio in small LDL (%)	564	0.12	-0.36	0.60	0.628
Triglycerides to total lipids ratio in small LDL (%)	564	-0.03	-0.49	0.44	0.906
Phospholipids to total lipds ratio in very large HDL (%)	564	-0.15	-0.62	0.32	0.537
Total cholesterol to total lipids ratio in very large HDL (%)	564	0.13	-0.33	0.59	0.578
Cholesterol esters to total lipids ratio in very large HDL (%)	564	0.13	-0.34	0.59	0.591
Free cholesterol to total lipids ratio in very large HDL (%)	564	-0.01	-0.47	0.44	0.953
Triglycerides to total lipids ratio in very large HDL (%)	564	0.15	-0.36	0.67	0.552
Phospholipids to total lipds ratio in large HDL (%)	564	-0.15	-0.63	0.33	0.553
Total cholesterol to total lipids ratio in large HDL (%)	564	0.07	-0.44	0.58	0.785
Cholesterol esters to total lipids ratio in large HDL (%)	564	0.03	-0.48	0.54	0.900
Free cholesterol to total lipids ratio in large HDL (%)	564	0.21	-0.27	0.69	0.384
Triglycerides to total lipids ratio in large HDL (%)	564	0.11	-0.42	0.64	0.673
Phospholipids to total lipds ratio in medium HDL (%)	564	0.04	-0.37	0.45	0.834
Total cholesterol to total lipids ratio in medium HDL (%)	564	0.00	-0.41	0.41	0.996
Cholesterol esters to total lipids ratio in medium HDL (%)	564	-0.05	-0.47	0.37	0.811

S12 Table Negative control one-sample MR estimates of associations of age at voice breaking (per year later) with adiposity and cardiometabolic traits at age 8y among males in ALSPAC, using a refined GRS of 115 SNPs for age at menarche/voice breaking

	Unadj.				
**Standardised outcome at age 8y**	**N**	**Beta (2SLS)**	**LCL**	**UCL**	**P-value**
Free cholesterol to total lipids ratio in medium HDL (%)	564	0.30	-0.18	0.78	0.220
Triglycerides to total lipids ratio in medium HDL (%)	564	-0.08	-0.55	0.39	0.731
Phospholipids to total lipds ratio in small HDL (%)	564	-0.13	-0.58	0.31	0.554
Total cholesterol to total lipids ratio in small HDL (%)	564	0.09	-0.34	0.53	0.673
Cholesterol esters to total lipids ratio in small HDL (%)	564	0.10	-0.34	0.54	0.663
Free cholesterol to total lipids ratio in small HDL (%)	564	-0.08	-0.53	0.38	0.739
Triglycerides to total lipids ratio in small HDL (%)	564	0.06	-0.36	0.47	0.791
Mean diameter for VLDL particles (nm)	564	-0.10	-0.52	0.32	0.647
Mean diameter for LDL particles (nm)	564	0.18	-0.29	0.64	0.459
Mean diameter for HDL particles (nm)	564	0.03	-0.44	0.50	0.910
Serum total cholesterol (mmol/l)	564	0.10	-0.31	0.50	0.641
Total cholesterol in VLDL (mmol/l)	564	0.08	-0.35	0.51	0.716
Remnant cholesterol (non-HDL, non-LDL -cholesterol) (mmol/l)	564	0.14	-0.30	0.57	0.538
Total cholesterol in LDL (mmol/l)	564	0.12	-0.29	0.53	0.561
Total cholesterol in HDL (mmol/l)	564	-0.07	-0.53	0.39	0.755
Total cholesterol in HDL2 (mmol/l)	564	-0.07	-0.53	0.39	0.752
Total cholesterol in HDL3 (mmol/l)	564	-0.07	-0.52	0.38	0.770
Esterified cholesterol (mmol/l)	564	0.05	-0.36	0.45	0.817
Free cholesterol (mmol/l)	564	0.21	-0.21	0.63	0.335
Serum total triglycerides (mmol/l)	564	-0.04	-0.44	0.36	0.839
Triglycerides in VLDL (mmol/l)	564	-0.06	-0.46	0.34	0.759
Triglycerides in LDL (mmol/l)	564	0.06	-0.34	0.46	0.766
Triglycerides in HDL (mmol/l)	564	-0.06	-0.48	0.35	0.766
Diacylglycerol (mmol/l)	564	0.05	-0.30	0.41	0.765
Ratio of diacylglycerol to triglycerides	564	0.02	-0.34	0.39	0.895
Total phosphoglycerides (mmol/l)	564	0.05	-0.36	0.46	0.812
Ratio of triglycerides to phosphoglycerides	564	-0.07	-0.50	0.35	0.731
Phosphatidylcholine and other cholines (mmol/l)	564	-0.04	-0.46	0.39	0.865
Total cholines (mmol/l)	564	0.09	-0.32	0.50	0.675
Apolipoprotein A-I (g/l)	564	-0.07	-0.52	0.37	0.740
Apolipoprotein B (g/l)	564	0.09	-0.34	0.51	0.683
Ratio of apolipoprotein B to apolipoprotein A-I	564	0.12	-0.34	0.59	0.603
Total fatty acids (mmol/l)	564	0.05	-0.35	0.46	0.797
Estimated description of fatty acid chain length, not actual carbon number	564	-0.16	-0.60	0.28	0.485
Estimated degree of unsaturation	564	-0.32	-0.84	0.20	0.226
22:6, docosahexaenoic acid (mmol/l)	564	-0.39	-0.88	0.11	0.126
18:2, linoleic acid (mmol/l)	564	-0.09	-0.53	0.34	0.676
Conjugated linoleic acid (mmol/l)	564	-0.17	-0.62	0.28	0.464
Omega-3 fatty acids (mmol/l)	564	-0.03	-0.47	0.40	0.877
Omega-6 fatty acids (mmol/l)	564	-0.04	-0.46	0.38	0.847
Polyunsaturated fatty acids (mmol/l)	564	-0.04	-0.46	0.38	0.843
Monounsaturated fatty acids; 16:1, 18:1 (mmol/l)	564	0.15	-0.25	0.56	0.455
Saturated fatty acids (mmol/l)	564	0.03	-0.39	0.45	0.886
Ratio of 22:6 docosahexaenoic acid to total fatty acids (%)	564	-0.54	-1.11	0.04	0.066
Ratio of 18:2 linoleic acid to total fatty acids (%)	564	-0.22	-0.69	0.25	0.367
Ratio of conjugated linoleic acid to total fatty acids (%)	564	-0.19	-0.68	0.30	0.456
Ratio of omega-3 fatty acids to total fatty acids (%)	564	-0.11	-0.57	0.35	0.638
Ratio of omega-6 fatty acids to total fatty acids (%)	564	-0.15	-0.60	0.30	0.513
Ratio of polyunsaturated fatty acids to total fatty acids (%)	564	-0.16	-0.61	0.28	0.474
Ratio of monounsaturated fatty acids to total fatty acids (%)	564	0.20	-0.25	0.64	0.380
Ratio of saturated fatty acids to total fatty acids (%)	564	-0.05	-0.51	0.41	0.830
Glucose (mmol/l)	564	-0.35	-0.82	0.12	0.145
Lactate (mmol/l)	564	-0.01	-0.34	0.33	0.975
Pyruvate (mmol/l)	564	0.15	-0.26	0.57	0.472
Citrate (mmol/l)	564	0.32	-0.14	0.77	0.172
Alanine (mmol/l)	564	-0.07	-0.51	0.37	0.751
Glutamine (mmol/l)	564	-0.17	-0.63	0.29	0.480
Histidine (mmol/l)	564	-0.16	-0.51	0.19	0.361
Isoleucine (mmol/l)	564	-0.37	-0.83	0.10	0.121
Leucine (mmol/l)	564	-0.37	-0.84	0.10	0.123
Valine (mmol/l)	564	-0.43	-0.94	0.08	0.096
Phenylalanine (mmol/l)	564	-0.49	-1.09	0.11	0.109
Tyrosine (mmol/l)	564	-0.13	-0.55	0.30	0.565
Acetate (mmol/l)	564	-0.47	-1.07	0.12	0.120
Acetoacetate (mmol/l)	564	-0.16	-0.57	0.24	0.433
3-hydroxybutyrate (mmol/l)	564	-0.12	-0.51	0.26	0.535
Creatinine (mmol/l)	564	0.07	-0.34	0.49	0.732
Albumin (signal area)	564	-0.02	-0.46	0.42	0.933

S12 Table Negative control one-sample MR estimates of associations of age at voice breaking (per year later) with adiposity and cardiometabolic traits at age 8y among males in ALSPAC, using a refined GRS of 115 SNPs for age at menarche/voice breaking

	Unadj.				
**Standardised outcome at age 8y**	**N**	**Beta (2SLS)**	**LCL**	**UCL**	**P-value**
Glycoprotein acetyls, mainly a1-acid glycoprotein (mmol/l)	564	0.11	-0.32	0.54	0.616
