# Supplementary material for: Influence of puberty timing on adiposity and cardiometabolic traits: A Mendelian randomisation study
Source: PLoS Med. 2018 Aug 28;15(8):e1002641. doi: 10.1371/journal.pmed.1002641 (PMC6112630; doi:10.1371/journal.pmed.1002641)
Supplement: S13 Table — (PDF) [file pmed.1002641.s032.pdf]

S13 Table One-sample Mendelian randomization estimates of body mass index with blood pressure and summary cardiometabolic traits at age 8y and 18y in ALSPAC, for sample power comparisons

	**Among females and males combined**			**Among females**			**Among males**		
	**N**	**Standardized beta (95% CI) per SD higher BMI**	**P-value**	**N**	**Standardized beta (95% CI) per SD higher BMI**	**P-value**	**N**	**Standardized beta (95% CI) per SD higher BMI**	**P-value**
**At age 8y**									
Systolic blood pressure	6275	0.47 (0.33, 0.61)	3.75E-11	3090	0.57 (0.38, 0.75)	1.12E-09	3180	0.37 (0.15, 0.58)	1.00E-03
Diastolic blood pressure	6274	0.35 (0.20, 0.50)	5.93E-06	3091	0.36 (0.17, 0.56)	1.90E-04	3178	0.34 (0.11, 0.58)	0.010
Triglycerides	4734	0.14 (-0.04, 0.32)	0.130	2294	0.23 (-0.001, 0.46)	0.050	2436	0.02 (-0.27, 0.30)	0.920
HDL cholesterol	4734	-0.17 (-0.33, 0.003)	0.050	2294	-0.22 (-0.43, -0.02)	0.030	2436	-0.09 (-0.37, 0.19)	0.540
LDL cholesterol	4734	-0.02 (-0.19, 0.15)	0.840	2294	0.03 (-0.18, 0.24)	0.800	2436	-0.08 (-0.34, 0.19)	0.570
Total cholesterol	4734	-0.01 (-0.17, 0.16)	0.940	2294	0.04 (-0.17, 0.25)	0.700	2436	-0.07 (-0.33, 0.20)	0.630
Glucose	4713	0.15 (-0.01, 0.32)	0.070	2282	0.16 (-0.04, 0.36)	0.110	2427	0.14 (-0.14, 0.41)	0.330
Glycoprotein acetyls	4735	0.24 (0.06, 0.42)	0.010	2295	0.31 (0.11, 0.52)	0.003	2436	0.15 (-0.15, 0.45)	0.330
**At age 18y**									
Systolic blood pressure	3609	0.23 (0.07, 0.40)	0.010	2014	0.10 (-0.08, 0.28)	0.270	1594	0.42 (0.20, 0.64)	2.06E-04
Diastolic blood pressure	3609	0.11 (-0.06, 0.27)	0.200	2014	0.15 (-0.07, 0.38)	0.190	1594	0.05 (-0.19, 0.29)	0.680
Triglycerides	2549	0.13 (-0.05, 0.31)	0.170	1312	0.12 (-0.12, 0.36)	0.320	1236	0.13 (-0.14, 0.41)	0.340
HDL cholesterol	2549	-0.11 (-0.32, 0.09)	0.280	1312	-0.11 (-0.38, 0.16)	0.420	1236	-0.09 (-0.34, 0.16)	0.480
LDL cholesterol	2549	-0.01 (-0.22, 0.19)	0.900	1312	0.04 (-0.23, 0.32)	0.770	1236	-0.05 (-0.33, 0.22)	0.710
Total cholesterol	2549	-0.02 (-0.22, 0.18)	0.840	1312	0.02 (-0.25, 0.28)	0.910	1236	-0.04 (-0.31, 0.23)	0.770
Glucose	2548	0.15 (-0.01, 0.31)	0.070	1311	0.09 (-0.10, 0.28)	0.340	1236	0.19 (-0.05, 0.44)	0.120
Glycoprotein acetyls	2548	0.17 (-0.03, 0.37)	0.100	1311	0.01 (-0.28, 0.30)	0.950	1236	0.35 (0.10, 0.61)	0.010
