# Supplementary material for: Influence of puberty timing on adiposity and cardiometabolic traits: A Mendelian randomisation study
Source: PLoS Med. 2018 Aug 28;15(8):e1002641. doi: 10.1371/journal.pmed.1002641 (PMC6112630; doi:10.1371/journal.pmed.1002641)
Supplement: S14 Table — (PDF) [file pmed.1002641.s033.pdf]

S14 Table Two-sample MR estimates of associations of puberty timing (per year later) with post-pubertal adiposity and cardiometabolic traits among males and females in GWAS data, using a full set of up to 303 SNPs for age at menarche

Standardised outcome in adulthood	# SNPs	Beta (IVW)	LCL	UCL	P-value
Body mass index	234	-0.17	-0.21	-0.12	4.00E-15
Concentration of chylomicrons and largest VLDL particles	303	0.00	-0.04	0.04	0.826
Total lipids in chylomicrons and largest VLDL particles	303	-0.02	-0.06	0.02	0.311
Phospholipids in chylomicrons and largest VLDL particles	303	-0.02	-0.05	0.02	0.366
Triglycerides in chylomicrons and largest VLDL particles	303	-0.02	-0.06	0.02	0.268
Concentration of very large VLDL particles	301	-0.04	-0.08	0.00	0.034
Total lipids in very large VLDL	303	-0.03	-0.07	0.01	0.094
Phospholipids in very large VLDL	303	-0.02	-0.06	0.01	0.233
Triglycerides in very large VLDL	303	-0.03	-0.07	0.01	0.118
Concentration of large VLDL particles	303	-0.02	-0.06	0.02	0.311
Total lipids in large VLDL	303	-0.02	-0.06	0.02	0.276
Phospholipids in large VLDL	303	-0.02	-0.06	0.02	0.258
Total cholesterol in large VLDL	303	-0.03	-0.07	0.01	0.144
Cholesterol esters in large VLDL	303	-0.03	-0.07	0.01	0.212
Free cholesterol in large VLDL	303	-0.03	-0.06	0.01	0.159
Triglycerides in large VLDL	303	-0.02	-0.06	0.01	0.231
Concentration of medium VLDL particles	303	-0.02	-0.06	0.02	0.236
Total lipids in medium VLDL	303	-0.02	-0.06	0.02	0.445
Phospholipids in medium VLDL	303	-0.02	-0.06	0.02	0.281
Total cholesterol in medium VLDL	303	-0.02	-0.06	0.02	0.244
Cholesterol esters in medium VLDL	303	-0.02	-0.06	0.02	0.332
Free cholesterol in medium VLDL	303	-0.02	-0.06	0.02	0.288
Triglycerides in medium VLDL	303	-0.02	-0.05	0.02	0.435
Concentration of small VLDL particles	303	-0.03	-0.07	0.02	0.235
Total lipids in small VLDL	303	-0.03	-0.07	0.02	0.241
Phospholipids in small VLDL	303	-0.03	-0.07	0.01	0.161
Total cholesterol in small VLDL	303	-0.03	-0.07	0.01	0.141
Free cholesterol in small VLDL	303	-0.03	-0.07	0.01	0.191
Triglycerides in small VLDL	303	-0.03	-0.07	0.01	0.163
Concentration of very small VLDL particles	303	-0.04	-0.08	0.00	0.062
Total lipids in very small VLDL	303	-0.04	-0.08	0.00	0.065
Phospholipids in very small VLDL	303	-0.03	-0.07	0.01	0.177
Triglycerides in very small VLDL	303	-0.04	-0.08	0.01	0.102
Concentration of IDL particles	303	-0.03	-0.07	0.01	0.144
Total lipids in IDL	303	-0.03	-0.07	0.01	0.191
Phospholipids in IDL	303	-0.03	-0.07	0.01	0.113
Total cholesterol in IDL	303	-0.02	-0.06	0.02	0.358
Free cholesterol in IDL	303	-0.03	-0.07	0.01	0.142
Triglycerides in IDL	303	-0.04	-0.08	0.00	0.063
Concentration of large LDL particles	303	-0.02	-0.06	0.02	0.290
Total lipids in large LDL	303	-0.02	-0.06	0.02	0.358
Phospholipids in large LDL	303	-0.02	-0.06	0.02	0.338
Total cholesterol in large LDL	303	-0.01	-0.06	0.03	0.475
Cholesterol esters in large LDL	303	-0.01	-0.06	0.03	0.525
Free cholesterol in large LDL	303	-0.02	-0.06	0.02	0.392
Concentration of medium LDL particles	303	-0.02	-0.06	0.02	0.372
Total lipids in medium LDL	303	-0.02	-0.06	0.02	0.377
Phospholipids in medium LDL	303	-0.01	-0.05	0.03	0.511
Total cholesterol in medium LDL	303	-0.01	-0.05	0.03	0.605
Cholesterol esters in medium LDL	303	-0.02	-0.06	0.03	0.476
Concentration of small LDL particles	303	-0.03	-0.07	0.02	0.224
Total lipids in small LDL	303	-0.02	-0.07	0.02	0.257
Total cholesterol in small LDL	303	-0.02	-0.06	0.02	0.400
Concentration of very large HDL particles	303	0.02	-0.03	0.06	0.456
Total lipids in very large HDL	303	0.01	-0.03	0.05	0.705
Phospholipids in very large HDL	303	0.02	-0.02	0.06	0.359
Total cholesterol in very large HDL	303	0.00	-0.04	0.03	0.814
Cholesterol esters in very large HDL	303	-0.01	-0.05	0.03	0.774
Free cholesterol in very large HDL	303	0.01	-0.03	0.05	0.553
Triglycerides in very large HDL	303	-0.01	-0.05	0.03	0.637
Concentration of large HDL particles	303	0.03	-0.01	0.08	0.112
Total lipids in large HDL	303	0.03	-0.01	0.08	0.110
Phospholipids in large HDL	303	0.03	-0.01	0.07	0.137
Total cholesterol in large HDL	303	0.03	-0.01	0.08	0.088
Cholesterol esters in large HDL	303	0.03	-0.01	0.08	0.115
Free cholesterol in large HDL	303	0.04	-0.01	0.08	0.087
Concentration of medium HDL particles	303	0.02	-0.02	0.06	0.454
Total lipids in medium HDL	303	0.02	-0.02	0.06	0.428
Phospholipids in medium HDL	303	0.01	-0.02	0.05	0.485
Total cholesterol in medium HDL	303	0.02	-0.02	0.06	0.282
Cholesterol esters in medium HDL	303	0.02	-0.02	0.06	0.314
Free cholesterol in medium HDL	303	0.02	-0.02	0.06	0.259

S14 Table Two-sample MR estimates of associations of puberty timing (per year later) with post-pubertal adiposity and cardiometabolic traits among males and females in GWAS data, using a full set of up to 303 SNPs for age at menarche

Standardised outcome in adulthood	# SNPs	Beta (IVW)	LCL	UCL	P-value
Concentration of small HDL particles	303	0.01	-0.03	0.04	0.794
Total lipids in small HDL	303	0.01	-0.03	0.05	0.731
Triglycerides in small HDL	303	-0.03	-0.07	0.01	0.164
Mean diameter for VLDL particles	303	-0.01	-0.05	0.03	0.680
Mean diameter for LDL particles	303	-0.01	-0.04	0.03	0.769
Mean diameter for HDL particles	303	0.02	-0.02	0.06	0.306
Serum total cholesterol	303	-0.01	-0.05	0.03	0.520
Total cholesterol in LDL	303	-0.02	-0.06	0.02	0.427
Total cholesterol in HDL	303	0.03	-0.01	0.07	0.111
Free cholesterol	303	-0.02	-0.07	0.02	0.331
Free cholesterol to esterified cholesterol ratio	303	-0.02	-0.07	0.03	0.339
Serum total triglycerides	303	-0.03	-0.07	0.01	0.091
Total phosphoglycerides	303	-0.02	-0.06	0.03	0.522
Glycerol	303	-0.04	-0.07	0.00	0.075
Glycoproteins	303	-0.02	-0.06	0.02	0.307
Phosphatidylcholine and other cholines	303	-0.01	-0.06	0.03	0.607
Apolipoprotein A-I	303	0.02	-0.02	0.06	0.310
Apolipoprotein B	303	-0.02	-0.06	0.03	0.447
Total fatty acids	303	-0.04	-0.08	0.01	0.123
Description of average fatty acid chain length, not actual carbon number	303	0.02	-0.02	0.07	0.324
22:6, docosahexaenoic acid	303	-0.02	-0.07	0.02	0.339
18:2, linoleic acid (LA)	303	0.00	-0.05	0.05	0.973
Omega-3 fatty acids	303	-0.01	-0.06	0.03	0.609
Omega-6 fatty acids	303	-0.01	-0.06	0.04	0.616
Omega-9 and saturated fatty acids	303	-0.04	-0.09	0.00	0.068
Mono-unsaturated fatty acids	303	-0.04	-0.09	0.01	0.112
Other polyunsaturated fatty acids than 18:2	303	-0.03	-0.08	0.01	0.168
Glucose	303	-0.01	-0.04	0.03	0.686
Lactate	303	-0.02	-0.05	0.02	0.376
Pyruvate	303	-0.03	-0.07	0.00	0.057
Citrate	303	-0.02	-0.06	0.01	0.196
Alanine	303	-0.02	-0.06	0.01	0.163
Glutamine	303	0.03	-0.01	0.07	0.116
Histidine	303	-0.02	-0.06	0.02	0.269
Isoleucine	303	-0.03	-0.07	0.00	0.072
Leucine	303	-0.03	-0.07	0.00	0.075
Valine	303	-0.03	-0.07	0.00	0.074
Phenylalanine	303	-0.05	-0.09	-0.01	0.012
Tyrosine	303	-0.06	-0.09	-0.02	1.72E-03
Acetate	303	-0.02	-0.05	0.02	0.354
Acetoacetate	303	-0.03	-0.08	0.01	0.116
3-hydroxybutyrate	303	-0.04	-0.07	0.00	0.035
Creatinine	303	0.00	-0.04	0.04	0.981
Albumin	303	0.04	0.00	0.07	0.073
Glycoprotein acetyls	303	-0.02	-0.06	0.02	0.345
