# Supplementary material for: Influence of puberty timing on adiposity and cardiometabolic traits: A Mendelian randomisation study
Source: PLoS Med. 2018 Aug 28;15(8):e1002641. doi: 10.1371/journal.pmed.1002641 (PMC6112630; doi:10.1371/journal.pmed.1002641)
Supplement: S15 Table — (PDF) [file pmed.1002641.s034.pdf]

S15 Table Two-sample MR estimates of associations of puberty timing (per year later) with post-pubertal adiposity and cardiometabolic traits among males and females in GWAS data, using a refined set of up to 104 SNPs for age at menarche

Standardised outcome in adulthood	# SNPs	Beta (IVW)	LCL	UCL	P-value
Body mass index	82	-0.16	-0.24	-0.08	4.33E-05
Concentration of chylomicrons and largest VLDL particles	104	-0.01	-0.08	0.05	0.700
Total lipids in chylomicrons and largest VLDL particles	104	-0.03	-0.09	0.04	0.407
Phospholipids in chylomicrons and largest VLDL particles	104	-0.01	-0.07	0.04	0.679
Triglycerides in chylomicrons and largest VLDL particles	104	-0.03	-0.09	0.03	0.348
Concentration of very large VLDL particles	104	-0.04	-0.10	0.02	0.202
Total lipids in very large VLDL	104	-0.03	-0.10	0.03	0.264
Phospholipids in very large VLDL	104	-0.02	-0.07	0.04	0.616
Triglycerides in very large VLDL	104	-0.02	-0.08	0.04	0.476
Concentration of large VLDL particles	104	0.00	-0.06	0.06	0.931
Total lipids in large VLDL	104	0.00	-0.06	0.06	0.997
Phospholipids in large VLDL	104	0.00	-0.06	0.06	0.959
Total cholesterol in large VLDL	104	-0.02	-0.08	0.04	0.617
Cholesterol esters in large VLDL	104	-0.02	-0.08	0.05	0.575
Free cholesterol in large VLDL	104	-0.02	-0.08	0.05	0.624
Triglycerides in large VLDL	104	0.00	-0.06	0.06	0.927
Concentration of medium VLDL particles	104	0.02	-0.05	0.08	0.579
Total lipids in medium VLDL	104	0.00	-0.06	0.07	0.930
Phospholipids in medium VLDL	104	0.00	-0.06	0.06	0.997
Total cholesterol in medium VLDL	104	-0.01	-0.07	0.05	0.826
Cholesterol esters in medium VLDL	104	-0.01	-0.08	0.06	0.699
Free cholesterol in medium VLDL	104	-0.01	-0.07	0.05	0.830
Triglycerides in medium VLDL	104	0.00	-0.06	0.07	0.878
Concentration of small VLDL particles	104	-0.01	-0.07	0.06	0.884
Total lipids in small VLDL	104	-0.01	-0.08	0.06	0.832
Phospholipids in small VLDL	104	0.00	-0.07	0.06	0.888
Total cholesterol in small VLDL	104	-0.02	-0.08	0.04	0.495
Free cholesterol in small VLDL	104	0.00	-0.07	0.06	0.875
Triglycerides in small VLDL	104	0.00	-0.06	0.06	0.945
Concentration of very small VLDL particles	104	-0.03	-0.10	0.03	0.333
Total lipids in very small VLDL	104	-0.03	-0.10	0.03	0.284
Phospholipids in very small VLDL	104	-0.04	-0.10	0.02	0.189
Triglycerides in very small VLDL	104	-0.02	-0.08	0.05	0.625
Concentration of IDL particles	104	-0.04	-0.11	0.02	0.196
Total lipids in IDL	104	-0.04	-0.10	0.02	0.215
Phospholipids in IDL	104	-0.04	-0.10	0.01	0.140
Total cholesterol in IDL	104	-0.04	-0.10	0.03	0.247
Free cholesterol in IDL	104	-0.05	-0.11	0.01	0.092
Triglycerides in IDL	104	-0.04	-0.11	0.03	0.248
Concentration of large LDL particles	104	-0.03	-0.10	0.03	0.327
Total lipids in large LDL	104	-0.03	-0.10	0.04	0.362
Phospholipids in large LDL	104	-0.02	-0.08	0.05	0.600
Cholesterol esters in large LDL	104	-0.02	-0.09	0.05	0.538
Total cholesterol in large LDL	104	-0.01	-0.08	0.05	0.666
Free cholesterol in large LDL	104	-0.03	-0.09	0.03	0.369
Concentration of medium LDL particles	104	-0.02	-0.09	0.04	0.494
Total lipids in medium LDL	104	-0.02	-0.09	0.04	0.473
Phospholipids in medium LDL	104	-0.01	-0.07	0.05	0.769
Total cholesterol in medium LDL	104	-0.02	-0.08	0.05	0.616
Cholesterol esters in medium LDL	104	-0.02	-0.09	0.05	0.538
Concentration of small LDL particles	104	-0.03	-0.09	0.04	0.432
Total lipids in small LDL	104	-0.03	-0.09	0.04	0.382
Total cholesterol in small LDL	104	-0.03	-0.09	0.03	0.265
Concentration of very large HDL particles	104	-0.01	-0.07	0.05	0.808
Total lipids in very large HDL	104	-0.03	-0.08	0.03	0.347
Phospholipids in very large HDL	104	-0.02	-0.08	0.03	0.435
Total cholesterol in very large HDL	104	-0.03	-0.08	0.03	0.348
Cholesterol esters in very large HDL	104	-0.03	-0.08	0.03	0.365
Free cholesterol in very large HDL	104	-0.02	-0.08	0.03	0.459
Triglycerides in very large HDL	104	-0.02	-0.08	0.04	0.469
Concentration of large HDL particles	104	0.01	-0.04	0.07	0.639
Total lipids in large HDL	104	0.01	-0.04	0.07	0.661
Phospholipids in large HDL	104	0.01	-0.05	0.07	0.703
Total cholesterol in large HDL	104	0.02	-0.03	0.08	0.449
Cholesterol esters in large HDL	104	0.02	-0.04	0.07	0.571
Free cholesterol in large HDL	104	0.01	-0.05	0.07	0.719
Concentration of medium HDL particles	104	0.05	-0.01	0.11	0.083
Total lipids in medium HDL	104	0.05	-0.01	0.11	0.092
Phospholipids in medium HDL	104	0.05	-0.01	0.10	0.099
Total cholesterol in medium HDL	104	0.05	0.00	0.11	0.071
Cholesterol esters in medium HDL	104	0.05	-0.01	0.11	0.087
Free cholesterol in medium HDL	104	0.05	-0.01	0.10	0.103

S15 Table Two-sample MR estimates of associations of puberty timing (per year later) with post-pubertal adiposity and cardiometabolic traits among males and females in GWAS data, using a refined set of up to 104 SNPs for age at menarche

Standardised outcome in adulthood	# SNPs	Beta (IVW)	LCL	UCL	P-value
Concentration of small HDL particles	104	0.06	0.00	0.12	0.048
Total lipids in small HDL	104	0.06	0.00	0.12	0.051
Triglycerides in small HDL	104	0.01	-0.05	0.07	0.832
Mean diameter for VLDL particles	104	0.03	-0.03	0.09	0.254
Mean diameter for LDL particles	104	-0.03	-0.09	0.03	0.357
Mean diameter for HDL particles	104	-0.01	-0.07	0.05	0.691
Serum total cholesterol	104	-0.01	-0.07	0.05	0.683
Total cholesterol in LDL	104	-0.02	-0.08	0.04	0.470
Total cholesterol in HDL	104	0.03	-0.03	0.08	0.362
Free cholesterol	104	-0.03	-0.11	0.04	0.389
Free cholesterol to esterified cholesterol ratio	104	-0.02	-0.09	0.05	0.506
Serum total triglycerides	104	-0.01	-0.08	0.05	0.659
Total phosphoglycerides	104	-0.03	-0.10	0.04	0.464
Glycerol	104	0.01	-0.04	0.07	0.613
Glycoproteins	104	0.05	-0.01	0.10	0.127
Phosphatidylcholine and other cholines	104	-0.02	-0.09	0.06	0.660
Apolipoprotein A-I	104	0.02	-0.04	0.08	0.533
Apolipoprotein B	104	-0.01	-0.08	0.05	0.715
Total fatty acids	104	-0.05	-0.13	0.02	0.155
Description of average fatty acid chain length, not actual carbon number	104	0.05	-0.02	0.12	0.182
22:6, docosahexaenoic acid	104	-0.02	-0.10	0.05	0.537
18:2, linoleic acid (LA)	104	-0.02	-0.09	0.05	0.556
Omega-3 fatty acids	104	0.01	-0.06	0.08	0.811
Omega-6 fatty acids	104	-0.04	-0.11	0.03	0.248
Omega-9 and saturated fatty acids	104	-0.05	-0.13	0.02	0.178
Mono-unsaturated fatty acids	104	-0.03	-0.11	0.05	0.428
Other polyunsaturated fatty acids than 18:2	104	-0.02	-0.09	0.05	0.579
Glucose	104	0.00	-0.05	0.06	0.864
Lactate	104	-0.01	-0.06	0.04	0.804
Pyruvate	104	-0.02	-0.07	0.03	0.386
Citrate	104	-0.02	-0.07	0.04	0.566
Alanine	104	0.01	-0.04	0.06	0.638
Glutamine	104	0.07	0.02	0.12	0.007
Histidine	104	-0.01	-0.07	0.06	0.837
Isoleucine	104	-0.01	-0.07	0.04	0.684
Leucine	104	-0.02	-0.08	0.03	0.427
Valine	104	-0.02	-0.07	0.03	0.406
Phenylalanine	104	-0.04	-0.10	0.01	0.146
Tyrosine	104	-0.03	-0.08	0.03	0.326
Acetate	104	-0.02	-0.08	0.03	0.385
Acetoacetate	104	-0.03	-0.10	0.03	0.276
3-hydroxybutyrate	104	-0.07	-0.12	-0.02	0.011
Creatinine	104	0.00	-0.05	0.06	0.884
Albumin	104	0.04	-0.02	0.10	0.154
Glycoprotein acetyls	104	-0.02	-0.09	0.04	0.469
